# Differentially expressed full-length, fusion and novel isoforms transcripts-based signature of well-differentiated keratinized oral squamous cell carcinoma

**DOI:** 10.18632/oncotarget.27693

**Published:** 2020-08-25

**Authors:** Neetu Singh, Dinesh Kumar Sahu, Ratnesh Kumar Tripathi, Archana Mishra, Hari Shyam, Pratap Shankar, Mayank Jain, Nawazish Alam, Anil Kumar, Abhishek Mishra, Rebecca Chowdhry, Anjana Singh, Sameer Gupta, Divya Mehrotra, Preeti Agarwal, Madhu Mati Goel, Arun Chaturvedi, Satya Prakash Agarwal, Manish Bajpai, Devendra Kumar Gupta, Madan Lal Brahma Bhatt, Ravi Kant

**Affiliations:** ^1^Department of Molecular Biology, Center for Advance Research, King George's Medical University, Lucknow, India; ^2^Department of Surgery, King George’s Medical University, Lucknow, India; ^3^Department of Periodontology, All India Institute of Medical Sciences, Rishikesh, India; ^4^Department of Biochemistry, All India Institute of Medical Sciences, Rishikesh, India; ^5^Department of Surgical Oncology, King George's Medical University, Lucknow, India; ^6^Department of Oral and Maxillofacial Surgery, King George's Medical University, Lucknow, India; ^7^Department of Pathology, King George's Medical University, Lucknow, India; ^8^Department of Otorhinolaryngology, King George's Medical University, Lucknow, India; ^9^Department of Physiology, King George's Medical University, Lucknow, India; ^10^Department of Pediatric Surgery, Super Speciality Pediatric Hospital and Post Graduate Teaching Institute, Noida, India; ^11^Department of Radiotherapy, King George's Medical University, Lucknow, India; ^12^Department of Surgical Oncology, All India Institute of Medical Sciences, Rishikesh, India; ^*^These authors contributed equally to this work

**Keywords:** oral tongue squamous cell carcinoma, microarray, transcriptomics, integrative bioinformatics, differentially expressed gene

## Abstract

Highly keratinized oral squamous cell carcinoma (OSCC) exhibits an improved response to treatment and prognosis compared with weakly keratinized OSCC. Therefore, we aimed to develop gene transcript signature and to identify novel full-length isoforms, fusion transcript and non-coding RNA to differentiate well-differentiated (WD) with Moderately Differentiated (MD)/Poorly Differentiated (PD)/WD-lymphadenopathy OSCC through, HTA, Isoform sequencing, and NanoString. Additionally, specific copy number gain and loss were also identify in WD keratinized OSCC through Oncoscan array and validated through Real-time PCR in histopathologically characterized FFPE-WD keratinized OSCC. Three-hundred-thirty-eight (338) differentially expressed full-length (FL) transcript isoforms (317 upregulated and 21 down-regulated in OSCC) were identified through Isoform Sequencing using the PacBio platform. Thirty-four (34) highly upregulated differentially expressed transcripts from IsoSeq data were also correlated with HTA2.0 and validated in 42 OSCC samples. We were able to identify 18 differentially expressed transcripts, 12 fusion transcripts, and two long noncoding RNAs. These transcripts were involved in increased cell proliferation, dysregulated metabolic reprogramming, oxidative stress, and immune system markers with enhanced immune rearrangements, suggesting a cancerous nature. However, an increase in proteasomal activity and hemidesmosome proteins suggested an improved prognosis and tumor cell stability in keratinized OSCC and helped to characterize WD with MD/PD/WD with lymphadenopathy OSCC. Additionally, novel isoforms of IL37, NAA10, UCHL3, SPAG7, and RAB24 were identified while in silico functionally validated SPAG7 represented the premalignant phenotype of keratinized (K4) OSCC. Most importantly we found copy number gain and overexpression of EGFR suggest that TKIs may also be used as therapeutics in WD-OSCCs.

## INTRODUCTION

The oral cavity includes the lips, the inner lining of the lips and cheeks (buccal mucosa), the teeth, the gums, the front two-thirds of the tongue, the floor of the mouth below the tongue, and the bony roof of the mouth (hard palate). Different parts of the oral cavity are composed of several types of cells. However, keratinizing lesions may occur in any of the cell types in the oral cavity and may be initiated due to defects in keratinization, including reactive, preneoplastic and neoplastic lesions. Keratins (KRTs) are important differentiation markers both in normal, keratinized and neoplastic oral squamous cell carcinoma (OSCC). So, the WHO has categorized OSCC into four grades: Grade I (well-differentiated; > 50% cellular keratinizaion-K4), Grade II (moderately differentiated; 20-50% keratinization-K3), Grade III (poorly differentiated; 5–20% keratinization-K2) and Grade IV (undifferentiated; 0–5% keratinization-K0-K1) [1].

OSCC patients are regarded as harboring tumors with various degree of keratinization (K0 to K4), which plays an important role in the prognosis of OSCC. Keratinized OSCC plays an important role in the prognosis of OSCC. A recent study demonstrated that patients with low degree of keratinization have an increased recurrence of disease, a high propensity for early metastasis to regional lymph nodes and a reduced incidence of 5-yr disease-free survival rates [2]. However, there are very few studies reported the degree of keratinization is the prognostic and risk factors for OSCC [3]. Notably, patients with both a high degree of keratinization and human papillomavirus (HPV)-positive oral cancers have an improved response to treatment and an improved prognosis compared with patients with a low degree of keratinization and HPV-negative OSCCs [4]. In addition to keratinization, the proliferation index (Ki-67), vascularization (CD34), p53 and bcl-2 expression and HPV are also used to evaluate the prognosis of OSCC [5].

A recent report identified two sets of transcripts relevant to diagnosis and therapeutics of oral tongue squamous cell carcinoma: one set was associated with the extracellular matrix (ECM), and another set was associated with HPV and the altered expression of hypo- and hypermethylated oncogenes and tumor suppressor genes [6]. For prognosis, HMGA2 expression has been identified as an independent prognostic factor related to epithelial-to-mesenchymal transition (EMT) in undifferentiated OSCC [7]. Additionally, large and growing public databases of oral cancer transcriptome sequencing data (RNA-Seq) are available [8]. However, the above studies have been conducted either through a probe-based approaches or short-read sequencing methods. These approaches are unable to provide full-length (FL) transcript sequences, which required to identifying novel tumor-specific isoforms and fusion genes. Various studies have been reported the involvement of tumor-specific isoforms and fusion gens in pathogenesis and progression of cancer [1, 9–12]. Furthermore, studies suggest that the epidermal growth factor receptor (EGFR) may be a promising target for therapy of OSCC and EGFR overexpression is associated with worse prognosis of the disease [13, 14]. Therefore, the aim of this study to characterize keratinized OSCCs to identify differentially expressed Full-Length (FL) transcript isoforms, novel FL transcript isoforms, fusion genes and gene expression-based signatures that could help for the diagnosis, prognosis and targeted therapeutics of the disease.

## RESULTS

**Figure 1:**
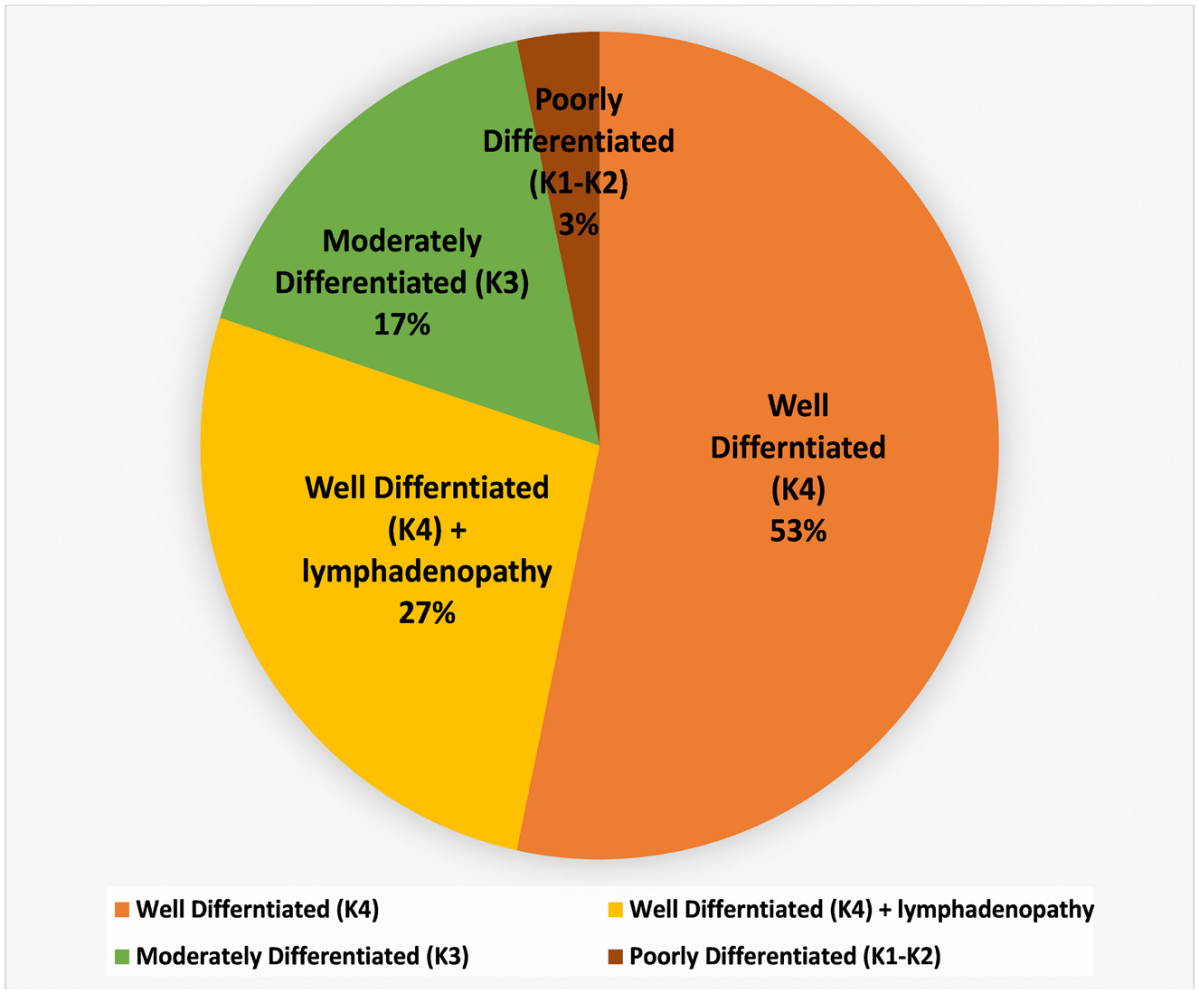
Details of keratinized OSCC collected from different anatomical sites (buccal mucosa; tongue and alveolus) of the oral cavity. Histopathological classification, level of differentiation, and involvement of node have also been included.

The study was carried out in 30 Oral Squamous Cell Carcinoma (OSCC) patients based on the degree of keratinization. According to the WHO grading system of OSCC, we enrolled 24 well-differentiated; > 50% cellular keratinization (K4) including 8 unilateral/bilateral lymphadenopathy with metastatic features, 5 moderately differentiated; 20–50% keratinization (K3) and 1 poorly differentiated; 5–20% keratinization (K2) as shown in Figure 1, Supplementary Table 1A. Eight healthy volunteers were recruited as oral control in Supplementary Table 1B.

### Differential expression of genes through human transcriptome array (HTA)

**Figure 2:**
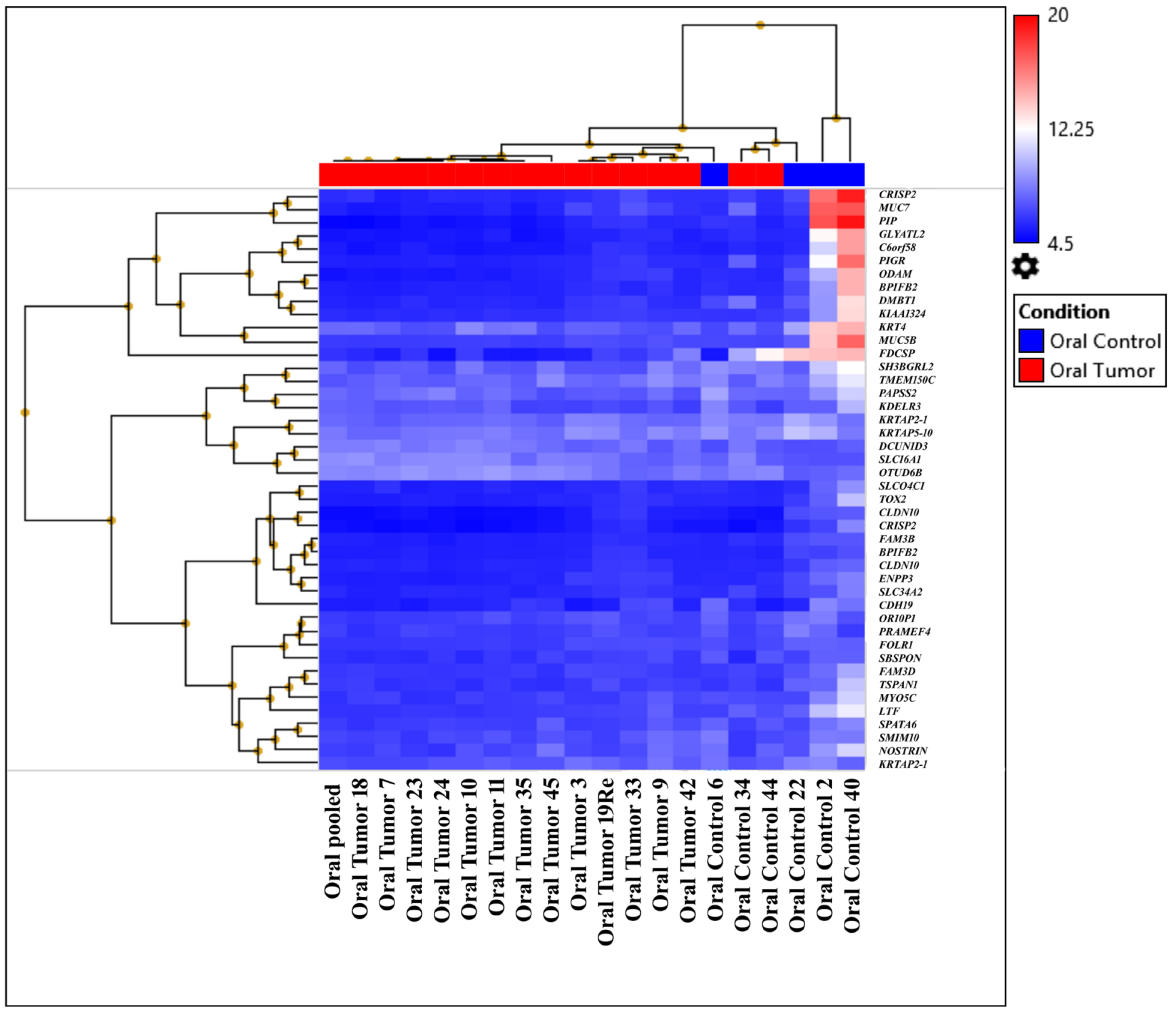
Heat map created based on the expression patterns of each gene across tumor and control samples. The samples were clustered into two subgroups distinct from their normal counterparts by hierarchical clustering (first subgroup: OT-19, OT-3, OT-35, OT-7, OT-23, OT-24, OT-11, OT-pooled, OT-18, OT-10, and OT-34; second subgroup: OT-42, OT-9, OT-45, OT-44, and OT-33).

Analysis of HTA data was performed using strict statistical criteria as defined in the "Materials and Methods" section to detect the differentially expressed coding and noncoding transcripts. At gene level 44 highly significant differentially expressed coding transcript were identified in which 3 were up-regulated and 41 are down-regulated represented in hierarchical clustering of oral tumor (OT) group (first subgroup: OT-19, OT-3, OT-35, OT-7, OT-23, OT-24, OT-11, OT-pooled, OT-18, OT-10, and OT-34; second subgroup: OT-42, OT-9, OT-45, OT-44, and OT-33) (Figure 2, Supplementary Table 7A). Differential pathway analysis revealed the downregulation of amino acid conjugation of benzoic acid; sulfation biotransformation reaction; miscellaneous transport and binding events; and photodynamic therapy-induced HIF-1 survival signaling pathways (Supplementary Table 7B). The samples from the first subgroup and histopathologically characterized keratinized OSCCs from different sites, OT-10, OT-11, OT-18, OT-19, OT-23, and OT-24, were pooled. Additionally, the pooled sample was also processed with HTA2.0 and placed in the first cluster (Figure 2). The analysis at exonic level, 2 genes (SLC2A1, PTHLH) were upregulated, and 6 genes (MUC5B, ODAM, HTN1, AGR2, PIGR, CRISP3) were downregulated. The tumor samples showed significant (*p*-value ≤ 0.001) upregulation and downregulation in different signaling pathways as shown in Supplementary Table 7C–7E.

### Functional annotation and identification of high-quality FL transcripts

**Figure 3:**
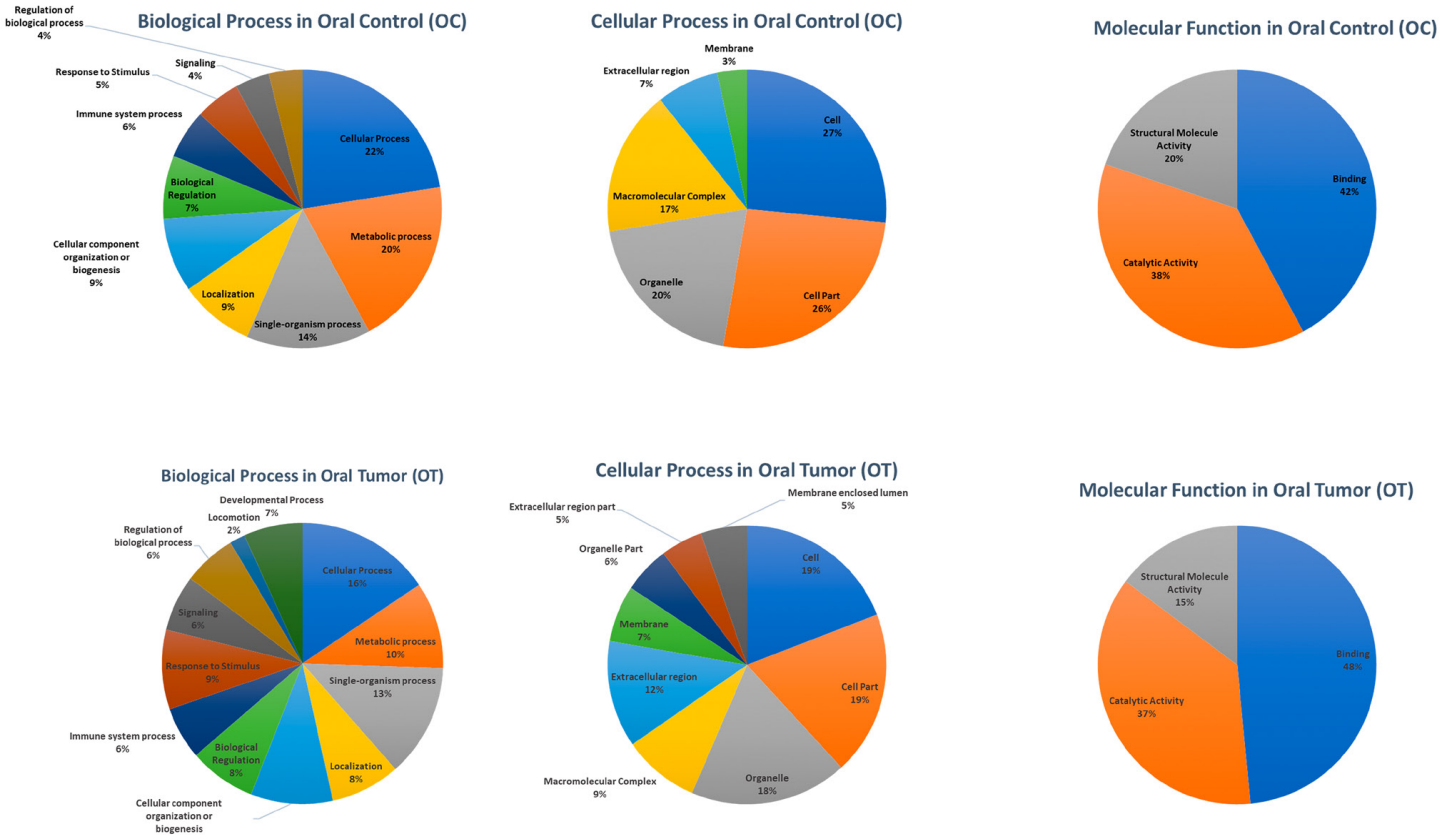
The number of sequences (GO term) involved in different subgroups under Biological Process, Cellular Process, and Molecular function in Level 2 after processing 20, 600, and 10, 637 high-quality full-length transcripts in OC and OT, respectively, through Blast2Go.

Based on HTA analysis, samples were pool as described in the above section. Subsequently, pool-OT and pool-OC samples were processed for IsoSeq analysis. The identified 20,600 and 10,637 high-quality FL transcripts in oral control (OC) and OT, respectively, were annotated and classified with Blast2GO (Table 1). The number of sequences (Gene Ontology [GO] terms) involved in different subgroups under the categories biological process, the cellular process and molecular function in level 2 was identified and is shown in Figure 3. A total of 20,600 and 10,637 high-quality FL transcripts in OC and OT, respectively, had significant BLASTX hits corresponding to 9,620 and 10,036 unique protein accessions in OC and OT, respectively, in the non-redundant (nr) protein database. GO analysis of these 9,620 (OC) and 10,036 (OT) unique proteins resulted in a total of 41,457 and 102,682 annotations/GO terms in OC and OT, respectively, including 17,439 (42.06%) terms from OC and 48,465 (47.20%) terms from OT in biological process, 16,726 (40.34%) terms from OC and 44,579 (43.41%) terms from OT in cellular component and 7,292 (17.58%) terms from OC and 9,638 (9.38%) terms from OT in molecular function. Among the biological process terms, 3,405 and 4,820 genes from OC and OT, respectively, were related to the metabolic process (GO: 0008152), and 3,918 and 7,548 genes from OC and OT, respectively, were involved in the cellular process (GO: 0009987). Similarly, under the cellular component category, 4,447 genes from OC and 8,530 genes from OT were classified as a cell (GO: 0005623), whereas in the cell part (GO: 0044464) subcategory, 4,374 genes from OC and 8,486 genes from OT were the most represented categories. Under the molecular function category, 3,073 genes from OC and 4,673 genes from OT were involved in the binding process (GO: 0005488), and 2,775 genes from OC and 3,541 genes from OT were involved in the catalytic activity (GO: 0003824) subcategory (Figure 3).

**Table 1:** Statistics of full length (FL) consensus isoforms after processing through Oral Control (OC) and Oral Tumor (OT) classified reads after polishing, error correction, clustering as mentioned in "Materials and Methods" section

Transcript Classification Analysis metrics of OC and OT		
**Analysis Metric**	**Value in OC**	**Value in OT**
Number of consensus reads	411,798	204,341
Number of five prime reads	335,914	161,179
Number of three prime reads	336,104	165,275
Number of poly-A reads	293,532	153,157
Number of filtered short reads	231	45
Number of non-full-length reads	152,394	69,390
Number of full-length reads	259,173	134,906
Number of full-length non-chimeric reads	255,047	116,273
Number of full-length non-chimeric bases	254,365,817	115,475,416
Mean full-length non-chimeric read length	997	993
Transcript Clustering results in oral control		
**Analysis Metric**	**Value in OC**	**Value in OT**
Number of unpolished consensus isoforms	144,929	65,823
Number of polished high-quality isoforms	20,600	10,637
Number of polished low-quality isoforms	124,072	55,109
Mean unpolished consensus isoform read length	1,013	1,003

Further, the Kyoto Encyclopedia of Genes and Genomes (KEGG) pathway analysis was performed using the bidirectional best hit (BBH) method (Moriya *et al*., 2007) on the high-quality FL transcripts in OC and OT, respectively, as an alternative approach for functional categorization and annotation. Enzyme Commission (EC) numbers were obtained and putatively mapped for protein sequences to a specific biochemical pathway (Supplementary Table 2).

### Identification of differentially expressed transcripts and fusion gene using the high-quality isoform sequencing data

For differentially expressed genes between the pooled-OT and pooled-OC samples, GFOLD (*p* = 0.01; specifically designed for data without biological replicates to search) was used. The analysis yielded 338 differentially expressed FL transcripts with a threefold cutoff (317 upregulated and 21 downregulated in the pooled-OT sample; Supplementary Table 3). Further, the differentially expressed transcripts identified *via* GFOLD analysis were compared with HTA data of differentially expressed isoforms, and most of the transcripts were validated (Supplementary Table 3). We also identified novel intrachromosomal Ch12 fusion between KRT6B–KRT6A and interchromosomal fusions between CKB-Ch14 and CKM-Ch19, ACTB-Ch7–ACTA2-Ch10, ACTB-Ch7–ACTC1-Ch15, ACTB-Ch7–ACTG2-Ch2 and IGKV1-27–IGKV3-15.

### Validation of upregulated and fusion transcripts through NanoString nCounter Platform

Validation of upregulated and fusion transcripts in 42 tumor samples (15 histopathologically characterized FFPE keratinized tumor samples, 27 keratinized OSCC samples, and four control samples) were performed on the NanoString nCounter platform. Among 34 transcripts, 16 gene transcripts showed more than 50% expression, while 18 gene transcripts showed more than 20% and less than 50% expression in keratinized OSCC compared to control samples (Supplementary Table 4A). These genes were involved in 467 different pathways when subjected to Reactome pathway analysis, of which the 25 most relevant pathways sorted by *p*-value are shown in Supplementary Table 4B. Specific pathways including Wnt (PSMB6, PSMD8, PRDX5, PSMC5, UBB), Hedgehog (PSMB6, PSMD8, PSMC5, UBB), the formation of the cornified envelope and type I hemidesmosome assembly (KRT14, KRT16, KRT17, laminin-5 γ2 [LAMC2]) and the assembly of collagen fibrils and other multimeric structures (LAMC2) were also upregulated. Fusions of ACTB–ACTC1, ACTB–ACTG2, IGKV1-27–IGKV3-15, and KRT6B–KRT6A were expressed both in OC and FFPE OT samples. Additionally, reported fusions in CFLAR-NDUFB3, GLIS3-CTNNA2, and SFN-ELK3 were not identified in our samples. Of the three-long noncoding RNAs, NR_037633.1, NR_037926.1, and NR_027166.1, only NR_027166.1 was positive in 97.67% of samples, including both OC and OT (Supplementary Table 4C). No significant differential expression of fusions was observed among WD FFPE OT samples and 38 MD/PD/WD keratinized (K2-K4) OSCC samples with unilateral/bilateral lymphadenopathy.

**Figure 4:**
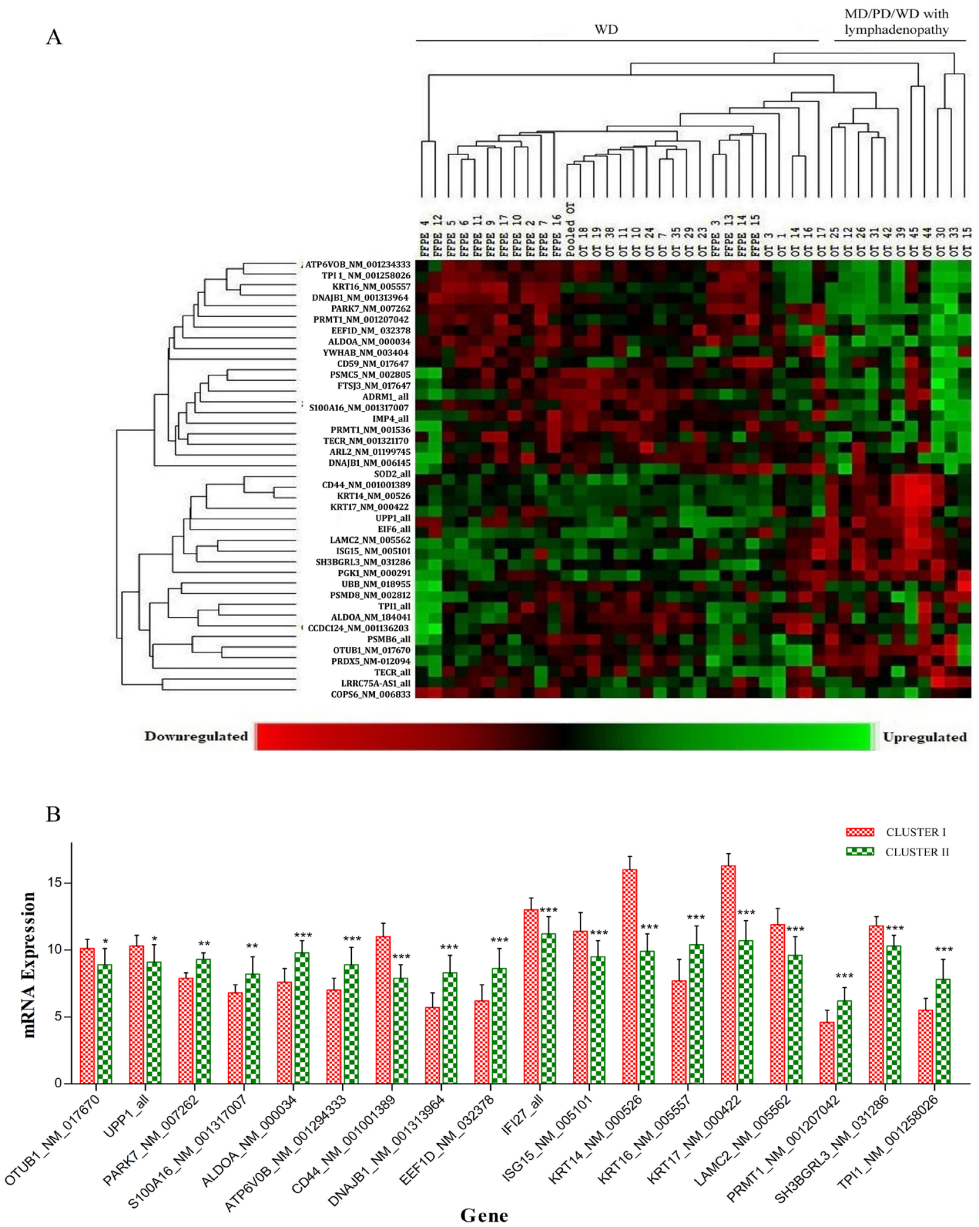
(**A**) Based on unsupervised clustering significantly differentially regulated transcripts between keratinized WD and keratinized MD/PD/WD with metastatic lymphadenopathy groups. The first cluster including 31 WD and 3 MD tumors with no lymph node involvement. Second cluster included 2 MD and 1 PD with no lymph node involvement including 8 WD tumors with metastatic lymphadenopathy were clustered. (**B**) of 34 transcripts, 18 transcripts were significantly expressed between the keratinized WD and keratinized MD/PD/WD-metastatic lymphadenopathy groups.

Based on unsupervised clustering, 34 transcripts (with isoforms of TPI1 and TECR) and 5 housekeeping genes were distributed among keratinized OSCCs at different levels of differentiation, i.e., K2-K4, including 15 WD FFPE (K4), 16 WD (K4), 5 MD (K3), 1 PD (K2), and 8 WD (K4) samples with unilateral/bilateral lymphadenopathy with metastatic features. The samples were grouped into two clusters. The first cluster included 31 WD (K4) and 3 MD (K3) tumors with no lymph node involvement. In the second cluster, 2 MD (K3) and 1 PD (K2) with no lymph node involvement including 8 WD (K4) tumors with metastatic lymphadenopathy were clustered (Figure 4A). Of 34 transcripts, 18 transcripts were significantly expressed between the keratinized WD and keratinized MD/PD/WD-metastatic lymphadenopathy groups (Figure 4B).

### Identification and validation of novel isoforms

**Figure 5:**
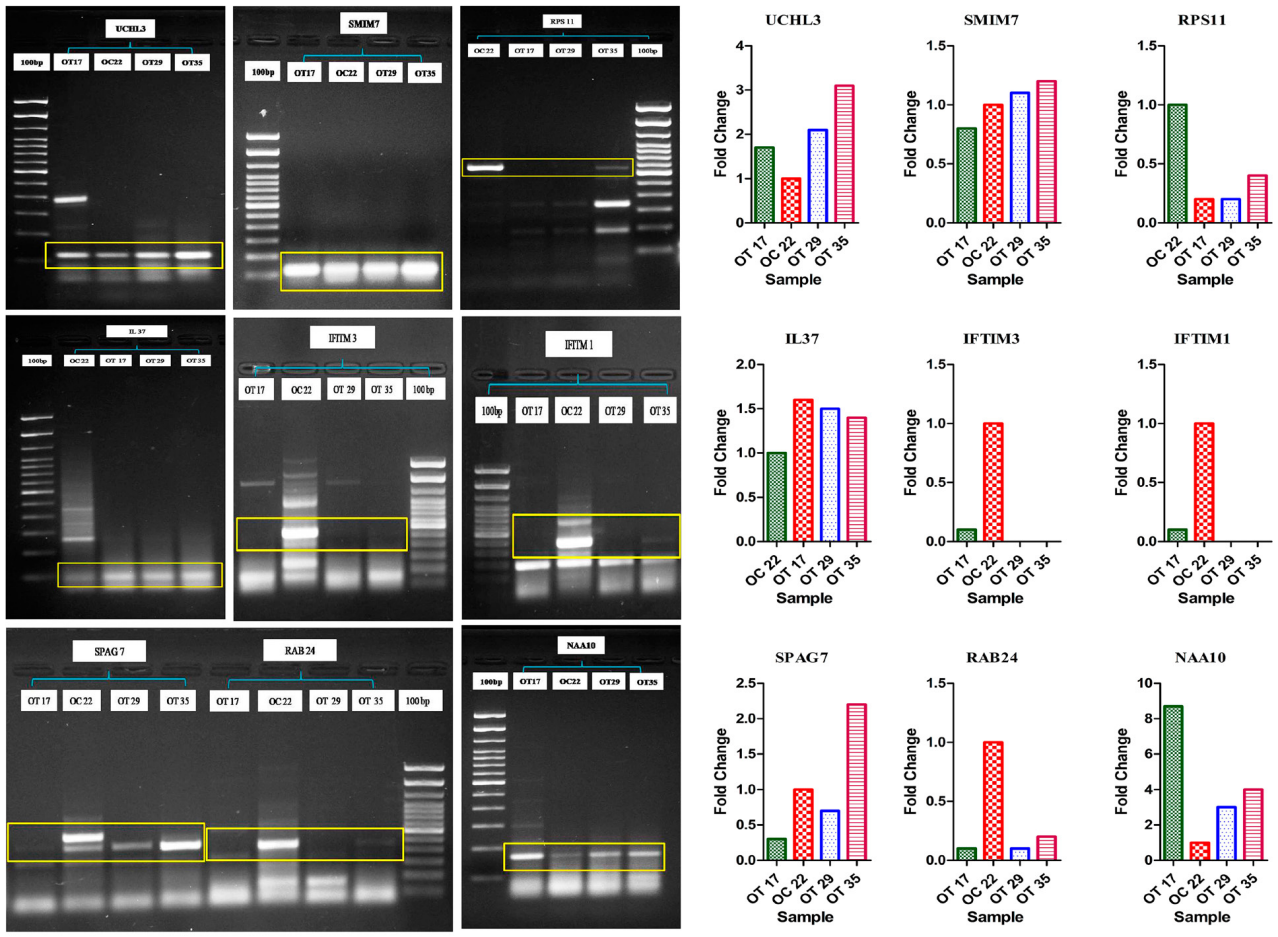
PCR-based validation of RAB24 (missing exon in OT), SPAG7 (fused exon in OT), UCHL3 (inserted exons in OT), IL37 (inserted exons in OT), NAA10 (inserted exons in OT) transcript isoforms.

Full-length transcript isoforms on multiple alignments represented inserted, deleted or fused exonic nucleotide sequences in the coding regions of pooled-OT samples (Supplementary Table 5). After comparison with the ISOexpresso database, we identified 30 isoforms in keratinized OSCC samples that were not reported earlier, while other isoforms of the same transcripts showed differential expression in OSCC versus the normal controls (Supplementary Table 6). For the validation of identified novel transcript isoforms, we performed convention RT-PCR for each inserted/missing/fused exon in pooled-OT and pooled-OC samples. Out of thirty isoforms, 9 novel isoforms including RAB24 (missing an exon in OT), SPAG7 (fused exon in OT), IFITM1 (fused exons), IFITM3 (fused exons), RPS11 (fused exons), UCHL3 (inserted exons in OT), IL37 (inserted exons in OT), NAA10 (inserted exons in OT), and SMIM7 (inserted exons in OT) were validated. We further validated these isoforms in three WD tumors, OT17, OT29, and OT35, and OC22, control sample. RAB24, NAA10, UCHL3, and IL-37 were validated in all three tumors; SPAG7 was validated in two OT compared to the control sample. Remaining 21 novel isoforms were not validated (Figure 5).

### *In silico* functional validation of novel transcript isoforms

For functional validation, we analyzed the physiochemical properties and secondary structures of these isoforms *via* comparison with wild-type (reference data set from NCBI). We predicted the physicochemical properties of IL37, RAB24, NAA10, SPAG7 and UCHL3 wild-type and their novel isoforms by ProtParam and ProtScale (Supplementary Table 8A). Besides, the total hydropathicities of the wild-type and novel proteins as displayed in figure (Supplementary Figure 1A–1E). The results suggest that wild-type and novel proteins are hydropathical molecules, and except SPAG7 novel isoform, RAB24, NAA10, UCHL3, IL-37 novel isoforms were less stable than the wild-type. Secondary structures of the RAB24, NAA10, UCHL3, IL-37, and SPAG7 wild-type and their novel isoform were predicted by DNAStar Protean and online tool CFSSP. The results showed that the proportions of different types of secondary structures in wild-type and novel-isoform derived proteins were different than wild-type (Supplementary Table 8B).

### Identification of copy number variations (CNVs)

**Figure 6:**
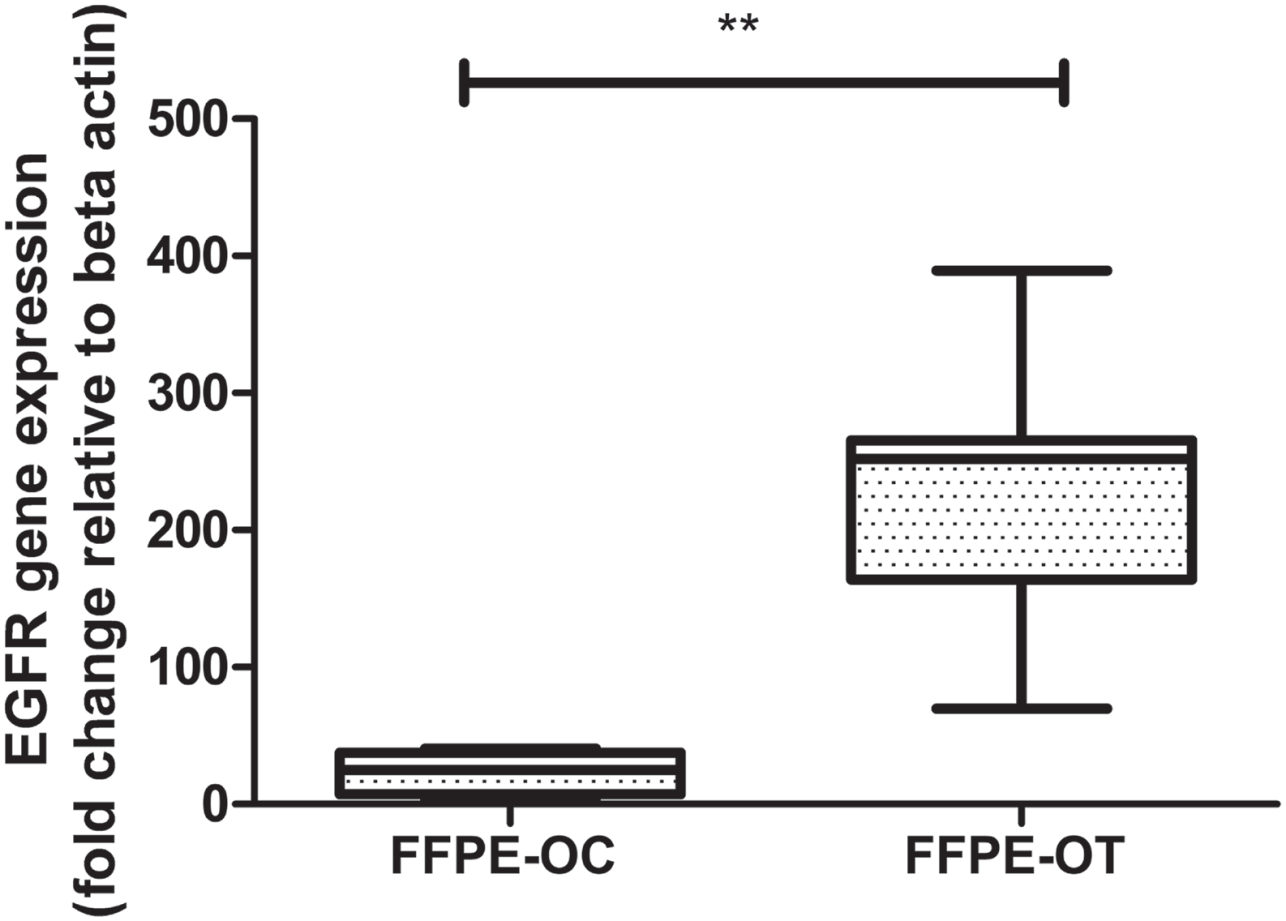
Real-time PCR based validation of EGFR expression in 12 histopathologically characterized FFPE keratinized OSCC by comparison to 5 OC samples using β-actin as an endogenous control.

OT-10, OT-11, OT-18, OT-19, OT-23, OT-24, pooled-OT, OC-2, OC-6, and OC-22 were processed on the OncoScan array (Supplementary Figure 2, Table 2 showing CNVs and sequence variants). By aggregate analysis of pooled oral cancer samples, we identified a significant copy number gain of Ch7p11.2 (EGFR-100% CNV overlap and 81.81% frequency) and copy number loss of Ch3p21.1 (PBRM1-25.46% CNV overlap and 54.54% frequency), Ch3p14.2 (FHIT-0.32% CNV overlap and 54.54% frequency), Ch19p13.3 (STK11-100% CNV overlap and 45.45% frequency) and Ch16 p13.3 (TSC2-79.73% CNV overlap and 45.45% frequency). After further validation, we detected EGFR amplification in 12 histopathologically characterized formalin-fixed, paraffin-embedded (FFPE) keratinized OSCCs, while EGFR amplification was detected in only 5 OC samples (Figure 6).

**Table 2:** Aggregate analysis of (OT-10, OT-11, OT-18, OT-19, OT-23 and OT-24) and control (OC-2, OC-6, OC-22) samples along with other OSCC and control samples processed on Oncoscan array, we identified significant copy number gain and copy number loss showing CNV overlap and frequency

Region	Region Length	Cytoband Location	Event	Genes	miRNAs	Frequency %	*p*-value	% of CNV Overlap	Count of Gene Symbols	Cancer Gene Census-Sanger.txt
chr2:89,138,631-89,389,171	250540	p11.2	CN Gain	0	0	36.36364	0.002	100	0	
chr3:52,631,633-52,741,160	109527	p21.1	CN Loss	9	0	54.54545	0.048	0.318643	9	PBRM1
chr3:60,380,259-60,571,437	191178	p14.2	CN Loss	1	0	54.54545	0.048	25.46527	1	FHIT
chr7:55,114,950-55,124,319	9369	p11.2	CN Gain	1	0	81.81818	0.004	100	1	EGFR
chr9:21,903,166-22,027,402	124236	p21.3	CN Loss	4	0	36.36364	0.002	100	4	
chr9:22,028,315-22,140,224	111909	p21.3	CN Loss	1	0	36.36364	0.002	100	1	
chr11:69,508,372-70,096,585	588213	q13.3	CN Gain	7	0	72.72727	0.002	7.257745	7	
chr11:70,120,785-70,158,876	38091	q13.3	CN Gain	2	1	72.72727	0.002	11.77969	2	
chr11:70,172,204-70,289,645	117441	q13.3	CN Gain	2	0	72.72727	0.002	39.32443	2	
chr11:70,295,897-70,420,282	124385	q13.3–q13.4	CN Gain	1	0	72.72727	0.002	0	1	
chr12:132,982,204-133,331,537	349333	q24.33	CN Loss	8	0	36.36364	0.001	32.73724	8	
chr16:1,154,125-1,486,352	332227	p13.3	CN Loss	12	0	45.45455	0.01	100	12	
chr16:1,790,665-2,305,328	514663	p13.3	CN Loss	50	1	45.45455	0.01	79.73334	50	TSC2
chr16:88,846,849-88,874,778	27929	q24.3	CN Loss	2	0	36.36364	0	30.77804	2	
chr19:1,225,825-1,259,625	33800	p13.3	CN Loss	4	0	45.45455	0.038	100	4	STK11
chr21:46,869,264-47,061,267	192003	q22.3	CN Loss	4	0	63.63636	0.003	91.45586	4	
chr21:47,155,316-47,841,692	686376	q22.3	CN Loss	15	0	63.63636	0.003	13.00585	15	
chr22:22,266,808-22,289,397	22589	q11.22	CN Gain	1	0	36.36364	0.013	100	1	
chrX:177,942-2,686,899	2508957	p22.33	CN Gain	24	0	63.63636	0	29.06012	24	CRLF2, P2RY8
chrX:154,479,421-154,929,412	449991	q28	CN Gain	26	2	45.45455	0.008	100	26	
chrX:154,979,673-155,219,364	239691	q28	CN Gain	2	0	63.63636	0	97.69119	2	

## DISCUSSION

Heterogeneity, such as high and low degrees of keratinization in OSCC, can be well characterized through transcriptome phenotypes. Most therapeutics are based on phenotypes rather than on genotypes; thus, an interesting hypothesis is that specific transcript isoform expression patterns could define keratinization phenotypes.

Cell proliferative markers such as mitotic cell cycle (PSMB6, PSMD8, PSMC5, UBB), late cytokinetic (CCDC124), generic transcription (PSMB6, PSMD8, PRDX5, PSMC5, UBB), rRNA processing in the nucleolus and cytosol (IMP4, FTSJ3), eukaryotic translation elongation (EIF6, EEF1D), posttranslational modification (PARK7, SUMOylation), pyrimidine salvage (UPP1), and the ubiquitin-proteasome system (UPS), which manages hundreds of different proteins and participates in the regulation of almost every cellular process, including cell cycle control, gene transcription, DNA repair, and apoptosis induction, were the prominent features of keratinized OSCC (Supplementary Table 5A). UPS includes deubiquitinases such as PSMB6, PSMD8, PSMC5, UBB, ADRM1, and OTUB1, which catalyze the removal of ubiquitin moieties from target proteins or polyubiquitin chains, resulting in altered signaling or changes in protein stability. Importantly, it has been reported that tumor cells show high proteasome activity and the subsequent inhibition of deubiquitinase is a promising cancer therapeutic strategy. However, low proteasome activity has been reported in radio-resistant human head and neck cancer cell lines, in patients with poor overall survival [15] and CSCs/progenitor cells [16]. Hence, the overexpression of UPS in keratinized OSCC suggests a promising cancer therapeutic target (Supplementary Table 5A).

However, the upregulation of PRMT1 and EEF1D in MD/PD/WD metastatic OSCC compared to WD OSCC suggests that PRMT1-dependent-C/EBPα-methylation/cyclin D1 expression and Akt-mTOR/Akt-bad signaling mediated enhanced cell proliferation, respectively [17, 18]. The upregulation of S100A16 expression in MD/PD/WD-metastatic OSCC compared to WD OSCC suggests increased EMT *via* the Notch1 pathway [19]. OTUB1- and UPP1-mediated low proteasomal activity may make MD/PD/WD-metastatic OSCC relatively more resistant to chemotherapeutics and cause poor overall survival.

In addition to the UPS system, the ISGylation of activated proteins [20] was also enhanced, as suggested by the upregulation of the ISG15 ubiquitin-like modifier (ISG15) in keratinized OSCC (Supplementary Table 6), and, as indicated by its differentiation, ISG15 was overexpressed in keratinized WD OSCC compared to MD/PD/WD-metastatic OSCC. This ISGylation may induce natural killer cell proliferation, which acts as a chemotactic factor for neutrophils and acts as an IFN-gamma-inducing cytokine. IFN-gamma subsequently modulates innate immunity by NFkB, JNK, and IRF-3 cell signaling pathways. Further, the overexpression of IFI27 interferon alpha-inducible protein 27 (IFI27) in keratinized WD OSCC compared to MD/PD/WD-metastatic OSCC suggests the downregulation of the immune system in latter [21].

Another alternative proteasome pathway mediated by COSP6 that was overexpressed in keratinized OSCC (Supplementary Table 6), which modulates transcription-coupled nucleotide excision repair (TC-NER) and vesicle-mediated transport (gene card), was unchanged at the level of differentiation. Both UPS and alternative proteasome pathways modulate multiple signaling pathways, such as ATP6V0B, which subsequently enhances signaling by the insulin receptor, and UBB, which mediates the TGF-β receptor complex. Both ATP6V0B and UBB were upregulated in keratinized OSCC (Supplementary Table 5B). Further enhancement of ATP6V0B in MD/PD/WD metastatic OSCC compared to WD OSCC suggested enhanced signaling by the insulin receptor in the former.

Two additional hallmarks of cancer metabolic reprogramming, ALDOA, and PGK1, were upregulated in keratinized OSCC, as indicated by increased glycolysis and metabolic reprogramming, which creates oncogenic stress [22, 23], as suggested in other solid tumors, such as non-small cell lung cancer (NSCLC) [24]. Oncogenic stress is balanced by mitochondrial biogenesis (PSMB6, PSMD8, DNAJB1, PRDX5, PSMC5, UBB, SOD2) and deregulated CDK5. Enhanced expression of PSMB6, PSMD8, DNAJB1, PRDX5, PSMC5, UBB, and SOD2 in keratinized OSCC supports oncogenic stress (Supplementary Table 6). However, significant enhancement of ALDOA, TPI1, and DNAJB1 in MD/PD/WD metastatic OSCC compared to WD OSCC suggests enhanced cancer cell proliferation, metabolic reprogramming and oncogenic stress in the former.

The overexpression of another metabolic marker, triosephosphate isomerase 1 (TPI1), a tumor suppressor, was observed in keratinized OSCC (Supplementary Table 5A), suggesting a decline in tumor growth, as suggested in a previous study on hepatocellular carcinoma [25].

Another marker, PARK7, a positive regulator of AKT, stimulates HIF-1-mediated transcriptional activity, promoting the transcription of angiogenic factors, glucose transporters, and glycolytic enzymes [26]. Enhanced expression of PARK7 in keratinized OSCC compared to control samples supported tumor development (Table 6A), which corroborated the results of Xu *et al*. [27]. At the level of differentiation, the enhanced expression of PARK7 in MD/PD/WD metastatic OSCC compared to keratinized WD OSCC supports tumor progression and is correlated with a poor clinical outcome [26].

The fibrous nature of keratinized OSCC may be subjected to the enhanced formation of the cornified envelope and type I hemidesmosome assembly (KRT14, KRT16, KRT17, LAMC2) and the assembly of collagen fibrils and other multimeric structures (LAMC2). LAMC2, an isoform of the laminin family, and the KRT14, KRT16, and KRT17 hemi-desmosomal proteins play crucial roles in tumor cell stability and filament formation anchorage, migration, and proliferation. Increased expression of LAMC2 has been evaluated in a variety of cancers, including esophageal, colorectal, gastric, oral squamous cell, and prostate cancers, and it has also been associated with invasiveness in cervical lesions [28] and KRT17 in oral cancer [29]. Increased expression of KRT14, KRT17, and LAMC2 in WD keratinized OSCC suggests reduced invasiveness and migration, as observed by Yamamoto *et al.* [30] (Supplementary Table 5A). Decreased expression of KRT14, KRT17, and LAMC2 and increased expression of KRT16 in MD/PD/WD metastatic OSCC suggest the invasive and migration potential of the cancer cells, as supported by Hao *et al*. 2001 [31] and Huang *et al*. 2019 [32].

Immunohistochemically, CD44 has been suggested to be a prognostic marker in OSCC and is correlated with metastasis [33]. Decreased expression in MD/PD/WD metastatic OSCC compared to WD OSCC further supports the above hypothesis.

SH3 domain-binding glutamic acid-rich protein-like 3 (SH3BGRL3), a thioredoxin superfamily member, shows a significant association with increased levels of EGFR in bladder cancer. SH3BGRL3 promotes EMT, cell migration, and proliferation of urothelial carcinoma *in vitro* [34]. However, the differential expression levels between MD/PD/WD metastatic OSCC and WD OSCC are not in agreement. However, as of limited samples availability these markers need to be validated in more number of samples, is highly suggestive to obtain similarly more defined results.

The identified novel intrachromosomal Ch12 fusion between KRT6B–KRT6A and interchromosomal fusions between CKB-Ch14 and CKM-Ch19, ACTB-Ch7–ACTA2-Ch10, ACTB-Ch7–ACTC1-Ch15, ACTB-Ch7–ACTG2-Ch2, and IGKV1-27–IGKV3-15 identified through long-read sequencing after validation demonstrated IGKV/IGKJ rearrangements in keratinized OSCC. Except for IGKV1-27–IGKV3-15, all other rearrangements, IGKV4-1–IGKJ1, IGKV4-1–IGKJ2, IGKV4-1–IGKJ3, and IGKV4-1–IGKJ4, were highly represented in keratinized OSCCs compared to their normal counterparts. The IGKV/IGKJ rearrangements have been reported under various pathological conditions [35, 36]; however, myeloid-derived IGKV/IGKJ sequences are involved in the migration and chemotaxis of acute myeloid leukemia (AML) cells [37]. Additionally, ACTA2–ACTB, ACTB–ACTC1, and ACTB–ACTG2 were observed in keratinized OSCC and have not been observed in healthy samples (ACTB–ACTG2-RNA-Seq data from samples of the 1000 genomes project). CKM–CKB and KRT6B–KRT6A fusions were observed in metabolic reprogramming and hemidesmosome assembly pathways (Supplementary Table 5B).

Five validated novel isoforms can now be explored for their role in highly keratinized OSCC with improved prognosis. For example, IL37, with five isoforms reported to date, exhibited specifically increased expression of the IL-37β isoform that has been observed in skin equivalent models of epidermal keratinocyte differentiation [38] and many other tissues, including the epidermis [39], stratum corneum [40], and cancerous tissues [41]. Lin *et al*. 2016 [42] reported a wave-curve pattern in the development of OSCC and specified that expression was greater in nonmetastatic with lower migratory potential than in metastatic OSCC. Additionally, IL-37 reduces inflammation and suppresses immune responses [43] both in epidermal keratinization and cancer; hence, exploring the role of the novel IL-37 isoform (similar to IL-37β, sharing exons 1, 2, 4, 5, and 6) with less stability than reference transcript identified in our study in highly keratinized OSCC will be of interest.

Similarly, the novel NAA10 isoform with a fusion of exons 1 and 2 with less stability than reference transcript in our study which may alter the acetyltransferase domain affecting the N-terminal acetylation of proteins [42]. The fusion present in SPAG7 with higher stability than a reference transcript may also be important to understand because it is a cancer-testis (CT) antigen responsible for anticancer immune response activation. Rab24 exon 1, 567 bp in length, was expressed only in the control compared to the keratinized OSCC samples (missing exon, less stability than reference transcript may play an important role in controlling the premalignant phenotype of keratinized OSCC. The insertion of one exonic fragment between the 6th and 7th exons of constitutive UCHL3 may lower its stability than the reference transcript, subsequently altering its deubiquitination activity.

Our identified gene expression signature and novel isoform could play an important role in the prognosis of keratinized OSCC independently of or in conjunction with previously characterized genetic alterations. Additionally, the significant CN gain of EGFR with a frequency of 81.81% and further over-expression in 100% histopathologically characterized FFPE-WD keratinized OSCC suggests the use of targeted therapy of TKI in well-differentiated OSCC.

## MATERIALS AND METHODS

### Ethical approval and informed consent

This study was approved by the Institutional Ethics Committees of King George’s Medical University, Lucknow, India. All patients were recruited in this study after taking written informed consent. All participants had keratinized OSCC, with 63.33% of OSCC originating from the buccal mucosa, 30% originating from the tongue and 6.6% originating from the alveolus. None of the patients were reported to have verrucous and basaloid squamous cell carcinoma.

### Isolation of DNA and RNA

Genomic DNA was extracted from both control and tumor tissue samples using a QIAamp Tissue DNA isolation kit (Qiagen) following the manufacturer’s protocols. Additionally, the concentrations of dsDNA samples were also measured through Qubit DNA BR reagent and were processed for molecular inversion-based probe array (MIP-based array) hybridization. Total RNA was extracted from 50 to 100 mg of both control and tumor specimens using the TRIzol reagent (Invitrogen, Carlsbad, CA, USA) and FFPE RNA was isolated using the RNeasy FFPE kit (Qiagen, Venlo, Netherlands) from the preserved FFPE blocks per manufacturer manual.

### HTA2.0 Hybridization

Total 16 tumor i.e., OT-3, OT-7, OT-9, OT-33, OT-34, OT-35, OT-42, OT-44, OT-45 including OT-10, OT-11, OT-18, OT-19, OT-23, and OT-24 alone and pool and 4 oral control (OC-2, OC-6, OC-22, OC-40) alone & pool samples were processed through GeneChip^®^ Human Transcriptome Array 2.0 (HTA 2.0, Affymetrix, Santa Clara, CA, USA) per manufacturer’s instructions. Quality examined HTA 2.0 chip’s raw data (CEL files) were converted into. rma-gene-ful. chp and .rma-alt-splice-dabg. chp files through Affymetrix Expression Console™ Software (version 1.3). After running ANOVA, a multi-testing correction was performed using the Benjamini–Hochberg step-up false discovery rate (FDR)-controlling procedure (*p* < 0.05) for all expressed genes and expressed probe selection regions (PSRs) and junctions (i.e., expressed in at least one condition). Finally, data were analyzed both at the gene and exonic level. Highly significant (*p* < 0.001) gene-level differentially expressed coding and non-coding transcript clusters were analyzed using a one-way ANOVA algorithm and default filtering criteria (Abs FC ≥ 2 and ANOVA *p*-value ≤ 0.001). At exonic-level, differential expression was analyzed using specific splicing index filter criteria [Exon Splicing Index (linear and exon expressed in at least one condition) < −10 or > 10; 2. (ANOVA Exon *p*-value < 0.0013.) Gene fold change (due to linear and exon expressed in both conditions) < −4 or Gene fold change (linear) > 4]. All microarray data were submitted to NCBI GEO. The GEO submission number is GSE138682. z [44].

### IsoSeq sequencing

IsoSeq sequencing was performed on histopathologically and molecularly classified pooled-OT samples and pooled-OC samples. Total RNA with an RNA integrity number (RIN) > 8.0 was considered for library preparation. The library was constructed according to the Clontech SMARTer-PCR full-length cDNA synthesis preparation guide. Libraries 500 bp-1.5 KB, 1.5–3.0 KB, and 3.0–6.0 KB in size were selected through Blue Pippin, purified, end-repaired, and finally blunt-end ligated to SMART bell adapters. The libraries were quantified using Qubit (Invitrogen) and validated for quality and size by running a LabChip^GX^ (Caliper Life Sciences). Subsequently, sequencing was performed in an 8-well SMRT Cell v3 in PacBioRSII, and data were generated with a 6-hr collection protocol and 10–12 SMRT cells with a total count of 50 cells.

### Bioinformatics analysis for the identification of novel transcript isoforms and long noncoding RNAs

The generated raw sequences were further processed through the IsoSeq pipeline using the following parameters at the filtering step: minimum full passes = 2 and minimum predicted accuracy = 85. At the classification step, reads with less than 100 bases were removed and classified as FL or non-FL reads. The reads classified as FL were further polished with the quiver algorithm to improve error-corrected consensus accuracy and clustered using isoform-level clustering (ICE) into high-quality and low-quality FL consensus reads, yielding 20,600 and 10,637 FL consensus isoforms for OC and OT conditions, respectively, with expected accuracy greater than 0.99 (NCBI accession: SRA temporary ID: SUB5166507; Bio project accession no: PRJNA521842).

All high-quality FL transcripts were aligned to the hg38 genome using GMAP to predict consensus isoforms as FASTA and FASTQ files. The aligned sequences (FASTAQ files) were parsed with TAMA (https://github.com/GenomeRIK/tama/) to remove highly similar sequences using default parameters, (https://github.com/PacificBiosciences/cDNA_primer/wiki; minimum alignment accuracy of 0.95 and minimum coverage of 0.85) [45]. Specifically, the alignments were further collapsed by redundant transcript models in OC and OT separately using the “Transcription Start Site Collapse” (TSSC) model in the TAMA pipeline (https://github.com/GenomeRIK/tama/). Transcripts sharing the same exons, except those with an extended 5′ end (or transcription start site) and 3′ ends aligned with redundant transcripts, were collapsed into transcript isoforms (OC and OT bed files). The generated high-quality isoforms were screened for the identification of novel isoforms with different splicing events and long noncoding RNA using Integrated Genome Viewer (IGV) [46].

For the identification of long noncoding RNA, the GMAP-aligned sequences were searched for alignment with the hg38 genome, unavailable and unannotated sequences were searched against the nucleotide database, and the sequences without homology were further screened against noncoding RNA databases. The aligned sequences against the noncoding RNA database were classified as long noncoding RNAs.

### Differential gene expression analysis of FL high-quality and circular consensus sequencing (CCS) reads between the OC and OT samples

The 20,600 and 10,637 high-quality FL isoform reads in OC and OT, respectively, and CCS reads, were aligned to the hg38 genome reference using the STAR tool. The alignments were converted to SAM alignments using SAMtools, and the GFOLD tool was used for differential analysis between OC and OT FL isoforms using default parameters. The GFOLD (Generalized Fold Change) algorithm method produces biologically meaningful rankings of differentially expressed genes (*p* < 0.05) which considers the posterior distribution of log fold change such that each gene is assigned a reliable fold change. Hence, on applying GFOLD (more than 2-fold), it ranked differentially expressed genes for well-differentiated Keratinized [47].

### Identification of fusion genes

The 20,600 and 10,637 FL isoform reads of OC and OT, respectively, were aligned against a reference genome (human genome hg38) with the STAR tool and were analyzed with STAR long aligner with the parameters –chim Segment Min 12, –chim Junction Overhang Min 12, and –chim Segment Read Gap Max parameter 3. The output file “Chimeric.out.junction” was then used by the STAR-Fusion pipeline to detect the fusion genes with the following parameters: STAR-Fusion–genome_lib_dir, -J Chimeric. out. junction and –output_dir star_fusion_outdir. A minimum of three total fusion-supporting RNA-Seq fragments per 20 M total reads (or normalized to 0.15 fusion fragments per million total RNA-Seq fragments) and highly accurate fusion transcripts were identified. The settings used were appropriate to remove low-scoring fusion events. The genome annotation file in .gff format was used to annotate the fusion genes in the STAR-Fusion pipeline, and the obtained fusion genes were manually inspected in IGV to confirm their occurrence. Further, each fusion-specific probe set was designed for validation [48].

### Homology analysis and functional assignment

The putative function of the assembled contigs was deduced by using them as queries against the SwissProt and nr protein databases in the BLASTX program using a cutoff E-value set at 1e-5, and only the top gene ID and name were initially assigned to each contig. GO annotation analysis was further performed with Blast2GO (https://www.blast2go.org/) version 2.5.0 [49] for the assignment of GO terms. After gene ID mapping, GO term assignment, annotation augmentation, and a generic GO-slim process, the final annotation file was produced, and the results were categorized into biological process, molecular function, and cellular component at level 2.

Pathway analyses of unique sequences were carried out based on the KEGG database using the online KEGG Automatic Annotation Server (KAAS) (http://www.genome.jp/tools/kaas/) and the BBH method. EC numbers were obtained and putatively mapped for protein sequences to a specific biochemical pathway [50]. A threshold of *p* < 0.05 was used to indicate significant function and pathway categories.

### Validation for oral fusion and transcripts through NanoString nCounter platform

Forty-one samples including 27 OT samples, i.e., pooled-OT (OT-10, OT-11, OT-18, OT-19, OT-23, and OT-24), OT-1, OT-3, OT-7, OT-10, OT-11, OT-12, OT-14, OT-15, OT-16, OT-17, OT-18, OT-19, OT-23, OT-24, OT-25, OT-26, OT-29(M), OT-30, OT-31, OT-33, OT-35, OT-38, OT-39, OT-42, OT-44, and OT-45 tumor samples; 14 histopathologically characterized WD FFPE samples, FFPE2, FFPE3, FFPE4, FFPE5, FFPE6, FFPE8, FFPE9, FFPE11, FFPE12, FFPE13, FFPE14, FFPE15, FFPE16, and FFPE17; and 5 control samples, i.e., pooled-OC (OC-2, OC-6, OC-22), OC21, OC27, OC28, and OC34, were processed for NanoString nCounter gene expression analysis. Probes for nCounter were designed for 41 code sets for oral transcripts from mRNA of *Homo sapiens* and 12 highly accurate STAR-Fusion-derived fusion transcripts from both the 20,600 and 10,637 FL isoform reads in OC and OT, respectively. The code set also included two long noncoding RNAs (OC_KGMU_lnRNA_1371 and OC_KGMU_lnRNA_1297) identified *via* GFOLD differential transcripts of FL transcript isoforms. Experiments have been performed according to the instruction manual (NanoString Technologies). nSolver™ Analysis Software 3.0 (NanoString Technologies) was used to perform background subtraction, spike-in-control normalization, and reference gene normalization. A heat map and scatterplot were generated in nSolver using normalized gene expression values for the 46 genes that were significantly different (*p* < 0.05; FDR < 0.05%) and 5 housekeeping genes (ACTB, GAPDH, RPL19, TBP, TUBB). For fusion transcripts, for each sample, transcript counts were normalized to the 6 positive and 8 negative controls in the nCounter panel and the 5 housekeeping genes (ACTB, GAPDH, RPL19, TBP, TUBB) and compared to the control samples.

### The expression-based analysis of identified novel isoforms in the ISOexpresso database

Multiple alignments through Clustalw2 were performed to validate the insertion, deletion or fusion of exonic nucleotide sequences in the coding region of 33 FL transcripts of pooled-OC and pooled-OT samples and nmIDs of the human genome (hg38). Additionally, the expression analyses of various novel isoforms were also correlated using the ISOexpresso database, a database that facilitates expression-based isoform-level analysis in cancer cells. In this database, RNA sequencing data and patient clinical data are available for 520 tumors and 44 normal controls of head and neck squamous cell carcinoma from The Cancer Genome Atlas (TCGA) data portal using gene and isoform information based on hg19/GRCh37, including IDs of genes and isoforms, genomic location, and known canonical/principal isoforms from the UCSC Annotation database, Universal Protein Resource (UniProt), NCBI Reference Sequence Database (RefSeq), Ensembl, Consensus CDS (CCDS), Annotating principal splice isoforms (APPRIS), and HUGO Gene Nomenclature Committee (HGNC).

### Validation of the expression of inserted/deleted/fused exons in identified novel transcripts through quantitative real-time PCR

The relative expression levels of inserted, deleted and fused exons in the identified novel transcripts were measured by real-time PCR on a 7500 fast Dx Real-Time PCR instrument (Applied Bioscience Inc.) using β-actin as a reference gene, and analysis was performed with REST 2009 software; the whisker-box plots were extracted with 2000 time iterations (http://www.REST.de.com).

### Validation of the inserted/deleted/fused exons in the identified novel transcripts *via* PCR

To validate the inserted/deleted/fused exons in the identified novel transcripts, exon-specific primer PCR was conducted for each inserted/deleted/fused exon, and band intensities for pooled-OC and pooled-OT samples were observed in agarose gel electrophoresis with β-actin as the control. The images were taken by image Quant LAS 4000 and band densities were analyzed using Image ‘J’ software.

### Bioinformatics tools for the prediction of the structural and functional role of proteins in identified novel transcripts

Protein structures of UCHL3, RAB24, IL-37, NAA10, and SPAG7 wild type, oral control (OC), and oral tumor (OT) sample were predicted by bioinformatics tools. The mRNA CDS and AA sequence of wild-type were gained in NCBI (http://www.ncbi.nlm.nih.gov/blast). Also, the transcript of UCHL3, RAB24, IL-37, NAA10, and SPAG7 of OC and OT was translated into a new AA sequence using expasy translate (https://web.expasy.org/translate/). Then, the AA sequence of the OT and OC isoform were aligned with the wild-type by NCBI protein blast (http://blast.ncbi.nlm.nih.gov/). Physicochemical properties of the wild-type and new isoform were predicted by online tools ProtParam and ProtScale (http://www.expasy.ch/tools/protscale.html), respectively. Secondary structures of the wild-type and new isoform AA sequences were predicted by CFSSP (http://www.biogem.org/tool/chou-fasman/).

### MIP-based array hybridization

Total 18 tumor i.e., pooled-OT, OT-3, OT-7, OT-9, OT-33, OT-38, OT-39, OT-40, OT-42, OT-43, OT-44, and OT-45, and 5 oral control i.e., OC-2, OC-6, OC-22, kit-based positive and negative controls, were processed for CNVs. DNA (12 ng/μL per sample) was processed on a MIP-based OncoScan array for CNV profiling. According to the recommended protocol, the chips were processed for hybridization, staining, and washing procedures and were finally scanned through GeneChip Scanner-7G (Affymetrix, Santa Clara, CA, USA) for identification of the copy number and somatic mutation variations as reported previously [44, 51]. The OSCHP files were generated using OncoScan Console Software (Biodiscovery, Inc., CA, USA) and were analyzed through tumor Scan (TuScan) and BioDiscovery’s SNP-FASST2 algorithm using Nexus Express for OncoScan software version 7.5 [51].

### Validation of the EGFR exon 19 amplicon

Using the EGFR exon 19-specific primer, quantitative real-time PCR as described above was conducted in five OC samples and twelve histopathologically characterized FFPE OT samples with β-actin as the reference gene. Statistically, 2^–ΔΔCt^ was calculated that indicates amplicon doubled during each cycle, then there would be the same expression ratio derived from each group. The data have been expressed as a fold change in relative gene expression. Statistical analysis and graphs were drowned in GraphPad Prism software. The statistical significance of fold change, the *p*-value was calculated by Mann–Whitney *U*-test. A statistically significant difference was defined as ^*^ *p* < 0.05, ^**^ *p* < 0.01, and ^***^ *p* < 0.001.

## CONCLUSIONS

Increased cell proliferative markers, dysregulated metabolic reprogramming, increased oxidative stress, increased the involvement of the immune system, and enhanced immune rearrangements suggest the cancerous nature of keratinized OSCC. However, increased proteasomal activity and type I hemidesmosome assembly suggests improved prognosis and tumor cell stability in keratinized OSCC. Hence, EGFR amplification/overexpression, 18 differentially expressed FL transcripts, and enhanced immune rearrangements of IGKV4-1–IGKJ1, IGKV4-1–IGKJ2, IGKV4-1–IGKJ3, and IGKV4-1–IGKJ4 can be used as signature markers for characterizing keratinized OSCC with MD/PD/WD with lymphadenopathy and may play an important role in controlling the premalignant phenotype of keratinized OSCC and progression of the disease. Additionally, novel isoforms of IL37, NAA10, UCHL3, SPAG7, and RAB24 were identified while *in silico* functionally validated SPAG7 represented the premalignant phenotype of keratinized (K4) OSCC.

## SUPPLEMENTARY MATERIALS






