# Supplementary material for: Differentially expressed full-length, fusion and novel isoforms transcripts-based signature of well-differentiated keratinized oral squamous cell carcinoma
Source: Oncotarget. 2020 Aug 25;11(34):3227–43. doi: 10.18632/oncotarget.27693 (PMC7456611; doi:10.18632/oncotarget.27693)
Supplement: Supplementary file 1 [file oncotarget-11-3227-s001.pdf]

Differentially expressed full-length, fusion and novel isoforms transcripts-based signature of well-differentiated keratinized oral squamous cell carcinoma

SUPPLEMENTARY MATERIALS

A

(i)

```
CLUSTAL O(1.2.4) multiple sequence alignment

IL37_Ref      MSFVGENSGVKMGSEDWEKDEPQCCLEDPAGSPLEPGPSLPTMNFVHTSPKVKNLNPKKF   60
IL37_OT       MSFVGENSGVKMGSEDWEKDEPQCCLEDPAVSPLEPGPSLPAMNFVHTSPKVKNLNPKKF   60
              ****************************** **********:******************

IL37_Ref      SIHDQDHKVLVLDSGNLIAVPDKNYIRPEIFFALASSLSSASAEKGSPILLGVSKGEFCL   120
IL37_OT       SIHDQDHKVLVLDSGNLIAVPDKNYIRPEIFFALASSLSSASAEKGSPILLGVSKGEFCL   120
              ************************************************************

IL37_Ref      YCDKDKGQSHPSLQLKKEKLMKLAAQKESARRPFIFYRAQVGSWNMLESAAHPGWFICTS   180
IL37_OT       YCDKDKGQSHPSLQLKKEKLMKLAAQKESARRPFIFYRAQVGSWNMLESAAHPGWFICTS   180
              ************************************************************

IL37_Ref      CNCNEPVGVTDKFENRKHIEFSFQPVCKAEMSPSEVSD       218
IL37_OT       CNCNEPVGVTDKFENRKHIEFSFQPVCKAEMSPSEVSD       218
              **************************************
```

(ii)

Ref: IL-37

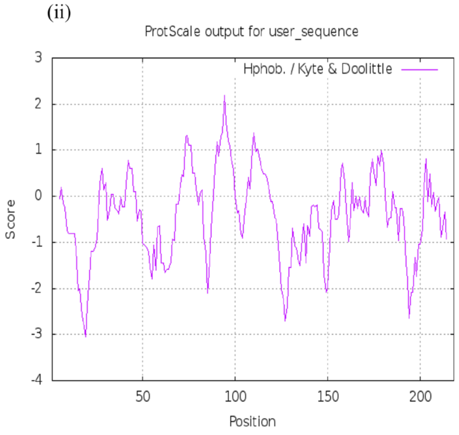

(iii)

OT: IL-37

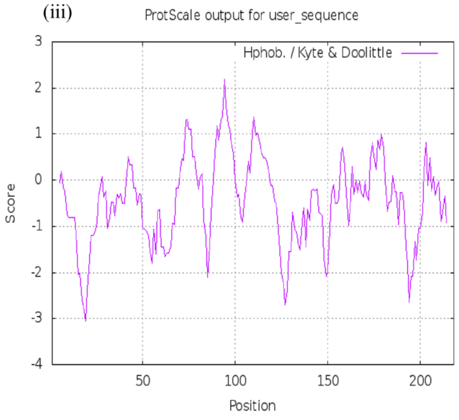

B

(i)

```
CLUSTAL O(1.2.4) multiple sequence alignment

RAB24_Ref      MSGQRVDVKVVMLGKEYVGKTSLVERYVHDRFLVGPYQNTIGAAFVAKVMSVGDRTVTLG  60
RAB24_OC       MSGQRVDVKVVMLGKEYVGKTSLVERYVHDRFLVGPYQNTIGAAFVAKVMSVGDRTVTLG  60
RAB24_OT       -------------------------------------------------MSVGDRTVTLG  11
                                                                 ***********

RAB24_Ref      IWDTAGSERYEAMSRIYYRGAKAAIVCYDLTDSSSFERAKFWVKELRSLEEGCQIYLCGT  120
RAB24_OC       IWDTAGSERYEAMSRIYYRGAKAAIVCYDLTDSSSFERAKFWVKELRSLEEGCQIYLCGT  120
RAB24_OT       IWDTAGSERYEAMSRIYYRGAKAAIVCYDLTDSSSFERAKFWVKELRSLEEGCQIYLCGT  71
               ************************************************************

RAB24_Ref      KSDLLEEDRRRRRVDFHDVQDYADNIKAQLFETSSKTGQSVDELFQKVAEDYVSVAAFQV  180
RAB24_OC       KSDLLEEDRRRRRVDFHDVQDYADNIKAQLFETSSKTGQSVDELFQKVAEDYVSVAAFQV  180
RAB24_OT       KSDLLEEDRRRRRVDFHDVQDYADNIKAQLFETSSKTGQSVDELFQKVAEDYVSVAAFQV  131
               ************************************************************

RAB24_Ref      MTEDKGVDLGQKPNPYFYSCCHH     203
RAB24_OC       MTEDKGVDLGQKPNPYFYSCCHH     203
RAB24_OT       MTEDKGVDLGQKPNPYFYSCCHH     154
               ***********************
```

Ref: RAB24

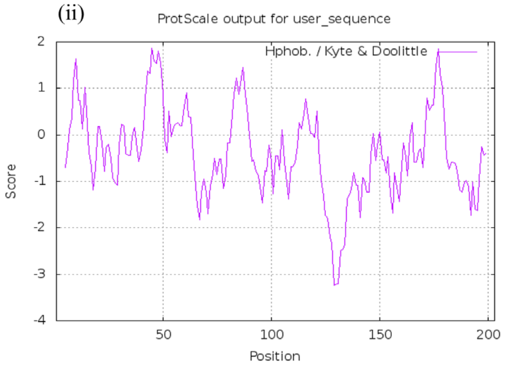

OC: RAB24

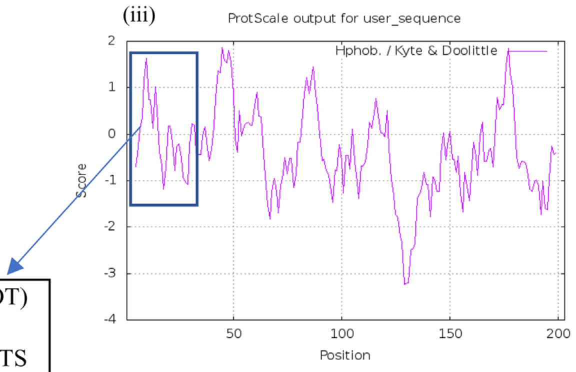

(Deleted Amino Acid sequence in OT)

MSGQRVDVKVVMLGKEYVGKTS
LVERYVHDRFLVGPYQNTIGAAF

OT: RAB24

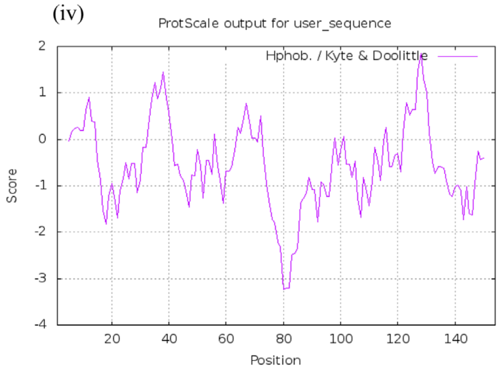

C

(i)

```
CLUSTAL O(1.2.4) multiple sequence alignment
NAA10_Ref      ---------------MNIRN----------------------------------------     5
NAA10_OC       ---------------MNIR-----------------------------------------     4
NAA10_OT       MSGLRWVGSGDLRGAHSCSCAPGVVQSQIVTVPAQPRGRGPSRPTGSRLLTRGHRRLRLS     60

NAA10_Ref      -------ARPEDLMNMQHCNLLCLPENYQMKYYFYHGLSWPQLSYIAEDENGKIVGE---     55
NAA10_OC       ------NARPEDLMNMQHCNLLCLPENYQMKYYFYHGLSWPQLSYIAEDENGKIVGYVLA     58
NAA10_OT       AFHCPPSLQPEDLMNMQHCNLLCLPENYQMKYYFYHGLSWPQLSYIAEDENGKIVGYVLA     120
                        ***********************************************

NAA10_Ref      ---EDPDDVPHGHITSLAVKRSHRRLGLAQKLMDQASRAMIENFNAKYVSLHVRKSNRAA     112
NAA10_OT       KMEEDPDDVPHGHITSLAVKRSHRRLGLAQKLMDQASRAMIENFNAKYVSLHVRKSNRAA     180
NAA10_OC       KMEEDPDDVPHGHITSLAVKRSHRRLGLAQKLMDQASRAMIENFNAKYVSLHVRKSNRAA     118
                  *********************************************************

NAA10_Ref      LHLYSNTLNFQISEVEPKYYADGEDAYAMKRDLTQMADELRRHLELKEKGRHVVLGAIEN     172
NAA10_OT       LHLYSNTLNFQISEVEPKYYADGEDAYAMKRDLTQMADELRRHLELKEKGRHVVLGAIEN     240
NAA10_OC       LHLYSNTLNFQISEVEPKYYADGEDAYAMKRDLTQMADELRRHLELKEKGRHVVLGAIEN     178
               ************************************************************

NAA10_Ref      KVESKGNSPPSSGEACREEKGLAAEDSGGDSKDLSEVSETTESTDVKDSSEASDSAS  229
NAA10_OT       KVESKGNSPPSSGEACREEKGLAAEDSGGDSKDLSEVSETTESTDVKDSSEASDSAS  297
NAA10_OC       KVESKGNSPPSSGEACREEKGLAAEDSGGDSKDLSEVSETTESTDVKDSSEASDSAS  235
               *********************************************************
```

Ref: NAA10

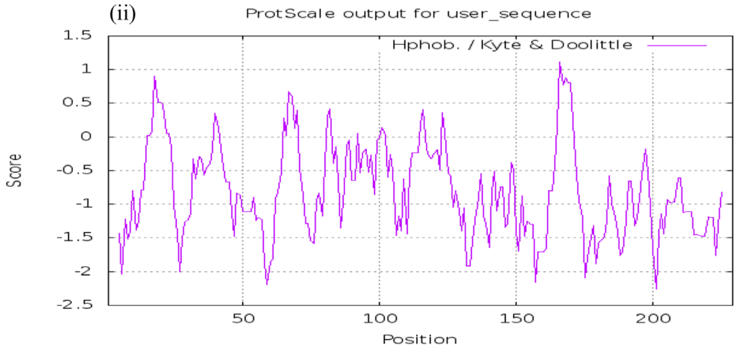

OC: NAA10

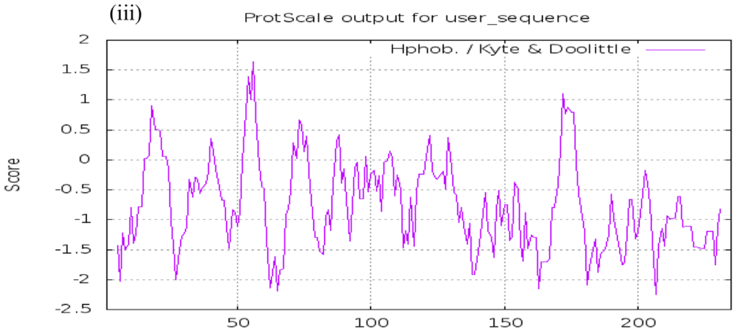

OT: NAA10

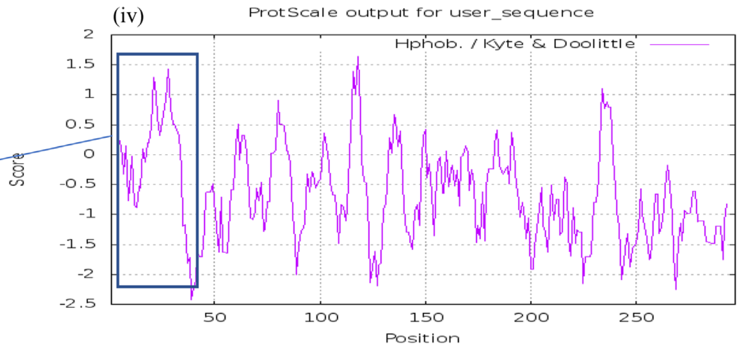

(Inserted Amino Acid sequence in OT)

MSGLRWVGSGDLRGAHSCSCAPGVVQSQ
IVTVPAQPRGRGPSRPTGSRLLTRGHRR

D

(i)

```
CLUSTAL O(1.2.4) multiple sequence alignment

SPAG7_OT       MADLLGSILSSMEKPPSLGDQETRRKAREQAARLKKLQEQEKQQKVEFRKRMEKEVSDFI     60
SPAG7_OC       MADLLGSILSSMEKPPSLGDQETRRKAREQAARLKKLQEQEKQQKVEFRKRMEKEVSDFI     60
SPAG7_Ref      MADLLGSILSSMEKPPSLGDQETRRKAREQAARLKKLQEQEKQQKVEFRKRMEKEVSDFI     60
               ************************************************************

SPAG7_OT       QDSGQIKKKFQPMNKIERSILHDVVEVAGLTSFSFGEDDDCRYVMIFKKEFAPSDEELDS     120
SPAG7_OC       QDSGQIKKKFQPMNKIERSILHDVVEVAGLTSFSFGEDDDCRYVMIFKKEFAPSDEELDS     120
SPAG7_Ref      QDSGQIKKKFQPMNKIERSILHDVVEVAGLTSFSFGEDDDCRYVMIFKKEFAPSDEELDS     120
               ************************************************************

SPAG7_OT       YRRGEEWDPQKAEEKRKLKELAQRQEEEAAQQGPVVVSPASDYKDKYSHLIGKGAAKDAA     180
SPAG7_OC       YRRGEEWDPQKAEEKRKLKELAQRQEEEAAQQGPVVVSPASDYKDKYSHLIGKGAAKDAA     180
SPAG7_Ref      YRRGEEWDPQKAEEKRKLKELAQRQEEEAAQQGPVVVSPASDYKDKYSHLIGKGAAKDAA     180
               ************************************************************

SPAG7_OT       HMLQANKTYGCGEATVRLGVAGRGAWMWQEGSGGMRYGFLGPPTLLSAPSARGQ     234
SPAG7_OC       HMLQANKTYGCVPVANKRDTRSIEEAMNEIRAKKRLRQSGEELPPTS-------     227
SPAG7_Ref      HMLQANKTYGCVPVANKRDTRSIEEAMNEIRAKKRLRQSGEELPPTS-------     227
               ***********               *                   *
```

(ii)

Ref: SPAG7

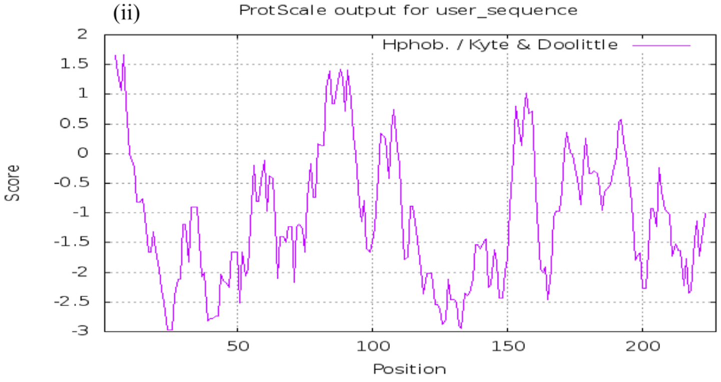

(iii)

OC: SPAG7

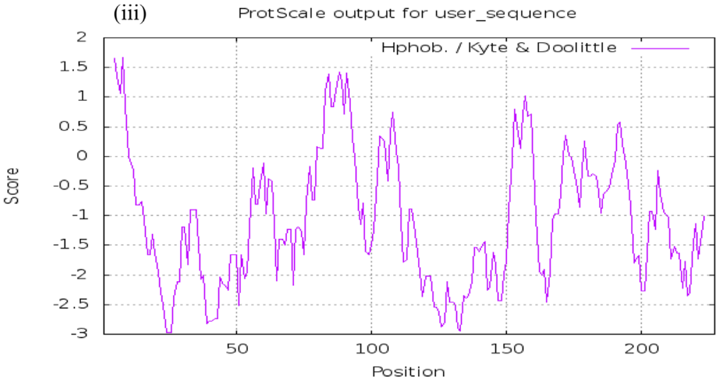

(iv)

OT: SPAG7

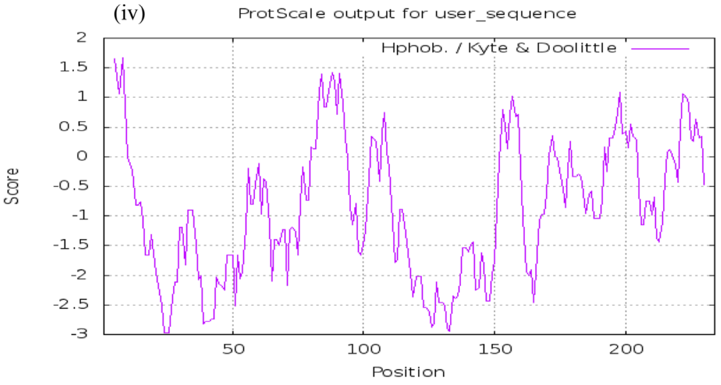

E

(i)

```
CLUSTAL O(1.2.4) multiple sequence alignment

UCHL3_Ref      MEGQRWLPLEANPEVTNQFLKQLGLHPNWQFVDVYGMDPELLSMVPRPVCAVLLLFPITE  60
UCHL3_OC       MEGQRWLPLEANPEVTNQFLKQLGLHPNWQFVDVYGMDPELLSMVPRPVCAVLLLFPITE  60
UCHL3_OT       ------------------------------------MDPELLSMVPRPVCAVLLLFPITE  24
                                                   ************************

UCHL3_Ref      KYEVFRTEEEEKIKSQGQDVTSSVYFMKQTISNACGTIGLIHAIANNKDKMHFESGSTLK  120
UCHL3_OC       KYEVFRTEEEEKIKSQGQDVTSSVYFMKQTISNACGTIGLIHAIANNKDKMHFESGSTLK  120
UCHL3_OT       KYEVFRTEEEEKIKSQGQDVTSSVYFMKQTISNACGTIGLIHAIANNKDKMHFESGSTLK  84
               ************************************************************

UCHL3_Ref      KFLEESVSMSPEERARYLENYDAIRVTHETSAHEGQTE----------------------  158
UCHL3_OC       KFLEESVSMSPEERARYLENYDAIRVTHETSAHEGQTE----------------------  158
UCHL3_OT       KFLEESVSMSPEERARYLENYDAIRVTHETSAHEGQTESSSPSSSQPHSSHCRTKASSLC  144
               **************************************

UCHL3_Ref      ------------------------------------------APSIDEKVDLHFIALVHV  176
UCHL3_OC       ------------------------------------------APSIDEKVDLHFIALVHV  176
UCHL3_OT       HHASLPWVKRNHVGPAKATSPSLRLRRWRPFLRLPSLGLHSVAPSIDEKVDLHFIALVHV  204
                                                         ******************

UCHL3_Ref      DGHLYELDGRKPFFPINHGETSDETLLEDAIEVCKKFMERDPDELRFNAIALSAA       230
UCHL3_OC       DGHLYELDGRKPFFPINHGETSDETLLEDAIEVCKKFMERDPDELRFNAIALSAA       230
UCHL3_OT       DGHLYELDGRKPFFPINHGETSDETLLEDAIEVCKKIMERDPDELRFNAIALSAA       258
               ************************************:*****************
```

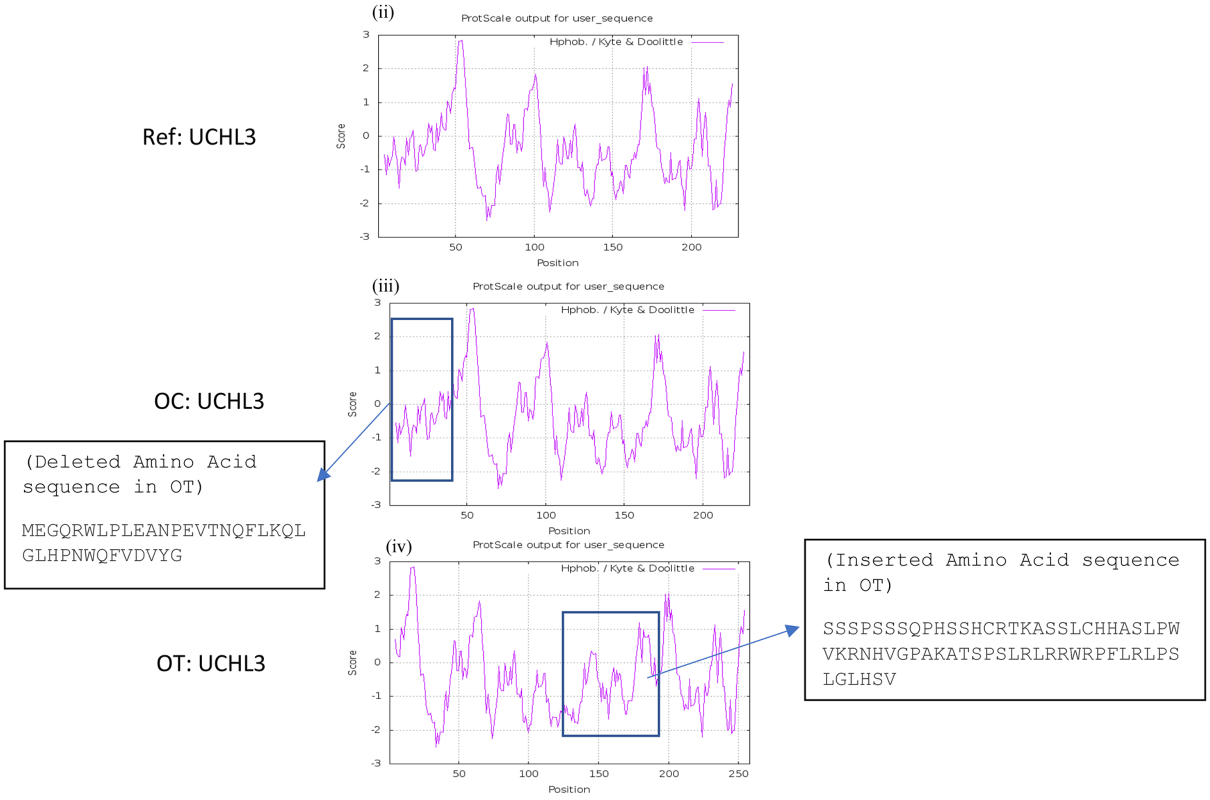

Supplementary Figure 1: (**A**) Analysis of the amino acid sequences of the IL37 wild-type and OT isoform. (i)The amino acid sequence of the OT was aligned with the IL37 wild-type by NCBI protein blast. Hydropathicity of the (ii) wild- type and (iii) the OT isoform was predicted by ProtParam and ProtScale, respectively. (**B**) Analysis of the amino acid sequences of RAB24 wild-type, OC and OT isoform. (i) The amino acid sequence of OC and OT isoform was aligned with the RAB24 wild-type by NCBI protein blast, and deletion of 45 amino acid sequence was found. Hydropathicity of (ii) wild-type, (iii) OC and (iv) OT isoform, (box indicates the deleted 45 AA) of RAB24 was predicted by ProtParam and ProtScale, respectively. (**C**) Analysis of the amino acid sequences of NAA10 wild-type, OC and OT isoform. (i) The amino acid sequence of the OC and OT isoform was aligned with the NAA10 wild-type by NCBI protein blast, and insertion of 69 amino acid sequence was found. Hydropathicity of (ii) wild-type, (iii) OC and (iv) OT isoform, box indicates the inserted 69 AA) of NAA10 was predicted by ProtParam and ProtScale, respectively. (**D**) Analysis of the amino acid sequences of SPAG7 wild-type, OC and OT isoform. (i) The amino acid sequence of the OC and OT isoform was aligned with the SPAG7 wild-type by NCBI protein blast.

Hydropathicity of (ii) wild-type, (iii) OC and (iv) OT isoform of SPAG7 was predicted by ProtParam and ProtScale, respectively. (**E**) Analysis of the amino acid sequences of UCHL3 wild-type, OC and OT isoform. (i) The amino acid sequence of the OC and OT isoform was aligned with the UCHL3 wild-type by NCBI protein blast, and insertion of 36 amino acid and deletion of 64 AA sequence was found (A). Hydropathicity of (ii) wild-type, (iii) OC and (iv) OT isoform, (box indicates the inserted and deleted AA) of UCHL3 was predicted by ProtParam and ProtScale, respectively.

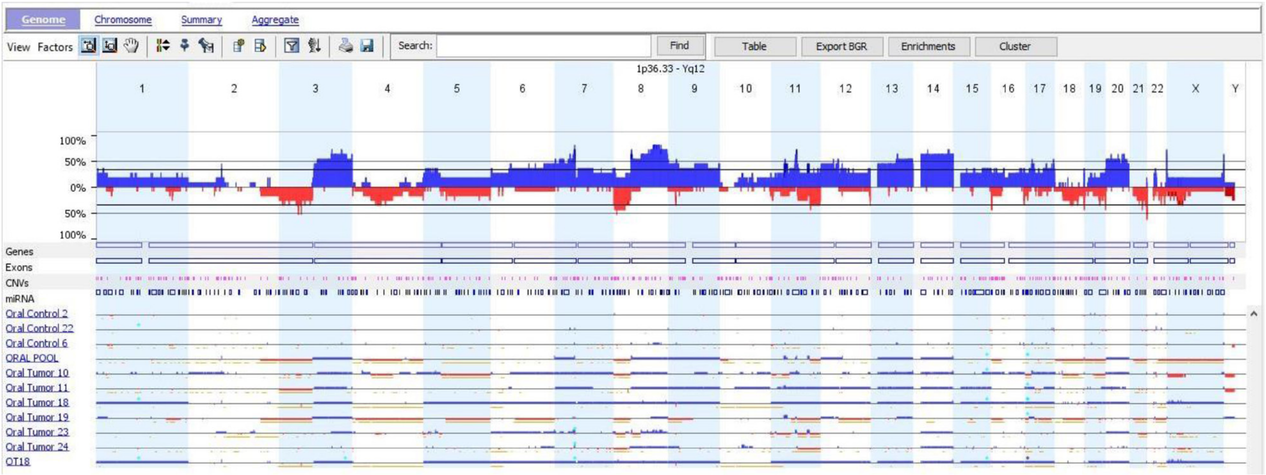

Supplementary Figure 2: Molecular karyogram of OT-10, OT-11, OT-18, OT-19, OT-23 and OT-24 tumor samples and OC-2, OC-6, and OC-22 control samples processed via OncoScan array and analyzed with tumor Scan (TuScan) and BioDiscovery's SNP-FASST2 algorithm using Nexus Express for OncoScan software version 7.5 (Biodiscovery, Inc., CA USA).

Supplementary Table 1: (**A**) Details of keratinized OSCC collected from different anatomical sites (buccal mucosa; tongue and alveolous) of oral cavity. Histopathological classification, Level of differentiation, and involvement of node have also been included. (**B**) Details of oral control samples collected from different anatomical sites. See Supplementary Table 1

Supplementary Table 2: Details of enzymes found in KEGG pathway database from Homo sapiens. See Supplementary Table 2

Supplementary Table 3: Identified differentially expressed (more than 2 fold) isoforms between high quality 20, 600 and 10, 637 FL isoform reads in OC and OT respectively through G-FOLD Tool using default parameters. See Supplementary Table 3

Supplementary Table 4: (**A**) Differential expression of 34 transcripts and five housekeeping genes in 42 tumour samples (15 histo-pathologically characterized formalin fixed paraffin embedded keratinized tumor samples and fresh 27 oral tumor samples as well as four control samples. (**B**) 25 most relevant pathways sorted by *p*-value of validated 34 transcripts in 42 tumor samples (15 histo-pathologically characterized FFPE keratinized. (**C**) Percentage Expression Fusion transcripts in 23 OT and 15 FFPE keratinized OSCC samples compared to 4 oral control samples. See Supplementary Table 4

Supplementary Table 5: Validation of isoforms through Multiple alignment of identified and validated 33 novel full-length transcripts isoforms with RefSeq (NCBI Reference Sequence Database. See Supplementary Table 5

Supplementary Table 6: List of 33 full length novel transcript isoforms showing exonic-insertion, -deletion or -fusion in pooled-OC, pooled-OT samples and NM IDs. See Supplementary Table 6

Supplementary Table 7: (**A**) Highly significant gene-level differentially expressed coding and non-coding transcript clusters between 16 OT and 4 OC-samples, using one-way between-subject ANOVA algorithm and default filtering criteria (Abs FC$\geq$2 and ANOVA *p*-value $\leq$0.001). (**B**) Highly significant (*p*-value $\leq$0.001) differential pathways at gene level between 16 OSCC and 4 Control samples. See Supplementary Table 7

Supplementary Table 8: (**A**) Physicochemical properties of the wild-type, Oral Control and Oral Tumor isoforms of IL37, RAB24, NAA10, SPAG7 and UCHL3. (**B**) Secondary structures of the IL37, RAB24, NAA10, SPAG7 and UCHL3 in wild-type, OC and OT samples. See Supplementary Table 8
