# Supplementary material for: Differentially expressed full-length, fusion and novel isoforms transcripts-based signature of well-differentiated keratinized oral squamous cell carcinoma
Source: Oncotarget. 2020 Aug 25;11(34):3227–43. doi: 10.18632/oncotarget.27693 (PMC7456611; doi:10.18632/oncotarget.27693)
Supplement: Supplementary file 3 [file oncotarget-11-3227-s003.pdf]

**Supplementary Table 5: Validation of isoforms through Multiple alignment of identified and validated 33 novel full-length transcripts isoforms with RefSeq (NCBI Reference Sequence Database.**

## Colours Assigned for Exons by Numbers

| Exon Number | Colour Assigned |
|---|---|
| 1 | |
| 2 | |
| 3 | |
| 4 | |
| 5 | |
| 6 | |
| 7 | |
| 8 | |
| 9 | |
| 10 | |

**SCAMP3**

| **Sequence** | **Header** | **AA Seq** |
|---|---|---|
| **Present** | i1_HQ_samplec9816d\|c20030/f5p0/1447 | >VIRT31885 |
| **Absent** | i1_HQ_samplec9816d\|c29753/f2p0/1106 | >VIRT38385 |
| **NCBI** | NM_005698 | NP_005689.2 |
| | NM_052837 | NP_443069.1 |

>i1_HQ_samplec9816d|c20030/f5p0/1447
isoform=c20030;full_length_coverage=5;non_full_length_coverage=0;isoform_length=1447

agagaagcggggcgaactgaggcgagtgaagtggactctgagggctaccgctaccgCCACTGCTGCGGCAGGGGCGTGGAGGGCAGAGGGCCGCGGA
GGCCGCAGTTGCAAACATGGCTCAGAGCAGAGACGGCGGAAACCCGTTCGCCGAGCCCAGCGAGCTTGACAACCCCTT
TCAGGACCCAGCTGTGATCCAGCACCGACCCAGCCGGCAGTATGCCACGCTTGACGTCTACAACCCTTTTGAGACCCGG
GAGCCACCACCAGCCTATGAGCCTCCAGCCCCTGCCCCATTGCCTCCACCCTCAGCTCCCTCCTTGCAGCCCTCGAGAAA
GCTCAGCCCCACAGAACCTAAGAACTATGGCTCATACAGCACTCAGGCCTCAGCTGCAGCAGCCACAGCTGAGCTGCTG
AAGAAACAGGAGGAGCTCAACCGGAAGGCAGAGGAGTTGGACCGAAGGGAGCGAGAGCTGCAGCATGCTGCCCTGGG
AGGCACAGCTACTCGACAGAACAATTGGCCCCCTCTACCTTCTTTTTGTCCAGTTCAGCCCTGCTTTTTCCAGGACATCT
CCATGGAGATCCCCCAAGAATTTCAGAAGACTGTATCCACCATGTACTACCTCTGGATGTGCAGCACGCTGGCTCTTCTC
CTGAACTTCCTCGCCTGCCTGGCCAGCTTCTGTGTGGAAACCAACAATGGCGCAGGCTTTGGGCTTTCTATCCTCTGGGT
CCTCCTTTTCACTCCCTGCTCCTTTGTCTGCTGGTACCGCCCCATGTATAAGGCTTTCCGGAGTGACAGTTCATTCAATTT
CTTCGTTTTCTTCTTCATTTTCTTCGTCCAGGATGTGCTCTTTGTCCTCCAGGCCATTGGTATCCCAGGTTGGGGATTCAG
TGGCTGGATCTCTGCTCTGGTGGTGCCGAAGGGCAACACAGCAGTATCCGTGCTCATGCTGCTGGTCGCCCTGCTCTTCA
CTGGCATTGCTGTGCTAGGAATTGTCATGCTGAAACGGATCCACTCCTTATACCGCCGCACAGGTGCCAGCTTTCAGAA
GGCCCAGCAAGAATTTGCTGCTGGTGTCTTCTCCAACCCTGCGGTGCGAACCGCAGCTGCCAATGCAGCCGCTGGGGCT
GCTGAAAATGCCTTCCGGGCCCCGTGACCCCTGACTGGGATGCCCTGGCCCTGCTACTTGAGGGAGCTGACTTAGCTCC
CGTCCCTAAGGTCTCTGGGACTTGGAGAGACATCACTAACTGATGGCTCCTCCGTAGTGCTCCCAATCCTATGGCCATG
ACTGCTGAACCTGACAGGCGTGTGGGGAGTTCACTGTGACCTAGTCCCCCCATCAGGCCACACTGCTGCCACCTCTCAC
ACGCCCCAACCCAGCTTCCCTCTGCTGTGCCACGGCTGTTGCTTCGGTTatttaaataaaaagaaagtggaactggaact

>VIRT31885
MAQSRDGGNPFAEPSELDNPFQDPAVIQHRPSRQYATLDVYNPFETREPPPAYEPPAPAP
LPPPSAPSLQPSRKLSPTEPKNYGSYSTQASAAAATAELLKKQEELNRKAEELDRREREL
QHAALGGTATRQNNWPPLPSFCPVQPCFFQDISMEIPQEFQKTVSTMYYLWMCSTLALLL
NFLACLASFCVETNNGAGFGLSILWVLLFTPCSFVCWYRPMYKAFRSDSSFNFFVFFFIF
FVQDVLFVLQAIGIPGWGFSGWISALVVPKGNTAVSVLMLLVALLFTGIAVLGIVMLKRI
HSLYRRTGASFQKAQQEFAAGVFSNPAVRTAAANAAAGAAENAFRAP

>i1_HQ_samplec9816d|c29753/f2p0/1106
isoform=c29753;full_length_coverage=2;non_full_length_coverage=0;isoform_length=1106

acagagaagcggggcgaactgaggcgagtgaagtggactctgagggctaccgctaccgccaCTGCTGCGGCAGGGGCGTGGAGGGCAGAGGGCCGCGGA
GGCCGCAGTTGCAAACATGGCTCAGAGCAGAGACGGCGGAAACCCGTTCGCCGAGCCCAGCGAGCTTGACAACCCCTT
TCAGCCACCACCAGCCTATGAGCCTCCAGCCCCTGCCCCATTGCCTCCACCCTCAGCTCCCTCCTTGCAGCCCTCGAGAA
AGCTCAGCCCCACAGAACCTAAGAACTATGGCTCATACAGCACTCAGGCCTCAGCTGCAGCAGCCACAGCTGAGCTGCT
GAAGAAACAGGAGGAGCTCAACCGGAAGGCGGAGGAGTTGGACCGAAGGGAGCGAGAGCTGCAGCATGCTGCCCTGG
GAGGCACAGCTACTCGACAGAACAATTGGCCCCCTCTACCTTCTTTTTGTCCAGTTCAGCCCTGCTTTTTCCAGGACATC
TCCATGGAGATCCCCCAAGAATTTCAGAAGACTGTATCCACCATGTACTACCTCTGGATGTTGGCTGGATCTCTGCTCTG
GTGGTGCCGAAGGGCAACACAGCAGTATCCGTGCTCATGCTGCTGGTCGCCCTGCTCTTCACTGGCATTGCTGTGCTAG
GAATTGTCATGCTGAAACGGATCCACTCCTTATACCGCCGCACAGGTGCCAGCTTTCAGAAGGCCCAGCAAGAATTTGC
TGCTGGTGTCTTCTCCAACCCTGCGGTGCGAACCGCAGCTGCCAATGCAGCCGCTGGGGCTGCTGAAAATGCCTTCCGG
GCCCCGTGACCCCTGACTGGGATGCCCTGGCCCTGCTACTTGAGGGAGCTGACTTAGCTCCCGTCCCTAAGGTCTCTGG
GACTTGGAGAGACATCACTAACTGATGGCTCCTCCGTAGTGCTCCCAATCCTATGGCCATGACTGCTGAACCTGACAGG
CGTGTGGGGAGTTCACTGTGACCTAGTCCCCCCATCAGGCCACACTGCTGCCACCTCTCACACGCCCCAACCCAGCTTCC
CTCTGCTGTCACGGCTGTTGcttcggttatttaaataaaaagaaagtggaactggaac

>VIRT38385
MAQSRDGGNPFAEPSELDNPFQPPPAYEPPAPAPLPPPSAPSLQPSRKLSPTEPKNYGSY
STQASAAAATAELLKKQEELNRKAEELDRRERELQHAALGGTATRQNNWPPLPSFCPVQP
CFFQDISMEIPQEFQKTVSTMYYLWMLAGSLLWWCRRATQQYPCSCCWSPCSSLALLC

>NM_005698.3 Homo sapiens secretory carrier membrane protein 3 (SCAMP3), transcript variant 1, mRNA
TGGGTTAGAGGTATCACGTGGGGGCACTTTCGTCTTAGCTTTTGGACAAGACGCAGGCGCAACCCACGGC
TGCTGCGGGGATCCTTGTGGCCCTTCCGGTCGGTGGAACCAATCCGTGCACAGAGAAGCGGGGCGAACTG
AGGCGAGTGAAGTGGACTCTGAGGGCTACCGCTACCGCCACTGCTGCGGCAGGGGCGTGGAGGGCAGAGG
GCCGCGGAGGCCGCAGTTGCAAACATGGCTCAGAGCAGAGACGGCGGAAACCCGTTCGCCGAGCCCAGCG
AGCTTGACAACCCCTTTCAGGACCCAGCTGTGATCCAGCACCGACCCAGCCGGCAGTATGCCACGCTTGA
CGTCTACAACCCTTTTGAGACCCGGGAGCCACCACCAGCCTATGAGCCTCCAGCCCCTGCCCCATTGCCT
CCACCCTCAGCTCCCTCCTTGCAGCCCTCGAGAAAGCTCAGCCCCACAGAACCTAAGAACTATGGCTCAT
ACAGCACTCAGGCCTCAGCTGCAGCAGCCACAGCTGAGCTGCTGAAGAAACAGGAGGAGCTCAACCGGAA
GGCAGAGGAGTTGGACCGAAGGGAGCGAGAGCTGCAGCATGCTGCCCTGGGGGGCACAGCTACTCGACAG
AACAATTGGCCCCCTCTACCTTCTTTTTGTCCAGTTCAGCCCTGCTTTTTCCAGGACATCTCCATGGAGA
TCCCCCAAGAATTTCAGAAGACTGTATCCACCATGTACTACCTCTGGATGTGCAGCACGCTGGCTCTTCT
CCTGAACTTCCTCGCCTGCCTGGCCAGCTTCTGTGTGGAAACCAACAATGGCGCAGGCTTTGGGCTTTCT
ATCCTCTGGGTCCTCCTTTTCACTCCCTGCTCCTTTGTCTGCTGGTACCGCCCCATGTATAAGGCTTTCC
GGAGTGACAGTTCATTCAATTTCTTCGTTTTCTTCTTCATTTTCTTCGTCCAGGATGTGCTCTTTGTCCT
CCAGGCCATTGGTATCCCAGGTTGGGGATTCAGTGGCTGGATCTCTGCTCTGGTGGTGCCGAAGGGCAAC
ACAGCAGTATCCGTGCTCATGCTGCTGGTCGCCCTGCTCTTCACTGGCATTGCTGTGCTAGGAATTGTCA
TGCTGAAACGGATCCACTCCTTATACCGCCGCACAGGTGCCAGCTTTCAGAAGGCCCAGCAAGAATTTGC
TGCTGGTGTCTTCTCCAACCCTGCGGTGCGAACCGCAGCTGCCAATGCAGCCGCTGGGGCTGCTGAAAAT
GCCTTCCGGGCCCCGTGACCCCTGACTGGGATGCCCTGGCCCTGCTACTTGAGGGAGCTGACTTAGCTCC
CGTCCCTAAGGTCTCTGGGACTTGGAGAGACATCACTAACTGATGGCTCCTCCGTAGTGCTCCCAATCCT
ATGGCCATGACTGCTGAACCTGACAGGCGTGTGGGGAGTTCACTGTGACCTAGTCCCCCCATCAGGCCAC
ACTGCTGCCACCTCTCACACGCCCCAACCCAGCTTCCCTCTGCTGTGCCACGGCTGTTGCTTCGGTTATT
TAAATAAAAAGAAAGTGGAACTGGAACTGACAAAAAAAAAAAAAAAAAAAAAAAA

>NP_005689.2 secretory carrier-associated membrane protein 3 isoform 1 [Homo sapiens]
MAQSRDGGNPFAEPSELDNPFQDPAVIQHRPSRQYATLDVYNPFETREPPPAYEPPAPAPLPPPSAPSLQ
PSRKLSPTEPKNYGSYSTQASAAAATAELLKKQEELNRKAEELDRRERELQHAALGGTATRQNNWPPLPS
FCPVQPCFFQDISMEIPQEFQKTVSTMYYLWMCSTLALLLNFLACLASFCVETNNGAGFGLSILWVLLFT
PCSFVCWYRPMYKAFRSDSSFNFFVFFFIFFVQDVLFVLQAIGIPGWGFSGWISALVVPKGNTAVSVLML
LVALLFTGIAVLGIVMLKRIHSLYRRTGASFQKAQQEFAAGVFSNPAVRTAAANAAAGAAENAFRAP

>NM_052837.2 Homo sapiens secretory carrier membrane protein 3 (SCAMP3), transcript variant 2, mRNA
TGGGTTAGAGGTATCACGTGGGGGCACTTTCGTCTTAGCTTTTGGACAAGACGCAGGCGCAACCCACGGC
TGCTGCGGGGATCCTTGTGGCCCTTCCGGTCGGTGGAACCAATCCGTGCACAGAGAAGCGGGGCGAACTG
AGGCGAGTGAAGTGGACTCTGAGGGCTACCGCTACCGCCACTGCTGCGGCAGGGGCGTGGAGGGCAGAGG
GCCGCGGAGGCCGCAGTTGCAAACATGGCTCAGAGCAGAGACGGCGGAAACCCGTTCGCCGAGCCCAGCG
AGCTTGACAACCCCTTTCAGCCACCACCAGCCTATGAGCCTCCAGCCCCTGCCCCATTGCCTCCACCCTC

```
AGCTCCCTCCTTGCAGCCCTCGAGAAAGCTCAGCCCCACAGAACCTAAGAACTATGGCTCATACAGCACT
CAGGCCTCAGCTGCAGCAGCCACAGCTGAGCTGCTGAAGAAACAGGAGGAGCTCAACCGGAAGGCAGAGG
AGTTGGACCGAAGGGAGCGAGAGCTGCAGCATGCTGCCCTGGGGGGCACAGCTACTCGACAGAACAATTG
GCCCCCTCTACCTTCTTTTTGTCCAGTTCAGCCCTGCTTTTTCCAGGACATCTCCATGGAGATCCCCCAA
GAATTTCAGAAGACTGTATCCACCATGTACTACCTCTGGATGTGCAGCACGCTGGCTCTTCTCCTGAACT
TCCTCGCCTGCCTGGCCAGCTTCTGTGTGGAAACCAACAATGGCGCAGGCTTTGGGCTTTCTATCCTCTG
GGTCCTCCTTTTCACTCCCTGCTCCTTTGTCTGCTGGTACCGCCCCATGTATAAGGCTTTCCGGAGTGAC
AGTTCATTCAATTTCTTCGTTTTCTTCTTCATTTTCTTCGTCCAGGATGTGCTCTTTGTCCTCCAGGCCA
TTGGTATCCCAGGTTGGGGATTCAGTGGCTGGATCTCTGCTCTGGTGGTGCCGAAGGGCAACACAGCAGT
ATCCGTGCTCATGCTGCTGGTCGCCCTGCTCTTCACTGGCATTGCTGTGCTAGGAATTGTCATGCTGAAA
CGGATCCACTCCTTATACCGCCGCACAGGTGCCAGCTTTCAGAAGGCCCAGCAAGAATTTGCTGCTGGTG
TCTTCTCCAACCCTGCGGTGCGAACCGCAGCTGCCAATGCAGCCGCTGGGGCTGCTGAAAATGCCTTCCG
GGCCCCGTGACCCCTGACTGGGATGCCCTGGCCCTGCTACTTGAGGGAGCTGACTTAGCTCCCGTCCCTA
AGGTCTCTGGGACTTGGAGAGACATCACTAACTGATGGCTCCTCCGTAGTGCTCCCAATCCTATGGCCAT
GACTGCTGAACCTGACAGGCGTGTGGGGAGTTCACTGTGACCTAGTCCCCCCATCAGGCCACACTGCTGC
CACCTCTCACACGCCCCAACCCAGCTTCCCTCTGCTGTGCCACGGCTGTTGCTTCGGTTATTTAAATAAA
AAGAAAGTGGAACTGGAACTGACAAAAAAAAAAAAAAAAAAAAAAAA
```

```
>NP_443069.1 secretory carrier-associated membrane protein 3 isoform 2 [Homo sapiens]
MAQSRDGGNPFAEPSELDNPFQPPPAYEPPAPAPLPPPSAPSLQPSRKLSPTEPKNYGSYSTQASAAAAT
AELLKKQEELNRKAEELDRRERELQHAALGGTATRQNNWPPLPSFCPVQPCFFQDISMEIPQEFQKTVST
MYYLWMCSTLALLLNFLACLASFCVETNNGAGFGLSILWVLLFTPCSFVCWYRPMYKAFRSDSSFNFFVF
FFIFFVQDVLFVLQAIGIPGWGFSGWISALVVPKGNTAVSVLMLLVALLFTGIAVLGIVMLKRIHSLYRR
TGASFQKAQQEFAAGVFSNPAVRTAAANAAAGAAENAFRAP
```

```
i1_HQ_samplec9816d|c29753/f2p0/1106      ------------------------------------------------------------ 0
NM_052837.2                              TGGGTTAGAGGTATCACGTGGGGGCACTTTCGTCTTAGCTTTTGGACAAGACGCAGGCGC 60
i1_HQ_samplec9816d|c20030/f5p0/1447      ------------------------------------------------------------ 0
NM_005698.3                              TGGGTTAGAGGTATCACGTGGGGGCACTTTCGTCTTAGCTTTTGGACAAGACGCAGGCGC 60

i1_HQ_samplec9816d|c29753/f2p0/1106      -----------------------------------------------------------a 1
NM_052837.2                              AACCCACGGCTGCTGCGGGGATCCTTGTGGCCCTTCCGGTCGGTGGAACCAATCCGTGCA 120
i1_HQ_samplec9816d|c20030/f5p0/1447      ------------------------------------------------------------ 0
NM_005698.3                              AACCCACGGCTGCTGCGGGGATCCTTGTGGCCCTTCCGGTCGGTGGAACCAATCCGTGCA 120

i1_HQ_samplec9816d|c29753/f2p0/1106      cagagaagcggggcgaactgaggcgagtgaagtggactctgagggctaccgctaccgcca 61
NM_052837.2                              CAGAGAAGCGGGGCGAACTGAGGCGAGTGAAGTGGACTCTGAGGGCTACCGCTACCGCCA 180
i1_HQ_samplec9816d|c20030/f5p0/1447      -agagaagcggggcgaactgaggcgagtgaagtggactctgagggctaccgctaccgCCA 59
NM_005698.3                              CAGAGAAGCGGGGCGAACTGAGGCGAGTGAAGTGGACTCTGAGGGCTACCGCTACCGCCA 180
                                          ***********************************************************

i1_HQ_samplec9816d|c29753/f2p0/1106      CTGCTGCGGCAGGGGCGTGGAGGGCAGAGGGCCGCGGAGGCCGCAGTTGCAAACATGGCT 121
NM_052837.2                              CTGCTGCGGCAGGGGCGTGGAGGGCAGAGGGCCGCGGAGGCCGCAGTTGCAAACATGGCT 240
i1_HQ_samplec9816d|c20030/f5p0/1447      CTGCTGCGGCAGGGGCGTGGAGGGCAGAGGGCCGCGGAGGCCGCAGTTGCAAACATGGCT 119
NM_005698.3                              CTGCTGCGGCAGGGGCGTGGAGGGCAGAGGGCCGCGGAGGCCGCAGTTGCAAACATGGCT 240
                                         ************************************************************

i1_HQ_samplec9816d|c29753/f2p0/1106      CAGAGCAGAGACGGCGGAAACCCGTTCGCCGAGCCCAGCGAGCTTGACAACCCCTTTCAG 181
NM_052837.2                              CAGAGCAGAGACGGCGGAAACCCGTTCGCCGAGCCCAGCGAGCTTGACAACCCCTTTCAG 300
i1_HQ_samplec9816d|c20030/f5p0/1447      CAGAGCAGAGACGGCGGAAACCCGTTCGCCGAGCCCAGCGAGCTTGACAACCCCTTTCAG 179
NM_005698.3                              CAGAGCAGAGACGGCGGAAACCCGTTCGCCGAGCCCAGCGAGCTTGACAACCCCTTTCAG 300
                                         ************************************************************

i1_HQ_samplec9816d|c29753/f2p0/1106      C----------------------------------------------------------- 182
NM_052837.2                              C----------------------------------------------------------- 301
i1_HQ_samplec9816d|c20030/f5p0/1447      GACCCAGCTGTGATCCAGCACCGACCCAGCCGGCAGTATGCCACGCTTGACGTCTACAAC 239
NM_005698.3                              GACCCAGCTGTGATCCAGCACCGACCCAGCCGGCAGTATGCCACGCTTGACGTCTACAAC 360

i1_HQ_samplec9816d|c29753/f2p0/1106      -------------------CACCACCAGCCTATGAGCCTCCAGCCCCTGCCCCATTGCCT 223
NM_052837.2                              -------------------CACCACCAGCCTATGAGCCTCCAGCCCCTGCCCCATTGCCT 342
i1_HQ_samplec9816d|c20030/f5p0/1447      CCTTTTGAGACCCGGGAGCCACCACCAGCCTATGAGCCTCCAGCCCCTGCCCCATTGCCT 299
NM_005698.3                              CCTTTTGAGACCCGGGAGCCACCACCAGCCTATGAGCCTCCAGCCCCTGCCCCATTGCCT 420
                                                            *****************************************

i1_HQ_samplec9816d|c29753/f2p0/1106      CCACCCTCAGCTCCCTCCTTGCAGCCCTCGAGAAAGCTCAGCCCCACAGAACCTAAGAAC 283
NM_052837.2                              CCACCCTCAGCTCCCTCCTTGCAGCCCTCGAGAAAGCTCAGCCCCACAGAACCTAAGAAC 402
i1_HQ_samplec9816d|c20030/f5p0/1447      CCACCCTCAGCTCCCTCCTTGCAGCCCTCGAGAAAGCTCAGCCCCACAGAACCTAAGAAC 359
NM_005698.3                              CCACCCTCAGCTCCCTCCTTGCAGCCCTCGAGAAAGCTCAGCCCCACAGAACCTAAGAAC 480
                                         ************************************************************
```

```
i1_HQ_samplec9816d|c29753/f2p0/1106        TATGGCTCATACAGCACTCAGGCCTCAGCTGCAGCAGCCACAGCTGAGCTGCTGAAGAAA 343
NM_052837.2                                TATGGCTCATACAGCACTCAGGCCTCAGCTGCAGCAGCCACAGCTGAGCTGCTGAAGAAA 462
i1_HQ_samplec9816d|c20030/f5p0/1447        TATGGCTCATACAGCACTCAGGCCTCAGCTGCAGCAGCCACAGCTGAGCTGCTGAAGAAA 419
NM_005698.3                                TATGGCTCATACAGCACTCAGGCCTCAGCTGCAGCAGCCACAGCTGAGCTGCTGAAGAAA 540
                                           ************************************************************

i1_HQ_samplec9816d|c29753/f2p0/1106        CAGGAGGAGCTCAACCGGAAGGCGGAGGAGTTGGACCGAAGGGAGCGAGAGCTGCAGCAT 403
NM_052837.2                                CAGGAGGAGCTCAACCGGAAGGCAGAGGAGTTGGACCGAAGGGAGCGAGAGCTGCAGCAT 522
i1_HQ_samplec9816d|c20030/f5p0/1447        CAGGAGGAGCTCAACCGGAAGGCAGAGGAGTTGGACCGAAGGGAGCGAGAGCTGCAGCAT 479
NM_005698.3                                CAGGAGGAGCTCAACCGGAAGGCAGAGGAGTTGGACCGAAGGGAGCGAGAGCTGCAGCAT 600
                                           *********************** ************************************

i1_HQ_samplec9816d|c29753/f2p0/1106        GCTGCCCTGGGAGGCACAGCTACTCGACAGAACAATTGGCCCCCTCTACCTTCTTTTTGT 463
NM_052837.2                                GCTGCCCTGGGGGGCACAGCTACTCGACAGAACAATTGGCCCCCTCTACCTTCTTTTTGT 582
i1_HQ_samplec9816d|c20030/f5p0/1447        GCTGCCCTGGGAGGCACAGCTACTCGACAGAACAATTGGCCCCCTCTACCTTCTTTTTGT 539
NM_005698.3                                GCTGCCCTGGGGGGCACAGCTACTCGACAGAACAATTGGCCCCCTCTACCTTCTTTTTGT 660
                                           *********** ************************************************

i1_HQ_samplec9816d|c29753/f2p0/1106        CCAGTTCAGCCCTGCTTTTTCCAGGACATCTCCATGGAGATCCCCCAAGAATTTCAGAAG 523
NM_052837.2                                CCAGTTCAGCCCTGCTTTTTCCAGGACATCTCCATGGAGATCCCCCAAGAATTTCAGAAG 642
i1_HQ_samplec9816d|c20030/f5p0/1447        CCAGTTCAGCCCTGCTTTTTCCAGGACATCTCCATGGAGATCCCCCAAGAATTTCAGAAG 599
NM_005698.3                                CCAGTTCAGCCCTGCTTTTTCCAGGACATCTCCATGGAGATCCCCCAAGAATTTCAGAAG 720
                                           ************************************************************

i1_HQ_samplec9816d|c29753/f2p0/1106        ACTGTATCCACCATGTACTACCTCTGGATGTT---------------------------- 555
NM_052837.2                                ACTGTATCCACCATGTACTACCTCTGGATGTGCAGCACGCTGGCTCTTCTCCTGAACTTC 702
i1_HQ_samplec9816d|c20030/f5p0/1447        ACTGTATCCACCATGTACTACCTCTGGATGTGCAGCACGCTGGCTCTTCTCCTGAACTTC 659
NM_005698.3                                ACTGTATCCACCATGTACTACCTCTGGATGTGCAGCACGCTGGCTCTTCTCCTGAACTTC 780
                                           *******************************

i1_HQ_samplec9816d|c29753/f2p0/1106        ------------------------------------------------------------ 555
NM_052837.2                                CTCGCCTGCCTGGCCAGCTTCTGTGTGGAAACCAACAATGGCGCAGGCTTTGGGCTTTCT 762
i1_HQ_samplec9816d|c20030/f5p0/1447        CTCGCCTGCCTGGCCAGCTTCTGTGTGGAAACCAACAATGGCGCAGGCTTTGGGCTTTCT 719
NM_005698.3                                CTCGCCTGCCTGGCCAGCTTCTGTGTGGAAACCAACAATGGCGCAGGCTTTGGGCTTTCT 840

i1_HQ_samplec9816d|c29753/f2p0/1106        ------------------------------------------------------------ 555
NM_052837.2                                ATCCTCTGGGTCCTCCTTTTCACTCCCTGCTCCTTTGTCTGCTGGTACCGCCCCATGTAT 822
i1_HQ_samplec9816d|c20030/f5p0/1447        ATCCTCTGGGTCCTCCTTTTCACTCCCTGCTCCTTTGTCTGCTGGTACCGCCCCATGTAT 779
NM_005698.3                                ATCCTCTGGGTCCTCCTTTTCACTCCCTGCTCCTTTGTCTGCTGGTACCGCCCCATGTAT 900

i1_HQ_samplec9816d|c29753/f2p0/1106        ------------------------------------------------------------ 555
NM_052837.2                                AAGGCTTTCCGGAGTGACAGTTCATTCAATTTCTTCGTTTTCTTCTTCATTTTCTTCGTC 882
i1_HQ_samplec9816d|c20030/f5p0/1447        AAGGCTTTCCGGAGTGACAGTTCATTCAATTTCTTCGTTTTCTTCTTCATTTTCTTCGTC 839
NM_005698.3                                AAGGCTTTCCGGAGTGACAGTTCATTCAATTTCTTCGTTTTCTTCTTCATTTTCTTCGTC 960

i1_HQ_samplec9816d|c29753/f2p0/1106        ------------------------------------------------------GGCTGG 561
NM_052837.2                                CAGGATGTGCTCTTTGTCCTCCAGGCCATTGGTATCCCAGGTTGGGGATTCAGTGGCTGG 942
i1_HQ_samplec9816d|c20030/f5p0/1447        CAGGATGTGCTCTTTGTCCTCCAGGCCATTGGTATCCCAGGTTGGGGATTCAGTGGCTGG 899
NM_005698.3                                CAGGATGTGCTCTTTGTCCTCCAGGCCATTGGTATCCCAGGTTGGGGATTCAGTGGCTGG 1020
                                                                                                 ******

i1_HQ_samplec9816d|c29753/f2p0/1106        ATCTCTGCTCTGGTGGTGCCGAAGGGCAACACAGCAGTATCCGTGCTCATGCTGCTGGTC 621
NM_052837.2                                ATCTCTGCTCTGGTGGTGCCGAAGGGCAACACAGCAGTATCCGTGCTCATGCTGCTGGTC 1002
i1_HQ_samplec9816d|c20030/f5p0/1447        ATCTCTGCTCTGGTGGTGCCGAAGGGCAACACAGCAGTATCCGTGCTCATGCTGCTGGTC 959
NM_005698.3                                ATCTCTGCTCTGGTGGTGCCGAAGGGCAACACAGCAGTATCCGTGCTCATGCTGCTGGTC 1080
                                           ************************************************************

i1_HQ_samplec9816d|c29753/f2p0/1106        GCCCTGCTCTTCACTGGCATTGCTGTGCTAGGAATTGTCATGCTGAAACGGATCCACTCC 681
NM_052837.2                                GCCCTGCTCTTCACTGGCATTGCTGTGCTAGGAATTGTCATGCTGAAACGGATCCACTCC 1062
i1_HQ_samplec9816d|c20030/f5p0/1447        GCCCTGCTCTTCACTGGCATTGCTGTGCTAGGAATTGTCATGCTGAAACGGATCCACTCC 1019
NM_005698.3                                GCCCTGCTCTTCACTGGCATTGCTGTGCTAGGAATTGTCATGCTGAAACGGATCCACTCC 1140
                                           ************************************************************

i1_HQ_samplec9816d|c29753/f2p0/1106        TTATACCGCCGCACAGGTGCCAGCTTTCAGAAGGCCCAGCAAGAATTTGCTGCTGGTGTC 741
NM_052837.2                                TTATACCGCCGCACAGGTGCCAGCTTTCAGAAGGCCCAGCAAGAATTTGCTGCTGGTGTC 1122
i1_HQ_samplec9816d|c20030/f5p0/1447        TTATACCGCCGCACAGGTGCCAGCTTTCAGAAGGCCCAGCAAGAATTTGCTGCTGGTGTC 1079
NM_005698.3                                TTATACCGCCGCACAGGTGCCAGCTTTCAGAAGGCCCAGCAAGAATTTGCTGCTGGTGTC 1200
                                           ************************************************************

i1_HQ_samplec9816d|c29753/f2p0/1106        TTCTCCAACCCTGCGGTGCGAACCGCAGCTGCCAATGCAGCCGCTGGGGCTGCTGAAAAT 801
NM_052837.2                                TTCTCCAACCCTGCGGTGCGAACCGCAGCTGCCAATGCAGCCGCTGGGGCTGCTGAAAAT 1182
i1_HQ_samplec9816d|c20030/f5p0/1447        TTCTCCAACCCTGCGGTGCGAACCGCAGCTGCCAATGCAGCCGCTGGGGCTGCTGAAAAT 1139
NM_005698.3                                TTCTCCAACCCTGCGGTGCGAACCGCAGCTGCCAATGCAGCCGCTGGGGCTGCTGAAAAT 1260
                                           ************************************************************

i1_HQ_samplec9816d|c29753/f2p0/1106        GCCTTCCGGGCCCCGTGACCCCTGACTGGGATGCCCTGGCCCTGCTACTTGAGGGAGCTG 861
NM_052837.2                                GCCTTCCGGGCCCCGTGACCCCTGACTGGGATGCCCTGGCCCTGCTACTTGAGGGAGCTG 1242
i1_HQ_samplec9816d|c20030/f5p0/1447        GCCTTCCGGGCCCCGTGACCCCTGACTGGGATGCCCTGGCCCTGCTACTTGAGGGAGCTG 1199
NM_005698.3                                GCCTTCCGGGCCCCGTGACCCCTGACTGGGATGCCCTGGCCCTGCTACTTGAGGGAGCTG 1320
```

```
                                     ************************************************************

i1_HQ_samplec9816d|c29753/f2p0/1106     ACTTAGCTCCCGTCCCTAAGGTCTCTGGGACTTGGAGAGACATCACTAACTGATGGCTCC 921
NM_052837.2                             ACTTAGCTCCCGTCCCTAAGGTCTCTGGGACTTGGAGAGACATCACTAACTGATGGCTCC 1302
i1_HQ_samplec9816d|c20030/f5p0/1447     ACTTAGCTCCCGTCCCTAAGGTCTCTGGGACTTGGAGAGACATCACTAACTGATGGCTCC 1259
NM_005698.3                             ACTTAGCTCCCGTCCCTAAGGTCTCTGGGACTTGGAGAGACATCACTAACTGATGGCTCC 1380
                                        ************************************************************

i1_HQ_samplec9816d|c29753/f2p0/1106     TCCGTAGTGCTCCCAATCCTATGGCCATGACTGCTGAACCTGACAGGCGTGTGGGGAGTT 981
NM_052837.2                             TCCGTAGTGCTCCCAATCCTATGGCCATGACTGCTGAACCTGACAGGCGTGTGGGGAGTT 1362
i1_HQ_samplec9816d|c20030/f5p0/1447     TCCGTAGTGCTCCCAATCCTATGGCCATGACTGCTGAACCTGACAGGCGTGTGGGGAGTT 1319
NM_005698.3                             TCCGTAGTGCTCCCAATCCTATGGCCATGACTGCTGAACCTGACAGGCGTGTGGGGAGTT 1440
                                        ************************************************************

i1_HQ_samplec9816d|c29753/f2p0/1106     CACTGTGACCTAGTCCCCCCATCAGGCCACACTGCTGCCACCTCTCACACGCCCCAACCC 1041
NM_052837.2                             CACTGTGACCTAGTCCCCCCATCAGGCCACACTGCTGCCACCTCTCACACGCCCCAACCC 1422
i1_HQ_samplec9816d|c20030/f5p0/1447     CACTGTGACCTAGTCCCCCCATCAGGCCACACTGCTGCCACCTCTCACACGCCCCAACCC 1379
NM_005698.3                             CACTGTGACCTAGTCCCCCCATCAGGCCACACTGCTGCCACCTCTCACACGCCCCAACCC 1500
                                        ************************************************************

i1_HQ_samplec9816d|c29753/f2p0/1106     AGCTTCCCTCTGCTGT--CACGGCTGTTGcttcggttatttaaataaaaagaaagtggaa 1099
NM_052837.2                             AGCTTCCCTCTGCTGTGCCACGGCTGTTGCTTCGGTTATTTAAATAAAAAGAAAGTGGAA 1482
i1_HQ_samplec9816d|c20030/f5p0/1447     AGCTTCCCTCTGCTGTGCCACGGCTGTTGCTTCGGTTatttaaataaaaagaaagtggaa 1439
NM_005698.3                             AGCTTCCCTCTGCTGTGCCACGGCTGTTGCTTCGGTTATTTAAATAAAAAGAAAGTGGAA 1560
                                        ****************  ******************************************

i1_HQ_samplec9816d|c29753/f2p0/1106     ctggaac---------------------------      1106
NM_052837.2                             CTGGAACTGACAAAAAAAAAAAAAAAAAAAAAAA      1517
i1_HQ_samplec9816d|c20030/f5p0/1447     ctggaact--------------------------      1447
NM_005698.3                             CTGGAACTGACAAAAAAAAAAAAAAAAAAAAAAA      1595
                                        *******
```

```
VIRT31885        MAQSRDGGNPFAEPSELDNPFQDPAVIQHRPSRQYATLDVYNPFETREPPPAYEPPAPAP     60
NP_005689.2      MAQSRDGGNPFAEPSELDNPFQDPAVIQHRPSRQYATLDVYNPFETREPPPAYEPPAPAP     60
VIRT38385        MAQSRDGGNP--------------------------FAEPSELDNPFQPPPAYEPPAPAP     34
NP_443069.1      MAQSRDGGNP--------------------------FAEPSELDNPFQPPPAYEPPAPAP     34
                 **********                                      ************

VIRT31885        LPPPSAPSLQPSRKLSPTEPKNYGSYSTQASAAAATAELLKKQEELNRKAEELDRREREL     120
NP_005689.2      LPPPSAPSLQPSRKLSPTEPKNYGSYSTQASAAAATAELLKKQEELNRKAEELDRREREL     120
VIRT38385        LPPPSAPSLQPSRKLSPTEPKNYGSYSTQASAAAATAELLKKQEELNRKAEELDRREREL     94
NP_443069.1      LPPPSAPSLQPSRKLSPTEPKNYGSYSTQASAAAATAELLKKQEELNRKAEELDRREREL     94
                 ************************************************************

VIRT31885        QHAALGGTATRQNNWPPLPSFCPVQPCFFQDISMEIPQEFQKTVSTMYYLWMCSTLALLL     180
NP_005689.2      QHAALGGTATRQNNWPPLPSFCPVQPCFFQDISMEIPQEFQKTVSTMYYLWMCSTLALLL     180
VIRT38385        QHAALGGTATRQNNWPPLPSFCPVQPCFFQDISMEIPQEFQKTVSTMYYLWMLAGSLLWW     154
NP_443069.1      QHAALGGTATRQNNWPPLPSFCPVQPCFFQDISMEIPQEFQKTVSTMYYLWMCSTLALLL     154
                 ****************************************************     *

VIRT31885        NFLACLASFCVETNNGAGFGLSILWVLLFTPCSFVCWYRPMYKAFRSDSSFNFFVFFFIF     240
NP_005689.2      NFLACLASFCVETNNGAGFGLSILWVLLFTPCSFVCWYRPMYKAFRSDSSFNFFVFFFIF     240
VIRT38385        CRRATQ-----------------------QYPCSCCWSPCSSLALLC-------------     178
NP_443069.1      NFLACLASFCVETNNGAGFGLSILWVLLFTPCSFVCWYRPMYKAFRSDSSFNFFVFFFIF     214
                    *                               **      *

VIRT31885        FVQDVLFVLQAIGIPGWGFSGWISALVVPKGNTAVSVLMLLVALLFTGIAVLGIVMLKRI     300
NP_005689.2      FVQDVLFVLQAIGIPGWGFSGWISALVVPKGNTAVSVLMLLVALLFTGIAVLGIVMLKRI     300
VIRT38385        ------------------------------------------------------------     178
NP_443069.1      FVQDVLFVLQAIGIPGWGFSGWISALVVPKGNTAVSVLMLLVALLFTGIAVLGIVMLKRI     274


VIRT31885        HSLYRRTGASFQKAQQEFAAGVFSNPAVRTAAANAAAGAAENAFRAP       347
NP_005689.2      HSLYRRTGASFQKAQQEFAAGVFSNPAVRTAAANAAAGAAENAFRAP       347
VIRT38385        -----------------------------------------------       178
NP_443069.1      HSLYRRTGASFQKAQQEFAAGVFSNPAVRTAAANAAAGAAENAFRAP       321
```

**RAB24**

| **Sequence** | **Header** | **AA Seq** |
|---|---|---|
| **Present** | i1_HQ_samplec9816d\|c63433/f11p0/1318 | >VIRT1094 |
| | i1_HQ_samplec9816d\|c43648/f2p0/1435 | VIRT2751 |
| **Absent** | i0_HQ_samplec9816d\|c43092/f2p19/798 | VIRT5954 |
| **NCBI** | NM_001031677 | NP_001026847.1 |
| | NM_130781 | NP_570137.2 |
| | NR_109789 | Protein not available in NCBI |

>i1_HQ_samplec9816d|c63433/f11p0/1318
isoform=c63433;full_length_coverage=11;non_full_length_coverage=0;isoform_length=1318

gtcccgcgggagtcgcggcgctgcgggtaggagccgggttgcgggagaccccaggttcggttgggaTTCCCAGCCAGAACGGAGCTTAAGCCGGGCAGGCG
AGCGAATGACGGAGTAGCGAGCTGCACGGCGGCGTGCTGCGCTGTTGAGGACGCTGTCCCGCGCGCTCCCAGGCCGCC
CCGAGGCTTGGGGTCTTCGAAGGATAATCGGCGCCCGGGGCCGAACAGCGGGGGCACACGGGGCGCTGCCGAAGTGCA
AGGCCACGGCCAGAGCTCGAGCCCGACGCGCTGTCTGGAGTCGTAGGTTGGCGCCGTTTGGGGTCGGGGTCTGAGGCTT
GGGCGCTGCCTGGGCCGAGCGGAGATCGGGGTTTGCCTCCCGTCCCCGCTCAGGACCCTGACGTGGCTGAAGCGGCCCC
GGGAGCATGAGCGGGCAGCGCGTGGACGTCAAGGTGGTGATGCTGGGCAAGGAGTACGTGGGCAAGACTAGCCTGGT
GGAGCGCTACGTGCACGACCGCTTTCTGGTGGGGCCTTATCAGAACACCATCGGGGCCGCCTTCGTGGCCAAGGTGATG
TCGGTCGGAGACCGGACTGTGACATTAGGTATTTGGGACACAGCAGGCTCTGAGCGCTATGAGGCCATGAGTAGAATCT
ACTATCGGGGTGCCAAGGCTGCCATCGTCTGCTATGACCTCACAGACAGCAGCAGCTTTGAGCGAGCAAAGTTCTGGGT
GAAGGAACTGCGCAGCCTAGAGGAGGGCTGCCAAATCTACTTATGTGGCACCAAGAGTGACCTGCTGGAAGAAGACCG
GAGGCGTCGACGTGTGGACTTCCACGACGTCCAGGACTATGCAGACAATATCAAAGCTCAGCTCTTTGAAACATCCAGC
AAGACAGGCCAGAGTGTGGACGAGCTCTTCCAGAAAGTGGCAGAGGATTACGTCAGTGTGGCTGCCTTCCAGGTGATG
ACAGAGGACAAGGGCGTGGATCTGGGCCAGAAGCCAAACCCCTACTTCTACAGCTGTTGTCATCACTGAGTCAGCACTC
ACCTGGCCTGGGGGAATTAAAGGAATTCCCCGTAAGGGCTGGACCCAGCTCCTTTCTGGGCTTGGGTAGTCAAATGTCT
GAGCTACCCCAGGTCCTCATGTCAGCAGAGTGGCGCCTGCCTGTGCTGGCCCATGGAACGGAGACAGCATTGGGCTGAC
TGTGGGCATGAGGAGGGATAAGGCTGATTTGGACCCCAGGCTTCTGCCCTGGACAGCACTTGTGTCTGCAGATTATTTA
AGTGGCTTTTGatctgtaaataaaatcagtgcactgtg

>VIRT1094
MSGQRVDVKVVMLGKEYVGKTSLVERYVHDRFLVGPYQNTIGAAFVAKVMSVGDRTVTLG
IWDTAGSERYEAMSRIYYRGAKAAIVCYDLTDSSSFERAKFWVKELRSLEEGCQIYLCGT
KSDLLEEDRRRRRVDFHDVQDYADNIKAQLFETSSKTGQSVDELFQKVAEDYVSVAAFQV
MTEDKGVDLGQKPNPYFYSCCHH

>i1_HQ_samplec9816d|c43648/f2p0/1435
isoform=c43648;full_length_coverage=2;non_full_length_coverage=0;isoform_length=1435

gcggcgctgcgggtaggagccgggttgcgggagaccccaggttcggttgggattcccagccaGAACGGAGCTTAAGCCGGGCAGGCGAGCGAATGACGGA
GTAGCGAGCTGCACGGCGGCGTGCTGCGCTGTTGAGGACGCTGTCCCGCGCGCTCCCAGGCCGCCCCGAGGCTTGGGGT
CTTCGAAGGATAATCGGCGCCCGGGGCCGAACAGCGGGGGCACACGGGGCGCTGCCGAAGTGCAAGGCCACGGCCAG
AGCTCGAGCCCGACGCGCTGTCTGGAGTCGTAGGACCCTGACGTGGCTGAAGCGGCCCCGGGAGCATGAGCGGGCAGC
GCGTGGACGTCAAGGTGGTGATGCTGGGCAAGGAGTACGTGGGCAAGACTAGCCTGGTGGAGCGCTACGTGCACGACC
GCTTTCTGGTGGGGCCTTATCAGAACACCATCGGGGCCGCCTTCGTGGCCAAGGTGATGTCGGTCGGAGACCGGACTGT
GACATTAGGTATTTGGGACACAGCAGGCTCTGAGCGCTATGAGGCCATGAGTAGAATCTACTATCGGGGTGCCAAGGCT
GCCATCGTCTGCTATGACCTCACAGACAGCAGCAGCTTTGAGCGAGCAAAGTTCTGGGTGAAGGAACTGCGCAGCCTA
GAGGAGGGCTGCCAAATCTACTTATGTGGCACCAAGAGTGACCTGCTGGAAGAAGACCGGAGGCGTCGACGTGTGGAC
TTCCACGACGTCCAGGACTATGCAGACAGTAGCTGCTCCTCAGCCCTTTGGGGGGTGGGGGTGTGTGGCTGTCTGGGTG
GATCAAAGAAAATAGGGACTGCCTTGGCTGCCAGGGCAAGGTGCTCTAGGAGGTCTTCCTGGCCTCCTTGAACTGTGGG
GTCCAGGAGACTCCCTGAACTGCTAGCCCTCCCTTTTGTCTGTTTATCTAATTCTCAGGTATGAGGCTTTAGTCACTTCTC
TTTACAGATATCAAAGCTCAGCTCTTTGAAACATCCAGCAAGACAGGCCAGAGTGTGGACGAGCTCTTCCAGAAAGTGG
CAGAGGATTACGTCAGTGTGGCTGCCTTCCAGGTGATGACAGAGGACAAGGGCGTGGATCTGGGCCAGAAGCCAAACC
CCTACTTCTACAGCTGTTGTCATCACTGAGTCAGCACTCACCTGGCCTGGGGGAATTAAAGGAATTCCCCGTAAGGGCT
GGACCCAGCTCCTTTCTGGGCTTGGGTAGTCAAATGTCTGAGCTACCCCAGGTCCTCATGTCAGCAGAGTGGCGCCTGC
CTGTGCTGGCCCATGGAACGGAGACAGCATTGGGCTGACTGTGGGCATGAGGAGGGATAAGGCTGATTTGGACCCCAG
GCTTCTGCCCTGGACAGCACTTGTGTCTGCAGATTATTTAAgtggcttttgatctgtaaataaaatcagtgcactgt

>VIRT2751
MSGQRVDVKVVMLGKEYVGKTSLVERYVHDRFLVGPYQNTIGAAFVAKVMSVGDRTVTLG
IWDTAGSERYEAMSRIYYRGAKAAIVCYDLTDSSSFERAKFWVKELRSLEEGCQIYLCGT
KSDLLEEDRRRRRVDFHDVQDYADSSCSSALWGVGVCGCLGGSKKIGTALAARARCSRRS
SWPP
>i0_HQ_samplec9816d|c43092/f2p19/798
isoform=c43092;full_length_coverage=2;non_full_length_coverage=19;isoform_length=798

ggagtcgcggcgctgcggACCATCGGGGCCGCCTTCGTGGCCAAGGTGATGTCGGTCGGAGACCGGACTGTGACATTAGGTATTT
GGGACACAGCAGGCTCTGAGCGCTATGAGGCCATGAGTAGAATCTACTATCGGGGTGCCAAGGCTGCCATCGTCTGCTA
TGACCTCACAGACAGCAGCAGCTTTGAGCGAGCAAAGTTCTGGGTGAAGGAACTGCGCAGCCTAGAGGAGGGCTGCCA
AATCTACTTATGTGGCACCAAGAGTGACCTGCTGGAAGAAGACCGGAGGCGTCGACGTGTGGACTTCCACGACGTCCA
GGACTATGCAGACAATATCAAAGCTCAGCTCTTTGAAACATCCAGCAAGACAGGCCAGAGTGTGGACGAGCTCTTCCA
GAAAGTGGCAGAGGATTACGTCAGTGTGGCTGCCTTCCAGGTGATGACAGAGGACAAGGGCGTGGATCTGGGCCAGAA
GCCAAACCCCTACTTCTACAGCTGTTGTCATCACTGAGTCAGCACTCACCTGGCCTGGGGGAATTAAAGGAATTCCCCG
TAAGGGCTGGACCCAGCTCCTTTCTGGGCTTGGGTAGTCAAATGTCTGAGCTACCCCAGGTCCTCATGTCAGCAGAGTG
GCGCCTGCCTGTGCTGGCCCATGGAACGGAGACAGCATTGGGCTGACTGTGGGCATGAGGAGGGATAAGGCTGATTTG
GACCCCAGGCTTCTGCCCTGGACAGCACTTGTGTCTGCAGATTATTTAAGTGGCTTTTGATCTGTAAATAAAATCAGTGC
ACTGTG

>VIRT5954
MSVGDRTVTLGIWDTAGSERYEAMSRIYYRGAKAAIVCYDLTDSSSFERAKFWVKELRSL
EEGCQIYLCGTKSDLLEEDRRRRRVDFHDVQDYADNIKAQLFETSSKTGQSVDELFQKVA
EDYVSVAAFQVMTEDKGVDLGQKPNPYFYSCCHH
>NM_001031677.3 Homo sapiens RAB24, member RAS oncogene family (RAB24), transcript variant 1, mRNA
GACCTTGCGGCCCCGCCCCCTCGCCCTCTAGCCCCCTCCCGCGGGAGTCGCGGCGCTGCGGGTAGGAGCC
GGGTTGCGGGAGACCCCAGGTTCGGTTGGGATTCCCAGCCAGAACGGAGCTTAAGCCGGGCAGGCGAGCG
AATGACGGAGTAGCGAGCTGCACGGCGGCGTGCTGCGCTGTTGAGGACGCTGTCCCGCGCGCTCCCAGGC
CGCCCCGAGGCTTGGGGTCTTCGAAGGATAATCGGCGCCCGGGGCCGAACAGCGGGGGCACACGGGGCGC
TGCCGAAGTGCAAGGCCACGGCCAGAGCTCGAGCCCGACGCGCTGTCTGGAGTCGTAGGTTGGCGCCGTT
TGGGGTCGGGGTCTGAGGCTTGGGCGCTGCCTGGGCCGAGCGGAGATCGGGGTTTGCCTCCCGTCCCCGC
TCAGGACCCTGACGTGGCTGAAGCGGCCCCGGGAGCATGAGCGGGCAGCGCGTGGACGTCAAGGTGGTGA
TGCTGGGCAAGGAGTACGTGGGCAAGACTAGCCTGGTGGAGCGCTACGTGCACGACCGCTTTCTGGTGGG
GCCTTATCAGAACACCATCGGGGCCGCCTTCGTGGCCAAGGTGATGTCGGTCGGAGACCGGACTGTGACA
TTAGGTATTTGGGACACAGCAGGCTCTGAGCGCTATGAGGCCATGAGTAGAATCTACTATCGGGGTGCCA
AGGCTGCCATCGTCTGCTATGACCTCACAGACAGCAGCAGCTTTGAGCGAGCAAAGTTCTGGGTGAAGGA
ACTGCGCAGCCTAGAGGAGGGCTGCCAAATCTACTTATGTGGCACCAAGAGTGACCTGCTGGAAGAAGAC
CGGAGGCGTCGACGTGTGGACTTCCACGACGTCCAGGACTATGCAGACAATATCAAAGCTCAGCTCTTTG
AAACATCCAGCAAGACAGGCCAGAGTGTGGACGAGCTCTTCCAGAAAGTGGCAGAGGATTACGTCAGTGT
GGCTGCCTTCCAGGTGATGACAGAGGACAAGGGCGTGGATCTGGGCCAGAAGCCAAACCCCTACTTCTAC
AGCTGTTGTCATCACTGAGTCAGCACTCACCTGGCCTGGGGGAATTAAAGGAATTCCCCGTAAGGGCTGG
ACCCAGCTCCTTTCTGGGCTTGGGTAGTCAAATGTCTGAGCTACCCCAGGTCCTCATGTCAGCAGAGTGG
CGCCTGCCTGTGCTGGCCCATGGAACGGAGACAGCATTGGGCTGACTGTGGGCATGAGGAGGGATAAGGC
TGATTTGGACCCCAGGCTTCTGCCCTGGACAGCACTTGTGTCTGCAGATTATTTAAGTGGCTTTTGATCT
GTAAATAAAATCAGTGCACTGTGCATCACACCCAGCCCCTTTCCCTGCTGTGTGGATTAGGTGTCAAGAC
ACCTAGTTCTTCCTGGGGCCACCCGGCTGGCCTCACTGCTTATATTAAGGCTCCTCCCAACTCTCATTTT
CCTTTGGAAAACAAGACTTTTTCCCCATGGTTACCGCTGAGATACTGGGGCTGTAGTAGTATAAAAGCT
CACAGTTCCTTCTGAGTGCTGAAAAGAGTGCATGAGTTGCTTCGAAATAAAAGGGTCAAGCATTCCTACC
TGAGACAGGTTAAAAAAAAAAA
>NP_001026847.1 ras-related protein Rab-24 [Homo sapiens]
MSGQRVDVKVVMLGKEYVGKTSLVERYVHDRFLVGPYQNTIGAAFVAKVMSVGDRTVTLGIWDTAGSERY
EAMSRIYYRGAKAAIVCYDLTDSSSFERAKFWVKELRSLEEGCQIYLCGTKSDLLEEDRRRRRVDFHDVQ
DYADNIKAQLFETSSKTGQSVDELFQKVAEDYVSVAAFQVMTEDKGVDLGQKPNPYFYSCCHH

>NM_130781.3 Homo sapiens RAB24, member RAS oncogene family (RAB24), transcript variant 2, mRNA
GACCTTGCGGCCCCGCCCCCTCGCCCTCTAGCCCCCTCCCGCGGGAGTCGCGGCGCTGCGGGTAGGAGCC
GGGTTGCGGGAGACCCCAGGTTCGGTTGGGATTCCCAGCCAGAACGGAGCTTAAGCCGGGCAGGCGAGCG
AATGACGGAGTAGCGAGCTGCACGGCGGCGTGCTGCGCTGTTGAGGACGCTGTCCCGCGCGCTCCCAGGC
CGCCCCGAGGCTTGGGGTCTTCGAAGGATAATCGGCGCCCGGGGCCGAACAGCGGGGGCACACGGGGCGC
TGCCGAAGTGCAAGGCCACGGCCAGAGCTCGAGCCCGACGCGCTGTCTGGAGTCGTAGGACCCTGACGTG
GCTGAAGCGGCCCCGGGAGCATGAGCGGGCAGCGCGTGGACGTCAAGGTGGTGATGCTGGGCAAGGAGTA
CGTGGGCAAGACTAGCCTGGTGGAGCGCTACGTGCACGACCGCTTTCTGGTGGGGCCTTATCAGAACACC
ATCGGGGCCGCCTTCGTGGCCAAGGTGATGTCGGTCGGAGACCGGACTGTGACATTAGGTATTTGGGACA
CAGCAGGCTCTGAGCGCTATGAGGCCATGAGTAGAATCTACTATCGGGGTGCCAAGGCTGCCATCGTCTG
CTATGACCTCACAGACAGCAGCAGCTTTGAGCGAGCAAAGTTCTGGGTGAAGGAACTGCGCAGCCTAGAG
GAGGGCTGCCAAATCTACTTATGTGGCACCAAGAGTGACCTGCTGGAAGAAGACCGGAGGCGTCGACGTG
TGGACTTCCACGACGTCCAGGACTATGCAGACAATATCAAAGCTCAGCTCTTTGAAACATCCAGCAAGAC
AGGCCAGAGTGTGGACGAGCTCTTCCAGAAAGTGGCAGAGGATTACGTCAGTGTGGCTGCCTTCCAGGTG
ATGACAGAGGACAAGGGCGTGGATCTGGGCCAGAAGCCAAACCCCTACTTCTACAGCTGTTGTCATCACT
GAGTCAGCACTCACCTGGCCTGGGGGAATTAAAGGAATTCCCCGTAAGGGCTGGACCCAGCTCCTTTCTG
GGCTTGGGTAGTCAAATGTCTGAGCTACCCCAGGTCCTCATGTCAGCAGAGTGGCGCCTGCCTGTGCTGG
CCCATGGAACGGAGACAGCATTGGGCTGACTGTGGGCATGAGGAGGGATAAGGCTGATTTGGACCCCAGG

```
CTTCTGCCCTGGACAGCACTTGTGTCTGCAGATTATTTAAGTGGCTTTTGATCTGTAAATAAAATCAGTG
CACTGTGCATCACACCCAGCCCCTTTCCCTGCTGTGTGGATTAGGTGTCAAGACACCTAGTTCTTCCTGG
GGCCACCCGGCTGGCCTCACTGCTTATATTAAGGCTCCTCCCAACTCTCATTTTCCTTTGGAAAACAAGA
CTTTTTTCCCCATGGTTACCGCTGAGATACTGGGGCTGTAGTAGTATAAAAGCTCACAGTTCCTTCTGAG
TGCTGAAAAGAGTGCATGAGTTGCTTCGAAATAAAAGGGTCAAGCATTCCTACCTGAGACAGGTTAAAAA
AAAAAA
>NP_570137.2 ras-related protein Rab-24 [Homo sapiens]
MSGQRVDVKVVMLGKEYVGKTSLVERYVHDRFLVGPYQNTIGAAFVAKVMSVGDRTVTLGIWDTAGSERY
EAMSRIYYRGAKAAIVCYDLTDSSSFERAKFWVKELRSLEEGCQIYLCGTKSDLLEEDRRRRRVDFHDVQ
DYADNIKAQLFETSSKTGQSVDELFQKVAEDYVSVAAFQVMTEDKGVDLGQKPNPYFYSCCHH
>NR_109789.1 Homo sapiens RAB24, member RAS oncogene family (RAB24), transcript variant 3, non-coding RNA
GACCTTGCGGCCCCGCCCCCTCGCCCTCTAGCCCCCTCCCGCGGGAGTCGCGGCGCTGCGGGTAGGAGCC
GGGTTGCGGGAGACCCCAGGTTCGGTTGGGATTCCCAGCCAGAACGGAGCTTAAGCCGGGCAGGCGAGCG
AATGACGGAGTAGCGAGCTGCACGGCGGCGTGCTGCGCTGTTGAGGACGCTGTCCCGCGCGCTCCCAGGC
CGCCCCGAGGCTTGGGGTCTTCGAAGGATAATCGGCGCCCGGGGCCGAACAGCGGGGGCACACGGGGCGC
TGCCGAAGTGCAAGGCCACGGCCAGAGCTCGAGCCCGACGCGCTGTCTGGAGTCGTAGGACCCTGACGTG
GCTGAAGCGGCCCCGGGAGCATGAGCGGGCAGCGCGTGGACGTCAAGGTGGTGATGCTGGGCAAGGAGTA
CGTGGGCAAGACTAGCCTGGTGGAGCGCTACGTGCACGACCGCTTTCTGGTGGGGCCTTATCAGAACACC
ATCGGGGCCGCCTTCGTGGCCAAGGTGATGTCGGTCGGAGACCGGACTGTGACATTAGGTATTTGGGACA
CAGCAGGCTCTGAGCGCTATGAGGCCATGAGTAGAATCTACTATCGGGGTGCCAAGGCTGCCATCGTCTG
CTATGACCTCACAGACAGCAGCAGCTTTGAGCGAGCAAAGTTCTGGGTGAAGGAACTGCGCAGCCTAGAG
GAGGGCTGCCAAATCTACTTATGTGGCACCAAGAGTGACCTGCTGGAAGAAGACCGGAGGCGTCGACGTG
TGGACTTCCACGACGTCCAGGACTATGCAGACAGTAGCTGCTCCTCAGCCCTTTGGGGGGTGGGGGTGTG
TGGCTGTCTGGGTGGATCAAAGAAAATAGGGACTGCCTTGGCTGCCAGGGCAAGGTGCTCTAGGAGGTCT
TCCTGGCCTCCTTGAACTGTGGGGTCCAGGAGACTCCCTGAACTGCTAGCCCTCCCTTTTGTCTGTTTAT
CTAATTCTCAGGTATGAGGCTTTAGTCACTTCTCTTTACAGATATCAAAGCTCAGCTCTTTGAAACATCC
AGCAAGACAGGCCAGAGTGTGGACGAGCTCTTCCAGAAAGTGGCAGAGGATTACGTCAGTGTGGCTGCCT
TCCAGGTGATGACAGAGGACAAGGGCGTGGATCTGGGCCAGAAGCCAAACCCCTACTTCTACAGCTGTTG
TCATCACTGAGTCAGCACTCACCTGGCCTGGGGGAATTAAAGGAATTCCCCGTAAGGGCTGGACCCAGCT
CCTTTCTGGGCTTGGGTAGTCAAATGTCTGAGCTACCCCAGGTCCTCATGTCAGCAGAGTGGCGCCTGCC
TGTGCTGGCCCATGGAACGGAGACAGCATTGGGCTGACTGTGGGCATGAGGAGGGATAAGGCTGATTTGG
ACCCCAGGCTTCTGCCCTGGACAGCACTTGTGTCTGCAGATTATTTAAGTGGCTTTTGATCTGTAAATAA
AATCAGTGCACTGTGCATCACACCCAGCCCCTTTCCCTGCTGTGTGGATTAGGTGTCAAGACACCTAGTT
CTTCCTGGGGCCACCCGGCTGGCCTCACTGCTTATATTAAGGCTCCTCCCAACTCTCATTTTCCTTTGGA
AAACAAGACTTTTTTCCCCATGGTTACCGCTGAGATACTGGGGCTGTAGTAGTATAAAAGCTCACAGTTC
CTTCTGAGTGCTGAAAAGAGTGCATGAGTTGCTTCGAAATAAAAGGGTCAAGCATTCCTACCTGAGACAG
GTTAAAAAAAAAAA
```

```
i1_HQ_samplec9816d|c43648/f2p0/1435     -------------------------------------------------gcggcgctgcg     11
NR_109789.1                             GACCTTGCGGCCCCGCCCCCTCGCCCTCTAGCCCCCTCCCGCGGGAGTCGCGGCGCTGCG     60
i0_HQ_samplec9816d|c43092/f2p19/798     ------------------------------------------------------------     0
NM_130781.3                             GACCTTGCGGCCCCGCCCCCTCGCCCTCTAGCCCCCTCCCGCGGGAGTCGCGGCGCTGCG     60
i1_HQ_samplec9816d|c63433/f11p0/1318    -----------------------------------gtcccgcgggagtcgcggcgctgcg     25
NM_001031677.3                          GACCTTGCGGCCCCGCCCCCTCGCCCTCTAGCCCCCTCCCGCGGGAGTCGCGGCGCTGCG     60
```

```
i1_HQ_samplec9816d|c43648/f2p0/1435     ggtaggagccgggttgcgggagaccccaggttcggttgggattcccagccaGAACGGAGC     71
NR_109789.1                             GGTAGGAGCCGGGTTGCGGGAGACCCCAGGTTCGGTTGGGATTCCCAGCCAGAACGGAGC     12
i0_HQ_samplec9816d|c43092/f2p19/798     ------------------------------------------------------------     0
NM_130781.3                             GGTAGGAGCCGGGTTGCGGGAGACCCCAGGTTCGGTTGGGATTCCCAGCCAGAACGGAGC     12
i1_HQ_samplec9816d|c63433/f11p0/1318    ggtaggagccgggttgcgggagaccccaggttcggttgggaTTCCCAGCCAGAACGGAGC     85
NM_001031677.3                          GGTAGGAGCCGGGTTGCGGGAGACCCCAGGTTCGGTTGGGATTCCCAGCCAGAACGGAGC     12
```

```
i1_HQ_samplec9816d|c43648/f2p0/1435     TTAAGCCGGGCAGGCGAGCGAATGACGGAGTAGCGAGCTGCACGGCGGCGTGCTGCGCTG     13
NR_109789.1                             TTAAGCCGGGCAGGCGAGCGAATGACGGAGTAGCGAGCTGCACGGCGGCGTGCTGCGCTG     18
i0_HQ_samplec9816d|c43092/f2p19/798     ------------------------------------------------------------     0
NM_130781.3                             TTAAGCCGGGCAGGCGAGCGAATGACGGAGTAGCGAGCTGCACGGCGGCGTGCTGCGCTG     18
i1_HQ_samplec9816d|c63433/f11p0/1318    TTAAGCCGGGCAGGCGAGCGAATGACGGAGTAGCGAGCTGCACGGCGGCGTGCTGCGCTG     14
NM_001031677.3                          TTAAGCCGGGCAGGCGAGCGAATGACGGAGTAGCGAGCTGCACGGCGGCGTGCTGCGCTG     18
```

```
i1_HQ_samplec9816d|c43648/f2p0/1435     TTGAGGACGCTGTCCCGCGCGCTCCCAGGCCGCCCCGAGGCTTGGGGTCTTCGAAGGATA     19
NR_109789.1                             TTGAGGACGCTGTCCCGCGCGCTCCCAGGCCGCCCCGAGGCTTGGGGTCTTCGAAGGATA     24
i0_HQ_samplec9816d|c43092/f2p19/798     ------------------------------------------------------------     0
NM_130781.3                             TTGAGGACGCTGTCCCGCGCGCTCCCAGGCCGCCCCGAGGCTTGGGGTCTTCGAAGGATA     24
i1_HQ_samplec9816d|c63433/f11p0/1318    TTGAGGACGCTGTCCCGCGCGCTCCCAGGCCGCCCCGAGGCTTGGGGTCTTCGAAGGATA     20
NM_001031677.3                          TTGAGGACGCTGTCCCGCGCGCTCCCAGGCCGCCCCGAGGCTTGGGGTCTTCGAAGGATA     24
```

```
i1_HQ_samplec9816d|c43648/f2p0/1435     ATCGGCGCCCGGGGCCGAACAGCGGGGGCACACGGGGCGCTGCCGAAGTGCAAGGCCACG     25
NR_109789.1                             ATCGGCGCCCGGGGCCGAACAGCGGGGGCACACGGGGCGCTGCCGAAGTGCAAGGCCACG     30
```

```
i0_HQ_samplec9816d|c43092/f2p19/798     ------------------------------------------------------------ 0
NM_130781.3                             ATCGGCGCCCGGGGCCGAACAGCGGGGGCACACGGGGCGCTGCCGAAGTGCAAGGCCACG 30
i1_HQ_samplec9816d|c63433/f11p0/1318    ATCGGCGCCCGGGGCCGAACAGCGGGGGCACACGGGGCGCTGCCGAAGTGCAAGGCCACG 26
NM_001031677.3                          ATCGGCGCCCGGGGCCGAACAGCGGGGGCACACGGGGCGCTGCCGAAGTGCAAGGCCACG 30

i1_HQ_samplec9816d|c43648/f2p0/1435     GCCAGAGCTCGAGCCCGACGCGCTGTCTGGAGTCGTAG---------------------- 28
NR_109789.1                             GCCAGAGCTCGAGCCCGACGCGCTGTCTGGAGTCGTAG---------------------- 33
i0_HQ_samplec9816d|c43092/f2p19/798     ------------------------------------------------------------ 0
NM_130781.3                             GCCAGAGCTCGAGCCCGACGCGCTGTCTGGAGTCGTAG---------------------- 33
i1_HQ_samplec9816d|c63433/f11p0/1318    GCCAGAGCTCGAGCCCGACGCGCTGTCTGGAGTCGTAGGTTGGCGCCGTTTGGGGTCGGG 32
NM_001031677.3                          GCCAGAGCTCGAGCCCGACGCGCTGTCTGGAGTCGTAGGTTGGCGCCGTTTGGGGTCGGG 36

i1_HQ_samplec9816d|c43648/f2p0/1435     ------------------------------------------------------------ 28
NR_109789.1                             ------------------------------------------------------------ 33
i0_HQ_samplec9816d|c43092/f2p19/798     ------------------------------------------------------------ 0
NM_130781.3                             ------------------------------------------------------------ 33
i1_HQ_samplec9816d|c63433/f11p0/1318    GTCTGAGGCTTGGGCGCTGCCTGGGCCGAGCGGAGATCGGGGTTTGCCTCCCGTCCCCGC 38
NM_001031677.3                          GTCTGAGGCTTGGGCGCTGCCTGGGCCGAGCGGAGATCGGGGTTTGCCTCCCGTCCCCGC 42

i1_HQ_samplec9816d|c43648/f2p0/1435     ----GACCCTGACGTGGCTGAAGCGGCCCCGGGAGCATGAGCGGGCAGCGCGTGGACGTC 34
NR_109789.1                             ----GACCCTGACGTGGCTGAAGCGGCCCCGGGAGCATGAGCGGGCAGCGCGTGGACGTC 39
i0_HQ_samplec9816d|c43092/f2p19/798     ------------------------------------------------------------ 0
NM_130781.3                             ----GACCCTGACGTGGCTGAAGCGGCCCCGGGAGCATGAGCGGGCAGCGCGTGGACGTC 39
i1_HQ_samplec9816d|c63433/f11p0/1318    TCAGGACCCTGACGTGGCTGAAGCGGCCCCGGGAGCATGAGCGGGCAGCGCGTGGACGTC 44
NM_001031677.3                          TCAGGACCCTGACGTGGCTGAAGCGGCCCCGGGAGCATGAGCGGGCAGCGCGTGGACGTC 48

i1_HQ_samplec9816d|c43648/f2p0/1435     AAGGTGGTGATGCTGGGCAAGGAGTACGTGGGCAAGACTAGCCTGGTGGAGCGCTACGTG 40
NR_109789.1                             AAGGTGGTGATGCTGGGCAAGGAGTACGTGGGCAAGACTAGCCTGGTGGAGCGCTACGTG 45
i0_HQ_samplec9816d|c43092/f2p19/798     ------------------------------------------------------------ 0
NM_130781.3                             AAGGTGGTGATGCTGGGCAAGGAGTACGTGGGCAAGACTAGCCTGGTGGAGCGCTACGTG 45
i1_HQ_samplec9816d|c63433/f11p0/1318    AAGGTGGTGATGCTGGGCAAGGAGTACGTGGGCAAGACTAGCCTGGTGGAGCGCTACGTG 50
NM_001031677.3                          AAGGTGGTGATGCTGGGCAAGGAGTACGTGGGCAAGACTAGCCTGGTGGAGCGCTACGTG 54

i1_HQ_samplec9816d|c43648/f2p0/1435     CACGACCGCTTTCTGGTGGGGCCTTATCAGAACACCATCGGGGCCGCCTTCGTGGCCAAG 46
NR_109789.1                             CACGACCGCTTTCTGGTGGGGCCTTATCAGAACACCATCGGGGCCGCCTTCGTGGCCAAG 51
i0_HQ_samplec9816d|c43092/f2p19/798     ---------------ggagtcgcggcgctgcggACCATCGGGGCCGCCTTCGTGGCCAAG 45
NM_130781.3                             CACGACCGCTTTCTGGTGGGGCCTTATCAGAACACCATCGGGGCCGCCTTCGTGGCCAAG 51
i1_HQ_samplec9816d|c63433/f11p0/1318    CACGACCGCTTTCTGGTGGGGCCTTATCAGAACACCATCGGGGCCGCCTTCGTGGCCAAG 56
NM_001031677.3                          CACGACCGCTTTCTGGTGGGGCCTTATCAGAACACCATCGGGGCCGCCTTCGTGGCCAAG 60
                                                       *  *   *    * *   ***************************

i1_HQ_samplec9816d|c43648/f2p0/1435     GTGATGTCGGTCGGAGACCGGACTGTGACATTAGGTATTTGGGACACAGCAGGCTCTGAG 52
NR_109789.1                             GTGATGTCGGTCGGAGACCGGACTGTGACATTAGGTATTTGGGACACAGCAGGCTCTGAG 57
i0_HQ_samplec9816d|c43092/f2p19/798     GTGATGTCGGTCGGAGACCGGACTGTGACATTAGGTATTTGGGACACAGCAGGCTCTGAG 10
NM_130781.3                             GTGATGTCGGTCGGAGACCGGACTGTGACATTAGGTATTTGGGACACAGCAGGCTCTGAG 57
i1_HQ_samplec9816d|c63433/f11p0/1318    GTGATGTCGGTCGGAGACCGGACTGTGACATTAGGTATTTGGGACACAGCAGGCTCTGAG 62
NM_001031677.3                          GTGATGTCGGTCGGAGACCGGACTGTGACATTAGGTATTTGGGACACAGCAGGCTCTGAG 66
                                        ************************************************************

i1_HQ_samplec9816d|c43648/f2p0/1435     CGCTATGAGGCCATGAGTAGAATCTACTATCGGGGTGCCAAGGCTGCCATCGTCTGCTAT 58
NR_109789.1                             CGCTATGAGGCCATGAGTAGAATCTACTATCGGGGTGCCAAGGCTGCCATCGTCTGCTAT 63
i0_HQ_samplec9816d|c43092/f2p19/798     CGCTATGAGGCCATGAGTAGAATCTACTATCGGGGTGCCAAGGCTGCCATCGTCTGCTAT 16
NM_130781.3                             CGCTATGAGGCCATGAGTAGAATCTACTATCGGGGTGCCAAGGCTGCCATCGTCTGCTAT 63
i1_HQ_samplec9816d|c63433/f11p0/1318    CGCTATGAGGCCATGAGTAGAATCTACTATCGGGGTGCCAAGGCTGCCATCGTCTGCTAT 68
NM_001031677.3                          CGCTATGAGGCCATGAGTAGAATCTACTATCGGGGTGCCAAGGCTGCCATCGTCTGCTAT 72
                                        ************************************************************

i1_HQ_samplec9816d|c43648/f2p0/1435     GACCTCACAGACAGCAGCAGCTTTGAGCGAGCAAAGTTCTGGGTGAAGGAACTGCGCAGC 64
NR_109789.1                             GACCTCACAGACAGCAGCAGCTTTGAGCGAGCAAAGTTCTGGGTGAAGGAACTGCGCAGC 69
i0_HQ_samplec9816d|c43092/f2p19/798     GACCTCACAGACAGCAGCAGCTTTGAGCGAGCAAAGTTCTGGGTGAAGGAACTGCGCAGC 22
NM_130781.3                             GACCTCACAGACAGCAGCAGCTTTGAGCGAGCAAAGTTCTGGGTGAAGGAACTGCGCAGC 69
i1_HQ_samplec9816d|c63433/f11p0/1318    GACCTCACAGACAGCAGCAGCTTTGAGCGAGCAAAGTTCTGGGTGAAGGAACTGCGCAGC 74
NM_001031677.3                          GACCTCACAGACAGCAGCAGCTTTGAGCGAGCAAAGTTCTGGGTGAAGGAACTGCGCAGC 78
                                        ************************************************************

i1_HQ_samplec9816d|c43648/f2p0/1435     CTAGAGGAGGGCTGCCAAATCTACTTATGTGGCACCAAGAGTGACCTGCTGGAAGAAGAC 70
NR_109789.1                             CTAGAGGAGGGCTGCCAAATCTACTTATGTGGCACCAAGAGTGACCTGCTGGAAGAAGAC 75
i0_HQ_samplec9816d|c43092/f2p19/798     CTAGAGGAGGGCTGCCAAATCTACTTATGTGGCACCAAGAGTGACCTGCTGGAAGAAGAC 28
NM_130781.3                             CTAGAGGAGGGCTGCCAAATCTACTTATGTGGCACCAAGAGTGACCTGCTGGAAGAAGAC 75
i1_HQ_samplec9816d|c63433/f11p0/1318    CTAGAGGAGGGCTGCCAAATCTACTTATGTGGCACCAAGAGTGACCTGCTGGAAGAAGAC 80
NM_001031677.3                          CTAGAGGAGGGCTGCCAAATCTACTTATGTGGCACCAAGAGTGACCTGCTGGAAGAAGAC 84
                                        ************************************************************

i1_HQ_samplec9816d|c43648/f2p0/1435     CGGAGGCGTCGACGTGTGGACTTCCACGACGTCCAGGACTATGCAGACAGTAGCTGCTCC 76
NR_109789.1                             CGGAGGCGTCGACGTGTGGACTTCCACGACGTCCAGGACTATGCAGACAGTAGCTGCTCC 81
i0_HQ_samplec9816d|c43092/f2p19/798     CGGAGGCGTCGACGTGTGGACTTCCACGACGTCCAGGACTATGCAGACA----------- 33
NM_130781.3                             CGGAGGCGTCGACGTGTGGACTTCCACGACGTCCAGGACTATGCAGACA----------- 80
```

```
i1_HQ_samplec9816d|c63433/f11p0/1318      CGGAGGCGTCGACGTGTGGACTTCCACGACGTCCAGGACTATGCAGACA-----------     85
NM_001031677.3                            CGGAGGCGTCGACGTGTGGACTTCCACGACGTCCAGGACTATGCAGACA-----------     88
                                          *************************************************

i1_HQ_samplec9816d|c43648/f2p0/1435       TCAGCCCTTTGGGGGGTGGGGGTGTGTGGCTGTCTGGGTGGATCAAAGAAAATAGGGACT     82
NR_109789.1                               TCAGCCCTTTGGGGGGTGGGGGTGTGTGGCTGTCTGGGTGGATCAAAGAAAATAGGGACT     87
i0_HQ_samplec9816d|c43092/f2p19/798       ------------------------------------------------------------     33
NM_130781.3                               ------------------------------------------------------------     80
i1_HQ_samplec9816d|c63433/f11p0/1318      ------------------------------------------------------------     85
NM_001031677.3                            ------------------------------------------------------------     88

i1_HQ_samplec9816d|c43648/f2p0/1435       GCCTTGGCTGCCAGGGCAAGGTGCTCTAGGAGGTCTTCCTGGCCTCCTTGAACTGTGGGG     88
NR_109789.1                               GCCTTGGCTGCCAGGGCAAGGTGCTCTAGGAGGTCTTCCTGGCCTCCTTGAACTGTGGGG     93
i0_HQ_samplec9816d|c43092/f2p19/798       ------------------------------------------------------------     33
NM_130781.3                               ------------------------------------------------------------     80
i1_HQ_samplec9816d|c63433/f11p0/1318      ------------------------------------------------------------     85
NM_001031677.3                            ------------------------------------------------------------     88

i1_HQ_samplec9816d|c43648/f2p0/1435       TCCAGGAGACTCCCTGAACTGCTAGCCCTCCCTTTTGTCTGTTTATCTAATTCTCAGGTA     94
NR_109789.1                               TCCAGGAGACTCCCTGAACTGCTAGCCCTCCCTTTTGTCTGTTTATCTAATTCTCAGGTA     99
i0_HQ_samplec9816d|c43092/f2p19/798       ------------------------------------------------------------     33
NM_130781.3                               ------------------------------------------------------------     80
i1_HQ_samplec9816d|c63433/f11p0/1318      ------------------------------------------------------------     85
NM_001031677.3                            ------------------------------------------------------------     88

i1_HQ_samplec9816d|c43648/f2p0/1435       TGAGGCTTTAGTCACTTCTCTTTACAGATATCAAAGCTCAGCTCTTTGAAACATCCAGCA     10
NR_109789.1                               TGAGGCTTTAGTCACTTCTCTTTACAGATATCAAAGCTCAGCTCTTTGAAACATCCAGCA     10
i0_HQ_samplec9816d|c43092/f2p19/798       ---------------------------ATATCAAAGCTCAGCTCTTTGAAACATCCAGCA     36
NM_130781.3                               ---------------------------ATATCAAAGCTCAGCTCTTTGAAACATCCAGCA     83
i1_HQ_samplec9816d|c63433/f11p0/1318      ---------------------------ATATCAAAGCTCAGCTCTTTGAAACATCCAGCA     88
NM_001031677.3                            ---------------------------ATATCAAAGCTCAGCTCTTTGAAACATCCAGCA     92
                                                                     *********************************

i1_HQ_samplec9816d|c43648/f2p0/1435       AGACAGGCCAGAGTGTGGACGAGCTCTTCCAGAAAGTGGCAGAGGATTACGTCAGTGTGG     10
NR_109789.1                               AGACAGGCCAGAGTGTGGACGAGCTCTTCCAGAAAGTGGCAGAGGATTACGTCAGTGTGG     11
i0_HQ_samplec9816d|c43092/f2p19/798       AGACAGGCCAGAGTGTGGACGAGCTCTTCCAGAAAGTGGCAGAGGATTACGTCAGTGTGG     42
NM_130781.3                               AGACAGGCCAGAGTGTGGACGAGCTCTTCCAGAAAGTGGCAGAGGATTACGTCAGTGTGG     89
i1_HQ_samplec9816d|c63433/f11p0/1318      AGACAGGCCAGAGTGTGGACGAGCTCTTCCAGAAAGTGGCAGAGGATTACGTCAGTGTGG     94
NM_001031677.3                            AGACAGGCCAGAGTGTGGACGAGCTCTTCCAGAAAGTGGCAGAGGATTACGTCAGTGTGG     98
                                          ************************************************************

i1_HQ_samplec9816d|c43648/f2p0/1435       CTGCCTTCCAGGTGATGACAGAGGACAAGGGCGTGGATCTGGGCCAGAAGCCAAACCCCT     11
NR_109789.1                               CTGCCTTCCAGGTGATGACAGAGGACAAGGGCGTGGATCTGGGCCAGAAGCCAAACCCCT     11
i0_HQ_samplec9816d|c43092/f2p19/798       CTGCCTTCCAGGTGATGACAGAGGACAAGGGCGTGGATCTGGGCCAGAAGCCAAACCCCT     48
NM_130781.3                               CTGCCTTCCAGGTGATGACAGAGGACAAGGGCGTGGATCTGGGCCAGAAGCCAAACCCCT     95
i1_HQ_samplec9816d|c63433/f11p0/1318      CTGCCTTCCAGGTGATGACAGAGGACAAGGGCGTGGATCTGGGCCAGAAGCCAAACCCCT     10
NM_001031677.3                            CTGCCTTCCAGGTGATGACAGAGGACAAGGGCGTGGATCTGGGCCAGAAGCCAAACCCCT     10
                                          ************************************************************

i1_HQ_samplec9816d|c43648/f2p0/1435       ACTTCTACAGCTGTTGTCATCACTGAGTCAGCACTCACCTGGCCTGGGGGAATTAAAGGA     11
NR_109789.1                               ACTTCTACAGCTGTTGTCATCACTGAGTCAGCACTCACCTGGCCTGGGGGAATTAAAGGA     12
i0_HQ_samplec9816d|c43092/f2p19/798       ACTTCTACAGCTGTTGTCATCACTGAGTCAGCACTCACCTGGCCTGGGGGAATTAAAGGA     54
NM_130781.3                               ACTTCTACAGCTGTTGTCATCACTGAGTCAGCACTCACCTGGCCTGGGGGAATTAAAGGA     10
i1_HQ_samplec9816d|c63433/f11p0/1318      ACTTCTACAGCTGTTGTCATCACTGAGTCAGCACTCACCTGGCCTGGGGGAATTAAAGGA     10
NM_001031677.3                            ACTTCTACAGCTGTTGTCATCACTGAGTCAGCACTCACCTGGCCTGGGGGAATTAAAGGA     11
                                          ************************************************************

i1_HQ_samplec9816d|c43648/f2p0/1435       ATTCCCCGTAAGGGCTGGACCCAGCTCCTTTCTGGGCTTGGGTAGTCAAATGTCTGAGCT     12
NR_109789.1                               ATTCCCCGTAAGGGCTGGACCCAGCTCCTTTCTGGGCTTGGGTAGTCAAATGTCTGAGCT     12
i0_HQ_samplec9816d|c43092/f2p19/798       ATTCCCCGTAAGGGCTGGACCCAGCTCCTTTCTGGGCTTGGGTAGTCAAATGTCTGAGCT     60
NM_130781.3                               ATTCCCCGTAAGGGCTGGACCCAGCTCCTTTCTGGGCTTGGGTAGTCAAATGTCTGAGCT     10
i1_HQ_samplec9816d|c63433/f11p0/1318      ATTCCCCGTAAGGGCTGGACCCAGCTCCTTTCTGGGCTTGGGTAGTCAAATGTCTGAGCT     11
NM_001031677.3                            ATTCCCCGTAAGGGCTGGACCCAGCTCCTTTCTGGGCTTGGGTAGTCAAATGTCTGAGCT     11
                                          ************************************************************

i1_HQ_samplec9816d|c43648/f2p0/1435       ACCCCAGGTCCTCATGTCAGCAGAGTGGCGCCTGCCTGTGCTGGCCCATGGAACGGAGAC     13
NR_109789.1                               ACCCCAGGTCCTCATGTCAGCAGAGTGGCGCCTGCCTGTGCTGGCCCATGGAACGGAGAC     13
i0_HQ_samplec9816d|c43092/f2p19/798       ACCCCAGGTCCTCATGTCAGCAGAGTGGCGCCTGCCTGTGCTGGCCCATGGAACGGAGAC     66
NM_130781.3                               ACCCCAGGTCCTCATGTCAGCAGAGTGGCGCCTGCCTGTGCTGGCCCATGGAACGGAGAC     11
i1_HQ_samplec9816d|c63433/f11p0/1318      ACCCCAGGTCCTCATGTCAGCAGAGTGGCGCCTGCCTGTGCTGGCCCATGGAACGGAGAC     11
NM_001031677.3                            ACCCCAGGTCCTCATGTCAGCAGAGTGGCGCCTGCCTGTGCTGGCCCATGGAACGGAGAC     12
                                          ************************************************************

i1_HQ_samplec9816d|c43648/f2p0/1435       AGCATTGGGCTGACTGTGGGCATGAGGAGGGATAAGGCTGATTTGGACCCCAGGCTTCTG     13
NR_109789.1                               AGCATTGGGCTGACTGTGGGCATGAGGAGGGATAAGGCTGATTTGGACCCCAGGCTTCTG     14
i0_HQ_samplec9816d|c43092/f2p19/798       AGCATTGGGCTGACTGTGGGCATGAGGAGGGATAAGGCTGATTTGGACCCCAGGCTTCTG     72
NM_130781.3                               AGCATTGGGCTGACTGTGGGCATGAGGAGGGATAAGGCTGATTTGGACCCCAGGCTTCTG     11
i1_HQ_samplec9816d|c63433/f11p0/1318      AGCATTGGGCTGACTGTGGGCATGAGGAGGGATAAGGCTGATTTGGACCCCAGGCTTCTG     12
NM_001031677.3                            AGCATTGGGCTGACTGTGGGCATGAGGAGGGATAAGGCTGATTTGGACCCCAGGCTTCTG     12
```

```
                                                                   ************************************************************

i1_HQ_samplec9816d|c43648/f2p0/1435        CCCTGGACAGCACTTGTGTCTGCAGATTATTTAAgtggcttttgatctgtaaataaaatc        14[illegible]
NR_109789.1                                CCCTGGACAGCACTTGTGTCTGCAGATTATTTAAGTGGCTTTTGATCTGTAAATAAAATC        14[illegible]
i0_HQ_samplec9816d|c43092/f2p19/798        CCCTGGACAGCACTTGTGTCTGCAGATTATTTAAGTGGCTTTTGATCTGTAAATAAAATC        78[illegible]
NM_130781.3                                CCCTGGACAGCACTTGTGTCTGCAGATTATTTAAGTGGCTTTTGATCTGTAAATAAAATC        12[illegible]
i1_HQ_samplec9816d|c63433/f11p0/1318       CCCTGGACAGCACTTGTGTCTGCAGATTATTTAAGTGGCTTTTGatctgtaaataaaatc        13[illegible]
NM_001031677.3                             CCCTGGACAGCACTTGTGTCTGCAGATTATTTAAGTGGCTTTTGATCTGTAAATAAAATC        13[illegible]
                                           ************************************************************

i1_HQ_samplec9816d|c43648/f2p0/1435        agtgcactgt--------------------------------------------------        14[illegible]
NR_109789.1                                AGTGCACTGTGCATCACACCCAGCCCCTTTCCCTGCTGTGTGGATTAGGTGTCAAGACAC        15[illegible]
i0_HQ_samplec9816d|c43092/f2p19/798        AGTGCACTGTG-------------------------------------------------        79[illegible]
NM_130781.3                                AGTGCACTGTGCATCACACCCAGCCCCTTTCCCTGCTGTGTGGATTAGGTGTCAAGACAC        13[illegible]
i1_HQ_samplec9816d|c63433/f11p0/1318       agtgcactgtg-------------------------------------------------        13[illegible]
NM_001031677.3                             AGTGCACTGTGCATCACACCCAGCCCCTTTCCCTGCTGTGTGGATTAGGTGTCAAGACAC        14[illegible]
                                           **********

i1_HQ_samplec9816d|c43648/f2p0/1435        ------------------------------------------------------------        14[illegible]
NR_109789.1                                CTAGTTCTTCCTGGGGCCACCCGGCTGGCCTCACTGCTTATATTAAGGCTCCTCCCAACT        15[illegible]
i0_HQ_samplec9816d|c43092/f2p19/798        ------------------------------------------------------------        79[illegible]
NM_130781.3                                CTAGTTCTTCCTGGGGCCACCCGGCTGGCCTCACTGCTTATATTAAGGCTCCTCCCAACT        13[illegible]
i1_HQ_samplec9816d|c63433/f11p0/1318       ------------------------------------------------------------        13[illegible]
NM_001031677.3                             CTAGTTCTTCCTGGGGCCACCCGGCTGGCCTCACTGCTTATATTAAGGCTCCTCCCAACT        14[illegible]

i1_HQ_samplec9816d|c43648/f2p0/1435        ------------------------------------------------------------        14[illegible]
NR_109789.1                                CTCATTTTCCTTTGGAAAACAAGACTTTTTTCCCCATGGTTACCGCTGAGATACTGGGGC        16[illegible]
i0_HQ_samplec9816d|c43092/f2p19/798        ------------------------------------------------------------        79[illegible]
NM_130781.3                                CTCATTTTCCTTTGGAAAACAAGACTTTTTTCCCCATGGTTACCGCTGAGATACTGGGGC        14[illegible]
i1_HQ_samplec9816d|c63433/f11p0/1318       ------------------------------------------------------------        13[illegible]
NM_001031677.3                             CTCATTTTCCTTTGGAAAACAAGACTTTTTTCCCCATGGTTACCGCTGAGATACTGGGGC        15[illegible]

i1_HQ_samplec9816d|c43648/f2p0/1435        ------------------------------------------------------------        14[illegible]
NR_109789.1                                TGTAGTAGTATAAAAGCTCACAGTTCCTTCTGAGTGCTGAAAAGAGTGCATGAGTTGCTT        17[illegible]
i0_HQ_samplec9816d|c43092/f2p19/798        ------------------------------------------------------------        79[illegible]
NM_130781.3                                TGTAGTAGTATAAAAGCTCACAGTTCCTTCTGAGTGCTGAAAAGAGTGCATGAGTTGCTT        14[illegible]
i1_HQ_samplec9816d|c63433/f11p0/1318       ------------------------------------------------------------        13[illegible]
NM_001031677.3                             TGTAGTAGTATAAAAGCTCACAGTTCCTTCTGAGTGCTGAAAAGAGTGCATGAGTTGCTT        15[illegible]

i1_HQ_samplec9816d|c43648/f2p0/1435        --------------------------------------------------        1435
NR_109789.1                                CGAAATAAAAGGGTCAAGCATTCCTACCTGAGACAGGTTAAAAAAAAAAA        1764
i0_HQ_samplec9816d|c43092/f2p19/798        --------------------------------------------------        798
NM_130781.3                                CGAAATAAAAGGGTCAAGCATTCCTACCTGAGACAGGTTAAAAAAAAAAA        1546
i1_HQ_samplec9816d|c63433/f11p0/1318       --------------------------------------------------        1318
NM_001031677.3                             CGAAATAAAAGGGTCAAGCATTCCTACCTGAGACAGGTTAAAAAAAAAAA        1632
```

```
VIRT2751            MSGQRVDVKVVMLGKEYVGKTSLVERYVHDRFLVGPYQNTIGAAFVAKVMSVGDRTVTLG  60
VIRT1094            MSGQRVDVKVVMLGKEYVGKTSLVERYVHDRFLVGPYQNTIGAAFVAKVMSVGDRTVTLG  60
VIRT5954            -------------------------------------------------MSVGDRTVTLG  11
NP_001026847.1      MSGQRVDVKVVMLGKEYVGKTSLVERYVHDRFLVGPYQNTIGAAFVAKVMSVGDRTVTLG  60
NP_570137.2         MSGQRVDVKVVMLGKEYVGKTSLVERYVHDRFLVGPYQNTIGAAFVAKVMSVGDRTVTLG  60
                                                                     ***********

VIRT2751            IWDTAGSERYEAMSRIYYRGAKAAIVCYDLTDSSSFERAKFWVKELRSLEEGCQIYLCGT  120
VIRT1094            IWDTAGSERYEAMSRIYYRGAKAAIVCYDLTDSSSFERAKFWVKELRSLEEGCQIYLCGT  120
VIRT5954            IWDTAGSERYEAMSRIYYRGAKAAIVCYDLTDSSSFERAKFWVKELRSLEEGCQIYLCGT  71
NP_001026847.1      IWDTAGSERYEAMSRIYYRGAKAAIVCYDLTDSSSFERAKFWVKELRSLEEGCQIYLCGT  120
NP_570137.2         IWDTAGSERYEAMSRIYYRGAKAAIVCYDLTDSSSFERAKFWVKELRSLEEGCQIYLCGT  120
                    ************************************************************

VIRT2751            KSDLLEEDRRRRRVDFHDVQDYADSSCSSALWGVGVCGCLGGS--KKIGTALAARA----  174
VIRT1094            KSDLLEEDRRRRRVDFHDVQDYADNIKAQLFETSSKTGQSVDELFQKVAEDYVSVAAFQV  180
VIRT5954            KSDLLEEDRRRRRVDFHDVQDYADNIKAQLFETSSKTGQSVDELFQKVAEDYVSVAAFQV  131
NP_001026847.1      KSDLLEEDRRRRRVDFHDVQDYADNIKAQLFETSSKTGQSVDELFQKVAEDYVSVAAFQV  180
NP_570137.2         KSDLLEEDRRRRRVDFHDVQDYADNIKAQLFETSSKTGQSVDELFQKVAEDYVSVAAFQV  180
                    ************************.  :. :    .   *    ..  :*:.   .: *

VIRT2751            ----RCSRRSSWP-P--------         184
VIRT1094            MTEDKGVDLGQKPNPYFYSCCHH         203
VIRT5954            MTEDKGVDLGQKPNPYFYSCCHH         154
NP_001026847.1      MTEDKGVDLGQKPNPYFYSCCHH         203
NP_570137.2         MTEDKGVDLGQKPNPYFYSCCHH         203
                        :     .. * *
```

**ARPC1A**

| Sequence | Header | AA Seq | |
|---|---|---|---|

| | | | |
|---|---|---|---|
| **Present** | i1_HQ_samplec9816d\|c18442/f2p0/1395 | >VIRT50347 (3-5’) | VIRT61659 (5-3’) |
| **Absent** | i1_HQ_samplec9816d\|c21346/f2p0/1056 | VIRT64494 (3-5’) | VIRT65386 (5-3’) |
| **NCBI** | NM_001190996 | NP_001177925.1 | |
| | NM_006409 | NP_006400.2 | |

>i1_HQ_samplec9816d|c18442/f2p0/1395
isoform=c18442;full_length_coverage=2;non_full_length_coverage=0;isoform_length=1395

aacagggatcgtactcagattgccctcagtcccaataatcacgaagtgcacatctataagaagaaCGGGAGCCAGTGGGTGAAAGCTCATGA
ACTCAAGGAGCACAACGGACACATCACAGGTATTGACTGGGCTCCCAAGAGCGACCGCATTGTCACTTGTGGGGCAGA
CCGCAATGCCTATGTCTGGAGTCAGAAAGATGGTGTTTGGAAGCCAACCCTGGTGATCCTGAGAATTAATCGCGCAGCT
ACTTTTGTGAAGTGGTCCCCCCTAGAGAACAAATTTGCTGTGGGAAGTGGAGCACGACTCATTTCTGTTTGTTACTTTGA
GTCTGAAAATGACTGGTGGGTGAGCAAGCACATTAAAAAGCCGATTCGCTCCACAGTCCTCAGCTTGGATTGGCATCCC
AACAACGTTTTGCTGGCAGCAGGATCATGTGACTTCAAATGCAGAGTGTTTTCTGCCTACATTAAAGAAGTGGATGAAA
AGCCAGCCAGCACGCCCTGGGGCAGCAAGATGCCTTTTGGGCAGCTGATGTCAGAGTTTGGTGGCAGTGGCACTGGTG
GCTGGGTCCACGGGGTAAGCTTCTCTGCCAGTGGGAGCCGCCTGGCCTGGGTCAGCCACGACAGCACCGTGTCTGTTG
CTGATGCCTCAAAAAGTGTGCAGGTCTCGACTCTGAAGACAGAGTTCCTGCCGCTCCTAAGTGTGTCATTTGTCTCAGA
GAACAGCGTCGTGGCTGCTGGCCATGACTGCTGCCCAATGCTCTTTAACTACGATGACCGCGGCTGCCTGACCTTCGTC
TCCAAGTTAGATATTCCAAAACAGAGCATCCAACGCAACATGTCTGCCATGGAACGCTTCCGCAACATGGACAAGAGAG
CCACAACTGAGGACCGCAACACGGCCTTGGAGACGCTGCACCAGAATAGCATCACTCAAGTCTCTATTTATGAGGTGGA
CAAGCAAGATTGTCGCAAATTTTGCACTACTGGCATCGATGGAGCCATGACAATTTGGGATTTCAAGACCCTCGAGTCT
TCCATCCAGGGCCTCCGGATAATGTGAAGCTGAGTGAGCCTCCGCCATCCAGCATGACAAACTGTGGCCGACCGCAGCT
GTGCCGTGGCACGATGGCGAGGAAGCCAGCCCCAAGGAAACACTGAAAACACATATCACGCCAATGCCGTGTGGTTTT
GTTTGAATATAAAATTGGTGAAAGTGTTGGTTTTTTTAAGGCAGTAATTTTTTTGTTTGTTTTTTTGCGATTTCATTCCATT
CTTGACCAAAGCTTCTCTTTAAGTAGTTTATTATGGAAAATTGTCACACTAACTTAAAAGACAGGGTGAGGGAGATATG
TAAATtgtccactagaaaattaaataaaagaactgaatg

>VIRT50347 (3-5’)
MAEAHSASHYPEALDGRLEGLEIPNCHGSIDASSAKFATILLVHLINRDLSDAILVQRLQ
GRVAVLSCGSLVHVAEAFHGRHVALDALFWNI
>VIRT61659 (5-3’)
MPFGQLMSEFGGSGTGGWVHGVSFSASGSRLAWVSHDSTVSVADASKSVQVSTLKTEFLP
LLSVSFVSENSVVAAGHDCCPMLFNYDDRGCLTFVSKLDIPKQSIQRNMSAMERFRNMDK
RATTEDRNTALETLHQNSITQVSIYEVDKQDCRKFCTTGIDGAMTIWDFKTLESSIQGLR
IM
>i1_HQ_samplec9816d|c21346/f2p0/1056
isoform=c21346;full_length_coverage=2;non_full_length_coverage=0;isoform_length=1056

actggtgggtgagcaagcacattaaaaagccgattcgctccacagtcctcagcTTGGATTGGCATCCCAACAACGTTTTGCTGGCAGCAGG
ATCATGTGACTTCAAATGCAGAGTGTTTTCTGCCTACATTAAAGAAGTGGATGAAAAGCCAGCCAGCACGCCCTGGGGC
AGCAAGATGCCTTTTGGGCAGCTGATGTCAGAGTTTGGTGGCAGTGGCACTGGTGGCTGGGTCCACGGGGTAAGCTTC
TCTGCCAGTGGGAGCCGCCTGGCCTGGGTCAGCCACGACAGCACCGTGTCTGTTGCTGATGCCTCAAAAAGTGTGCAG
GTCTCGACTCTGAAGACAGAGTTCCTGCCGCTCCTAAGTGTGTCATTTGTCTCAGAGAACAGCGTCGTGGCTGCTGGCC
ATGACTGCTGCCCAATGCTCTTTAACTACGATGACCGCGGCTGCCTGACCTTCGTCTCCAAGTTAGATATTCCAAAACAG
AGCATCCAACGCAACATGTCTGCCATGGAACGCTTCCGCAACATGGACAAGAGAGCCACAACTGAGGACCGCAACACG

GCCTTGGAGACGCTGCACCAGAATAGCATCACTCAAGTCTCTATTTATGAGGTGGACAAGCAAGATTGTCGCAAATTTT
GCACTACTGGCATCGATGGAGCCATGACAATTTGGGATTTCAAGACCCTCGAGTCTTCCATCCAGGGCCTCCGGATAAT
GTGAAGCTGAGTGAGCCTCCGCCATCCAGCATGACAAACTGTGGCCGACCGCAGCTGTGCCGTGGCACGATGGCGAG
GAAGCCAGCCCCAAGGAAACACTGAAAACACATATCACGCCAATGCCGTGTGGTTTTGTTTGAATATAAAATTGGTGAA
AGTGTTGGTTTTTTTAAGGCAGTAATTTTTTTGTTTGTTTTTTTGCGATTTCATTCCATTCTTGACCAAAGCTTCTCTTTAAG
TAGTTTATTATGGAAAATTGTCACACTAACTTAAAAGACAGGGTGAGGGAGATATGTAAATTgtccactagaaaattaaataaa
agaactgaatgt

>VIRT64494 (3-5’)
MAEAHSASHYPEALDGRLEGLEIPNCHGSIDASSAKFATILLVHLINRDLSDAILVQRLQ
GRVAVLSCGSLVHVAEAFHGRHVALDALFWNI
>VIRT65386 (5-3’)
MPFGQLMSEFGGSGTGGWVHGVSFSASGSRLAWVSHDSTVSVADASKSVQVSTLKTEFLP
LLSVSFVSENSVVAAGHDCCPMLFNYDDRGCLTFVSKLDIPKQSIQRNMSAMERFRNMDK
RATTEDRNTALETLHQNSITQVSIYEVDKQDCRKFCTTGIDGAMTIWDFKTLESSIQGLR
IM
>NM_001190996.1 Homo sapiens actin related protein 2/3 complex subunit 1A (ARPC1A), transcript variant 2, mRNA
ACTTCCGGGATCTGTCAGCCGCTCCCTCTGGGCTTCCGTCCTCCGCCCGCGCCCGACGGAGCCTGTTCGC
GTCGACTGCCCAGAGTCCGCGAATCCTCCGCTCCGAGCCCGTCCGGACTCCCCCGATCCCAGCTTTCTCT
CCTTTGAAAACACTAAGAATAATGTCACTGCATCAGTTTTTACTAGAGCCAATCACCTGTCATGCCTGGA
ACAGGGATCGTACTCATTGCCCTCAGTCCCAATAATCACGAAGTGCACATCTATAAGAAGAACGGGAGCC
AGTGGGTGAAAGCTCATGAACTCAAGGAGCACAACGGACACATCACAGGTATTGACTGGGCTCCCAAGAG
CGACCGCATTGTCACTTGTGGGGCAGACCGCAATGCCTATGTCTGGAGTCAGAAAGATGGTGTTTGGAAG
CCAACCCTGGTGATCCTGAGAATTAATCGCGCAGCTACTTTTGTGAAGTGGTCCCCCCTAGAGAACAAAT
TTGCTGTGGGAAGTGGAGCACGACTCATTTCTGTTTGTTACTTTGAGTCTGAAAATGACTGGTGGGTGAG
CAAGCACATTAAAAAGCCGATTCGCTCCACAGTCCTCAGCTTGGATTGGCATCCCAACAACGTTTTGCTG
GCAGCAGGATCATGTGACTTCAAATGCAGAGTGTTTTCTGCCTACATTAAAGAAGTGGATGAAAAGCCAG
CCAGCACGCCCTGGGGCAGCAAGATGCCTTTTGGGCAGCTGATGTCAGAGTTTGGTGGCAGTGGCACTGG
TGGCTGGGTCCACGGGGTAAGCTTCTCTGCCAGTGGGAGCCGCCTGGCCTGGGTCAGCCACGACAGCACC
GTGTCTGTTGCTGATGCCTCAAAAAGTGTGCAGGTCTCGACTCTGAAGACAGAGTTCCTGCCGCTCCTAA
GTGTGTCATTTGTCTCAGAGAACAGCGTCGTGGCTGCTGGCCATGACTGCTGCCCAATGCTCTTTAACTA
CGATGACCGCGGCTGCCTGACCTTCGTCTCCAAGTTAGATATTCCAAAACAGAGCATCCAACGCAACATG
TCTGCCATGGAACGCTTCCGCAACATGGACAAGAGAGCCACAACTGAGGACCGCAACACGGCCTTGGAGA
CGCTGCACCAGAATAGCATCACTCAAGTCTCTATTTATGAGGTGGACAAGCAAGATTGTCGCAAATTTTG
CACTACTGGCATCGATGGAGCCATGACAATTTGGGATTTCAAGACCCTCGAGTCTTCCATCCAGGGCCTC
CGGATAATGTGAAGCTGAGTGAGCCTCCGCCATCCAGCATGACAAACTGTGGCCGACCGCAGCTGTGCCG
TGGCACGATGGCGAGGAAGCCAGCCCCAAGGAAACACTGAAAACACATATCACGCCAATGCCGTGTGGTT
TTGTTTGAATATAAAATTGGTGAAAGTGTTGGTTTTTTTAAGGCAGTAATTTTTTTGTTTGTTTTTTTGC
GATTTCATTCCATTCTTGACCAAAGCTTCTCTTTAAGTAGTTTATTATGGAAAATTGTCACACTAACTTA
AAAGACAGGGTGAGGGAGATATGTAAATTGTCCACTAGAAAATTAAATAAAAGAACTGAATGTGGAAAAA
AAAAAAAAAAAAAAAAAAAAA
>NP_001177925.1 actin-related protein 2/3 complex subunit 1A isoform 2 [Homo sapiens]
MPGTGIVLIALSPNNHEVHIYKKNGSQWVKAHELKEHNGHITGIDWAPKSDRIVTCGADRNAYVWSQKDG
VWKPTLVILRINRAATFVKWSPLENKFAVGSGARLISVCYFESENDWWVSKHIKKPIRSTVLSLDWHPNN
VLLAAGSCDFKCRVFSAYIKEVDEKPASTPWGSKMPFGQLMSEFGGSGTGGWVHGVSFSASGSRLAWVSH
DSTVSVADASKSVQVSTLKTEFLPLLSVSFVSENSVVAAGHDCCPMLFNYDDRGCLTFVSKLDIPKQSIQ
RNMSAMERFRNMDKRATTEDRNTALETLHQNSITQVSIYEVDKQDCRKFCTTGIDGAMTIWDFKTLESSI
QGLRIM
>NM_006409.3 Homo sapiens actin related protein 2/3 complex subunit 1A (ARPC1A), transcript variant 1, mRNA
ACTTCCGGGATCTGTCAGCCGCTCCCTCTGGGCTTCCGTCCTCCGCCCGCGCCCGACGGAGCCTGTTCGC
GTCGACTGCCCAGAGTCCGCGAATCCTCCGCTCCGAGCCCGTCCGGACTCCCCCGATCCCAGCTTTCTCT
CCTTTGAAAACACTAAGAATAATGTCACTGCATCAGTTTTTACTAGAGCCAATCACCTGTCATGCCTGGA
ACAGGGATCGTACTCAGATTGCCCTCAGTCCCAATAATCACGAAGTGCACATCTATAAGAAGAACGGGAG
CCAGTGGGTGAAAGCTCATGAACTCAAGGAGCACAACGGACACATCACAGGTATTGACTGGGCTCCCAAG
AGCGACCGCATTGTCACTTGTGGGGCAGACCGCAATGCCTATGTCTGGAGTCAGAAAGATGGTGTTTGGA
AGCCAACCCTGGTGATCCTGAGAATTAATCGCGCAGCTACTTTTGTGAAGTGGTCCCCCCTAGAGAACAA
ATTTGCTGTGGGAAGTGGAGCACGACTCATTTCTGTTTGTTACTTTGAGTCTGAAAATGACTGGTGGGTG
AGCAAGCACATTAAAAAGCCGATTCGCTCCACAGTCCTCAGCTTGGATTGGCATCCCAACAACGTTTTGC
TGGCAGCAGGATCATGTGACTTCAAATGCAGAGTGTTTTCTGCCTACATTAAAGAAGTGGATGAAAAGCC
AGCCAGCACGCCCTGGGGCAGCAAGATGCCTTTTGGGCAGCTGATGTCAGAGTTTGGTGGCAGTGGCACT
GGTGGCTGGGTCCACGGGGTAAGCTTCTCTGCCAGTGGGAGCCGCCTGGCCTGGGTCAGCCACGACAGCA
CCGTGTCTGTTGCTGATGCCTCAAAAAGTGTGCAGGTCTCGACTCTGAAGACAGAGTTCCTGCCGCTCCT
AAGTGTGTCATTTGTCTCAGAGAACAGCGTCGTGGCTGCTGGCCATGACTGCTGCCCAATGCTCTTTAAC
TACGATGACCGCGGCTGCCTGACCTTCGTCTCCAAGTTAGATATTCCAAAACAGAGCATCCAACGCAACA

```
TGTCTGCCATGGAACGCTTCCGCAACATGGACAAGAGAGCCACAACTGAGGACCGCAACACGGCCTTGGA
GACGCTGCACCAGAATAGCATCACTCAAGTCTCTATTTATGAGGTGGACAAGCAAGATTGTCGCAAATTT
TGCACTACTGGCATCGATGGAGCCATGACAATTTGGGATTTCAAGACCCTCGAGTCTTCCATCCAGGGCC
TCCGGATAATGTGAAGCTGAGTGAGCCTCCGCCATCCAGCATGACAAACTGTGGCCGACCGCAGCTGTGC
CGTGGCACGATGGCGAGGAAGCCAGCCCCAAGGAAACACTGAAAACACATATCACGCCAATGCCGTGTGG
TTTTGTTTGAATATAAAATTGGTGAAAGTGTTGGTTTTTTTAAGGCAGTAATTTTTTTGTTTGTTTTTTT
GCGATTTCATTCCATTCTTGACCAAAGCTTCTCTTTAAGTAGTTTATTATGGAAAATTGTCACACTAACT
TAAAAGACAGGGTGAGGGAGATATGTAAATTGTCCACTAGAAAATTAAATAAAAGAACTGAATGTGGAAA
AAAAAAAAAAAAA
>NP_006400.2 actin-related protein 2/3 complex subunit 1A isoform 1 [Homo sapiens]
MSLHQFLLEPITCHAWNRDRTQIALSPNNHEVHIYKKNGSQWVKAHELKEHNGHITGIDWAPKSDRIVTC
GADRNAYVWSQKDGVWKPTLVILRINRAATFVKWSPLENKFAVGSGARLISVCYFESENDWWVSKHIKKP
IRSTVLSLDWHPNNVLLAAGSCDFKCRVFSAYIKEVDEKPASTPWGSKMPFGQLMSEFGGSGTGGWVHGV
SFSASGSRLAWVSHDSTVSVADASKSVQVSTLKTEFLPLLSVSFVSENSVVAAGHDCCPMLFNYDDRGCL
TFVSKLDIPKQSIQRNMSAMERFRNMDKRATTEDRNTALETLHQNSITQVSIYEVDKQDCRKFCTTGIDG
AMTIWDFKTLESSIQGLRIM
```

```
i1_HQ_samplec9816d|c18442/f2p0/1395      ------------------------------------------------------------ 0
NM_006409.3                              ACTTCCGGGATCTGTCAGCCGCTCCCTCTGGGCTTCCGTCCTCCGCCCGCGCCCGACGGA 60
i1_HQ_samplec9816d|c21346/f2p0/1056      ------------------------------------------------------------ 0
NM_001190996.1                           ACTTCCGGGATCTGTCAGCCGCTCCCTCTGGGCTTCCGTCCTCCGCCCGCGCCCGACGGA 60

i1_HQ_samplec9816d|c18442/f2p0/1395      ------------------------------------------------------------ 0
NM_006409.3                              GCCTGTTCGCGTCGACTGCCCAGAGTCCGCGAATCCTCCGCTCCGAGCCCGTCCGGACTC 120
i1_HQ_samplec9816d|c21346/f2p0/1056      ------------------------------------------------------------ 0
NM_001190996.1                           GCCTGTTCGCGTCGACTGCCCAGAGTCCGCGAATCCTCCGCTCCGAGCCCGTCCGGACTC 120

i1_HQ_samplec9816d|c18442/f2p0/1395      ------------------------------------------------------------ 0
NM_006409.3                              CCCCGATCCCAGCTTTCTCTCCTTTGAAAACACTAAGAATAATGTCACTGCATCAGTTTT 180
i1_HQ_samplec9816d|c21346/f2p0/1056      ------------------------------------------------------------ 0
NM_001190996.1                           CCCCGATCCCAGCTTTCTCTCCTTTGAAAACACTAAGAATAATGTCACTGCATCAGTTTT 180

i1_HQ_samplec9816d|c18442/f2p0/1395      -----------------------------aacagggatcgtactcagattgccctcagtc 31
NM_006409.3                              TACTAGAGCCAATCACCTGTCATGCCTGGAACAGGGATCGTACTCAGATTGCCCTCAGTC 240
i1_HQ_samplec9816d|c21346/f2p0/1056      ------------------------------------------------------------ 0
NM_001190996.1                           TACTAGAGCCAATCACCTGTCATGCCTGGAACAGGGATCGTACTC--ATTGCCCTCAGTC 238

i1_HQ_samplec9816d|c18442/f2p0/1395      ccaataatcacgaagtgcacatctataagaagaaCGGGAGCCAGTGGGTGAAAGCTCATG 91
NM_006409.3                              CCAATAATCACGAAGTGCACATCTATAAGAAGAACGGGAGCCAGTGGGTGAAAGCTCATG 300
i1_HQ_samplec9816d|c21346/f2p0/1056      ------------------------------------------------------------ 0
NM_001190996.1                           CCAATAATCACGAAGTGCACATCTATAAGAAGAACGGGAGCCAGTGGGTGAAAGCTCATG 298

i1_HQ_samplec9816d|c18442/f2p0/1395      AACTCAAGGAGCACAACGGACACATCACAGGTATTGACTGGGCTCCCAAGAGCGACCGCA 151
NM_006409.3                              AACTCAAGGAGCACAACGGACACATCACAGGTATTGACTGGGCTCCCAAGAGCGACCGCA 360
i1_HQ_samplec9816d|c21346/f2p0/1056      ------------------------------------------------------------ 0
NM_001190996.1                           AACTCAAGGAGCACAACGGACACATCACAGGTATTGACTGGGCTCCCAAGAGCGACCGCA 358

i1_HQ_samplec9816d|c18442/f2p0/1395      TTGTCACTTGTGGGGCAGACCGCAATGCCTATGTCTGGAGTCAGAAAGATGGTGTTTGGA 211
NM_006409.3                              TTGTCACTTGTGGGGCAGACCGCAATGCCTATGTCTGGAGTCAGAAAGATGGTGTTTGGA 420
i1_HQ_samplec9816d|c21346/f2p0/1056      ------------------------------------------------------------ 0
NM_001190996.1                           TTGTCACTTGTGGGGCAGACCGCAATGCCTATGTCTGGAGTCAGAAAGATGGTGTTTGGA 418

i1_HQ_samplec9816d|c18442/f2p0/1395      AGCCAACCCTGGTGATCCTGAGAATTAATCGCGCAGCTACTTTTGTGAAGTGGTCCCCCC 271
NM_006409.3                              AGCCAACCCTGGTGATCCTGAGAATTAATCGCGCAGCTACTTTTGTGAAGTGGTCCCCCC 480
i1_HQ_samplec9816d|c21346/f2p0/1056      ------------------------------------------------------------ 0
NM_001190996.1                           AGCCAACCCTGGTGATCCTGAGAATTAATCGCGCAGCTACTTTTGTGAAGTGGTCCCCCC 478

i1_HQ_samplec9816d|c18442/f2p0/1395      TAGAGAACAAATTTGCTGTGGGAAGTGGAGCACGACTCATTTCTGTTTGTTACTTTGAGT 331
NM_006409.3                              TAGAGAACAAATTTGCTGTGGGAAGTGGAGCACGACTCATTTCTGTTTGTTACTTTGAGT 540
i1_HQ_samplec9816d|c21346/f2p0/1056      ------------------------------------------------------------ 0
NM_001190996.1                           TAGAGAACAAATTTGCTGTGGGAAGTGGAGCACGACTCATTTCTGTTTGTTACTTTGAGT 538

i1_HQ_samplec9816d|c18442/f2p0/1395      CTGAAAATGACTGGTGGGTGAGCAAGCACATTAAAAAGCCGATTCGCTCCACAGTCCTCA 391
NM_006409.3                              CTGAAAATGACTGGTGGGTGAGCAAGCACATTAAAAAGCCGATTCGCTCCACAGTCCTCA 600
i1_HQ_samplec9816d|c21346/f2p0/1056      ---------actggtgggtgagcaagcacattaaaaagccgattcgctccacagtcctca 51
NM_001190996.1                           CTGAAAATGACTGGTGGGTGAGCAAGCACATTAAAAAGCCGATTCGCTCCACAGTCCTCA 598
                                                  ***************************************************

i1_HQ_samplec9816d|c18442/f2p0/1395      GCTTGGATTGGCATCCCAACAACGTTTTGCTGGCAGCAGGATCATGTGACTTCAAATGCA 451
NM_006409.3                              GCTTGGATTGGCATCCCAACAACGTTTTGCTGGCAGCAGGATCATGTGACTTCAAATGCA 660
```

```
i1_HQ_samplec9816d|c21346/f2p0/1056      gcTTGGATTGGCATCCCAACAACGTTTTGCTGGCAGCAGGATCATGTGACTTCAAATGCA 111
NM_001190996.1                           GCTTGGATTGGCATCCCAACAACGTTTTGCTGGCAGCAGGATCATGTGACTTCAAATGCA 658
                                         ************************************************************

i1_HQ_samplec9816d|c18442/f2p0/1395      GAGTGTTTTCTGCCTACATTAAAGAAGTGGATGAAAAGCCAGCCAGCACGCCCTGGGGCA 511
NM_006409.3                              GAGTGTTTTCTGCCTACATTAAAGAAGTGGATGAAAAGCCAGCCAGCACGCCCTGGGGCA 720
i1_HQ_samplec9816d|c21346/f2p0/1056      GAGTGTTTTCTGCCTACATTAAAGAAGTGGATGAAAAGCCAGCCAGCACGCCCTGGGGCA 171
NM_001190996.1                           GAGTGTTTTCTGCCTACATTAAAGAAGTGGATGAAAAGCCAGCCAGCACGCCCTGGGGCA 718
                                         ************************************************************

i1_HQ_samplec9816d|c18442/f2p0/1395      GCAAGATGCCTTTTGGGCAGCTGATGTCAGAGTTTGGTGGCAGTGGCACTGGTGGCTGGG 571
NM_006409.3                              GCAAGATGCCTTTTGGGCAGCTGATGTCAGAGTTTGGTGGCAGTGGCACTGGTGGCTGGG 780
i1_HQ_samplec9816d|c21346/f2p0/1056      GCAAGATGCCTTTTGGGCAGCTGATGTCAGAGTTTGGTGGCAGTGGCACTGGTGGCTGGG 231
NM_001190996.1                           GCAAGATGCCTTTTGGGCAGCTGATGTCAGAGTTTGGTGGCAGTGGCACTGGTGGCTGGG 778
                                         ************************************************************

i1_HQ_samplec9816d|c18442/f2p0/1395      TCCACGGGGTAAGCTTCTCTGCCAGTGGGAGCCGCCTGGCCTGGGTCAGCCACGACAGCA 631
NM_006409.3                              TCCACGGGGTAAGCTTCTCTGCCAGTGGGAGCCGCCTGGCCTGGGTCAGCCACGACAGCA 840
i1_HQ_samplec9816d|c21346/f2p0/1056      TCCACGGGGTAAGCTTCTCTGCCAGTGGGAGCCGCCTGGCCTGGGTCAGCCACGACAGCA 291
NM_001190996.1                           TCCACGGGGTAAGCTTCTCTGCCAGTGGGAGCCGCCTGGCCTGGGTCAGCCACGACAGCA 838
                                         ************************************************************

i1_HQ_samplec9816d|c18442/f2p0/1395      CCGTGTCTGTTGCTGATGCCTCAAAAAGTGTGCAGGTCTCGACTCTGAAGACAGAGTTCC 691
NM_006409.3                              CCGTGTCTGTTGCTGATGCCTCAAAAAGTGTGCAGGTCTCGACTCTGAAGACAGAGTTCC 900
i1_HQ_samplec9816d|c21346/f2p0/1056      CCGTGTCTGTTGCTGATGCCTCAAAAAGTGTGCAGGTCTCGACTCTGAAGACAGAGTTCC 351
NM_001190996.1                           CCGTGTCTGTTGCTGATGCCTCAAAAAGTGTGCAGGTCTCGACTCTGAAGACAGAGTTCC 898
                                         ************************************************************

i1_HQ_samplec9816d|c18442/f2p0/1395      TGCCGCTCCTAAGTGTGTCATTTGTCTCAGAGAACAGCGTCGTGGCTGCTGGCCATGACT 751
NM_006409.3                              TGCCGCTCCTAAGTGTGTCATTTGTCTCAGAGAACAGCGTCGTGGCTGCTGGCCATGACT 960
i1_HQ_samplec9816d|c21346/f2p0/1056      TGCCGCTCCTAAGTGTGTCATTTGTCTCAGAGAACAGCGTCGTGGCTGCTGGCCATGACT 411
NM_001190996.1                           TGCCGCTCCTAAGTGTGTCATTTGTCTCAGAGAACAGCGTCGTGGCTGCTGGCCATGACT 958
                                         ************************************************************

i1_HQ_samplec9816d|c18442/f2p0/1395      GCTGCCCAATGCTCTTTAACTACGATGACCGCGGCTGCCTGACCTTCGTCTCCAAGTTAG 811
NM_006409.3                              GCTGCCCAATGCTCTTTAACTACGATGACCGCGGCTGCCTGACCTTCGTCTCCAAGTTAG 1020
i1_HQ_samplec9816d|c21346/f2p0/1056      GCTGCCCAATGCTCTTTAACTACGATGACCGCGGCTGCCTGACCTTCGTCTCCAAGTTAG 471
NM_001190996.1                           GCTGCCCAATGCTCTTTAACTACGATGACCGCGGCTGCCTGACCTTCGTCTCCAAGTTAG 1018
                                         ************************************************************

i1_HQ_samplec9816d|c18442/f2p0/1395      ATATTCCAAAACAGAGCATCCAACGCAACATGTCTGCCATGGAACGCTTCCGCAACATGG 871
NM_006409.3                              ATATTCCAAAACAGAGCATCCAACGCAACATGTCTGCCATGGAACGCTTCCGCAACATGG 1080
i1_HQ_samplec9816d|c21346/f2p0/1056      ATATTCCAAAACAGAGCATCCAACGCAACATGTCTGCCATGGAACGCTTCCGCAACATGG 531
NM_001190996.1                           ATATTCCAAAACAGAGCATCCAACGCAACATGTCTGCCATGGAACGCTTCCGCAACATGG 1078
                                         ************************************************************

i1_HQ_samplec9816d|c18442/f2p0/1395      ACAAGAGAGCCACAACTGAGGACCGCAACACGGCCTTGGAGACGCTGCACCAGAATAGCA 931
NM_006409.3                              ACAAGAGAGCCACAACTGAGGACCGCAACACGGCCTTGGAGACGCTGCACCAGAATAGCA 1140
i1_HQ_samplec9816d|c21346/f2p0/1056      ACAAGAGAGCCACAACTGAGGACCGCAACACGGCCTTGGAGACGCTGCACCAGAATAGCA 591
NM_001190996.1                           ACAAGAGAGCCACAACTGAGGACCGCAACACGGCCTTGGAGACGCTGCACCAGAATAGCA 1138
                                         ************************************************************

i1_HQ_samplec9816d|c18442/f2p0/1395      TCACTCAAGTCTCTATTTATGAGGTGGACAAGCAAGATTGTCGCAAATTTTGCACTACTG 991
NM_006409.3                              TCACTCAAGTCTCTATTTATGAGGTGGACAAGCAAGATTGTCGCAAATTTTGCACTACTG 1200
i1_HQ_samplec9816d|c21346/f2p0/1056      TCACTCAAGTCTCTATTTATGAGGTGGACAAGCAAGATTGTCGCAAATTTTGCACTACTG 651
NM_001190996.1                           TCACTCAAGTCTCTATTTATGAGGTGGACAAGCAAGATTGTCGCAAATTTTGCACTACTG 1198
                                         ************************************************************

i1_HQ_samplec9816d|c18442/f2p0/1395      GCATCGATGGAGCCATGACAATTTGGGATTTCAAGACCCTCGAGTCTTCCATCCAGGGCC 1051
NM_006409.3                              GCATCGATGGAGCCATGACAATTTGGGATTTCAAGACCCTCGAGTCTTCCATCCAGGGCC 1260
i1_HQ_samplec9816d|c21346/f2p0/1056      GCATCGATGGAGCCATGACAATTTGGGATTTCAAGACCCTCGAGTCTTCCATCCAGGGCC 711
NM_001190996.1                           GCATCGATGGAGCCATGACAATTTGGGATTTCAAGACCCTCGAGTCTTCCATCCAGGGCC 1258
                                         ************************************************************

i1_HQ_samplec9816d|c18442/f2p0/1395      TCCGGATAATGTGAAGCTGAGTGAGCCTCCGCCATCCAGCATGACAAACTGTGGCCGACC 1111
NM_006409.3                              TCCGGATAATGTGAAGCTGAGTGAGCCTCCGCCATCCAGCATGACAAACTGTGGCCGACC 1320
i1_HQ_samplec9816d|c21346/f2p0/1056      TCCGGATAATGTGAAGCTGAGTGAGCCTCCGCCATCCAGCATGACAAACTGTGGCCGACC 771
NM_001190996.1                           TCCGGATAATGTGAAGCTGAGTGAGCCTCCGCCATCCAGCATGACAAACTGTGGCCGACC 1318
                                         ************************************************************

i1_HQ_samplec9816d|c18442/f2p0/1395      GCAGCTGTGCCGTGGCACGATGGCGAGGAAGCCAGCCCCAAGGAAACACTGAAAACACAT 1171
NM_006409.3                              GCAGCTGTGCCGTGGCACGATGGCGAGGAAGCCAGCCCCAAGGAAACACTGAAAACACAT 1380
i1_HQ_samplec9816d|c21346/f2p0/1056      GCAGCTGTGCCGTGGCACGATGGCGAGGAAGCCAGCCCCAAGGAAACACTGAAAACACAT 831
NM_001190996.1                           GCAGCTGTGCCGTGGCACGATGGCGAGGAAGCCAGCCCCAAGGAAACACTGAAAACACAT 1378
                                         ************************************************************

i1_HQ_samplec9816d|c18442/f2p0/1395      ATCACGCCAATGCCGTGTGGTTTTGTTTGAATATAAAATTGGTGAAAGTGTTGGTTTTTT 1231
NM_006409.3                              ATCACGCCAATGCCGTGTGGTTTTGTTTGAATATAAAATTGGTGAAAGTGTTGGTTTTTT 1440
i1_HQ_samplec9816d|c21346/f2p0/1056      ATCACGCCAATGCCGTGTGGTTTTGTTTGAATATAAAATTGGTGAAAGTGTTGGTTTTTT 891
NM_001190996.1                           ATCACGCCAATGCCGTGTGGTTTTGTTTGAATATAAAATTGGTGAAAGTGTTGGTTTTTT 1438
                                         ************************************************************
```

```
i1_HQ_samplec9816d|c18442/f2p0/1395      TAAGGCAGTAATTTTTTTGTTTGTTTTTTTGCGATTTCATTCCATTCTTGACCAAAGCTT 1291
NM_006409.3                              TAAGGCAGTAATTTTTTTGTTTGTTTTTTTGCGATTTCATTCCATTCTTGACCAAAGCTT 1500
i1_HQ_samplec9816d|c21346/f2p0/1056      TAAGGCAGTAATTTTTTTGTTTGTTTTTTTGCGATTTCATTCCATTCTTGACCAAAGCTT 951
NM_001190996.1                           TAAGGCAGTAATTTTTTTGTTTGTTTTTTTGCGATTTCATTCCATTCTTGACCAAAGCTT 1498
                                         ************************************************************

i1_HQ_samplec9816d|c18442/f2p0/1395      CTCTTTAAGTAGTTTATTATGGAAAATTGTCACACTAACTTAAAAGACAGGGTGAGGGAG 1351
NM_006409.3                              CTCTTTAAGTAGTTTATTATGGAAAATTGTCACACTAACTTAAAAGACAGGGTGAGGGAG 1560
i1_HQ_samplec9816d|c21346/f2p0/1056      CTCTTTAAGTAGTTTATTATGGAAAATTGTCACACTAACTTAAAAGACAGGGTGAGGGAG 1011
NM_001190996.1                           CTCTTTAAGTAGTTTATTATGGAAAATTGTCACACTAACTTAAAAGACAGGGTGAGGGAG 1558
                                         ************************************************************

i1_HQ_samplec9816d|c18442/f2p0/1395      ATATGTAAATtgtccactagaaaattaaataaaagaactgaatg---------------- 1395
NM_006409.3                              ATATGTAAATTGTCCACTAGAAAATTAAATAAAAGAACTGAATGTGGAAAAAAAAAAAAA 1620
i1_HQ_samplec9816d|c21346/f2p0/1056      ATATGTAAATTgtccactagaaaattaaataaaagaactgaatgt--------------- 1056
NM_001190996.1                           ATATGTAAATTGTCCACTAGAAAATTAAATAAAAGAACTGAATGTGGAAAAAAAAAAAAA 1618
                                         ********************************************

i1_HQ_samplec9816d|c18442/f2p0/1395      -------------        1395
NM_006409.3                              AAA----------        1623
i1_HQ_samplec9816d|c21346/f2p0/1056      -------------        1056
NM_001190996.1                           AAAAAAAAAAAAA        1631


VIRT50347                   -------------------------MAEAHSAS------------HYPEALDGRLEGLEI  23
VIRT64494                   -------------------------MAEAHSAS------------HYPEALDGRLEGLEI  23
NP_006400.2                 MSLHQFLLEPITCHAWNRDRTQIALSPNNHEVHIYKKNGSQWVKAHELKEHNGHITGIDW  60
VIRT61659                   ------------------------------------------------------------  0
VIRT65386                   ------------------------------------------------------------  0
NP_001177925.1              --------------MPGTGIVLIALSPNNHEVHIYKKNGSQWVKAHELKEHNGHITGIDW  46


VIRT50347                   P-------NCHGSIDA-----SSAKFATILLVHLINRDLSDAILVQRLQGRVAVLSCGSL  71
VIRT64494                   P-------NCHGSIDA-----SSAKFATILLVHLINRDLSDAILVQRLQGRVAVLSCGSL  71
NP_006400.2                 APKSDRIVTCGADRNAYVWSQKDGVWKPTLVILRINRAAT-FVKWSPLENKFAVGSGARL  119
VIRT61659                   ------------------------------------------------------------  0
VIRT65386                   ------------------------------------------------------------  0
NP_001177925.1              APKSDRIVTCGADRNAYVWSQKDGVWKPTLVILRINRAAT-FVKWSPLENKFAVGSGARL  105


VIRT50347                   V-----------------------------------------------------------  72
VIRT64494                   V-----------------------------------------------------------  72
NP_006400.2                 ISVCYFESENDWWVSKHIKKPIRSTVLSLDWHPNNVLLAAGSCDFKCRVFSAYIKEVDEK  179
VIRT61659                   ------------------------------------------------------------  0
VIRT65386                   ------------------------------------------------------------  0
NP_001177925.1              ISVCYFESENDWWVSKHIKKPIRSTVLSLDWHPNNVLLAAGSCDFKCRVFSAYIKEVDEK  165


VIRT50347                   -------------------------------------------------HVA--------  75
VIRT64494                   -------------------------------------------------HVA--------  75
NP_006400.2                 PASTPWGSKMPFGQLMSEFGGSGTGGWVHGVSFSASGSRLAWVSHDSTVSVADASKSVQV  239
VIRT61659                   ---------MPFGQLMSEFGGSGTGGWVHGVSFSASGSRLAWVSHDSTVSVADASKSVQV  51
VIRT65386                   ---------MPFGQLMSEFGGSGTGGWVHGVSFSASGSRLAWVSHDSTVSVADASKSVQV  51
NP_001177925.1              PASTPWGSKMPFGQLMSEFGGSGTGGWVHGVSFSASGSRLAWVSHDSTVSVADASKSVQV  225
                                                                              **

VIRT50347                   ------------------------------------------------------------  75
VIRT64494                   ------------------------------------------------------------  75
NP_006400.2                 STLKTEFLPLLSVSFVSENSVVAAGHDCCPMLFNYDDRGCLTFVSKLDIPKQSIQRNMSA  299
VIRT61659                   STLKTEFLPLLSVSFVSENSVVAAGHDCCPMLFNYDDRGCLTFVSKLDIPKQSIQRNMSA  111
VIRT65386                   STLKTEFLPLLSVSFVSENSVVAAGHDCCPMLFNYDDRGCLTFVSKLDIPKQSIQRNMSA  111
NP_001177925.1              STLKTEFLPLLSVSFVSENSVVAAGHDCCPMLFNYDDRGCLTFVSKLDIPKQSIQRNMSA  285


VIRT50347                   -----------------------------------------EAFHGRHVALDALFWNI--  92
VIRT64494                   -----------------------------------------EAFHGRHVALDALFWNI--  92
NP_006400.2                 MERFRNMDKRATTEDRNTALETLHQNSITQVSIYEVDKQDCRKFCTTGIDGAMTIWDFKT  359
VIRT61659                   MERFRNMDKRATTEDRNTALETLHQNSITQVSIYEVDKQDCRKFCTTGIDGAMTIWDFKT  171
VIRT65386                   MERFRNMDKRATTEDRNTALETLHQNSITQVSIYEVDKQDCRKFCTTGIDGAMTIWDFKT  171
NP_001177925.1              MERFRNMDKRATTEDRNTALETLHQNSITQVSIYEVDKQDCRKFCTTGIDGAMTIWDFKT  345
                                                                     . *    :     :*::

VIRT50347                   -----------         92
VIRT64494                   -----------         92
NP_006400.2                 LESSIQGLRIM         370
VIRT61659                   LESSIQGLRIM         182
VIRT65386                   LESSIQGLRIM         182
NP_001177925.1              LESSIQGLRIM         356
```

**ARPC1B**

| **Sequence** | **Header** | **AA Seq** | | |
|---|---|---|---|---|
| **Present** | i1_HQ_samplec9816d\|c43701/f2p0/1471 | VIRT91963 (3-5’) | VIRT95841 (3-5’) | VIRT89465 (5-3’) |
| **Absent** | i1_HQ_samplec9816d\|c14866/f2p0/1185 | >VIRT99058 | | |
| **NCBI** | NM_005720 | >NP_005711.1 | | |

>i1_HQ_samplec9816d|c43701/f2p0/1471
isoform=c43701;full_length_coverage=2;non_full_length_coverage=0;isoform_length=1471

agagccggttcggcgcgtcgactgcccagagtCCGCGGCCGGGGCGCGGGAGGAGCCAAGCCGCCATGGCCTACCACAGCTTCCT
GGTGGAGCCCATCAGCTGCCACGCCTGGAACAAGGACCGCACCCAGATTGCCATCTGCCCCAACAACCATGAGGTGCA
TATCTATGAAAAGAGCGGTGCCAAATGGACCAAGGTGCACGAGCTCAAGGAGCACAACGGGCAGGTGACAGGCATCG
ACTGGGCCCCCGAGAGTAACCGTATTGTGACCTGCGGCACAGACCGCAACGCCTACGTGTGGACGCTGAAGGGCCGCA
CATGGAAGCCCACGCTGGTCATCCTGCGGATCAACCGGGCTGCCCGCTGCGTGCGCTGGGCCCCCAACGAGAACAAGT
TTGCTGTGGGCAGCGGCTCTCGTGTGATCTCCATCTGTTATTTCGAGCAGGAGAATGACTGGTGGGTTTGCAAGCACAT
CAAGAAGCCCATCCGCTCCACCGTCCTCAGCCTGGACTGGCACCCCAACAATGTGCTGCTGGCTGCCGGCTCCTGTGAC
TTCAAGTGTCGGATCTTTTCAGCCTACATCAAGGAGGTGGAGGAACGGCCGGCACCCACCCCGTGGGGCTCCAAGATG
CCCTTTGGGGAACTGATGTTCGAATCCAGCAGTAGCTGCGGCTGGGTACATGGCGTCTGTTTCTCAGCCAGCGGGAGC
CGCGTGGCCTGGGTAAGCCACGACAGCACCGTCTGCCTGGCTGATGCCGACAAGAAGATGGCCGTCGCGACTCTGGCC
TCTGAAACACTACCACTGCTGGCGCTGACCTTCATCACAGACAACAGCCTGGTGGCAGCGGGCCACGACTGCTTCCCGG
TGCTGTTCACCTATGACGCCGCCGCGGGGATGCTGAGCTTCGGCGGGCGGCTGGACGTTCCTAAGCAGAGCTCGCAGC
GTGGCTTGACGGCCCGCGAGCGCTTCCAGAACCTGGACAAGAAGGCGAGCTCCGAGGGTGGCACGGCTGCGGGCGCG
GGCCTAGACTCGCTGCACAAGAACAGCGTCAGCCAGATCTCGGTGCTCAGCGGCGGCAAGGCCAAGTGCTCGCAGTTC
TGCACCACTGGCATGGATGGCGGCATGAGTATCTGGGATGTGAAGAGCTTGGAGTCAGCCTTGAAGGACCTCAAGATC
AAATGACCTGTGAGGAATATGTTGCCTTCATCCTAGCTGCTGGGGAAGCGGGGAGAGGGGTCAGGGAGGCTAATGGT
TGCTTTGCTGAATGTTTCTGGGGTACCAATACGAGTTCCCATAGGGGCTGCTCCCTCAAAAAGGGAGGGGACAGATGG
GGAGCTTTTCTTACCTATTCAAGGAATACGTGCCTTTTTCTTAAATGCTTTCATTTATTGAAAAAAAAAAAAAAATGCCCCC
AAAGCACTATGCTGGTcatgaactgcttcaaaatgtggaggtaataaaatgcaactg

>VIRT91963 (3-5’)

MPPSMPVVQNCEHLALPPLSTEIWLTLFLCSESRPAPAAVPPSELAFLSRFWKRSRAVKP
RCELCLGTSSRPPKLSIPAAAS

>VIRT95841 (3-5’)

MKVSASSGSVSEARVATAIFLSASARQTVLSWLTQATRLPLAEKQTPCTQPQLLLDSNIS
SPKGILEPHGVGAGRSSTSLM

>VIRT89465 (5-3’)

MAYHSFLVEPISCHAWNKDRTQIAICPNNHEVHIYEKSGAKWTKVHELKEHNGQVTGIDW
APESNRIVTCGTDRNAYVWTLKGRTWKPTLVILRINRAARCVRWAPNENKFAVGSGSRVI

SICYFEQENDWWVCKHIKKPIRSTVLSLDWHPNNVLLAAGSCDFKCRIFSAYIKEVEERP
APTPWGSKMPFGELMFESSSSCGWVHGVCFSASGSRVAWVSHDSTVCLADADKKMAVATL
ASETLPLLALTFITDNSLVAAGHDCFPVLFTYDAAAGMLSFGGRLDVPKQSSQRGLTARE
RFQNLDKKASSEGGTAAGAGLDSLHKNSVSQISVLSGGKAKCSQFCTTGMDGGMSIWDVK
SLESALKDLKIK

>i1_HQ_samplec9816d|c14866/f2p0/1185
isoform=c14866;full_length_coverage=2;non_full_length_coverage=0;isoform_length=1185

aggaagtggctgctgctccggcgcggagcccagagccggttcggcgcgtcgactgcccagagtccgcggCCGGGGCGCGGGAGGAGCCAAGC
CGCCATGGCCTACCACAGCTTCCTGGTGGAGCCCATCAGCTGCCACGCCTGGAACAAGGACCGCACCCAGATTGCCATC
TGCCCCAACAACCATGAGGTGCATATCTATGAAAAGAGCGGTGCCAAATGGACCAAGGTGCACGAGCTCAAGGAGCAC
AACGGGCAGGTGACAGGCATCGACTGGGCCCCCGAGAGTAACCGTATTGTGACCTGCGGCACAGACCGCAACGCCTAC
GTGTGGACGCTGAAGGGCCGCACATGGAAGCCCACGCTGGTCATCCTGCGGATCAACCGGGCTGCCCGCTGCGTGCGC
TGGGCCCCCAACGAGAACAAGTTTGCTGTGGGCAGCGGCTCTCGTGTGATCTCCATCTGTTATTTCGAGCAGGAGAATG
ACTGGTGGGTTTGCAAGCACATCAAGAAGCCCATCCGCTCCACCGTCCTCAGCCTGGACTGGCACCCCAACAATGTGCT
GCTGGCTGCCGGCTCCTGTGACTTCAAGTGTCGGATCTTTTCAGCCTACATCAAGGAGGTGGAGGAACGGCCGGCACC
CACCCCGTGGGGCTCCAAGATGCCCTTTGGGGAACTGATGTTCGAATCCAGCAGTAGCTGCGGCTGGGTACATGGCGT
CTGTTTCTCAGCCAGCGGGAGCCGCGTGGCCTGGGTAAGCCACGACAGCACCGTCTGCCTGGCTGATGCCGACAAGGC
CAAGTGCTCGCAGTTCTGCACCACTGGCATGGATGGCGGCATGAGTATCTGGGATGTGAAGAGCTTGGAGTCAGCCTT
GAAGGACCTCAAGATCAAATGACCTGTGAGGAATATGTTGCCTTCATCCTAGCTGCTGGGGAAGCGGGGAGAGGGGT
CAGGGAGGCTAATGGTTGCTTTGCTGAATGTTTCTGGGGTACCAATACGAGTTCCCATAGGGGCTGCTCCCTCAAAAAG
GGAGGGGACAGATGGGGAGCTTTTCTTACCTATTCAAGGAATACGTGCCTTTTTCTTAAATGCTTTCATTTATTGAAAAA
AAAAAAAAATGCCCCCAAAGCACTATGCTGGTCATGAACTGcttcaaaatgtggaggtaataaaatgcaactgt

>VIRT99058

MAYHSFLVEPISCHAWNKDRTQIAICPNNHEVHIYEKSGAKWTKVHELKEHNGQVTGIDW
APESNRIVTCGTDRNAYVWTLKGRTWKPTLVILRINRAARCVRWAPNENKFAVGSGSRVI
SICYFEQENDWWVCKHIKKPIRSTVLSLDWHPNNVLLAAGSCDFKCRIFSAYIKEVEERP
APTPWGSKMPFGELMFESSSSCGWVHGVCFSASGSRVAWVSHDSTVCLADADKAKCSQFC
TTGMDGGMSIWDVKSLESALKDLKIK

>NM_005720.4 Homo sapiens actin related protein 2/3 complex subunit 1B (ARPC1B), mRNA
AGAGCCGGTTCGGCGCGTCGACTGCCCAGAGTCCGCGGCCGGGGCGCGGGAGGAGCCAAGCCGCCATGGC
CTACCACAGCTTCCTGGTGGAGCCCATCAGCTGCCACGCCTGGAACAAGGACCGCACCCAGATTGCCATC
TGCCCCAACAACCATGAGGTGCATATCTATGAAAAGAGCGGTGCCAAATGGACCAAGGTGCACGAGCTCA
AGGAGCACAACGGGCAGGTGACAGGCATCGACTGGGCCCCCGAGAGTAACCGTATTGTGACCTGCGGCAC
AGACCGCAACGCCTACGTGTGGACGCTGAAGGGCCGCACATGGAAGCCCACGCTGGTCATCCTGCGGATC
AACCGGGCTGCCCGCTGCGTGCGCTGGGCCCCCAACGAGAACAAGTTTGCTGTGGGCAGCGGCTCTCGTG
TGATCTCCATCTGTTATTTCGAGCAGGAGAATGACTGGTGGGTTTGCAAGCACATCAAGAAGCCCATCCG
CTCCACCGTCCTCAGCCTGGACTGGCACCCCAACAATGTGCTGCTGGCTGCCGGCTCCTGTGACTTCAAG
TGTCGGATCTTTTCAGCCTACATCAAGGAGGTGGAGGAACGGCCGGCACCCACCCCGTGGGGCTCCAAGA
TGCCCTTTGGGGAACTGATGTTCGAATCCAGCAGTAGCTGCGGCTGGGTACATGGCGTCTGTTTCTCAGC
CAGCGGGAGCCGCGTGGCCTGGGTAAGCCACGACAGCACCGTCTGCCTGGCTGATGCCGACAAGAAGATG
GCCGTCGCGACTCTGGCCTCTGAAACACTACCACTGCTGGCGCTGACCTTCATCACAGACAACAGCCTGG
TGGCAGCGGGCCACGACTGCTTCCCGGTGCTGTTCACCTATGACGCCGCCGCGGGGATGCTGAGCTTCGG
CGGGCGGCTGGACGTTCCTAAGCAGAGCTCGCAGCGTGGCTTGACGGCCCGCGAGCGCTTCCAGAACCTG
GACAAGAAGGCGAGCTCCGAGGGTGGCACGGCTGCGGGCGCGGGCCTAGACTCGCTGCACAAGAACAGCG
TCAGCCAGATCTCGGTGCTCAGCGGCGGCAAGGCCAAGTGCTCGCAGTTCTGCACCACTGGCATGGATGG
CGGCATGAGTATCTGGGATGTGAAGAGCTTGGAGTCAGCCTTGAAGGACCTCAAGATCAAATGACCTGTG
AGGAATATGTTGCCTTCATCCTAGCTGCTGGGGAAGCGGGGAGAGGGGTCAGGGAGGCTAATGGTTGCTT
TGCTGAATGTTTCTGGGGTACCAATACGAGTTCCCATAGGGGCTGCTCCCTCAAAAAGGGAGGGGACAGA

```
TGGGGAGCTTTTCTTACCTATTCAAGGAATACGTGCCTTTTTCTTAAATGCTTTCATTTATTGAAAAAAA
AAAAAAATGCCCCCAAAGCACTATGCTGGTCATGAACTGCTTCAAAATGTGGAGGTAATAAAATGCAACT
GTGTAAAAAAAAAAAAAAAAAAAAAAAAGTAATTATGGACATG
```

```
>NP_005711.1 actin-related protein 2/3 complex subunit 1B [Homo sapiens]
MAYHSFLVEPISCHAWNKDRTQIAICPNNHEVHIYEKSGAKWTKVHELKEHNGQVTGIDWAPESNRIVTC
GTDRNAYVWTLKGRTWKPTLVILRINRAARCVRWAPNENKFAVGSGSRVISICYFEQENDWWVCKHIKKP
IRSTVLSLDWHPNNVLLAAGSCDFKCRIFSAYIKEVEERPAPTPWGSKMPFGELMFESSSSCGWVHGVCF
SASGSRVAWVSHDSTVCLADADKKMAVATLASETLPLLALTFITDNSLVAAGHDCFPVLFTYDAAAGMLS
FGGRLDVPKQSSQRGLTARERFQNLDKKASSEGGTAAGAGLDSLHKNSVSQISVLSGGKAKCSQFCTTGM
DGGMSIWDVKSLESALKDLKIK
```

```
i1_HQ_samplec9816d|c14866/f2p0/1185      aggaagtggctgctgctccggcgcggagcccagagccggttcggcgcgtcgactgcccag 60
i1_HQ_samplec9816d|c43701/f2p0/1471      -------------------------------agagccggttcggcgcgtcgactgcccag 29
NM_005720.4                              -------------------------------AGAGCCGGTTCGGCGCGTCGACTGCCCAG 29
                                                                        *****************************

i1_HQ_samplec9816d|c14866/f2p0/1185      agtccgcggCCGGGGCGCGGGAGGAGCCAAGCCGCCATGGCCTACCACAGCTTCCTGGTG 120
i1_HQ_samplec9816d|c43701/f2p0/1471      agtCCGCGGCCGGGGCGCGGGAGGAGCCAAGCCGCCATGGCCTACCACAGCTTCCTGGTG 89
NM_005720.4                              AGTCCGCGGCCGGGGCGCGGGAGGAGCCAAGCCGCCATGGCCTACCACAGCTTCCTGGTG 89
                                         ************************************************************

i1_HQ_samplec9816d|c14866/f2p0/1185      GAGCCCATCAGCTGCCACGCCTGGAACAAGGACCGCACCCAGATTGCCATCTGCCCCAAC 180
i1_HQ_samplec9816d|c43701/f2p0/1471      GAGCCCATCAGCTGCCACGCCTGGAACAAGGACCGCACCCAGATTGCCATCTGCCCCAAC 149
NM_005720.4                              GAGCCCATCAGCTGCCACGCCTGGAACAAGGACCGCACCCAGATTGCCATCTGCCCCAAC 149
                                         ************************************************************

i1_HQ_samplec9816d|c14866/f2p0/1185      AACCATGAGGTGCATATCTATGAAAAGAGCGGTGCCAAATGGACCAAGGTGCACGAGCTC 240
i1_HQ_samplec9816d|c43701/f2p0/1471      AACCATGAGGTGCATATCTATGAAAAGAGCGGTGCCAAATGGACCAAGGTGCACGAGCTC 209
NM_005720.4                              AACCATGAGGTGCATATCTATGAAAAGAGCGGTGCCAAATGGACCAAGGTGCACGAGCTC 209
                                         ************************************************************

i1_HQ_samplec9816d|c14866/f2p0/1185      AAGGAGCACAACGGGCAGGTGACAGGCATCGACTGGGCCCCCGAGAGTAACCGTATTGTG 300
i1_HQ_samplec9816d|c43701/f2p0/1471      AAGGAGCACAACGGGCAGGTGACAGGCATCGACTGGGCCCCCGAGAGTAACCGTATTGTG 269
NM_005720.4                              AAGGAGCACAACGGGCAGGTGACAGGCATCGACTGGGCCCCCGAGAGTAACCGTATTGTG 269
                                         ************************************************************

i1_HQ_samplec9816d|c14866/f2p0/1185      ACCTGCGGCACAGACCGCAACGCCTACGTGTGGACGCTGAAGGGCCGCACATGGAAGCCC 360
i1_HQ_samplec9816d|c43701/f2p0/1471      ACCTGCGGCACAGACCGCAACGCCTACGTGTGGACGCTGAAGGGCCGCACATGGAAGCCC 329
NM_005720.4                              ACCTGCGGCACAGACCGCAACGCCTACGTGTGGACGCTGAAGGGCCGCACATGGAAGCCC 329
                                         ************************************************************

i1_HQ_samplec9816d|c14866/f2p0/1185      ACGCTGGTCATCCTGCGGATCAACCGGGCTGCCCGCTGCGTGCGCTGGGCCCCCAACGAG 420
i1_HQ_samplec9816d|c43701/f2p0/1471      ACGCTGGTCATCCTGCGGATCAACCGGGCTGCCCGCTGCGTGCGCTGGGCCCCCAACGAG 389
NM_005720.4                              ACGCTGGTCATCCTGCGGATCAACCGGGCTGCCCGCTGCGTGCGCTGGGCCCCCAACGAG 389
                                         ************************************************************

i1_HQ_samplec9816d|c14866/f2p0/1185      AACAAGTTTGCTGTGGGCAGCGGCTCTCGTGTGATCTCCATCTGTTATTTCGAGCAGGAG 480
i1_HQ_samplec9816d|c43701/f2p0/1471      AACAAGTTTGCTGTGGGCAGCGGCTCTCGTGTGATCTCCATCTGTTATTTCGAGCAGGAG 449
NM_005720.4                              AACAAGTTTGCTGTGGGCAGCGGCTCTCGTGTGATCTCCATCTGTTATTTCGAGCAGGAG 449
                                         ************************************************************

i1_HQ_samplec9816d|c14866/f2p0/1185      AATGACTGGTGGGTTTGCAAGCACATCAAGAAGCCCATCCGCTCCACCGTCCTCAGCCTG 540
i1_HQ_samplec9816d|c43701/f2p0/1471      AATGACTGGTGGGTTTGCAAGCACATCAAGAAGCCCATCCGCTCCACCGTCCTCAGCCTG 509
NM_005720.4                              AATGACTGGTGGGTTTGCAAGCACATCAAGAAGCCCATCCGCTCCACCGTCCTCAGCCTG 509
                                         ************************************************************

i1_HQ_samplec9816d|c14866/f2p0/1185      GACTGGCACCCCAACAATGTGCTGCTGGCTGCCGGCTCCTGTGACTTCAAGTGTCGGATC 600
i1_HQ_samplec9816d|c43701/f2p0/1471      GACTGGCACCCCAACAATGTGCTGCTGGCTGCCGGCTCCTGTGACTTCAAGTGTCGGATC 569
NM_005720.4                              GACTGGCACCCCAACAATGTGCTGCTGGCTGCCGGCTCCTGTGACTTCAAGTGTCGGATC 569
                                         ************************************************************

i1_HQ_samplec9816d|c14866/f2p0/1185      TTTTCAGCCTACATCAAGGAGGTGGAGGAACGGCCGGCACCCACCCCGTGGGGCTCCAAG 660
i1_HQ_samplec9816d|c43701/f2p0/1471      TTTTCAGCCTACATCAAGGAGGTGGAGGAACGGCCGGCACCCACCCCGTGGGGCTCCAAG 629
NM_005720.4                              TTTTCAGCCTACATCAAGGAGGTGGAGGAACGGCCGGCACCCACCCCGTGGGGCTCCAAG 629
                                         ************************************************************

i1_HQ_samplec9816d|c14866/f2p0/1185      ATGCCCTTTGGGGAACTGATGTTCGAATCCAGCAGTAGCTGCGGCTGGGTACATGGCGTC 720
i1_HQ_samplec9816d|c43701/f2p0/1471      ATGCCCTTTGGGGAACTGATGTTCGAATCCAGCAGTAGCTGCGGCTGGGTACATGGCGTC 689
NM_005720.4                              ATGCCCTTTGGGGAACTGATGTTCGAATCCAGCAGTAGCTGCGGCTGGGTACATGGCGTC 689
                                         ************************************************************

i1_HQ_samplec9816d|c14866/f2p0/1185      TGTTTCTCAGCCAGCGGGAGCCGCGTGGCCTGGGTAAGCCACGACAGCACCGTCTGCCTG 780
i1_HQ_samplec9816d|c43701/f2p0/1471      TGTTTCTCAGCCAGCGGGAGCCGCGTGGCCTGGGTAAGCCACGACAGCACCGTCTGCCTG 749
NM_005720.4                              TGTTTCTCAGCCAGCGGGAGCCGCGTGGCCTGGGTAAGCCACGACAGCACCGTCTGCCTG 749
                                         ************************************************************
```

```
i1_HQ_samplec9816d|c14866/f2p0/1185      GCTGATGCCG-------------------------------------------------- 790
i1_HQ_samplec9816d|c43701/f2p0/1471      GCTGATGCCGACAAGAAGATGGCCGTCGCGACTCTGGCCTCTGAAACACTACCACTGCTG 809
NM_005720.4                              GCTGATGCCGACAAGAAGATGGCCGTCGCGACTCTGGCCTCTGAAACACTACCACTGCTG 809
                                         **********

i1_HQ_samplec9816d|c14866/f2p0/1185      ------------------------------------------------------------ 790
i1_HQ_samplec9816d|c43701/f2p0/1471      GCGCTGACCTTCATCACAGACAACAGCCTGGTGGCAGCGGGCCACGACTGCTTCCCGGTG 869
NM_005720.4                              GCGCTGACCTTCATCACAGACAACAGCCTGGTGGCAGCGGGCCACGACTGCTTCCCGGTG 869

i1_HQ_samplec9816d|c14866/f2p0/1185      ------------------------------------------------------------ 790
i1_HQ_samplec9816d|c43701/f2p0/1471      CTGTTCACCTATGACGCCGCCGCGGGGATGCTGAGCTTCGGCGGGCGGCTGGACGTTCCT 929
NM_005720.4                              CTGTTCACCTATGACGCCGCCGCGGGGATGCTGAGCTTCGGCGGGCGGCTGGACGTTCCT 929

i1_HQ_samplec9816d|c14866/f2p0/1185      ------------------------------------------------------------ 790
i1_HQ_samplec9816d|c43701/f2p0/1471      AAGCAGAGCTCGCAGCGTGGCTTGACGGCCCGCGAGCGCTTCCAGAACCTGGACAAGAAG 989
NM_005720.4                              AAGCAGAGCTCGCAGCGTGGCTTGACGGCCCGCGAGCGCTTCCAGAACCTGGACAAGAAG 989

i1_HQ_samplec9816d|c14866/f2p0/1185      ------------------------------------------------------------ 790
i1_HQ_samplec9816d|c43701/f2p0/1471      GCGAGCTCCGAGGGTGGCACGGCTGCGGGCGCGGGCCTAGACTCGCTGCACAAGAACAGC 1049
NM_005720.4                              GCGAGCTCCGAGGGTGGCACGGCTGCGGGCGCGGGCCTAGACTCGCTGCACAAGAACAGC 1049

i1_HQ_samplec9816d|c14866/f2p0/1185      ----------------------------ACAAGGCCAAGTGCTCGCAGTTCTGCACCACT 822
i1_HQ_samplec9816d|c43701/f2p0/1471      GTCAGCCAGATCTCGGTGCTCAGCGGCGGCAAGGCCAAGTGCTCGCAGTTCTGCACCACT 1109
NM_005720.4                              GTCAGCCAGATCTCGGTGCTCAGCGGCGGCAAGGCCAAGTGCTCGCAGTTCTGCACCACT 1109
                                                                     ********************************

i1_HQ_samplec9816d|c14866/f2p0/1185      GGCATGGATGGCGGCATGAGTATCTGGGATGTGAAGAGCTTGGAGTCAGCCTTGAAGGAC 882
i1_HQ_samplec9816d|c43701/f2p0/1471      GGCATGGATGGCGGCATGAGTATCTGGGATGTGAAGAGCTTGGAGTCAGCCTTGAAGGAC 1169
NM_005720.4                              GGCATGGATGGCGGCATGAGTATCTGGGATGTGAAGAGCTTGGAGTCAGCCTTGAAGGAC 1169
                                         ************************************************************

i1_HQ_samplec9816d|c14866/f2p0/1185      CTCAAGATCAAATGACCTGTGAGGAATATGTTGCCTTCATCCTAGCTGCTGGGGAAGCGG 942
i1_HQ_samplec9816d|c43701/f2p0/1471      CTCAAGATCAAATGACCTGTGAGGAATATGTTGCCTTCATCCTAGCTGCTGGGGAAGCGG 1229
NM_005720.4                              CTCAAGATCAAATGACCTGTGAGGAATATGTTGCCTTCATCCTAGCTGCTGGGGAAGCGG 1229
                                         ************************************************************

i1_HQ_samplec9816d|c14866/f2p0/1185      GGAGAGGGGTCAGGGAGGCTAATGGTTGCTTTGCTGAATGTTTCTGGGGTACCAATACGA 1002
i1_HQ_samplec9816d|c43701/f2p0/1471      GGAGAGGGGTCAGGGAGGCTAATGGTTGCTTTGCTGAATGTTTCTGGGGTACCAATACGA 1289
NM_005720.4                              GGAGAGGGGTCAGGGAGGCTAATGGTTGCTTTGCTGAATGTTTCTGGGGTACCAATACGA 1289
                                         ************************************************************

i1_HQ_samplec9816d|c14866/f2p0/1185      GTTCCCATAGGGGCTGCTCCCTCAAAAAGGGAGGGGACAGATGGGGAGCTTTTCTTACCT 1062
i1_HQ_samplec9816d|c43701/f2p0/1471      GTTCCCATAGGGGCTGCTCCCTCAAAAAGGGAGGGGACAGATGGGGAGCTTTTCTTACCT 1349
NM_005720.4                              GTTCCCATAGGGGCTGCTCCCTCAAAAAGGGAGGGGACAGATGGGGAGCTTTTCTTACCT 1349
                                         ************************************************************

i1_HQ_samplec9816d|c14866/f2p0/1185      ATTCAAGGAATACGTGCCTTTTTCTTAAATGCTTTCATTTATTGAAAAAAAAAAAAAATG 1122
i1_HQ_samplec9816d|c43701/f2p0/1471      ATTCAAGGAATACGTGCCTTTTTCTTAAATGCTTTCATTTATTGAAAAAAAAAAAAAATG 1409
NM_005720.4                              ATTCAAGGAATACGTGCCTTTTTCTTAAATGCTTTCATTTATTGAAAAAAAAAAAAAATG 1409
                                         ************************************************************

i1_HQ_samplec9816d|c14866/f2p0/1185      CCCCCAAAGCACTATGCTGGTCATGAACTGcttcaaaatgtggaggtaataaaatgcaac 1182
i1_HQ_samplec9816d|c43701/f2p0/1471      CCCCCAAAGCACTATGCTGGTcatgaactgcttcaaaatgtggaggtaataaaatgcaac 1469
NM_005720.4                              CCCCCAAAGCACTATGCTGGTCATGAACTGCTTCAAAATGTGGAGGTAATAAAATGCAAC 1469
                                         ************************************************************

i1_HQ_samplec9816d|c14866/f2p0/1185      tgt---------------------------------------       1185
i1_HQ_samplec9816d|c43701/f2p0/1471      tg----------------------------------------       1471
NM_005720.4                              TGTGTAAAAAAAAAAAAAAAAAAAAAAGTAATTATGGACATG       1511
                                         **
VIRT95841           ------------------------------------------------------------      0
VIRT91963           ------------------------------------------------------------      0
VIRT89465           MAYHSFLVEPISCHAWNKDRTQIAICPNNHEVHIYEKSGAKWTKVHELKEHNGQVTGIDW      60
NP_005711.1         MAYHSFLVEPISCHAWNKDRTQIAICPNNHEVHIYEKSGAKWTKVHELKEHNGQVTGIDW      60
VIRT99058           MAYHSFLVEPISCHAWNKDRTQIAICPNNHEVHIYEKSGAKWTKVHELKEHNGQVTGIDW      60

VIRT95841           ------------------------------------------------MKVSASSGS---      9
VIRT91963           MP-------------------------PS---------------------------MPVV      8
VIRT89465           APESNRIVTCGTDRNAYVWTLKGRTWKPTLVILRINRAARCVRWAPNENKFAVGSGSRVI      120
NP_005711.1         APESNRIVTCGTDRNAYVWTLKGRTWKPTLVILRINRAARCVRWAPNENKFAVGSGSRVI      120
VIRT99058           APESNRIVTCGTDRNAYVWTLKGRTWKPTLVILRINRAARCVRWAPNENKFAVGSGSRVI      120

VIRT95841           -----------------------------------------V-SEARVATAIFLS-----      22
VIRT91963           -----------QNCEHLALPPLS----------------------TEIWLTLFLCSESRP      35
VIRT89465           SICYFEQENDWWVCKHIKKPIRSTVLSLDWHPNNVLLAAGSCDFKCRIFSAYIKEVEERP      180
NP_005711.1         SICYFEQENDWWVCKHIKKPIRSTVLSLDWHPNNVLLAAGSCDFKCRIFSAYIKEVEERP      180
```

```
VIRT99058            SICYFEQENDWWVCKHIKKPIRSTVLSLDWHPNNVLLAAGSCDFKCRIFSAYIKEVEERP    180
                                                                   .:  : :

VIRT95841            -----------------------------ASARQTVLSWLTQATRLPLAEKQTP------    47
VIRT91963            APAA----VPPSELAFLSR---FWKRS----------RAVKPRCELCLGTSSRPP-----    73
VIRT89465            APTPWGSKMPFGELMFESSSSCGWVHGVCFSASGSRVAWVSHDSTVCLADADKKMAVATL    240
NP_005711.1          APTPWGSKMPFGELMFESSSSCGWVHGVCFSASGSRVAWVSHDSTVCLADADKKMAVATL    240
VIRT99058            APTPWGSKMPFGELMFESSSSCGWVHGVCFSASGSRVAWVSHDSTVCLADAD--------    232
                                                            :.    : *.  .

VIRT95841            ------------------------CTQPQLLLD---------SNISSP-----KGIL---    66
VIRT91963            -------------------------------------------KLSIPAAAS--------    82
VIRT89465            ASETLPLLALTFITDNSLVAAGHDCFPVLFTYDAAAGMLSFGGRLDVPKQSSQRGLTARE    300
NP_005711.1          ASETLPLLALTFITDNSLVAAGHDCFPVLFTYDAAAGMLSFGGRLDVPKQSSQRGLTARE    300
VIRT99058            ------------------------------------------------------------    232

VIRT95841            ----------EPHGVGAGRSSTSLM-----------------------------------    81
VIRT91963            ------------------------------------------------------------    82
VIRT89465            RFQNLDKKASSEGGTAAGAGLDSLHKNSVSQISVLSGGKAKCSQFCTTGMDGGMSIWDVK    360
NP_005711.1          RFQNLDKKASSEGGTAAGAGLDSLHKNSVSQISVLSGGKAKCSQFCTTGMDGGMSIWDVK    360
VIRT99058            --------------------------------------KAKCSQFCTTGMDGGMSIWDVK    254

VIRT95841            ------------ 81
VIRT91963            ------------ 82
VIRT89465            SLESALKDLKIK 372
NP_005711.1          SLESALKDLKIK 372
VIRT99058            SLESALKDLKIK 266
```

## WDR4

| Sequence | Header | AA Seq |
|---|---|---|
| Present | | |
| Absent | i1_HQ_samplec9816d\|c20457/f2p0/1218 | VIRT138907 |
| NCBI | NM_033661 | NP_387510.1 |
| | NM_001260476 | NP_001247405.1 |

>i1_HQ_samplec9816d|c20457/f2p0/1218
isoform=c20457;full_length_coverage=2;non_full_length_coverage=0;isoform_length=1218

CggacaggaagaggctctgggctggcacatgtgtatggcggtgaggcggggggtacatgGCGGGGCTCTGTGGGACTGGCGTTGTGCGG
GCAGACGTTGGTGGTGCGGGGCGGCAGCCGATTCCTGGCCACCTCCATAGCAAGCAGTGATGATGACAGCCTCTTCAT
CTATGACTGCAGTGCTGCAGAAAAGAAGTCACAAGAAAATAAAGGGGAGGACGCGCCCTTGGACCAGGGGAGCGGT
GCGATTCTGGCGTCCACCTTCTCCAACTCTGGCAGCTATTTTGCTTTAACCGATGACAGTAAGCGTCTGATTCTTTTCCGT
ACAAAACCATGGCAATGTCTGAGTGTCAGGTTTGTGAGCCGTATCTCCGTGGTGCCAACTCAGCCCGGGCTGCTTCTGT
CCTCCTCTGGGGATGGCACCCTGAGGCTCTGGGAGTACAGGAGCGGCCGCCAGCTGCACTGCTGTCACCTGGCCAGTC
TGCAGGAGCTGGTGGACCCCCAGGCCCCCCAGAAGTTTGCCGCGTCCAGCATTGCATTCTGGTGCCAGGAGAACTGCG
TGGCGCTCCTGTGCGACGGCACTTCTGTGGTCTACATCTTCCAGCTGGACGCCCGCAGACAGCAGTTGGTGTACAGGCA
GCAGCTGGCGTTCCAGCACCAAGTGTGGGACGTGGCTTTCGAGGAGACCCAGGGGCTGTGGGTGCTCCAGGACTGCC
AGGAAGCCCCCCTGGTGCTCTACAGGCCTGTGGGCGACCAGTGGCAGTCTGTTCCTGAGAGCACCGTGTTAAAGAAAG
TCTCTGGTGTTCTTCGTGGGAACTGGGCCATGCTGGAAGGCTCTGCCGGCGCAGACGCCAGCTTCAGCAGTCTCTACAA
GGCCACGTTCGACAACGTGACCTCCTACCTGAAGAAGAAAAGGAGAGACTGCAGCAGCAGCTAGAGAAGAAGCAGCG
GCGCCAGAGTCCCCCGCCTGGGCCCGACGGGCATGCCAAGAAGATGAGACCGGGGGAGGCGACGCTAAGTTGCTGAT
CGTGGCGGTCTGTTTCTGTCGACTGTGGACCACTTATGTGCGATCCGTGGACCACTTGCGTGCGATCTGTCGGCCGACG
ATGAGCTTGTTCGGATGTAGCTCCATCGTAAGTCGAGGAGCATCTGTGATTTGTCCTCTGCTTATGGGATATGTTTTTccg
ctactgagtctgtgtagtaaattttgtgact

>VIRT138907

MCMAVRRGGTWRGSVGLALCGQTLVVRGGSRFLATSIASSDDDSLFIYDCSAAEKKSQEN
KGEDAPLDQGSGAILASTFSNSGSYFALTDDSKRLILFRTKPWQCLSVRFVSRISVVPTQ
PGLLLSSSGDGTLRLWEYRSGRQLHCCHLASLQELVDPQAPQKFAASSIAFWCQENCVAL
LCDGTSVVYIFQLDARRQQLVYRQQLAFQHQVWDVAFEETQGLWVLQDCQEAPLVLYRPV
GDQWQSVPESTVLKKVSGVLRGNWAMLEGSAGADASFSSLYKATFDNVTSYLKKKRRDCS
SS

>NM_033661.4 Homo sapiens WD repeat domain 4 (WDR4), transcript variant 2, mRNA
CGGCGTCTGCGCCTGGCGTCACCGACCGGTGCGGACAGGAAGAGGCTCTGGGCTGGCACATGTGTATGGC
GGTGAGGCGGGCGGGTACATGGCGGGCTCTGTGGGACTGGCGTTGTGCGGGCAGACGTTGGTGGTGCGGG
GCGGCAGCCGATTCCTGGCCACCTCCATAGCAAGCAGTGATGATGACAGCCTCTTCATCTATGACTGCAG
TGCTGCAGAAAAGAAGTCACAAGAAAATAAAGGGGAGGACGCGCCCTTGGACCAGGGGAGCGGTGCGATT
CTGGCGTCCACCTTCTCCAAGTCTGGCAGCTATTTTGCTTTAACCGATGACAGTAAGCGTCTGATTCTTT
TCCGTACAAAACCATGGCAATGTCTGAGTGTCAGGACCGTGGCAAGGAGGTGTACAGCCCTGACTTTCAT
AGCCTCGGAGGAGAAGGTCTTGGTGGCCGACAAGTCTGGAGACGTCTACTCCTTTTCGGTGCTGGAGCCA
CACGGGTGTGGCCGTCTAGAGCTGGGGCACCTGTCTATGCTGTTAGATGTGGCTGTGAGTCCTGATGACC
GCTTCATCCTCACTGCCGACCGGGACGAGAAGATCCGAGTCAGCTGGGCCGCGGCGCCCCATAGCATCGA
GTCCTTCTGCTTGGGGCACACAGAGTTTGTGAGCCGTATCTCCGTGGTGCCAACTCAGCCCGGGCTGCTT
CTGTCCTCCTCTGGGGACGGCACCCTGAGGCTCTGGGAGTACAGGAGCGGCCGCCAGCTGCACTGCTGTC
ACCTGGCCAGTCTGCAGGAGCTGGTGGACCCCCAGGCCCCCCAGAAGTTTGCCGCGTCCAGGATTGCATT
CTGGTGCCAGGAGAACTGCGTGGCGCTCCTGTGCGACGGCACTCCTGTGGTCTACATCTTCCAGCTGGAC
GCCCGCAGACAGCAGTTGGTGTACAGGCAGCAGCTGGCGTTCCAGCACCAAGTGTGGGACGTGGCTTTCG
AGGAGACCCAGGGGCTGTGGGTGCTCCAGGACTGCCAGGAAGCCCCCCTGGTGCTCTACAGGCCTGTGGG
CGACCAGTGGCAGTCTGTTCCTGAGAGCACCGTGTTAAAGAAAGTCTCTGGTGTTCTTCGTGGGAACTGG
GCCATGCTGGAAGGCTCTGCCGGCGCAGACGCCAGCTTCAGCAGTCTCTACAAGGCCACGTTCGACAACG
TGACCTCCTACCTGAAGAAGAAAGAGGAGAGACTGCAGCAGCAGCTAGAGAAGAAGCAGCGGCGCCGGAG
TCCCCCGCCTGGGCCCGACGGGCATGCCAAGAAGATGAGACCGGGGGAGGCGACGCTAAGTTGCTGATCG
TGGCGGTCTGTTTCTGTCGACTGTGGACCACTTATGTGCGATCCGTGGACCACTTGCGTGCGATCTGTCG
GCCGACGATGAGCTTGTTCGGATGTAGCTCCATCGTAAGTCGAGGAGCATCTGTGATTTGTCCTCTGCTT
ATGGGATATGTTTTTCCGCTACTGAGTCTGTGTAGTAAATTTTTGACTAGGAACATGGTGAGATTTAAAA
AAAAAAAAAAAAAA

>NP_387510.1 tRNA (guanine-N(7)-)-methyltransferase non-catalytic subunit WDR4 isoform 1 [Homo sapiens]
MAGSVGLALCGQTLVVRGGSRFLATSIASSDDDSLFIYDCSAAEKKSQENKGEDAPLDQGSGAILASTFS
KSGSYFALTDDSKRLILFRTKPWQCLSVRTVARRCTALTFIASEEKVLVADKSGDVYSFSVLEPHGCGRL
ELGHLSMLLDVAVSPDDRFILTADRDEKIRVSWAAAPHSIESFCLGHTEFVSRISVVPTQPGLLLSSSGD
GTLRLWEYRSGRQLHCCHLASLQELVDPQAPQKFAASRIAFWCQENCVALLCDGTPVVYIFQLDARRQQL
VYRQQLAFQHQVWDVAFEETQGLWVLQDCQEAPLVLYRPVGDQWQSVPESTVLKKVSGVLRGNWAMLEGS
AGADASFSSLYKATFDNVTSYLKKKEERLQQQLEKKQRRRSPPPGPDGHAKKMRPGEATLSC

>NM_001260476.1 Homo sapiens WD repeat domain 4 (WDR4), transcript variant 5, mRNA
ATCTCTCGGCGTCTGCGCCTGGCGTCACCGACCGGTGCGGACAGGAAGAGGCTCTGGGCTGGCACATGTG
TATGGCGTGATGATGACAGCCTCTTCATCTATGACTGCAGTGCTGCAGAAAAGAAGTCACAAGAAAATAA
AGGGGAGGACGCGCCCTTGGACCAGGGGAGCGGTGCGATTCTGGCGTCCACCTTCTCCAAGTCTGGCAGC
TATTTTGCTTTAACCGATGACAGACCGTGGCAAGGAGGTGTACAGCCCTGACTTTCATAGCCTCGGAGGA
GAAGGTCTTGGTGGCCGACAAGTCTGGAGACGTCTACTCCTTTTCGGTGCTGGAGCCACACGGGTGTGGC
CGTCTAGAGCTGGGGCACCTGTCTATGCTGTTAGATGTGGCTGTGAGTCCTGATGACCGCTTCATCCTCA
CTGCCGACCGGGACGAGAAGATCCGAGTCAGCTGGGCCGCGGCGCCCCATAGCATCGAGTCCTTCTGCTT
GGGGCACACAGAGTTTGTGAGCCGTATCTCCGTGGTGCCAACTCAGCCCGGGCTGCTTCTGTCCTCCTCT
GGGGACGGCACCCTGAGGCTCTGGGAGTACAGGAGCGGCCGCCAGCTGCACTGCTGTCACCTGGCCAGTC
TGCAGGAGCTGGTGGACCCCCAGGCCCCCCAGAAGTTTGCCGCGTCCAGGATTGCATTCTGGTGCCAGGA
GAACTGCGTGGCGCTCCTGTGCGACGGCACTCCTGTGGTCTACATCTTCCAGCTGGACGCCCGCAGACAG
CAGTTGGTGTACAGGCAGCAGCTGGCGTTCCAGCACCAAGTGTGGGACGTGGCTTTCGAGGAGACCCAGG
GGCTGTGGGTGCTCCAGGACTGCCAGGAAGCCCCCCTGGTGCTCTACAGGCCTGTGGGCGACCAGTGGCA
GTCTGTTCCTGAGAGCACCGTGTTAAAGAAAGTCTCTGGTGTTCTTCGTGGGAACTGGGCCATGCTGGAA
GGCTCTGCCGGCGCAGACGCCAGCTTCAGCAGTCTCTACAAGGCCACGTTCGACAACGTGACCTCCTACC
TGAAGAAGAAAGAGGAGAGACTGCAGCAGCAGCTAGAGAAGAAGCAGCGGCGCCGGAGTCCCCCGCCTGG
GCCCGACGGGCATGCCAAGAAGATGAGACCGGGGGAGGCGACGCTAAGTTGCTGATCGTGGCGGTCAGTG
GTGTCTCTCACCTCAAATTAGGAAAAGCTTTTTAACTTGTTCCCCTGCATCCTGGCATGTCCCTTCAAGA
AGGAAAAGGTGACATCAGTTGTGACCAGCTCAGAGGATGGGGCTGTCCCCTGGGCTCTAGAAAGAACATT
CTGTGTCACCAGCACAGGCTCTTGCTGGGTGCCTGTGCCCCCTTTCTGGTTGAAGGGAAGCCAGACCAGT
GGAGTGGAAGCTCTTGAAGCACTTTCTTTTCATGTCGGAGGCTGCGTGTGAGCCGGTGGTGGCGGCAGCT
GTCTGTGTTTCTCCTGTTCTCATACCGCCCTTCGTGTCCGCTGCATCTTCCTCTAGGAGCATCTGTGGGC
TGACGTTTCTGGCATGGTGGATGTTTTCATGTTGTTCCGCCGTCTCAGCATCCAGGTGACGTGCTGTCCC
GCAGTAGCTGCTCGCCACACAGGCTGTTGAAACTCAGCACGAATGCTTGAGCCTGCCCCAGCCCCAGCCG
TAGCCCTTGTGCTCAGGAGCTGCTCCTGGAGAGTGGCTCCCTTGTGGGAAGTGAGAAACACGTTTCGGAG

```
TCGCAGGGAGCTCTGTCTGGCATCGGTGGCACAGATGGTGAACTTGTTTCAGGAAGTGTGAGCACGGGCC
TGGCTGGCACCCGGTTTACAGGGAGGTGATTGAGGACGTCGTTATGATTTGATTTGACCTTGGCACACAC
TGCAGAGCCACAGGGATGTTGCTGAATTTCAATCACCGATCAGCAAGGATGAACCTTCTCAACTTCCTAG
GAATGTTTAGCAGCAACCTAATATACCAGGCAAAACTCAAAAAAAAAAAAAAAAAA
```

```
>NP_001247405.1 tRNA (guanine-N(7)-)-methyltransferase non-catalytic subunit WDR4 isoform 3 [Homo
sapiens]
MLLDVAVSPDDRFILTADRDEKIRVSWAAAPHSIESFCLGHTEFVSRISVVPTQPGLLLSSSGDGTLRLW
EYRSGRQLHCCHLASLQELVDPQAPQKFAASRIAFWCQENCVALLCDGTPVVYIFQLDARRQQLVYRQQL
AFQHQVWDVAFEETQGLWVLQDCQEAPLVLYRPVGDQWQSVPESTVLKKVSGVLRGNWAMLEGSAGADAS
FSSLYKATFDNVTSYLKKKEERLQQQLEKKQRRRSPPPGPDGHAKKMRPGEATLSC
```

```
NM_001260476.1                           ------------------------------------------------------------ 0
i1_HQ_samplec9816d|c20457/f2p0/1218      -------------------------------Cggacaggaagaggctctgggctggcaca 29
NM_033661.4                              CGGCGTCTGCGCCTGGCGTCACCGACCGGTGCGGACAGGAAGAGGCTCTGGGCTGGCACA 60

NM_001260476.1                           -------------------------------------ATCTCTCGGCGT----CTGCGCC 19
i1_HQ_samplec9816d|c20457/f2p0/1218      tgtgtatggcggtgaggcgggggggtacatgGCGGGGCTCTGTGGGACTGGCGTTGTGCG 89
NM_033661.4                              TGTGTATGGCGGTGAGGCGGGCGGGTACATGGC-GGGCTCTGTGGGACTGGCGTTGTGCG 119
                                                                               *** * **  *     ** **

NM_001260476.1                           TGGCGTCACCGACCGGTGCGGACAGGAAGAGGCTCTGGGCTGGCACATGTGTATGGCGTG 79
i1_HQ_samplec9816d|c20457/f2p0/1218      GGCAGACGTTGGTGGTGCGGGGCGGCAGCCGATTCCTGGCCACCTCCATAGCAAGCAGTG 149
NM_033661.4                              GGCAGACGTTGGTGGTGCGGGGCGGCAGCCGATTCCTGGCCACCTCCATAGCAAGCAGTG 179
                                          *  * *   *   *    ** * * *   *  **  ***   * *    * * *  ***

NM_001260476.1                           ATGATGACAGCCTCTTCATCTATGACTGCAGTGCTGCAGAAAAGAAGTCACAAGAAAATA 139
i1_HQ_samplec9816d|c20457/f2p0/1218      ATGATGACAGCCTCTTCATCTATGACTGCAGTGCTGCAGAAAAGAAGTCACAAGAAAATA 209
NM_033661.4                              ATGATGACAGCCTCTTCATCTATGACTGCAGTGCTGCAGAAAAGAAGTCACAAGAAAATA 239
                                         ************************************************************

NM_001260476.1                           AAGGGGAGGACGCGCCCTTGGACCAGGGGAGCGGTGCGATTCTGGCGTCCACCTTCTCCA 199
i1_HQ_samplec9816d|c20457/f2p0/1218      AAGGGGAGGACGCGCCCTTGGACCAGGGGAGCGGTGCGATTCTGGCGTCCACCTTCTCCA 269
NM_033661.4                              AAGGGGAGGACGCGCCCTTGGACCAGGGGAGCGGTGCGATTCTGGCGTCCACCTTCTCCA 299
                                         ************************************************************

NM_001260476.1                           AGTCTGGCAGCTATTTTGCTTTAACCGATGACAGA------------------------- 234
i1_HQ_samplec9816d|c20457/f2p0/1218      ACTCTGGCAGCTATTTTGCTTTAACCGATGACAGTAAGCGTCTGATTCTTTTCCGTACAA 329
NM_033661.4                              AGTCTGGCAGCTATTTTGCTTTAACCGATGACAGTAAGCGTCTGATTCTTTTCCGTACAA 359
                                         * ********************************

NM_001260476.1                           ---------------------------CCGTGGCAAGGAGGTGTACAGCCCTGACTTTCA 267
i1_HQ_samplec9816d|c20457/f2p0/1218      AACCATGGCAATGTCTGAGTGTCAGGTT-------------------------------- 357
NM_033661.4                              AACCATGGCAATGTCTGAGTGTCAGGACCGTGGCAAGGAGGTGTACAGCCCTGACTTTCA 419

NM_001260476.1                           TAGCCTCGGAGGAGAAGGTCTTGGTGGCCGACAAGTCTGGAGACGTCTACTCCTTTTCGG 327
i1_HQ_samplec9816d|c20457/f2p0/1218      ------------------------------------------------------------ 357
NM_033661.4                              TAGCCTCGGAGGAGAAGGTCTTGGTGGCCGACAAGTCTGGAGACGTCTACTCCTTTTCGG 479

NM_001260476.1                           TGCTGGAGCCACACGGGTGTGGCCGTCTAGAGCTGGGGCACCTGTCTATGCTGTTAGATG 387
i1_HQ_samplec9816d|c20457/f2p0/1218      ------------------------------------------------------------ 357
NM_033661.4                              TGCTGGAGCCACACGGGTGTGGCCGTCTAGAGCTGGGGCACCTGTCTATGCTGTTAGATG 539

NM_001260476.1                           TGGCTGTGAGTCCTGATGACCGCTTCATCCTCACTGCCGACCGGGACGAGAAGATCCGAG 447
i1_HQ_samplec9816d|c20457/f2p0/1218      ------------------------------------------------------------ 357
NM_033661.4                              TGGCTGTGAGTCCTGATGACCGCTTCATCCTCACTGCCGACCGGGACGAGAAGATCCGAG 599

NM_001260476.1                           TCAGCTGGGCCGCGGCGCCCCATAGCATCGAGTCCTTCTGCTTGGGGCACACAGAGTTTG 507
i1_HQ_samplec9816d|c20457/f2p0/1218      ----------------------------------------------------------TG 359
NM_033661.4                              TCAGCTGGGCCGCGGCGCCCCATAGCATCGAGTCCTTCTGCTTGGGGCACACAGAGTTTG 659
                                                                                                   **

NM_001260476.1                           TGAGCCGTATCTCCGTGGTGCCAACTCAGCCCGGGCTGCTTCTGTCCTCCTCTGGGGACG 567
i1_HQ_samplec9816d|c20457/f2p0/1218      TGAGCCGTATCTCCGTGGTGCCAACTCAGCCCGGGCTGCTTCTGTCCTCCTCTGGGGATG 419
NM_033661.4                              TGAGCCGTATCTCCGTGGTGCCAACTCAGCCCGGGCTGCTTCTGTCCTCCTCTGGGGACG 719
                                         ********************************************************** *

NM_001260476.1                           GCACCCTGAGGCTCTGGGAGTACAGGAGCGGCCGCCAGCTGCACTGCTGTCACCTGGCCA 627
i1_HQ_samplec9816d|c20457/f2p0/1218      GCACCCTGAGGCTCTGGGAGTACAGGAGCGGCCGCCAGCTGCACTGCTGTCACCTGGCCA 479
NM_033661.4                              GCACCCTGAGGCTCTGGGAGTACAGGAGCGGCCGCCAGCTGCACTGCTGTCACCTGGCCA 779
                                         ************************************************************
```

```
NM_001260476.1                          GTCTGCAGGAGCTGGTGGACCCCCAGGCCCCCCAGAAGTTTGCCGCGTCCAGGATTGCAT 687
i1_HQ_samplec9816d|c20457/f2p0/1218     GTCTGCAGGAGCTGGTGGACCCCCAGGCCCCCCAGAAGTTTGCCGCGTCCAGCATTGCAT 539
NM_033661.4                             GTCTGCAGGAGCTGGTGGACCCCCAGGCCCCCCAGAAGTTTGCCGCGTCCAGGATTGCAT 839
                                        **************************************************** *******

NM_001260476.1                          TCTGGTGCCAGGAGAACTGCGTGGCGCTCCTGTGCGACGGCACTCCTGTGGTCTACATCT 747
i1_HQ_samplec9816d|c20457/f2p0/1218     TCTGGTGCCAGGAGAACTGCGTGGCGCTCCTGTGCGACGGCACTTCTGTGGTCTACATCT 599
NM_033661.4                             TCTGGTGCCAGGAGAACTGCGTGGCGCTCCTGTGCGACGGCACTCCTGTGGTCTACATCT 899
                                        ******************************************** ***************

NM_001260476.1                          TCCAGCTGGACGCCCGCAGACAGCAGTTGGTGTACAGGCAGCAGCTGGCGTTCCAGCACC 807
i1_HQ_samplec9816d|c20457/f2p0/1218     TCCAGCTGGACGCCCGCAGACAGCAGTTGGTGTACAGGCAGCAGCTGGCGTTCCAGCACC 659
NM_033661.4                             TCCAGCTGGACGCCCGCAGACAGCAGTTGGTGTACAGGCAGCAGCTGGCGTTCCAGCACC 959
                                        ************************************************************

NM_001260476.1                          AAGTGTGGGACGTGGCTTTCGAGGAGACCCAGGGGCTGTGGGTGCTCCAGGACTGCCAGG 867
i1_HQ_samplec9816d|c20457/f2p0/1218     AAGTGTGGGACGTGGCTTTCGAGGAGACCCAGGGGCTGTGGGTGCTCCAGGACTGCCAGG 719
NM_033661.4                             AAGTGTGGGACGTGGCTTTCGAGGAGACCCAGGGGCTGTGGGTGCTCCAGGACTGCCAGG 1019
                                        ************************************************************

NM_001260476.1                          AAGCCCCCCTGGTGCTCTACAGGCCTGTGGGCGACCAGTGGCAGTCTGTTCCTGAGAGCA 927
i1_HQ_samplec9816d|c20457/f2p0/1218     AAGCCCCCCTGGTGCTCTACAGGCCTGTGGGCGACCAGTGGCAGTCTGTTCCTGAGAGCA 779
NM_033661.4                             AAGCCCCCCTGGTGCTCTACAGGCCTGTGGGCGACCAGTGGCAGTCTGTTCCTGAGAGCA 1079
                                        ************************************************************

NM_001260476.1                          CCGTGTTAAAGAAAGTCTCTGGTGTTCTTCGTGGGAACTGGGCCATGCTGGAAGGCTCTG 987
i1_HQ_samplec9816d|c20457/f2p0/1218     CCGTGTTAAAGAAAGTCTCTGGTGTTCTTCGTGGGAACTGGGCCATGCTGGAAGGCTCTG 839
NM_033661.4                             CCGTGTTAAAGAAAGTCTCTGGTGTTCTTCGTGGGAACTGGGCCATGCTGGAAGGCTCTG 1139
                                        ************************************************************

NM_001260476.1                          CCGGCGCAGACGCCAGCTTCAGCAGTCTCTACAAGGCCACGTTCGACAACGTGACCTCCT 1047
i1_HQ_samplec9816d|c20457/f2p0/1218     CCGGCGCAGACGCCAGCTTCAGCAGTCTCTACAAGGCCACGTTCGACAACGTGACCTCCT 899
NM_033661.4                             CCGGCGCAGACGCCAGCTTCAGCAGTCTCTACAAGGCCACGTTCGACAACGTGACCTCCT 1199
                                        ************************************************************

NM_001260476.1                          ACCTGAAGAAGAAAGAGGAGAGACTGCAGCAGCAGCTAGAGAAGAAGCAGCGGCGCCGGA 1107
i1_HQ_samplec9816d|c20457/f2p0/1218     ACCTGAAGAAGAA-AAGGAGAGACTGCAGCAGCAGCTAGAGAAGAAGCAGCGGCGCCAGA 958
NM_033661.4                             ACCTGAAGAAGAAAGAGGAGAGACTGCAGCAGCAGCTAGAGAAGAAGCAGCGGCGCCGGA 1259
                                        *************  ****************************************** **

NM_001260476.1                          GTCCCCCGCCTGGGCCCGACGGGCATGCCAAGAAGATGAGACCGGGGGAGGCGACGCTAA 1167
i1_HQ_samplec9816d|c20457/f2p0/1218     GTCCCCCGCCTGGGCCCGACGGGCATGCCAAGAAGATGAGACCGGGGGAGGCGACGCTAA 1018
NM_033661.4                             GTCCCCCGCCTGGGCCCGACGGGCATGCCAAGAAGATGAGACCGGGGGAGGCGACGCTAA 1319
                                        ************************************************************

NM_001260476.1                          GTTGCTGATCGTGGCGGTCAGTGGTGTCTCTCACCTCAAATTAGGAAAAGCTTTTTAACT 1227
i1_HQ_samplec9816d|c20457/f2p0/1218     GTTGCTGATCGTGGCGGTCTGTTTCTGTCGACTGTGGACCACTTATGT---GCGATCCGT 1075
NM_033661.4                             GTTGCTGATCGTGGCGGTCTGTTTCTGTCGACTGTGGACCACTTATGT---GCGATCCGT 1376
                                        ******************* **         *     *                 *   *

NM_001260476.1                          TGTTCCCCTGCATCCTGGCATGTCCCTTCAAGAAGGAAAAGGTGACATCAGTTGTGACCA 1287
i1_HQ_samplec9816d|c20457/f2p0/1218     GGACCACTTGCGTG-CGATCTGTCGGCCGACGATGAGCT-----TGTTCGGATGTAGCTC 1129
NM_033661.4                             GGACCACTTGCGTG-CGATCTGTCGGCCGACGATGAGCT-----TGTTCGGATGTAGCTC 1430
                                         *  * * *** *   *   ****     * ** *            ** * ***  *

NM_001260476.1                          GCTCAGAGGA---TGGG--GCTGTCCCCTGGGCTCTAGAAAGAACATTCTGTGTCACCAG 1342
i1_HQ_samplec9816d|c20457/f2p0/1218     CATCGTAAGTCGAGGAGCATCTGTGATTTGTCCTCTGCTTATGGGA-TATGTTTTTccgc 1188
NM_033661.4                             CATCGTAAGTCGAGGAGCATCTGTGATTTGTCCTCTGCTTATGGGA-TATGTTTTTCCGC 1489
                                          **  * *     * *   ****    **  ****    *    * * *** *  **

NM_001260476.1                          CACAGGCTCTTGCTGGGTGC-------CTGTGCCCCCTTTCTGGTTGAAGGGAAGCCAGA 1395
i1_HQ_samplec9816d|c20457/f2p0/1218     tactgagtctgtgtagtaaattttgtgact------------------------------ 1218
NM_033661.4                             TACTGAGTCTGTGTAGTAAATTTTTGACTAGGAACATGGTGAGATTTAAAAAAAAAAAAA 1549
                                         ** *  ***   * *

NM_001260476.1                          CCAGTGGAGTGGAAGCTCTTGAAGCACTTTCTTTTCATGTCGGAGGCTGCGTGTGAGCCG 1455
i1_HQ_samplec9816d|c20457/f2p0/1218     ------------------------------------------------------------ 1218
NM_033661.4                             AAAAA------------------------------------------------------- 1554

NM_001260476.1                          GTGGTGGCGGCAGCTGTCTGTGTTTCTCCTGTTCTCATACCGCCCTTCGTGTCCGCTGCA 1515
i1_HQ_samplec9816d|c20457/f2p0/1218     ------------------------------------------------------------ 1218
NM_033661.4                             ------------------------------------------------------------ 1554

NM_001260476.1                          TCTTCCTCTAGGAGCATCTGTGGGCTGACGTTTCTGGCATGGTGGATGTTTTCATGTTGT 1575
i1_HQ_samplec9816d|c20457/f2p0/1218     ------------------------------------------------------------ 1218
NM_033661.4                             ------------------------------------------------------------ 1554

NM_001260476.1                          TCCGCCGTCTCAGCATCCAGGTGACGTGCTGTCCCGCAGTAGCTGCTCGCCACACAGGCT 1635
i1_HQ_samplec9816d|c20457/f2p0/1218     ------------------------------------------------------------ 1218
```

```
NM_033661.4                                                      ------------------------------------------------------------ 1554

NM_001260476.1                                                   GTTGAAACTCAGCACGAATGCTTGAGCCTGCCCCAGCCCCAGCCGTAGCCCTTGTGCTCA 1695
i1_HQ_samplec9816d|c20457/f2p0/1218                              ------------------------------------------------------------ 1218
NM_033661.4                                                      ------------------------------------------------------------ 1554

NM_001260476.1                                                   GGAGCTGCTCCTGGAGAGTGGCTCCCTTGTGGGAAGTGAGAAACACGTTTCGGAGTCGCA 1755
i1_HQ_samplec9816d|c20457/f2p0/1218                              ------------------------------------------------------------ 1218
NM_033661.4                                                      ------------------------------------------------------------ 1554

NM_001260476.1                                                   GGGAGCTCTGTCTGGCATCGGTGGCACAGATGGTGAACTTGTTTCAGGAAGTGTGAGCAC 1815
i1_HQ_samplec9816d|c20457/f2p0/1218                              ------------------------------------------------------------ 1218
NM_033661.4                                                      ------------------------------------------------------------ 1554

NM_001260476.1                                                   GGGCCTGGCTGGCACCCGGTTTACAGGGAGGTGATTGAGGACGTCGTTATGATTTGATTT 1875
i1_HQ_samplec9816d|c20457/f2p0/1218                              ------------------------------------------------------------ 1218
NM_033661.4                                                      ------------------------------------------------------------ 1554

NM_001260476.1                                                   GACCTTGGCACACACTGCAGAGCCACAGGGATGTTGCTGAATTTCAATCACCGATCAGCA 1935
i1_HQ_samplec9816d|c20457/f2p0/1218                              ------------------------------------------------------------ 1218
NM_033661.4                                                      ------------------------------------------------------------ 1554

NM_001260476.1                                                   AGGATGAACCTTCTCAACTTCCTAGGAATGTTTAGCAGCAACCTAATATACCAGGCAAAA 1995
i1_HQ_samplec9816d|c20457/f2p0/1218                              ------------------------------------------------------------ 1218
NM_033661.4                                                      ------------------------------------------------------------ 1554

NM_001260476.1                                                   CTCAAAAAAAAAAAAAAAAAA         2016
i1_HQ_samplec9816d|c20457/f2p0/1218                              ---------------------         1218
NM_033661.4                                                      ---------------------         1554
```

```
VIRT138907          MCMAVRRGGTWRGSVGLALCGQTLVVRGGSRFLATSIASSDDDSLFIYDCSAAEKKSQEN  60
NP_387510.1         ----------MAGSVGLALCGQTLVVRGGSRFLATSIASSDDDSLFIYDCSAAEKKSQEN  50
NP_001247405.1      ------------------------------------------------------------  0

VIRT138907          KGEDAPLDQGSGAILASTFSNSGSYFALTDDSKRLILFRTKPWQCLSVR-----------  109
NP_387510.1         KGEDAPLDQGSGAILASTFSKSGSYFALTDDSKRLILFRTKPWQCLSVRTVARRCTALTF  110
NP_001247405.1      ------------------------------------------------------------  0

VIRT138907          ------------------------------------------------------------  109
NP_387510.1         IASEEKVLVADKSGDVYSFSVLEPHGCGRLELGHLSMLLDVAVSPDDRFILTADRDEKIR  170
NP_001247405.1      ------------------------------------MLLDVAVSPDDRFILTADRDEKIR  24

VIRT138907          -------------------FVSRISVVPTQPGLLLSSSGDGTLRLWEYRSGRQLHCCHLA  150
NP_387510.1         VSWAAAPHSIESFCLGHTEFVSRISVVPTQPGLLLSSSGDGTLRLWEYRSGRQLHCCHLA  230
NP_001247405.1      VSWAAAPHSIESFCLGHTEFVSRISVVPTQPGLLLSSSGDGTLRLWEYRSGRQLHCCHLA  84
                                       *****************************************

VIRT138907          SLQELVDPQAPQKFAASSIAFWCQENCVALLCDGTSVVYIFQLDARRQQLVYRQQLAFQH  210
NP_387510.1         SLQELVDPQAPQKFAASRIAFWCQENCVALLCDGTPVVYIFQLDARRQQLVYRQQLAFQH  290
NP_001247405.1      SLQELVDPQAPQKFAASRIAFWCQENCVALLCDGTPVVYIFQLDARRQQLVYRQQLAFQH  144
                    ***************** ***************** ************************

VIRT138907          QVWDVAFEETQGLWVLQDCQEAPLVLYRPVGDQWQSVPESTVLKKVSGVLRGNWAMLEGS  270
NP_387510.1         QVWDVAFEETQGLWVLQDCQEAPLVLYRPVGDQWQSVPESTVLKKVSGVLRGNWAMLEGS  350
NP_001247405.1      QVWDVAFEETQGLWVLQDCQEAPLVLYRPVGDQWQSVPESTVLKKVSGVLRGNWAMLEGS  204
                    ************************************************************

VIRT138907          AGADASFSSLYKATFDNVTSYLKKKRRDCSSS----------------------------  302
NP_387510.1         AGADASFSSLYKATFDNVTSYLKKKEERLQQQLEKKQRRRSPPPGPDGHAKKMRPGEATL  410
NP_001247405.1      AGADASFSSLYKATFDNVTSYLKKKEERLQQQLEKKQRRRSPPPGPDGHAKKMRPGEATL  264
                    *************************

VIRT138907          --         302
NP_387510.1         SC         412
NP_001247405.1      SC         266
```

## SUMO3

| Sequence | Header | AA Seq | |
|---|---|---|---|
| Present | | | |
| Absent | i0_HQ_samplec9816d\|c13953/f3p5/509 | >VIRT175354 | >VIRT176199 |
| NCBI | NM_006936 | NP_008867.2 | |
| | NM_001286416 | | |

>i0_HQ_samplec9816d|c13953/f3p5/509
isoform=c13953;full_length_coverage=3;non_full_length_coverage=5;isoform_length=509

AgttgcGGCGGGAGAGCGGCGGGGCCGAGAGCGTGACTCGCCCGCTCCGCGCTGCTTCCCCCGCGCCGCCTCCCCGCGC
CGCTCGGCGCAGCCATGTCCGAGGAGAAGCCCAAGGGCTTGTCAATGAGGCAGATCAGATTCAGGTTCGACGGGCAG
CCAATCAATGAAACTGACACTCCAGCACAGCTGGAGATGGAGGACGAGGACACCATCGACGTGTTCCAGCAGCAGACG
GGAGGTGTGCCGGAGAGCAGCCTGGCAGGGCACAGTTTCTAGAGGGCCCGTCCCCAGCCCGGGCCGTCCATCCTCGC
ATTGCTGTTGAATGGTGAGCACGTGACCATGCCGACCACAAAGGTGTCTGCGGAAACTCGAGGACATTCACCACGATG
ATTTTCCTCTCTTTGATGTACTTCAAGTGCAACTCAAAACTATATCTGCAGGGATGAATCTGTAACTTAAATTGGGCCAAT
CAGAATTGTTATCTTTGTTCAGGTAAAATGAGTTGCAAG

>VIRT175354

MSEEKPKGLSMRQIRFRFDGQPINETDTPAQLEMEDEDTIDVFQQQTGGVPESSLAGHSF

>VIRT176199

MKLTLQHSWRWRTRTPSTCSSSRREVCRRAAWQGTVSRGPVPSPGRPSSHCC

```
>NM_006936.2 Homo sapiens small ubiquitin-like modifier 3 (SUMO3), transcript variant 1, mRNA
AGCCACGTGACCCCCGTGCCGGCCAACGGGTGCGCCGGGATTTGGGGGATAAAGCGCGGCCCCGCGCACA
GTTGCGGCGGGAGAGCGGCGGGGCCGAGAGCGTGACTCGCCCGCTCCGCGCTGCTTCCCCCGCGCCGCCT
CCCCGCGCCGCTCGCGCAGCCATGTCCGAGGAGAAGCCCAAGGAGGGTGTGAAGACAGAGAATGACCACA
TCAACCTGAAGGTGGCCGGGCAGGACGGCTCCGTGGTGCAGTTCAAGATCAAGAGGCACACGCCGCTGAG
CAAGCTGATGAAGGCCTACTGCGAGAGGCAGGGCTTGTCAATGAGGCAGATCAGATTCAGGTTCGACGGG
CAGCCAATCAATGAAACTGACACTCCAGCACAGCTGGAGATGGAGGACGAGGACACCATCGACGTGTTCC
AGCAGCAGACGGGAGGTGTGCCGGAGAGCAGCCTGGCAGGGCACAGTTTCTAGAGGGCCCGTCCCCAGCC
CGGGCCGTCCATCCTCGCATTGCTGTTGAATGGTGAGCACGTGACCATGCCGACCACAAAGGTGTCTGCG
GAAACTCGAGGACATTCACCACGATGATTTTCCTCTCTTTGATGTACTTCAAGTGCAACTCAAAACTATA
TCTGCAGGGATGAATCTGTAACTTAAATTGGGCCAATCAGAATTGTTATCTTTGTTCAGGTAAAATGAGT
TGCAAGATATTGTGGGTACTTTTGTGTGCTCATTTGTGTTTTCCCCCCCTCCTACAACATTTTTTTAACC
CCAAAATTATAGCCTGAATGTTCGCTTTTAGTCTGGCCAGGGATCTGACTCCTGAGTTGGTTGCCTCTCC
CCTGCTCACTCCAGTCACATAGAGAATTGGTGTTTCCCGCAGTGGGGATGCAGCTGTTGGACAGGTATTG
GGGGCAAGGTTGGTAGGGAGGACAGACTGTCACTTGCTGTTACAGGCACAGGTGATTAAAATGCTAAATA
TTGCAAATTTAAGCTTTGTCAGTATATGGAAAAGTTGAAGGGAAAATACTGGAATGCTTCTTCAAAGGTT
AAAAAATAACCGAGTCTTTTGGTAATTTGACCCCACGTGCTCTCTGGCCCTCAAGCATGTAACCTCGGGG
TCTGAGGCCCAGGACCCACCCCCCTGCCACCCCTCCCACCCCACTCCCTGCTCAGTACCTGGCGTTGGTA
CACAGGCAAGGATTGGCACAACCAAAATTGGCCTTTTTCTCCCTCTTAATATTGAAGAAATTCCCACATT
TCTCATTTGGTAATGGTGTTGTGGCCTCAGATTTCTTCTAGTATTTGCTTCTGATGAATGATTATGGTCT
ATACATAAAAAAGTAAGACTAAGTATTGCTGAATTTGCAGTTATGTTGTCGTGTATAAGAGCTACTTCCA
AGTGTGGTTACAAATGAACCCATGGAATGATGACTTCATGTTCTTCTCGTGGGTTTGTGCCGTGCTGCTT
TCCAAATAGGTATTGAATTTATGCATTAGTCTGGTGATTTCAGTTCTGTGAAATATTTTGGATCTATAC
CAATTAAACATTTTCATAGTTCTGCCTATTGTCCTTCCCTGAGGCTCCATTGCTGCTTGGTGGCCATTCT
CTGCCTTTTTACAGTCACCTGAACAATGACCCATCATCTCTTGCTTGCTTGAAATCTTGCTGAAATGTTC
TCATTTCCTGTTTGCTGTATGGGCTCGGGTGGATGTTTGTTGGCTCTGTTGTGTTTATTCACCAATTTG
TACATTATTTGTTGTCCTTTACTACTGTAAACAGTAAATATAGTTTGGTATTCTGTCAAAAAAAAAAAAA
AAAAAAAAAAA
```

```
>NP_008867.2 small ubiquitin-related modifier 3 isoform 1 precursor [Homo sapiens]
MSEEKPKEGVKTENDHINLKVAGQDGSVVQFKIKRHTPLSKLMKAYCERQGLSMRQIRFRFDGQPINETD
TPAQLEMEDEDTIDVFQQQTGGVPESSLAGHSF
```

```
>NM_001286416.1 Homo sapiens small ubiquitin-like modifier 3 (SUMO3), transcript variant 2, mRNA
AGCCACGTGACCCCCGTGCCGGCCAACGGGTGCGCCGGGATTTGGGGGATAAAGCGCGGCCCCGCGCACA
GTTGCGGCGGGAGAGCGGCGGGGCCGAGAGCGTGACTCGCCCGCTCCGCGCTGCTTCCCCCGCGCCGCCT
CCCCGCGCCGCTCGCGCAGCCATGTCCGAGGAGAAGCCCAAGGAGGGTGTGAAGACAGAGAATGACCACA
TCAACCTGAAGGTGGCCGGGCAGGACGGCTCCGTGGTGCAGTTCAAGATCAAGAGGCACACGCCGCTGAG
CAAGCTGATGAAGGCCTACTGCGAGAGGCAGGTGCGGCACCTTGCTCCCCCGCAGAGCCTCCCCGTGTGC
GCACTGGTCCTGTGCGTTCCAGGCATCCCCAGAGCACGAGCGTCTCGGGGCTGGACCCAGATGCAGCTGC
CCGAGGGCTTGTCAATGAGGCAGATCAGATTCAGGTTCGACGGGCAGCCAATCAATGAAACTGACACTCC
AGCACAGCTGGAGATGGAGGACGAGGACACCATCGACGTGTTCCAGCAGCAGACGGGAGGTGTGCCGGAG
AGCAGCCTGGCAGGGCACAGTTTCTAGAGGGCCCGTCCCCAGCCCGGGCCGTCCATCCTCGCATTGCTGT
TGAATGGTGAGCACGTGACCATGCCGACCACAAAGGTGTCTGCGGAAACTCGAGGACATTCACCACGATG
ATTTTCCTCTCTTTGATGTACTTCAAGTGCAACTCAAAACTATATCTGCAGGGATGAATCTGTAACTTAA
ATTGGGCCAATCAGAATTGTTATCTTTGTTCAGGTAAAATGAGTTGCAAGATATTGTGGGTACTTTTGTG
TGCTCATTTGTGTTTTCCCCCCCTCCTACAACATTTTTTTAACCCCAAAATTATAGCCTGAATGTTCGCT
TTTAGTCTGGCCAGGGATCTGACTCCTGAGTTGGTTGCCTCTCCCCTGCTCACTCCAGTCACATAGAGAA
TTGGTGTTTCCCGCAGTGGGGATGCAGCTGTTGGACAGGTATTGGGGGCAAGGTTGGTAGGGAGGACAGA
CTGTCACTTGCTGTTACAGGCACAGGTGATTAAAATGCTAAATATTGCAAATTTAAGCTTTGTCAGTATA
TGGAAAAGTTGAAGGGAAAATACTGGAATGCTTCTTCAAAGGTTAAAAAATAACCGAGTCTTTTGGTAAT
TTGACCCCACGTGCTCTCTGGCCCTCAAGCATGTAACCTCGGGGTCTGAGGCCCAGGACCCACCCCCCTG
CCACCCCTCCCACCCCACTCCCTGCTCAGTACCTGGCGTTGGTACACAGGCAAGGATTGGCACAACCAAA
ATTGGCCTTTTTCTCCCTCTTAATATTGAAGAAATTCCCACATTTCTCATTTGGTAATGGTGTTGTGGCC
TCAGATTTCTTCTAGTATTTGCTTCTGATGAATGATTATGGTCTATACATAAAAAAGTAAGACTAAGTAT
TGCTGAATTTGCAGTTATGTTGTCGTGTATAAGAGCTACTTCCAAGTGTGGTTACAAATGAACCCATGGA
ATGATGACTTCATGTTCTTCTCGTGGGTTTGTGCCGTGCTGCTTTCCAAATAGGTATTGAATTTATGCAT
TAGTCTGGTGATTTCAGTTCTGTGAAATATTTTGGGATCTATACCAATTAAACATTTTCATAGTTCTGCC
TATTGTCCTTCCCTGAGGCTCCATTGCTGCTTGGTGGCCATTCTCTGCCTTTTTACAGTCACCTGAACAA
TGACCCATCATCTCTTGCTTGCTTGAAATCTTGCTGAAATGTTCTCATTTCCTGTTTGCTGTATGGGCTC
GGGTGGGATGTTTGTTGGCTCTGTTGTGTTTATTCACCAATTTGTACATTATTTGTTGTCCTTTACTACT
GTAAACAGTAAATATAGTTTGGTATTCTGTCAAAAAAAAAAAAAAAAAAAAAAAAA
```

```
>NP_001273345.1 small ubiquitin-related modifier 3 isoform 2 [Homo sapiens]
MSEEKPKEGVKTENDHINLKVAGQDGSVVQFKIKRHTPLSKLMKAYCERQVRHLAPPQSLPVCALVLCVP
GIPRARASRGWTQMQLPEGLSMRQIRFRFDGQPINETDTPAQLEMEDEDTIDVFQQQTGGVPESSLAGHS
F
```

```
i0_HQ_samplec9816d|c13953/f3p5/509      ------------------------------------------------------------  0
NM_006936.2                             AGCCACGTGACCCCCGTGCCGGCCAACGGGTGCGCCGGGATTTGGGGGATAAAGCGCGGC  60
NM_001286416.1                          AGCCACGTGACCCCCGTGCCGGCCAACGGGTGCGCCGGGATTTGGGGGATAAAGCGCGGC  60

i0_HQ_samplec9816d|c13953/f3p5/509      ------------------------------------------------------------  0
NM_006936.2                             CCCGCGCACAGTTGCGGCGGGAGAGCGGCGGGGCCGAGAGCGTGACTCGCCCGCTCCGCG  120
NM_001286416.1                          CCCGCGCACAGTTGCGGCGGGAGAGCGGCGGGGCCGAGAGCGTGACTCGCCCGCTCCGCG  120

i0_HQ_samplec9816d|c13953/f3p5/509      ------------------------------------------------------------  0
NM_006936.2                             CTGCTTCCCCCGCGCCGCCTCCCCGCGCCGCTCGCGCAGCCATGTCCGAGGAGAAGCCCA  180
NM_001286416.1                          CTGCTTCCCCCGCGCCGCCTCCCCGCGCCGCTCGCGCAGCCATGTCCGAGGAGAAGCCCA  180

i0_HQ_samplec9816d|c13953/f3p5/509      ------------------------------------------------------------  0
NM_006936.2                             AGGAGGGTGTGAAGACAGAGAATGACCACATCAACCTGAAGGTGGCCGGGCAGGACGGCT  240
NM_001286416.1                          AGGAGGGTGTGAAGACAGAGAATGACCACATCAACCTGAAGGTGGCCGGGCAGGACGGCT  240

i0_HQ_samplec9816d|c13953/f3p5/509      ------------------------------------------AgttgcGGC-------GG  11
NM_006936.2                             CCGTGGTGCAGTTCAAGATCAAGAGGCACACGCCGCTGAGCAAGCTGATGAAGGCCTACT  300
NM_001286416.1                          CCGTGGTGCAGTTCAAGATCAAGAGGCACACGCCGCTGAGCAAGCTGATGAAGGCCTACT  300
                                                                                  ** **  *

i0_HQ_samplec9816d|c13953/f3p5/509      GAGAGCGGCGGGGCCGAGAGCG-------------TGACTCGCCCGCTCCGCGCTGC---  55
NM_006936.2                             GCGAGAGGCA--------------------------------------------------  310
NM_001286416.1                          GCGAGAGGCAGGTGCGGCACCTTGCTCCCCCGCAGAGCCTCCCCGTGTGCGCACTGGTCC  360
                                        * *** ***

i0_HQ_samplec9816d|c13953/f3p5/509      ------TTCCCCCGCGCCGCCTCCCCGCGCCGCTCGGCGCAGCCATGTCCGAGGAGAAGC  109
NM_006936.2                             ------------------------------------------------------------  310
NM_001286416.1                          TGTGCGTTCCAGGCATCCCCAGAGCACGAGCGTCTCGGGGCTGGACCCAGATGCAGCTGC  420

i0_HQ_samplec9816d|c13953/f3p5/509      CCAAGGGCTTGTCAATGAGGCAGATCAGATTCAGGTTCGACGGGCAGCCAATCAATGAAA  169
NM_006936.2                             ----GGGCTTGTCAATGAGGCAGATCAGATTCAGGTTCGACGGGCAGCCAATCAATGAAA  366
NM_001286416.1                          CCGAGGGCTTGTCAATGAGGCAGATCAGATTCAGGTTCGACGGGCAGCCAATCAATGAAA  480
```

```
                                          ************************************************************

i0_HQ_samplec9816d|c13953/f3p5/509        CTGACACTCCAGCACAGCTGGAGATGGAGGACGAGGACACCATCGACGTGTTCCAGCAGC  229
NM_006936.2                               CTGACACTCCAGCACAGCTGGAGATGGAGGACGAGGACACCATCGACGTGTTCCAGCAGC  426
NM_001286416.1                            CTGACACTCCAGCACAGCTGGAGATGGAGGACGAGGACACCATCGACGTGTTCCAGCAGC  540
                                          ************************************************************

i0_HQ_samplec9816d|c13953/f3p5/509        AGACGGGAGGTGTGCCGGAGAGCAGCCTGGCAGGGCACAGTTTCTAGAGGGCCCGTCCCC  289
NM_006936.2                               AGACGGGAGGTGTGCCGGAGAGCAGCCTGGCAGGGCACAGTTTCTAGAGGGCCCGTCCCC  486
NM_001286416.1                            AGACGGGAGGTGTGCCGGAGAGCAGCCTGGCAGGGCACAGTTTCTAGAGGGCCCGTCCCC  600
                                          ************************************************************

i0_HQ_samplec9816d|c13953/f3p5/509        AGCCCGGGCCGTCCATCCTCGCATTGCTGTTGAATGGTGAGCACGTGACCATGCCGACCA  349
NM_006936.2                               AGCCCGGGCCGTCCATCCTCGCATTGCTGTTGAATGGTGAGCACGTGACCATGCCGACCA  546
NM_001286416.1                            AGCCCGGGCCGTCCATCCTCGCATTGCTGTTGAATGGTGAGCACGTGACCATGCCGACCA  660
                                          ************************************************************

i0_HQ_samplec9816d|c13953/f3p5/509        CAAAGGTGTCTGCGGAAACTCGAGGACATTCACCACGATGATTTTCCTCTCTTTGATGTA  409
NM_006936.2                               CAAAGGTGTCTGCGGAAACTCGAGGACATTCACCACGATGATTTTCCTCTCTTTGATGTA  606
NM_001286416.1                            CAAAGGTGTCTGCGGAAACTCGAGGACATTCACCACGATGATTTTCCTCTCTTTGATGTA  720
                                          ************************************************************

i0_HQ_samplec9816d|c13953/f3p5/509        CTTCAAGTGCAACTCAAAACTATATCTGCAGGGATGAATCTGTAACTTAAATTGGGCCAA  469
NM_006936.2                               CTTCAAGTGCAACTCAAAACTATATCTGCAGGGATGAATCTGTAACTTAAATTGGGCCAA  666
NM_001286416.1                            CTTCAAGTGCAACTCAAAACTATATCTGCAGGGATGAATCTGTAACTTAAATTGGGCCAA  780
                                          ************************************************************

i0_HQ_samplec9816d|c13953/f3p5/509        TCAGAATTGTTATCTTTGTTCAGGTAAAATGAGTTGCAAG--------------------  509
NM_006936.2                               TCAGAATTGTTATCTTTGTTCAGGTAAAATGAGTTGCAAGATATTGTGGGTACTTTTGTG  726
NM_001286416.1                            TCAGAATTGTTATCTTTGTTCAGGTAAAATGAGTTGCAAGATATTGTGGGTACTTTTGTG  840
                                          ****************************************

i0_HQ_samplec9816d|c13953/f3p5/509        ------------------------------------------------------------  509
NM_006936.2                               TGCTCATTTGTGTTTTCCCCCCCTCCTACAACATTTTTTTAACCCCAAAATTATAGCCTG  786
NM_001286416.1                            TGCTCATTTGTGTTTTCCCCCCCTCCTACAACATTTTTTTAACCCCAAAATTATAGCCTG  900

i0_HQ_samplec9816d|c13953/f3p5/509        ------------------------------------------------------------  509
NM_006936.2                               AATGTTCGCTTTTAGTCTGGCCAGGGATCTGACTCCTGAGTTGGTTGCCTCTCCCCTGCT  846
NM_001286416.1                            AATGTTCGCTTTTAGTCTGGCCAGGGATCTGACTCCTGAGTTGGTTGCCTCTCCCCTGCT  960

i0_HQ_samplec9816d|c13953/f3p5/509        ------------------------------------------------------------  509
NM_006936.2                               CACTCCAGTCACATAGAGAATTGGTGTTTCCCGCAGTGGGGATGCAGCTGTTGGACAGGT  906
NM_001286416.1                            CACTCCAGTCACATAGAGAATTGGTGTTTCCCGCAGTGGGGATGCAGCTGTTGGACAGGT  1020

i0_HQ_samplec9816d|c13953/f3p5/509        ------------------------------------------------------------  509
NM_006936.2                               ATTGGGGGCAAGGTTGGTAGGGAGGACAGACTGTCACTTGCTGTTACAGGCACAGGTGAT  966
NM_001286416.1                            ATTGGGGGCAAGGTTGGTAGGGAGGACAGACTGTCACTTGCTGTTACAGGCACAGGTGAT  1080

i0_HQ_samplec9816d|c13953/f3p5/509        ------------------------------------------------------------  509
NM_006936.2                               TAAAATGCTAAATATTGCAAATTTAAGCTTTGTCAGTATATGGAAAAGTTGAAGGGAAAA  1026
NM_001286416.1                            TAAAATGCTAAATATTGCAAATTTAAGCTTTGTCAGTATATGGAAAAGTTGAAGGGAAAA  1140

i0_HQ_samplec9816d|c13953/f3p5/509        ------------------------------------------------------------  509
NM_006936.2                               TACTGGAATGCTTCTTCAAAGGTTAAAAAATAACCGAGTCTTTTGGTAATTTGACCCCAC  1086
NM_001286416.1                            TACTGGAATGCTTCTTCAAAGGTTAAAAAATAACCGAGTCTTTTGGTAATTTGACCCCAC  1200

i0_HQ_samplec9816d|c13953/f3p5/509        ------------------------------------------------------------  509
NM_006936.2                               GTGCTCTCTGGCCCTCAAGCATGTAACCTCGGGGTCTGAGGCCCAGGACCCACCCCCCTG  1146
NM_001286416.1                            GTGCTCTCTGGCCCTCAAGCATGTAACCTCGGGGTCTGAGGCCCAGGACCCACCCCCCTG  1260

i0_HQ_samplec9816d|c13953/f3p5/509        ------------------------------------------------------------  509
NM_006936.2                               CCACCCCTCCCACCCCACTCCCTGCTCAGTACCTGGCGTTGGTACACAGGCAAGGATTGG  1206
NM_001286416.1                            CCACCCCTCCCACCCCACTCCCTGCTCAGTACCTGGCGTTGGTACACAGGCAAGGATTGG  1320

i0_HQ_samplec9816d|c13953/f3p5/509        ------------------------------------------------------------  509
NM_006936.2                               CACAACCAAAATTGGCCTTTTTCTCCCTCTTAATATTGAAGAAATTCCCACATTTCTCAT  1266
NM_001286416.1                            CACAACCAAAATTGGCCTTTTTCTCCCTCTTAATATTGAAGAAATTCCCACATTTCTCAT  1380

i0_HQ_samplec9816d|c13953/f3p5/509        ------------------------------------------------------------  509
NM_006936.2                               TTGGTAATGGTGTTGTGGCCTCAGATTTCTTCTAGTATTTGCTTCTGATGAATGATTATG  1326
NM_001286416.1                            TTGGTAATGGTGTTGTGGCCTCAGATTTCTTCTAGTATTTGCTTCTGATGAATGATTATG  1440
```

```
i0_HQ_samplec9816d|c13953/f3p5/509      ------------------------------------------------------------  509
NM_006936.2                             GTCTATACATAAAAAAGTAAGACTAAGTATTGCTGAATTTGCAGTTATGTTGTCGTGTAT  1386
NM_001286416.1                          GTCTATACATAAAAAAGTAAGACTAAGTATTGCTGAATTTGCAGTTATGTTGTCGTGTAT  1500

i0_HQ_samplec9816d|c13953/f3p5/509      ------------------------------------------------------------  509
NM_006936.2                             AAGAGCTACTTCCAAGTGTGGTTACAAATGAACCCATGGAATGATGACTTCATGTTCTTC  1446
NM_001286416.1                          AAGAGCTACTTCCAAGTGTGGTTACAAATGAACCCATGGAATGATGACTTCATGTTCTTC  1560

i0_HQ_samplec9816d|c13953/f3p5/509      ------------------------------------------------------------  509
NM_006936.2                             TCGTGGGTTTGTGCCGTGCTGCTTTCCAAATAGGTATTGAATTTATGCATTAGTCTGGTG  1506
NM_001286416.1                          TCGTGGGTTTGTGCCGTGCTGCTTTCCAAATAGGTATTGAATTTATGCATTAGTCTGGTG  1620

i0_HQ_samplec9816d|c13953/f3p5/509      ------------------------------------------------------------  509
NM_006936.2                             ATTTCAGTTCTGTGAAATATTTTGGGATCTATACCAATTAAACATTTTCATAGTTCTGCC  1566
NM_001286416.1                          ATTTCAGTTCTGTGAAATATTTTGGGATCTATACCAATTAAACATTTTCATAGTTCTGCC  1680

i0_HQ_samplec9816d|c13953/f3p5/509      ------------------------------------------------------------  509
NM_006936.2                             TATTGTCCTTCCCTGAGGCTCCATTGCTGCTTGGTGGCCATTCTCTGCCTTTTTACAGTC  1626
NM_001286416.1                          TATTGTCCTTCCCTGAGGCTCCATTGCTGCTTGGTGGCCATTCTCTGCCTTTTTACAGTC  1740

i0_HQ_samplec9816d|c13953/f3p5/509      ------------------------------------------------------------  509
NM_006936.2                             ACCTGAACAATGACCCATCATCTCTTGCTTGCTTGAAATCTTGCTGAAATGTTCTCATTT  1686
NM_001286416.1                          ACCTGAACAATGACCCATCATCTCTTGCTTGCTTGAAATCTTGCTGAAATGTTCTCATTT  1800

i0_HQ_samplec9816d|c13953/f3p5/509      ------------------------------------------------------------  509
NM_006936.2                             CCTGTTTGCTGTATGGGCTCGGGTGGGATGTTTGTTGGCTCTGTTGTGTTTATTCACCAA  1746
NM_001286416.1                          CCTGTTTGCTGTATGGGCTCGGGTGGGATGTTTGTTGGCTCTGTTGTGTTTATTCACCAA  1860

i0_HQ_samplec9816d|c13953/f3p5/509      ------------------------------------------------------------  509
NM_006936.2                             TTTGTACATTATTTGTTGTCCTTTACTACTGTAAACAGTAAATATAGTTTGGTATTCTGT  1806
NM_001286416.1                          TTTGTACATTATTTGTTGTCCTTTACTACTGTAAACAGTAAATATAGTTTGGTATTCTGT  1920

i0_HQ_samplec9816d|c13953/f3p5/509      -------------------------       509
NM_006936.2                             CAAAAAAAAAAAAAAAAAAAAAAAA       1831
NM_001286416.1                          CAAAAAAAAAAAAAAAAAAAAAAAA       1945
```

```
VIRT175354          ------------------------------------------------------------ 0
NP_008867.2         MSEEKPKEGVKTENDHINLKVAGQDGSVVQFKIKRHTPLSKLMKAYCERQ---------- 50
NP_001273345.1      MSEEKPKEGVKTENDHINLKVAGQDGSVVQFKIKRHTPLSKLMKAYCERQVRHLAPPQSL 60
VIRT176199          ------------------MKLTLQH--SWRWRTRTPSTCSSSRREVCRRAAWQGT----- 35

VIRT175354          ---------------------MSEEKPKGLSMRQIRFRFDGQPINETDTPAQLEMEDEDT 39
NP_008867.2         ----------------------------GLSMRQIRFRFDGQPINETDTPAQLEMEDEDT 82
NP_001273345.1      PVCALVLCVPGIPRARASRGWTQMQLPEGLSMRQIRFRFDGQPINETDTPAQLEMEDEDT 120
VIRT176199          -VSRGPVPSPGRPSSHCC------------------------------------------ 52

VIRT175354          IDVFQQQTGGVPESSLAGHSF          60
NP_008867.2         IDVFQQQTGGVPESSLAGHSF          103
NP_001273345.1      IDVFQQQTGGVPESSLAGHSF          141
VIRT176199          ---------------------          52
```

## DGCR6L

| Sequence | Header | AA Seq | |
|---|---|---|---|
| Present | i1_HQ_samplec9816d\|c64363/f2p0/1107 | VIRT194119 | |
| Absent | i1_HQ_samplec9816d\|c55690/f2p0/1026 | VIRT195959 | VIRT671 |
| NCBI | NM_033257 | NP_150282.2 | |
| | | | |

>i1_HQ_samplec9816d|c64363/f2p0/1107
isoform=c64363;full_length_coverage=2;non_full_length_coverage=0;isoform_length=1107

GtcgccgccgccggcggctagcgggcgtccgCGCCATGGAGCGCTACGCGGCCGCCTTGGAGGAGGTGGCGGACGGTGCCCGGC
AGCAGGAGCGACACTACCAGTTGCTGTCGGCGCTACAGAGCCTGGTGAAGGAGTTGCCCAGCTCTTTCCAGCAGCGCC
TGTCCTACACCACGCTCAGCGACCTGGCCCTGGCGCTTCTCGACGGCACCGTGTTCGAAATCGTGCAGGGGCTACTGGA
GATCCAGCACCTCACCGAAAAGAGCCTGTACAACCAGCGCCTGCGCCTACAGAACGAGCACCGAGTGCTCAGGCAGGC
GCTGCGGCAGAAGCACCAGGAAGCCCAGCAGGCCTGCCGGCCCCACAACCTGCCTGTGGTTCAGGCGGCTCAGCAGC
GAGAACTAGAGGCCGTGGAACACCGGATCCGTGAGGAGCAGCGGGCGATGGACCAGAAGATCATCCTGGAGCTGGA
CCGGAAGGTGGCTGACCAGCAGAGCACACTGGAGAAGGCGGGGGTGGCTGGCTTCTACGTGACCACCAACCCACAGG
AGCTGATGCTGCAGATGAACCTGCTGGAACTCATCCGAAAGCTGCAGCAGAGGGGCTGCCGGGCAGGGAATGCAGCC
CTGGGACTGGGAGGTCCCTGGCAGTCGCCTGCTGCCCAGTGTGACCAGAAAGGCAGCCCTGTCCCACCATAGCCACAG
GCAGCAGAAGTCTGGGCAGAGTTCATCTTCTTGACCTTTGGCCACTGCCTTCCCAGCTGCCCGCAGGGGGTTCCCCCTG
CTGAGGAGAGACCAGGTGGACCCCAGCTGCCTGTCACCCTTCATCTGGGACTTGCTGTCAAACCCTAGGATAGTCTCAT
AAAGGGGAGGCTGGGCCAGCCTGCTGCTGTCTGCTTCAGGACCAGGCAGAGAGTGAGGCTGGGGGTTCTCACACCTT
ACTCCACCGGGCACATCCCAACCTGCACTGGGGCCCACCCGAGCGCTTGTTCTGGTCTCAGCCGCTCCCTTGGCAGCTG
CAGCCCCCATGCAGAAGAGGCTCCCAGGCCCAAGCTCTGTGTGACCCAGAGAaataaagatgcctcagtgtggcccacatgtggctt
gt

>VIRT194119

MERYAAALEEVADGARQQERHYQLLSALQSLVKELPSSFQQRLSYTTLSDLALALLDGTV
FEIVQGLLEIQHLTEKSLYNQRLRLQNEHRVLRQALRQKHQEAQQACRPHNLPVVQAAQQ
RELEAVEHRIREEQRAMDQKIILELDRKVADQQSTLEKAGVAGFYVTTNPQELMLQMNLL
ELIRKLQQRGCRAGNAALGLGGPWQSPAAQCDQKGSPVPP

>i1_HQ_samplec9816d|c55690/f2p0/1026
isoform=c55690;full_length_coverage=2;non_full_length_coverage=0;isoform_length=1026

CgccgcgctcgggatgtcgtagctggggagctgtcgccgcgccgcggctagcgggcgtcgcgCCATGGAGCGCTACGCGGCCGCCTTGGAGG
AGGTGGCGGACGGTGCCCGGCAGCAGGAGCGACACTACCAGTTGCTGTCGGCGCTACAGAGCCTGGTGAAGGAGTTG
CCCAGCTCTTTCCAGCAGCGCCTGTCCTACACCACGCTCAGCGACCTGGCCCTGGCGCTTCTCGACGGCACCGTGTTCGA
AATCGTGCAGGGGCTACTGGAGATCCAGCACCTCACCGAAAAGAGCCTGTACAACCAGCGCCTGCGCCTACAGAACGA
GCACCGAGGCCGTGGAACACCGGATCCGTGAGGAGCAGCGGGCGATGGACCAGAAGATCATCCTGGAGCTGGACCG
GAAGGTGGCTGACCAGCAGAGCACACTGGAGAAGGCGGGGGTGGCTGGCTTCTACGTGACCACCAACCCACAGGAGC
TGATGCTGCAGATGAACCTGCTGGAACTCATCCGAAAGCTGCAGCAGAGGGGCTGCCGGGCAGGGAATGCAGCCCTG
GGACTGGGAGGTCCCTGGCAGTCGCCTGCTGCCCAGTGTGACCAGAAAGGCAGCCCTGTCCCACCATAGCCACAGGCA
GCAGAAGTCTGGGCAGAGTTCATCTTCTTGACCTTTGGCCACTGCCTTCCCAGCTGCCCGCAGGGGGTTCCCCCTGCTG
AGGAGAGACCAGGTGGACCCCAGCTGCCTGTCACCCTTCATCTGGGACTTGCTGTCAAACCCTAGGATAGTCTCATAAA
GGGGAGGCTGGGCCAGCCTGCTGCTGTCTGCTTCAGGACCAGGCAGAGAGTGAGGCTGGGGGTTCTCACACCTTACTC
CACCGGGCACATCCCAACCTGCACTGGGGCCCACCCGAGCGCTTGTTCTGGTCTCAGCCGCTCCCTTGGCAGCTGCAGC
CCCCATGCAGAAGAGGCTCCAGGCCCAAGCTCTGTGTGACccagagaaataaagatgcctcagtgtggcccacatg

>VIRT195959

MERYAAALEEVADGARQQERHYQLLSALQSLVKELPSSFQQRLSYTTLSDLALALLDGTV
FEIVQGLLEIQHLTEKSLYNQRLRLQNEHRGRGTPDP

>VIRT671

MDQKIILELDRKVADQQSTLEKAGVAGFYVTTNPQELMLQMNLLELIRKLQQRGCRAGNA
ALGLGGPWQSPAAQCDQKGSPVPP

```
>NM_033257.3 Homo sapiens DiGeorge syndrome critical region gene 6 like (DGCR6L), mRNA
GGGCGGAGCGCGCCGCGCGTGGGACAATCGTAAAAGGGGCGGGACGCGCCGCGCTCGGGATGTCGTGAAG
CTGGGGGAGCTCGTCGCCGCCGCCGGCGGCTAGCGGGCGTCCGCGCCATGGAGCGCTACGCGGCCGCCTT
GGAGGAGGTGGCGGACGGTGCCCGGCAGCAGGAGCGACACTACCAGTTGCTGTCGGCGCTACAGAGCCTG
GTGAAGGAGTTGCCCAGCTCTTTCCAGCAGCGCCTGTCCTACACCACGCTCAGCGACCTGGCCCTGGCGC
TTCTCGACGGCACCGTGTTCGAAATCGTGCAGGGGCTACTGGAGATCCAGCACCTCACCGAAAAGAGCCT
GTACAACCAGCGCCTGCGCCTACAGAACGAGCACCGAGTGCTCAGGCAGGCGCTGCGGCAGAAGCACCAG
GAAGCCCAGCAGGCCTGCCGGCCCCACAACCTGCCTGTGGTTCAGGCGGCTCAGCAGCGAGAACTAGAGG
CCGTGGAACACCGGATCCGTGAGGAGCAGCGGGCGATGGACCAGAAGATCATCCTGGAGCTGGACCGGAA
GGTGGCTGACCAGCAGAGCACACTGGAGAAGGCGGGGGTGGCTGGCTTCTACGTGACCACCAACCCACAG
GAGCTGATGCTGCAGATGAACCTGCTGGAACTCATCCGAAAGCTGCAGCAGAGGGGCTGCCGGGCAGGGA
ATGCAGCCCTGGGACTGGGAGGTCCCTGGCAGTCGCCTGCTGCCCAGTGTGACCAGAAAGGCAGCCCTGT
CCCACCATAGCCACAGGCAGCAGAAGTCTGGGCAGAGTTCATCTTCTTGACCTTTGGCCACTGCCTTCCC
AGCTGCCCGCAGGGGGTTCCCCCTGCTGAGGAGAGACCAGGTGGACCCCAGCTGCCTGTCACCCTTCATC
TGGGACTTGCTGTCAAACCCTAGGATAGTCTCATAAAGGGGAGGCTGGGCCAGCCTGCTGCTGTCTGCTT
CAGGACCAGGCAGAGAGTGAGGCTGGGGGTTCTCACACCTTACTCCACCGGGCACATCCCAACCTGCACT
GGGGCCCACCCGAGCGCTTGTTCTGGTCTCAGCCGCTCCCTTGGCAGCTGCAGCCCCCATGCAGAAGAGG
CTCCCAGGCCCAAGCTCTGTGTGACCCAGAGAAATAAAGATGCCTCAGTGTGGCCCAAATGTGGCTTGTG
TGATAGCTGCAGGTTCTGCCATAACCG
```

```
>NP_150282.2 protein DGCR6L [Homo sapiens]
MERYAAALEEVADGARQQERHYQLLSALQSLVKELPSSFQQRLSYTTLSDLALALLDGTVFEIVQGLLEI
QHLTEKSLYNQRLRLQNEHRVLRQALRQKHQEAQQACRPHNLPVVQAAQQRELEAVEHRIREEQRAMDQK
IILELDRKVADQQSTLEKAGVAGFYVTTNPQELMLQMNLLELIRKLQQRGCRAGNAALGLGGPWQSPAAQ
CDQKGSPVPP
```

```
i1_HQ_samplec9816d|c55690/f2p0/1026      ----------------------------------------------Cgccgcgctcggga 14
i1_HQ_samplec9816d|c64363/f2p0/1107      ------------------------------------------------------------ 0
NM_033257.3                              GGGCGGAGCGCGCCGCGCGTGGGACAATCGTAAAAGGGGCGGGACGCGCCGCGCTCGGGA 60


i1_HQ_samplec9816d|c55690/f2p0/1026      tgtcgtagctggg-------gagctgtcgccgcgccgcggctagcgggcgtcgcgCCATG 67
i1_HQ_samplec9816d|c64363/f2p0/1107      ----------------------GtcgccgccgccggcggctagcgggcgtccgCGCCATG 38
NM_033257.3                              TGTCGTGAAGCTGGGGGAGCTCGTCGCCGCCGCCGGCGGCTAGCGGGCGTCCGCGCCATG 120
                                                               *  * ******     *      **    *********

i1_HQ_samplec9816d|c55690/f2p0/1026      GAGCGCTACGCGGCCGCCTTGGAGGAGGTGGCGGACGGTGCCCGGCAGCAGGAGCGACAC 127
i1_HQ_samplec9816d|c64363/f2p0/1107      GAGCGCTACGCGGCCGCCTTGGAGGAGGTGGCGGACGGTGCCCGGCAGCAGGAGCGACAC 98
NM_033257.3                              GAGCGCTACGCGGCCGCCTTGGAGGAGGTGGCGGACGGTGCCCGGCAGCAGGAGCGACAC 180
                                         ************************************************************

i1_HQ_samplec9816d|c55690/f2p0/1026      TACCAGTTGCTGTCGGCGCTACAGAGCCTGGTGAAGGAGTTGCCCAGCTCTTTCCAGCAG 187
i1_HQ_samplec9816d|c64363/f2p0/1107      TACCAGTTGCTGTCGGCGCTACAGAGCCTGGTGAAGGAGTTGCCCAGCTCTTTCCAGCAG 158
NM_033257.3                              TACCAGTTGCTGTCGGCGCTACAGAGCCTGGTGAAGGAGTTGCCCAGCTCTTTCCAGCAG 240
                                         ************************************************************

i1_HQ_samplec9816d|c55690/f2p0/1026      CGCCTGTCCTACACCACGCTCAGCGACCTGGCCCTGGCGCTTCTCGACGGCACCGTGTTC 247
i1_HQ_samplec9816d|c64363/f2p0/1107      CGCCTGTCCTACACCACGCTCAGCGACCTGGCCCTGGCGCTTCTCGACGGCACCGTGTTC 218
NM_033257.3                              CGCCTGTCCTACACCACGCTCAGCGACCTGGCCCTGGCGCTTCTCGACGGCACCGTGTTC 300
                                         ************************************************************

i1_HQ_samplec9816d|c55690/f2p0/1026      GAAATCGTGCAGGGGCTACTGGAGATCCAGCACCTCACCGAAAAGAGCCTGTACAACCAG 307
i1_HQ_samplec9816d|c64363/f2p0/1107      GAAATCGTGCAGGGGCTACTGGAGATCCAGCACCTCACCGAAAAGAGCCTGTACAACCAG 278
NM_033257.3                              GAAATCGTGCAGGGGCTACTGGAGATCCAGCACCTCACCGAAAAGAGCCTGTACAACCAG 360
                                         ************************************************************

i1_HQ_samplec9816d|c55690/f2p0/1026      CGCCTGCGCCTACAGAACGAGCACCGA--------------------------------- 334
i1_HQ_samplec9816d|c64363/f2p0/1107      CGCCTGCGCCTACAGAACGAGCACCGAGTGCTCAGGCAGGCGCTGCGGCAGAAGCACCAG 338
NM_033257.3                              CGCCTGCGCCTACAGAACGAGCACCGAGTGCTCAGGCAGGCGCTGCGGCAGAAGCACCAG 420
                                         ***************************

i1_HQ_samplec9816d|c55690/f2p0/1026      ------------------------------------------------------------ 334
i1_HQ_samplec9816d|c64363/f2p0/1107      GAAGCCCAGCAGGCCTGCCGGCCCCACAACCTGCCTGTGGTTCAGGCGGCTCAGCAGCGA 398
NM_033257.3                              GAAGCCCAGCAGGCCTGCCGGCCCCACAACCTGCCTGTGGTTCAGGCGGCTCAGCAGCGA 480


i1_HQ_samplec9816d|c55690/f2p0/1026      --------GGCCGTGGAACACCGGATCCGTGAGGAGCAGCGGGCGATGGACCAGAAGATC 386
```

```
i1_HQ_samplec9816d|c64363/f2p0/1107      GAACTAGAGGCCGTGGAACACCGGATCCGTGAGGAGCAGCGGGCGATGGACCAGAAGATC 458
NM_033257.3                              GAACTAGAGGCCGTGGAACACCGGATCCGTGAGGAGCAGCGGGCGATGGACCAGAAGATC 540
                                                 ****************************************************

i1_HQ_samplec9816d|c55690/f2p0/1026      ATCCTGGAGCTGGACCGGAAGGTGGCTGACCAGCAGAGCACACTGGAGAAGGCGGGGGTG 446
i1_HQ_samplec9816d|c64363/f2p0/1107      ATCCTGGAGCTGGACCGGAAGGTGGCTGACCAGCAGAGCACACTGGAGAAGGCGGGGGTG 518
NM_033257.3                              ATCCTGGAGCTGGACCGGAAGGTGGCTGACCAGCAGAGCACACTGGAGAAGGCGGGGGTG 600
                                         ************************************************************

i1_HQ_samplec9816d|c55690/f2p0/1026      GCTGGCTTCTACGTGACCACCAACCCACAGGAGCTGATGCTGCAGATGAACCTGCTGGAA 506
i1_HQ_samplec9816d|c64363/f2p0/1107      GCTGGCTTCTACGTGACCACCAACCCACAGGAGCTGATGCTGCAGATGAACCTGCTGGAA 578
NM_033257.3                              GCTGGCTTCTACGTGACCACCAACCCACAGGAGCTGATGCTGCAGATGAACCTGCTGGAA 660
                                         ************************************************************

i1_HQ_samplec9816d|c55690/f2p0/1026      CTCATCCGAAAGCTGCAGCAGAGGGGCTGCCGGGCAGGGAATGCAGCCCTGGGACTGGGA 566
i1_HQ_samplec9816d|c64363/f2p0/1107      CTCATCCGAAAGCTGCAGCAGAGGGGCTGCCGGGCAGGGAATGCAGCCCTGGGACTGGGA 638
NM_033257.3                              CTCATCCGAAAGCTGCAGCAGAGGGGCTGCCGGGCAGGGAATGCAGCCCTGGGACTGGGA 720
                                         ************************************************************

i1_HQ_samplec9816d|c55690/f2p0/1026      GGTCCCTGGCAGTCGCCTGCTGCCCAGTGTGACCAGAAAGGCAGCCCTGTCCCACCATAG 626
i1_HQ_samplec9816d|c64363/f2p0/1107      GGTCCCTGGCAGTCGCCTGCTGCCCAGTGTGACCAGAAAGGCAGCCCTGTCCCACCATAG 698
NM_033257.3                              GGTCCCTGGCAGTCGCCTGCTGCCCAGTGTGACCAGAAAGGCAGCCCTGTCCCACCATAG 780
                                         ************************************************************

i1_HQ_samplec9816d|c55690/f2p0/1026      CCACAGGCAGCAGAAGTCTGGGCAGAGTTCATCTTCTTGACCTTTGGCCACTGCCTTCCC 686
i1_HQ_samplec9816d|c64363/f2p0/1107      CCACAGGCAGCAGAAGTCTGGGCAGAGTTCATCTTCTTGACCTTTGGCCACTGCCTTCCC 758
NM_033257.3                              CCACAGGCAGCAGAAGTCTGGGCAGAGTTCATCTTCTTGACCTTTGGCCACTGCCTTCCC 840
                                         ************************************************************

i1_HQ_samplec9816d|c55690/f2p0/1026      AGCTGCCCGCAGGGGGTTCCCCCTGCTGAGGAGAGACCAGGTGGACCCCAGCTGCCTGTC 746
i1_HQ_samplec9816d|c64363/f2p0/1107      AGCTGCCCGCAGGGGGTTCCCCCTGCTGAGGAGAGACCAGGTGGACCCCAGCTGCCTGTC 818
NM_033257.3                              AGCTGCCCGCAGGGGGTTCCCCCTGCTGAGGAGAGACCAGGTGGACCCCAGCTGCCTGTC 900
                                         ************************************************************

i1_HQ_samplec9816d|c55690/f2p0/1026      ACCCTTCATCTGGGACTTGCTGTCAAACCCTAGGATAGTCTCATAAAGGGGAGGCTGGGC 806
i1_HQ_samplec9816d|c64363/f2p0/1107      ACCCTTCATCTGGGACTTGCTGTCAAACCCTAGGATAGTCTCATAAAGGGGAGGCTGGGC 878
NM_033257.3                              ACCCTTCATCTGGGACTTGCTGTCAAACCCTAGGATAGTCTCATAAAGGGGAGGCTGGGC 960
                                         ************************************************************

i1_HQ_samplec9816d|c55690/f2p0/1026      CAGCCTGCTGCTGTCTGCTTCAGGACCAGGCAGAGAGTGAGGCTGGGGGTTCTCACACCT 866
i1_HQ_samplec9816d|c64363/f2p0/1107      CAGCCTGCTGCTGTCTGCTTCAGGACCAGGCAGAGAGTGAGGCTGGGGGTTCTCACACCT 938
NM_033257.3                              CAGCCTGCTGCTGTCTGCTTCAGGACCAGGCAGAGAGTGAGGCTGGGGGTTCTCACACCT 1020
                                         ************************************************************

i1_HQ_samplec9816d|c55690/f2p0/1026      TACTCCACCGGGCACATCCCAACCTGCACTGGGGCCCACCCGAGCGCTTGTTCTGGTCTC 926
i1_HQ_samplec9816d|c64363/f2p0/1107      TACTCCACCGGGCACATCCCAACCTGCACTGGGGCCCACCCGAGCGCTTGTTCTGGTCTC 998
NM_033257.3                              TACTCCACCGGGCACATCCCAACCTGCACTGGGGCCCACCCGAGCGCTTGTTCTGGTCTC 1080
                                         ************************************************************

i1_HQ_samplec9816d|c55690/f2p0/1026      AGCCGCTCCCTTGGCAGCTGCAGCCCCCATGCAGAAGAGGCTCC-AGGCCCAAGCTCTGT 985
i1_HQ_samplec9816d|c64363/f2p0/1107      AGCCGCTCCCTTGGCAGCTGCAGCCCCCATGCAGAAGAGGCTCCCAGGCCCAAGCTCTGT 1058
NM_033257.3                              AGCCGCTCCCTTGGCAGCTGCAGCCCCCATGCAGAAGAGGCTCCCAGGCCCAAGCTCTGT 1140
                                         ******************************************** ***************

i1_HQ_samplec9816d|c55690/f2p0/1026      GTGACccagagaaataaagatgcctcagtgtggcccacatg------------------- 1026
i1_HQ_samplec9816d|c64363/f2p0/1107      GTGACCCAGAGAaataaagatgcctcagtgtggcccacatgtggcttgt----------- 1107
NM_033257.3                              GTGACCCAGAGAAATAAAGATGCCTCAGTGTGGCCCAAATGTGGCTTGTGTGATAGCTGC 1200
                                         ************************************* ***

i1_HQ_samplec9816d|c55690/f2p0/1026      -----------------   1026
i1_HQ_samplec9816d|c64363/f2p0/1107      -----------------   1107
NM_033257.3                              AGGTTCTGCCATAACCG   1217
```

```
VIRT195959        MERYAAALEEVADGARQQERHYQLLSALQSLVKELPSSFQQRLSYTTLSDLALALLDGTV     60
VIRT194119        MERYAAALEEVADGARQQERHYQLLSALQSLVKELPSSFQQRLSYTTLSDLALALLDGTV     60
VIRT671           ------------------------------------------------------------     0
NP_150282.2       MERYAAALEEVADGARQQERHYQLLSALQSLVKELPSSFQQRLSYTTLSDLALALLDGTV     60

VIRT195959        FEIVQGLLEIQHLTEKSLYNQRLRLQNEHRGRGTPDP-----------------------     97
VIRT194119        FEIVQGLLEIQHLTEKSLYNQRLRLQNEHRVLRQALRQKHQEAQQACRPHNLPVVQAAQQ     120
VIRT671           ------------------------------------------------------------     0
NP_150282.2       FEIVQGLLEIQHLTEKSLYNQRLRLQNEHRVLRQALRQKHQEAQQACRPHNLPVVQAAQQ     120

VIRT195959        ------------------------------------------------------------     97
```

```
VIRT194119        RELEAVEHRIREEQRAMDQKIILELDRKVADQQSTLEKAGVAGFYVTTNPQELMLQMNLL     180
VIRT671           ----------------MDQKIILELDRKVADQQSTLEKAGVAGFYVTTNPQELMLQMNLL     44
NP_150282.2       RELEAVEHRIREEQRAMDQKIILELDRKVADQQSTLEKAGVAGFYVTTNPQELMLQMNLL     180
```

```
VIRT195959        ----------------------------------------     97
VIRT194119        ELIRKLQQRGCRAGNAALGLGGPWQSPAAQCDQKGSPVPP     220
VIRT671           ELIRKLQQRGCRAGNAALGLGGPWQSPAAQCDQKGSPVPP     84
NP_150282.2       ELIRKLQQRGCRAGNAALGLGGPWQSPAAQCDQKGSPVPP     220
```

**PDCD10**

| Sequence | Header | AA Seq |
|---|---|---|
| **Present** | | |
| **Absent** | i0_HQ_samplec9816d\|c43184/f2p1/954 | >VIRT111671 |
| **NCBI** | NM_145859 | NP_665858.1 |
| | NM_145860 | NP_665859.1 |
| | NM_007217 | NP_009148.2 |

>i0_HQ_samplec9816d|c43184/f2p1/954
isoform=c43184;full_length_coverage=2;non_full_length_coverage=1;isoform_length=954

ggagtgaattgatccgggagtttgaagagggctgcaaggtgggaagtgaagtcagtgcctcagttgctGGCTGAAAAAGAAAATCCAGGTCTCA
CACAAGACATCATTATGAAAATTTTAGAGAAAAAAAGCGTGGAAGTTAACTTCACGGAGTCCCTTCTTCGTATGGCAGC
TGATGATGTAGAAGAGTATATGATTGAACGACCAGAGCCAGAATTCCAAGACCTAAACGAAAAGGCACGAGCACTTAA
ACAAATTCTCAGTAAGATCCCAGATGAGATCAATGACAGAGTGAGGTTTCTGCAGACAATCAAGGATATAGCTAGTGC
AATAAAAGAACTTCTTGATACAGTGAATAATGTCTTCAAGAAATATCAATACCAGAACCGCAGGGCACTTGAACACCAA
AAGAAAGAATTTGTAAAGTACTCCAAAAGTTTCAGTGATACTCTGAAAACGTATTTTAAAGATGGCAAGGCAATAAATG
TGTTCGTAAGTGCCAACCGACTAATTCATCAAACCAACTTAATACTTCAGACCTCAAAACTGTGGCCTGAAAGTTGTATA
TGTTAAGAGATGTACTTCTCAGTGGCAGTATTGAACTGCCTTTATCTGTAAATTTTAAAGTTTGACTGTATAAATTATCAG
TCCCTCCTGAAGGGATCTAATCCAGGATGTTGAATGGGATTATTGCCATCTTACACCATATTTTTGTAAAATGTAGCTTA
ATCATAATCTCACACTGAAGATTTTGCATCACTTTTGCTATTATCATTCTTTTAAGAATTATAAGCCAAAAGAATTTACGC
CTTAATGTGTCATTATATAACATTCCTTAAAAGAATTGTAAATATTGGTGTTTGTTTCTGACATTTTAACTTGAAAGCGAT
ATGCTGCAAGATAATGTATTTAACAATATTTGGTGGCAAATATTCAATAAATAGTTTACATCTG

>VIRT111671

MKILEKKSVEVNFTESLLRMAADDVEEYMIERPEPEFQDLNEKARALKQILSKIPDEIND
RVRFLQTIKDIASAIKELLDTVNNVFKKYQYQNRRALEHQKKEFVKYSKSFSDTLKTYFK
DGKAINVFVSANRLIHQTNLILQTSKLWPESCIC

```
>NM_145859.1 Homo sapiens programmed cell death 10 (PDCD10), transcript variant 2, mRNA
GATCCGGGAGTTGAAGAGGGCTGCAAGGTGGGAAGTGAAGTCAGTGCCTCAGTTGCTGCTTTCTCCTCTA
AATCTTGGTCTCTTGTTTGGAAGTGACTAACAGCATTTTGGCATGAGAAAGCCCTAAAAAGATCAGTGTG
TTTTTTGTGTCCAATTCTTTTATCACCAAAAAAGAGAAGAAATATTGCAGTGAATGAAGATTCCTCTGCA
TTTTAGCACTGCTTTTTCAACTGTAGTTGGCTTTTGAATGAGGATGACAATGGAAGAGATGAAGAATGAA
GCTGAGACCACATCCATGGTTTCTATGCCCCTCTATGCAGTCATGTATCCTGTGTTTAATGAGCTAGAAC
GAGTAAATCTGTCTGCAGCCCAGACACTGAGAGCCGCTTTCATCAAGGCTGAAAAAGAAAATCCAGGTCT
CACACAAGACATCATTATGAAAATTTTAGAGAAAAAAAGCGTGGAAGTTAACTTCACGGAGTCCCTTCTT
CGTATGGCAGCTGATGATGTAGAAGAGTATATGATTGAACGACCAGAGCCAGAATTCCAAGACCTAAACG
AAAAGGCACGAGCACTTAAACAAATTCTCAGTAAGATCCCAGATGAGATCAATGACAGAGTGAGGTTTCT
GCAGACAATCAAGGATATAGCTAGTGCAATAAAAGAACTTCTTGATACAGTGAATAATGTCTTCAAGAAA
TATCAATACCAGAACCGCAGGGCACTTGAACACCAAAAGAAAGAATTTGTAAAGTACTCCAAAAGTTTCA
GTGATACTCTGAAAACGTATTTTAAAGATGGCAAGGCAATAAATGTGTTCGTAAGTGCCAACCGACTAAT
TCATCAAACCAACTTAATACTTCAGACCTTCAAAACTGTGGCCTGAAAGTTGTATATGTTAAGAGATGTA
CTTCTCAGTGGCAGTATTGAACTGCCTTTATCTGTAAATTTTAAAGTTTGACTGTATAAATTATCAGTCC
CTCCTGAAGGGATCTAATCCAGGATGTTGAATGGGATTATTGCCATCTTACACCATATTTTTGTAAAATG
```

```
TAGCTTAATCATAATCTCACACTGAAGATTTTGCATCACTTTTGCTATTATCATTCTTTTAAGAATTATA
AGCCAAAAGAATTTACGCCTTAATGTGTCATTATATAACATTCCTTAAAAGAATTGTAAATATTGGTGTT
TGTTTCTGACATTTTAACTTGAAAGCGATATGCTGCAAGATAATGTATTTAACAATATTTGGTGGCAAAT
ATTCAATAAATAGTTTACATCTGTTAAAAAAAAAAAAAAAAAAAAAAAAAAAA
```

```
>NP_665858.1 programmed cell death protein 10 [Homo sapiens]
MRMTMEEMKNEAETTSMVSMPLYAVMYPVFNELERVNLSAAQTLRAAFIKAEKENPGLTQDIIMKILEKK
SVEVNFTESLLRMAADDVEEYMIERPEPEFQDLNEKARALKQILSKIPDEINDRVRFLQTIKDIASAIKE
LLDTVNNVFKKYQYQNRRALEHQKKEFVKYSKSFSDTLKTYFKDGKAINVFVSANRLIHQTNLILQTFKT
VA
```

```
>NM_145860.1 Homo sapiens programmed cell death 10 (PDCD10), transcript variant 3, mRNA
TGCAAGGTGGGAAGTGAAGTCAGTGCCTCAGTTGCTGATCAGTGTGTTTTTTGTGTCCAATTCTTTTATC
ACCAAAAAAGAGAAGAAATATTGCAGTGAATGAAGATTCCTCTGCATTTTAGCACTGCTTTTTCAACTGT
AGTTGGCTTTTGAATGAGGATGACAATGGAAGAGATGAAGAATGAAGCTGAGACCACATCCATGGTTTCT
ATGCCCCTCTATGCAGTCATGTATCCTGTGTTTAATGAGCTAGAACGAGTAAATCTGTCTGCAGCCCAGA
CACTGAGAGCCGCTTTCATCAAGGCTGAAAAAGAAAATCCAGGTCTCACACAAGACATCATTATGAAAAT
TTTAGAGAAAAAAAGCGTGGAAGTTAACTTCACGGAGTCCCTTCTTCGTATGGCAGCTGATGATGTAGAA
GAGTATATGATTGAACGACCAGAGCCAGAATTCCAAGACCTAAACGAAAAGGCACGAGCACTTAAACAAA
TTCTCAGTAAGATCCCAGATGAGATCAATGACAGAGTGAGGTTTCTGCAGACAATCAAGGATATAGCTAG
TGCAATAAAAGAACTTCTTGATACAGTGAATAATGTCTTCAAGAAATATCAATACCAGAACCGCAGGGCA
CTTGAACACCAAAAGAAAGAATTTGTAAAGTACTCCAAAAGTTTCAGTGATACTCTGAAAACGTATTTTA
AAGATGGCAAGGCAATAAATGTGTTCGTAAGTGCCAACCGACTAATTCATCAAACCAACTTAATACTTCA
GACCTTCAAAACTGTGGCCTGAAAGTTGTATATGTTAAGAGATGTACTTCTCAGTGGCAGTATTGAACTG
CCTTTATCTGTAAATTTTAAAGTTTGACTGTATAAATTATCAGTCCCTCCTGAAGGGATCTAATCCAGGA
TGTTGAATGGGATTATTGCCATCTTACACCATATTTTTGTAAAATGTAGCTTAATCATAATCTCACACTG
AAGATTTTGCATCACTTTTGCTATTATCATTCTTTTAAGAATTATAAGCCAAAAGAATTTACGCCTTAAT
GTGTCATTATATAACATTCCTTAAAAGAATTGTAAATATTGGTGTTTGTTTCTGACATTTTAACTTGAAA
GCGATATGCTGCAAGATAATGTATTTAACAATATTTGGTGGCAAATATTCAATAAATAGTTTACATCTGT
TAAAAAAAAAAAAAAAAAAAAA
>NP_665859.1 programmed cell death protein 10 [Homo sapiens]
MRMTMEEMKNEAETTSMVSMPLYAVMYPVFNELERVNLSAAQTLRAAFIKAEKENPGLTQDIIMKILEKK
SVEVNFTESLLRMAADDVEEYMIERPEPEFQDLNEKARALKQILSKIPDEINDRVRFLQTIKDIASAIKE
LLDTVNNVFKKYQYQNRRALEHQKKEFVKYSKSFSDTLKTYFKDGKAINVFVSANRLIHQTNLILQTFKT
VA
```

```
>NM_007217.3 Homo sapiens programmed cell death 10 (PDCD10), transcript variant 1, mRNA
GGTAAGGTGTAGCTGTTCTGTCCCAGTCCCCGTAGCTCTGCACCGAGCAGAAGAGGTCTAGGGTCACGCA
GGGGCCATGGCTGAGGCACTTGGGCTTGGTTCTGGCAGGCGGAGCGGACGGGGCTGAGAGCGGGGCCCTA
CGGGCGGAAGAGGAGCACTGGAAGAAGGAAGAGACAAATGTTGGGGTCTACGGATTCAGTTCCAGTTTAA
TGATGTGGCCCTCTAAGGAGATAATTTTGCCTTAAACAGAAAGACTTGACCCTCTTAGGTGTCATACCTA
AGATCAGTGTGTTTTTTGTGTCCAATTCTTTTATCACCAAAAAAGAGAAGAAATATTGCAGTGAATGAAG
ATTCCTCTGCATTTTAGCACTGCTTTTTCAACTGTAGTTGGCTTTTGAATGAGGATGACAATGGAAGAGA
TGAAGAATGAAGCTGAGACCACATCCATGGTTTCTATGCCCCTCTATGCAGTCATGTATCCTGTGTTTAA
TGAGCTAGAACGAGTAAATCTGTCTGCAGCCCAGACACTGAGAGCCGCTTTCATCAAGGCTGAAAAAGAA
AATCCAGGTCTCACACAAGACATCATTATGAAAATTTTAGAGAAAAAAAGCGTGGAAGTTAACTTCACGG
AGTCCCTTCTTCGTATGGCAGCTGATGATGTAGAAGAGTATATGATTGAACGACCAGAGCCAGAATTCCA
AGACCTAAACGAAAAGGCACGAGCACTTAAACAAATTCTCAGTAAGATCCCAGATGAGATCAATGACAGA
GTGAGGTTTCTGCAGACAATCAAGGATATAGCTAGTGCAATAAAAGAACTTCTTGATACAGTGAATAATG
TCTTCAAGAAATATCAATACCAGAACCGCAGGGCACTTGAACACCAAAAGAAAGAATTTGTAAAGTACTC
CAAAAGTTTCAGTGATACTCTGAAAACGTATTTTAAAGATGGCAAGGCAATAAATGTGTTCGTAAGTGCC
AACCGACTAATTCATCAAACCAACTTAATACTTCAGACCTTCAAAACTGTGGCCTGAAAGTTGTATATGT
TAAGAGATGTACTTCTCAGTGGCAGTATTGAACTGCCTTTATCTGTAAATTTTAAAGTTTGACTGTATAA
ATTATCAGTCCCTCCTGAAGGGATCTAATCCAGGATGTTGAATGGGATTATTGCCATCTTACACCATATT
TTTGTAAAATGTAGCTTAATCATAATCTCACACTGAAGATTTTGCATCACTTTTGCTATTATCATTCTTT
TAAGAATTATAAGCCAAAAGAATTTACGCCTTAATGTGTCATTATATAACATTCCTTAAAAGAATTGTAA
ATATTGGTGTTTGTTTCTGACATTTTAACTTGAAAGCGATATGCTGCAAGATAATGTATTTAACAATATT
TGGTGGCAAATATTCAATAAATAGTTTACATCTGTTAAAAAAAAAAAAAAAAAA
```

```
>NP_009148.2 programmed cell death protein 10 [Homo sapiens]
MRMTMEEMKNEAETTSMVSMPLYAVMYPVFNELERVNLSAAQTLRAAFIKAEKENPGLTQDIIMKILEKK
SVEVNFTESLLRMAADDVEEYMIERPEPEFQDLNEKARALKQILSKIPDEINDRVRFLQTIKDIASAIKE
LLDTVNNVFKKYQYQNRRALEHQKKEFVKYSKSFSDTLKTYFKDGKAINVFVSANRLIHQTNLILQTFKT
VA
```

```
NM_007217.3                          GGTAAGGTGTAGCTGTTCTGTCCCAGTCCCCGTAGCTCTGCACCGAGCAGAAGAGGTCTA  60
i0_HQ_samplec9816d|c43184/f2p1/954   ------------------------------------------------------------  0
NM_145859.1                          ------------------------------------------------------------  0
```

```
NM_145860.1                         ------------------------------------------------------------ 0

NM_007217.3                         GGGTCACGCAGGGGCCATGGCTGAGGCACTTGGGCTTGGTTCTGGCAGGCGGAGCGGACG 120
i0_HQ_samplec9816d|c43184/f2p1/954  ------------------------------------------------------------ 0
NM_145859.1                         ------------------------------------------------------------ 0
NM_145860.1                         ------------------------------------------------------------ 0

NM_007217.3                         GGGCTGAGAGCGGGGCCCTACGGGCGGAAGAGGAGCACTGGAAGAAGGAAGAGACAAATG 180
i0_HQ_samplec9816d|c43184/f2p1/954  ------------------------------------------------------------ 0
NM_145859.1                         ----------------GA-TCCGGGAGTTGAAGAGGGCTGCAAGGTGGG---AAGTGAAG 40
NM_145860.1                         ------------------------------------------------------------ 0

NM_007217.3                         TTGGGGTCTACGGATTCAGTTCCAGTTTAATGATGTGGCCCTCTAAGGAGATAATTTTGC 240
i0_HQ_samplec9816d|c43184/f2p1/954  ------------------------------------------------------------ 0
NM_145859.1                         TCAG-------TGCCTCAGTTGCTGCTTTCTCCTCTA--AATCTTGGTCTCTTGTTTGG- 90
NM_145860.1                         ------------------------------------------------------------ 0

NM_007217.3                         CTTAAACAGAAAGACTTGACCCTCTTAGGTGTCATACCTAAGATCAGTGTGTTTTTTGTG 300
i0_HQ_samplec9816d|c43184/f2p1/954  ------------------------------------------------------------ 0
NM_145859.1                         -AAGTGACTAACAGCATTTTGGCATGAGAAAGCCCTAAAAAGATCAGTGTGTTTTTTGTG 149
NM_145860.1                         -----TGCAAGGTGGGAAGTGAAGTCAGTGCCTCAGTTGCTGATCAGTGTGTTTTTTGTG 55

NM_007217.3                         TCCAATTCTTTTATCACCAAAAAAGAGAAGAAATATTGCAGTGAATGAAGATTCCTCTGC 360
i0_HQ_samplec9816d|c43184/f2p1/954  ------------------------------------------------------------ 0
NM_145859.1                         TCCAATTCTTTTATCACCAAAAAAGAGAAGAAATATTGCAGTGAATGAAGATTCCTCTGC 209
NM_145860.1                         TCCAATTCTTTTATCACCAAAAAAGAGAAGAAATATTGCAGTGAATGAAGATTCCTCTGC 115

NM_007217.3                         ATTTTAGCACTGCTTTTTCAACTGTAGTTGGCTTTTGAATGAGGATGACAATGGAAGAGA 420
i0_HQ_samplec9816d|c43184/f2p1/954  ------------------------------------------------------------ 0
NM_145859.1                         ATTTTAGCACTGCTTTTTCAACTGTAGTTGGCTTTTGAATGAGGATGACAATGGAAGAGA 269
NM_145860.1                         ATTTTAGCACTGCTTTTTCAACTGTAGTTGGCTTTTGAATGAGGATGACAATGGAAGAGA 175

NM_007217.3                         TGAAGAATGAAGCTGAGACCACATCCATGGTTTCTATGCCCCTCTATGCAGTCATGTATC 480
i0_HQ_samplec9816d|c43184/f2p1/954  ------------------------------------------------------------ 0
NM_145859.1                         TGAAGAATGAAGCTGAGACCACATCCATGGTTTCTATGCCCCTCTATGCAGTCATGTATC 329
NM_145860.1                         TGAAGAATGAAGCTGAGACCACATCCATGGTTTCTATGCCCCTCTATGCAGTCATGTATC 235

NM_007217.3                         CTGTGTTTAATGAGCTAGAACGAGTAAATCTGTCTGC---AGCCCAGACACTGAGAGCCG 537
i0_HQ_samplec9816d|c43184/f2p1/954  -ggagtgaattgat-ccgggagtttgaagagggctgcaaggtgggaagtgaagtcagtgc 58
NM_145859.1                         CTGTGTTTAATGAGCTAGAACGAGTAAATCTGTCTGC---AGCCCAGACACTGAGAGCCG 386
NM_145860.1                         CTGTGTTTAATGAGCTAGAACGAGTAAATCTGTCTGC---AGCCCAGACACTGAGAGCCG 292
                                      * **  * ***    *   *  * **   * ****        *      *  **

NM_007217.3                         CTTTCATCAAGGCTGAAAAAGAAAATCCAGGTCTCACACAAGACATCATTATGAAAATTT 597
i0_HQ_samplec9816d|c43184/f2p1/954  ctcagttgctGGCTGAAAAAGAAAATCCAGGTCTCACACAAGACATCATTATGAAAATTT 118
NM_145859.1                         CTTTCATCAAGGCTGAAAAAGAAAATCCAGGTCTCACACAAGACATCATTATGAAAATTT 446
NM_145860.1                         CTTTCATCAAGGCTGAAAAAGAAAATCCAGGTCTCACACAAGACATCATTATGAAAATTT 352
                                    **    *   **************************************************

NM_007217.3                         TAGAGAAAAAAAGCGTGGAAGTTAACTTCACGGAGTCCCTTCTTCGTATGGCAGCTGATG 657
i0_HQ_samplec9816d|c43184/f2p1/954  TAGAGAAAAAAAGCGTGGAAGTTAACTTCACGGAGTCCCTTCTTCGTATGGCAGCTGATG 178
NM_145859.1                         TAGAGAAAAAAAGCGTGGAAGTTAACTTCACGGAGTCCCTTCTTCGTATGGCAGCTGATG 506
NM_145860.1                         TAGAGAAAAAAAGCGTGGAAGTTAACTTCACGGAGTCCCTTCTTCGTATGGCAGCTGATG 412
                                    ************************************************************

NM_007217.3                         ATGTAGAAGAGTATATGATTGAACGACCAGAGCCAGAATTCCAAGACCTAAACGAAAAGG 717
i0_HQ_samplec9816d|c43184/f2p1/954  ATGTAGAAGAGTATATGATTGAACGACCAGAGCCAGAATTCCAAGACCTAAACGAAAAGG 238
NM_145859.1                         ATGTAGAAGAGTATATGATTGAACGACCAGAGCCAGAATTCCAAGACCTAAACGAAAAGG 566
NM_145860.1                         ATGTAGAAGAGTATATGATTGAACGACCAGAGCCAGAATTCCAAGACCTAAACGAAAAGG 472
                                    ************************************************************

NM_007217.3                         CACGAGCACTTAAACAAATTCTCAGTAAGATCCCAGATGAGATCAATGACAGAGTGAGGT 777
i0_HQ_samplec9816d|c43184/f2p1/954  CACGAGCACTTAAACAAATTCTCAGTAAGATCCCAGATGAGATCAATGACAGAGTGAGGT 298
NM_145859.1                         CACGAGCACTTAAACAAATTCTCAGTAAGATCCCAGATGAGATCAATGACAGAGTGAGGT 626
NM_145860.1                         CACGAGCACTTAAACAAATTCTCAGTAAGATCCCAGATGAGATCAATGACAGAGTGAGGT 532
                                    ************************************************************

NM_007217.3                         TTCTGCAGACAATCAAGGATATAGCTAGTGCAATAAAAGAACTTCTTGATACAGTGAATA 837
i0_HQ_samplec9816d|c43184/f2p1/954  TTCTGCAGACAATCAAGGATATAGCTAGTGCAATAAAAGAACTTCTTGATACAGTGAATA 358
NM_145859.1                         TTCTGCAGACAATCAAGGATATAGCTAGTGCAATAAAAGAACTTCTTGATACAGTGAATA 686
NM_145860.1                         TTCTGCAGACAATCAAGGATATAGCTAGTGCAATAAAAGAACTTCTTGATACAGTGAATA 592
                                    ************************************************************

NM_007217.3                         ATGTCTTCAAGAAATATCAATACCAGAACCGCAGGGCACTTGAACACCAAAAGAAAGAAT 897
```

```
i0_HQ_samplec9816d|c43184/f2p1/954      ATGTCTTCAAGAAATATCAATACCAGAACCGCAGGGCACTTGAACACCAAAAGAAAGAAT  418
NM_145859.1                             ATGTCTTCAAGAAATATCAATACCAGAACCGCAGGGCACTTGAACACCAAAAGAAAGAAT  746
NM_145860.1                             ATGTCTTCAAGAAATATCAATACCAGAACCGCAGGGCACTTGAACACCAAAAGAAAGAAT  652
                                        ************************************************************

NM_007217.3                             TTGTAAAGTACTCCAAAAGTTTCAGTGATACTCTGAAAACGTATTTTAAAGATGGCAAGG  957
i0_HQ_samplec9816d|c43184/f2p1/954      TTGTAAAGTACTCCAAAAGTTTCAGTGATACTCTGAAAACGTATTTTAAAGATGGCAAGG  478
NM_145859.1                             TTGTAAAGTACTCCAAAAGTTTCAGTGATACTCTGAAAACGTATTTTAAAGATGGCAAGG  806
NM_145860.1                             TTGTAAAGTACTCCAAAAGTTTCAGTGATACTCTGAAAACGTATTTTAAAGATGGCAAGG  712
                                        ************************************************************

NM_007217.3                             CAATAAATGTGTTCGTAAGTGCCAACCGACTAATTCATCAAACCAACTTAATACTTCAGA  1017
i0_HQ_samplec9816d|c43184/f2p1/954      CAATAAATGTGTTCGTAAGTGCCAACCGACTAATTCATCAAACCAACTTAATACTTCAGA  538
NM_145859.1                             CAATAAATGTGTTCGTAAGTGCCAACCGACTAATTCATCAAACCAACTTAATACTTCAGA  866
NM_145860.1                             CAATAAATGTGTTCGTAAGTGCCAACCGACTAATTCATCAAACCAACTTAATACTTCAGA  772
                                        ************************************************************

NM_007217.3                             CCTTCAAAACTGTGGCCTGAAAGTTGTATATGTTAAGAGATGTACTTCTCAGTGGCAGTA  1077
i0_HQ_samplec9816d|c43184/f2p1/954      CCT-CAAAACTGTGGCCTGAAAGTTGTATATGTTAAGAGATGTACTTCTCAGTGGCAGTA  597
NM_145859.1                             CCTTCAAAACTGTGGCCTGAAAGTTGTATATGTTAAGAGATGTACTTCTCAGTGGCAGTA  926
NM_145860.1                             CCTTCAAAACTGTGGCCTGAAAGTTGTATATGTTAAGAGATGTACTTCTCAGTGGCAGTA  832
                                        *** ********************************************************

NM_007217.3                             TTGAACTGCCTTTATCTGTAAATTTTAAAGTTTGACTGTATAAATTATCAGTCCCTCCTG  1137
i0_HQ_samplec9816d|c43184/f2p1/954      TTGAACTGCCTTTATCTGTAAATTTTAAAGTTTGACTGTATAAATTATCAGTCCCTCCTG  657
NM_145859.1                             TTGAACTGCCTTTATCTGTAAATTTTAAAGTTTGACTGTATAAATTATCAGTCCCTCCTG  986
NM_145860.1                             TTGAACTGCCTTTATCTGTAAATTTTAAAGTTTGACTGTATAAATTATCAGTCCCTCCTG  892
                                        ************************************************************

NM_007217.3                             AAGGGATCTAATCCAGGATGTTGAATGGGATTATTGCCATCTTACACCATATTTTTGTAA  1197
i0_HQ_samplec9816d|c43184/f2p1/954      AAGGGATCTAATCCAGGATGTTGAATGGGATTATTGCCATCTTACACCATATTTTTGTAA  717
NM_145859.1                             AAGGGATCTAATCCAGGATGTTGAATGGGATTATTGCCATCTTACACCATATTTTTGTAA  1046
NM_145860.1                             AAGGGATCTAATCCAGGATGTTGAATGGGATTATTGCCATCTTACACCATATTTTTGTAA  952
                                        ************************************************************

NM_007217.3                             AATGTAGCTTAATCATAATCTCACACTGAAGATTTTGCATCACTTTTGCTATTATCATTC  1257
i0_HQ_samplec9816d|c43184/f2p1/954      AATGTAGCTTAATCATAATCTCACACTGAAGATTTTGCATCACTTTTGCTATTATCATTC  777
NM_145859.1                             AATGTAGCTTAATCATAATCTCACACTGAAGATTTTGCATCACTTTTGCTATTATCATTC  1106
NM_145860.1                             AATGTAGCTTAATCATAATCTCACACTGAAGATTTTGCATCACTTTTGCTATTATCATTC  1012
                                        ************************************************************

NM_007217.3                             TTTTAAGAATTATAAGCCAAAAGAATTTACGCCTTAATGTGTCATTATATAACATTCCTT  1317
i0_HQ_samplec9816d|c43184/f2p1/954      TTTTAAGAATTATAAGCCAAAAGAATTTACGCCTTAATGTGTCATTATATAACATTCCTT  837
NM_145859.1                             TTTTAAGAATTATAAGCCAAAAGAATTTACGCCTTAATGTGTCATTATATAACATTCCTT  1166
NM_145860.1                             TTTTAAGAATTATAAGCCAAAAGAATTTACGCCTTAATGTGTCATTATATAACATTCCTT  1072
                                        ************************************************************

NM_007217.3                             AAAAGAATTGTAAATATTGGTGTTTGTTTCTGACATTTTAACTTGAAAGCGATATGCTGC  1377
i0_HQ_samplec9816d|c43184/f2p1/954      AAAAGAATTGTAAATATTGGTGTTTGTTTCTGACATTTTAACTTGAAAGCGATATGCTGC  897
NM_145859.1                             AAAAGAATTGTAAATATTGGTGTTTGTTTCTGACATTTTAACTTGAAAGCGATATGCTGC  1226
NM_145860.1                             AAAAGAATTGTAAATATTGGTGTTTGTTTCTGACATTTTAACTTGAAAGCGATATGCTGC  1132
                                        ************************************************************

NM_007217.3                             AAGATAATGTATTTAACAATATTTGGTGGCAAATATTCAATAAATAGTTTACATCTGTTA  1437
i0_HQ_samplec9816d|c43184/f2p1/954      AAGATAATGTATTTAACAATATTTGGTGGCAAATATTCAATAAATAGTTTACATCTG---  954
NM_145859.1                             AAGATAATGTATTTAACAATATTTGGTGGCAAATATTCAATAAATAGTTTACATCTGTTA  1286
NM_145860.1                             AAGATAATGTATTTAACAATATTTGGTGGCAAATATTCAATAAATAGTTTACATCTGTTA  1192
                                        *********************************************************

NM_007217.3                             AAAAAAAAAAAAAAAAA----------    1454
i0_HQ_samplec9816d|c43184/f2p1/954      ---------------------------    954
NM_145859.1                             AAAAAAAAAAAAAAAAAAAAAAAAAAA    1313
NM_145860.1                             AAAAAAAAAAAAAAAAAAAA-------    1212

VIRT111671         ------------------------------------------------------------    0
NP_665858.1        MRMTMEEMKNEAETTSMVSMPLYAVMYPVFNELERVNLSAAQTLRAAFIKAEKENPGLTQ    60
NP_665859.1        MRMTMEEMKNEAETTSMVSMPLYAVMYPVFNELERVNLSAAQTLRAAFIKAEKENPGLTQ    60
NP_009148.2        MRMTMEEMKNEAETTSMVSMPLYAVMYPVFNELERVNLSAAQTLRAAFIKAEKENPGLTQ    60


VIRT111671         ---MKILEKKSVEVNFTESLLRMAADDVEEYMIERPEPEFQDLNEKARALKQILSKIPDE    57
NP_665858.1        DIIMKILEKKSVEVNFTESLLRMAADDVEEYMIERPEPEFQDLNEKARALKQILSKIPDE    120
NP_665859.1        DIIMKILEKKSVEVNFTESLLRMAADDVEEYMIERPEPEFQDLNEKARALKQILSKIPDE    120
NP_009148.2        DIIMKILEKKSVEVNFTESLLRMAADDVEEYMIERPEPEFQDLNEKARALKQILSKIPDE    120
                      *********************************************************

VIRT111671         INDRVRFLQTIKDIASAIKELLDTVNNVFKKYQYQNRRALEHQKKEFVKYSKSFSDTLKT    117
NP_665858.1        INDRVRFLQTIKDIASAIKELLDTVNNVFKKYQYQNRRALEHQKKEFVKYSKSFSDTLKT    180
NP_665859.1        INDRVRFLQTIKDIASAIKELLDTVNNVFKKYQYQNRRALEHQKKEFVKYSKSFSDTLKT    180
NP_009148.2        INDRVRFLQTIKDIASAIKELLDTVNNVFKKYQYQNRRALEHQKKEFVKYSKSFSDTLKT    180
                   ************************************************************
```

```
VIRT111671        YFKDGKAINVFVSANRLIHQTNLILQTSKLWPESCIC      154
NP_665858.1       YFKDGKAINVFVSANRLIHQTNLILQTFKTVA-----      212
NP_665859.1       YFKDGKAINVFVSANRLIHQTNLILQTFKTVA-----      212
NP_009148.2       YFKDGKAINVFVSANRLIHQTNLILQTFKTVA-----      212
                  *************************** *
```

**SPRR4**

| Sequence | Header | AA Seq |
|---|---|---|
| **Present** | i0_HQ_samplec9816d\|c2034/f4p1/662 | |
| | i0_HQ_samplec9816d\|c1580/f7p10/768 | |
| **Absent** | | |
| **NCBI** | NM_173080 | |
| | | |

>i0_HQ_samplec9816d|c2034/f4p1/662
isoform=c2034;full_length_coverage=4;non_full_length_coverage=1;isoform_length=662

agagcagggggcatcatggagaggaatgaggcagaaaCTGTGCACCAGCCTCTGTGACCCTACTTGTCAGGGTGGAACCCCAGAAG
AAGCCCAGACACTTTCCTCACTTTGGGAAATGGATGGGCCTTAGCTGTGGCCACGCTGGAGCCTGCAGAGAATGAGGA
GGAGATGTGAGAGGAGGGTAGGCTCAAGAGCTGGAGGGAAGAGGAGAAGGAAGAAGAGAAGGTTCCATCTGGTAG
CCAGGATGGTGGATGATGGCCAGACCTCCCCCACTTCCTCTTCCCAAGCCTAGCAACACTAAGAATGTAATATTCTGGGT
TCCAGATTCTGGTTCTGCATTCCACCTCCAAGAAGAACACCATCTCCACCTGCAGCCCTCACTTCCACATACCCCCAGTCC
TCTGTGCTCTTGCTGATATGAACTCTCCACAAGGAAACTACGCAGCAAGACAGATTTGCATGGACATAGACCAAGGCTT
TTCATATACCTGGTGCCTTGAAGGCACTGGTATGTGTGTATTGCCTGTGAACAGATGGGCAGCTCCATCCCTCATGTCCC
AGTCACCCATGTGGGTTTACTGCAATGAATTTTACTCCTATTTTTCCCAATTTCATCATTGCCCTTTTAtttattaataaaggact
cctgcttagttttgaag

>VIRT53999

MMARPPPLPLPKPSNTKNVIFWVPDSGSAFHLQEEHHLHLQPSLPHTPSPLCSC

>i0_HQ_samplec9816d|c1580/f7p10/768
isoform=c1580;full_length_coverage=7;non_full_length_coverage=10;isoform_length=768

ggacaagggctcagcctcctctcctggggtccagcttgTCGCCTCTGGCTCACCTGTTCCTAGAGCAATGTCTTCCCAGCAGCAGCAGC
GGCAGCAGCAGCAGTGCCCACCCCAGAGGGCCCAGCAGCAGCAAGTGAAGCAGCCTTGTCAGCCACCCCCTGTTAAAT
GTCAAGAGACATGTGCACCCAAAACCAAGGATCCATGTGCTCCCCAGGTCAAGAAGCAATGCCCACCGAAAGGCACCA
TCATTCCAGCCCAGCAGAAGTGTCCCTCAGCCCAGCAAGCCTCCAAGAGCAAACAGAAGTAAGGATGGACTGGATATT
ACCATCATCCACCATCCTGGCTACCAGATGGAACCTTCTCTTCTTCCTTCTCCTCTTCCCTCCAGCTCTTGAGCCTACCCTC
CTCTCACATCTCCTCCTGCCCAAGATGTAAGGAAGCATTGTAAGGATTTCTTCCCATCGTACCCTTCCCCACACATACCAC
CTTGGCTTCTTCTATATCCCACCCCGATGCTCTCCCAGGTGGGTGTGAGAGAGACCTCATTCTCTGCAGGCTCCAGCGTG
GCCACAGCTAAGGCCCATCCATTTCCCAAAGTGAGGAAAGTGTCTGGGCTTCTTCTGGGGTTCCACCCTGACAAGTAGG
GTCACAGAGGCTGGTGCACAGTTTCTGCCTCATTCCTCTCCATGATGCCCCCTGCTCTGGGCTTCTCTCCTGTTTTCCCCA
ATAAATATGTGCCTCATGTAATAAATGTGTCTGCTTCCTGGG

>VIRT55277

```
MLPYILGRRRCERRVGSRAGGKRRRKKRRFHLVARMVDDGNIQSILTSVCSWRLAGLRDT
SAGLE
```

```
>NM_173080.2 Homo sapiens small proline rich protein 4 (SPRR4), mRNA
AGGGCTCAGCCTCCTCTCCTGGGGTCCAGCTTGTCGCCTCTGGCTCACCTGTTCCTAGAGCAATGTCTTC
CCAGCAGCAGCAGCGGCAGCAGCAGCAGTGCCCACCCCAGAGGGCCCAGCAGCAGCAAGTGAAGCAGCCT
TGTCAGCCACCCCCTGTTAAATGTCAAGAGACATGTGCACCCAAAACCAAGGATCCATGTGCTCCCCAGG
TCAAGAAGCAATGCCCACCGAAAGGCACCATCATTCCAGCCCAGCAGAAGTGTCCCTCAGCCCAGCAAGC
CTCCAAGAGCAAACAGAAGTAAGGATGGACTGGATATTACCATCATCCACCATCCTGGCTACCAGATGGA
ACCTTCTCTTCTTCCTTCTCCTCTTCCCTCCAGCTCTTGAGCCTACCCTCCTCTCACATCTCCTCCTGCC
CAAGATGTAAGGAAGCATTGTAAGGATTTCTTCCCATCGTACCCTTCCCCACACATACCACCTTGGCTTC
TTCTATATCCCACCCCGATGCTCTCCCAGGTGGGTGTGAGAGAGACCTCATTCTCTGCAGGCTCCAGCGT
GGCCACAGCTAAGGCCCATCCATTTCCCAAAGTGAGGAAAGTGTCTGGGCTTCTTCTGGGGTTCCACCCT
GACAAGTAGGGTCACAGAGGCTGGTGCACAGTTTCTGCCTCATTCCTCTCCATGATGCCCCCTGCTCTGG
GCTTCTCTCCTGTTTTCCCCAATAAATATGTGCCTCATGTAATAAAAAAAAAA
>NP_775103.1 small proline-rich protein 4 [Homo sapiens]
MSSQQQQRQQQQCPPQRAQQQQVKQPCQPPPVKCQETCAPKTKDPCAPQVKKQCPPKGTIIPAQQKCPSA
QQASKSKQK
```

```
i0_HQ_samplec9816d|c2034/f4p1/662       ------------------------------------------------------------  0
i0_HQ_samplec9816d|c1580/f7p10/768      ggacaagggctcagcctcctctcctggggtccagcttgTCGCCTCTGGCTCACCTGTTCC  60
NM_173080.2                             -----AGGGCTCAGCCTCCTCTCCTGGGGTCCAGCTTGTCGCCTCTGGCTCACCTGTTCC  55

i0_HQ_samplec9816d|c2034/f4p1/662       -ag-----------------------------------agcaggggcatcatggagagg  24
i0_HQ_samplec9816d|c1580/f7p10/768      TAGAGCAATGTCTTCCCAGCAGCAGCAGCGGCAGCAGCAGCAGTGCCCACCCCAGAGGGC  120
NM_173080.2                             TAGAGCAATGTCTTCCCAGCAGCAGCAGCGGCAGCAGCAGCAGTGCCCACCCCAGAGGGC  115
                                         **                                   ***** *  ** *   *** *

i0_HQ_samplec9816d|c2034/f4p1/662       aatgaggcagaaaC-----------TGTGCACCAGCCTCTGTGACCCTACTTGTCAGGGT  73
i0_HQ_samplec9816d|c1580/f7p10/768      CCAGCAGCAGCAAGTGAAGCAGCCTTGTCAGCCACCCCCTGTTAAATGTCAAGAGACATG  180
NM_173080.2                             CCAGCAGCAGCAAGTGAAGCAGCCTTGTCAGCCACCCCCTGTTAAATGTCAAGAGACATG  175
                                           *  ****  **            ***   ***  **  ****  *     *  *  *

i0_HQ_samplec9816d|c2034/f4p1/662       GGAACCCCAGAAGAAGCCCAGACACTTTCCTCACTTTGGGAAATGGATGGGCCTTAGCTG  133
i0_HQ_samplec9816d|c1580/f7p10/768      TGCACCCAAAACCAAGGATCCATGTGCTCCCCAGGTCAAGAAG-CAATGC---CCACCGA  236
NM_173080.2                             TGCACCCAAAACCAAGGATCCATGTGCTCCCCAGGTCAAGAAG-CAATGC---CCACCGA  231
                                         * **** * *  ***     *     *** **  *   ***    ***      * *

i0_HQ_samplec9816d|c2034/f4p1/662       TGGCCACGCTGGAGCCTGCAGAGAATGAGGAGGAGATGTGAGAGGAGGGTAGGCTCAAGA  193
i0_HQ_samplec9816d|c1580/f7p10/768      AAGGCACCATCATTCCAGCCCA---GCAGAAGTGTCCCTCAGCCCAGCAAGCCTCCAAGA  293
NM_173080.2                             AAGGCACCATCATTCCAGCCCA---GCAGAAGTGTCCCTCAGCCCAGCAAGCCTCCAAGA  288
                                          * ***  *    ** **  *     ** **      * **   **        *****

i0_HQ_samplec9816d|c2034/f4p1/662       GCTGGAGGGAAGAGGAGAAGGAAGAAGAGAAGGTTCCATCTGG---TAGCCAGGATGGTG  250
i0_HQ_samplec9816d|c1580/f7p10/768      GCAA-ACAGAAGTAAGG---ATGGACTGGATATTACCATCATCCACCATCCTGGCTACCA  349
NM_173080.2                             GCAA-ACAGAAGTAAGG---ATGGACTGGATATTACCATCATCCACCATCCTGGCTACCA  344
                                        **   *  ****    *      **   **   * *****       * ** ** *

i0_HQ_samplec9816d|c2034/f4p1/662       GATGATGGCCAGACCTCCCCCACTTCCTCTTCCCAAGCCTAGCAACACTAAGAATGTAAT  310
i0_HQ_samplec9816d|c1580/f7p10/768      GATGGAACCTTCTCTTCTTCCTTCTCCTCTTCCCTCC-----AGCTCTTGAGCCTACCCT  404
NM_173080.2                             GATGGAACCTTCTCTTCTTCCTTCTCCTCTTCCCTCC-----AGCTCTTGAGCCTACCCT  399
                                        ****    *    * **  **   **********              * **  *    *

i0_HQ_samplec9816d|c2034/f4p1/662       ATTCTGGGTTCCAGATTCTGGTTCTGCATTCCACCTC-----CAAGAAGAACACCATCTC  365
i0_HQ_samplec9816d|c1580/f7p10/768      C-------CTCTCACATCTCCTCCTGCCCAAGATGTAAGGAAGCATTGTAAGGATTTCTT  457
NM_173080.2                             C-------CTCTCACATCTCCTCCTGCCCAAGATGTAAGGAAGCATTGTAAGGATTTCTT  452
                                                 **     *** * ****      *  *        *    **     ***

i0_HQ_samplec9816d|c2034/f4p1/662       CACCTGCAGCCCTCACTTCCACATACCCCCAGTCCTCTGTGCTCTTGCTGATATGAACTC  425
i0_HQ_samplec9816d|c1580/f7p10/768      CCCATCGTACCCTTCCCCACACATACCACCTTGGCTT-CTTCTATATCCCACCCCGATGC  516
NM_173080.2                             CCCATCGTACCCTTCCCCACACATACCACCTTGGCTT-CTTCTATATCCCACCCCGATGC  511
                                        * * *    ****  *   ******** **    **   * ** *  *  *     *  *

i0_HQ_samplec9816d|c2034/f4p1/662       TCCACAAGGAAACTACGCAGCAAGACAGA------------TTTGCATGGACATAGACC  472
i0_HQ_samplec9816d|c1580/f7p10/768      TCTCCCAGGTGGGTGTGAGAGAGACCTCATTCTCTGCAGGCTCCAGCGTGGCCACAGCTA  576
NM_173080.2                             TCTCCCAGGTGGGTGTGAGAGAGACCTCATTCTCTGCAGGCTCCAGCGTGGCCACAGCTA  571
                                        **  * ***    *  *    *   *  *                **  *** ** **

i0_HQ_samplec9816d|c2034/f4p1/662       AAGGCTTTTCATATACCTGGTGCCTTGAAGGCACTG------GTATGTGTGTATTGCCTG  526
i0_HQ_samplec9816d|c1580/f7p10/768      AGGCCCATCCATTTCCCAAAGTGAGGAAAGTGTCTGGGCTTCTTCTGGGGTTCCACCCTG  636
NM_173080.2                             AGGCCCATCCATTTCCCAAAGTGAGGAAAGTGTCTGGGCTTCTTCTGGGGTTCCACCCTG  631
                                        * * *  * *** * **          ***   ***       * ** *  *    ****

i0_HQ_samplec9816d|c2034/f4p1/662       T-------GAACAGATGGGCAGCTCCATCCCTCATGTCCCAGTCACCCA-TGTGGGTTTA  578
i0_HQ_samplec9816d|c1580/f7p10/768      ACAAGTAGGGTCACAGAGGCTGGTGCACAGTTTCTGCCTCATTCCTCTCCATGATGCCCC  696
NM_173080.2                             ACAAGTAGGGTCACAGAGGCTGGTGCACAGTTTCTGCCTCATTCCTCTCCATGATGCCCC  691
```

```
                                                                 *   ** *   *** * * **     *   ** * ** **   *         *

i0_HQ_samplec9816d|c2034/f4p1/662      CTGCAATGAATTTTACTCCTATTTTTCCCAATTTCATCATTGCCCTTTTAtttattaata  638
i0_HQ_samplec9816d|c1580/f7p10/768     CTGCTCTGGGCTTCTCTCCTGTTTTCCCCAATAAATATGTGCCTCATGTAATAAA-----  751
NM_173080.2                            CTGCTCTGGGCTTCTCTCCTGTTTTCCCCAATAAATATGTGCCTCATGTAATAAA-----  746
                                       ****  **   **  ***** **** ******        *  * * * ** * *

i0_HQ_samplec9816d|c2034/f4p1/662      aaggactcctgcttagttttgaag      662
i0_HQ_samplec9816d|c1580/f7p10/768     ---TGTGTCTGCTTCCTGGG----      768
NM_173080.2                            ---AAAAAAA--------------      753
```

```
VIRT55277       ---------------------------MLPYILGRRRCERRVGSRAGGKRRRKKRRFHLV      33
VIRT53999       -----------------------MMARPPPLPLPKPSNTK----------------NVI      20
NP_775103.1     MSSQQQQRQQQQCPPQRAQQQQVKQPCQPPPVKCQETCAPKTKDPCA---------PQVK      51
                                             * :    . .   :                ::

VIRT55277       ARMVDDGNIQ---------------SILTSVCSWRLAGLRDTSAGLE      65
VIRT53999       FWVPDSGSAFHLQEEHHLHLQPSLPHTPSPLCSC-------------      54
NP_775103.1     KQCPPKGTIIPAQQKCPSAQQASKSKQK-------------------      79
```

**JAGN1**

| Sequence | Header | AA Seq |
|---|---|---|
| **Present** | i1_HQ_samplec9816d\|c7012/f2p0/1248 | |
| | | |
| **Absent** | i1_HQ_samplec9816d\|c64100/f41p0/1184 | |
| **NCBI** | NM_032492 | |
| | | |

>i1_HQ_samplec9816d|c7012/f2p0/1248
isoform=c7012;full_length_coverage=2;non_full_length_coverage=0;isoform_length=1248

ctcttcacggagccgcggctgcgggcgcaaatagtcagtgggcgcttggGGTGTCGTTGCGGTACCAGGTCCGCGTGAGGGGTTCGGGG
GTTCTGGGCAGGCACAATGGCGTCTCGAGCAGGCCCGCGAGCGGCCGGCACCGACGGCAGCGACTTTCAGCACCGGG
AGCGCGTCGCCATGCACTACCAGATGAGGCCCCGGCTGAAAGGGCCCTCACAACTTAAGCAGGTGTTTTCATGCCAGCC
CAGAGTAGAAACATCGCCTTTAGGGATATGAGGAGGATACGTTTGGGACGTGAGAGAATTAGAAGAATAAGATAATG
TGACTCTCAAGTATGAAATCAAGAAGCTGATCTACGTACATCTGGTCATATGGCTGCTGCTGGTTGCTAAGATGAGCGT
GGGACACCTGAGGCTCTTGTCACATGATCAGGTGGCCATGCCCTATCAGTGGGAATACCCGTATTTGCTGAGCATTTTG
CCCTCTCTCTTGGGCCTTCTCTCCTTTCCCCGCAACAACATTAGCTACCTGGTGCTCTCCATGATCAGCATGGGACTCTTTT
CCATCGCTCCACTCATTTATGGCAGCATGGAGATGTTCCCTGCTGCACAGCAGCTCTACCGCCATGGCAAGGCCTACCGT
TTCCTCTTTGGTTTTTCTGCCGTTTCCATCATGTACCTGGTGTTGGTGTTGGCAGTGCAAGTGCATGCCTGGCAGTTGTAC
TACAGCAAGAAGCTCCTAGACTCTTGGTTCACCAGCACACAGGAGAAGAAGCATAAATGAAGCCTCTTTGGGGTGAAG
CCTGGACATCCCATCGAATGAAAGGACACTAGTACAGCGGTTCCAAAATCCCTTCTGGTGATTTTAGCAGCTGTGATGTT
GGTACCTGGTGCAGACCAGGCCAAAGTTCTGGAAAGCTCCTTTTGCCATCTGCTGAGGTGGCAAAACTATAATTTATTC
CTGGTTGGCTAGAACTGGGTGACCAACAGCTATGAAACAAATTTCAGCTGTTTGAAGTTGAACTTTGAGGTTTTTCTTTA
AGAATGAGCTTCGTCCTTGCCTCTACTCGGTCATTCTCCCCATTTCCATCCATTACCCCTTAGCCATTGAGACTAAAGGAA
ATAGGGAATAAATCAAATTACTTCATCTCTAGGTCACGGGTCAGGAAACATTTGGGCAGCTGCTCCCTTGGCAGGCTGT
GGTCTCCTCTgcaagcattttaattaaaaactcaataaagatgcctgc

>VIRT87749

MHLHCQHQHQVHDGNGRKTKEETVGLAMAVELLCSREHLHAAINEWSDGKESHADHGEHQ
VANVVAGKGEKAQERGQNAQQIRVFPLIGHGHLIM

>i1_HQ_samplec9816d|c64100/f41p0/1184
isoform=c64100;full_length_coverage=41;non_full_length_coverage=0;isoform_length=1184

ggggccggaagtTCTCTTTCAACGGAGCCGCGCGGCTGCGGGGGGCGCAAATAGGGTCACTGGGCCGCTTGGCGGTGTCG
TTGCGGTACCAGGTCCGCGTGAGGGGTTCGGGGGTTCTGGGCAGGCACAATGGCGTCTCGAGCAGGCCCGCGAGCGG
CCGGCACCGACGGCAGCGACTTTCAGCACCGGGAGCGCGTCGCCATGCACTACCAGATGAGTGTGACCCTCAAGTATG
AAATCAAGAAGCTGATCTACGTACATCTGGTCATATGGCTGCTGCTGGTTGCTAAGATGAGCGTGGGACACCTGAGGC
TCTTGTCACATGATCAGGTGGCCATGCCCTATCAGTGGGAATACCCGTATTTGCTGAGCATTTTGCCCTCTCTCTTGGGC
CTTCTCTCCTTTCCCCGCAACAACATTAGCTACCTGGTGCTCTCCATGATCAGCATGGGACTCTTTTCCATCGCTCCACTCA
TTTATGGCAGCATGGAGATGTTCCCTGCTGCACAGCAGCTCTACCGCCATGGCAAGGCCTACCGTTTCCTCTTTGGTTTT
TCTGCCGTTTCCATCATGTACCTGGTGTTGGTGTTGGCAGTGCAAGTGCATGCCTGGCAGTTGTACTACAGCAAGAAGC
TCCTAGACTCTTGGTTCACCAGCACACAGGAGAAGAAGCATAAATGAAGCCTCTTTGGGGTGAAGCCTGGACATCCCAT
CGAATGAAAGGACACTAGTACAGCGGTTCCAAAATCCCTTCTGGTGATTTTAGCAGCTGTGATGTTGGTACCTGGTGCA
GACCAGGCCAAAGTTCTGGAAAGCTCCTTTTGCCATCTGCTGAGGTGGCAAAACTATAATTTATTCCTGGTTGGCTAGA
ACTGGGTGACCAACAGCTATGAAACAAATTTCAGCTGTTTGAAGTTGAACTTTGAGGTTTTTCTTTAAGAATGAGCTTCG
TCCTTGCCTCTACTCGGTCATTCTCCCCATTTCCATCCATTACCCCTTAGCCATTGAGACTAAAGGAAATAGGGAATAAAT
CAAATTACTTCATCTCTAGGTCACGGGTCAGGAAACATTTGGGCAGCTGCTCCCTTGGCAGCTGTGGTCTCCTCTGCAAA
GCATTTTAATTAAAAACCTCAATAAAGATGGCCCTGCCCACActgtgccttgtgtttggggcttgatagagt

>VIRT88771

MHLHCQHQHQVHDGNGRKTKEETVGLAMAVELLCSREHLHAAINEWSDGKESHADHGEHQ
VANVVAGKGEKAQERGQNAQQIRVFPLIGHGHLIM

```
>NM_032492.4 Homo sapiens jagunal homolog 1 (JAGN1), transcript variant 1, mRNA
CTCTTCACGGAGCCGCGCGGCTGCGGGGGCGCAAATAGGGTCAGTGGGCCGCTTGGCGGTGTCGTTGCGG
TACCAGGTCCGCGTGAGGGGTTCGGGGGTTCTGGGCAGGCACAATGGCGTCTCGAGCAGGCCCGCGAGCG
GCCGGCACCGACGGCAGCGACTTTCAGCACCGGGAGCGCGTCGCCATGCACTACCAGATGAGTGTGACTC
TCAAGTATGAAATCAAGAAGCTGATCTACGTACATCTGGTCATATGGCTGCTGCTGGTTGCTAAGATGAG
CGTGGGACACCTGAGGCTCTTGTCACATGATCAGGTGGCCATGCCCTATCAGTGGGAATACCCGTATTTG
CTGAGCATTTTGCCCTCTCTCTTGGGCCTTCTCTCCTTTCCCCGCAACAACATTAGCTACCTGGTGCTCT
CCATGATCAGCATGGGACTCTTTTCCATCGCTCCACTCATTTATGGCAGCATGGAGATGTTCCCTGCTGC
ACAGCAGCTCTACCGCCATGGCAAGGCCTACCGTTTCCTCTTTGGTTTTTCTGCCGTTTCCATCATGTAC
CTGGTGTTGGTGTTGGCAGTGCAAGTGCATGCCTGGCAGTTGTACTACAGCAAGAAGCTCCTAGACTCTT
GGTTCACCAGCACACAGGAGAAGAAGCATAAATGAAGCCTCTTTGGGGTGAAGCCTGGACATCCCATCGA
ATGAAAGGACACTAGTACAGCGGTTCCAAAATCCCTTCTGGTGATTTTAGCAGCTGTGATGTTGGTACCT
GGTGCAGACCAGGCCAAAGTTCTGGAAAGCTCCTTTTGCCATCTGCTGAGGTGGCAAAACTATAATTTAT
TCCTGGTTGGCTAGAACTGGGTGACCAACAGCTATGAAACAAATTTCAGCTGTTTGAAGTTGAACTTTGA
GGTTTTTCTTTAAGAATGAGCTTCGTCCTTGCCTCTACTCGGTCATTCTCCCCATTTCCATCCATTACCC
CTTAGCCATTGAGACTAAAGGAAATAGGGAATAAATCAAATTACTTCATCTCTAGGTCACGGGTCAGGAA
ACATTTGGGCAGCTGCTCCCTTGGCAGGCTGTGGTCTCCTCTGCAAAGCATTTTAATTAAAAACCTCAAT
AAAGATGGCCCTGCCCACACTGTGCCTTGTGTTTGGGGCTTGATAGAGTGTGAAAGGTGTGTTGCTTGAA
AGGTGTGGCCTTAGACACTAGGAGATGATGGGTAGAAGCTCCTTGAGAAACCTAGCAGTGGCCATAGAGA
AGTAGACTGGGTATGGGGAACCTAAGTCATAGTTGTAACCTACAGCTGATTGGTAAGAACTATGGGCCAG
ACATTTAATCTCTCCATCTATGAAATAGTCCATTCTCATTTATCTACCTTAGGTTATGGGCTCTTCTTTT
AGTTGGATATTCACATTTATCTTTCAGTGTTAACTGGGTATCAGTTATGAGTCTACTACTGTATAATGGC
ATCCAAAAGAATTTTAAAGTACCTCGGTTTATAGGGATTTACAATTCAGTAGAAACTTGTTATTCATGTG
AAACAGTGAACATAATATCTAATGATGTGGAGCAAGTACATTAAAAACAGGAAGGAAGAGATCTGTGATT
AAACTAAAGGCCCTTTCACACCTAAAA
```

```
>NP_115881.3 protein jagunal homolog 1 isoform a [Homo sapiens]
MASRAGPRAAGTDGSDFQHRERVAMHYQMSVTLKYEIKKLIYVHLVIWLLLVAKMSVGHLRLLSHDQVAM
PYQWEYPYLLSILPSLLGLLSFPRNNISYLVLSMISMGLFSIAPLIYGSMEMFPAAQQLYRHGKAYRFLF
GFSAVSIMYLVLVLAVQVHAWQLYYSKKLLDSWFTSTQEKKHK
```

```
i1_HQ_samplec9816d|c7012/f2p0/1248      ---------------ctcttcacggagccgc-----gg----ctgcgggcgcaaatagtc   36
i1_HQ_samplec9816d|c64100/f41p0/1184    ggggccggaagtTCTCTTTCAACGGAGCCGCGCGGCTGCGGGGGGCGCAAATAGGGTCAC   60
NM_032492.4                             ---------------CTCTTCACGGAGCCGCGCG-GCTGCGGGGGCGCAAATAGGGTCAG   44
                                                       ** *  **********             ***     *

i1_HQ_samplec9816d|c7012/f2p0/1248      agtgggcgcttggGGTGTCGTTGCGGTACCAGGTCCGCGTGAGGGGTTCGGGGGTTCTGG   96
i1_HQ_samplec9816d|c64100/f41p0/1184    TGGGCCGCTTGGCGGTGTCGTTGCGGTACCAGGTCCGCGTGAGGGGTTCGGGGGTTCTGG   12
NM_032492.4                             TGGGCCGCTTGGCGGTGTCGTTGCGGTACCAGGTCCGCGTGAGGGGTTCGGGGGTTCTGG   10
                                         * *     * * ***********************************************

i1_HQ_samplec9816d|c7012/f2p0/1248      GCAGGCACAATGGCGTCTCGAGCAGGCCCGCGAGCGGCCGGCACCGACGGCAGCGACTTT   15
i1_HQ_samplec9816d|c64100/f41p0/1184    GCAGGCACAATGGCGTCTCGAGCAGGCCCGCGAGCGGCCGGCACCGACGGCAGCGACTTT   18
```

```
NM_032492.4                                 GCAGGCACAATGGCGTCTCGAGCAGGCCCGCGAGCGGCCGGCACCGACGGCAGCGACTTT       16
                                            ************************************************************

i1_HQ_samplec9816d|c7012/f2p0/1248          CAGCACCGGGAGCGCGTCGCCATGCACTACCAGATGAGGCCCCGGCTGAAAGGGCCCTCA       21
i1_HQ_samplec9816d|c64100/f41p0/1184        CAGCACCGGGAGCGCGTCGC----------------------------------------       20
NM_032492.4                                 CAGCACCGGGAGCGCGTCGC----------------------------------------       18
                                            ********************

i1_HQ_samplec9816d|c7012/f2p0/1248          CAACTTAAGCAGGTGTTTTCATGCCAGCCCAGAGTAGAAACATCGCCTTTAGGGATATGA       27
i1_HQ_samplec9816d|c64100/f41p0/1184        ------------------------------------------------------------       20
NM_032492.4                                 ------------------------------------------------------------       18

i1_HQ_samplec9816d|c7012/f2p0/1248          GGAGGATACGTTTGGGACGTGAGAGAATTAGAAGAATAAGATAATGTGACTCTCAAGTAT       33
i1_HQ_samplec9816d|c64100/f41p0/1184        --------------------------CATGCACTACCAGATGAGTGTGACCCTCAAGTAT       23
NM_032492.4                                 --------------------------CATGCACTACCAGATGAGTGTGACTCTCAAGTAT       21
                                                                        *  *  *  *    * ****** *********

i1_HQ_samplec9816d|c7012/f2p0/1248          GAAATCAAGAAGCTGATCTACGTACATCTGGTCATATGGCTGCTGCTGGTTGCTAAGATG       39
i1_HQ_samplec9816d|c64100/f41p0/1184        GAAATCAAGAAGCTGATCTACGTACATCTGGTCATATGGCTGCTGCTGGTTGCTAAGATG       29
NM_032492.4                                 GAAATCAAGAAGCTGATCTACGTACATCTGGTCATATGGCTGCTGCTGGTTGCTAAGATG       27
                                            ************************************************************

i1_HQ_samplec9816d|c7012/f2p0/1248          AGCGTGGGACACCTGAGGCTCTTGTCACATGATCAGGTGGCCATGCCCTATCAGTGGGAA       45
i1_HQ_samplec9816d|c64100/f41p0/1184        AGCGTGGGACACCTGAGGCTCTTGTCACATGATCAGGTGGCCATGCCCTATCAGTGGGAA       35
NM_032492.4                                 AGCGTGGGACACCTGAGGCTCTTGTCACATGATCAGGTGGCCATGCCCTATCAGTGGGAA       33
                                            ************************************************************

i1_HQ_samplec9816d|c7012/f2p0/1248          TACCCGTATTTGCTGAGCATTTTGCCCTCTCTCTTGGGCCTTCTCTCCTTTCCCCGCAAC       51
i1_HQ_samplec9816d|c64100/f41p0/1184        TACCCGTATTTGCTGAGCATTTTGCCCTCTCTCTTGGGCCTTCTCTCCTTTCCCCGCAAC       41
NM_032492.4                                 TACCCGTATTTGCTGAGCATTTTGCCCTCTCTCTTGGGCCTTCTCTCCTTTCCCCGCAAC       39
                                            ************************************************************

i1_HQ_samplec9816d|c7012/f2p0/1248          AACATTAGCTACCTGGTGCTCTCCATGATCAGCATGGGACTCTTTTCCATCGCTCCACTC       57
i1_HQ_samplec9816d|c64100/f41p0/1184        AACATTAGCTACCTGGTGCTCTCCATGATCAGCATGGGACTCTTTTCCATCGCTCCACTC       47
NM_032492.4                                 AACATTAGCTACCTGGTGCTCTCCATGATCAGCATGGGACTCTTTTCCATCGCTCCACTC       45
                                            ************************************************************

i1_HQ_samplec9816d|c7012/f2p0/1248          ATTTATGGCAGCATGGAGATGTTCCCTGCTGCACAGCAGCTCTACCGCCATGGCAAGGCC       63
i1_HQ_samplec9816d|c64100/f41p0/1184        ATTTATGGCAGCATGGAGATGTTCCCTGCTGCACAGCAGCTCTACCGCCATGGCAAGGCC       53
NM_032492.4                                 ATTTATGGCAGCATGGAGATGTTCCCTGCTGCACAGCAGCTCTACCGCCATGGCAAGGCC       51
                                            ************************************************************

i1_HQ_samplec9816d|c7012/f2p0/1248          TACCGTTTCCTCTTTGGTTTTTCTGCCGTTTCCATCATGTACCTGGTGTTGGTGTTGGCA       69
i1_HQ_samplec9816d|c64100/f41p0/1184        TACCGTTTCCTCTTTGGTTTTTCTGCCGTTTCCATCATGTACCTGGTGTTGGTGTTGGCA       59
NM_032492.4                                 TACCGTTTCCTCTTTGGTTTTTCTGCCGTTTCCATCATGTACCTGGTGTTGGTGTTGGCA       57
                                            ************************************************************

i1_HQ_samplec9816d|c7012/f2p0/1248          GTGCAAGTGCATGCCTGGCAGTTGTACTACAGCAAGAAGCTCCTAGACTCTTGGTTCACC       75
i1_HQ_samplec9816d|c64100/f41p0/1184        GTGCAAGTGCATGCCTGGCAGTTGTACTACAGCAAGAAGCTCCTAGACTCTTGGTTCACC       65
NM_032492.4                                 GTGCAAGTGCATGCCTGGCAGTTGTACTACAGCAAGAAGCTCCTAGACTCTTGGTTCACC       63
                                            ************************************************************

i1_HQ_samplec9816d|c7012/f2p0/1248          AGCACACAGGAGAAGAAGCATAAATGAAGCCTCTTTGGGGTGAAGCCTGGACATCCCATC       81
i1_HQ_samplec9816d|c64100/f41p0/1184        AGCACACAGGAGAAGAAGCATAAATGAAGCCTCTTTGGGGTGAAGCCTGGACATCCCATC       71
NM_032492.4                                 AGCACACAGGAGAAGAAGCATAAATGAAGCCTCTTTGGGGTGAAGCCTGGACATCCCATC       69
                                            ************************************************************

i1_HQ_samplec9816d|c7012/f2p0/1248          GAATGAAAGGACACTAGTACAGCGGTTCCAAAATCCCTTCTGGTGATTTTAGCAGCTGTG       87
i1_HQ_samplec9816d|c64100/f41p0/1184        GAATGAAAGGACACTAGTACAGCGGTTCCAAAATCCCTTCTGGTGATTTTAGCAGCTGTG       77
NM_032492.4                                 GAATGAAAGGACACTAGTACAGCGGTTCCAAAATCCCTTCTGGTGATTTTAGCAGCTGTG       75
                                            ************************************************************

i1_HQ_samplec9816d|c7012/f2p0/1248          ATGTTGGTACCTGGTGCAGACCAGGCCAAAGTTCTGGAAAGCTCCTTTTGCCATCTGCTG       93
i1_HQ_samplec9816d|c64100/f41p0/1184        ATGTTGGTACCTGGTGCAGACCAGGCCAAAGTTCTGGAAAGCTCCTTTTGCCATCTGCTG       83
NM_032492.4                                 ATGTTGGTACCTGGTGCAGACCAGGCCAAAGTTCTGGAAAGCTCCTTTTGCCATCTGCTG       81
                                            ************************************************************

i1_HQ_samplec9816d|c7012/f2p0/1248          AGGTGGCAAAACTATAATTTATTCCTGGTTGGCTAGAACTGGGTGACCAACAGCTATGAA       99
i1_HQ_samplec9816d|c64100/f41p0/1184        AGGTGGCAAAACTATAATTTATTCCTGGTTGGCTAGAACTGGGTGACCAACAGCTATGAA       89
NM_032492.4                                 AGGTGGCAAAACTATAATTTATTCCTGGTTGGCTAGAACTGGGTGACCAACAGCTATGAA       87
                                            ************************************************************

i1_HQ_samplec9816d|c7012/f2p0/1248          ACAAATTTCAGCTGTTTGAAGTTGAACTTTGAGGTTTTTCTTTAAGAATGAGCTTCGTCC       10
i1_HQ_samplec9816d|c64100/f41p0/1184        ACAAATTTCAGCTGTTTGAAGTTGAACTTTGAGGTTTTTCTTTAAGAATGAGCTTCGTCC       95
NM_032492.4                                 ACAAATTTCAGCTGTTTGAAGTTGAACTTTGAGGTTTTTCTTTAAGAATGAGCTTCGTCC       93
                                            ************************************************************

i1_HQ_samplec9816d|c7012/f2p0/1248          TTGCCTCTACTCGGTCATTCTCCCCATTTCCATCCATTACCCCTTAGCCATTGAGACTAA       11
i1_HQ_samplec9816d|c64100/f41p0/1184        TTGCCTCTACTCGGTCATTCTCCCCATTTCCATCCATTACCCCTTAGCCATTGAGACTAA       10
NM_032492.4                                 TTGCCTCTACTCGGTCATTCTCCCCATTTCCATCCATTACCCCTTAGCCATTGAGACTAA       99
                                            ************************************************************
```

```
i1_HQ_samplec9816d|c7012/f2p0/1248          AGGAAATAGGGAATAAATCAAATTACTTCATCTCTAGGTCACGGGTCAGGAAACATTTGG     11
i1_HQ_samplec9816d|c64100/f41p0/1184        AGGAAATAGGGAATAAATCAAATTACTTCATCTCTAGGTCACGGGTCAGGAAACATTTGG     10
NM_032492.4                                 AGGAAATAGGGAATAAATCAAATTACTTCATCTCTAGGTCACGGGTCAGGAAACATTTGG     10
                                            ************************************************************

i1_HQ_samplec9816d|c7012/f2p0/1248          GCAGCTGCTCCCTTGGCAGGCTGTGGTCTCCTCTgcaagcattttaattaaaaactcaat     12
i1_HQ_samplec9816d|c64100/f41p0/1184        GCAGCTGCTCCCTTGGCA-GCTGTGGTCTCCTCTGCAAAGCATTTTAATTAAAAACCTCA     11
NM_032492.4                                 GCAGCTGCTCCCTTGGCAGGCTGTGGTCTCCTCTGCAAAGCATTTTAATTAAAAACCTCA     11
                                            ****************** *******************    *** * * ****  *

i1_HQ_samplec9816d|c7012/f2p0/1248          aaa--gatgcctgc----------------------------------------------     12
i1_HQ_samplec9816d|c64100/f41p0/1184        ATAAAGATGGCCCTGCCCACActgtgccttgtgtttggggcttgatagagt---------     11
NM_032492.4                                 ATAAAGATGGCCCTGCCCACACTGTGCCTTGTGTTTGGGGCTTGATAGAGTGTGAAAGGT     11
                                            * *  **** *

i1_HQ_samplec9816d|c7012/f2p0/1248          ------------------------------------------------------------     12
i1_HQ_samplec9816d|c64100/f41p0/1184        ------------------------------------------------------------     11
NM_032492.4                                 GTGTTGCTTGAAAGGTGTGGCCTTAGACACTAGGAGATGATGGGTAGAAGCTCCTTGAGA     12

i1_HQ_samplec9816d|c7012/f2p0/1248          ------------------------------------------------------------     12
i1_HQ_samplec9816d|c64100/f41p0/1184        ------------------------------------------------------------     11
NM_032492.4                                 AACCTAGCAGTGGCCATAGAGAAGTAGACTGGGTATGGGGAACCTAAGTCATAGTTGTAA     12

i1_HQ_samplec9816d|c7012/f2p0/1248          ------------------------------------------------------------     12
i1_HQ_samplec9816d|c64100/f41p0/1184        ------------------------------------------------------------     11
NM_032492.4                                 CCTACAGCTGATTGGTAAGAACTATGGGCCAGACATTTAATCTCTCCATCTATGAAATAG     13

i1_HQ_samplec9816d|c7012/f2p0/1248          ------------------------------------------------------------     12
i1_HQ_samplec9816d|c64100/f41p0/1184        ------------------------------------------------------------     11
NM_032492.4                                 TCCATTCTCATTTATCTACCTTAGGTTATGGGCTCTTCTTTTAGTTGGATATTCACATTT     14

i1_HQ_samplec9816d|c7012/f2p0/1248          ------------------------------------------------------------     12
i1_HQ_samplec9816d|c64100/f41p0/1184        ------------------------------------------------------------     11
NM_032492.4                                 ATCTTTCAGTGTTAACTGGGTATCAGTTATGAGTCTACTACTGTATAATGGCATCCAAAA     14

i1_HQ_samplec9816d|c7012/f2p0/1248          ------------------------------------------------------------     12
i1_HQ_samplec9816d|c64100/f41p0/1184        ------------------------------------------------------------     11
NM_032492.4                                 GAATTTTAAAGTACCTCGGTTTATAGGGATTTACAATTCAGTAGAAACTTGTTATTCATG     15

i1_HQ_samplec9816d|c7012/f2p0/1248          ------------------------------------------------------------     12
i1_HQ_samplec9816d|c64100/f41p0/1184        ------------------------------------------------------------     11
NM_032492.4                                 TGAAACAGTGAACATAATATCTAATGATGTGGAGCAAGTACATTAAAAACAGGAAGGAAG     15

i1_HQ_samplec9816d|c7012/f2p0/1248          --------------------------------------- 1248
i1_HQ_samplec9816d|c64100/f41p0/1184        --------------------------------------- 1184
NM_032492.4                                 AGATCTGTGATTAAACTAAAGGCCCTTTCACACCTAAAA 1637
```

```
VIRT87749        ------------------------------------------------------------     0
VIRT88771        ------------------------------------------------------------     0
NP_115881.3      MASRAGPRAAGTDGSDFQHRERVAMHYQMSVTLKYEIKKLIYVHLVIWLLLVAKMSVGHL     60

VIRT87749        -----------------------------------------------------------M     1
VIRT88771        -----------------------------------------------------------M     1
NP_115881.3      RLLSHDQVAMPYQWEYPYLLSILPSLLGLLSFPRNNISYLVLSMISMGLFSIAPLIYGSM     120
                                                                            *

VIRT87749        HLHCQHQHQVHDGNGRKTKE--ETVGLAMAVELLCSREH------LHAAINEWSDGKESH     53
VIRT88771        HLHCQHQHQVHDGNGRKTKE--ETVGLAMAVELLCSREH------LHAAINEWSDGKESH     53
NP_115881.3      EMFPAAQQLYRHGKAYRFLFGFSAVSIMYLVLVLAVQVHAWQLYYSKKLLDSWFTSTQEK     180
                 .:.   *:  :.*:. :     .:*.:   * :*. : *       :  ::.*  ..:.:

VIRT87749        ADHGEHQVANVVAGKGEKAQERGQNAQQIRVFPLIGHGHLIM     95
VIRT88771        ADHGEHQVANVVAGKGEKAQERGQNAQQIRVFPLIGHGHLIM     95
NP_115881.3      KHK---------------------------------------     183
                  .:
```

**EMC3**

| Sequence | Header | AA Seq |
|---|---|---|
| **Present** | i1_HQ_samplec9816d\|c26333/f2p57/1162 | |
| | | |
| **Absent** | i1_HQ_samplec9816d\|c26920/f2p0/1048 | |
| **NCBI** | NM_018447 | |
| | NR_103821 | |

>i1_HQ_samplec9816d|c26333/f2p57/1162
isoform=c26333;full_length_coverage=2;non_full_length_coverage=57;isoform_length=1162

agctgaaggcagtaagctcggctcacagtcgcaggagagttctggggtacACGGGCAAAGGGGCTTGAGAAGGCCCGGAGGCGAAGCC
GAAGAGAAGCAACTGTGCCCCGGAGAAGAGAAGCTCGCCCATTCCAGACTGGGAACCAGCTTTCAGTGAAGATGGCA
GGGCCAGAACTGTTGCTCGACTCCAACATCCGCCTCTGGGTGGTCCTACCCATCGTTATCATCACTTTCTTCGTAGGCAT
GATCCGCCACTACGTGTCCATCCTGCTGCAGAGCGACAAGAAGCTCACCCAGGAACAAGTATCTGACAGAAAATATCTT
TCAGGCCTGGTGCAATGGCTCACACCTATAATCCCAGCACTTTGGGGGGCTGAGATGGGAGGATCCCTTGAGCCCAGG
AATTTGAGACCAACCTTGGCAGCATGTCAAGTCCTAATTCGAAGCAGAGTCCTCAGGGAAAATGGAAAATACATTCCCA
AACAGTCTTTCTTGACACGAAAATATTATTTCAACAACCCAGAGGATGGATTTTTCAAAAAAACTAAACGGAAGGTAGT
GCCACCTTCTCCTATGACTGATCCTACTATGTTGACAGACATGATGAAAGGGAATGTAACAAATGTCCTCCCTATGATTC
TTATTGGTGGATGGATCAACATGACATTCTCAGGCTTTGTCACAACCAAGGTCCCATTTCCACTGACCCTCCGTTTTAAG
CCTATGTTACAGCAAGGAATCGAGCTACTCACATTAGATGCATCCTGGGTGAGTTCTGCATCCTGGTACTTCCTCAATGT
ATTTGGGCTTCGGAGCATTTACTCTCTGATTCTGGGCCAAGATAATGCCGCTGACCAATCACGAATGATGCAGGAGCAG
ATGACGGGAGCAGCCATGGCCATGCCCGCAGACACAAACAAAGCTTTCAAGACAGAGTGGGAAGCTTTGGAGCTGAC
GGATCACCAGTGGGCACTAGATGATGTCGAAGAAGAGCTCATGGCCAAAGACCTCCACTTCGAAGGCATGTTCAAAAA
GGAATTACAGACCTCTATTTTTTGAAGACCGAGCAGGGATTAGCTGTGTCAGGAACTTGGAGTTGCACTTAACCTTGTA
ACTTTGTTTGGAgctggcacctcttgaaataaaaaggaggatgcacgag

>VIRT151947

MAGPELLLDSNIRLWVVLPIVIITFFVGMIRHYVSILLQSDKKLTQEQVSDRKYLSGLVQ
WLTPIIPALWGAEMGGSLEPRNLRPTLAACQVLIRSRVLRENGKYIPKQSFLTRKYYFNN
PEDGFFKKTKRKVVPPSPMTDPTMLTDMMKGNVTNVLPMILIGGWINMTFSGFVTTKVPF
PLTLRFKPMLQQGIELLTLDASWVSSASWYFLNVFGLRSIYSLILGQDNAADQSRMMQEQ
MTGAAMAMPADTNKAFKTEWEALELTDHQWALDDVEEELMAKDLHFEGMFKKELQTSIF

>i1_HQ_samplec9816d|c26920/f2p0/1048
isoform=c26920;full_length_coverage=2;non_full_length_coverage=0;isoform_length=1048

agctgaaggcagtaagctcggctcacagtcgcaggagagttctggggtacacgggcaaagggccttgaGAAGCCCGGAGGCGAAGCCGAAGA
GAAGCAACTGTGCCCCGGAGAAGAGAAGCTCGCCCATTCCAGACTGGGAACCAGCTTTCAGTGAAGATGGCAGGGCC
AGAACTGTTGCTCGACTCCAACATCCGCCTCTGGGTGGTCCTACCCATCGTTATCATCACTTTCTTCGTAGGCATGATCCG
CCACTACGTGTCCATCCTGCTGCAGAGCGACAAGAAGCTCACCCAGGAACAAGTATCTGACAGTCAAGTCCTAATTCGA
AGCAGAGTCCTCAGGGAAAATGGAAAATACATTCCCAAACAGTCTTTCTTGACACGAAAATATTATTTCAACAACCCAG
AGGATGGATTTTTCAAAAAAACTAAACGGAAGGTAGTGCCACCTTCTCCTATGACTGATCCTACTATGTTGACAGACAT
GATGAAAGGGAATGTAACAAATGTCCTCCCTATGATTCTTATTGGTGGATGGATCAACATGACATTCTCAGGCTTTGTCA
CAACCAAGGTCCCATTTCCACTGACCCTCCGTTTTAAGCCTATGTTACAGCAAGGAATCGAGCTACTCACATTAGATGCA
TCCTGGGTGAGTTCTGCATCCTGGTACTTCCTCAATGTATTTGGGCTTCGGAGCATTTACTCTCTGATTCTGGGCCAAGA
TAATGCCGCTGACCAATCACGAATGATGCAGGAGCAGATGACGGGAGCAGCCATGGCCATGCCCGCAGACACAAACA

AAGCTTTCAAGACAGAGTGGGAAGCTTTGGAGCTGACGGATCACCAGTGGGCACTAGATGATGTCAAGAAGAGCTCAT
GGCCAAAGACCTCCACTTCGAAGGCATGTTCAAAAAGGAATTACAGACCTCTATTTTTTGAAGACCGAGCAGGGATTAG
CTGTGTCAGGAACTTGGAGTTGCACTTAACCTTGTAACTTTGTTTGGAGCTggcacctcttgaaataaaaaggaggatgcacgagcg

```
>VIRT153469
```

```
MAGPELLLDSNIRLWVVLPIVIITFFVGMIRHYVSILLQSDKKLTQEQVSDSQVLIRSRV
LRENGKYIPKQSFLTRKYYFNNPEDGFFKKTKRKVVPPSPMTDPTMLTDMMKGNVTNVLP
MILIGGWINMTFSGFVTTKVPFPLTLRFKPMLQQGIELLTLDASWVSSASWYFLNVFGLR
SIYSLILGQDNAADQSRMMQEQMTGAAMAMPADTNKAFKTEWEALELTDHQWALDDVKKS
SWPKTSTSKACSKRNYRPLFFEDRAGISCVRNLELHLTL
```

```
>NM_018447.3 Homo sapiens ER membrane protein complex subunit 3 (EMC3), transcript variant 1, mRNA
GGGGGTGGGGCCAGGCGGCGGCGAGCGGCGGCGGGCGGGGCTTTTAACGGCCATCGGCCCCTCCCAGCCT
GGACAAAGACACAGAGCGCGGCCTGGGAGGCGGAGCTCTGCGGGCGGCCGGGGCCGCCTCCTTCCAGGCG
CTGCTGCCCCAACACAGGCGGGCACTCTCTGCGGGTCCCGCGGCTCCCGCGGCTTCCGGCCCACTCAGCG
CCTCCAGAGGCCTCAGGCCCGACGCCGCTCTGACGCCACTGAGGTGCGCCACGACGTGACGTCAACGTTC
GCGCCGGAAGTGGAAGACCAGGCAGCCCAGCTGAAGGCAGTAAGCTCGGCTCACAGTCGCAGGAGAGTTC
TGGGGTACACGGGCAAAGGGGCTTGAGAAGGCCCGGAGGCGAAGCCGAAGAGAAGCAACTGTGCCCCGGA
GAAGAGAAGCTCGCCCATTCCAGACTGGGAACCAGCTTTCAGTGAAGATGGCAGGGCCAGAACTGTTGCT
CGACTCCAACATCCGCCTCTGGGTGGTCCTACCCATCGTTATCATCACTTTCTTCGTAGGCATGATCCGC
CACTACGTGTCCATCCTGCTGCAGAGCGACAAGAAGCTCACCCAGGAACAAGTATCTGACAGTCAAGTCC
TAATTCGAAGCAGAGTCCTCAGGGAAAATGGAAAATACATTCCCAAACAGTCTTTCTTGACACGAAAATA
TTATTTCAACAACCCAGAGGATGGATTTTTCAAAAAAACTAAACGGAAGGTAGTGCCACCTTCTCCTATG
ACTGATCCTACTATGTTGACAGACATGATGAAAGGGAATGTAACAAATGTCCTCCCTATGATTCTTATTG
GTGGATGGATCAACATGACATTCTCAGGCTTTGTCACAACCAAGGTCCCATTTCCACTGACCCTCCGTTT
TAAGCCTATGTTACAGCAAGGAATCGAGCTACTCACATTAGATGCATCCTGGGTGAGTTCTGCATCCTGG
TACTTCCTCAATGTATTTGGGCTTCGGAGCATTTACTCTCTGATTCTGGGCCAAGATAATGCCGCTGACC
AATCACGAATGATGCAGGAGCAGATGACGGGAGCAGCCATGGCCATGCCCGCAGACACAAACAAAGCTTT
CAAGACAGAGTGGGAAGCTTTGGAGCTGACGGATCACCAGTGGGCACTAGATGATGTCGAAGAAGAGCTC
ATGGCCAAAGACCTCCACTTCGAAGGCATGTTCAAAAAGGAATTACAGACCTCTATTTTTTGAAGACCGA
GCAGGGATTAGCTGTGTCAGGAACTTGGAGTTGCACTTAACCTTGTAACTTTGTTTGGAGCTGGCACCTC
TTGAAATAAAAAGGAGGATGCACGAGCTGGCAGGCATGCAGCAAGGCTTGTTCTTGTCTGGGCTGGGTTC
CCCTTTATGTTTGAAACTAGAGGAAATAGGAGTATGCTGGGCAACTTGTTATTTTTAAAATGTTGTAAAT
GTTCCTTCTGGTGACTCTAGTAATGAAAGTCAGAGAAAGGGGGAAACCTCAGGCTACTGATAATATATAA
AATCTTGGCCAGGCGCAGTGGCTCACGCCTGTAATCCCAGGATTTTGGGAGGCTGAGGCGGGTGGGTCAC
GAGATCAGGAGTTCAAGATCAGCCTGGCCAAGATGGTGAAACCCCATCTCTACTAAAAATACAAAAAAAA
ATTAGCCGGGCGTGGTGGTGGGCACCTGTAATCCCAGCTACTTGGGAGGCTGAGGCAGAGAACTGCTTAA
ACCTAGGAGGCGGAGGATGCAGTGAGCTGACATCGTGCCACTGCGCTCCAGCCTGGGCGACAGAGCAAAA
CTCCATCTGAAAAAAAAAAAAAAATATATATATATATATATATATATCTATCTTAGCAGAAGCCCAGTCT
TCGAAGGAAGCATTGTTAATTATAGCTAATGATTATGTGCAGTCAGCCTGACATCTAGCCACAGTAAACT
CCTGTTTTCTTGGGATCCAAACACCCTGTGTTTTACCATTAGTTTTGTTAGTATTGTTAACTTTCTTTAC
ACAAGTAAACCATTTCCACTACCAAAGGTTTAGAAACTATGGATGAACAAAATGAATCTCCCTGTTCATT
TTGAGCCTACATAGACATAGAGACCAAATATGTCAAAACCTTATTCAAATAAACTCTCTTCAGAGTGAAT
ACCCAGGGTCAGGACAGACTCAGCAAACCTTCTATATCACTTAATTCTGCCCAGTGAAAATCAGTGACCC
TGAGGCACTGCCAGATTCTAAAGTGCCTTTGGGATCATCAGAGACTGTCTATAAATAATGCAAAATCTTG
ATCTGGATGTCAGTGTATCAAATCTTATCAAATCCATTGACAAATCAGTGGTGCTATACTGGAAATGGCT
ACTCCTTTGGATGAAGGACCTCATTTCCCAGTTGCTTGGAACAAAAGAGAATTTACATATTGCTGTAAGA
AGGGAGAGACGGCAGTTGTTGGTAGACACACAGATAAGGGTGCCCAAGAATAATTCTGTCTGGATTAAAG
GTCCCAGGGAAGTGACGGTGCCATTTCTGTACCAAGAGGTGAATATAATTTGCATGTTGAGTGCAGTAAT
TAAGAACAAACGACTCGACTCCACAAATTAAACCGTCCTGTTTTCTCTTCTTAAAAAAAAAAAAAAAAAA
>NP_060917.1 ER membrane protein complex subunit 3 [Homo sapiens]
MAGPELLLDSNIRLWVVLPIVIITFFVGMIRHYVSILLQSDKKLTQEQVSDSQVLIRSRVLRENGKYIPK
QSFLTRKYYFNNPEDGFFKKTKRKVVPPSPMTDPTMLTDMMKGNVTNVLPMILIGGWINMTFSGFVTTKV
PFPLTLRFKPMLQQGIELLTLDASWVSSASWYFLNVFGLRSIYSLILGQDNAADQSRMMQEQMTGAAMAM
PADTNKAFKTEWEALELTDHQWALDDVEEELMAKDLHFEGMFKKELQTSIF
```

```
>NR_103821.1 Homo sapiens EMC3 antisense RNA 1 (EMC3-AS1), long non-coding RNA
GGCGTCGGGCCTGGCGGGAAAGTGGAAAACTATCGGCGGTGGCCCAGGCTCGGTGGCTCACTCCTGGAAT
CCCAGCACTTTGGGAGGCCAAGGTGGGGGGATCACGAGGTCAGGGGATCGAGACCATCCTGGCTAACAGG
TGAAACCCTGTCTCTACTAAAAATACAAAAAAATTAGCCAGGCGTGGTGGAGGCGCCTGTAGTCGCAGCT
ACTCGGGAGGCTGAGGCAGGAGAATGGCGTGAACCCAGGAGGCGGAGCCTGCAGTGAGCCGAGATTGCTG
CACTGCACTCCAGCCTGGGCGACAGAGGAAGACTCCGTCTCAAAAAAAAAAAAAAAAAAAAGAAAGAAAAC
TACAGGCGGGGACGGCTTCTCGGCAATTGAAAACACTGCCTCAGTATCTGAACACAAGTAACAGAGTATG
CAAGAGGAGGAGACCTGCACAACTGGATACACCCAGAAATGTCCCTGGCTCAACAAGCCTTCCAGCATGA
CCCTGTGTCTTCCCCCTCCACGCAGAGCACCAGCAACAGTAGTGCAGACCCAGAAACCTCCCTGGCTCAA
CAAGGCCCCCAGCATGACCCCATGGCCTTCCCCTCCACCCAGAGCACCTGTAGCAGTGGTGGAGTCCCAG
AAATGTCCCTGGCTCAACAAGCCTTCCAGTATCAGCCTGTGGCCTTCTTCTCCACCCAGAGCACCAGCAG
CAGTGGTGGAGACCCAGAAACATCCCAGGCTCAACAAGCCGCCCAGCATGACCCTGTGGCCTACCCCTAT
ACCCAGAGCAACAGCAGCAGTGGTGCAGACCCAGAAACCTCTGGCTCACCACCTCCCCAGCAACACCCTG
TGGCCTTCTCCACCAAGAGCACCAGTAGCAGTGGCAGAGACCCAGAAATGTCCCTGACACAGCAACAACC
ATCCCAGGAAGCCAGTGTCATTCAGGCTGGGCAGCCCAAGGCTTTGACTTCAGCCTTGCCAATAAGGAGG
```

```
CTGTGACTGCCGCAGGGCTGCCAGGGGATGCACTTCCATTGTATTAAAATGTTTTTGCCTGTGTCCACCG
CCGAAAAGGAGGAATAAGATGACCCCAACATAGTTGCACGGCTGAAGACAAAAATCAAGTTGGTGGGGTC
AATATTGTTACCATAGTCACTACACTTAGAGTAGTTTTATAGTCAGTGTAGACAGGTGGCACCATGCAGC
AGGGATCAGCCTCCACCACACCTGACCAGGAGCTCCAGAACTGTAAAATCCTGGACACCATTGGCCGTGG
CACGTTCAGTGAGGTCCAGGATCACATGCTGATTGGGACCCAAATGGCCATCAAAATCATCCCCAAGGCT
GGCTCCCTTGGCATCACTCTCCAGAGAGTGATAAGTATTTTAAAGTTACTCTGTCACTTCAATATTGTAC
GGTTGTATCAAGTGATTGACACCCCCAACACCAGTTATTTATTTAGTAACGGAGTATGCAAGAGGAGGAC
ACCCACGCAACCAATACACCACCATGGCCTCATGAGGGAGGAGAAGGCCTAGACCATGTTCAGGCAGATT
CTGTCGGCCATGCAGTAGTGCCATAGCAAATTGCGCAGAGACCTGAACCCAGAAAACATCATCCTTGATG
AGGACGGTAACGTTAAGATCGCAGACTTCGGCTTTGGTACCACATTCCATGATGGGCAGAAGCTGACAGC
CCTTTGTAGCACTTAACCCCTACATGGCCCTGGAACGTTTCCTAGGCCAGGGCTACCAATGTGCCACCAT
GGATATTCAGAGCCTCAGAGTAATTTTATACCACATGGTGGCTGGGGTTCTGCCCTTCTGCTCATGCAGC
ATTAGGGTCCTCTCAGCAAAAATTTAAAGTGGAAGCTATTTTTCCCCAGTCTACTTTTCCTGAGGTCTTA
AAAGCCTCATTAAAAAACTATTAACAGTAGACCCCAGGGAGCAGACCACACTAGAAGAAGTTATGAGGGA
CCCGTGGGTGAACAGTGGTCAGGAGTTGCCTCTGACAACATGAAGAACAAATCCTGGACCACCTGAATCC
CAAAACAACCCAGCTTTTGGTGGCCATGGGATTCCAGGCTGAGAACCTATCTGTGGCAATCAAAGAAAAA
TTATTCAGTTATCCCATGGCCACCTACCTTGTTTTGGAACAAACAAAACAGAAGAAGCGGTCCACTATCA
GACCACAGACCCTTCCTCCTGGGGATCCCACTTGTCCTCTCTACATTGAAGTTTCCACCTTCCCTCTTTC
ACTGAAGCGGGCTCATAGCATTCAGCAGAAGACTGTGGGTGCCAAGTCTGGGCAGGGCCTTTGCCCTTGG
GAGTCCTGTTTTAGACCCAGCTCCACCACGTGGCCCTGGCCTCTTCTCTGCCCTGTCTGTGCTTGCAGCC
ATGGTGCCCAGTCTGGCCCCACCAGGAGACACCAGAACGACTGTCCATGTGTCTACTCCTTGGCACCTTC
CCCGCATGGTGGGTAGATGGGCAGGGAGGGGTGGGAATCTGTGTAACCGTGGAAGGGAACGGCCCTCAGA
CCCAGAGGGTTTGGAGCCTGGGGTATTGTCACCGACAGTGGCCTTAGCCTAGCTAGCACAGTTAGTGCTG
TGCATCACAGCATAGCTTTTAGAGGCAGGATTTGTAGTCAGTATAGTTGGGAGGCACCATGGAGCAGGGA
TCACCCTCCACCTCACTTGACAAGGAGATACAAAACTATCAGTTCATAGACACCATCTGATAGGGAACTG
GCTCAGCATAGGCCAACTGGGACCCAGGTTGCCATCTTGAAGACTTTCCATCACCCAAATATCATTCAGC
TCTTCCAGGTGGTGAGGGAGTAAACCAGAGGAGGAGAGTTGCACCACCAGATATACCACTATGGCCACAT
CGAGGAGGAAGAGGAGGCCCGGACCATGTTCAGGCAGATTCTGTCAGCCCTGCAGTACTGCCACTTAAAA
AAAAAAAAAAAATCACACATCCAGACCTAAAGCCACAAAACATCCTATTCAAAGAATGAGGCAACATACA
TTGTGGACTTTGGCTTCAGTACAACATTCAGAGAGGGGCAGATGCTGACAGCTTTTTGTGGCATGTACCC
CTACGTGGCCCCAGAACGCTCCCTGGGCCAGGCATGCCAGTGACCCGCCAGGGACATACAAAGCCTCAGT
GTCATACTGTATTTCAGGAATACAGTAGGTAGAAGGGCCAGGACTTTGCCCTTTTACTCAGGGAAGCCTC
CAAACTTCAAGAAAAAATTCTCACAGGAAGATACCATGCCCCACCACTTCTTGCCCTTCAACTTGACTCA
TTAAAAAATTACTAATGCTGAACGCCAGGAAGTGTCCTTCACTGTAACTGATGAAAAATCCATGGGTGAA
AAGTAGCCAGAAGATGCCACTGATACCATACGAAGAGCCACTCCTGGACCACCCCAAACAATCCAGCTCA
TGGTGGCCATGGGATTTCAGGCCAAGAACATCTCTGTGGCAATCATAGAAAGAAAATTCAACTATCCCAT
GGCCACCTACCTCATTTTAGAGCACACAAAACAAGAGAGGAAGTGCTCCACCATCAGAGAACTGTCCCTT
CCTCCCGGGGTTCCCACCTCTCCTTCCCCATCCACTGAACTTTCCACCTTCCCTCTCTCACTGATGCGGG
CTCATAGGGAGCCAGCTTTTAACGTTCAGCCTCCCGAAGAAAGCCAGATGTCAGGGCAGAAGACCGCCAT
TCTTGCCAGTACACCTGCCAGCCTACAGAGGAAGCCAGATCGGGGAAGCCAGGAAGTTTTAGTGAAGCCC
CCAGCATGACCCTATTGTCTCCCCTTTCACCCAGAGCACCAGCAACTGGTGAAGTCTTAGAAACGTCCCT
GATACACCAAAAACCATCCCAGGAAGCCAGCATCGTCCAAGCTGAGAAGTCCAAGCTGAGAAGCCTGAGG
CTGTGACGTCAGCCTGCTCGGCAAGAAGCTGGGGCTGCCGCAGGGTTGCCAGGAGATAGATGCCTTTGCA
TACTATTAAAATATTTTTGCCTGTCTCTACCAAAGAAAAGGAGGAATAAGATCGCCCCAACATAGTTGCA
TGGCTGAAGACAAAAATCAAGTCGTTGTGGTGGTCAAGCCACAATTCCTGCATTTTACATTTCTTCTGTC
TTTATTGTATTGCTTCAATTGGCAAATCATGCTTGTATTCATTCATGGGGTACAATGTGGAATGAGGAAA
TCCCACTACTTAGCATCTCCACTACCTCAGAGAGACCAATTCCACGTGAGGTCCCAGAAGTGTTGATCTA
AACAAGTTGACCCCATAGAAGTAGCAAGTAGATCGATGGTGACCAGGGGTCAGAGAGTGGCAGAGGGAGG
GAATGGGAGGGGGGTTGTCAGTTAAAGGACCAAAGTCTCAGGGAGGAGGAAGAGGTTTTGACATGTAGTG
CACAGCAGAGTGACCAGAGTCAATGAGAATGTGTTGCATTTTGCAAAACACCTGAGAGAGTCCATGTCAA
ATGTCTCTCTGCATTTAGATTGGAGAGGACGAAGGCCCTGAGGTCCAAGAACATTGAAACCTGACAGTGG
ATGCCAATGGCTGTGGGGAGGAGCTGGGCGAGGCGCCCAGGTTGTCGTCTGCCCGGCAACGTGGGAGCCT
GCAGCACTTTCCTGCAAAGCTCTATTTTATCTTTTTAAATAAAAAATGGTTACTGATAAGTGACCGGTTCT
TGCAAA
```

```
NR_103821.1                              -GGCGTCGGGCCTGGCGGGAAAGTGGAAAACTATCGGCGGTGGCCCAGGCTCGGTGGCTC    59
i1_HQ_samplec9816d|c26333/f2p57/1162     ------------------------------------------------------------    0
i1_HQ_samplec9816d|c26920/f2p0/1048      ------------------------------------------------------------    0
NM_018447.3                              GGGGGTGGGGCCAGGCG---------GCGGCGAGCGGCGGCGGGCGGGGCTTTTAACGGC    51

NR_103821.1                              ACTCCTGGAATCCCAGCACTTTGGGAGGCCAAGGTGGGGGGATCACGAGGTCAGGGGATC    119
i1_HQ_samplec9816d|c26333/f2p57/1162     ------------------------------------------------------------    0
i1_HQ_samplec9816d|c26920/f2p0/1048      ------------------------------------------------------------    0
NM_018447.3                              CAT---------------------------------------------------------    54

NR_103821.1                              GAGACCATCCTGGCTAACAGGTGAAACCCTGTCTCTACTAAAAATACAAAAAAATTAGCC    179
i1_HQ_samplec9816d|c26333/f2p57/1162     ------------------------------------------------------------    0
i1_HQ_samplec9816d|c26920/f2p0/1048      ------------------------------------------------------------    0
NM_018447.3                              ------------------------------------------------------------    54

NR_103821.1                              AGGCGTGGTGGAGGCGCCTGTAGTCGCAGCTACTCGGGAGGCTGAGGCAGGAGAATGGCG    239
i1_HQ_samplec9816d|c26333/f2p57/1162     ------------------------------------------------------------    0
i1_HQ_samplec9816d|c26920/f2p0/1048      ------------------------------------------------------------    0
NM_018447.3                              ------------------------------------------------------------    54

NR_103821.1                              TGAACCCAGGAGGCGGAGCCTGCAGTGAGCCGAGATTGCTGCACTGCACTCCAGCCTGGG    299
i1_HQ_samplec9816d|c26333/f2p57/1162     ------------------------------------------------------------    0
i1_HQ_samplec9816d|c26920/f2p0/1048      ------------------------------------------------------------    0
NM_018447.3                              ------------------------------------------------------------    54
```

```
NR_103821.1                               CGACAGAGGAAGACTCCGTCTCAAAAAAAAAAAAAAAAAAAAAGAAAGAAAACTACAGGCGG    359
i1_HQ_samplec9816d|c26333/f2p57/1162      ------------------------------------------------------------    0
i1_HQ_samplec9816d|c26920/f2p0/1048       ------------------------------------------------------------    0
NM_018447.3                               ------------------------------------------------------------    54

NR_103821.1                               GGACGGCTTCTCGGCAATTGAAAACACTGCCTCAGTATCTGAACACAAGTAACAGAGTAT    419
i1_HQ_samplec9816d|c26333/f2p57/1162      ------------------------------------------------------------    0
i1_HQ_samplec9816d|c26920/f2p0/1048       ------------------------------------------------------------    0
NM_018447.3                               ------------------------------------------------------------    54

NR_103821.1                               GCAAGAGGAGGAGACCTGCACAACTGGATACACCCAGAAATGTCCCTGGCTCAACAAGCC    479
i1_HQ_samplec9816d|c26333/f2p57/1162      ------------------------------------------------------------    0
i1_HQ_samplec9816d|c26920/f2p0/1048       ------------------------------------------------------------    0
NM_018447.3                               ------------------------------------------------------------    54

NR_103821.1                               TTCCAGCATGACCCTGTGTCTTCCCCCTCCACGCAGAGCACCAGCAACAGTAGTGCAGAC    539
i1_HQ_samplec9816d|c26333/f2p57/1162      ------------------------------------------------------------    0
i1_HQ_samplec9816d|c26920/f2p0/1048       ------------------------------------------------------------    0
NM_018447.3                               ------------------------------------------------------------    54

NR_103821.1                               CCAGAAACCTCCCTGGCTCAACAAGGCCCCCAGCATGACCCCATGGCCTTCCCCTCCACC    599
i1_HQ_samplec9816d|c26333/f2p57/1162      ------------------------------------------------------------    0
i1_HQ_samplec9816d|c26920/f2p0/1048       ------------------------------------------------------------    0
NM_018447.3                               --CGGCCCCTCCCAGCCTGGACAAA-------------------------------GACA    81

NR_103821.1                               CAGAGCACCTGTAGCAGTGGTGGAGTCCCAGAAATGTCCCTGGCTCAACAAGCCTTCCAG    659
i1_HQ_samplec9816d|c26333/f2p57/1162      ------------------------------------------------------------    0
i1_HQ_samplec9816d|c26920/f2p0/1048       ------------------------------------------------------------    0
NM_018447.3                               ----------------------------CAGAGCGCGGCCTGGGAGGCGGAGCTCTGCGG    113

NR_103821.1                               TATCAGCCTGTGGCCTTCTTCTCCACCCAGAGCACCAGCAGCAGTGGTGGAGACCCAGAA    719
i1_HQ_samplec9816d|c26333/f2p57/1162      ------------------------------------------------------------    0
i1_HQ_samplec9816d|c26920/f2p0/1048       ------------------------------------------------------------    0
NM_018447.3                               GCGGCCGGGGCCGCCTCCTTCCAGGCGCTGCTGCCCCAACACAGGCGGGCACTCTCTGCG    173

NR_103821.1                               ACATCCCAGGCTCAACAAGCCGCCCAGCATGACCCTGTGGCCTACCCCTATACCCAGAGC    779
i1_HQ_samplec9816d|c26333/f2p57/1162      ------------------------------------------------------------    0
i1_HQ_samplec9816d|c26920/f2p0/1048       ------------------------------------------------------------    0
NM_018447.3                               GGTCCCGCGGCTCCC---------------------GCGG-CTTCCGGCCCACTCAG---    208

NR_103821.1                               AACAGCAGCAGTGGTGCAGACCCAGAAACCTCTGGCTCACCACCTCCCCAGCAACACCCT    839
i1_HQ_samplec9816d|c26333/f2p57/1162      ------------------------------------------------------------    0
i1_HQ_samplec9816d|c26920/f2p0/1048       ------------------------------------------------------------    0
NM_018447.3                               ----------------CGCCTCCAGAGGCCTCAGGCCCGACGCCGCTCTGACGCCAC--T    250

NR_103821.1                               GTGGCCTTCTCCACCAAGAGCACCAGTAGCAGTGGCAGAGACCCAGAAATGTCCCTGACA    899
i1_HQ_samplec9816d|c26333/f2p57/1162      ------------------------------------------------------------    0
i1_HQ_samplec9816d|c26920/f2p0/1048       ------------------------------------------------------------    0
NM_018447.3                               --GAGGTGCGCCACGACGTG-------------------------ACGTCAACGTTCGCG    283

NR_103821.1                               CAGCAACAACCATCCCAGGAAGCCAGTGTCATTCAGGCTGGGCAGCCCAAGGCTTTGACT    959
i1_HQ_samplec9816d|c26333/f2p57/1162      -------------------------------------------agctgaaggcagtaagc    17
i1_HQ_samplec9816d|c26920/f2p0/1048       -------------------------------------------agctgaaggcagtaagc    17
NM_018447.3                               CCGGAAGTGGAA------------------GACCAGGCAGCCCAGCTGAAGGCAGTAAGC    325
                                                                                     ***  *****  * *

NR_103821.1                               TCAGCCTTGCCAATAAGGAGGCTGTGA-----CTGCCGCAGGGCTGCCAGGGGATGCACT    1014
i1_HQ_samplec9816d|c26333/f2p57/1162      tcggctcacagtcgcaggagagttctggggtacACGGGCAAAGGGGCTTGAGAAGGCCCG    77
i1_HQ_samplec9816d|c26920/f2p0/1048       tcggctcacagtcgcaggagagttctggggtacacgggcaaagggccttgaGAA-GCCCG    76
NM_018447.3                               TCGGCTCACAGTCGCAGGAGAGTTCTGGGGTACACGGGCAAAGGGGCTTGAGAAGGCCCG    385
                                          ** **          *****  *         *    ***  *   *  * * * ** *

NR_103821.1                               TCCATTGTATTAAAATGTTTTTGCCTGTGTCCACCGCCGAAAAGGAGGAATAAGATGACC    1074
i1_HQ_samplec9816d|c26333/f2p57/1162      G-AGGCGAAGCCGAAGA--GAAGCAACTGTGCCCCGGAGAAGAGAAGCT---CGCCCATT    131
i1_HQ_samplec9816d|c26920/f2p0/1048       G-AGGCGAAGCCGAAGA--GAAGCAACTGTGCCCCGGAGAAGAGAAGCT---CGCCCATT    130
NM_018447.3                               G-AGGCGAAGCCGAAGA--GAAGCAACTGTGCCCCGGAGAAGAGAAGCT---CGCCCATT    439
                                                * *    **       **   *** * ***  *** ** **      *   *

NR_103821.1                               CCAACATAGTTGCACGGCTGAAGACAAAAATCAAGTTGGTGGGGTCAATATTGTTACCAT    1134
i1_HQ_samplec9816d|c26333/f2p57/1162      CCAGAC-------TGGGAACCAGCTTTCAGTGAAGATGGCAGGGCCAGAACTGTTGCTCG    184
i1_HQ_samplec9816d|c26920/f2p0/1048       CCAGAC-------TGGGAACCAGCTTTCAGTGAAGATGGCAGGGCCAGAACTGTTGCTCG    183
NM_018447.3                               CCAGAC-------TGGGAACCAGCTTTCAGTGAAGATGGCAGGGCCAGAACTGTTGCTCG    492
                                          ***            **    **     * * *** ***  *** **  * **** *

NR_103821.1                               AGTCACTAC----ACTTAGAGTAGTTT------------TA----TAGTCAGTGTAGACA    1174
i1_HQ_samplec9816d|c26333/f2p57/1162      ACTCCAACATCCGCCTCTGGGTGGTCCTACCCATCGTTATCATCACTTTCTTCGTAGGCA    244
i1_HQ_samplec9816d|c26920/f2p0/1048       ACTCCAACATCCGCCTCTGGGTGGTCCTACCCATCGTTATCATCACTTTCTTCGTAGGCA    243
NM_018447.3                               ACTCCAACATCCGCCTCTGGGTGGTCCTACCCATCGTTATCATCACTTTCTTCGTAGGCA    552
                                          * **          **  * ** **              *        **   **** **

NR_103821.1                               GGTGGCACCATGCAGCAGGGATCAGCCTCCACC------------ACACCTGACCAGGAG    1222
i1_HQ_samplec9816d|c26333/f2p57/1162      TGATCCGCCACTACGTGTCCATCCTGCTGCAGAGCGACAAGAAGCTCACCCAGGAACAAG    304
i1_HQ_samplec9816d|c26920/f2p0/1048       TGATCCGCCACTACGTGTCCATCCTGCTGCAGAGCGACAAGAAGCTCACCCAGGAACAAG    303
```

```
NM_018447.3                             TGATCCGCCACTACGTGTCCATCCTGCTGCAGAGCGACAAGAAGCTCACCCAGGAACAAG    612
                                         *   * ***    *     ***   ** **               ****     *  **

NR_103821.1                             CTCCAGAACTGTAAAATCCTGGACACCATTGGCCGTGGCACGTTCAGTGAGGTCCAGGAT    1282
i1_HQ_samplec9816d|c26333/f2p57/1162    TATCTGACAGAAAATATCTTTCAGGCCTGGTGCAATGGCTCACACCTATAATCCCAGCAC    364
i1_HQ_samplec9816d|c26920/f2p0/1048     TATCTGACA---------------------------------------------------    312
NM_018447.3                             TATCTGACA---------------------------------------------------    621
                                           * **

NR_103821.1                             C--------ACATGCTGATTGGGACCCAAATGGCCATCAAAATCATCCCCAAGGCTGGCT    1334
i1_HQ_samplec9816d|c26333/f2p57/1162    TTTGGGGGGCTGAGATGGGAGGATCCCTTGAGCCCAGGAATTT-GAGACCAACCTTGGCA    423
i1_HQ_samplec9816d|c26920/f2p0/1048     ------------------------------------------------------------    312
NM_018447.3                             ------------------------------------------------------------    621

NR_103821.1                             ----CCCTTGGCATCACTCTCCAGAGAGTGATAAGT--ATTTTAAAGTTACTCTGTCACT    1388
i1_HQ_samplec9816d|c26333/f2p57/1162    GCATGTCAAGTCCTAATTCGAAGCAGAGTCCTCAGGGAAAATGGAAAATACATTCCCAAA    483
i1_HQ_samplec9816d|c26920/f2p0/1048     ----GTCAAGTCCTAATTCGAAGCAGAGTCCTCAGGGAAAATGGAAAATACATTCCCAAA    368
NM_018447.3                             ----GTCAAGTCCTAATTCGAAGCAGAGTCCTCAGGGAAAATGGAAAATACATTCCCAAA    677
                                              *  * * * * **     *****  * **   *  *  **  ***  *  **

NR_103821.1                             TCAATATTGTACGGTTGTATCAAGTGATTGACACCCCCAACACCAGTTATTTATTTAGTA    1448
i1_HQ_samplec9816d|c26333/f2p57/1162    -CAGTCTTTCTTGACACGAAAATATTATTTCAACAACCCAGAGGATGGATTTTTCAAAAA    542
i1_HQ_samplec9816d|c26920/f2p0/1048     -CAGTCTTTCTTGACACGAAAATATTATTTCAACAACCCAGAGGATGGATTTTTCAAAAA    427
NM_018447.3                             -CAGTCTTTCTTGACACGAAAATATTATTTCAACAACCCAGAGGATGGATTTTTCAAAAA    736
                                         ** * **    *     *  *  * ***   **  ** * *  *   **** *  *  *

NR_103821.1                             ACGGAGTATGCAAGAGGAGGACACCCACGCAACCAATACACCACCATGGCCTCATGAGGG    1508
i1_HQ_samplec9816d|c26333/f2p57/1162    AACTA-AACGGAAGGTAGTGCCACCTT---CTCCTATGACTGATCCTACT----------    588
i1_HQ_samplec9816d|c26920/f2p0/1048     AACTA-AACGGAAGGTAGTGCCACCTT---CTCCTATGACTGATCCTACT----------    473
NM_018447.3                             AACTA-AACGGAAGGTAGTGCCACCTT---CTCCTATGACTGATCCTACT----------    782
                                        *   *  * * ***     * ****       ** **     * * *

NR_103821.1                             AGGAGAAGGCCTAGACCATGTTCAGGCAGATTCTGTCGGCCATGCAGTAGTGCCATAGCA    1568
i1_HQ_samplec9816d|c26333/f2p57/1162    -----------------ATGTTGAC------------AGACATGATGAAAGGG-------    612
i1_HQ_samplec9816d|c26920/f2p0/1048     -----------------ATGTTGAC------------AGACATGATGAAAGGG-------    497
NM_018447.3                             -----------------ATGTTGAC------------AGACATGATGAAAGGG-------    806
                                                         ***** *              * ****  * *  *

NR_103821.1                             AATTGCGCAGAGACCTGAACCCAGAAAACATCATCCTTGATGAGGACGGTAACGTTAAGA    1628
i1_HQ_samplec9816d|c26333/f2p57/1162    -----------AATGTAACAAATGTCCTCCCTATGATTCTTATTGGTGGATGGATCAACA    661
i1_HQ_samplec9816d|c26920/f2p0/1048     -----------AATGTAACAAATGTCCTCCCTATGATTCTTATTGGTGGATGGATCAACA    546
NM_018447.3                             -----------AATGTAACAAATGTCCTCCCTATGATTCTTATTGGTGGATGGATCAACA    855
                                                    *  * *     *    *   **  **  *   *  **     * ** *

NR_103821.1                             TCGCAGACTTCGGCTTTGGTACCACATTCCATGATGGGCAGAAGCTGACAGCCCTTTGTA    1688
i1_HQ_samplec9816d|c26333/f2p57/1162    TGACATTCTCAGGCTTTGTCACAACCAAG---GTCCCATTTCCACTGACCCTCCGTTTTA    718
i1_HQ_samplec9816d|c26920/f2p0/1048     TGACATTCTCAGGCTTTGTCACAACCAAG---GTCCCATTTCCACTGACCCTCCGTTTTA    603
NM_018447.3                             TGACATTCTCAGGCTTTGTCACAACCAAG---GTCCCATTTCCACTGACCCTCCGTTTTA    912
                                        *  **  **  *******  ** **       *           *****   ** ** **

NR_103821.1                             GCACTTAACCCCTACATGGCCCTGGAACGTTTCCTAGGCCAGGGCTACCAATGTGCCACC    1748
i1_HQ_samplec9816d|c26333/f2p57/1162    -------------------------AGCCTATGTTACAGCAAGGAATCGAGCTACTCACA    753
i1_HQ_samplec9816d|c26920/f2p0/1048     -------------------------AGCCTATGTTACAGCAAGGAATCGAGCTACTCACA    638
NM_018447.3                             -------------------------AGCCTATGTTACAGCAAGGAATCGAGCTACTCACA    947
                                                                 * * * *  **   ** **   * *      ***

NR_103821.1                             A-----------TGGATATTCAGAGCCTCAGAGTAATTTTATACCACATGGTGGCTGGGG    1797
i1_HQ_samplec9816d|c26333/f2p57/1162    TTAGATGCATCCTGGGTGAGTTCTGCATCCTGGTACTT-----CCTCAATGTATTTGGGC    808
i1_HQ_samplec9816d|c26920/f2p0/1048     TTAGATGCATCCTGGGTGAGTTCTGCATCCTGGTACTT-----CCTCAATGTATTTGGGC    693
NM_018447.3                             TTAGATGCATCCTGGGTGAGTTCTGCATCCTGGTACTT-----CCTCAATGTATTTGGGC    1002
                                                    *** *       ** **   *** **     ** **  **   ****

NR_103821.1                             TTCTGCCCTTCTGCTCATGCAGCATTAGGGTCCTCTCAGCAAAAATTTAAAGTGGAAGCT    1857
i1_HQ_samplec9816d|c26333/f2p57/1162    TTCGGAGCATTTACTCTCTGAT--------------------------------------    830
i1_HQ_samplec9816d|c26920/f2p0/1048     TTCGGAGCATTTACTCTCTGAT--------------------------------------    715
NM_018447.3                             TTCGGAGCATTTACTCTCTGAT--------------------------------------    1024
                                        *** *  * * * ***    *

NR_103821.1                             ATTTTTCCCCAGTCTACTTTTCCTGAGGTCTTAAAAGCCTCATTAAAAAACTATTAACAG    1917
i1_HQ_samplec9816d|c26333/f2p57/1162    --TCTGGGCCAAGATAATGCCGCTGACCA--ATCACGAATGATGCAGGAGCAGATGACGG    886
i1_HQ_samplec9816d|c26920/f2p0/1048     --TCTGGGCCAAGATAATGCCGCTGACCA--ATCACGAATGATGCAGGAGCAGATGACGG    771
NM_018447.3                             --TCTGGGCCAAGATAATGCCGCTGACCA--ATCACGAATGATGCAGGAGCAGATGACGG    1080
                                          * *   ***   ** *    ****        * *  * **  *  * *   * ** *

NR_103821.1                             TAGACCCCAGGGAGCAGACCACAC---TAGAAGAAGTTATGA---------GGGACCCGT    1965
i1_HQ_samplec9816d|c26333/f2p57/1162    GAGCAGCCATGGCCATGCCCGCAGACACAAACAAAGCTTTCAAGACAGAGTGGGAAGCTT    946
i1_HQ_samplec9816d|c26920/f2p0/1048     GAGCAGCCATGGCCATGCCCGCAGACACAAACAAAGCTTTCAAGACAGAGTGGGAAGCTT    831
NM_018447.3                             GAGCAGCCATGGCCATGCCCGCAGACACAAACAAAGCTTTCAAGACAGAGTGGGAAGCTT    1140
                                         **   *** **    * ** **     * *  *** * * *         ****  * *

NR_103821.1                             GGGTGAACAGTGGTCAGGAGTTGCCTCTG--ACAACATGAAGAACAAATCCTGGACCACC    2023
i1_HQ_samplec9816d|c26333/f2p57/1162    TGGAGCTGACGGATCACCAGTGGGCACTAGATGATGTCGAAGAAGAGCTCATGGCCAAAG    1006
i1_HQ_samplec9816d|c26920/f2p0/1048     TGGAGCTGACGGATCACCAGTGGGCACTAGATGATGTCA-AGAAGAGCTCATGGCCAAAG    890
NM_018447.3                             TGGAGCTGACGGATCACCAGTGGGCACTAGATGATGTCGAAGAAGAGCTCATGGCCAAAG    1200
                                         ** *   *  * ***  *** * * **     *      **** *  ** *** * *

NR_103821.1                             TGAATCCCAAAACAACCCAGCTTTTGGTGGCCATGGGATTCCAGGCTGAGA---------    2074
i1_HQ_samplec9816d|c26333/f2p57/1162    AC-CTCCACTTCGAAGGC-----ATGTTCAAAAAGGAATTACAGACCTCTATTTTTTGAA    1060
i1_HQ_samplec9816d|c26920/f2p0/1048     AC-CTCCACTTCGAAGGC-----ATGTTCAAAAAGGAATTACAGACCTCTATTTTTTGAA    944
NM_018447.3                             AC-CTCCACTTCGAAGGC-----ATGTTCAAAAAGGAATTACAGACCTCTATTTTTTGAA    1254
                                            ***      **  *      ** *    * ** *** *** *    *

NR_103821.1                             -ACCTATCTGTGGCAATCAAAGAAAAATTATTCAGTTATCCCATGGCCACCTACCTTGTT    2133
```

```
i1_HQ_samplec9816d|c26333/f2p57/1162       GACCGAGCAGGGATTAGCTGTGTCAGGAACTTGGAGTTGCACTTAACCTTGTAACTTTGT    1120
i1_HQ_samplec9816d|c26920/f2p0/1048        GACCGAGCAGGGATTAGCTGTGTCAGGAACTTGGAGTTGCACTTAACCTTGTAACTTTGT    1004
NM_018447.3                                GACCGAGCAGGGATTAGCTGTGTCAGGAACTTGGAGTTGCACTTAACCTTGTAACTTTGT    1314
                                            *** * * * *    * *    *  *      **     *  * * *  **    ** ***  *

NR_103821.1                                TTGGAACAAACAAAACAGAA-------GAAGCGGTCCACTATCAGACCACAGACCCTTCC    2186
i1_HQ_samplec9816d|c26333/f2p57/1162       TTGGAgctggcacctcttgaaataaaaaggaggatgcacgag------------------    1162
i1_HQ_samplec9816d|c26920/f2p0/1048        TTGGAGCTggcacctcttgaaataaaaaggaggatgcacgagcg----------------    1048
NM_018447.3                                TTGGAGCTGGCACCTCTTGAAATAAAAAGGAGGATGCACGAGCTGGCAG-----GCATGC    1369
                                           ***** *    **    *    *             * * *** *

NR_103821.1                                TCCTGGGGATCCCACTTGTCCTCTCTACATTGAAGTTTCCACCTTCCCTCTTTCACTGAA    2246
i1_HQ_samplec9816d|c26333/f2p57/1162       ------------------------------------------------------------    1162
i1_HQ_samplec9816d|c26920/f2p0/1048        ------------------------------------------------------------    1048
NM_018447.3                                AGCA-AGGCTTGTTCTTGTCTGGGCTGGG-------TTCCCCTTTATGTTTGAAAC-TAG    1420

NR_103821.1                                GCGGGCTCATAGCATTCAGCAGAAGACTGTGGGTGCCAAGTCTGGGCAGGGCCTTTGCCC    2306
i1_HQ_samplec9816d|c26333/f2p57/1162       ------------------------------------------------------------    1162
i1_HQ_samplec9816d|c26920/f2p0/1048        ------------------------------------------------------------    1048
NM_018447.3                                AGGAAATAGGAGTATGCTGGGCAACTTGTTATTTTTAAAATGTTGTAAA-----------    1469

NR_103821.1                                TTGGGAGTCCTGTTTTAGACCCAGCTCCACCACGTGGCCCTGGCCTCTTCTCTGCCCTGT    2366
i1_HQ_samplec9816d|c26333/f2p57/1162       ------------------------------------------------------------    1162
i1_HQ_samplec9816d|c26920/f2p0/1048        ------------------------------------------------------------    1048
NM_018447.3                                ---------------------------------------------------------TGT    1472

NR_103821.1                                CTGTGCTTGCAGCCATGGTGCCCAGTCTGGCCCCACCAGGAGACACCAGAACGACTGTCC    2426
i1_HQ_samplec9816d|c26333/f2p57/1162       ------------------------------------------------------------    1162
i1_HQ_samplec9816d|c26920/f2p0/1048        ------------------------------------------------------------    1048
NM_018447.3                                TCCTTCTGGTGACTCTAGTAATGAAAGTCAGAGAAAGGGGGAAACCTCAGGCTACTGATA    1532

NR_103821.1                                ATGTGTCTACTCCTTGGCACC--------------------TTCCCCGCATGGTGGGTAG    2466
i1_HQ_samplec9816d|c26333/f2p57/1162       ------------------------------------------------------------    1162
i1_HQ_samplec9816d|c26920/f2p0/1048        ------------------------------------------------------------    1048
NM_018447.3                                ATATATAAAATCTTGGCCAGGCGCAGTGGCTCACGCCTGTAATCCCAGGATTTTGGGAGG    1592

NR_103821.1                                ATGGGCAGGGAGGGGT---------GGG------AATCTGTGTAACCGTGGAAGGGAACG    2511
i1_HQ_samplec9816d|c26333/f2p57/1162       ------------------------------------------------------------    1162
i1_HQ_samplec9816d|c26920/f2p0/1048        ------------------------------------------------------------    1048
NM_018447.3                                CTGAGGCGGGTGGGTCACGAGATCAGGAGTTCAAGATCAGCCTGGCCAAGATGGTGAAAC    1652

NR_103821.1                                GCCCTCAGACCCA--------GAGGGTTTGGAGCCTGGGGT----ATTGTCACCGACAGT    2559
i1_HQ_samplec9816d|c26333/f2p57/1162       ------------------------------------------------------------    1162
i1_HQ_samplec9816d|c26920/f2p0/1048        ------------------------------------------------------------    1048
NM_018447.3                                CCCATCTCTACTAAAAATACAAAAAAAAATTAGCCGGGCGTGGTGGTGGGCACCTGTAAT    1712

NR_103821.1                                GGCCTTAGCCTAGCTAGCACAGTTAGTGCTGTGCATCACAGCATAGCTTTTAGAGGCAGG    2619
i1_HQ_samplec9816d|c26333/f2p57/1162       ------------------------------------------------------------    1162
i1_HQ_samplec9816d|c26920/f2p0/1048        ------------------------------------------------------------    1048
NM_018447.3                                C--------CCAGCTACTTGGGAGGCTGAGGCAGAGAACTGCTTAAACCTAGGAGGCGGA    1764

NR_103821.1                                ATTTGTAGTCAGTATAG---------TTGGGAGGCACCATGGAGCAG-GGATCACCCTCC    2669
i1_HQ_samplec9816d|c26333/f2p57/1162       ------------------------------------------------------------    1162
i1_HQ_samplec9816d|c26920/f2p0/1048        ------------------------------------------------------------    1048
NM_018447.3                                GGATGCAGTGAGCTGACATCGTGCCACTGCGCTCCAGCCTGGGCGACAGAGCAAAACTCC    1824

NR_103821.1                                ACCTCACTTGACAAGGAGATACAAAACTATCAGTTCATAGACACCATCTGATAGGGAACT    2729
i1_HQ_samplec9816d|c26333/f2p57/1162       ------------------------------------------------------------    1162
i1_HQ_samplec9816d|c26920/f2p0/1048        ------------------------------------------------------------    1048
NM_018447.3                                AT-----CTGAAAAAAAAAAAAAAAATATATATATATAT----------ATATATATATCT    1869

NR_103821.1                                GGCTCAGCATAGGCCAACTGGG------ACCCAGG------TTGCCATCTTGAAGACTTT    2777
i1_HQ_samplec9816d|c26333/f2p57/1162       ------------------------------------------------------------    1162
i1_HQ_samplec9816d|c26920/f2p0/1048        ------------------------------------------------------------    1048
NM_018447.3                                ATCTTAGCAGAAGCCCAGTCTTCGAAGGAAGCATTGTTAATTATAGCTAATGATTATGTG    1929

NR_103821.1                                CCATCACCCAAATATCATTCAGCTCTTCCAGGTGGTGAGGGAGTAAACCAGAGGAGGAGA    2837
i1_HQ_samplec9816d|c26333/f2p57/1162       ------------------------------------------------------------    1162
i1_HQ_samplec9816d|c26920/f2p0/1048        ------------------------------------------------------------    1048
NM_018447.3                                CAGTCAGCCTGACATCTAGCCACAGTAAACTCCTGTTTTCTTGGGATCCAAACACCCTGT    1989

NR_103821.1                                GTTGCACCACCAGATATACCACTATGGCCACATCGAGGAGGAAGAGGAGGCCCG------    2891
i1_HQ_samplec9816d|c26333/f2p57/1162       ------------------------------------------------------------    1162
i1_HQ_samplec9816d|c26920/f2p0/1048        ------------------------------------------------------------    1048
NM_018447.3                                GTTTTACCATTAGTTTTGTTAGTATTGTTAACTTTCTTTACACAAGTAAACCATTTCCAC    2049

NR_103821.1                                ---------------GACCATGTTCAGGCAGATTCTGTCAGCCCTGCAGTACTGCCACTT    2936
i1_HQ_samplec9816d|c26333/f2p57/1162       ------------------------------------------------------------    1162
i1_HQ_samplec9816d|c26920/f2p0/1048        ------------------------------------------------------------    1048
NM_018447.3                                TACCAAAGGTTTAGAAACTATGGATGAACAAAATGAATCTCCCTGTTCATTTTGAGCCTA    2109
```

```
NR_103821.1                              AAAAAAAAAAAAAAAAATCACACATCCAGACCTAAAGCCACAAAACATCCTATTCAAAGAA    2996
i1_HQ_samplec9816d|c26333/f2p57/1162     ------------------------------------------------------------    1162
i1_HQ_samplec9816d|c26920/f2p0/1048      ------------------------------------------------------------    1048
NM_018447.3                              CATAGACATAGAGACCAAATATGTCAAAACCTTATTCAAATAAACTC--TCTTCAGA---    2164

NR_103821.1                              TGAGGCAACATACATTGTGGACTTTGGCTTCAGTACAACATTCAGAGAGGGGCAGATGCT    3056
i1_HQ_samplec9816d|c26333/f2p57/1162     ------------------------------------------------------------    1162
i1_HQ_samplec9816d|c26920/f2p0/1048      ------------------------------------------------------------    1048
NM_018447.3                              --------------------------------GTGAATACCCAGGGTCAGGACAGACTCA    2192

NR_103821.1                              GACAGCTTTTTGTGGCA-------------TGTACCCCTACGTGGCCCCAGAACGCTCCC    3103
i1_HQ_samplec9816d|c26333/f2p57/1162     ------------------------------------------------------------    1162
i1_HQ_samplec9816d|c26920/f2p0/1048      ------------------------------------------------------------    1048
NM_018447.3                              GCAAACCTTCTATATCACTTAATTCTGCCCAGTGAAAATCAGTGACCCTGAGGCACTGCC    2252

NR_103821.1                              TGGGCCAGGCATGCCAGTGACCCGCCAGGGACATACAAAGCCTCAGTGTCATACTGTATT    3163
i1_HQ_samplec9816d|c26333/f2p57/1162     ------------------------------------------------------------    1162
i1_HQ_samplec9816d|c26920/f2p0/1048      ------------------------------------------------------------    1048
NM_018447.3                              AGATTCTAAAGT-----------------GCCTTTGGGATCATCAGAGA----CTGTCTA    2291

NR_103821.1                              TCAGGAATACAGTAGGTAGAAGGGCCAGGACTTTGCCCTTTTACTCAGGGAAGCCTCCAA    3223
i1_HQ_samplec9816d|c26333/f2p57/1162     ------------------------------------------------------------    1162
i1_HQ_samplec9816d|c26920/f2p0/1048      ------------------------------------------------------------    1048
NM_018447.3                              TAAATAATGCAAAATCTTGATCTGGATGTCAGTGTATCAAATCTTATCAAATCCATTGAC    2351

NR_103821.1                              ACTTCAAGA-AAAAATTCTC--ACAG----GAAGATACCATGCCCCACCACTTCTTGCCC    3276
i1_HQ_samplec9816d|c26333/f2p57/1162     ------------------------------------------------------------    1162
i1_HQ_samplec9816d|c26920/f2p0/1048      ------------------------------------------------------------    1048
NM_018447.3                              AAATCAGTGGTGCTATACTGGAAATGGCTACTCCTTTGGATGAAGGACCTCATTTCCCAG    2411

NR_103821.1                              TTCAACTTGACTCATTAAAAAATTACTAATGCTGAACGCCAGGAAGTGTCCTTCACTGTA    3336
i1_HQ_samplec9816d|c26333/f2p57/1162     ------------------------------------------------------------    1162
i1_HQ_samplec9816d|c26920/f2p0/1048      ------------------------------------------------------------    1048
NM_018447.3                              TTGCTTGGAACAAAAGAGAATTTACATATTGCTGTAAGAA-GGGAGAGACGGCAGTTGTT    2470

NR_103821.1                              ACTGATGAAAAATCCATGGGTGAAAAGTAGCCAGAAGATGCCACTGATACCATACGAAGA    3396
i1_HQ_samplec9816d|c26333/f2p57/1162     ------------------------------------------------------------    1162
i1_HQ_samplec9816d|c26920/f2p0/1048      ------------------------------------------------------------    1048
NM_018447.3                              GGTAGACACACAGATAAGGGTGCCC--------AAGAATAATTCTGTCTGGATTAAAGGT    2522

NR_103821.1                              GCCACTCCTGGACCACCCCAAACAATCCAGCTCATGGTGGCCATGGGATTTCAGGCCAAG    3456
i1_HQ_samplec9816d|c26333/f2p57/1162     ------------------------------------------------------------    1162
i1_HQ_samplec9816d|c26920/f2p0/1048      ------------------------------------------------------------    1048
NM_018447.3                              CCCAGGGAAGTGACGGTGCCATTTCTGTACCAAGAGGTGA--ATATAATTTG-------C    2573

NR_103821.1                              AACATCTCTGTGGCAATCATAGAAAGAAAATTCAACTATCCCATGGCCACCTACCTCATT    3516
i1_HQ_samplec9816d|c26333/f2p57/1162     ------------------------------------------------------------    1162
i1_HQ_samplec9816d|c26920/f2p0/1048      ------------------------------------------------------------    1048
NM_018447.3                              ATGTTGAGTGCAGTAATTAAGAACAAACGACTCGACTCCACAAATTAAACCGTCCTGTTT    2633

NR_103821.1                              TTAGAGCACACAAAACAAGAGAGGAAGTGCTCCACCATCAGAGAACTGTCCCTTCCTCCC    3576
i1_HQ_samplec9816d|c26333/f2p57/1162     ------------------------------------------------------------    1162
i1_HQ_samplec9816d|c26920/f2p0/1048      ------------------------------------------------------------    1048
NM_018447.3                              TCTCTTCTTAAAAAAAAAAAAAAAAAA---------------------------------    2660

NR_103821.1                              GGGGTTCCCACCTCTCCTTCCCCATCCACTGAACTTTCCACCTTCCCTCTCTCACTGATG    3636
i1_HQ_samplec9816d|c26333/f2p57/1162     ------------------------------------------------------------    1162
i1_HQ_samplec9816d|c26920/f2p0/1048      ------------------------------------------------------------    1048
NM_018447.3                              ------------------------------------------------------------    2660

NR_103821.1                              CGGGCTCATAGGGAGCCAGCTTTTAACGTTCAGCCTCCCGAAGAAAGCCAGATGTCAGGG    3696
i1_HQ_samplec9816d|c26333/f2p57/1162     ------------------------------------------------------------    1162
i1_HQ_samplec9816d|c26920/f2p0/1048      ------------------------------------------------------------    1048
NM_018447.3                              ------------------------------------------------------------    2660

NR_103821.1                              CAGAAGACCGCCATTCTTGCCAGTACACCTGCCAGCCTACAGAGGAAGCCAGATCGGGGA    3756
i1_HQ_samplec9816d|c26333/f2p57/1162     ------------------------------------------------------------    1162
i1_HQ_samplec9816d|c26920/f2p0/1048      ------------------------------------------------------------    1048
NM_018447.3                              ------------------------------------------------------------    2660

NR_103821.1                              AGCCAGGAAGTTTTAGTGAAGCCCCCAGCATGACCCTATTGTCTCCCCTTTCACCCAGAG    3816
i1_HQ_samplec9816d|c26333/f2p57/1162     ------------------------------------------------------------    1162
i1_HQ_samplec9816d|c26920/f2p0/1048      ------------------------------------------------------------    1048
NM_018447.3                              ------------------------------------------------------------    2660

NR_103821.1                              CACCAGCAACTGGTGAAGTCTTAGAAACGTCCCTGATACACCAAAAACCATCCCAGGAAG    3876
i1_HQ_samplec9816d|c26333/f2p57/1162     ------------------------------------------------------------    1162
i1_HQ_samplec9816d|c26920/f2p0/1048      ------------------------------------------------------------    1048
```

```
NM_018447.3                                 ------------------------------------------------------------    2660

NR_103821.1                                 CCAGCATCGTCCAAGCTGAGAAGTCCAAGCTGAGAAGCCTGAGGCTGTGACGTCAGCCTG    3936
i1_HQ_samplec9816d|c26333/f2p57/1162        ------------------------------------------------------------    1162
i1_HQ_samplec9816d|c26920/f2p0/1048         ------------------------------------------------------------    1048
NM_018447.3                                 ------------------------------------------------------------    2660

NR_103821.1                                 CTCGGCAAGAAGCTGGGGCTGCCGCAGGGTTGCCAGGAGATAGATGCCTTTGCATACTAT    3996
i1_HQ_samplec9816d|c26333/f2p57/1162        ------------------------------------------------------------    1162
i1_HQ_samplec9816d|c26920/f2p0/1048         ------------------------------------------------------------    1048
NM_018447.3                                 ------------------------------------------------------------    2660

NR_103821.1                                 TAAAATATTTTTGCCTGTCTCTACCAAAGAAAAGGAGGAATAAGATCGCCCCAACATAGT    4056
i1_HQ_samplec9816d|c26333/f2p57/1162        ------------------------------------------------------------    1162
i1_HQ_samplec9816d|c26920/f2p0/1048         ------------------------------------------------------------    1048
NM_018447.3                                 ------------------------------------------------------------    2660

NR_103821.1                                 TGCATGGCTGAAGACAAAAATCAAGTCGTTGTGGTGGTCAAGCCACAATTCCTGCATTTT    4116
i1_HQ_samplec9816d|c26333/f2p57/1162        ------------------------------------------------------------    1162
i1_HQ_samplec9816d|c26920/f2p0/1048         ------------------------------------------------------------    1048
NM_018447.3                                 ------------------------------------------------------------    2660

NR_103821.1                                 ACATTTCTTCTGTCTTTATTGTATTGCTTCAATTGGCAAATCATGCTTGTATTCATTCAT    4176
i1_HQ_samplec9816d|c26333/f2p57/1162        ------------------------------------------------------------    1162
i1_HQ_samplec9816d|c26920/f2p0/1048         ------------------------------------------------------------    1048
NM_018447.3                                 ------------------------------------------------------------    2660

NR_103821.1                                 GGGGTACAATGTGGAATGAGGAAATCCCACTACTTAGCATCTCCACTACCTCAGAGAGAC    4236
i1_HQ_samplec9816d|c26333/f2p57/1162        ------------------------------------------------------------    1162
i1_HQ_samplec9816d|c26920/f2p0/1048         ------------------------------------------------------------    1048
NM_018447.3                                 ------------------------------------------------------------    2660

NR_103821.1                                 CAATTCCACGTGAGGTCCCAGAAGTGTTGATCTAAACAAGTTGACCCCATAGAAGTAGCA    4296
i1_HQ_samplec9816d|c26333/f2p57/1162        ------------------------------------------------------------    1162
i1_HQ_samplec9816d|c26920/f2p0/1048         ------------------------------------------------------------    1048
NM_018447.3                                 ------------------------------------------------------------    2660

NR_103821.1                                 AGTAGATCGATGGTGACCAGGGGTCAGAGAGTGGCAGAGGGAGGGAATGGGAGGGGGGTT    4356
i1_HQ_samplec9816d|c26333/f2p57/1162        ------------------------------------------------------------    1162
i1_HQ_samplec9816d|c26920/f2p0/1048         ------------------------------------------------------------    1048
NM_018447.3                                 ------------------------------------------------------------    2660

NR_103821.1                                 GTCAGTTAAAGGACCAAAGTCTCAGGGAGGAGGAAGAGGTTTTGACATGTAGTGCACAGC    4416
i1_HQ_samplec9816d|c26333/f2p57/1162        ------------------------------------------------------------    1162
i1_HQ_samplec9816d|c26920/f2p0/1048         ------------------------------------------------------------    1048
NM_018447.3                                 ------------------------------------------------------------    2660

NR_103821.1                                 AGAGTGACCAGAGTCAATGAGAATGTGTTGCATTTTGCAAAACACCTGAGAGAGTCCATG    4476
i1_HQ_samplec9816d|c26333/f2p57/1162        ------------------------------------------------------------    1162
i1_HQ_samplec9816d|c26920/f2p0/1048         ------------------------------------------------------------    1048
NM_018447.3                                 ------------------------------------------------------------    2660

NR_103821.1                                 TCAAATGTCTCTCTGCATTTAGATTGGAGAGGACGAAGGCCCTGAGGTCCAAGAACATTG    4536
i1_HQ_samplec9816d|c26333/f2p57/1162        ------------------------------------------------------------    1162
i1_HQ_samplec9816d|c26920/f2p0/1048         ------------------------------------------------------------    1048
NM_018447.3                                 ------------------------------------------------------------    2660

NR_103821.1                                 AAACCTGACAGTGGATGCCAATGGCTGTGGGGAGGAGCTGGGCGAGGCGCCCAGGTTGTC    4596
i1_HQ_samplec9816d|c26333/f2p57/1162        ------------------------------------------------------------    1162
i1_HQ_samplec9816d|c26920/f2p0/1048         ------------------------------------------------------------    1048
NM_018447.3                                 ------------------------------------------------------------    2660

NR_103821.1                                 GTCTGCCCGGCAACGTGGGAGCCTGCAGCACTTTCCTGCAAAGCTCTATTTTATCTTTTT    4656
i1_HQ_samplec9816d|c26333/f2p57/1162        ------------------------------------------------------------    1162
i1_HQ_samplec9816d|c26920/f2p0/1048         ------------------------------------------------------------    1048
NM_018447.3                                 ------------------------------------------------------------    2660

NR_103821.1                                 AAATAAAAATGGTTACTGATAAGTGACCGGTTCTTGCAAA 4696
i1_HQ_samplec9816d|c26333/f2p57/1162        ----------------------------------------1162
i1_HQ_samplec9816d|c26920/f2p0/1048         ----------------------------------------1048
NM_018447.3                                 ----------------------------------------2660

VIRT153469          MAGPELLLDSNIRLWVVLPIVIITFFVGMIRHYVSILLQSDKKLTQEQVSD---------     51
VIRT151947          MAGPELLLDSNIRLWVVLPIVIITFFVGMIRHYVSILLQSDKKLTQEQVSDRKYLSGLVQ     60
NP_060917.1         MAGPELLLDSNIRLWVVLPIVIITFFVGMIRHYVSILLQSDKKLTQEQVSD---------     51
                    ***************************************************

VIRT153469          -----------------------------SQVLIRSRVLRENGKYIPKQSFLTRKYYFNN     82
VIRT151947          WLTPIIPALWGAEMGGSLEPRNLRPTLAACQVLIRSRVLRENGKYIPKQSFLTRKYYFNN     120
```

```
NP_060917.1         -----------------------------SQVLIRSRVLRENGKYIPKQSFLTRKYYFNN      82
                                                 .******************************

VIRT153469          PEDGFFKKTKRKVVPPSPMTDPTMLTDMMKGNVTNVLPMILIGGWINMTFSGFVTTKVPF     142
VIRT151947          PEDGFFKKTKRKVVPPSPMTDPTMLTDMMKGNVTNVLPMILIGGWINMTFSGFVTTKVPF     180
NP_060917.1         PEDGFFKKTKRKVVPPSPMTDPTMLTDMMKGNVTNVLPMILIGGWINMTFSGFVTTKVPF     142
                    ************************************************************

VIRT153469          PLTLRFKPMLQQGIELLTLDASWVSSASWYFLNVFGLRSIYSLILGQDNAADQSRMMQEQ     202
VIRT151947          PLTLRFKPMLQQGIELLTLDASWVSSASWYFLNVFGLRSIYSLILGQDNAADQSRMMQEQ     240
NP_060917.1         PLTLRFKPMLQQGIELLTLDASWVSSASWYFLNVFGLRSIYSLILGQDNAADQSRMMQEQ     202
                    ************************************************************

VIRT153469          MTGAAMAMPADTNKAFKTEWEALELTDHQWALDDVKKSSWPKTSTSKACSKRNYRPLFFE     262
VIRT151947          MTGAAMAMPADTNKAFKTEWEALELTDHQWALDDVEEELMAKDLHFEGMFKKELQTSIF-     299
NP_060917.1         MTGAAMAMPADTNKAFKTEWEALELTDHQWALDDVEEELMAKDLHFEGMFKKELQTSIF-     261
                    ***********************************::.   *    :.  *:: :  :*

VIRT153469          DRAGISCVRNLELHLTL     279
VIRT151947          -----------------     299
NP_060917.1         -----------------     261
```

## MCM3

| Sequence | Header | AA Seq |
|---|---|---|
| **Present** | i1_HQ_samplec9816d\|c23601/f2p0/1036 | |
| | | |
| **Absent** | i1_HQ_samplec9816d\|c22727/f2p0/1166 | |
| **NCBI** | NM_002388 | |
| | NM_001270472 | |

>i1_HQ_samplec9816d|c23601/f2p0/1036
isoform=c23601;full_length_coverage=2;non_full_length_coverage=0;isoform_length=1036

ggattcatACGACCCCTATGACTTCAGTGACACAGAGGAGGAAATGCCTCAAGTACACACTCCAAAGACGGCAGACTCAC
AGGAGACCAAGGAATCCCAGAAAGTGGAGTTGAGTGAATCCAGAGACGGGATCTCACCATGTTGCCCAGGCTGGTCTT
GGACTCCTGGCCTCAAGCAGTCCTTCTACCTGAGCCTCCCAAAAGGCTGGGATTACAGGCATAAGCCACTGTGCTTGTT
TGATCTCATTCTTTTTAATGACTGCAGGGTTTCCATGGTTGAAGGCATTCAAGGTGGCCCTCTTGGTGTGTTCCGGGAAG
CTCATGCGCAGTCAATCGGCATGAATCGCCTCACAGAATCCATCAACCGGGACAGCGAAGAGCCCTTCTCTTCAGTTGA
GATCCAGGCTGCTCTGAGCAAGATGCAGGATGACAATCAGGTCATGGTGTCTGAGGGCATCATCTTCCTCATCTGAGG
AGGCCTCGTCTCTGAACTTGGGTTGTGCCGAGAGAGTTTGTTCTGTGTTTCCCACCCTCTCCCTGACCCAAGTCTTTGCCT
CTACTCCCTTAACAGTGTTGAATTCAACTGAAGGCGAGGAATGTTGGTGATGAAGCTGAGTTCAGGACTCGGTGGACC
CTTTGGGAATGGGTCATGAAAGCTGCCATGGGGTGAGGAAAGAGGAGACAGTGGGAGAGGACAATGACTATTGCATC
TTCATTGCAAAAGCACTGGCTCATCCGCCCTACTTCCCATCCCACACAAACCCAATTGTAAATAACATATGACTTCTGAGT
ACTTTTGGGGGCACAACTGTTTTCTGTTTGCTGTTTTTTTGTTTTGTTTTTTTTCTCCAGAGCACTTTGGTCTAGACTAGGC
TTTGGGTGGTTCCAATTGGTGGAGAGAAGCTCTGAGGCACGTCATGCAGGTCAAGAAAGCTTTCTTTGCAGTAGCACC
AGTTAAGGTGAATATGTATTGTATCACAAAACAAACCCAATATCCAGATGAATatccgagatgttgaataaacttagccatttcg

>VIRT161936

MPQVHTPKTADSQETKESQKVELSESRDGISPCCPGWSWTPGLKQSFYLSLPKGWDYRHK
PLCLFDLILFNDCRVSMVEGIQGGPLGVFREAHAQSIGMNRLTESINRDSEEPFSSVEIQ
AALSKMQDDNQVMVSEGIIFLI

>i1_HQ_samplec9816d|c22727/f2p0/1166
isoform=c22727;full_length_coverage=2;non_full_length_coverage=0;isoform_length=1166

aggacatctccagttacagcccgaacactggaaactctgattcgactggccacagcccatgcgaaggcCCGCATGAGCAAGACTGTGGACCTGC
AGGATGCAGAGGAAGCTGTGGAGTTGGTCCAGTATGCTTACTTTAAGAAGGTTCTGGAGAAGGAGAAGAAACGTAAG
AAGCGAAGTGAGGATGAATCAGAGACAGAAGATGAAGAGGAGAAAAGCCAAGAGGACCAGGAGCAGAAGAGGAAG
AGAAGGAAGACTCGCCAGCCAGATGCCAAAGATGGGGATTCATACGACCCCTATGACTTCAGTGACACAGAGGAGGA
AATGCCTCAAGTACACACTCCAAAGACGGCAGACTCACAGGAGACCAAGGAATCCCAGAAAGTGGAGTTGAGTGAATC
CAGGTTGAAGGCATTCAAGGTGGCCCTCTTGGATGTGTTCCGGGAAGCTCATGCGCAGTCAATCGGCATGAATCGCCTC
ACAGAATCCATCAACCGGGACAGCGAAGAGCCCTTCTCTTCAGTTGAGATCCAGGCTGCTCTGAGCAAGATGCAGGAT
GACAATCAGGTCATGGTGTCTGAGGGCATCATCTTCCTCATCTGAGGAGGCCTCGTCTCTGAACTTGGGTTGTGCCGAG
AGAGTTTGTTCTGTGTTTCCCACCCTCTCCCTGACCCAAGTCTTTGCCTCTACTCCCTTAACAGTGTTGAATTCAACTGAA
GGCGAGGAATGTTGGTGATGAAGCTGAGTTCAGGACTCGGTGGACCCTTTGGGAATGGGTCATGAAAGCTGCCATGG
GGTGAGGAAAGAGGAGACAGTGGGAGAGGACAATGACTATTGCATCTTCATTGCAAAAGCACTGGCTCATCCGCCCTA
CTTCCCATCCCACACAAACCCAATTGTAAATAACATATGACTTCTGAGTACTTTTGGGGGCACAACTGTTTTCTGTTTGCT
GTTTTTTTGTTTTGTTTTTTTTCTCCAGAGCACTTTGGTCTAGACTAGGCTTTGGGTGGTTCCAATTGGTGGAGAGAAGCT
CTGAGGCACGTCATGCAGGTCAAGAAAGCTTTCTTTGCAGTAGCACCAGTTAAGGTGAATATGTATTGTATCACAAAAC
AAACCCAATATCCAGATGAATATCcgagatgttgataaacttagccatttcg

>VIRT162782

MSKTVDLQDAEEAVELVQYAYFKKVLEKEKKRKKRSEDESETEDEEEKSQEDQEQKRKRR
KTRQPDAKDGDSYDPYDFSDTEEEMPQVHTPKTADSQETKESQKVELSESRLKAFKVALL
DVFREAHAQSIGMNRLTESINRDSEEPFSSVEIQAALSKMQDDNQVMVSEGIIFLI

>NM_002388.4 Homo sapiens minichromosome maintenance complex component 3 (MCM3), transcript variant 1, mRNA
GAAGAAGAAGAAGCTTTTGATTGGACAGTCGCTAGTCCGACCTCAGAGAACTCAGCCAATCATAATGCTT
ATATGCTCCCAAGGTCCCCGCCTCTTCCTCGAGGTAACCTTTGGTGGCGGGAAGAGTTCGGAAGTTTTCG
CGCCGGGGTGGAGTCATCCTGGGAACCTCCACGCGACTTTGGTGGAGGTAGTTCTTTGGCAGCGGGCATG
GCGGGTACCGTGGTGCTGGACGATGTGGAGCTGCGGGAGGCTCAGAGAGATTACCTGGACTTCCTGGACG
ACGAGGAAGACCAGGGAATTTATCAGAGCAAAGTTCGGGAGCTGATCAGTGACAACCAATACCGGCTGAT
TGTCAATGTGAATGACCTGCGCAGGAAAAACGAGAAGAGGGCTAACCGGCTTCTGAACAATGCCTTTGAG
GAGCTGGTTGCCTTCCAGCGGGCCTTAAAGGATTTTGTGGCCTCCATTGATGCTACCTATGCCAAGCAGT
ATGAGGAGTTCTACGTAGGACTGGAAGGCAGCTTTGGCTCCAAGCACGTCTCCCCGCGGACTCTTACCTC
CTGCTTCCTCAGCTGTGTGGTCTGTGTGGAGGGCATTGTCACTAAATGTTCTCTAGTTCGTCCCAAAGTC
GTCCGCAGTGTCCACTACTGTCCTGCTACTAAGAAGACCATAGAGCGACGTTATTCTGATCTCACCACCC
TGGTGGCCTTTCCCTCCAGCTCTGTCTATCCTACCAAGGATGAGGAGAACAATCCCCTTGAGACAGAATA
TGGCCTTTCTGTCTACAAGGATCACCAGACCATCACCATCCAGGAGATGCCGGAGAAGGCCCCAGCCGGC
CAGCTCCCCCGCTCTGTGGACGTCATTCTGGATGATGACTTGGTGGATAAAGCGAAGCCTGGTGACCGGG
TTCAGGTGGTGGGAACCTACCGTTGCCTTCCTGGAAAGAAGGGAGGCTACACCTCTGGGACCTTCAGGAC
TGTCCTGATTGCCTGTAATGTTAAGCAGATGAGCAAGGATGCTCAGCCCTCTTTCTCTGCTGAGGATATA
GCCAAGATCAAGAAGTTCAGTAAAACCCGATCCAAGGATATCTTTGACCAGCTGGCCAAGTCATTGGCCC
CAAGTATCCATGGGCATGACTATGTCAAGAAAGCAATCCTCTGCTTGCTCTTGGGAGGGGTGGAACGAGA
CCTAGAAAATGGCAGCCACATCCGTGGGGACATCAATATTCTTCTAATAGGAGACCCATCCGTTGCCAAG
TCTCAGCTTCTGCGGTATGTGCTTTGCACTGCACCCCGAGCTATCCCCACCACTGGCCGGGGCTCCTCTG
GAGTGGGTCTGACGGCTGCTGTCACCACAGACCAGGAAACAGGAGAGCGCCGTCTGGAAGCAGGGGCCAT
GGTCCTGGCTGACCGAGGCGTGGTTTGCATTGATGAATTTGACAAAATGTCTGACATGGATCGCACAGCC
ATCCATGAAGTGATGGAGCAGGGTCGAGTGACCATTGCCAAGGCTGGCATCCATGCTCGGCTGAATGCCC
GCTGCAGTGTTTTGGCAGCTGCCAACCCTGTCTACGGCAGGTATGACCAGTATAAGACTCCAATGGAGAA
CATTGGGCTACAGGACTCACTGCTGTCACGATTTGACTTGCTCTTCATCATGCTGGATCAGATGGATCCT
GAGCAGGATCGGGAGATCTCAGACCATGTCCTTCGGATGCACCGTTACAGAGCACCTGGGGAGCAGGATG
GCGATGCTATGCCCTTGGGTAGTGCTGTGGATATCCTGGCCACAGATGATCCCAACTTTAGCCAGGAAGA
TCAGCAGGACACCCAGATTTATGAGAAGCATGACAACCTTCTACATGGGACCAAGAAGAAAAAGGAGAAG
ATGGTGAGTGCAGCATTCATGAAGAAGTACATCCATGTGGCCAAAATCATCAAGCCTGTCCTGACACAGG
AGTCGGCCACCTACATTGCAGAAGAGTATTCACGCCTGCGCAGCCAGGATAGCATGAGCTCAGACACCGC
CAGGACATCTCCAGTTACAGCCCGAACACTGGAAACTCTGATTCGACTGGCCACAGCCCATGCGAAGGCC
CGCATGAGCAAGACTGTGGACCTGCAGGATGCAGAGGAAGCTGTGGAGTTGGTCCAGTATGCTTACTTTA
AGAAGGTTCTGGAGAAGGAGAAGAAACGTAAGAAGCGAAGTGAGGATGAATCAGAGACAGAAGATGAAGA
GGAGAAAAGCCAAGAGGACCAGGAGCAGAAGAGGAAGAGAAGGAAGACTCGCCAGCCAGATGCCAAAGAT

GGGGATTCATACGACCCCTATGACTTCAGTGACACAGAGGAGGAAATGCCTCAAGTACACACTCCAAAGA
CGGCAGACTCACAGGAGACCAAGGAATCCCAGAAAGTGGAGTTGAGTGAATCCAGGTTGAAGGCATTCAA
GGTGGCCCTCTTGGATGTGTTCCGGGAAGCTCATGCGCAGTCAATCGGCATGAATCGCCTCACAGAATCC
ATCAACCGGGACAGCGAAGAGCCCTTCTCTTCAGTTGAGATCCAGGCTGCTCTGAGCAAGATGCAGGATG
ACAATCAGGTCATGGTGTCTGAGGGCATCATCTTCCTCATCTGAGGAGGCCTCGTCTCTGAACTTGGGTT
GTGCCGAGAGAGTTTGTTCTGTGTTTCCCACCCTCTCCCTGACCCAAGTCTTTGCCTCTACTCCCTTAAC
AGTGTTGAATTCAACTGAAGGCGAGGAATGTTGGTGATGAAGCTGAGTTCAGGACTCGGTGGACCCTTTG
GGAATGGGTCATGAAAGCTGCCATGGGGTGAGGAAAGAGGAGACAGTGGGAGAGGACAATGACTATTGCA
TCTTCATTGCAAAAGCACTGGCTCATCCGCCCTACTTCCCATCCCACACAAACCCAATTGTAAATAACAT
ATGACTTCTGAGTACTTTTGGGGGCACAACTGTTTTCTGTTTGCTGTTTTTTTGTTTTGTTTTTTTTCTC
CAGAGCACTTTGGTCTAGACTAGGCTTTGGGTGGTTCCAATTGGTGGAGAGAAGCTCTGAGGCACGTCAT
GCAGGTCAAGAAAGCTTTCTTTGCAGTAGCACCAGTTAAGGTGAATATGTATTGTATCACAAAACAAACC
CAATATCCAGATGAATATCCGAGATGTTGAATAAACTTAGCCATTTCGTACACATGGTAAAAAAAAAAAA
AAAAAAAAAAAAAA
>NP_002379.3 DNA replication licensing factor MCM3 isoform 1 [Homo sapiens]
MLPRSPPLPRGNLWWREEFGSFRAGVESSWEPPRDFGGGSSLAAGMAGTVVLDDVELREAQRDYLDFLDD
EEDQGIYQSKVRELISDNQYRLIVNVNDLRRKNEKRANRLLNNAFEELVAFQRALKDFVASIDATYAKQY
EEFYVGLEGSFGSKHVSPRTLTSCFLSCVVCVEGIVTKCSLVRPKVVRSVHYCPATKKTIERRYSDLTTL
VAFPSSSVYPTKDEENNPLETEYGLSVYKDHQTITIQEMPEKAPAGQLPRSVDVILDDDLVDKAKPGDRV
QVVGTYRCLPGKKGGYTSGTFRTVLIACNVKQMSKDAQPSFSAEDIAKIKKFSKTRSKDIFDQLAKSLAP
SIHGHDYVKKAILCLLLGGVERDLENGSHIRGDINILLIGDPSVAKSQLLRYVLCTAPRAIPTTGRGSSG
VGLTAAVTTDQETGERRLEAGAMVLADRGVVCIDEFDKMSDMDRTAIHEVMEQGRVTIAKAGIHARLNAR
CSVLAAANPVYGRYDQYKTPMENIGLQDSLLSRFDLLFIMLDQMDPEQDREISDHVLRMHRYRAPGEQDG
DAMPLGSAVDILATDDPNFSQEDQQDTQIYEKHDNLLHGTKKKKEKMVSAAFMKKYIHVAKIIKPVLTQE
SATYIAEEYSRLRSQDSMSSDTARTSPVTARTLETLIRLATAHAKARMSKTVDLQDAEEAVELVQYAYFK
KVLEKEKKRKKRSEDESETEDEEEKSQEDQEQKRKRRKTRQPDAKDGDSYDPYDFSDTEEEMPQVHTPKT
ADSQETKESQKVELSESRLKAFKVALLDVFREAHAQSIGMNRLTESINRDSEEPFSSVEIQAALSKMQDD
NQVMVSEGIIFLI
>NM_001270472.1 Homo sapiens minichromosome maintenance complex component 3 (MCM3), transcript variant 2, mRNA
GAAGAAGAAGAAGCTTTTGATTGGACAGTCGCTAGTCCGACCTCAGAGAACTCAGCCAATCATAATGCTT
ATATGCTCCCAAGGTCCCCGCCTCTTCCTCGAGGTAACCTTTGGTGGCGGGAAGAGTTCGGAAGTTTTCG
CGCCGGGGTGGAGTCATCCTGGGAACCTCCACGCGACTTTGGTGGAGGTAGTTCTTTGGCAGCGGGCATG
GCGGGTACCGTGGTGCTGGACGATGTGGAGCTGCGGGAGGCTCAGAGAGATTACCTGGACTTCCTGGACG
ACGAGGCTTCTGAACAATGCCTTTGAGGAGCTGGTTGCCTTCCAGCGGGCCTTAAAGGATTTTGTGGCCT
CCATTGATGCTACCTATGCCAAGCAGTATGAGGAGTTCTACGTAGGACTGGAAGGCAGCTTTGGCTCCAA
GCACGTCTCCCCGCGGACTCTTACCTCCTGCTTCCTCAGCTGTGTGGTCTGTGTGGAGGGCATTGTCACT
AAATGTTCTCTAGTTCGTCCCAAAGTCGTCCGCAGTGTCCACTACTGTCCTGCTACTAAGAAGACCATAG
AGCGACGTTATTCTGATCTCACCACCCTGGTGGCCTTTCCCTCCAGCTCTGTCTATCCTACCAAGGATGA
GGAGAACAATCCCCTTGAGACAGAATATGGCCTTTCTGTCTACAAGGATCACCAGACCATCACCATCCAG
GAGATGCCGGAGAAGGCCCCAGCCGGCCAGCTCCCCCGCTCTGTGGACGTCATTCTGGATGATGACTTGG
TGGATAAAGCGAAGCCTGGTGACCGGGTTCAGGTGGTGGGAACCTACCGTTGCCTTCCTGGAAAGAAGGG
AGGCTACACCTCTGGGACCTTCAGGACTGTCCTGATTGCCTGTAATGTTAAGCAGATGAGCAAGGATGCT
CAGCCCTCTTTCTCTGCTGAGGATATAGCCAAGATCAAGAAGTTCAGTAAAACCCGATCCAAGGATATCT
TTGACCAGCTGGCCAAGTCATTGGCCCCAAGTATCCATGGGCATGACTATGTCAAGAAAGCAATCCTCTG
CTTGCTCTTGGGAGGGGTGGAACGAGACCTAGAAAATGGCAGCCACATCCGTGGGGACATCAATATTCTT
CTAATAGGAGACCCATCCGTTGCCAAGTCTCAGCTTCTGCGGTATGTGCTTTGCACTGCACCCCGAGCTA
TCCCCACCACTGGCCGGGGCTCCTCTGGAGTGGGTCTGACGGCTGCTGTCACCACAGACCAGGAAACAGG
AGAGCGCCGTCTGGAAGCAGGGGCCATGGTCCTGGCTGACCGAGGCGTGGTTTGCATTGATGAATTTGAC
AAAATGTCTGACATGGATCGCACAGCCATCCATGAAGTGATGGAGCAGGGTCGAGTGACCATTGCCAAGG
CTGGCATCCATGCTCGGCTGAATGCCCGCTGCAGTGTTTTGGCAGCTGCCAACCCTGTCTACGGCAGGTA
TGACCAGTATAAGACTCCAATGGAGAACATTGGGCTACAGGACTCACTGCTGTCACGATTTGACTTGCTC
TTCATCATGCTGGATCAGATGGATCCTGAGCAGGATCGGGAGATCTCAGACCATGTCCTTCGGATGCACC
GTTACAGAGCACCTGGGGAGCAGGATGGCGATGCTATGCCCTTGGGTAGTGCTGTGGATATCCTGGCCAC
AGATGATCCCAACTTTAGCCAGGAAGATCAGCAGGACACCCAGATTTATGAGAAGCATGACAACCTTCTA
CATGGGACCAAGAAGAAAAAGGAGAAGATGGTGAGTGCAGCATTCATGAAGAAGTACATCCATGTGGCCA
AAATCATCAAGCCTGTCCTGACACAGGAGTCGGCCACCTACATTGCAGAAGAGTATTCACGCCTGCGCAG
CCAGGATAGCATGAGCTCAGACACCGCCAGGACATCTCCAGTTACAGCCCGAACACTGGAAACTCTGATT
CGACTGGCCACAGCCCATGCGAAGGCCCGCATGAGCAAGACTGTGGACCTGCAGGATGCAGAGGAAGCTG
TGGAGTTGGTCCAGTATGCTTACTTTAAGAAGGTTCTGGAGAAGGAGAAGAAACGTAAGAAGCGAAGTGA
GGATGAATCAGAGACAGAAGATGAAGAGGAGAAAAGCCAAGAGGACCAGGAGCAGAAGAGGAAGAGAAGG
AAGACTCGCCAGCCAGATGCCAAAGATGGGGATTCATACGACCCCTATGACTTCAGTGACACAGAGGAGG
AAATGCCTCAAGTACACACTCCAAAGACGGCAGACTCACAGGAGACCAAGGAATCCCAGAAAGTGGAGTT
GAGTGAATCCAGGTTGAAGGCATTCAAGGTGGCCCTCTTGGATGTGTTCCGGGAAGCTCATGCGCAGTCA
ATCGGCATGAATCGCCTCACAGAATCCATCAACCGGGACAGCGAAGAGCCCTTCTCTTCAGTTGAGATCC
AGGCTGCTCTGAGCAAGATGCAGGATGACAATCAGGTCATGGTGTCTGAGGGCATCATCTTCCTCATCTG
AGGAGGCCTCGTCTCTGAACTTGGGTTGTGCCGAGAGAGTTTGTTCTGTGTTTCCCACCCTCTCCCTGAC
CCAAGTCTTTGCCTCTACTCCCTTAACAGTGTTGAATTCAACTGAAGGCGAGGAATGTTGGTGATGAAGC
TGAGTTCAGGACTCGGTGGACCCTTTGGGAATGGGTCATGAAAGCTGCCATGGGGTGAGGAAAGAGGAGA
CAGTGGGAGAGGACAATGACTATTGCATCTTCATTGCAAAAGCACTGGCTCATCCGCCCTACTTCCCATC
CCACACAAACCCAATTGTAAATAACATATGACTTCTGAGTACTTTTGGGGGCACAACTGTTTTCTGTTTG
CTGTTTTTTTGTTTTGTTTTTTTTCTCCAGAGCACTTTGGTCTAGACTAGGCTTTGGGTGGTTCCAATTG
GTGGAGAGAAGCTCTGAGGCACGTCATGCAGGTCAAGAAAGCTTTCTTTGCAGTAGCACCAGTTAAGGTG
AATATGTATTGTATCACAAAACAAACCCAATATCCAGATGAATATCCGAGATGTTGAATAAACTTAGCCA
TTTCGTACACATGGTAAAAAAAAAAAAAAAAAAAAAAAAAA
>NP_001257401.1 DNA replication licensing factor MCM3 isoform 2 [Homo sapiens]
MLICSQGPRLFLEVTFGGGKSSEVFAPGWSHPGNLHATLVEVVLWQRAWRVPWCWTMWSCGRLREITWTS
WTTRLLNNAFEELVAFQRALKDFVASIDATYAKQYEEFYVGLEGSFGSKHVSPRTLTSCFLSCVVCVEGI
VTKCSLVRPKVVRSVHYCPATKKTIERRYSDLTTLVAFPSSSVYPTKDEENNPLETEYGLSVYKDHQTIT
IQEMPEKAPAGQLPRSVDVILDDDLVDKAKPGDRVQVVGTYRCLPGKKGGYTSGTFRTVLIACNVKQMSK
DAQPSFSAEDIAKIKKFSKTRSKDIFDQLAKSLAPSIHGHDYVKKAILCLLLGGVERDLENGSHIRGDIN

```
ILLIGDPSVAKSQLLRYVLCTAPRAIPTTGRGSSGVGLTAAVTTDQETGERRLEAGAMVLADRGVVCIDE
FDKMSDMDRTAIHEVMEQGRVTIAKAGIHARLNARCSVLAAANPVYGRYDQYKTPMENIGLQDSLLSRFD
LLFIMLDQMDPEQDREISDHVLRMHRYRAPGEQDGDAMPLGSAVDILATDDPNFSQEDQQDTQIYEKHDN
LLHGTKKKKEKMVSAAFMKKYIHVAKIIKPVLTQESATYIAEEYSRLRSQDSMSSDTARTSPVTARTLET
LIRLATAHAKARMSKTVDLQDAEEAVELVQYAYFKKVLEKEKKRKKRSEDESETEDEEEKSQEDQEQKRK
RRKTRQPDAKDGDSYDPYDFSDTEEEMPQVHTPKTADSQETKESQKVELSESRLKAFKVALLDVFREAHA
QSIGMNRLTESINRDSEEPFSSVEIQAALSKMQDDNQVMVSEGIIFLI

i1_HQ_samplec9816d|c23601/f2p0/1036      ------------------------------------------------------------      0
i1_HQ_samplec9816d|c22727/f2p0/1166      ------------------------------------------------------------      0
NM_002388.4                              GAAGAAGAAGAAGCTTTTGATTGGACAGTCGCTAGTCCGACCTCAGAGAACTCAGCCAAT      60
NM_001270472.1                           GAAGAAGAAGAAGCTTTTGATTGGACAGTCGCTAGTCCGACCTCAGAGAACTCAGCCAAT      60

i1_HQ_samplec9816d|c23601/f2p0/1036      ------------------------------------------------------------      0
i1_HQ_samplec9816d|c22727/f2p0/1166      ------------------------------------------------------------      0
NM_002388.4                              CATAATGCTTATATGCTCCCAAGGTCCCCGCCTCTTCCTCGAGGTAACCTTTGGTGGCGG      120
NM_001270472.1                           CATAATGCTTATATGCTCCCAAGGTCCCCGCCTCTTCCTCGAGGTAACCTTTGGTGGCGG      120

i1_HQ_samplec9816d|c23601/f2p0/1036      ------------------------------------------------------------      0
i1_HQ_samplec9816d|c22727/f2p0/1166      ------------------------------------------------------------      0
NM_002388.4                              GAAGAGTTCGGAAGTTTTCGCGCCGGGGTGGAGTCATCCTGGGAACCTCCACGCGACTTT      180
NM_001270472.1                           GAAGAGTTCGGAAGTTTTCGCGCCGGGGTGGAGTCATCCTGGGAACCTCCACGCGACTTT      180

i1_HQ_samplec9816d|c23601/f2p0/1036      ------------------------------------------------------------      0
i1_HQ_samplec9816d|c22727/f2p0/1166      ------------------------------------------------------------      0
NM_002388.4                              GGTGGAGGTAGTTCTTTGGCAGCGGGCATGGCGGGTACCGTGGTGCTGGACGATGTGGAG      240
NM_001270472.1                           GGTGGAGGTAGTTCTTTGGCAGCGGGCATGGCGGGTACCGTGGTGCTGGACGATGTGGAG      240

i1_HQ_samplec9816d|c23601/f2p0/1036      ------------------------------------------------------------      0
i1_HQ_samplec9816d|c22727/f2p0/1166      ------------------------------------------------------------      0
NM_002388.4                              CTGCGGGAGGCTCAGAGAGATTACCTGGACTTCCTGGACGACGAGGAAGACCAGGGAATT      300
NM_001270472.1                           CTGCGGGAGGCTCAGAGAGATTACCTGGACTTCCTGGACGACG-----------------      283

i1_HQ_samplec9816d|c23601/f2p0/1036      ------------------------------------------------------------      0
i1_HQ_samplec9816d|c22727/f2p0/1166      ------------------------------------------------------------      0
NM_002388.4                              TATCAGAGCAAAGTTCGGGAGCTGATCAGTGACAACCAATACCGGCTGATTGTCAATGTG      360
NM_001270472.1                           ------------------------------------------------------------      283

i1_HQ_samplec9816d|c23601/f2p0/1036      ------------------------------------------------------------      0
i1_HQ_samplec9816d|c22727/f2p0/1166      ------------------------------------------------------------      0
NM_002388.4                              AATGACCTGCGCAGGAAAAACGAGAAGAGGGCTAACCGGCTTCTGAACAATGCCTTTGAG      420
NM_001270472.1                           ------------------------------------AGGCTTCTGAACAATGCCTTTGAG      307

i1_HQ_samplec9816d|c23601/f2p0/1036      ------------------------------------------------------------      0
i1_HQ_samplec9816d|c22727/f2p0/1166      ------------------------------------------------------------      0
NM_002388.4                              GAGCTGGTTGCCTTCCAGCGGGCCTTAAAGGATTTTGTGGCCTCCATTGATGCTACCTAT      480
NM_001270472.1                           GAGCTGGTTGCCTTCCAGCGGGCCTTAAAGGATTTTGTGGCCTCCATTGATGCTACCTAT      367

i1_HQ_samplec9816d|c23601/f2p0/1036      ------------------------------------------------------------      0
i1_HQ_samplec9816d|c22727/f2p0/1166      ------------------------------------------------------------      0
NM_002388.4                              GCCAAGCAGTATGAGGAGTTCTACGTAGGACTGGAAGGCAGCTTTGGCTCCAAGCACGTC      540
NM_001270472.1                           GCCAAGCAGTATGAGGAGTTCTACGTAGGACTGGAAGGCAGCTTTGGCTCCAAGCACGTC      427

i1_HQ_samplec9816d|c23601/f2p0/1036      ------------------------------------------------------------      0
i1_HQ_samplec9816d|c22727/f2p0/1166      ------------------------------------------------------------      0
NM_002388.4                              TCCCCGCGGACTCTTACCTCCTGCTTCCTCAGCTGTGTGGTCTGTGTGGAGGGCATTGTC      600
NM_001270472.1                           TCCCCGCGGACTCTTACCTCCTGCTTCCTCAGCTGTGTGGTCTGTGTGGAGGGCATTGTC      487

i1_HQ_samplec9816d|c23601/f2p0/1036      ------------------------------------------------------------      0
i1_HQ_samplec9816d|c22727/f2p0/1166      ------------------------------------------------------------      0
NM_002388.4                              ACTAAATGTTCTCTAGTTCGTCCCAAAGTCGTCCGCAGTGTCCACTACTGTCCTGCTACT      660
NM_001270472.1                           ACTAAATGTTCTCTAGTTCGTCCCAAAGTCGTCCGCAGTGTCCACTACTGTCCTGCTACT      547

i1_HQ_samplec9816d|c23601/f2p0/1036      ------------------------------------------------------------      0
i1_HQ_samplec9816d|c22727/f2p0/1166      ------------------------------------------------------------      0
NM_002388.4                              AAGAAGACCATAGAGCGACGTTATTCTGATCTCACCACCCTGGTGGCCTTTCCCTCCAGC      720
NM_001270472.1                           AAGAAGACCATAGAGCGACGTTATTCTGATCTCACCACCCTGGTGGCCTTTCCCTCCAGC      607

i1_HQ_samplec9816d|c23601/f2p0/1036      ------------------------------------------------------------      0
i1_HQ_samplec9816d|c22727/f2p0/1166      ------------------------------------------------------------      0
NM_002388.4                              TCTGTCTATCCTACCAAGGATGAGGAGAACAATCCCCTTGAGACAGAATATGGCCTTTCT      780
NM_001270472.1                           TCTGTCTATCCTACCAAGGATGAGGAGAACAATCCCCTTGAGACAGAATATGGCCTTTCT      667

i1_HQ_samplec9816d|c23601/f2p0/1036      ------------------------------------------------------------      0
i1_HQ_samplec9816d|c22727/f2p0/1166      ------------------------------------------------------------      0
NM_002388.4                              GTCTACAAGGATCACCAGACCATCACCATCCAGGAGATGCCGGAGAAGGCCCCAGCCGGC      840
NM_001270472.1                           GTCTACAAGGATCACCAGACCATCACCATCCAGGAGATGCCGGAGAAGGCCCCAGCCGGC      727

i1_HQ_samplec9816d|c23601/f2p0/1036      ------------------------------------------------------------      0
```

```
i1_HQ_samplec9816d|c22727/f2p0/1166      ------------------------------------------------------------      0
NM_002388.4                              CAGCTCCCCCGCTCTGTGGACGTCATTCTGGATGATGACTTGGTGGATAAAGCGAAGCCT      900
NM_001270472.1                           CAGCTCCCCCGCTCTGTGGACGTCATTCTGGATGATGACTTGGTGGATAAAGCGAAGCCT      787

i1_HQ_samplec9816d|c23601/f2p0/1036      ------------------------------------------------------------      0
i1_HQ_samplec9816d|c22727/f2p0/1166      ------------------------------------------------------------      0
NM_002388.4                              GGTGACCGGGTTCAGGTGGTGGGAACCTACCGTTGCCTTCCTGGAAAGAAGGGAGGCTAC      960
NM_001270472.1                           GGTGACCGGGTTCAGGTGGTGGGAACCTACCGTTGCCTTCCTGGAAAGAAGGGAGGCTAC      847

i1_HQ_samplec9816d|c23601/f2p0/1036      ------------------------------------------------------------      0
i1_HQ_samplec9816d|c22727/f2p0/1166      ------------------------------------------------------------      0
NM_002388.4                              ACCTCTGGGACCTTCAGGACTGTCCTGATTGCCTGTAATGTTAAGCAGATGAGCAAGGAT      1020
NM_001270472.1                           ACCTCTGGGACCTTCAGGACTGTCCTGATTGCCTGTAATGTTAAGCAGATGAGCAAGGAT      907

i1 HQ samplec9816d|c23601/f2p0/1036      ------------------------------------------------------------      0
i1_HQ_samplec9816d|c22727/f2p0/1166      ------------------------------------------------------------      0
NM_002388.4                              GCTCAGCCCTCTTTCTCTGCTGAGGATATAGCCAAGATCAAGAAGTTCAGTAAAACCCGA      1080
NM_001270472.1                           GCTCAGCCCTCTTTCTCTGCTGAGGATATAGCCAAGATCAAGAAGTTCAGTAAAACCCGA      967

i1_HQ_samplec9816d|c23601/f2p0/1036      ------------------------------------------------------------      0
i1_HQ_samplec9816d|c22727/f2p0/1166      ------------------------------------------------------------      0
NM_002388.4                              TCCAAGGATATCTTTGACCAGCTGGCCAAGTCATTGGCCCCAAGTATCCATGGGCATGAC      1140
NM_001270472.1                           TCCAAGGATATCTTTGACCAGCTGGCCAAGTCATTGGCCCCAAGTATCCATGGGCATGAC      1027

i1_HQ_samplec9816d|c23601/f2p0/1036      ------------------------------------------------------------      0
i1_HQ_samplec9816d|c22727/f2p0/1166      ------------------------------------------------------------      0
NM_002388.4                              TATGTCAAGAAAGCAATCCTCTGCTTGCTCTTGGGAGGGGTGGAACGAGACCTAGAAAAT      1200
NM_001270472.1                           TATGTCAAGAAAGCAATCCTCTGCTTGCTCTTGGGAGGGGTGGAACGAGACCTAGAAAAT      1087

i1_HQ_samplec9816d|c23601/f2p0/1036      ------------------------------------------------------------      0
i1_HQ_samplec9816d|c22727/f2p0/1166      ------------------------------------------------------------      0
NM_002388.4                              GGCAGCCACATCCGTGGGGACATCAATATTCTTCTAATAGGAGACCCATCCGTTGCCAAG      1260
NM_001270472.1                           GGCAGCCACATCCGTGGGGACATCAATATTCTTCTAATAGGAGACCCATCCGTTGCCAAG      1147

i1_HQ_samplec9816d|c23601/f2p0/1036      ------------------------------------------------------------      0
i1 HQ samplec9816d|c22727/f2p0/1166      ------------------------------------------------------------      0
NM_002388.4                              TCTCAGCTTCTGCGGTATGTGCTTTGCACTGCACCCCGAGCTATCCCCACCACTGGCCGG      1320
NM_001270472.1                           TCTCAGCTTCTGCGGTATGTGCTTTGCACTGCACCCCGAGCTATCCCCACCACTGGCCGG      1207

i1_HQ_samplec9816d|c23601/f2p0/1036      ------------------------------------------------------------      0
i1_HQ_samplec9816d|c22727/f2p0/1166      ------------------------------------------------------------      0
NM_002388.4                              GGCTCCTCTGGAGTGGGTCTGACGGCTGCTGTCACCACAGACCAGGAAACAGGAGAGCGC      1380
NM_001270472.1                           GGCTCCTCTGGAGTGGGTCTGACGGCTGCTGTCACCACAGACCAGGAAACAGGAGAGCGC      1267

i1_HQ_samplec9816d|c23601/f2p0/1036      ------------------------------------------------------------      0
i1_HQ_samplec9816d|c22727/f2p0/1166      ------------------------------------------------------------      0
NM_002388.4                              CGTCTGGAAGCAGGGGCCATGGTCCTGGCTGACCGAGGCGTGGTTTGCATTGATGAATTT      1440
NM_001270472.1                           CGTCTGGAAGCAGGGGCCATGGTCCTGGCTGACCGAGGCGTGGTTTGCATTGATGAATTT      1327

i1_HQ_samplec9816d|c23601/f2p0/1036      ------------------------------------------------------------      0
i1_HQ_samplec9816d|c22727/f2p0/1166      ------------------------------------------------------------      0
NM_002388.4                              GACAAAATGTCTGACATGGATCGCACAGCCATCCATGAAGTGATGGAGCAGGGTCGAGTG      1500
NM_001270472.1                           GACAAAATGTCTGACATGGATCGCACAGCCATCCATGAAGTGATGGAGCAGGGTCGAGTG      1387

i1_HQ_samplec9816d|c23601/f2p0/1036      ------------------------------------------------------------      0
i1_HQ_samplec9816d|c22727/f2p0/1166      ------------------------------------------------------------      0
NM 002388.4                              ACCATTGCCAAGGCTGGCATCCATGCTCGGCTGAATGCCCGCTGCAGTGTTTTGGCAGCT      1560
NM_001270472.1                           ACCATTGCCAAGGCTGGCATCCATGCTCGGCTGAATGCCCGCTGCAGTGTTTTGGCAGCT      1447

i1_HQ_samplec9816d|c23601/f2p0/1036      ------------------------------------------------------------      0
i1_HQ_samplec9816d|c22727/f2p0/1166      ------------------------------------------------------------      0
NM_002388.4                              GCCAACCCTGTCTACGGCAGGTATGACCAGTATAAGACTCCAATGGAGAACATTGGGCTA      1620
NM_001270472.1                           GCCAACCCTGTCTACGGCAGGTATGACCAGTATAAGACTCCAATGGAGAACATTGGGCTA      1507

i1_HQ_samplec9816d|c23601/f2p0/1036      ------------------------------------------------------------      0
i1_HQ_samplec9816d|c22727/f2p0/1166      ------------------------------------------------------------      0
NM 002388.4                              CAGGACTCACTGCTGTCACGATTTGACTTGCTCTTCATCATGCTGGATCAGATGGATCCT      1680
NM_001270472.1                           CAGGACTCACTGCTGTCACGATTTGACTTGCTCTTCATCATGCTGGATCAGATGGATCCT      1567

i1_HQ_samplec9816d|c23601/f2p0/1036      ------------------------------------------------------------      0
i1_HQ_samplec9816d|c22727/f2p0/1166      ------------------------------------------------------------      0
NM_002388.4                              GAGCAGGATCGGGAGATCTCAGACCATGTCCTTCGGATGCACCGTTACAGAGCACCTGGG      1740
NM_001270472.1                           GAGCAGGATCGGGAGATCTCAGACCATGTCCTTCGGATGCACCGTTACAGAGCACCTGGG      1627

i1_HQ_samplec9816d|c23601/f2p0/1036      ------------------------------------------------------------      0
i1_HQ_samplec9816d|c22727/f2p0/1166      ------------------------------------------------------------      0
NM_002388.4                              GAGCAGGATGGCGATGCTATGCCCTTGGGTAGTGCTGTGGATATCCTGGCCACAGATGAT      1800
NM_001270472.1                           GAGCAGGATGGCGATGCTATGCCCTTGGGTAGTGCTGTGGATATCCTGGCCACAGATGAT      1687
```

```
i1_HQ_samplec9816d|c23601/f2p0/1036     ------------------------------------------------------------     0
i1_HQ_samplec9816d|c22727/f2p0/1166     ------------------------------------------------------------     0
NM_002388.4                             CCCAACTTTAGCCAGGAAGATCAGCAGGACACCCAGATTTATGAGAAGCATGACAACCTT     1860
NM_001270472.1                          CCCAACTTTAGCCAGGAAGATCAGCAGGACACCCAGATTTATGAGAAGCATGACAACCTT     1747

i1_HQ_samplec9816d|c23601/f2p0/1036     ------------------------------------------------------------     0
i1_HQ_samplec9816d|c22727/f2p0/1166     ------------------------------------------------------------     0
NM_002388.4                             CTACATGGGACCAAGAAGAAAAAGGAGAAGATGGTGAGTGCAGCATTCATGAAGAAGTAC     1920
NM_001270472.1                          CTACATGGGACCAAGAAGAAAAAGGAGAAGATGGTGAGTGCAGCATTCATGAAGAAGTAC     1807

i1_HQ_samplec9816d|c23601/f2p0/1036     ------------------------------------------------------------     0
i1_HQ_samplec9816d|c22727/f2p0/1166     ------------------------------------------------------------     0
NM_002388.4                             ATCCATGTGGCCAAAATCATCAAGCCTGTCCTGACACAGGAGTCGGCCACCTACATTGCA     1980
NM_001270472.1                          ATCCATGTGGCCAAAATCATCAAGCCTGTCCTGACACAGGAGTCGGCCACCTACATTGCA     1867

i1_HQ_samplec9816d|c23601/f2p0/1036     ------------------------------------------------------------     0
i1_HQ_samplec9816d|c22727/f2p0/1166     ---------------------------------------------------aggacatct     9
NM_002388.4                             GAAGAGTATTCACGCCTGCGCAGCCAGGATAGCATGAGCTCAGACACCGCCAGGACATCT     2040
NM_001270472.1                          GAAGAGTATTCACGCCTGCGCAGCCAGGATAGCATGAGCTCAGACACCGCCAGGACATCT     1927

i1_HQ_samplec9816d|c23601/f2p0/1036     ------------------------------------------------------------     0
i1_HQ_samplec9816d|c22727/f2p0/1166     ccagttacagcccgaacactggaaactctgattcgactggccacagcccatgcgaaggcC     69
NM_002388.4                             CCAGTTACAGCCCGAACACTGGAAACTCTGATTCGACTGGCCACAGCCCATGCGAAGGCC     2100
NM_001270472.1                          CCAGTTACAGCCCGAACACTGGAAACTCTGATTCGACTGGCCACAGCCCATGCGAAGGCC     1987

i1_HQ_samplec9816d|c23601/f2p0/1036     --------------------------ggattca---------------tACGACCCCTAT     19
i1_HQ_samplec9816d|c22727/f2p0/1166     CGCATGAGCAAGACTGTGGACCTGCAGGATGCAGAGGAAGCTGTGGAGTTGGTCCAGTAT     129
NM_002388.4                             CGCATGAGCAAGACTGTGGACCTGCAGGATGCAGAGGAAGCTGTGGAGTTGGTCCAGTAT     2160
NM_001270472.1                          CGCATGAGCAAGACTGTGGACCTGCAGGATGCAGAGGAAGCTGTGGAGTTGGTCCAGTAT     2047
                                                                  **** **               *  * **  ***

i1_HQ_samplec9816d|c23601/f2p0/1036     GACTTCAGTGACACA---------GAGGAGGAAATGCCTCAAGTAC---------ACACT     61
i1_HQ_samplec9816d|c22727/f2p0/1166     GCTTACTTTAAGAAGGTTCTGGAGAAGGAGAAGAAACGTAAGAAGCGAAGTGAGGATGAA     189
NM_002388.4                             GCTTACTTTAAGAAGGTTCTGGAGAAGGAGAAGAAACGTAAGAAGCGAAGTGAGGATGAA     2220
NM_001270472.1                          GCTTACTTTAAGAAGGTTCTGGAGAAGGAGAAGAAACGTAAGAAGCGAAGTGAGGATGAA     2107
                                        *  * *  * * *            ***** * *  * * *    *         *

i1_HQ_samplec9816d|c23601/f2p0/1036     CCAAAGACGGCAGACTCACAGGAGACCAAGGAATCCCAGAAAGTGGAGTTGAGTGAATCC     121
i1_HQ_samplec9816d|c22727/f2p0/1166     TCAGAGACAGAAGATGAAGAGGAGAAAAGCCAAGAGGACCAGGAGCAGAAGAGGAAGAGA     249
NM_002388.4                             TCAGAGACAGAAGATGAAGAGGAGAAAAGCCAAGAGGACCAGGAGCAGAAGAGGAAGAGA     2280
NM_001270472.1                          TCAGAGACAGAAGATGAAGAGGAGAAAAGCCAAGAGGACCAGGAGCAGAAGAGGAAGAGA     2167
                                         ** **** * ***   * ******  *   **    *  * * * **  ***  *

i1_HQ_samplec9816d|c23601/f2p0/1036     AGAGACGGGATCTCACCATGTTGCCCAGGCTGGTCTTGGACTCCTGGCCTCAAGCAGTCC     181
i1_HQ_samplec9816d|c22727/f2p0/1166     -AGGAAGACTCGCCAGCCAGATGCCAAAGATGGGGATTCATACGACCCCTAT-GACTTCA     307
NM_002388.4                             -AGGAAGACTCGCCAGCCAGATGCCAAAGATGGGGATTCATACGACCCCTAT-GACTTCA     2338
NM_001270472.1                          -AGGAAGACTCGCCAGCCAGATGCCAAAGATGGGGATTCATACGACCCCTAT-GACTTCA     2225
                                           ** *      ** *  * **** * * ***   *  *  *    ***   *   **

i1_HQ_samplec9816d|c23601/f2p0/1036     TTCTACCTGAGCCTCCCA----AAAGGCTGGGATTACAGGCATAAGCCACTGTGCTTGTT     237
i1_HQ_samplec9816d|c22727/f2p0/1166     GTGACACAGAGGAGGAAATGCCTCAAGTACACACTCCAAAGACGGCAGACTCACAGGAGA     367
NM_002388.4                             GTGACACAGAGGAGGAAATGCCTCAAGTACACACTCCAAAGACGGCAGACTCACAGGAGA     2398
NM_001270472.1                          GTGACACAGAGGAGGAAATGCCTCAAGTACACACTCCAAAGACGGCAGACTCACAGGAGA     2285
                                         *    * ***      *      * *     * * **   *      ***

i1_HQ_samplec9816d|c23601/f2p0/1036     TGATCTCATTCTTTTTAATGACTGCAGGGTTTCCATGGTTGAAGGCATTCAAGGT-GGCC     296
i1_HQ_samplec9816d|c22727/f2p0/1166     CCAAGGAATCCCAGAAAGTGGAGTTGAGTGAATCCAGGTTGAAGGCATTCAAGGTGGCCC     427
NM_002388.4                             CCAAGGAATCCCAGAAAGTGGAGTTGAGTGAATCCAGGTTGAAGGCATTCAAGGTGGCCC     2458
NM_001270472.1                          CCAAGGAATCCCAGAAAGTGGAGTTGAGTGAATCCAGGTTGAAGGCATTCAAGGTGGCCC     2345
                                          *    ** *     * **       *     *  ******************* * **

i1_HQ_samplec9816d|c23601/f2p0/1036     CTCTTGGTGTGTTCCGGGAAGCTCATGCGCAGTCAATCGGCATGAATCGCCTCACAGAAT     356
i1_HQ_samplec9816d|c22727/f2p0/1166     TCTTGGATGTGTTCCGGGAAGCTCATGCGCAGTCAATCGGCATGAATCGCCTCACAGAAT     487
NM_002388.4                             TCTTGGATGTGTTCCGGGAAGCTCATGCGCAGTCAATCGGCATGAATCGCCTCACAGAAT     2518
NM_001270472.1                          TCTTGGATGTGTTCCGGGAAGCTCATGCGCAGTCAATCGGCATGAATCGCCTCACAGAAT     2405
                                           * * *****************************************************

i1_HQ_samplec9816d|c23601/f2p0/1036     CCATCAACCGGGACAGCGAAGAGCCCTTCTCTTCAGTTGAGATCCAGGCTGCTCTGAGCA     416
i1_HQ_samplec9816d|c22727/f2p0/1166     CCATCAACCGGGACAGCGAAGAGCCCTTCTCTTCAGTTGAGATCCAGGCTGCTCTGAGCA     547
NM_002388.4                             CCATCAACCGGGACAGCGAAGAGCCCTTCTCTTCAGTTGAGATCCAGGCTGCTCTGAGCA     2578
NM_001270472.1                          CCATCAACCGGGACAGCGAAGAGCCCTTCTCTTCAGTTGAGATCCAGGCTGCTCTGAGCA     2465
                                        ************************************************************

i1_HQ_samplec9816d|c23601/f2p0/1036     AGATGCAGGATGACAATCAGGTCATGGTGTCTGAGGGCATCATCTTCCTCATCTGAGGAG     476
i1_HQ_samplec9816d|c22727/f2p0/1166     AGATGCAGGATGACAATCAGGTCATGGTGTCTGAGGGCATCATCTTCCTCATCTGAGGAG     607
NM_002388.4                             AGATGCAGGATGACAATCAGGTCATGGTGTCTGAGGGCATCATCTTCCTCATCTGAGGAG     2638
NM_001270472.1                          AGATGCAGGATGACAATCAGGTCATGGTGTCTGAGGGCATCATCTTCCTCATCTGAGGAG     2525
                                        ************************************************************

i1_HQ_samplec9816d|c23601/f2p0/1036     GCCTCGTCTCTGAACTTGGGTTGTGCCGAGAGAGTTTGTTCTGTGTTTCCCACCCTCTCC     536
i1_HQ_samplec9816d|c22727/f2p0/1166     GCCTCGTCTCTGAACTTGGGTTGTGCCGAGAGAGTTTGTTCTGTGTTTCCCACCCTCTCC     667
NM_002388.4                             GCCTCGTCTCTGAACTTGGGTTGTGCCGAGAGAGTTTGTTCTGTGTTTCCCACCCTCTCC     2698
NM_001270472.1                          GCCTCGTCTCTGAACTTGGGTTGTGCCGAGAGAGTTTGTTCTGTGTTTCCCACCCTCTCC     2585
                                        ************************************************************

i1_HQ_samplec9816d|c23601/f2p0/1036     CTGACCCAAGTCTTTGCCTCTACTCCCTTAACAGTGTTGAATTCAACTGAAGGCGAGGAA     596
i1_HQ_samplec9816d|c22727/f2p0/1166     CTGACCCAAGTCTTTGCCTCTACTCCCTTAACAGTGTTGAATTCAACTGAAGGCGAGGAA     727
NM_002388.4                             CTGACCCAAGTCTTTGCCTCTACTCCCTTAACAGTGTTGAATTCAACTGAAGGCGAGGAA     2758
```

```
NM_001270472.1                        CTGACCCAAGTCTTTGCCTCTACTCCCTTAACAGTGTTGAATTCAACTGAAGGCGAGGAA    2645
                                      ************************************************************

i1_HQ_samplec9816d|c23601/f2p0/1036   TGTTGGTGATGAAGCTGAGTTCAGGACTCGGTGGACCCTTTGGGAATGGGTCATGAAAGC    656
i1_HQ_samplec9816d|c22727/f2p0/1166   TGTTGGTGATGAAGCTGAGTTCAGGACTCGGTGGACCCTTTGGGAATGGGTCATGAAAGC    787
NM_002388.4                           TGTTGGTGATGAAGCTGAGTTCAGGACTCGGTGGACCCTTTGGGAATGGGTCATGAAAGC    2818
NM_001270472.1                        TGTTGGTGATGAAGCTGAGTTCAGGACTCGGTGGACCCTTTGGGAATGGGTCATGAAAGC    2705
                                      ************************************************************

i1_HQ_samplec9816d|c23601/f2p0/1036   TGCCATGGGGTGAGGAAAGAGGAGACAGTGGGAGAGGACAATGACTATTGCATCTTCATT    716
i1_HQ_samplec9816d|c22727/f2p0/1166   TGCCATGGGGTGAGGAAAGAGGAGACAGTGGGAGAGGACAATGACTATTGCATCTTCATT    847
NM_002388.4                           TGCCATGGGGTGAGGAAAGAGGAGACAGTGGGAGAGGACAATGACTATTGCATCTTCATT    2878
NM_001270472.1                        TGCCATGGGGTGAGGAAAGAGGAGACAGTGGGAGAGGACAATGACTATTGCATCTTCATT    2765
                                      ************************************************************

i1_HQ_samplec9816d|c23601/f2p0/1036   GCAAAAGCACTGGCTCATCCGCCCTACTTCCCATCCCACACAAACCCAATTGTAAATAAC    776
i1_HQ_samplec9816d|c22727/f2p0/1166   GCAAAAGCACTGGCTCATCCGCCCTACTTCCCATCCCACACAAACCCAATTGTAAATAAC    907
NM_002388.4                           GCAAAAGCACTGGCTCATCCGCCCTACTTCCCATCCCACACAAACCCAATTGTAAATAAC    2938
NM_001270472.1                        GCAAAAGCACTGGCTCATCCGCCCTACTTCCCATCCCACACAAACCCAATTGTAAATAAC    2825
                                      ************************************************************

i1_HQ_samplec9816d|c23601/f2p0/1036   ATATGACTTCTGAGTACTTTTGGGGGCACAACTGTTTTCTGTTTGCTGTTTTTTTGTTTT    836
i1_HQ_samplec9816d|c22727/f2p0/1166   ATATGACTTCTGAGTACTTTTGGGGGCACAACTGTTTTCTGTTTGCTGTTTTTTTGTTTT    967
NM_002388.4                           ATATGACTTCTGAGTACTTTTGGGGGCACAACTGTTTTCTGTTTGCTGTTTTTTTGTTTT    2998
NM_001270472.1                        ATATGACTTCTGAGTACTTTTGGGGGCACAACTGTTTTCTGTTTGCTGTTTTTTTGTTTT    2885
                                      ************************************************************

i1_HQ_samplec9816d|c23601/f2p0/1036   GTTTTTTTTCTCCAGAGCACTTTGGTCTAGACTAGGCTTTGGGTGGTTCCAATTGGTGGA    896
i1_HQ_samplec9816d|c22727/f2p0/1166   GTTTTTTTTCTCCAGAGCACTTTGGTCTAGACTAGGCTTTGGGTGGTTCCAATTGGTGGA    1027
NM_002388.4                           GTTTTTTTTCTCCAGAGCACTTTGGTCTAGACTAGGCTTTGGGTGGTTCCAATTGGTGGA    3058
NM_001270472.1                        GTTTTTTTTCTCCAGAGCACTTTGGTCTAGACTAGGCTTTGGGTGGTTCCAATTGGTGGA    2945
                                      ************************************************************

i1_HQ_samplec9816d|c23601/f2p0/1036   GAGAAGCTCTGAGGCACGTCATGCAGGTCAAGAAAGCTTTCTTTGCAGTAGCACCAGTTA    956
i1_HQ_samplec9816d|c22727/f2p0/1166   GAGAAGCTCTGAGGCACGTCATGCAGGTCAAGAAAGCTTTCTTTGCAGTAGCACCAGTTA    1087
NM_002388.4                           GAGAAGCTCTGAGGCACGTCATGCAGGTCAAGAAAGCTTTCTTTGCAGTAGCACCAGTTA    3118
NM_001270472.1                        GAGAAGCTCTGAGGCACGTCATGCAGGTCAAGAAAGCTTTCTTTGCAGTAGCACCAGTTA    3005
                                      ************************************************************

i1_HQ_samplec9816d|c23601/f2p0/1036   AGGTGAATATGTATTGTATCACAAAACAAACCCAATATCCAGATGAATatccgagatgtt    1016
i1_HQ_samplec9816d|c22727/f2p0/1166   AGGTGAATATGTATTGTATCACAAAACAAACCCAATATCCAGATGAATATCcgagatgtt    1147
NM_002388.4                           AGGTGAATATGTATTGTATCACAAAACAAACCCAATATCCAGATGAATATCCGAGATGTT    3178
NM_001270472.1                        AGGTGAATATGTATTGTATCACAAAACAAACCCAATATCCAGATGAATATCCGAGATGTT    3065
                                      ************************************************************

i1_HQ_samplec9816d|c23601/f2p0/1036   gaataaacttagccatttcg------------------------------------        1036
i1_HQ_samplec9816d|c22727/f2p0/1166   gataaacttagccatttcg-------------------------------------        1166
NM_002388.4                           GAATAAACTTAGCCATTTCGTACACATGGTAAAAAAAAAAAAAAAAAAAAAAAAAA        3234
NM_001270472.1                        GAATAAACTTAGCCATTTCGTACACATGGTAAAAAAAAAAAAAAAAAAAAAAAAAA        3121
                                      **  **  *   *  **
```

```
VIRT161936          ------------------------------------------------------------  0
NP_001257401.1      MLICSQGP-----RLFLEVTFGGGKSSEVFAPGWSHPGNLHA----------TL----VE  41
VIRT162782          ------------------------------------------------------------  0
NP_002379.3         ML--PRSPPLPRGNLWWREEFGSFR--AGVESSWEPPRDFGGGSSLAAGMAGTVVLDDVE  56

VIRT161936          ------------------------------------------------------------  0
NP_001257401.1      VV-----------------LWQRAWRVP---WCWT-MWSCGRLREITWTSWTTRLLNNAF  80
VIRT162782          ------------------------------------------------------------  0
NP_002379.3         LREAQRDYLDFLDDEEDQGIYQSKVRELISDNQYRLIVNVNDLR-RKNEKRANRLLNNAF  115

VIRT161936          ------------------------------------------------------------  0
NP_001257401.1      EELVAFQRALKDFVASIDATYAKQYEEFYVGLEGSFGSKHVSPRTLTSCFLSCVVCVEGI  140
VIRT162782          ------------------------------------------------------------  0
NP_002379.3         EELVAFQRALKDFVASIDATYAKQYEEFYVGLEGSFGSKHVSPRTLTSCFLSCVVCVEGI  175

VIRT161936          ------------------------------------------------------------  0
NP_001257401.1      VTKCSLVRPKVVRSVHYCPATKKTIERRYSDLTTLVAFPSSSVYPTKDEENNPLETEYGL  200
VIRT162782          ------------------------------------------------------------  0
NP_002379.3         VTKCSLVRPKVVRSVHYCPATKKTIERRYSDLTTLVAFPSSSVYPTKDEENNPLETEYGL  235

VIRT161936          ------------------------------------------------------------  0
NP_001257401.1      SVYKDHQTITIQEMPEKAPAGQLPRSVDVILDDDLVDKAKPGDRVQVVGTYRCLPGKKGG  260
VIRT162782          ------------------------------------------------------------  0
NP_002379.3         SVYKDHQTITIQEMPEKAPAGQLPRSVDVILDDDLVDKAKPGDRVQVVGTYRCLPGKKGG  295

VIRT161936          ------------------------------------------------------------  0
NP_001257401.1      YTSGTFRTVLIACNVKQMSKDAQPSFSAEDIAKIKKFSKTRSKDIFDQLAKSLAPSIHGH  320
VIRT162782          ------------------------------------------------------------  0
NP_002379.3         YTSGTFRTVLIACNVKQMSKDAQPSFSAEDIAKIKKFSKTRSKDIFDQLAKSLAPSIHGH  355

VIRT161936          ------------------------------------------------------------  0
NP_001257401.1      DYVKKAILCLLLGGVERDLENGSHIRGDINILLIGDPSVAKSQLLRYVLCTAPRAIPTTG  380
VIRT162782          ------------------------------------------------------------  0
NP_002379.3         DYVKKAILCLLLGGVERDLENGSHIRGDINILLIGDPSVAKSQLLRYVLCTAPRAIPTTG  415
```

```
VIRT161936              ------------------------------------------------------------ 0
NP_001257401.1          RGSSGVGLTAAVTTDQETGERRLEAGAMVLADRGVVCIDEFDKMSDMDRTAIHEVMEQGR 440
VIRT162782              ------------------------------------------------------------ 0
NP_002379.3             RGSSGVGLTAAVTTDQETGERRLEAGAMVLADRGVVCIDEFDKMSDMDRTAIHEVMEQGR 475

VIRT161936              ------------------------------------------------------------ 0
NP_001257401.1          VTIAKAGIHARLNARCSVLAAANPVYGRYDQYKTPMENIGLQDSLLSRFDLLFIMLDQMD 500
VIRT162782              ------------------------------------------------------------ 0
NP_002379.3             VTIAKAGIHARLNARCSVLAAANPVYGRYDQYKTPMENIGLQDSLLSRFDLLFIMLDQMD 535

VIRT161936              ------------------------------------------------------------ 0
NP_001257401.1          PEQDREISDHVLRMHRYRAPGEQDGDAMPLGSAVDILATDDPNFSQEDQQDTQIYEKHDN 560
VIRT162782              ------------------------------------------------------------ 0
NP_002379.3             PEQDREISDHVLRMHRYRAPGEQDGDAMPLGSAVDILATDDPNFSQEDQQDTQIYEKHDN 595

VIRT161936              ------------------------------------------------------------ 0
NP_001257401.1          LLHGTKKKKEKMVSAAFMKKYIHVAKIIKPVLTQESATYIAEEYSRLRSQDSMSSDTART 620
VIRT162782              ------------------------------------------------------------ 0
NP_002379.3             LLHGTKKKKEKMVSAAFMKKYIHVAKIIKPVLTQESATYIAEEYSRLRSQDSMSSDTART 655

VIRT161936              ------------------------------------------------------------ 0
NP_001257401.1          SPVTARTLETLIRLATAHAKARMSKTVDLQDAEEAVELVQYAYFKKVLEKEKKRKKRSED 680
VIRT162782              ----------------------MSKTVDLQDAEEAVELVQYAYFKKVLEKEKKRKKRSED 38
NP_002379.3             SPVTARTLETLIRLATAHAKARMSKTVDLQDAEEAVELVQYAYFKKVLEKEKKRKKRSED 715

VIRT161936              ----------------------------------------------MPQVHTPKTADSQE 14
NP_001257401.1          ESETEDEEEKSQEDQEQKRKRRKTRQPDAKDGDSYDPYDFSDTEEEMPQVHTPKTADSQE 740
VIRT162782              ESETEDEEEKSQEDQEQKRKRRKTRQPDAKDGDSYDPYDFSDTEEEMPQVHTPKTADSQE 98
NP_002379.3             ESETEDEEEKSQEDQEQKRKRRKTRQPDAKDGDSYDPYDFSDTEEEMPQVHTPKTADSQE 775
                                                                      **************

VIRT161936              TKESQKVELSESRDGISPCCPGWSWTPGLKQSFYLSLPKGWDYRHKPLCLFDLILFNDCR 74
NP_001257401.1          TKESQKVELSESRLK--------------------------------------------- 755
VIRT162782              TKESQKVELSESRLK--------------------------------------------- 113
NP_002379.3             TKESQKVELSESRLK--------------------------------------------- 790
                        *************

VIRT161936              VSMVEGIQGGPLGVFREAHAQSIGMNRLTESINRDSEEPFSSVEIQAALSKMQDDNQVMV 134
NP_001257401.1          -----AFKVALLDVFREAHAQSIGMNRLTESINRDSEEPFSSVEIQAALSKMQDDNQVMV 810
VIRT162782              -----AFKVALLDVFREAHAQSIGMNRLTESINRDSEEPFSSVEIQAALSKMQDDNQVMV 168
NP_002379.3             -----AFKVALLDVFREAHAQSIGMNRLTESINRDSEEPFSSVEIQAALSKMQDDNQVMV 845
                             .:: . *.***********************************************

VIRT161936              SEGIIFLI        142
NP_001257401.1          SEGIIFLI        818
VIRT162782              SEGIIFLI        176
NP_002379.3             SEGIIFLI        853
                        ********
```

## NINJ1

| Sequence | Header | AA Seq |
|---|---|---|
| **Present** | i1_HQ_samplec9816d\|c43678/f2p0/1209 | |
| | | |
| **Absent** | i1_HQ_samplec9816d\|c77387/f3p0/1237 | |
| **NCBI** | NM_004148 | |
| | | |

>i1_HQ_samplec9816d|c43678/f2p0/1209
isoform=c43678;full_length_coverage=2;non_full_length_coverage=0;isoform_length=1209

GGCACACCCGGCTCCCCGGACGCCTCGCCCGCAGGTTGAAGGTTCCTGGGGCCACATCTGGGCCGAGAGGCATGGTCC
TTGGAGCCGGCCCGCTGGGGCTGGAGGCACGGGCCCATCAACGTGAACCATTACGCCAGCAAGAAGAGCGCAGCCGA
GAGCATGCTGGACATCGCGCTGCTGATGGCCAACGCGTCCCAGCTGAAGGCCGTCGTGGAACAGGGCCCCAGCTTCGC
CTTCTATGTGCCCCTGGTGGTCCTCATCTCCATCTCCCTTGTGCTGCAGATCGGCGTGGGGGTGCTGCTCATCTTCCTTGT
CAAGTACGACCTTAACAACCCGGACAAGCACGCCAAGCTGGACTTCCTCAACAACCTGGCCACGGGCCTGGTGTTCATC
ATCGTGGTAGTCAACATCTTCATCACGGCCTTCGGGGTCCAGAAGCCCTTGATGGACATGGCACCCCAGCAGTAGGACA

CCCAGGACCCTGGATGCTGCCTGCCCTGCAACTCAGCTGCCCGACCCCAGGAGTCGCCATACCTGTGAGGTGTCCACCT
CCCTGCACATGGCACTACCCAGACTGCCAGAGCCCAGGCTGGCCTCATCTGCACCATGTCCCCGGACCAGCCCTTGCTCT
GACTGCGGCCAAGCACCACGCAGGAGGCCACTCTTGTCTCTCAGCAGCTGTTCCCAGGAGGCAGCTCCCTCCTGGCACA
TGGGGGCTGGCCACAATAGCCCAGAGGGTCAGAACTGGACAGCTGCAGAGACCTGTGCCCAGAGAAGGGTCTCGACC
CACTCAAGGACACACAGCAGGTCCGTGGATGGGCTGGATGAGTGACCAGGGCCAGCCTCTGTCTCAGGACATTCCAGA
AGGACAAGGAGATGTCTCTCCCTCTCCCAAAGCACCAGCGTCCCTGCCTCCCGTGGGCCCTGTCCGGGTTGCCCTGGTG
ACCCCAGCCTCTGTCCACTTCCTAACCCAGGGACCCTGCACAGCCAGAACTGCCTTTGGCCCTACGGATGGCCACTGGCT
CTGGTCTAAAGTGCCTGGGCTTGGTGGCCATCAAGAGGGAGCCAGTCAGGCCTGTGAGGGCCGTAGACCTTGTATATA
CCCTGCACCAGCAGTGACCGGGCAGAGCCCAACCCCCTCCACGGGGGTCCCAGCACCCACTTTTCTAATCATGAATgaac
aatataaagcccacgctctttgtcagg

>VIRT68216

MLPALQLSCPTPGVAIPVRCPPPCTWHYPDCQSPGWPHLHHVPGPALALTAAKHHAGGHS
CLSAAVPRRQLPPGTWGLATIAQRVRTGQLQRPVPREGSRPTQGHTAGPWMGWMSDQGQP
LSQDIPEGQGDVSPSPKAPASLPPVGPVRVALVTPASVHFLTQGPCTARTAFGPTDGHWL
WSKVPGLGGHQEGASQACEGRRPCIYPAPAVTGQSPTPSTGVPAPTFLIMNEQYKAHALC
Q

>i1_HQ_samplec9816d|c77387/f3p0/1237
isoform=c77387;full_length_coverage=3;non_full_length_coverage=0;isoform_length=1237

cgctcaggcccggatcctggcggcctgggcggccgcaccatggactcgggaaccgaggagtacgaGCTCAACGGCGGCCTGCCTCCGGGCAC
ACCCGGCTCCCCGGACGCCTCGCCGGCCCGCTGGGGCTGGAGGCACGGGCCCATCAACGTGAACCATTACGCCAGCAA
GAAGAGCGCAGCCGAGAGCATGCTGGACATCGCGCTGCTGATGGCCAACGCGTCCCAGCTGAAGGCCGTCGTGGAAC
AGGGCCCCAGCTTCGCCTTCTATGTGCCCCTGGTGGTCCTCATCTCCATCTCCCTTGTGCTGCAGATCGGCGTGGGGGTG
CTGCTCATCTTCCTTGTCAAGTACGACCTTAACAACCCGGACAAGCACGCCAAGCTGGACTTCCTCAACAACCTGGCCAC
GGGCCTGGTGTTCATCATCGTGGTAGTCAACATCTTCATCACGGCCTTCGGGGTCCAGAAGCCCTTGATGGACATGGCA
CCCCAGCAGTAGGACACCCAGGACCCTGGATGCTGCCTGCCCTGCAACTCAGCTGCCCGACCCCAGGAGTCGCCATACC
TGTGAGGTGTCCACCTCCCTGCACATGGCACTACCCAGACTGCCAGAGCCCAGGCTGGCCTCATCTGCACCATGTCCCC
GGACCAGCCCTTGCTCTGACTGCGGCCAAGCACCACGCAGGAGGCCACTCTTGTCTCTCAGCAGCTGTTCCCAGGAGGC
AGCTCCCTCCTGGCACATGGGGGCTGGCCACAATAGCCCAGAGGGTCAGAACTGGACAGCTGCAGAGACCTGTGCCCA
GAGAAGGGTCTCGACCCACTCAAGGACACACAGCAGGTCCGTGGATGGGCTGGATGAGTGACCAGGGCCAGCCTCTG
TCTCAGGACATTCCAGAAGGACAAGGAGATGTCTCTCCCTCTCCCAAAGCACCAGCGTCCCTGCCTCCCGTGGGCCCTG
TCCGGGTTGCCCTGGTGACCCCAGCCTCTGTCCACTTCCTAACCCAGGGACCCTGCACAGCCAGAACTGCCTTTGGCCCT
ACGGATGGCCACTGGCTCTGGTCTAAAGTGCCTGGGCTTGGTGGCCATCAAGAGGGAGCCAGTCAGGCCTGTGAGGG
CCGTAGACCTTGTATATACCCTGCACCAGCAGTGACCGGGCAGAGCCCAACCCCCTCCACGGGGGTCCCAGCACCCACT
TTTCTAATCATGAATGAACAATaaagcccacgctctttgtcagg

>VIRT69015

MLPALQLSCPTPGVAIPVRCPPPCTWHYPDCQSPGWPHLHHVPGPALALTAAKHHAGGHS
CLSAAVPRRQLPPGTWGLATIAQRVRTGQLQRPVPREGSRPTQGHTAGPWMGWMSDQGQP
LSQDIPEGQGDVSPSPKAPASLPPVGPVRVALVTPASVHFLTQGPCTARTAFGPTDGHWL
WSKVPGLGGHQEGASQACEGRRPCIYPAPAVTGQSPTPSTGVPAPTFLIMNEQ

>NM_004148.3 Homo sapiens ninjurin 1 (NINJ1), mRNA
CGCAGCTGGAGCCTGCGGCTGAGGCTCGGGCGCGCTCAGGCCCGGATCCTGGCGGCCTGGGCGGCCGCAC

```
CATGGACTCGGGAACCGAGGAGTACGAGCTCAACGGCGGCCTGCCTCCGGGCACACCCGGCTCCCCGGAC
GCCTCGCCGGCCCGCTGGGGCTGGAGGCACGGGCCCATCAACGTGAACCATTACGCCAGCAAGAAGAGCG
CAGCCGAGAGCATGCTGGACATCGCGCTGCTGATGGCCAACGCGTCCCAGCTGAAGGCCGTCGTGGAACA
GGGCCCCAGCTTCGCCTTCTATGTGCCCCTGGTGGTCCTCATCTCCATCTCCCTTGTGCTGCAGATCGGC
GTGGGGGTGCTGCTCATCTTCCTTGTCAAGTACGACCTTAACAACCCGGCCAAGCACGCCAAGCTGGACT
TCCTCAACAACCTGGCCACGGGCCTGGTGTTCATCATCGTGGTAGTCAACATCTTCATCACGGCCTTCGG
GGTCCAGAAGCCCTTGATGGACATGGCACCCCAGCAGTAGGACACCCAGGACCCTGGATGCTGCCTGCCC
TGCAACTCAGCTGCCCGACCCCAGGAGTCGCCATACCTGTGAGGTGTCCACCTCCCTGCACATGGCACTA
CCCAGACTGCCAGAGCCCAGGCTGGCCTCATCTGCACCATGTCCCCGGACCAGCCCTTGCTCTGACTGCG
GCCAAGCACCACGCAGGAGGCCACTCTTGTCTCTCAGCAGCTGTTCCCAGGAGGCAGCTCCCTCCTGGCA
CATGGGGGCTGGCCACAATAGCCCAGAGGGTCAGAACTGGACAGCTGCAGAGACCTGTGCCCAGAGAAGG
GTCTCGACCCACTCAAGGACACACAGCAGGTCCGTGGATGGGCTGGATGAGTGACCAGGGCCAGCCTCTG
TCTCAGGACATTCCAGAAGGACAAGGAGATGTCTCTCCCTCTCCCAAAGCACCAGCGTCCCTGCCTCCCG
TGGGCCCTGTCCGGGTTGCCCTGGTGACCCCAGCCTCTGTCCACTTCCTAACCCAGGGACCCTGCACAGC
CAGAACTGCCTTTGGCCCTACGGATGGCCACTGGCTCTGGTCTTAAGTGCCTGGGCTTGGTGGCCATCAA
GAGGGAGCCAGTCAGGCCTGTGAGGGCCGTAGACCTTGTATATACCCTGCACCAGCAGTGACCGGGCAGA
GCCCAACCCCCTCCACGGGGGTCCCAGCACCCACTTTTCTAATCATGAATGAACAATAAAGCCCACGCTC
TTTGTCAGGCTCCACATGCCAAAAAAAAAAAAAAAAA
>NP_004139.2 ninjurin-1 [Homo sapiens]
MDSGTEEYELNGGLPPGTPGSPDASPARWGWRHGPINVNHYASKKSAAESMLDIALLMANASQLKAVVEQ
GPSFAFYVPLVVLISISLVLQIGVGVLLIFLVKYDLNNPAKHAKLDFLNNLATGLVFIIVVVNIFITAFG
VQKPLMDMAPQQ
```

```
i1_HQ_samplec9816d|c43678/f2p0/1209      ------------------------------------------------------------ 0
i1_HQ_samplec9816d|c77387/f3p0/1237      ---------------------------------cgctcaggcccggatcctggcggcctgg 28
NM_004148.3                              CGCAGCTGGAGCCTGCGGCTGAGGCTCGGGCGCGCTCAGGCCCGGATCCTGGCGGCCTGG 60

i1_HQ_samplec9816d|c43678/f2p0/1209      ---GGCACACCCGGCTCCCCGGACGCCTCGCCCGCAGGTTGAAGGTTCCTGGGGCCACAT 57
i1_HQ_samplec9816d|c77387/f3p0/1237      gcggccgcaccatggactcgggaaccgaggagtacgaGCTCAA-CGGCGGCCTGCCTCCG 87
NM_004148.3                              GCGGCCGCACCATGGACTCGGGAACCGAGGAGTACGAGCTCAA-CGGCGGCCTGCCTCCG 119
                                            * * ****  *  * * ***  *   *    *  * * **    *     *** *

i1_HQ_samplec9816d|c43678/f2p0/1209      CTGGGCCGAGAGGCATGGTCCTTGGAGCCGGCCCGCTGGGGCTGGAGGCACGGGCCCATC 117
i1_HQ_samplec9816d|c77387/f3p0/1237      GGCACACCCGGCTCCCCGGACGCCTCGCCGGCCCGCTGGGGCTGGAGGCACGGGCCCATC 147
NM_004148.3                              GGCACACCCGGCTCCCCGGACGCCTCGCCGGCCCGCTGGGGCTGGAGGCACGGGCCCATC 179
                                               *  *   *   *  *     **********************************

i1_HQ_samplec9816d|c43678/f2p0/1209      AACGTGAACCATTACGCCAGCAAGAAGAGCGCAGCCGAGAGCATGCTGGACATCGCGCTG 177
i1_HQ_samplec9816d|c77387/f3p0/1237      AACGTGAACCATTACGCCAGCAAGAAGAGCGCAGCCGAGAGCATGCTGGACATCGCGCTG 207
NM_004148.3                              AACGTGAACCATTACGCCAGCAAGAAGAGCGCAGCCGAGAGCATGCTGGACATCGCGCTG 239
                                         ************************************************************

i1_HQ_samplec9816d|c43678/f2p0/1209      CTGATGGCCAACGCGTCCCAGCTGAAGGCCGTCGTGGAACAGGGCCCCAGCTTCGCCTTC 237
i1_HQ_samplec9816d|c77387/f3p0/1237      CTGATGGCCAACGCGTCCCAGCTGAAGGCCGTCGTGGAACAGGGCCCCAGCTTCGCCTTC 267
NM_004148.3                              CTGATGGCCAACGCGTCCCAGCTGAAGGCCGTCGTGGAACAGGGCCCCAGCTTCGCCTTC 299
                                         ************************************************************

i1_HQ_samplec9816d|c43678/f2p0/1209      TATGTGCCCCTGGTGGTCCTCATCTCCATCTCCCTTGTGCTGCAGATCGGCGTGGGGGTG 297
i1_HQ_samplec9816d|c77387/f3p0/1237      TATGTGCCCCTGGTGGTCCTCATCTCCATCTCCCTTGTGCTGCAGATCGGCGTGGGGGTG 327
NM_004148.3                              TATGTGCCCCTGGTGGTCCTCATCTCCATCTCCCTTGTGCTGCAGATCGGCGTGGGGGTG 359
                                         ************************************************************

i1_HQ_samplec9816d|c43678/f2p0/1209      CTGCTCATCTTCCTTGTCAAGTACGACCTTAACAACCCGGACAAGCACGCCAAGCTGGAC 357
i1_HQ_samplec9816d|c77387/f3p0/1237      CTGCTCATCTTCCTTGTCAAGTACGACCTTAACAACCCGGACAAGCACGCCAAGCTGGAC 387
NM_004148.3                              CTGCTCATCTTCCTTGTCAAGTACGACCTTAACAACCCGGCCAAGCACGCCAAGCTGGAC 419
                                         **************************************** *******************

i1_HQ_samplec9816d|c43678/f2p0/1209      TTCCTCAACAACCTGGCCACGGGCCTGGTGTTCATCATCGTGGTAGTCAACATCTTCATC 417
i1_HQ_samplec9816d|c77387/f3p0/1237      TTCCTCAACAACCTGGCCACGGGCCTGGTGTTCATCATCGTGGTAGTCAACATCTTCATC 447
NM_004148.3                              TTCCTCAACAACCTGGCCACGGGCCTGGTGTTCATCATCGTGGTAGTCAACATCTTCATC 479
                                         ************************************************************

i1_HQ_samplec9816d|c43678/f2p0/1209      ACGGCCTTCGGGGTCCAGAAGCCCTTGATGGACATGGCACCCCAGCAGTAGGACACCCAG 477
i1_HQ_samplec9816d|c77387/f3p0/1237      ACGGCCTTCGGGGTCCAGAAGCCCTTGATGGACATGGCACCCCAGCAGTAGGACACCCAG 507
NM_004148.3                              ACGGCCTTCGGGGTCCAGAAGCCCTTGATGGACATGGCACCCCAGCAGTAGGACACCCAG 539
                                         ************************************************************

i1_HQ_samplec9816d|c43678/f2p0/1209      GACCCTGGATGCTGCCTGCCCTGCAACTCAGCTGCCCGACCCCAGGAGTCGCCATACCTG 537
i1_HQ_samplec9816d|c77387/f3p0/1237      GACCCTGGATGCTGCCTGCCCTGCAACTCAGCTGCCCGACCCCAGGAGTCGCCATACCTG 567
NM_004148.3                              GACCCTGGATGCTGCCTGCCCTGCAACTCAGCTGCCCGACCCCAGGAGTCGCCATACCTG 599
                                         ************************************************************

i1_HQ_samplec9816d|c43678/f2p0/1209      TGAGGTGTCCACCTCCCTGCACATGGCACTACCCAGACTGCCAGAGCCCAGGCTGGCCTC 597
i1_HQ_samplec9816d|c77387/f3p0/1237      TGAGGTGTCCACCTCCCTGCACATGGCACTACCCAGACTGCCAGAGCCCAGGCTGGCCTC 627
NM_004148.3                              TGAGGTGTCCACCTCCCTGCACATGGCACTACCCAGACTGCCAGAGCCCAGGCTGGCCTC 659
                                         ************************************************************

i1_HQ_samplec9816d|c43678/f2p0/1209      ATCTGCACCATGTCCCCGGACCAGCCCTTGCTCTGACTGCGGCCAAGCACCACGCAGGAG 657
i1_HQ_samplec9816d|c77387/f3p0/1237      ATCTGCACCATGTCCCCGGACCAGCCCTTGCTCTGACTGCGGCCAAGCACCACGCAGGAG 687
```

```
NM_004148.3                         ATCTGCACCATGTCCCCGGACCAGCCCTTGCTCTGACTGCGGCCAAGCACCACGCAGGAG 719
                                    ************************************************************

i1_HQ_samplec9816d|c43678/f2p0/1209 GCCACTCTTGTCTCTCAGCAGCTGTTCCCAGGAGGCAGCTCCCTCCTGGCACATGGGGGC 717
i1_HQ_samplec9816d|c77387/f3p0/1237 GCCACTCTTGTCTCTCAGCAGCTGTTCCCAGGAGGCAGCTCCCTCCTGGCACATGGGGGC 747
NM_004148.3                         GCCACTCTTGTCTCTCAGCAGCTGTTCCCAGGAGGCAGCTCCCTCCTGGCACATGGGGGC 779
                                    ************************************************************

i1_HQ_samplec9816d|c43678/f2p0/1209 TGGCCACAATAGCCCAGAGGGTCAGAACTGGACAGCTGCAGAGACCTGTGCCCAGAGAAG 777
i1_HQ_samplec9816d|c77387/f3p0/1237 TGGCCACAATAGCCCAGAGGGTCAGAACTGGACAGCTGCAGAGACCTGTGCCCAGAGAAG 807
NM_004148.3                         TGGCCACAATAGCCCAGAGGGTCAGAACTGGACAGCTGCAGAGACCTGTGCCCAGAGAAG 839
                                    ************************************************************

i1_HQ_samplec9816d|c43678/f2p0/1209 GGTCTCGACCCACTCAAGGACACACAGCAGGTCCGTGGATGGGCTGGATGAGTGACCAGG 837
i1_HQ_samplec9816d|c77387/f3p0/1237 GGTCTCGACCCACTCAAGGACACACAGCAGGTCCGTGGATGGGCTGGATGAGTGACCAGG 867
NM_004148.3                         GGTCTCGACCCACTCAAGGACACACAGCAGGTCCGTGGATGGGCTGGATGAGTGACCAGG 899
                                    ************************************************************

i1_HQ_samplec9816d|c43678/f2p0/1209 GCCAGCCTCTGTCTCAGGACATTCCAGAAGGACAAGGAGATGTCTCTCCCTCTCCCAAAG 897
i1_HQ_samplec9816d|c77387/f3p0/1237 GCCAGCCTCTGTCTCAGGACATTCCAGAAGGACAAGGAGATGTCTCTCCCTCTCCCAAAG 927
NM_004148.3                         GCCAGCCTCTGTCTCAGGACATTCCAGAAGGACAAGGAGATGTCTCTCCCTCTCCCAAAG 959
                                    ************************************************************

i1_HQ_samplec9816d|c43678/f2p0/1209 CACCAGCGTCCCTGCCTCCCGTGGGCCCTGTCCGGGTTGCCCTGGTGACCCCAGCCTCTG 957
i1_HQ_samplec9816d|c77387/f3p0/1237 CACCAGCGTCCCTGCCTCCCGTGGGCCCTGTCCGGGTTGCCCTGGTGACCCCAGCCTCTG 987
NM_004148.3                         CACCAGCGTCCCTGCCTCCCGTGGGCCCTGTCCGGGTTGCCCTGGTGACCCCAGCCTCTG 1019
                                    ************************************************************

i1_HQ_samplec9816d|c43678/f2p0/1209 TCCACTTCCTAACCCAGGGACCCTGCACAGCCAGAACTGCCTTTGGCCCTACGGATGGCC 1017
i1_HQ_samplec9816d|c77387/f3p0/1237 TCCACTTCCTAACCCAGGGACCCTGCACAGCCAGAACTGCCTTTGGCCCTACGGATGGCC 1047
NM_004148.3                         TCCACTTCCTAACCCAGGGACCCTGCACAGCCAGAACTGCCTTTGGCCCTACGGATGGCC 1079
                                    ************************************************************

i1_HQ_samplec9816d|c43678/f2p0/1209 ACTGGCTCTGGTCTAAAGTGCCTGGGCTTGGTGGCCATCAAGAGGGAGCCAGTCAGGCCT 1077
i1_HQ_samplec9816d|c77387/f3p0/1237 ACTGGCTCTGGTCTAAAGTGCCTGGGCTTGGTGGCCATCAAGAGGGAGCCAGTCAGGCCT 1107
NM_004148.3                         ACTGGCTCTGGTCTTAAGTGCCTGGGCTTGGTGGCCATCAAGAGGGAGCCAGTCAGGCCT 1139
                                    ************** *********************************************

i1_HQ_samplec9816d|c43678/f2p0/1209 GTGAGGGCCGTAGACCTTGTATATACCCTGCACCAGCAGTGACCGGGCAGAGCCCAACCC 1137
i1_HQ_samplec9816d|c77387/f3p0/1237 GTGAGGGCCGTAGACCTTGTATATACCCTGCACCAGCAGTGACCGGGCAGAGCCCAACCC 1167
NM_004148.3                         GTGAGGGCCGTAGACCTTGTATATACCCTGCACCAGCAGTGACCGGGCAGAGCCCAACCC 1199
                                    ************************************************************

i1_HQ_samplec9816d|c43678/f2p0/1209 CCTCCACGGGGGTCCCAGCACCCACTTTTCTAATCATGAATgaacaatataaagcccacg 1197
i1_HQ_samplec9816d|c77387/f3p0/1237 CCTCCACGGGGGTCCCAGCACCCACTTTTCTAATCATGAATGAACAATaaagcccacgct 1227
NM_004148.3                         CCTCCACGGGGGTCCCAGCACCCACTTTTCTAATCATGAATGAACAATAAAGCCCACGCT 1259
                                    ************************************************* *   * * *

i1_HQ_samplec9816d|c43678/f2p0/1209 ctctttgtcagg--------------------------   1209
i1_HQ_samplec9816d|c77387/f3p0/1237 ctttgtcagg----------------------------   1237
NM_004148.3                         CTTTGTCAGGCTCCACATGCCAAAAAAAAAAAAAAAAA   1297
                                    ** * *
```

```
NP_004139.2         -----------------------------------------------------MDSGT--      5
VIRT68216           MLPALQLSCPTPGVAIPVRCPPPCTWHYPDCQSPGWPHLHHVPGPALALTAAKHHAGGHS      60
VIRT69015           MLPALQLSCPTPGVAIPVRCPPPCTWHYPDCQSPGWPHLHHVPGPALALTAAKHHAGGHS      60
                                                                          .:*

NP_004139.2         --EEYELNGGLPPGTP--------------------------------------------      19
VIRT68216           CLSAAVPRRQLPPGTWGLATIAQRVRTGQLQRPVPREGSRPTQGHTAGPWMGWMSDQGQP      120
VIRT69015           CLSAAVPRRQLPPGTWGLATIAQRVRTGQLQRPVPREGSRPTQGHTAGPWMGWMSDQGQP      120
                      .    .  *****

NP_004139.2         -------------------------------------------------GSPDASPARWG      30
VIRT68216           LSQDIPEGQGDVSPSPKAPASLPPVGPVRVALVTPASVHFLTQGPCTARTAFGPTDGHWL      180
VIRT69015           LSQDIPEGQGDVSPSPKAPASLPPVGPVRVALVTPASVHFLTQGPCTARTAFGPTDGHWL      180
                                                                      : . : .:*

NP_004139.2         WRHGPINVNHYASKKSAAESMLDIALLMANASQLKAVVEQGPSFAFYVPLVVLISISLVL      90
VIRT68216           WSKVPGLGGHQEGASQACEGRRPCIY------PAPAVTGQSPTPSTGVPA----------      224
VIRT69015           WSKVPGLGGHQEGASQACEGRRPCIY------PAPAVTGQSPTPSTGVPA----------      224
                    * : *   .*  . ..*.*.               **. *.*: :  **

NP_004139.2         QIGVGVLLIFLVKYDLNNPAKHAKLDFLNNLATGLVFIIVVVNIFITAFGVQKPLMDMAP      150
VIRT68216           ----PTFLIMNEQYKAHALCQ---------------------------------------      241
VIRT69015           ----PTFLIMNEQ-----------------------------------------------      233
                         .:**:  :

NP_004139.2         QQ     152
VIRT68216           --     241
VIRT69015           --     233
```

## SMIM7

| Sequence | Header | AA Seq |
|---|---|---|
| Present | i1_HQ_samplec9816d\|c15024/f2p0/1053 | |
| | | |
| Absent | i0_HQ_samplec9816d\|c421/f18p17/953 | |
| NCBI | NM_024104 | |
| | NM_001300925 | |

>i1_HQ_samplec9816d|c15024/f2p0/1053
isoform=c15024;full_length_coverage=2;non_full_length_coverage=0;isoform_length=1053

gacggacgcttcgccgtaacgatgatcggagacatcctgctgttcgggacgttgctgatgaatGCCGGGCGGTGCTGAACTTTAAGCTGAAAA
AGAAGGACACGCAGGGCTTTGGGGAGGAGTCCAGGGAGCCCAGCACAGGGCTTGAGAGGGAGAGTGATTGACTGGA
AACCCTGGAAGAAGGGTCTAGAACCCAGGTCTCTGGAGTCTTCACGTACTAAATTGCCTTCCCTCGGAGGGGTTTCTGT
TCCTTGGCATTCAAGGGGACCAGAGTGACAACATCCGGGAATTCTTGCTGAGCCTCAGATACTTTCGAATCTTCATCGCC
CTGTGGAACATCTTCATGATGTTCTGCATGATTGTGCTGTTCGGCTCTTGAATCCCAGCGATGAAACCAGGAACTCACTT
TCCCGGGATGCCGAGTCTCCATTCCTCCATTCCTGATGACTTCAAGAATGTTTTTGACCAGAAAACCGACAACCTTCCCA
GAAAGTCCAAGCTCGTGGTGGGTGGAAAAGTGTTCGCCGAGGTGTGCATGGTTTCCCAGCCACGTCCCTGTTTTCAAA
GATAGTTTCACTTTGGTCTCTGAATTGAAATGCTGTCTACTGAAAGGGTTTCAGGAGCGTTTATGTAAGGGGCTGTGAT
GAAATTGCATTCCCCATAGATAAAAGAAAAATCATTTCTATCCAGAGATCTGAGCAGAAGGATTGGCTTGTTAGTTTAA
CACAGCCGTATTTTTGGACATTCAGTGTTACTTGCTGAGTCTGACAGCCTCTGGGCCCGGCCAGGGGCCCTGTTAACAA
ACTGCTTTCACATCCCAACAGGGTCTGCTTGGCCACTCAGTGCAGCTGCGATTAACCCTAAAGGCTTTAAGGAACGGGC
CACCTGTAACAGAGACACCAGCCTTCCTGTATAGACACTAAATTGTTAGCAAGAGTGTTGAGCTAGTTCCTGGTGAAGT
GTTTCCACAGAAGACATGTGGAGCAGTTGTGGGGATATTAAGGGAAACTTTCCTctgccttgaccctttgttaaataaaatgactttg
ggag

>VIRT77545

MTSRMFLTRKPTTFPESPSSWWVEKCSPRCAWFPSHVPVFKDSFTLVSELKCCLLKGFQE
RLCKGL

>i0_HQ_samplec9816d|c421/f18p17/953
isoform=c421;full_length_coverage=18;non_full_length_coverage=17;isoform_length=953

ggcttccgcttccggttctGACGGACGCTTCGGCCGTAACGATGATCGGAGACATCCTGCTGTTCGGGACGTTGCTGATGAAT
GCCGGGGCGGTGCTGAACTTTAAGCTGAAAAAGAAGGACACGCAGGGCTTTGGGGAGGAGTCCAGGGAGCCCAGCA
CAGGTGACAACATCCGGGAATTCTTGCTGAGCCTCAGATACTTTCGAATCTTCATCGCCCTGTGGAACATCTTCATGATG
TTCTGCATGATTGTGCTGTTCGGCTCTTGAATCCCAGCGATGAAACCAGGAACTCACTTTCCCGGGATGCCGAGTCTCCA
TTCCTCCATTCCTGATGACTTCAAGAATGTTTTTGACCAGAAAACCGACAACCTTCCCAGAAAGTCCAAGCTCGTGGTGG
GTGGAAAAGTGTTCGCCGAGGTGTGCATGGTTTCCCAGCCACGTCCCTGTTTTCAAAGATAGTTTCACTTTGGTCTCTGA
ATTGAAATGCTGTCTACTGAAAGGGTTTCAGGAGCGTTTATGTAAGGGGCTGTGATGAAATTGCATTCCCCATAGATAA
AAGAAAAATCATTTCTATCCAGAGATCTGAGCAGAAGGATTGGCTTGTTAGTTTAACACAGCCGTATTTTTGGACATTCA
GTGTTACTTGCTGAGTCTGACAGCCTCTGGGCCCGGCCAGGGGCCCTGTTAACAAACTGCTTTCACATCCCAACAGGGT
CTGCTTGGCCACTCAGTGCAGCTGCGATTAACCCTAAAGGCTTTAAGGAACGGGCCACCTGTAACAGAGACACCAGCCT
TCCTGTATAGACACTAAATTGTTAGCAAGAGTGTTGAGCTAGTTCCTGGTGAAGTGTTTCCACAGAAGACATGTGGAGC
AGTTGTGGGGATATTAAGGGAAACTTTCCTCTGCCTTGACCCCTTTGTTAAATAAAATGACTTTGGGAGCCAttcatt

>VIRT83180

MKPGTHFPGMPSLHSSIPDDFKNVFDQKTDNLPRKSKLVVGGKVFAEVCMVSQPRPCFQR

# >VIRT83564

MIGDILLFGTLLMNAGAVLNFKLKKKDTQGFGEESREPSTGDNIREFLLSLRYFRIFIAL
WNIFMMFCMIVLFGS

```
>NM_024104.3 Homo sapiens small integral membrane protein 7 (SMIM7), transcript variant 2, mRNA
CCCTGGGGCTTCCGCTTCCGGTTCTGACGGACGCTTCGGCCGTAACGATGATCGGAGACATCCTGCTGTT
CGGGACGTTGCTGATGAATGCCGGGGCGGTGCTGAACTTTAAGCTGAAAAAGAAGGACACGCAGGGCTTT
GGGGAGGAGTCCAGGGAGCCCAGCACAGGTGACAACATCCGGGAATTCTTGCTGAGCCTCAGATACTTTC
GAATCTTCATCGCCCTGTGGAACATCTTCATGATGTTCTGCATGATTGTGCTGTTCGGCTCTTGAATCCC
AGCGATGAAACCAGGAACTCACTTTCCCGGGATGCCGAGTCTCCATTCCTCCATTCCTGATGACTTCAAG
AATGTTTTTGACCAGAAAACCGACAACCTTCCCAGAAAGTCCAAGCTCGTGGTGGGTGGAAAAGTGTTCG
CCGAGGTGTGCATGGTTTCCCAGCCACGTCCCTGTTTTCAAAGATAGTTTCACTTTGGTCTCTGAATTGA
AATGCTGTCTACTGAAAGGGTTTCAGGAGCGTTTATGTAAGGGGCTGTGATGAAATTGCATTCCCCATAG
ATAAAAGAAAAATCATTTCTATCCAGAGATCTGAGCAGAAGGATTGGCTTGTTAGTTTAACACAGCCGTA
TTTTTGGACATTCAGTGTTACTTGCTGAGTCTGACAGCCTCTGGGCCCGGCCAGGGGCCCTGTTAACAAA
CTGCTTTCACATCCCAACAGGGTCTGCTTGGCCACTCAGTGCAGCTGCGATTAACCCTAAAGGCTTTAAG
GAACGGGCCACCTGTAACAGAGACACCAGCCTTCCTGTATAGACACTAAATTGTTAGCAAGAGTGTTGAG
CTAGTTCCTGGTGAAGTGTTTCCACAGAAGACATGTGGAGCAGTTGTGGGGATATTAAGGGAAACTTTCC
TCTGCCTTGACCCCTTTGTTAAATAAAATGACTTTGGGAGCCATTCATTGTACAGTTGCAGGAATGAGAG
TGATTTTATGATGTGGTACATTGGGACCATGTTCTAAAACCTTGGGTTTCTGAGTCTGCTTTTTGAGTAG
GTGATTTTGAGGTTGAAAAACCAGGGGCCTTCATCTAGGAAATACAGCATTTTCCAGAAGCTTCTTTGAA
AGGGAATCCTGGTTTTGTTGCCAAAATGAAACGCCCGGGGTTGGCGCTGAATCCCACAACTGTGTGATTT
GCTTGTTGAGTTTTTTGTTGTCTGGTTTTTTTGTTTGTTTGTTTATACCAATAAGAATGAGCCTGAATGT
TGGTGGTTTTTGAAATCCTGACTTGGAGGTAAACCTGGAGGAAGGAAAAAAAGTAAATATGCAGGCTTTT
AGGACTGAGTAGCCTTGAAAATAAACCTCATTTCTAAAAAGGAAAAAAAAAAAAAAAAAA
>NP_077009.2 small integral membrane protein 7 isoform 2 precursor [Homo sapiens]
MIGDILLFGTLLMNAGAVLNFKLKKKDTQGFGEESREPSTGDNIREFLLSLRYFRIFIALWNIFMMFCMI
VLFGS
>NM_001300925.1 Homo sapiens small integral membrane protein 7 (SMIM7), transcript variant 1, mRNA
CCCTGGGGCTTCCGCTTCCGGTTCTGACGGACGCTTCGGCCGTAACGATGATCGGAGACATCCTGCTGTT
CGGGACGTTGCTGATGAATGCCGGGGCGGTGCTGAACTTTAAGCTGAAAAAGAAGGACACGCAGGGCTTT
GGGGAGGAGTCCAGGGAGCCCAGCACAGGTGACAACATCCGGGAATTCTTGCTGAGCCTCAGATACTTTC
GAATCTTCATCGCCCTGTGGAACATCTTCATGATGTTCTGCATGATTGTGCTGAGAAAACTCAAGCAGAT
TGCCTTTCCATCTAGCACTGGGGCCATAACTCTGATACTACTGTTAACGAATTGTGAGATTTGCTGTAAA
TGGATTTAGGAAAAGACACGACGGGAGAATCTGGTGTTCTTGGCCTCTGTGGGTCCATCCCTGTATGCTT
CCCCCAGAAAGAGAGAGGACAAAAAGTTGTCTGTCCTGAGTGATTGAGACTCCGAATGTGGGGTTGCTGT
CCAGTCTGCGTGCCACCCCAGTGGCTGTTTGCGAAGATGCTTCCAGGACATAGGAGATCTGGGGAGGATG
GCATTTCACCCTCATCACAAGGACCGTGATGTATTCCCAGGAAGACTGTTGCCAAGGTATCCTACCTGGC
AGAGAGGATCTGAGAGCATGTATGAGACTTGCCTAGGAACAAAGAAGGCTTGTGAGGAGATCAAGGAACT
TACATGAAATTGTAGGAACGGCCAGGGGCAGTGGCTCAGGCCTGTAATCCCAGTACTTTGGGAGGCCAAG
GTGGGAGAATTGCTTGAGCCCAGGAGTTCAAGACCAGCCTGGGCAGCATGGTGAAACCCTGTCTTTACCA
AAAATAAAAAAATTAGCCGAGTGTAAAAAAAAAAAAAAAAA
>NP_001287854.1 small integral membrane protein 7 isoform 1 precursor [Homo sapiens]
MIGDILLFGTLLMNAGAVLNFKLKKKDTQGFGEESREPSTGDNIREFLLSLRYFRIFIALWNIFMMFCMI
VLRKLKQIAFPSSTGAITLILLLTNCEICCKWI
```

```
NM_001300925.1                      ------------------------------------------------------------     0
i1_HQ_samplec9816d|c15024/f2p0/1053 gacggacgcttcgccgtaacgatgatcggagacatcctgctgttcgggacgttgctgatg     60
i0_HQ_samplec9816d|c421/f18p17/953  ------------------------------------------------------------     0
NM_024104.3                         ------------------------------------------------------------     0

NM_001300925.1                      -----------------------------------------CCCTGGGGCTTCCGCTTCC     19
i1_HQ_samplec9816d|c15024/f2p0/1053 aatGCCGGGCGGTGCTGAACTTTAAGCTGAAAAAGAAGGACACGCAGGGCTTTGGGGAGG     120
i0_HQ_samplec9816d|c421/f18p17/953  -----------------------------------------------ggcttccgcttcc     13
NM_024104.3                         -----------------------------------------CCCTGGGGCTTCCGCTTCC     19
                                                                                *****   *

NM_001300925.1                      GGTTCTGAC-GGACGCTTCGGCCGTAACGATGATCGGAGACATCCT-GCTGTTCGGGACG     77
i1_HQ_samplec9816d|c15024/f2p0/1053 AGTCCAGGGAGCCCAGCACAGGGCTTGAGAGGGAGAGTGATTGACTGGAAACCCTGGAAG     180
i0_HQ_samplec9816d|c421/f18p17/953  ggttctGAC-GGACGCTTCGGCCGTAACGATGATCGGAGACATCCT-GCTGTTCGGGACG     71
NM_024104.3                         GGTTCTGAC-GGACGCTTCGGCCGTAACGATGATCGGAGACATCCT-GCTGTTCGGGACG     77
                                     ** * *   *  *    * *   *   ** *    * **    ** *     * *** *

NM_001300925.1                      TTGCTGATGAATGCCGGGGCGGTGCTGAA-CTTTAAGCTGAAAAAGAAGGACACGCAGGG     136
```

```
i1_HQ_samplec9816d|c15024/f2p0/1053      AAGGGTCTAGAACCCAGGTCTCTGGAGTCTTCACGTACTAAATTGCCTTCCCTCGGAGGG     240
i0_HQ_samplec9816d|c421/f18p17/953       TTGCTGATGAATGCCGGGGCGGTGCTGAA-CTTTAAGCTGAAAAAGAAGGACACGCAGGG     130
NM_024104.3                              TTGCTGATGAATGCCGGGGCGGTGCTGAA-CTTTAAGCTGAAAAAGAAGGACACGCAGGG     136
                                           *    *  *  ** ** *  **  *          ** **         * ** ****

NM_001300925.1                           CTTTGGGGAGGAGTCCAGGGAGCCCAGCACAGGTGACAACATCCGGGAATTCTTGCTGAG     196
i1_HQ_samplec9816d|c15024/f2p0/1053      GTTTCTGTTCCTTGGCATTCAAGGGGACCAGAGTGACAACATCCGGGAATTCTTGCTGAG     300
i0_HQ_samplec9816d|c421/f18p17/953       CTTTGGGGAGGAGTCCAGGGAGCCCAGCACAGGTGACAACATCCGGGAATTCTTGCTGAG     190
NM_024104.3                              CTTTGGGGAGGAGTCCAGGGAGCCCAGCACAGGTGACAACATCCGGGAATTCTTGCTGAG     196
                                          ***  *        **   *      *    ****************************

NM_001300925.1                           CCTCAGATACTTTCGAATCTTCATCGCCCTGTGGAACATCTTCATGATGTTCTGCATGAT     256
i1_HQ_samplec9816d|c15024/f2p0/1053      CCTCAGATACTTTCGAATCTTCATCGCCCTGTGGAACATCTTCATGATGTTCTGCATGAT     360
i0_HQ_samplec9816d|c421/f18p17/953       CCTCAGATACTTTCGAATCTTCATCGCCCTGTGGAACATCTTCATGATGTTCTGCATGAT     250
NM_024104.3                              CCTCAGATACTTTCGAATCTTCATCGCCCTGTGGAACATCTTCATGATGTTCTGCATGAT     256
                                         ************************************************************

NM_001300925.1                           TGTGCTGAGAAAACTCAAGCAGATTGCCTTTCCATCTAGCACTGGGGCCATAACT---CT     313
i1_HQ_samplec9816d|c15024/f2p0/1053      TGTGCTGTTCGGCTCTTGAATCCCAGCGATGAAACCAGGAACTCACTTTCCCGGGATGCC     420
i0_HQ_samplec9816d|c421/f18p17/953       TGTGCTGTTCGGCTCTTGAATCCCAGCGATGAAACCAGGAACTCACTTTCCCGGGATGCC     310
NM_024104.3                              TGTGCTGTTCGGCTCTTGAATCCCAGCGATGAAACCAGGAACTCACTTTCCCGGGATGCC     316
                                         *******                  **  *   * *  * ***               *

NM_001300925.1                           GATACTACTGTTAACGAATTGTGAGATTTGCTGTAAATGGATTTAGGAAAAGACACGAC-     372
i1_HQ_samplec9816d|c15024/f2p0/1053      GAGTCTCCATTCCTCCATTCCTGATGACTTCAAGAA-TGTTTTTGACCAGAAAACCGACA     479
i0_HQ_samplec9816d|c421/f18p17/953       GAGTCTCCATTCCTCCATTCCTGATGACTTCAAGAA-TGTTTTTGACCAGAAAACCGACA     369
NM_024104.3                              GAGTCTCCATTCCTCCATTCCTGATGACTTCAAGAA-TGTTTTTGACCAGAAAACCGACA     375
                                         **  ** *  *   * * *  ***    * *   ** **  ***    * * *  ****

NM_001300925.1                           ------------GGGAGAATCTGGTGTTCTT--GGCCTCTGTGGGTCCATCCCTGTATGC     418
i1_HQ_samplec9816d|c15024/f2p0/1053      ACCTTCCCAGAAAGTCCAAGCTCGTGGTGGGTGGAAAAGTGTTCGCCGAGGTGTGCATGG     539
i0_HQ_samplec9816d|c421/f18p17/953       ACCTTCCCAGAAAGTCCAAGCTCGTGGTGGGTGGAAAAGTGTTCGCCGAGGTGTGCATGG     429
NM_024104.3                              ACCTTCCCAGAAAGTCCAAGCTCGTGGTGGGTGGAAAAGTGTTCGCCGAGGTGTGCATGG     435
                                                      *   ** ** *** *     *     ***  * * *    ** ***

NM_001300925.1                           TTCCCCCAGAAAGAGAGAGGACAAAAAGTTGTCTGTCCTGAGTGATTGAGACTCCGAATG     478
i1_HQ_samplec9816d|c15024/f2p0/1053      TTTCCCAGCCACGTCCCTGTTTTCAAAGATAGTTTCACT-------TTGGTCTCTGAAT-     591
i0_HQ_samplec9816d|c421/f18p17/953       TTTCCCAGCCACGTCCCTGTTTTCAAAGATAGTTTCACT-------TTGGTCTCTGAAT-     481
NM_024104.3                              TTTCCCAGCCACGTCCCTGTTTTCAAAGATAGTTTCACT-------TTGGTCTCTGAAT-     487
                                         ** ***    * *     *     **** *   *   **       *  * *** ****

NM_001300925.1                           TGGGGTTGCTGTCCAGTCTGCG--TGCCACCCCAGTGGCTGTTTGCGAAGATGCTTCCAG     536
i1_HQ_samplec9816d|c15024/f2p0/1053      -TGAAATGCTGTCTACTGAAAGGGTTTCAGGAGCGTTTATGTAAGGGGCTGTGATGAAAT     650
i0_HQ_samplec9816d|c421/f18p17/953       -TGAAATGCTGTCTACTGAAAGGGTTTCAGGAGCGTTTATGTAAGGGGCTGTGATGAAAT     540
NM_024104.3                              -TGAAATGCTGTCTACTGAAAGGGTTTCAGGAGCGTTTATGTAAGGGGCTGTGATGAAAT     546
                                           *   ******* * *    *  *  **     **   ***  * *    ** *   *

NM_001300925.1                           GACATA--------------------------------GGAGATCTGG---GGAGGATGG     561
i1_HQ_samplec9816d|c15024/f2p0/1053      TGCATTCCCCATAGATAAAAGAAAAATCATTTCTATCCAGAGATCTGAGCAGAAGGATTG     710
i0_HQ_samplec9816d|c421/f18p17/953       TGCATTCCCCATAGATAAAAGAAAAATCATTTCTATCCAGAGATCTGAGCAGAAGGATTG     600
NM_024104.3                              TGCATTCCCCATAGATAAAAGAAAAATCATTTCTATCCAGAGATCTGAGCAGAAGGATTG     606
                                           ***                                  ********    * ***** *

NM_001300925.1                           CATTTCACCCTCATCACAAGGACCGTGATGTATTCCCAGGAAGACTGTTGCCAAGGTATC     621
i1_HQ_samplec9816d|c15024/f2p0/1053      GCTTGTTAGTTTAACACAGCCGTATTTTTGGACATTCAGTGT--TACTTGCTGAGTCTGA     768
i0_HQ_samplec9816d|c421/f18p17/953       GCTTGTTAGTTTAACACAGCCGTATTTTTGGACATTCAGTGT--TACTTGCTGAGTCTGA     658
NM_024104.3                              GCTTGTTAGTTTAACACAGCCGTATTTTTGGACATTCAGTGT--TACTTGCTGAGTCTGA     664
                                           **      * * ****       *  ** *    ***        ****  **

NM_001300925.1                           CTACCTGGCAGAGAGGATCTGAGAGCATGTATGAGACTTGCCTAGGAACAAAG--AAGGC     679
i1_HQ_samplec9816d|c15024/f2p0/1053      CAGCCTCTGGGCCCGGCCAGGGGCCCTGTTAACAAACTGCTTTCACATCCCAACAGGGTC     828
i0_HQ_samplec9816d|c421/f18p17/953       CAGCCTCTGGGCCCGGCCAGGGGCCCTGTTAACAAACTGCTTTCACATCCCAACAGGGTC     718
NM_024104.3                              CAGCCTCTGGGCCCGGCCAGGGGCCCTGTTAACAAACTGCTTTCACATCCCAACAGGGTC     724
                                         *  ***    *   **    * *  *   **  * ***    *   * *  *     * *

NM_001300925.1                           TTGTGAGGAGATCAAGGAACTTACATGAAA----------TTGTAGGAACGG-CC---AG     725
i1_HQ_samplec9816d|c15024/f2p0/1053      TGCTTGGCCACTCAGTGCAGCTGCGATTAACCCTAAAGGCTTTAAGGAACGGGCCACCTG     888
i0_HQ_samplec9816d|c421/f18p17/953       TGCTTGGCCACTCAGTGCAGCTGCGATTAACCCTAAAGGCTTTAAGGAACGGGCCACCTG     778
NM_024104.3                              TGCTTGGCCACTCAGTGCAGCTGCGATTAACCCTAAAGGCTTTAAGGAACGGGCCACCTG     784
                                         *  *  *    ***  * *  * *    **          **  ******** **    *

NM_001300925.1                           GGGCAGTGG----CTCAGGCCTGTAATCCCAGTACTTTGGGAGGCCAA--GGTGGGAGA-     778
i1_HQ_samplec9816d|c15024/f2p0/1053      TAACAGAGACACCAGCCTTCCTGTATAGACACTAAATTGTTAGCAAGAGTGTTGAGCTAG     948
i0_HQ_samplec9816d|c421/f18p17/953       TAACAGAGACACCAGCCTTCCTGTATAGACACTAAATTGTTAGCAAGAGTGTTGAGCTAG     838
NM_024104.3                              TAACAGAGACACCAGCCTTCCTGTATAGACACTAAATTGTTAGCAAGAGTGTTGAGCTAG     844
                                            *** *       *   ******    ** **  ***  **    *  * ** *  *

NM_001300925.1                           ----------ATTGCTTGAGCCCAGGAGTTCAAGACCAGCCTGGGCAGCATGGT-GAAAC     827
i1_HQ_samplec9816d|c15024/f2p0/1053      TTCCTGGTGAAGTGTTTCCACAGAAGACATGTGGAGCAGTTGTGGGGATATTAAGGGAAA     1008
i0_HQ_samplec9816d|c421/f18p17/953       TTCCTGGTGAAGTGTTTCCACAGAAGACATGTGGAGCAGTTGTGGGGATATTAAGGGAAA     898
NM_024104.3                              TTCCTGGTGAAGTGTTTCCACAGAAGACATGTGGAGCAGTTGTGGGGATATTAAGGGAAA     904
                                                   * ** **   *  * **  *   ** ***    **    **    * **

NM_001300925.1                           CCTG----------------TCTTTACCAAAAATAAAAAAATTAGCCGAGTGTAAAAAA     870
i1_HQ_samplec9816d|c15024/f2p0/1053      CTTTCCTctgccttgacccctttgttaaa-taaaatgactttgggag-------------     1053
i0_HQ_samplec9816d|c421/f18p17/953       CTTTCCTCTGCCTTGACCCCTTTGTTAAATAAAATGACTTTGGGAGCCAttcatt-----     953
NM_024104.3                              CTTTCCTCTGCCTTGACCCCTTTGTTAAATAAAATGACTTTGGGAGCCATTCATTGTACA     964
                                         * *                  *  * *   ***** *       **

NM_001300925.1                           AAAAAAAAAA--------------------------------------------------     880
i1_HQ_samplec9816d|c15024/f2p0/1053      ------------------------------------------------------------     1053
i0_HQ_samplec9816d|c421/f18p17/953       ------------------------------------------------------------     953
NM_024104.3                              GTTGCAGGAATGAGAGTGATTTTATGATGTGGTACATTGGGACCATGTTCTAAAACCTTG     1024
```

```
NM_001300925.1                          ------------------------------------------------------------    880
i1_HQ_samplec9816d|c15024/f2p0/1053     ------------------------------------------------------------    1053
i0_HQ_samplec9816d|c421/f18p17/953      ------------------------------------------------------------    953
NM_024104.3                             GGTTTCTGAGTCTGCTTTTTGAGTAGGTGATTTTGAGGTTGAAAAACCAGGGGCCTTCAT    1084

NM_001300925.1                          ------------------------------------------------------------    880
i1_HQ_samplec9816d|c15024/f2p0/1053     ------------------------------------------------------------    1053
i0_HQ_samplec9816d|c421/f18p17/953      ------------------------------------------------------------    953
NM_024104.3                             CTAGGAAATACAGCATTTTCCAGAAGCTTCTTTGAAAGGGAATCCTGGTTTTGTTGCCAA    1144

NM_001300925.1                          ------------------------------------------------------------    880
i1_HQ_samplec9816d|c15024/f2p0/1053     ------------------------------------------------------------    1053
i0_HQ_samplec9816d|c421/f18p17/953      ------------------------------------------------------------    953
NM_024104.3                             AATGAAACGCCCGGGGTTGGCGCTGAATCCCACAACTGTGTGATTTGCTTGTTGAGTTTT    1204

NM_001300925.1                          ------------------------------------------------------------    880
i1_HQ_samplec9816d|c15024/f2p0/1053     ------------------------------------------------------------    1053
i0_HQ_samplec9816d|c421/f18p17/953      ------------------------------------------------------------    953
NM_024104.3                             TTGTTGTCTGGTTTTTTTGTTTGTTTGTTTATACCAATAAGAATGAGCCTGAATGTTGGT    1264

NM_001300925.1                          ------------------------------------------------------------    880
i1_HQ_samplec9816d|c15024/f2p0/1053     ------------------------------------------------------------    1053
i0_HQ_samplec9816d|c421/f18p17/953      ------------------------------------------------------------    953
NM_024104.3                             GGTTTTTGAAATCCTGACTTGGAGGTAAACCTGGAGGAAGGAAAAAAAGTAAATATGCAG    1324

NM_001300925.1                          ------------------------------------------------------------    880
i1_HQ_samplec9816d|c15024/f2p0/1053     ------------------------------------------------------------    1053
i0_HQ_samplec9816d|c421/f18p17/953      ------------------------------------------------------------    953
NM_024104.3                             GCTTTTAGGACTGAGTAGCCTTGAAAATAAACCTCATTTCTAAAAAGGAAAAAAAAAAAA    1384

NM_001300925.1                          ------          880
i1_HQ_samplec9816d|c15024/f2p0/1053     ------          1053
i0_HQ_samplec9816d|c421/f18p17/953      ------          953
NM_024104.3                             AAAAAA          1390
```

```
VIRT83564            MIGDILLFGTLLMNAGAVLNFKLKKKDTQGFGEESREPSTGDNIREFLLSLRYFRIFIAL   60
NP_077009.2          MIGDILLFGTLLMNAGAVLNFKLKKKDTQGFGEESREPSTGDNIREFLLSLRYFRIFIAL   60
NP_001287854.1       MIGDILLFGTLLMNAGAVLNFKLKKKDTQGFGEESREPSTGDNIREFLLSLRYFRIFIAL   60
VIRT77545            ------------------------------------------------------------   0
VIRT83180            ------------------------------------------------------------   0

VIRT83564            WNIFMMFCMIVLFGS---------------------------------------------   75
NP_077009.2          WNIFMMFCMIVLFGS---------------------------------------------   75
NP_001287854.1       WNIFMMFCMIVLRKLKQIAFPSSTGAITL--ILLLTNCEICCKWI---------------   103
VIRT77545            ----MTSRMF--LTRKPTTFPESPSSWWVE------KCSPRCAWFPSH------VPVFKD   42
VIRT83180            -------------MKPGTHFPGMPSLHSSIPDDFKNVFDQKTDNLPRKSKLVVGGKVFAE   47

VIRT83564            ------------------------   75
NP_077009.2          ------------------------   75
NP_001287854.1       ------------------------   103
VIRT77545            SFTLVSELKCCLLKGFQERLCKGL   66
VIRT83180            -VCMVSQPRPCFQR----------   60
```

**PSME2 (**Exon 3 extended as well as two extra exons are present between 2&3**)**

| **Sequence** | **Header** | **AA Seq** |
|---|---|---|
| **Present** | i0_HQ_samplec9816d\|c30843/f3p5/855 | |
| | i0_HQ_samplec9816d\|c91455/f11p9/956 | |
| **Absent** | i0_HQ_samplec9816d\|c31918/f2p0/772 | |
| **NCBI** | NM_002818 | |
| | | |

>i0_HQ_samplec9816d|c30843/f3p5/855
isoform=c30843;full_length_coverage=3;non_full_length_coverage=5;isoform_length=855

GAGACCAGAGATCTAGCGACTGAAGCAGCATGGCCAAGCCGTGTGGGGTGCGCCTGAGCGGGGAAGCCCGCAAACA
GGTGGAGGTCTTCAGGCAGAATCTTTTCCAATTTATTTCATTTGCAGTTGGAGGTTCCTTCAGTTCATTTCAGAATCCAG

AATTCTTTTCCCAGAACAGGCTGAGGAATTCCTCTACAGATTCTTGCCACAGAAAATCATATACCTGAATCAGCTCTTGC
AAGAGGACTCCCTCAATGTGGCTGACTTGACTTCCCTCCGGGCCCCACTGGACATCCCCATCCCAGACCCTCCACCCAAG
GATGATGAGATGGAAACAGATAAGCAGGAGAAGAAAGAAGTCCCTAAGTGTGGATTTCTCCCTGGGAATGAGAAAGT
CCTGTCCCTGCTTGCCCTGGTTAAGCCAGAAGTCTGGACTCTCAAAGAGAAATGCATTCTGGTGATTACATGGATCCAAC
ACCTGATCCCCAAGATTGAAGATGGAAATGATTTTGGGGTAGCAATCCAGGAGAAGGTGCTGGAGAGGGTGAATGCC
GTCAAGACCAAAGTGGAAGCTTTCCAGACAACCATTTCCAAGTACTTCTCAGAACGTGGGGATGCTGTGGCCAAGGCCT
CCAAGGAGACTCATGTAATGGATTACCGGGCCTTGGTGCATGAGCGAGATGAGGCAGCCTATGGGGAGCTCAGGGCC
ATGGTGCTGGACCTGAGGGCCTTCTATGCTGAGCTTTATCATATCATCAGCAGCAACCTGGAGAAAATTGTCAACCCAA
AGGGTGAAGAAAAGCCATCTATGTACTGAACCCGGGACTAGAAggaaaataaatgatctatatgttgtgt

>VIRT18646

METDKQEKKEVPKCGFLPGNEKVLSLLALVKPEVWTLKEKCILVITWIQHLIPKIEDGND
FGVAIQEKVLERVNAVKTKVEAFQTTISKYFSERGDAVAKASKETHVMDYRALVHERDEA
AYGELRAMVLDLRAFYAELYHIISSNLEKIVNPKGEEKPSMY

>i0_HQ_samplec9816d|c91455/f11p9/956
isoform=c91455;full_length_coverage=11;non_full_length_coverage=9;isoform_length=956

ctggacctggggagtgaaagcgaaagcccgggcgactagccGGGAGACCAGAGATCTAGCGACTGAAGCAGCATGGCCAAGCCGTG
TGGGGTGCGCCTGAGCGGGGAAGCCCGCAAACAGGTGGAGGTCTTCAGGCAGAATCTTTTCCAGGAGGTCTTCAGTTT
CCTCATTTGCAAGTCTTGGACCCAGTGATTTCCAAAGGCCCTTCCAGTTCCATTTGTCAGACATCTCATTGAGCATATTCT
GTGATTCCCCTCCCTTGCAATGTAACCCACAGGCTGAGGAATTCCTCTACAGATTCTTGCCACAGAAAATCATATACCTG
AATCAGCTCTTGCAAGAGGACTCCCTCAATGTGGCTGACTTGACTTCCCTCCGGGCCCCACTGGACATCCCCATCCCAGA
CCCTCCACCCAAGGATGATGAGATGGAAACAGATAAGCAGGAGAAGAAAGAAGTCCCTAAGTGTGGATTTCTCCCTGG
GAATGAGAAAGTCCTGTCCCTGCTTGCCCTGGTTAAGCCAGAAGTCTGGACTCTCAAAGAGAAATGCATTCTGGTGATT
ACATGGATCCAACACCTGATCCCCAAGATTGAAGATGGAAATGATTTTGGGGTAGCAATCCAGGAGAAGGTGCTGGAG
AGGGTGAATGCCGTCAAGACCAAAGTGGAAGCTTTCCAGACAACCATTTCCAAGTACTTCTCAGAACGTGGGGATGCT
GTGGCCAAGGCCTCCAAGGAGACTCATGTAATGGATTACCGGGCCTTGGTGCATGAGCGAGATGAGGCAGCCTATGG
GGAGCTCAGGGCCATGGTGCTGGACCTGAGGGCCTTCTATGCTGAGCTTTATCATATCATCAGCAGCAACCTGGAGAA
AATTGTCAACCCAAAGGGTGAAGAAAAGCCATCTATGTACTGAACCCGGGACTAGAAGGAAAATAAATGATCTATATG
TTGTG

>VIRT19198

METDKQEKKEVPKCGFLPGNEKVLSLLALVKPEVWTLKEKCILVITWIQHLIPKIEDGND
FGVAIQEKVLERVNAVKTKVEAFQTTISKYFSERGDAVAKASKETHVMDYRALVHERDEA
AYGELRAMVLDLRAFYAELYHIISSNLEKIVNPKGEEKPSMY

>i0_HQ_samplec9816d|c31918/f2p0/772
isoform=c31918;full_length_coverage=2;non_full_length_coverage=0;isoform_length=772

tgagagcagcatggccaagccgtgtggggtgcgcctgagcggggaagcccgcaaacaggtggAGGTCTTCAGGCAGAATCTTTTCCAGGAG
GCTGAGGAATTCCTCTACAGATTCTTGCCACAGAAAATCATATACCTGAATCAGCTCTTGCAAGAGGACTCCCTCAATGT
GGCTGACTTGACTTCCCTCCGGGCCCCACTGGACATCCCCATCCCAGACCCTCCACCCAAGGATGATGAGATGGAAACA
GATAAGCAGGAGAAGAAAGAAGTCCCTAAGTGTGGATTTCTCCCTGGGAATGAGAAAGTCCTGTCCCTGCTTGCCCTG

GTTAAGCCAGAAGTCTGGACTCTCAAAGAGAAATGCATTCTGGTGATTACATGGATCCAACACCTGATCCCCAAGATTG
AAGATGGAAATGATTTTTGGGGTAGCAATCCAGGAGAAGGTGCTGGAGAGGGTGAATGCCGTCAAGACCAAAGTGGA
AGCTTTCCAGACAACCATTTCCAAGTACTTCTCAGAACGTGGGGATGCTGTGGCCAAGGCCTCCAAGGAGACTCATGTA
ATGGATTACCGGGCCTTGGTGCATGAGCGAGATGAGGCAGCCTATGGGGAGCTCAGGGCCATGGTGCTGGACCTGAG
GGCCTTCTATGCTGAGCTTTATCATATCATCAGCAGCAACCTGGAGAAAATTGTCAACCCAAAGGGTGAAGAAAAGCCA
TCTATGTACTGAACCCGGGACTAGAAGGAAAATAactgatctatatgttgtgt

```
>VIRT25459
```

```
MAKPCGVRLSGEARKQVEVFRQNLFQEAEEFLYRFLPQKIIYLNQLLQEDSLNVADLTSL
RAPLDIPIPDPPPKDDEMETDKQEKKEVPKCGFLPGNEKVLSLLALVKPEVWTLKEKCIL
VITWIQHLIPKIEDGNDFWGSNPGEGAGEGECRQDQSGSFPDNHFQVLLRTWGCCGQGLQ
GDSCNGLPGLGA
```

```
>NM_002818.2 Homo sapiens proteasome activator subunit 2 (PSME2), mRNA
TGGGGAGTGAAAGCGAAAGCCCGGGCGACTAGCCGGGAGACCAGAGATCTAGCGACTGAAGCAGCATGGC
CAAGCCGTGTGGGGTGCGCCTGAGCGGGGAAGCCCGCAAACAGGTGGAGGTCTTCAGACAGAATCTTTTC
CAGGAGGCTGAGGAATTCCTCTACAGATTCTTGCCACAGAAAATCATATACCTGAATCAGCTCTTGCAAG
AGGACTCCCTCAATGTGGCTGACTTGACTTCCCTCCGGGCCCCACTGGACATCCCCATCCCAGACCCTCC
ACCCAAGGATGATGAGATGGAAACAGATAAGCAGGAGAAGAAAGAAGTCCATAAGTGTGGATTTCTCCCT
GGGAATGAGAAAGTCCTGTCCCTGCTTGCCCTGGTTAAGCCAGAAGTCTGGACTCTCAAAGAGAAATGCA
TTCTGGTGATTACATGGATCCAACACCTGATCCCCAAGATTGAAGATGGAAATGATTTTGGGGTAGCAAT
CCAGGAGAAGGTGCTGGAGAGGGTGAATGCCGTCAAGACCAAAGTGGAAGCTTTCCAGACAACCATTTCC
AAGTACTTCTCAGAACGTGGGGATGCTGTGGCCAAGGCCTCCAAGGAGACTCATGTAATGGATTACCGGG
CCTTGGTGCATGAGCGAGATGAGGCAGCCTATGGGGAGCTCAGGGCCATGGTGCTGGACCTGAGGGCCTT
CTATGCTGAGCTTTATCATATCATCAGCAGCAACCTGGAGAAAATTGTCAACCCAAAGGGTGAAGAAAAG
CCATCTATGTACTGAACCCGGGACTAGAAGGAAAATAAATGATCTATATGTTGTGTGGA
>NP_002809.2 proteasome activator complex subunit 2 [Homo sapiens]
MAKPCGVRLSGEARKQVEVFRQNLFQEAEEFLYRFLPQKIIYLNQLLQEDSLNVADLTSLRAPLDIPIPD
PPPKDDEMETDKQEKKEVHKCGFLPGNEKVLSLLALVKPEVWTLKEKCILVITWIQHLIPKIEDGNDFGV
AIQEKVLERVNAVKTKVEAFQTTISKYFSERGDAVAKASKETHVMDYRALVHERDEAAYGELRAMVLDLR
AFYAELYHIISSNLEKIVNPKGEEKPSMY
```

```
i0_HQ_samplec9816d|c30843/f3p5/855      ------------------------------------------GAGACCAGAGATCTAGC 17
i0_HQ_samplec9816d|c31918/f2p0/772      ------------------------------------------------------------ 0
i0_HQ_samplec9816d|c91455/f11p9/956     ctggacctggggagtgaaagcgaaagcccgggcgactagccGGGAGACCAGAGATCTAGC 60
NM_002818.2                             -------TGGGGAGTGAAAGCGAAAGCCCGGGCGACTAGCCGGGAGACCAGAGATCTAGC 53
```

```
i0_HQ_samplec9816d|c30843/f3p5/855      GACTGAAGCAGCATGGCCAAGCCGTGTGGGGTGCGCCTGAGCGGGGAAGCCCGCAAACAG 77
i0_HQ_samplec9816d|c31918/f2p0/772      --tgagagcagcatggccaagccgtgtggggtgcgcctgagcggg--------------- 43
i0_HQ_samplec9816d|c91455/f11p9/956     GACTGAAGCAGCATGGCCAAGCCGTGTGGGGTGCGCCTGAGCGGGGAAGCCCGCAAACAG 120
NM_002818.2                             GACTGAAGCAGCATGGCCAAGCCGTGTGGGGTGCGCCTGAGCGGGGAAGCCCGCAAACAG 113
                                              ***************************************
```

```
i0_HQ_samplec9816d|c30843/f3p5/855      GTGGAGGTCTTCAGGCAGAATCTTTTCCAATTTATTTCATTTGCAGTTGGAGGTTCCT-- 135
i0_HQ_samplec9816d|c31918/f2p0/772      ------------------------------------------------------------ 43
i0_HQ_samplec9816d|c91455/f11p9/956     GTGGAGGTCTTCAGGCAGAATCTTTTCCAGGAGGTCTTCAGTTTCCTCATTTGCAAGTCT 180
NM_002818.2                             GTGGAGGTCTTCAGACAGAAT--------------------------------------- 134
```

```
i0_HQ_samplec9816d|c30843/f3p5/855      ---------------------------------------------------------TCA 138
i0_HQ_samplec9816d|c31918/f2p0/772      --------------------------------------------------gaagcccgcaa 54
i0_HQ_samplec9816d|c91455/f11p9/956     TGGACCCAGTGATTTCCAAAGGCCCTTCCAGTTCCATTTGTCAGACATCTCATTGAGCAT 240
NM_002818.2                             ------------------------------------------------------------ 134
```

```
i0_HQ_samplec9816d|c30843/f3p5/855      GTTCATTTCAGAATCCAGAATTCTTTTCCCAGAACAGGCTGAGGAATTCCTCTACAGATT 198
i0_HQ_samplec9816d|c31918/f2p0/772      acaggtggAGGTCTTCAGGCAGAATCTTTTCCAGGAGGCTGAGGAATTCCTCTACAGATT 114
i0_HQ_samplec9816d|c91455/f11p9/956     ATTCTGTGATTCCCCTCCCTTGCAATGTAACCCACAGGCTGAGGAATTCCTCTACAGATT 300
NM_002818.2                             -------------------------CTTTTCCAGGAGGCTGAGGAATTCCTCTACAGATT 169
                                                                       *************************
```

```
i0_HQ_samplec9816d|c30843/f3p5/855      CTTGCCACAGAAAATCATATACCTGAATCAGCTCTTGCAAGAGGACTCCCTCAATGTGGC 258
i0_HQ_samplec9816d|c31918/f2p0/772      CTTGCCACAGAAAATCATATACCTGAATCAGCTCTTGCAAGAGGACTCCCTCAATGTGGC 174
i0_HQ_samplec9816d|c91455/f11p9/956     CTTGCCACAGAAAATCATATACCTGAATCAGCTCTTGCAAGAGGACTCCCTCAATGTGGC 360
NM_002818.2                             CTTGCCACAGAAAATCATATACCTGAATCAGCTCTTGCAAGAGGACTCCCTCAATGTGGC 229
                                        ************************************************************
```

```
i0_HQ_samplec9816d|c30843/f3p5/855      TGACTTGACTTCCCTCCGGGCCCCACTGGACATCCCCATCCCAGACCCTCCACCCAAGGA 318
```

```
i0_HQ_samplec9816d|c31918/f2p0/772      TGACTTGACTTCCCTCCGGGCCCCACTGGACATCCCCATCCCAGACCCTCCACCCAAGGA 234
i0_HQ_samplec9816d|c91455/f11p9/956     TGACTTGACTTCCCTCCGGGCCCCACTGGACATCCCCATCCCAGACCCTCCACCCAAGGA 420
NM_002818.2                             TGACTTGACTTCCCTCCGGGCCCCACTGGACATCCCCATCCCAGACCCTCCACCCAAGGA 289
                                        ************************************************************

i0_HQ_samplec9816d|c30843/f3p5/855      TGATGAGATGGAAACAGATAAGCAGGAGAAGAAAGAAGTCCCTAAGTGTGGATTTCTCCC 378
i0_HQ_samplec9816d|c31918/f2p0/772      TGATGAGATGGAAACAGATAAGCAGGAGAAGAAAGAAGTCCCTAAGTGTGGATTTCTCCC 294
i0_HQ_samplec9816d|c91455/f11p9/956     TGATGAGATGGAAACAGATAAGCAGGAGAAGAAAGAAGTCCCTAAGTGTGGATTTCTCCC 480
NM_002818.2                             TGATGAGATGGAAACAGATAAGCAGGAGAAGAAAGAAGTCCATAAGTGTGGATTTCTCCC 349
                                        ***************************************** ******************

i0_HQ_samplec9816d|c30843/f3p5/855      TGGGAATGAGAAAGTCCTGTCCCTGCTTGCCCTGGTTAAGCCAGAAGTCTGGACTCTCAA 438
i0_HQ_samplec9816d|c31918/f2p0/772      TGGGAATGAGAAAGTCCTGTCCCTGCTTGCCCTGGTTAAGCCAGAAGTCTGGACTCTCAA 354
i0_HQ_samplec9816d|c91455/f11p9/956     TGGGAATGAGAAAGTCCTGTCCCTGCTTGCCCTGGTTAAGCCAGAAGTCTGGACTCTCAA 540
NM_002818.2                             TGGGAATGAGAAAGTCCTGTCCCTGCTTGCCCTGGTTAAGCCAGAAGTCTGGACTCTCAA 409
                                        ************************************************************

i0_HQ_samplec9816d|c30843/f3p5/855      AGAGAAATGCATTCTGGTGATTACATGGATCCAACACCTGATCCCCAAGATTGAAGATGG 498
i0_HQ_samplec9816d|c31918/f2p0/772      AGAGAAATGCATTCTGGTGATTACATGGATCCAACACCTGATCCCCAAGATTGAAGATGG 414
i0_HQ_samplec9816d|c91455/f11p9/956     AGAGAAATGCATTCTGGTGATTACATGGATCCAACACCTGATCCCCAAGATTGAAGATGG 600
NM_002818.2                             AGAGAAATGCATTCTGGTGATTACATGGATCCAACACCTGATCCCCAAGATTGAAGATGG 469
                                        ************************************************************

i0_HQ_samplec9816d|c30843/f3p5/855      AAATGATTT-TGGGGTAGCAATCCAGGAGAAGGTGCTGGAGAGGGTGAATGCCGTCAAGA 557
i0_HQ_samplec9816d|c31918/f2p0/772      AAATGATTTTTGGGGTAGCAATCCAGGAGAAGGTGCTGGAGAGGGTGAATGCCGTCAAGA 474
i0_HQ_samplec9816d|c91455/f11p9/956     AAATGATTT-TGGGGTAGCAATCCAGGAGAAGGTGCTGGAGAGGGTGAATGCCGTCAAGA 659
NM_002818.2                             AAATGATTT-TGGGGTAGCAATCCAGGAGAAGGTGCTGGAGAGGGTGAATGCCGTCAAGA 528
                                        ********* **************************************************

i0_HQ_samplec9816d|c30843/f3p5/855      CCAAAGTGGAAGCTTTCCAGACAACCATTTCCAAGTACTTCTCAGAACGTGGGGATGCTG 617
i0_HQ_samplec9816d|c31918/f2p0/772      CCAAAGTGGAAGCTTTCCAGACAACCATTTCCAAGTACTTCTCAGAACGTGGGGATGCTG 534
i0_HQ_samplec9816d|c91455/f11p9/956     CCAAAGTGGAAGCTTTCCAGACAACCATTTCCAAGTACTTCTCAGAACGTGGGGATGCTG 719
NM_002818.2                             CCAAAGTGGAAGCTTTCCAGACAACCATTTCCAAGTACTTCTCAGAACGTGGGGATGCTG 588
                                        ************************************************************

i0_HQ_samplec9816d|c30843/f3p5/855      TGGCCAAGGCCTCCAAGGAGACTCATGTAATGGATTACCGGGCCTTGGTGCATGAGCGAG 677
i0_HQ_samplec9816d|c31918/f2p0/772      TGGCCAAGGCCTCCAAGGAGACTCATGTAATGGATTACCGGGCCTTGGTGCATGAGCGAG 594
i0_HQ_samplec9816d|c91455/f11p9/956     TGGCCAAGGCCTCCAAGGAGACTCATGTAATGGATTACCGGGCCTTGGTGCATGAGCGAG 779
NM_002818.2                             TGGCCAAGGCCTCCAAGGAGACTCATGTAATGGATTACCGGGCCTTGGTGCATGAGCGAG 648
                                        ************************************************************

i0_HQ_samplec9816d|c30843/f3p5/855      ATGAGGCAGCCTATGGGGAGCTCAGGGCCATGGTGCTGGACCTGAGGGCCTTCTATGCTG 737
i0_HQ_samplec9816d|c31918/f2p0/772      ATGAGGCAGCCTATGGGGAGCTCAGGGCCATGGTGCTGGACCTGAGGGCCTTCTATGCTG 654
i0_HQ_samplec9816d|c91455/f11p9/956     ATGAGGCAGCCTATGGGGAGCTCAGGGCCATGGTGCTGGACCTGAGGGCCTTCTATGCTG 839
NM_002818.2                             ATGAGGCAGCCTATGGGGAGCTCAGGGCCATGGTGCTGGACCTGAGGGCCTTCTATGCTG 708
                                        ************************************************************

i0_HQ_samplec9816d|c30843/f3p5/855      AGCTTTATCATATCATCAGCAGCAACCTGGAGAAAATTGTCAACCCAAAGGGTGAAGAAA 797
i0_HQ_samplec9816d|c31918/f2p0/772      AGCTTTATCATATCATCAGCAGCAACCTGGAGAAAATTGTCAACCCAAAGGGTGAAGAAA 714
i0_HQ_samplec9816d|c91455/f11p9/956     AGCTTTATCATATCATCAGCAGCAACCTGGAGAAAATTGTCAACCCAAAGGGTGAAGAAA 899
NM_002818.2                             AGCTTTATCATATCATCAGCAGCAACCTGGAGAAAATTGTCAACCCAAAGGGTGAAGAAA 768
                                        ************************************************************

i0_HQ_samplec9816d|c30843/f3p5/855      AGCCATCTATGTACTGAACCCGGGACTAGAAggaaaataaatgatctatatgttgtgt-- 855
i0_HQ_samplec9816d|c31918/f2p0/772      AGCCATCTATGTACTGAACCCGGGACTAGAAGGAAAATAactgatctatatgttgtgt-- 772
i0_HQ_samplec9816d|c91455/f11p9/956     AGCCATCTATGTACTGAACCCGGGACTAGAAGGAAAATAAATGATCTATATGTTGTG--- 956
NM_002818.2                             AGCCATCTATGTACTGAACCCGGGACTAGAAGGAAAATAAATGATCTATATGTTGTGTGG 828
                                        **************************************** ****************

i0_HQ_samplec9816d|c30843/f3p5/855      -          855
i0_HQ_samplec9816d|c31918/f2p0/772      -          772
i0_HQ_samplec9816d|c91455/f11p9/956     -          956
NM_002818.2                             A          829
```

```
VIRT25459           MAKPCGVRLSGEARKQVEVFRQNLFQEAEEFLYRFLPQKIIYLNQLLQEDSLNVADLTSL     60
VIRT18646           ------------------------------------------------------------     0
VIRT19198           ------------------------------------------------------------     0
NP_002809.2         MAKPCGVRLSGEARKQVEVFRQNLFQEAEEFLYRFLPQKIIYLNQLLQEDSLNVADLTSL     60

VIRT25459           RAPLDIPIPDPPPKDDEMETDKQEKKEVPKCGFLPGNEKVLSLLALVKPEVWTLKEKCIL     120
VIRT18646           -----------------METDKQEKKEVPKCGFLPGNEKVLSLLALVKPEVWTLKEKCIL     43
VIRT19198           -----------------METDKQEKKEVPKCGFLPGNEKVLSLLALVKPEVWTLKEKCIL     43
NP_002809.2         RAPLDIPIPDPPPKDDEMETDKQEKKEVHKCGFLPGNEKVLSLLALVKPEVWTLKEKCIL     120
                                     *********** *******************************
```

```
VIRT25459        VITWIQHLIPKIEDGNDFWGSNPGEGAGEGECRQDQSGSFPDNHFQVLLRT-WGCCGQGL     179
VIRT18646        VITWIQHLIPKIEDGNDFGVAIQEKVLERVNAVKTKVEA-----FQTTISKYFSERGDAV     98
VIRT19198        VITWIQHLIPKIEDGNDFGVAIQEKVLERVNAVKTKVEA-----FQTTISKYFSERGDAV     98
NP_002809.2      VITWIQHLIPKIEDGNDFGVAIQEKVLERVNAVKTKVEA-----FQTTISKYFSERGDAV     175
                 ******************  :    :    . :. : :   :      **. :  . :.   *:.:

VIRT25459        QGDSC----NGLP---------GLGA-----------------------------------     192
VIRT18646        AKASKETHVMDYRALVHERDEAAYGELRAMVLDLRAFYAELYHIISSNLEKIVNPKGEEK     158
VIRT19198        AKASKETHVMDYRALVHERDEAAYGELRAMVLDLRAFYAELYHIISSNLEKIVNPKGEEK     158
NP_002809.2      AKASKETHVMDYRALVHERDEAAYGELRAMVLDLRAFYAELYHIISSNLEKIVNPKGEEK     235
                    *      .           . *

VIRT25459        ----           192
VIRT18646        PSMY           162
VIRT19198        PSMY           162
NP_002809.2      PSMY           239
```

**IL37**

| **Sequence** | **Header** | **AA Seq** |
|---|---|---|
| **Present** | i0_HQ_samplec9816d\|c22741/f2p0/859 | |
| | | |
| **Absent** | | |
| **NCBI** | NM_173204 | |
| | NM_173202 | |
| | NM_173205 | |

>i0_HQ_samplec9816d|c22741/f2p0/859
isoform=c22741;full_length_coverage=2;non_full_length_coverage=0;isoform_length=859

aaagtaaaggatcctgagaactgaaggcaaacagagctccaggagtccaagacagagccacagACCACGAGGATCCCTGGCCCAGGTCTTG
GACTTCATTCCATTTTCTGTTGAGTAATAAACTCAACGTTGAAAATGTCCTTTGTGGGGGAGAACTCAGGAGTGAAAAT
GGGCTCTGAGGACTGGGAAAAAGATGAACCCCAGTGCTGCTTAGAAGACCCGGCTGTAAGCCCCCTGGAACCAGGCCC
AAGCCTCCCCGCCATGAATTTTGTTCACACAAGTCCAAAGGTGAAGAACTTAAACCCGAAGAAATTCAGCATTCATGAC
CAGGATCACAAAGTACTGGTCCTGGACTCTGGGAATCTCATAGCAGTTCCAGATAAAAACTACATACGCCCAGAGATCT
TCTTTGCATTAGCCTCATCCTTGAGCTCAGCCTCTGCGGAGAAAGGAAGTCCGATTCTCCTGGGGGTCTCTAAAGGGGA
GTTTTGTCTCTACTGTGACAAGGATAAAGGACAAAGTCATCCATCCCTTCAGCTGAAGAAGGAGAAACTGATGAAGCTG
GCTGCCCAAAAGGAATCAGCACGCCGGCCCTTCATCTTTTATAGGGCTCAGGTGGGCTCCTGGAACATGCTGGAGTCG
GCGGCTCACCCCGGATGGTTCATCTGCACCTCCTGCAATTGTAATGAGCCTGTTGGGGTGACAGATAAATTTGAGAACA
GGAAACACATTGAATTTTCATTTCAACCAGTTTGCAAAGCTGAAATGAGCCCCAGTGAGGTCAGCGATTAGGAAACTGC
CCCATTGAACGCCTTCCTCGCTAatttgaactaattgtataaaaacaccaaacctgctc

>VIRT93267

MAGRLGPGSRGLTAGSSKQHWGSSFSQSSEPIFTPEFSPTKDIFNVEFITQQKME

```
>NM_173204.1 Homo sapiens interleukin 37 (IL37), transcript variant 4, mRNA
CTTCATTCCATTTTCTGTTGAGTAATAAACTCAACGTTGAAAATGTCCTTTGTGGGGGAGAACTCAGGAG
TGAAAATGGGCTCTGAGGACTGGGAAAAAGATGAACCCCAGTGCTGCTTAGAAGACCCGGCTGGAAGCCC
CCTGGAACCAGGCCCAAGCCTCCCCACCATGAATTTTGTTCACACAAAGATCTTCTTTGCATTAGCCTCA
TCCTTGAGCTCAGCCTCTGCGGAGAAAGGAAGTCCGATTCTCCTGGGGGTCTCTAAAGGGGAGTTTTGTC
TCTACTGTGACAAGGATAAAGGACAAAGTCATCCATCCCTTCAGCTGAAGAAGGAGAAACTGATGAAGCT
GGCTGCCCAAAAGGAATCAGCACGCCGGCCCTTCATCTTTTATAGGGCTCAGGTGGGCTCCTGGAACATG
CTGGAGTCGGCGGCTCACCCCGGATGGTTCATCTGCACCTCCTGCAATTGTAATGAGCCTGTTGGGGTGA
CAGATAAATTTGAGAACAGGAAACACATTGAATTTTCATTTCAACCAGTTTGCAAAGCTGAAATGAGCCC
CAGTGAGGTCAGCGATTAGGAAACTGCCCCATTGAACGCCTTCCTCGCTAATTTGAACTAATTGTATAAA
AACACCAAACCTGCTCACTAAAAAAAAAAAAAAAAAAA
>NP_775296.1 interleukin-37 isoform 4 [Homo sapiens]
MSFVGENSGVKMGSEDWEKDEPQCCLEDPAGSPLEPGPSLPTMNFVHTKIFFALASSLSSASAEKGSPIL
LGVSKGEFCLYCDKDKGQSHPSLQLKKEKLMKLAAQKESARRPFIFYRAQVGSWNMLESAAHPGWFICTS
```

```
CNCNEPVGVTDKFENRKHIEFSFQPVCKAEMSPSEVSD
>NM_173202.1 Homo sapiens interleukin 37 (IL37), transcript variant 2, mRNA
CTTCATTCCATTTTCTGTTGAGTAATAAACTCAACGTTGAAAATGTCCTTTGTGGGGGAGAACTCAGGAG
TGAAAATGGGCTCTGAGGACTGGGAAAAAGATGAACCCCAGTGCTGCTTAGAAGGTCCAAAGGTGAAGAA
CTTAAACCCGAAGAAATTCAGCATTCATGACCAGGATCACAAAGTACTGGTCCTGGACTCTGGGAATCTC
ATAGCAGTTCCAGATAAAAACTACATACGCCCAGAGATCTTCTTTGCATTAGCCTCATCCTTGAGCTCAG
CCTCTGCGGAGAAAGGAAGTCCGATTCTCCTGGGGGTCTCTAAAGGGGAGTTTTGTCTCTACTGTGACAA
GGATAAAGGACAAAGTCATCCATCCCTTCAGCTGAAGAAGGAGAAACTGATGAAGCTGGCTGCCCAAAAG
GAATCAGCACGCCGGCCCTTCATCTTTTATAGGGCTCAGGTGGGCTCCTGGAACATGCTGGAGTCGGCGG
CTCACCCCGGATGGTTCATCTGCACCTCCTGCAATTGTAATGAGCCTGTTGGGGTGACAGATAAATTTGA
GAACAGGAAACACATTGAATTTTCATTTCAACCAGTTTGCAAAGCTGAAATGAGCCCCAGTGAGGTCAGC
GATTAGGAAACTGCCCCATTGAACGCCTTCCTCGCTAATTTGAACTAATTGTATAAAAACACCAAACCTG
CTCACTAAAAAAAAAAAAAAAAAA
>NP_775294.1 interleukin-37 isoform 2 [Homo sapiens]
MSFVGENSGVKMGSEDWEKDEPQCCLEGPKVKNLNPKKFSIHDQDHKVLVLDSGNLIAVPDKNYIRPEIF
FALASSLSSASAEKGSPILLGVSKGEFCLYCDKDKGQSHPSLQLKKEKLMKLAAQKESARRPFIFYRAQV
GSWNMLESAAHPGWFICTSCNCNEPVGVTDKFENRKHIEFSFQPVCKAEMSPSEVSD
>NM_173205.1 Homo sapiens interleukin 37 (IL37), transcript variant 5, mRNA
ATGTCAGGCTGTGATAGGAGGGAAACAGAAACCAAAGGAAAGAACAGCTTTAAGAAGCGCTTAAGAGGTC
CAAAGGTGAAGAACTTAAACCCGAAGAAATTCAGCATTCATGACCAGGATCACAAAGTACTGGTCCTGGA
CTCTGGGAATCTCATAGCAGTTCCAGATAAAAACTACATACGCCCAGAGATCTTCTTTGCATTAGCCTCA
TCCTTGAGCTCAGCCTCTGCGGAGAAAGGAAGTCCGATTCTCCTGGGGGTCTCTAAAGGGGAGTTTTGTC
TCTACTGTGACAAGGATAAAGGACAAAGTCATCCATCCCTTCAGCTGAAGAAGGAGAAACTGATGAAGCT
GGCTGCCCAAAAGGAATCAGCACGCCGGCCCTTCATCTTTTATAGGGCTCAGGTGGGCTCCTGGAACATG
CTGGAGTCGGCGGCTCACCCCGGATGGTTCATCTGCACCTCCTGCAATTGTAATGAGCCTGTTGGGGTGA
CAGATAAATTTGAGAACAGGAAACACATTGAATTTTCATTTCAACCAGTTTGCAAAGCTGAAATGAGCCC
CAGTGAGGTCAGCGATTAGGAAACTGCCCCATTGAACGCCTTCCTCGCTAATTTGAACTAATTGTATAAA
AACACCAAACCTGCTCACTAAAAAAAAAAAAAAAAAA
>NP_775297.1 interleukin-37 isoform 5 [Homo sapiens]
MSGCDRRETETKGKNSFKKRLRGPKVKNLNPKKFSIHDQDHKVLVLDSGNLIAVPDKNYIRPEIFFALAS
SLSSASAEKGSPILLGVSKGEFCLYCDKDKGQSHPSLQLKKEKLMKLAAQKESARRPFIFYRAQVGSWNM
LESAAHPGWFICTSCNCNEPVGVTDKFENRKHIEFSFQPVCKAEMSPSEVSD
```

```
NM_173205.1                          ------------------------------------------------------------      0
NM_173204.1                          ------------------------------------------------------------      0
i0_HQ_samplec9816d|c22741/f2p0/859   aaagtaaaggatcctgagaactgaaggcaaacagagctccaggagtccaagacagagcca      60
NM_173202.1                          ------------------------------------------------------------      0

NM_173205.1                          ------------------------------------------------------------      0
NM_173204.1                          ---------------------------------CTTCATTCCATTTTCTGTTGAGTAATA      27
i0_HQ_samplec9816d|c22741/f2p0/859   cagACCACGAGGATCCCTGGCCCAGGTCTTGGACTTCATTCCATTTTCTGTTGAGTAATA      120
NM_173202.1                          ---------------------------------CTTCATTCCATTTTCTGTTGAGTAATA      27

NM_173205.1                          ------------------------------------------------------------      0
NM_173204.1                          AACTCAACGTTGAAAATGTCCTTTGTGGGGGAGAACTCAGGAGTGAAAATGGGCTCTGAG      87
i0_HQ_samplec9816d|c22741/f2p0/859   AACTCAACGTTGAAAATGTCCTTTGTGGGGGAGAACTCAGGAGTGAAAATGGGCTCTGAG      180
NM_173202.1                          AACTCAACGTTGAAAATGTCCTTTGTGGGGGAGAACTCAGGAGTGAAAATGGGCTCTGAG      87

NM_173205.1                          -----------------------------------ATGTCAGGCTGTGATAGGAGGGAAA      25
NM_173204.1                          GACTGGGAAAAAGATGAACCCCAGTGCTGCTTAGAAGACCCGGCTGGAAGCC--CCCTGG      145
i0_HQ_samplec9816d|c22741/f2p0/859   GACTGGGAAAAAGATGAACCCCAGTGCTGCTTAGAAGACCCGGCTGTAAGCC--CCCTGG      238
NM_173202.1                          GACTGGGAAAAAGATGAACCCCAGTGCTGCTTAGAAGG----------------------      125
                                                                        *

NM_173205.1                          CAGAAACCAAAGGAAAGAACAGCTTTAAGAAGCGCTTAAGAGGTCCAAAGGTGAAGAACT      85
NM_173204.1                          AACCAGGCCCAAGCCTCCCCACCATGAATTTTGTTC------------------------      181
i0_HQ_samplec9816d|c22741/f2p0/859   AACCAGGCCCAAGCCTCCCCGCCATGAATTTTGTTCACACAAGTCCAAAGGTGAAGAACT      298
NM_173202.1                          --------------------------------------------TCCAAAGGTGAAGAACT      142

NM_173205.1                          TAAACCCGAAGAAATTCAGCATTCATGACCAGGATCACAAAGTACTGGTCCTGGACTCTG      145
NM_173204.1                          ------------------------------------------------------------      181
i0_HQ_samplec9816d|c22741/f2p0/859   TAAACCCGAAGAAATTCAGCATTCATGACCAGGATCACAAAGTACTGGTCCTGGACTCTG      358
NM_173202.1                          TAAACCCGAAGAAATTCAGCATTCATGACCAGGATCACAAAGTACTGGTCCTGGACTCTG      202

NM_173205.1                          GGAATCTCATAGCAGTTCCAGATAAAAACTACATACGCCCAGAGATCTTCTTTGCATTAG      205
NM_173204.1                          ------------------------------------ACACAAAGATCTTCTTTGCATTAG      205
i0_HQ_samplec9816d|c22741/f2p0/859   GGAATCTCATAGCAGTTCCAGATAAAAACTACATACGCCCAGAGATCTTCTTTGCATTAG      418
NM_173202.1                          GGAATCTCATAGCAGTTCCAGATAAAAACTACATACGCCCAGAGATCTTCTTTGCATTAG      262
                                                                          * ** ******************

NM_173205.1                          CCTCATCCTTGAGCTCAGCCTCTGCGGAGAAAGGAAGTCCGATTCTCCTGGGGGTCTCTA      265
NM_173204.1                          CCTCATCCTTGAGCTCAGCCTCTGCGGAGAAAGGAAGTCCGATTCTCCTGGGGGTCTCTA      265
i0_HQ_samplec9816d|c22741/f2p0/859   CCTCATCCTTGAGCTCAGCCTCTGCGGAGAAAGGAAGTCCGATTCTCCTGGGGGTCTCTA      478
NM_173202.1                          CCTCATCCTTGAGCTCAGCCTCTGCGGAGAAAGGAAGTCCGATTCTCCTGGGGGTCTCTA      322
                                     ************************************************************

NM_173205.1                          AAGGGGAGTTTTGTCTCTACTGTGACAAGGATAAAGGACAAAGTCATCCATCCCTTCAGC      325
NM_173204.1                          AAGGGGAGTTTTGTCTCTACTGTGACAAGGATAAAGGACAAAGTCATCCATCCCTTCAGC      325
i0_HQ_samplec9816d|c22741/f2p0/859   AAGGGGAGTTTTGTCTCTACTGTGACAAGGATAAAGGACAAAGTCATCCATCCCTTCAGC      538
NM_173202.1                          AAGGGGAGTTTTGTCTCTACTGTGACAAGGATAAAGGACAAAGTCATCCATCCCTTCAGC      382
                                     ************************************************************
```

```
NM_173205.1                       TGAAGAAGGAGAAACTGATGAAGCTGGCTGCCCAAAAGGAATCAGCACGCCGGCCCTTCA     385
NM_173204.1                       TGAAGAAGGAGAAACTGATGAAGCTGGCTGCCCAAAAGGAATCAGCACGCCGGCCCTTCA     385
i0_HQ_samplec9816d|c22741/f2p0/859 TGAAGAAGGAGAAACTGATGAAGCTGGCTGCCCAAAAGGAATCAGCACGCCGGCCCTTCA     598
NM_173202.1                       TGAAGAAGGAGAAACTGATGAAGCTGGCTGCCCAAAAGGAATCAGCACGCCGGCCCTTCA     442
                                  ************************************************************

NM_173205.1                       TCTTTTATAGGGCTCAGGTGGGCTCCTGGAACATGCTGGAGTCGGCGGCTCACCCCGGAT     445
NM_173204.1                       TCTTTTATAGGGCTCAGGTGGGCTCCTGGAACATGCTGGAGTCGGCGGCTCACCCCGGAT     445
i0_HQ_samplec9816d|c22741/f2p0/859 TCTTTTATAGGGCTCAGGTGGGCTCCTGGAACATGCTGGAGTCGGCGGCTCACCCCGGAT     658
NM_173202.1                       TCTTTTATAGGGCTCAGGTGGGCTCCTGGAACATGCTGGAGTCGGCGGCTCACCCCGGAT     502
                                  ************************************************************

NM_173205.1                       GGTTCATCTGCACCTCCTGCAATTGTAATGAGCCTGTTGGGGTGACAGATAAATTTGAGA     505
NM_173204.1                       GGTTCATCTGCACCTCCTGCAATTGTAATGAGCCTGTTGGGGTGACAGATAAATTTGAGA     505
i0_HQ_samplec9816d|c22741/f2p0/859 GGTTCATCTGCACCTCCTGCAATTGTAATGAGCCTGTTGGGGTGACAGATAAATTTGAGA     718
NM_173202.1                       GGTTCATCTGCACCTCCTGCAATTGTAATGAGCCTGTTGGGGTGACAGATAAATTTGAGA     562
                                  ************************************************************

NM_173205.1                       ACAGGAAACACATTGAATTTTCATTTCAACCAGTTTGCAAAGCTGAAATGAGCCCCAGTG     565
NM_173204.1                       ACAGGAAACACATTGAATTTTCATTTCAACCAGTTTGCAAAGCTGAAATGAGCCCCAGTG     565
i0_HQ_samplec9816d|c22741/f2p0/859 ACAGGAAACACATTGAATTTTCATTTCAACCAGTTTGCAAAGCTGAAATGAGCCCCAGTG     778
NM_173202.1                       ACAGGAAACACATTGAATTTTCATTTCAACCAGTTTGCAAAGCTGAAATGAGCCCCAGTG     622
                                  ************************************************************

NM_173205.1                       AGGTCAGCGATTAGGAAACTGCCCCATTGAACGCCTTCCTCGCTAATTTGAACTAATTGT     625
NM_173204.1                       AGGTCAGCGATTAGGAAACTGCCCCATTGAACGCCTTCCTCGCTAATTTGAACTAATTGT     625
i0_HQ_samplec9816d|c22741/f2p0/859 AGGTCAGCGATTAGGAAACTGCCCCATTGAACGCCTTCCTCGCTAatttgaactaattgt     838
NM_173202.1                       AGGTCAGCGATTAGGAAACTGCCCCATTGAACGCCTTCCTCGCTAATTTGAACTAATTGT     682
                                  ************************************************************

NM_173205.1                       ATAAAAACACCAAACCTGCTCACTAAAAAAAAAAAAAAAAAA 667
NM_173204.1                       ATAAAAACACCAAACCTGCTCACTAAAAAAAAAAAAAAAAAA 667
i0_HQ_samplec9816d|c22741/f2p0/859 ataaaaacaccaaacctgctc--------------------- 859
NM_173202.1                       ATAAAAACACCAAACCTGCTCACTAAAAAAAAAAAAAAAAAA 724
                                  *********************
```

```
VIRT93267       -----------------------------MAGRLGP------------------------     7
NP_775296.1     MSFVGENSGVKMGSEDWEKDEPQCCLEDPAGSPLEPGP-------------------SLP     41
NP_775294.1     MSFVGENSGVKMGSEDWEKDEPQCCLEGPKVKNLNPKKFSIHDQDHKVLVLDSGNLIAVP     60
NP_775297.1     MSGCD-----RRETETKGKNSFKKRLRGPKVKNLNPKKFSIHDQDHKVLVLDSGNLIAVP     55
                                                  * *

VIRT93267       ------------------------------------------------------------     7
NP_775296.1     TMNFVHTKIFFALASSLSSASAEKGSPILLGVSKGEFCLYCDKDKGQSHPSLQLKKEKLM     101
NP_775294.1     DKNYIRPEIFFALASSLSSASAEKGSPILLGVSKGEFCLYCDKDKGQSHPSLQLKKEKLM     120
NP_775297.1     DKNYIRPEIFFALASSLSSASAEKGSPILLGVSKGEFCLYCDKDKGQSHPSLQLKKEKLM     115

VIRT93267       --------------------GSRGLTAGSSKQHWGSSFS--------------------Q     27
NP_775296.1     KLAAQKESARRPFIFYRAQVGSWNMLESAAHPGWFICTSCNCNEPVGVTDKFENRKHIEF     161
NP_775294.1     KLAAQKESARRPFIFYRAQVGSWNMLESAAHPGWFICTSCNCNEPVGVTDKFENRKHIEF     180
NP_775297.1     KLAAQKESARRPFIFYRAQVGSWNMLESAAHPGWFICTSCNCNEPVGVTDKFENRKHIEF     175
                                    ** .:  .:::  *   . *

VIRT93267       SSEPIFTPEFSPTKDIFNVEFITQQKME  55
NP_775296.1     SFQPVCKAEMSPSEVSD-----------  178
NP_775294.1     SFQPVCKAEMSPSEVSD-----------  197
NP_775297.1     SFQPVCKAEMSPSEVSD-----------  192
                * :*: . *:**::
```

## CGB8

| **Sequence** | **Header** | **AA Seq** |
|---|---|---|
| **Present** | i0_HQ_samplec9816d\|c13287/f2p2/890 | |
| | | |
| **Absent** | | |
| **NCBI** | NM_033183 | |
| | | |
| | | |

>i0_HQ_samplec9816d|c13287/f2p2/890 isoform=c13287;full_length_coverage=2;non_full_length_coverage=2;isoform_length=890

actttgggtgtgctcgaggtggaggtgttgggcgaggtgcctgcagctggcggcagttcCCTCTGCCAGCACTTCTTTGTCACCGCTTCGAGGC
CGATAACTCTGAGGAAGGCTCGACTAGTCCCTAGCACTCGACGACTGAGTCTCTGAGGTCACTTCACCGTGGTCTCCGC

CTCACCCTTGGCGCTGGACCAGTGAGAGGAGAGGGCTGGGGCGCTCCGCTGAGCCACTCCTGCGCCCCCCTGGCCTTG
TCTACCTCTTGCCCCCCGAAGGGTTAGTGTCGAGCTCACTCCAGCATCCTACAACCTCCTGGTGGCCTTGCCGCCCCCAC
AACCCCGAGGTTTAAAGCCAGGTACACGAGGCAGGGGACACACCAAGGATGGAGATGTTCCAGGGGCTGCTGCTGTT
GCTGCTGCTGAGCATGGGCGGGACATGGGCATCCAAGGAGCCGCTTCGGCCACGGTGCCGCCCCATCAATGCCACCCT
GGCTGTGGAGAAGGAGGGCTGCCCCGTGTGCATCACCGTCAACACCACCATCTGTGCCGGCTACTGCCCCACCATGACC
CGCGTGCTGCAGGGGGTCCTGCCGGCCCTGCCTCAGGTGGTGTGCAACTACCGCGATGTGCGCTTCGAGTCCATCCGG
CTCCCTGGCTGCCCGCGCGGCGTGAACCCCGTGGTCTCCTACGCCGTGGCTCTCAGCTGTCAATGTGCACTCTGCCGCC
GCAGCACCACTGACTGCGGGGGTCCCAAGGACCACCCCTTGACCTGTGATGACCCCGCTTCCAGGACTCCTCTTCCTC
AAAGGCCCCTCCCCCCAGCCTTCCAAGTCCATCCCGACTCCCGGGGCCCTCGGacaccccgatcctcccacaataaaggcttctcaat
cc

```
>VIRT171498
```

```
MEMFQGLLLLLLLSMGGTWASKEPLRPRCRPINATLAVEKEGCPVCITVNTTICAGYCPT
MTRVLQGVLPALPQVVCNYRDVRFESIRLPGCPRGVNPVVSYAVALSCQCALCRRSTTDC
GGPKDHPLTCDDPRFQDSSSSKAPPPSLPSPSRLPGPSDTPILPQ
```

```
>NM_033183.2 Homo sapiens chorionic gonadotropin subunit beta 8 (CGB8), mRNA
GCTTCAGTCCAGCACCTTTCTCGGGTCACGGCCTCCTCCTGGCTCCCAGGACCCCACCATAGGCAGAGGC
AGGCCTTCCTACACCCTACTCCCTGTGCCTCCAGGCTCGACTAGTCCCTAGCACTCGACGACTGAGTCTC
TGAGGTCACTTCACCGTGGTCTCCGCCTCACCCTTGGCGCTGGACCAGTGAGAGGAGAGGGCTGGGGCGC
TCCGCTGAGCCACTCCTGCGCCCCCCTGGCCTTGTCTACCTCTTGCCCCCCGAAGGGTTAGTGTCGAGCT
CACTCCAGCATCCTACAACCTCCTGGTGGCCTTGCCGCCCCCACAACCCCGAGGTTTAAAGCCAGGTACA
CGAGGCAGGGGACACACCAAGGATGGAGATGTTCCAGGGGCTGCTGCTGTTGCTGCTGCTGAGCATGGGC
GGGACATGGGCATCCAAGGAGCCGCTTCGGCCACGGTGCCGCCCCATCAATGCCACCCTGGCTGTGGAGA
AGGAGGGCTGCCCCGTGTGCATCACCGTCAACACCACCATCTGTGCCGGCTACTGCCCCACCATGACCCG
CGTGCTGCAGGGGGTCCTGCCGGCCCTGCCTCAGGTGGTGTGCAACTACCGCGATGTGCGCTTCGAGTCC
ATCCGGCTCCCTGGCTGCCCGCGCGGCGTGAACCCCGTGGTCTCCTACGCCGTGGCTCTCAGCTGTCAAT
GTGCACTCTGCCGCCGCAGCACCACTGACTGCGGGGGTCCCAAGGACCACCCCTTGACCTGTGATGACCC
CCGCTTCCAGGACTCCTCTTCCTCAAAGGCCCCTCCCCCCAGCCTTCCAAGTCCATCCCGACTCCCGGGG
CCCTCGGACACCCCGATCCTCCCACAATAAAGGCTTCTCAATCCGCAAAAAAAAAAAAAAAAAAA
>NP_149439.1 chorionic gonadotropin, beta polypeptide 8 precursor [Homo sapiens]
MEMFQGLLLLLLLSMGGTWASKEPLRPRCRPINATLAVEKEGCPVCITVNTTICAGYCPTMTRVLQGVLP
ALPQVVCNYRDVRFESIRLPGCPRGVNPVVSYAVALSCQCALCRRSTTDCGGPKDHPLTCDDPRFQDSSS
SKAPPPSLPSPSRLPGPSDTPILPQ
```

```
i0_HQ_samplec9816d|c13287/f2p2/890      acttgggtgtgctcgaggtggaggtgttgggcgaggtgcctgcagctggcggcagt-tcC  59
NM_033183.2                             GCTTCAGTC-----CAGC-AC-CTTTCTCGGGTCACGGCCTCCTCCTGGCTCCCAGGACC  53
                                         ***  **       **       *  * **      **** *  *****  *     **

i0_HQ_samplec9816d|c13287/f2p2/890      CTCTGCCAGCACTTCTTTGTCACCGCTTCGAGGCCGATAACTCTGAGGAAGGCTCGACTA  119
NM_033183.2                             CCACCATAGGCAGAGGCAGGCCTTCCTACACCCTACTCCCTGTGCCTCCAGGCTCGACTA  113
                                        *      **         * *    **  *                    ***********

i0_HQ_samplec9816d|c13287/f2p2/890      GTCCCTAGCACTCGACGACTGAGTCTCTGAGGTCACTTCACCGTGGTCTCCGCCTCACCC  179
NM_033183.2                             GTCCCTAGCACTCGACGACTGAGTCTCTGAGGTCACTTCACCGTGGTCTCCGCCTCACCC  173
                                        ************************************************************

i0_HQ_samplec9816d|c13287/f2p2/890      TTGGCGCTGGACCAGTGAGAGGAGAGGGCTGGGGCGCTCCGCTGAGCCACTCCTGCGCCC  239
NM_033183.2                             TTGGCGCTGGACCAGTGAGAGGAGAGGGCTGGGGCGCTCCGCTGAGCCACTCCTGCGCCC  233
                                        ************************************************************

i0_HQ_samplec9816d|c13287/f2p2/890      CCCTGGCCTTGTCTACCTCTTGCCCCCCGAAGGGTTAGTGTCGAGCTCACTCCAGCATCC  299
NM_033183.2                             CCCTGGCCTTGTCTACCTCTTGCCCCCCGAAGGGTTAGTGTCGAGCTCACTCCAGCATCC  293
                                        ************************************************************

i0_HQ_samplec9816d|c13287/f2p2/890      TACAACCTCCTGGTGGCCTTGCCGCCCCCACAACCCCGAGGTTTAAAGCCAGGTACACGA  359
NM_033183.2                             TACAACCTCCTGGTGGCCTTGCCGCCCCCACAACCCCGAGGTTTAAAGCCAGGTACACGA  353
                                        ************************************************************

i0_HQ_samplec9816d|c13287/f2p2/890      GGCAGGGGACACACCAAGGATGGAGATGTTCCAGGGGCTGCTGCTGTTGCTGCTGCTGAG  419
NM_033183.2                             GGCAGGGGACACACCAAGGATGGAGATGTTCCAGGGGCTGCTGCTGTTGCTGCTGCTGAG  413
                                        ************************************************************

i0_HQ_samplec9816d|c13287/f2p2/890      CATGGGCGGGACATGGGCATCCAAGGAGCCGCTTCGGCCACGGTGCCGCCCCATCAATGC  479
NM_033183.2                             CATGGGCGGGACATGGGCATCCAAGGAGCCGCTTCGGCCACGGTGCCGCCCCATCAATGC  473
                                        ************************************************************
```

```
i0_HQ_samplec9816d|c13287/f2p2/890      CACCCTGGCTGTGGAGAAGGAGGGCTGCCCCGTGTGCATCACCGTCAACACCACCATCTG  539
NM_033183.2                             CACCCTGGCTGTGGAGAAGGAGGGCTGCCCCGTGTGCATCACCGTCAACACCACCATCTG  533
                                        ************************************************************

i0_HQ_samplec9816d|c13287/f2p2/890      TGCCGGCTACTGCCCCACCATGACCCGCGTGCTGCAGGGGGTCCTGCCGGCCCTGCCTCA  599
NM_033183.2                             TGCCGGCTACTGCCCCACCATGACCCGCGTGCTGCAGGGGGTCCTGCCGGCCCTGCCTCA  593
                                        ************************************************************

i0_HQ_samplec9816d|c13287/f2p2/890      GGTGGTGTGCAACTACCGCGATGTGCGCTTCGAGTCCATCCGGCTCCCTGGCTGCCCGCG  659
NM_033183.2                             GGTGGTGTGCAACTACCGCGATGTGCGCTTCGAGTCCATCCGGCTCCCTGGCTGCCCGCG  653
                                        ************************************************************

i0_HQ_samplec9816d|c13287/f2p2/890      CGGCGTGAACCCCGTGGTCTCCTACGCCGTGGCTCTCAGCTGTCAATGTGCACTCTGCCG  719
NM_033183.2                             CGGCGTGAACCCCGTGGTCTCCTACGCCGTGGCTCTCAGCTGTCAATGTGCACTCTGCCG  713
                                        ************************************************************

i0_HQ_samplec9816d|c13287/f2p2/890      CCGCAGCACCACTGACTGCGGGGGTCCCAAGGACCACCCCTTGACCTGTGATGACCCCCG  779
NM_033183.2                             CCGCAGCACCACTGACTGCGGGGGTCCCAAGGACCACCCCTTGACCTGTGATGACCCCCG  773
                                        ************************************************************

i0_HQ_samplec9816d|c13287/f2p2/890      CTTCCAGGACTCCTCTTCCTCAAAGGCCCCTCCCCCCAGCCTTCCAAGTCCATCCCGACT  839
NM_033183.2                             CTTCCAGGACTCCTCTTCCTCAAAGGCCCCTCCCCCCAGCCTTCCAAGTCCATCCCGACT  833
                                        ************************************************************

i0_HQ_samplec9816d|c13287/f2p2/890      CCCGGGGCCCTCGGacaccccgatcctcccacaataaaggcttctcaatcc---------  890
NM_033183.2                             CCCGGGGCCCTCGGACACCCCGATCCTCCCACAATAAAGGCTTCTCAATCCGCAAAAAAA  893
                                        ***************************************************

i0_HQ_samplec9816d|c13287/f2p2/890      -----------          890
NM_033183.2                             AAAAAAAAAAA          904
```

```
VIRT171498       MEMFQGLLLLLLLSMGGTWASKEPLRPRCRPINATLAVEKEGCPVCITVNTTICAGYCPT       60
NP_149439.1      MEMFQGLLLLLLLSMGGTWASKEPLRPRCRPINATLAVEKEGCPVCITVNTTICAGYCPT       60
                 ************************************************************

VIRT171498       MTRVLQGVLPALPQVVCNYRDVRFESIRLPGCPRGVNPVVSYAVALSCQCALCRRSTTDC       120
NP_149439.1      MTRVLQGVLPALPQVVCNYRDVRFESIRLPGCPRGVNPVVSYAVALSCQCALCRRSTTDC       120
                 ************************************************************

VIRT171498       GGPKDHPLTCDDPRFQDSSSSKAPPPSLPSPSRLPGPSDTPILPQ           165
NP_149439.1      GGPKDHPLTCDDPRFQDSSSSKAPPPSLPSPSRLPGPSDTPILPQ           165
                 *********************************************
```

**RPS5**

| Sequence | Header | AA Seq |
|---|---|---|
| **Present** | i0_HQ_samplec9816d\|c29292/f2p0/819 | |
| | | |
| **Absent** | i0_HQ_samplec9816d\|c18100/f19p0/740 | |
| **NCBI** | NM_001009 | |
| | | |

>i0_HQ_samplec9816d|c29292/f2p0/819
isoform=c29292;full_length_coverage=2;non_full_length_coverage=0;isoform_length=819

acggcaacctggacctgcggagggcggcggtgaccactgggcctcactccgctaacccaggtccgcatCAGGCGCGTGCCGGTGAGGCGGCTG
TGGCCGCGGCGAGGAGTCAGAGGGAACTGAACTGGCTACTCCCGCGGCAGGTGGGATTTAAGGATGACCGGTGGGA
GACAGCAGCACCAGCGGTGGCAGAGACCCCAGACATCAAGCTCTTTGGGAAGTGGAGCACCGATGATGTGCAGATCA
ATGACATTTCCCTGCAGGATTACATTGCAGTGAAGGAGAAGTATGCCAAGTACCTGCCTCACAGTGCAGGGCGGTATG
CCGCCAAACGCTTCCGCAAAGCTCAGTGTCCCATTGTGGAGCGCCTCACTAACTCCATGATGATGCACGGCCGCAACAA
CGGCAAGAAGCTCATGACTGTGCGCATCGTCAAGCATGCCTTCGAGATCATACACCTGCTCACAGGCGAGAACCCTCTG
CAGGTCCTGGTGAACGCCATCATCAACAGTGGTCCCCGGGAGGACTCCACACGCATTGGGCGCGCCGGGACTGTGAGA
CGACAGGCTGTGGATGTGTCCCCCCTGCGCCGTGTGAACCAGGCCATCTGGCTGCTGTGCACAGGCGCTCGTGAGGCT
GCCTTCCGGAACATTAAGACCATTGCTGAGTGCCTGGCAGATGAGCTCATCAATGCTGCCAAGGGCTCCTCGAACTCCT

ATGCCATTAAGAAGAAGGACGAGCTGGAGCGTGTGGCCAAGTCCAACCGCTGATTTTCCCAGCTGCTGCCcaataaacctg tctgccctttggggcagcccc

>VIRT30957

MSSSARHSAMVLMFRKAASRAPVHSSQMAWFTRRRGDTSTACRLTVPARPMRVESSRGPL
LMMAFTRTCRGFSPVSRCMISKACLTMRTVMSFLPLLRPCIIMELVRRSTMGH

>i0_HQ_samplec9816d|c18100/f19p0/740 isoform=c18100;full_length_coverage=19;non_full_length_coverage=0;isoform_length=740

ctcttcctgtctgtaccagggcggcgcGTGGTCTACGCCGAGTGGACAGAGACGCTCAGGCTGTGTTCTCAGGATGACCGAGTGG
GAGACAGCAGCACCAGCGGTGGCAGAGACCCCAGACATCAAGCTCTTTGGGAAGTGGAGCACCGATGATGTGCAGAT
CAATGACATTTCCCTGCAGGATTACATTGCAGTGAAGGAGAAGTATGCCAAGTACCTGCCTCACAGTGCAGGGCGGTAT
GCCGCCAAACGCTTCCGCAAAGCTCAGTGTCCCATTGTGGAGCGCCTCACTAACTCCATGATGATGCACGGCCGCAACA
ACGGCAAGAAGCTCATGACTGTGCGCATCGTCAAGCATGCCTTCGAGATCATACACCTGCTCACAGGCGAGAACCCTCT
GCAGGTCCTGGTGAACGCCATCATCAACAGTGGTCCCCGGGAGGACTCCACACGCATTGGGCGCGCCGGGACTGTGAG
ACGACAGGCTGTGGATGTGTCCCCCCTGCGCCGTGTGAACCAGGCCATCTGGCTGCTGTGCACAGGCGCTCGTGAGGC
TGCCTTCCGGAACATTAAGACCATTGCTGAGTGCCTGGCAGATGAGCTCATCAATGCTGCCAAGGGCTCCTCGAACTCC
TATGCCATTAAGAAGAAGGACGAGCTGGAGCGTGTGGCCAAGTCCAACCGCTGATTTTCCCAGCTGCTGCCCAATAAAC
CTGTCTGCCCTTTggggcagtcccagc

>VIRT31560

MSSSARHSAMVLMFRKAASRAPVHSSQMAWFTRRRGDTSTACRLTVPARPMRVESSRGPL
LMMAFTRTCRGFSPVSRCMISKACLTMRTVMSFLPLLRPCIIMELVRRSTMGH

```
>NM_001009.3 Homo sapiens ribosomal protein S5 (RPS5), mRNA
CTCTTCCTGTCTGTACCAGGGCGGCGCGTGGTCTACGCCGAGTGACAGAGACGCTCAGGCTGTGTTCTCA
GGATGACCGAGTGGGAGACAGCAGCACCAGCGGTGGCAGAGACCCCAGACATCAAGCTCTTTGGGAAGTG
GAGCACCGATGATGTGCAGATCAATGACATTTCCCTGCAGGATTACATTGCAGTGAAGGAGAAGTATGCC
AAGTACCTGCCTCACAGTGCAGGGCGGTATGCCGCCAAACGCTTCCGCAAAGCTCAGTGTCCCATTGTGG
AGCGCCTCACTAACTCCATGATGATGCACGGCCGCAACAACGGCAAGAAGCTCATGACTGTGCGCATCGT
CAAGCATGCCTTCGAGATCATACACCTGCTCACAGGCGAGAACCCTCTGCAGGTCCTGGTGAACGCCATC
ATCAACAGTGGTCCCCGGGAGGACTCCACACGCATTGGGCGCGCCGGGACTGTGAGACGACAGGCTGTGG
ATGTGTCCCCCCTGCGCCGTGTGAACCAGGCCATCTGGCTGCTGTGCACAGGCGCTCGTGAGGCTGCCTT
CCGGAACATTAAGACCATTGCTGAGTGCCTGGCAGATGAGCTCATCAATGCTGCCAAGGGCTCCTCGAAC
TCCTATGCCATTAAGAAGAAGGACGAGCTGGAGCGTGTGGCCAAGTCCAACCGCTGATTTTCCCAGCTGC
TGCCCAATAAACCTGTCTGCCCTTTGGGGCAGTCCCAGCCAAAAAAAAAAAAAAA

>NP_001000.2 40S ribosomal protein S5 [Homo sapiens]
MTEWETAAPAVAETPDIKLFGKWSTDDVQINDISLQDYIAVKEKYAKYLPHSAGRYAAKRFRKAQCPIVE
RLTNSMMMHGRNNGKKLMTVRIVKHAFEIIHLLTGENPLQVLVNAIINSGPREDSTRIGRAGTVRRQAVD
VSPLRRVNQAIWLLCTGAREAAFRNIKTIAECLADELINAAKGSSNSYAIKKKDELERVAKSNR
i0_HQ_samplec9816d|c29292/f2p0/819      acggcaacctggacctgcggagggcggcggtgaccactgggcctcactccgctaacccag     60
i0_HQ_samplec9816d|c18100/f19p0/740     ------------------------------------------------------------     0
NM_001009.3                             ------------------------------------------------------------     0


i0_HQ_samplec9816d|c29292/f2p0/819      gtccgcatCAGGCGCGTGCCGGTGAGGCGGCTGTGGCCGCGGCGAGGAGTCAGAGGGAAC     120
i0_HQ_samplec9816d|c18100/f19p0/740     ---ctcttcctgtctgtac---------------cagggcggcgcGTGGTCTACG-----     37
NM_001009.3                             ---CTCTTCCTGTCTGTAC---------------CAGGGCGGCGCGTGGTCTACG-----     37
                                           * * **  *   ** *                ****** *  ***   *

i0_HQ_samplec9816d|c29292/f2p0/819      TGAACTGGCTACTCCCGCGGCAGGT-GGGATTTAAGGATGACCGGTGGGAGACAGCAGCA     179
i0_HQ_samplec9816d|c18100/f19p0/740     CCGAGTGGACAGAGACGCTCAGGCTGTGTTCTCAGGATGACCGAGTGGGAGACAGCAGCA     97
NM_001009.3                             CCGAGT-GACAGAGACGCTCAGGCTGTGTTCTCAGGATGACCGAGTGGGAGACAGCAGCA     96
                                           * * *  *    ***    * *  *   * * *     *  ***************

i0_HQ_samplec9816d|c29292/f2p0/819      CCAGCGGTGGCAGAGACCCCAGACATCAAGCTCTTTGGGAAGTGGAGCACCGATGATGTG     239
i0_HQ_samplec9816d|c18100/f19p0/740     CCAGCGGTGGCAGAGACCCCAGACATCAAGCTCTTTGGGAAGTGGAGCACCGATGATGTG     157
NM_001009.3                             CCAGCGGTGGCAGAGACCCCAGACATCAAGCTCTTTGGGAAGTGGAGCACCGATGATGTG     156
                                        ************************************************************
```

```
i0_HQ_samplec9816d|c29292/f2p0/819      CAGATCAATGACATTTCCCTGCAGGATTACATTGCAGTGAAGGAGAAGTATGCCAAGTAC    299
i0_HQ_samplec9816d|c18100/f19p0/740     CAGATCAATGACATTTCCCTGCAGGATTACATTGCAGTGAAGGAGAAGTATGCCAAGTAC    217
NM_001009.3                             CAGATCAATGACATTTCCCTGCAGGATTACATTGCAGTGAAGGAGAAGTATGCCAAGTAC    216
                                        ************************************************************

i0_HQ_samplec9816d|c29292/f2p0/819      CTGCCTCACAGTGCAGGGCGGTATGCCGCCAAACGCTTCCGCAAAGCTCAGTGTCCCATT    359
i0_HQ_samplec9816d|c18100/f19p0/740     CTGCCTCACAGTGCAGGGCGGTATGCCGCCAAACGCTTCCGCAAAGCTCAGTGTCCCATT    277
NM_001009.3                             CTGCCTCACAGTGCAGGGCGGTATGCCGCCAAACGCTTCCGCAAAGCTCAGTGTCCCATT    276
                                        ************************************************************

i0_HQ_samplec9816d|c29292/f2p0/819      GTGGAGCGCCTCACTAACTCCATGATGATGCACGGCCGCAACAACGGCAAGAAGCTCATG    419
i0_HQ_samplec9816d|c18100/f19p0/740     GTGGAGCGCCTCACTAACTCCATGATGATGCACGGCCGCAACAACGGCAAGAAGCTCATG    337
NM_001009.3                             GTGGAGCGCCTCACTAACTCCATGATGATGCACGGCCGCAACAACGGCAAGAAGCTCATG    336
                                        ************************************************************

i0_HQ_samplec9816d|c29292/f2p0/819      ACTGTGCGCATCGTCAAGCATGCCTTCGAGATCATACACCTGCTCACAGGCGAGAACCCT    479
i0_HQ_samplec9816d|c18100/f19p0/740     ACTGTGCGCATCGTCAAGCATGCCTTCGAGATCATACACCTGCTCACAGGCGAGAACCCT    397
NM_001009.3                             ACTGTGCGCATCGTCAAGCATGCCTTCGAGATCATACACCTGCTCACAGGCGAGAACCCT    396
                                        ************************************************************

i0_HQ_samplec9816d|c29292/f2p0/819      CTGCAGGTCCTGGTGAACGCCATCATCAACAGTGGTCCCCGGGAGGACTCCACACGCATT    539
i0_HQ_samplec9816d|c18100/f19p0/740     CTGCAGGTCCTGGTGAACGCCATCATCAACAGTGGTCCCCGGGAGGACTCCACACGCATT    457
NM_001009.3                             CTGCAGGTCCTGGTGAACGCCATCATCAACAGTGGTCCCCGGGAGGACTCCACACGCATT    456
                                        ************************************************************

i0_HQ_samplec9816d|c29292/f2p0/819      GGGCGCGCCGGGACTGTGAGACGACAGGCTGTGGATGTGTCCCCCCTGCGCCGTGTGAAC    599
i0_HQ_samplec9816d|c18100/f19p0/740     GGGCGCGCCGGGACTGTGAGACGACAGGCTGTGGATGTGTCCCCCCTGCGCCGTGTGAAC    517
NM_001009.3                             GGGCGCGCCGGGACTGTGAGACGACAGGCTGTGGATGTGTCCCCCCTGCGCCGTGTGAAC    516
                                        ************************************************************

i0_HQ_samplec9816d|c29292/f2p0/819      CAGGCCATCTGGCTGCTGTGCACAGGCGCTCGTGAGGCTGCCTTCCGGAACATTAAGACC    659
i0_HQ_samplec9816d|c18100/f19p0/740     CAGGCCATCTGGCTGCTGTGCACAGGCGCTCGTGAGGCTGCCTTCCGGAACATTAAGACC    577
NM_001009.3                             CAGGCCATCTGGCTGCTGTGCACAGGCGCTCGTGAGGCTGCCTTCCGGAACATTAAGACC    576
                                        ************************************************************

i0_HQ_samplec9816d|c29292/f2p0/819      ATTGCTGAGTGCCTGGCAGATGAGCTCATCAATGCTGCCAAGGGCTCCTCGAACTCCTAT    719
i0_HQ_samplec9816d|c18100/f19p0/740     ATTGCTGAGTGCCTGGCAGATGAGCTCATCAATGCTGCCAAGGGCTCCTCGAACTCCTAT    637
NM_001009.3                             ATTGCTGAGTGCCTGGCAGATGAGCTCATCAATGCTGCCAAGGGCTCCTCGAACTCCTAT    636
                                        ************************************************************

i0_HQ_samplec9816d|c29292/f2p0/819      GCCATTAAGAAGAAGGACGAGCTGGAGCGTGTGGCCAAGTCCAACCGCTGATTTTCCCAG    779
i0_HQ_samplec9816d|c18100/f19p0/740     GCCATTAAGAAGAAGGACGAGCTGGAGCGTGTGGCCAAGTCCAACCGCTGATTTTCCCAG    697
NM_001009.3                             GCCATTAAGAAGAAGGACGAGCTGGAGCGTGTGGCCAAGTCCAACCGCTGATTTTCCCAG    696
                                        ************************************************************

i0_HQ_samplec9816d|c29292/f2p0/819      CTGCTGCCcaataaacctgtctgccctttggggcagcccc-------------------    819
i0_HQ_samplec9816d|c18100/f19p0/740     CTGCTGCCCAATAAACCTGTCTGCCCTTTggggcagtcccagc----------------    740
NM_001009.3                             CTGCTGCCCAATAAACCTGTCTGCCCTTTGGGGCAGTCCCAGCCAAAAAAAAAAAAAAA    755
                                        ************************************ ***
```

```
VIRT30957       ------------------------------------------------------------    0
VIRT31560       ------------------------------------------------------------    0
NP_001000.2     MTEWETAAPAVAETPDIKLFGKWSTDDVQINDISLQDYIAVKEKYAKYLPHSAGRYAAKR    60

VIRT30957       ---------------MSSSARHSAMVLMFR-----------KAASRAPVHSSQMAWF---    31
VIRT31560       ---------------MSSSARHSAMVLMFR-----------KAASRAPVHSSQMAWF---    31
NP_001000.2     FRKAQCPIVERLTNSMMMHGRNNGKKLMTVRIVKHAFEIIHLLTGENPLQVLVNAIINSG    120
                               *   .*:..  **               :.. *::    * :

VIRT30957       ----TRRRGDTSTACRLTVPARPMRVESSRGPLLMMAFTRTCRGFSPVSRCMISKACLTM    87
VIRT31560       ----TRRRGDTSTACRLTVPARPMRVESSRGPLLMMAFTRTCRGFSPVSRCMISKACLTM    87
NP_001000.2     PREDSTRIGRAGTVRRQAVDVSPLRRVNQ-------AIWLLCTGARE-------------    160
                    : * * :.*. * :* . *:*  ..       *:   * *

VIRT30957       RTVMSFLPLLRPCIIMELVRRSTMGH-------------------    113
VIRT31560       RTVMSFLPLLRPCIIMELVRRSTMGH-------------------    113
NP_001000.2     -AAFRNIKTIAECLADELINAAKGSSNSYAIKKKDELERVAKSNR    204
                 :.:  :  :  *:  **:. :. .
```

## UCHL3

| Sequence | Header | AA Seq |
|---|---|---|
| Present | i1_HQ_samplec9816d\|c11981/f2p0/1124 | |
| Absent | i0_HQ_samplec9816d\|c69792/f39p31/881 | |
| NCBI | NM_001270952 | |
| | NM_006002 | |

>i1_HQ_samplec9816d|c11981/f2p0/1124

isoform=c11981;full_length_coverage=2;non_full_length_coverage=0;isoform_length=1124

tggcaaggctcggctcggaagagtccaagcgtgagggaagaggggctgtggattcagatactgtTTTTCTCCCGAAATAGGAAATTGGCTTAT
TTTTTTCTCCTCGGCAGCATCTTAATTTAAAATATGACACTTGACCTACGGCCCTGCACGGAGCGGTTAAGAGGGTCACC
AACCAGTTTCTTAAACAATTAGGTCTACATCCTAACTGGCAATTCGTTGATGTATATGGAATGGATCCTGAACTCCTTAG
CATGGTACCAAGACCAGTCTGTGCAGTCTTACTTCTCTTTCCTATTACAGAAAAGTATGAAGTATTCAGAACAGAAGAG
GAAGAAAAAATAAAATCTCAGGGACAAGATGTTACATCATCAGTATATTTCATGAAGCAAACAATCAGCAATGCCTGTG
GAACAATTGGACTGATTCATGCTATTGCAAACAATAAAGACAAGATGCACTTTGAATCTGGATCAACCTTGAAAAAATT
CCTGGAGGAATCTGTGTCAATGAGCCCTGAAGAACGAGCCAGATACCTGGAGAACTATGATGCCATCCGAGTTACTCAT
GAGACCAGTGCCCATGAAGGTCAGACTGAGTCCTCCTCGCCATCCTCATCACAGCCTCATTCTAGCCACTGCAGAACGA
AGGCCTCATCATTGTGTCACCATGCATCCCTGCCCTGGGTCAAACGTAACCATGTAGGCCCTGCAAAGGCTACCAGTCC
ATCTCTCCGTCTTCGTCGCTGGCGACCCTTCTTACGTCTACCATCTTTGGGTCTCCATTCTGTTGCACCAAGTATAGATGA
GAAAGTAGATCTTCATTTTATTGCATTAGTTCATGTAGATGGGCATCTCTATGAATTAGATGGGCGGAAGCCATTTCCAA
TTAACCATGGTGAAACTAGTGATGAAACTTTATTAGAGGATGCCATAGAAGTTTGCAAGAAGATTATGGAGCGCGACC
CTGATGAACTAAGATTTAATGCGATTGCTCTTTCTGCAGCATAGCTTGTCAATAATGGAAACACCAAAAACTGTATTATT
TGCAACTAAATTTTCTCTGCCATACACTAACTCAAAAATTTTGatattttcattaacttgatgattaaactttatgtg

>VIRT51255

MLRSSGSIPYTSTNCQLGCRPNCLRNWLVTLLTAPCRAVGQVSYFKLRCCRGEKNKPISY
FGRKTVSESTAPLPSRLDSSEPSLA

>i0_HQ_samplec9816d|c69792/f39p31/881
isoform=c69792;full_length_coverage=39;non_full_length_coverage=31;isoform_length=881

ggaagcggcggcggCGGCGAAGGCGGCGGCTGTCAGAGCTGGAGGGCCGGGCACCGCGGCCATGGAGGGTCAACGCTGG
CTGCCGCTGGAGGCCAATCCCGAGGTCACCAACCAGTTTCTTAAACAATTAGGTCTACATCCTAACTGGCAATTCGTTGA
TGTATATGGAATGGATCCTGAACTCCTTAGCATGGTACCAAGACCAGTCTGTGCAGTCTTACTTCTCTTTCCTATTACAGA
AAAGTATGAAGTATTCAGAACAGAAGAGGAAGAAAAAATAAAATCTCAGGGACAAGATGTTACATCATCAGTATATTT
CATGAAGCAAACAATCAGCAATGCCTGTGGAACAATTGGACTGATTCATGCTATTGCAAACAATAAAGACAAGATGCAC
TTTGAATCTGGATCAACCTTGAAAAAATTCCTGGAGGAATCTGTGTCAATGAGCCCTGAAGAACGAGCCAGATACCTGG
AGAACTATGATGCCATCCGAGTTACTCATGAGACCAGTGCCCATGAAGGTCAGACTGAGGCACCAAGTATAGATGAGA
AAGTAGATCTTCATTTTATTGCATTAGTTCATGTAGATGGGCATCTCTATGAATTAGATGGGCGGAAGCCATTTCCAATT
AACCATGGTGAAACTAGTGATGAAACTTTATTAGAGGATGCCATAGAAGTTTGCAAGAAGTTTATGGAGCGCGACCCT
GATGAACTAAGATTTAATGCGATTGCTCTTTCTGCAGCATAGCTTGTCAATAATGGAAACACCAAAAACTGTATTATTTG
CAACTAAATTTTCTCTGCCATACACTAACTCAAAAATTTTGATATTTTCATTAACTTGATGATTAAACTTTATGTGAGTTAA
ACTTTGC

>VIRT54307

MKIYFLIYTWCLSLTFMGTGLMSNSDGIIVLQVSGSFFRAH

>NM_001270952.1 Homo sapiens ubiquitin C-terminal hydrolase L3 (UCHL3), transcript variant 1, mRNA
GGCGCATGCGTAGAACGCGAGCGCTCGGCAAGGCTCGGCTCGGAAGAGTCCAAGCGTGAGGGGAGAGGGC
TGTGGATTCAGATACTGTTTTTCTCCCGAAATAGGAAATTGGCTTATTTTTTTCTCCTCGGCAGCATCTT
AATTTAAAATATGACACTTGACCTACGGCCCTGCACGGAGCGGTTAAGAGGGTCACCAACCAGTTTCTTA
AACAATTAGGTCTACATCCTAACTGGCAATTCGTTGATGTATATGGAATGGATCCTGAACTCCTTAGCAT
GGTACCAAGACCAGTCTGTGCAGTCTTACTTCTCTTTCCTATTACAGAAAAGTATGAAGTATTCAGAACA
GAAGAGGAAGAAAAAATAAAATCTCAGGGACAAGATGTTACATCATCAGTATATTTCATGAAGCAAACAA
TCAGCAATGCCTGTGGAACAATTGGACTGATTCATGCTATTGCAAACAATAAAGACAAGATGCACTTTGA
ATCTGGATCAACCTTGAAAAAATTCCTGGAGGAATCTGTGTCAATGAGCCCTGAAGAACGAGCCAGATAC
CTGGAGAACTATGATGCCATCCGAGTTACTCATGAGACCAGTGCCCATGAAGGTCAGACTGAGGCACCAA
GTATAGATGAGAAAGTAGATCTTCATTTTATTGCATTAGTTCATGTAGATGGGCATCTCTATGAATTAGA

```
TGGGCGGAAGCCATTTCCAATTAACCATGGTGAAACTAGTGATGAAACTTTATTAGAGGATGCCATAGAA
GTTTGCAAGAAGTTTATGGAGCGCGACCCTGATGAACTAAGATTTAATGCGATTGCTCTTTCTGCAGCAT
AGCTTGTCAATAATGGAAACACCAAAAACTGTATTATTTGCAACTAAATTTTCTCTGCCATACACTAACT
CAAAAATTTTGATATTTTCATTAACTTGATGATTAAACTTTATGTGAGTTAAACTTTGCCTTAACCTGTG
TTTTATGTTATTTTTGCTCCAGGTTAAAGGTGCAATGCTTTCCTCCTCTTTTCTTGTGAAGGATTTATCT
>NP_001257881.1 ubiquitin carboxyl-terminal hydrolase isozyme L3 isoform 1 [Homo sapiens]
MDPELLSMVPRPVCAVLLLFPITEKYEVFRTEEEEKIKSQGQDVTSSVYFMKQTISNACGTIGLIHAIAN
NKDKMHFESGSTLKKFLEESVSMSPEERARYLENYDAIRVTHETSAHEGQTEAPSIDEKVDLHFIALVHV
DGHLYELDGRKPFPINHGETSDETLLEDAIEVCKKFMERDPDELRFNAIALSAA


>NM_006002.4 Homo sapiens ubiquitin C-terminal hydrolase L3 (UCHL3), transcript variant 2, mRNA
GCGCGTGGGCGGAAGCGGCGGCGGCGGCGAAGGCGGCGGCTGTCAGAGCTGGAGGGCCGGGCACCGCGGC
CATGGAGGGTCAACGCTGGCTGCCGCTGGAGGCCAATCCCGAGGTCACCAACCAGTTTCTTAAACAATTA
GGTCTACATCCTAACTGGCAATTCGTTGATGTATATGGAATGGATCCTGAACTCCTTAGCATGGTACCAA
GACCAGTCTGTGCAGTCTTACTTCTCTTTCCTATTACAGAAAAGTATGAAGTATTCAGAACAGAAGAGGA
AGAAAAAATAAAATCTCAGGGACAAGATGTTACATCATCAGTATATTTCATGAAGCAAACAATCAGCAAT
GCCTGTGGAACAATTGGACTGATTCATGCTATTGCAAACAATAAAGACAAGATGCACTTTGAATCTGGAT
CAACCTTGAAAAAATTCCTGGAGGAATCTGTGTCAATGAGCCCTGAAGAACGAGCCAGATACCTGGAGAA
CTATGATGCCATCCGAGTTACTCATGAGACCAGTGCCCATGAAGGTCAGACTGAGGCACCAAGTATAGAT
GAGAAAGTAGATCTTCATTTTATTGCATTAGTTCATGTAGATGGGCATCTCTATGAATTAGATGGGCGGA
AGCCATTTCCAATTAACCATGGTGAAACTAGTGATGAAACTTTATTAGAGGATGCCATAGAAGTTTGCAA
GAAGTTTATGGAGCGCGACCCTGATGAACTAAGATTTAATGCGATTGCTCTTTCTGCAGCATAGCTTGTC
AATAATGGAAACACCAAAAACTGTATTATTTGCAACTAAATTTTCTCTGCCATACACTAACTCAAAAATT
TTGATATTTTCATTAACTTGATGATTAAACTTTATGTGAGTTAAACTTTGCCTTAACCTGTGTTTTATGT
TATTTTTGCTCCAGGTTAAAGGTGCAATGCTTTCCTCCTCTTTTCTTGTGAAGGATTTATCT
>NP_005993.1 ubiquitin carboxyl-terminal hydrolase isozyme L3 isoform 2 [Homo sapiens]
MEGQRWLPLEANPEVTNQFLKQLGLHPNWQFVDVYGMDPELLSMVPRPVCAVLLLFPITEKYEVFRTEEE
EKIKSQGQDVTSSVYFMKQTISNACGTIGLIHAIANNKDKMHFESGSTLKKFLEESVSMSPEERARYLEN
YDAIRVTHETSAHEGQTEAPSIDEKVDLHFIALVHVDGHLYELDGRKPFPINHGETSDETLLEDAIEVCK
KFMERDPDELRFNAIALSAA
i1_HQ_samplec9816d|c11981/f2p0/1124      -------------------------tggcaaggctcggctcggaagagtccaagcgtgag    35
NM_001270952.1                           GGCGCATGCGTAGAACGCGAGCGCTCGGCAAGGCTCGGCTCGGAAGAGTCCAAGCGTGAG    60
i0_HQ_samplec9816d|c69792/f39p31/881     ------------------------------------------------------------    0
NM_006002.4                              ---------------------------------------------------GCGCGTGGG    9


i1_HQ_samplec9816d|c11981/f2p0/1124      ggaagaggggctgtggattcagatactgtTTTTCTCCCGAAATAGGAAATTGGCTTATTT    95
NM_001270952.1                           GGGA-GAGGGCTGTGGATTCAGATACTGTTTTTCTCCCGAAATAGGAAATTGGCTTATTT    119
i0_HQ_samplec9816d|c69792/f39p31/881     -gga-agcggcggcggCGGCG------------------AAGGCGGCGGCT---------    31
NM_006002.4                              CGGA-AGCGGCGGCGGCGGCG------------------AAGGCGGCGGCT---------    41
                                          * *    *** * **   *                   **   **    *

i1_HQ_samplec9816d|c11981/f2p0/1124      TTTTCTCCTCGGCAGCATCTTAATTTAAAATATGACACTTGACCTACGGCCCTGCACGGA    155
NM_001270952.1                           TTTTCTCCTCGGCAGCATCTTAATTTAAAATATGACACTTGACCTACGGCCCTGCACGGA    179
i0_HQ_samplec9816d|c69792/f39p31/881     GTCAGAGCTGGAGGGCCGGGCACCGCGGCCATGGAGGGTCAACGCTGGCTGCCGCTGGAG    91
NM_006002.4                              GTCAGAGCTGGAGGGCCGGGCACCGCGGCCATGGAGGGTCAACGCTGGCTGCCGCTGGAG    101
                                          *     ** *   **      *             **    *  **     *    * **   *

i1_HQ_samplec9816d|c11981/f2p0/1124      GCGGTTAAGAGGGTCACCAACCAGTTTCTTAAACAATTAGGTCTACATCCTAACTGGCAA    215
NM_001270952.1                           GCGGTTAAGAGGGTCACCAACCAGTTTCTTAAACAATTAGGTCTACATCCTAACTGGCAA    239
i0_HQ_samplec9816d|c69792/f39p31/881     GCCAATCCCGAGGTCACCAACCAGTTTCTTAAACAATTAGGTCTACATCCTAACTGGCAA    151
NM_006002.4                              GCCAATCCCGAGGTCACCAACCAGTTTCTTAAACAATTAGGTCTACATCCTAACTGGCAA    161
                                         **   *     *************************************************

i1_HQ_samplec9816d|c11981/f2p0/1124      TTCGTTGATGTATATGGAATGGATCCTGAACTCCTTAGCATGGTACCAAGACCAGTCTGT    275
NM_001270952.1                           TTCGTTGATGTATATGGAATGGATCCTGAACTCCTTAGCATGGTACCAAGACCAGTCTGT    299
i0_HQ_samplec9816d|c69792/f39p31/881     TTCGTTGATGTATATGGAATGGATCCTGAACTCCTTAGCATGGTACCAAGACCAGTCTGT    211
NM_006002.4                              TTCGTTGATGTATATGGAATGGATCCTGAACTCCTTAGCATGGTACCAAGACCAGTCTGT    221
                                         ************************************************************

i1_HQ_samplec9816d|c11981/f2p0/1124      GCAGTCTTACTTCTCTTTCCTATTACAGAAAAGTATGAAGTATTCAGAACAGAAGAGGAA    335
NM_001270952.1                           GCAGTCTTACTTCTCTTTCCTATTACAGAAAAGTATGAAGTATTCAGAACAGAAGAGGAA    359
i0_HQ_samplec9816d|c69792/f39p31/881     GCAGTCTTACTTCTCTTTCCTATTACAGAAAAGTATGAAGTATTCAGAACAGAAGAGGAA    271
NM_006002.4                              GCAGTCTTACTTCTCTTTCCTATTACAGAAAAGTATGAAGTATTCAGAACAGAAGAGGAA    281
                                         ************************************************************

i1_HQ_samplec9816d|c11981/f2p0/1124      GAAAAAATAAAATCTCAGGGACAAGATGTTACATCATCAGTATATTTCATGAAGCAAACA    395
NM_001270952.1                           GAAAAAATAAAATCTCAGGGACAAGATGTTACATCATCAGTATATTTCATGAAGCAAACA    419
i0_HQ_samplec9816d|c69792/f39p31/881     GAAAAAATAAAATCTCAGGGACAAGATGTTACATCATCAGTATATTTCATGAAGCAAACA    331
NM_006002.4                              GAAAAAATAAAATCTCAGGGACAAGATGTTACATCATCAGTATATTTCATGAAGCAAACA    341
                                         ************************************************************

i1_HQ_samplec9816d|c11981/f2p0/1124      ATCAGCAATGCCTGTGGAACAATTGGACTGATTCATGCTATTGCAAACAATAAAGACAAG    455
NM_001270952.1                           ATCAGCAATGCCTGTGGAACAATTGGACTGATTCATGCTATTGCAAACAATAAAGACAAG    479
i0_HQ_samplec9816d|c69792/f39p31/881     ATCAGCAATGCCTGTGGAACAATTGGACTGATTCATGCTATTGCAAACAATAAAGACAAG    391
NM_006002.4                              ATCAGCAATGCCTGTGGAACAATTGGACTGATTCATGCTATTGCAAACAATAAAGACAAG    401
                                         ************************************************************

i1_HQ_samplec9816d|c11981/f2p0/1124      ATGCACTTTGAATCTGGATCAACCTTGAAAAAATTCCTGGAGGAATCTGTGTCAATGAGC    515
NM_001270952.1                           ATGCACTTTGAATCTGGATCAACCTTGAAAAAATTCCTGGAGGAATCTGTGTCAATGAGC    539
i0_HQ_samplec9816d|c69792/f39p31/881     ATGCACTTTGAATCTGGATCAACCTTGAAAAAATTCCTGGAGGAATCTGTGTCAATGAGC    451
NM_006002.4                              ATGCACTTTGAATCTGGATCAACCTTGAAAAAATTCCTGGAGGAATCTGTGTCAATGAGC    461
                                         ************************************************************

i1_HQ_samplec9816d|c11981/f2p0/1124      CCTGAAGAACGAGCCAGATACCTGGAGAACTATGATGCCATCCGAGTTACTCATGAGACC    575
NM_001270952.1                           CCTGAAGAACGAGCCAGATACCTGGAGAACTATGATGCCATCCGAGTTACTCATGAGACC    599
i0_HQ_samplec9816d|c69792/f39p31/881     CCTGAAGAACGAGCCAGATACCTGGAGAACTATGATGCCATCCGAGTTACTCATGAGACC    511
```

```
NM_006002.4                          CCTGAAGAACGAGCCAGATACCTGGAGAACTATGATGCCATCCGAGTTACTCATGAGACC    521
                                     ************************************************************

i1_HQ_samplec9816d|c11981/f2p0/1124  AGTGCCCATGAAGGTCAGACTGAGTCCTCCTCGCCATCCTCATCACAGCCTCATTCTAGC    635
NM_001270952.1                       AGTGCCCATGAAGGTCAGACTGA-------------------------------------    622
i0_HQ_samplec9816d|c69792/f39p31/881 AGTGCCCATGAAGGTCAGACTGA-------------------------------------    534
NM_006002.4                          AGTGCCCATGAAGGTCAGACTGA-------------------------------------    544
                                     ***********************

i1_HQ_samplec9816d|c11981/f2p0/1124  CACTGCAGAACGAAGGCCTCATCATTGTGTCACCATGCATCCCTGCCCTGGGTCAAACGT    695
NM_001270952.1                       ------------------------------------------------------------    622
i0_HQ_samplec9816d|c69792/f39p31/881 ------------------------------------------------------------    534
NM_006002.4                          ------------------------------------------------------------    544

i1_HQ_samplec9816d|c11981/f2p0/1124  AACCATGTAGGCCCTGCAAAGGCTACCAGTCCATCTCTCCGTCTTCGTCGCTGGCGACCC    755
NM_001270952.1                       ------------------------------------------------------------    622
i0_HQ_samplec9816d|c69792/f39p31/881 ------------------------------------------------------------    534
NM_006002.4                          ------------------------------------------------------------    544

i1_HQ_samplec9816d|c11981/f2p0/1124  TTCTTACGTCTACCATCTTTGGGTCTCCATTCTGTTGCACCAAGTATAGATGAGAAAGTA    815
NM_001270952.1                       -----------------------------------GGCACCAAGTATAGATGAGAAAGTA    647
i0_HQ_samplec9816d|c69792/f39p31/881 -----------------------------------GGCACCAAGTATAGATGAGAAAGTA    559
NM_006002.4                          -----------------------------------GGCACCAAGTATAGATGAGAAAGTA    569
                                                                    ************************

i1_HQ_samplec9816d|c11981/f2p0/1124  GATCTTCATTTTATTGCATTAGTTCATGTAGATGGGCATCTCTATGAATTAGATGGGCGG    875
NM_001270952.1                       GATCTTCATTTTATTGCATTAGTTCATGTAGATGGGCATCTCTATGAATTAGATGGGCGG    707
i0_HQ_samplec9816d|c69792/f39p31/881 GATCTTCATTTTATTGCATTAGTTCATGTAGATGGGCATCTCTATGAATTAGATGGGCGG    619
NM_006002.4                          GATCTTCATTTTATTGCATTAGTTCATGTAGATGGGCATCTCTATGAATTAGATGGGCGG    629
                                     ************************************************************

i1_HQ_samplec9816d|c11981/f2p0/1124  AAGCCATTTCCAATTAACCATGGTGAAACTAGTGATGAAACTTTATTAGAGGATGCCATA    935
NM_001270952.1                       AAGCCATTTCCAATTAACCATGGTGAAACTAGTGATGAAACTTTATTAGAGGATGCCATA    767
i0_HQ_samplec9816d|c69792/f39p31/881 AAGCCATTTCCAATTAACCATGGTGAAACTAGTGATGAAACTTTATTAGAGGATGCCATA    679
NM_006002.4                          AAGCCATTTCCAATTAACCATGGTGAAACTAGTGATGAAACTTTATTAGAGGATGCCATA    689
                                     ************************************************************

i1_HQ_samplec9816d|c11981/f2p0/1124  GAAGTTTGCAAGAAGATTATGGAGCGCGACCCTGATGAACTAAGATTTAATGCGATTGCT    995
NM_001270952.1                       GAAGTTTGCAAGAAGTTTATGGAGCGCGACCCTGATGAACTAAGATTTAATGCGATTGCT    827
i0_HQ_samplec9816d|c69792/f39p31/881 GAAGTTTGCAAGAAGTTTATGGAGCGCGACCCTGATGAACTAAGATTTAATGCGATTGCT    739
NM_006002.4                          GAAGTTTGCAAGAAGTTTATGGAGCGCGACCCTGATGAACTAAGATTTAATGCGATTGCT    749
                                     *************** ********************************************

i1_HQ_samplec9816d|c11981/f2p0/1124  CTTTCTGCAGCATAGCTTGTCAATAATGGAAACACCAAAAACTGTATTATTTGCAACTAA    1055
NM_001270952.1                       CTTTCTGCAGCATAGCTTGTCAATAATGGAAACACCAAAAACTGTATTATTTGCAACTAA    887
i0_HQ_samplec9816d|c69792/f39p31/881 CTTTCTGCAGCATAGCTTGTCAATAATGGAAACACCAAAAACTGTATTATTTGCAACTAA    799
NM_006002.4                          CTTTCTGCAGCATAGCTTGTCAATAATGGAAACACCAAAAACTGTATTATTTGCAACTAA    809
                                     ************************************************************

i1_HQ_samplec9816d|c11981/f2p0/1124  ATTTTCTCTGCCATACACTAACTCAAAAATTTTGatattttcattaacttgatgattaaa    1115
NM_001270952.1                       ATTTTCTCTGCCATACACTAACTCAAAAATTTTGATATTTTCATTAACTTGATGATTAAA    947
i0_HQ_samplec9816d|c69792/f39p31/881 ATTTTCTCTGCCATACACTAACTCAAAAATTTTGATATTTTCATTAACTTGATGATTAAA    859
NM_006002.4                          ATTTTCTCTGCCATACACTAACTCAAAAATTTTGATATTTTCATTAACTTGATGATTAAA    869
                                     ************************************************************

i1_HQ_samplec9816d|c11981/f2p0/1124  ctttatgtg---------------------------------------------------    1124
NM_001270952.1                       CTTTATGTGAGTTAAACTTTGCCTTAACCTGTGTTTTATGTTATTTTTGCTCCAGGTTAA    1007
i0_HQ_samplec9816d|c69792/f39p31/881 CTTTATGTGAGTTAAACTTTGC--------------------------------------    881
NM_006002.4                          CTTTATGTGAGTTAAACTTTGCCTTAACCTGTGTTTTATGTTATTTTTGCTCCAGGTTAA    929
                                     *********

i1_HQ_samplec9816d|c11981/f2p0/1124  -------------------------------------------         1124
NM_001270952.1                       AGGTGCAATGCTTTCCTCCTCTTTTCTTGTGAAGGATTTATCT         1050
i0_HQ_samplec9816d|c69792/f39p31/881 -------------------------------------------         881
NM_006002.4                          AGGTGCAATGCTTTCCTCCTCTTTTCTTGTGAAGGATTTATCT         972
```

```
VIRT54307           ------------------------------------------------------------ 0
VIRT51255           ------------------MLRSSGSIPYTSTNCQLG---------CRPNCLRN------- 26
NP_001257881.1      ------------------------------------MDPELLSMVPRPVCAVLLLFPITE 24
NP_005993.1         MEGQRWLPLEANPEVTNQFLKQLGLHPNWQFVDVYGMDPELLSMVPRPVCAVLLLFPITE 60


VIRT54307           ---------------------MKIYFLIYTWCLSLTFMGTGLMS----NSDG-------- 27
VIRT51255           -------------------------WLVTLLTAPCRA--VGQVSYFKLRCCR--GEKNKP 57
NP_001257881.1      KYEVFRTEEEEKIKSQGQDVTSSVYFMKQTISNACGT--IGLIHAIANNKDKMHFESGST 82
NP_005993.1         KYEVFRTEEEEKIKSQGQDVTSSVYFMKQTISNACGT--IGLIHAIANNKDKMHFESGST 118
                                             ::             * :     .

VIRT54307           ----------------------------------------------------IIVLQVSG 35
VIRT51255           ISYFGRKTVSESTAPLPSRL-----------------DSSEPSLA--------------- 85
NP_001257881.1      LKKFLEESVSMSPEERARYLENYDAIRVTHETSAHEGQTEAPSIDEKVDLHFIALVHVDG 142
NP_005993.1         LKKFLEESVSMSPEERARYLENYDAIRVTHETSAHEGQTEAPSIDEKVDLHFIALVHVDG 178
```

```
VIRT54307             SFFRAH------------------------------------------------          41
VIRT51255             ------------------------------------------------------          85
NP_001257881.1        HLYELDGRKPFPINHGETSDETLLEDAIEVCKKFMERDPDELRFNAIALSAA          194
NP_005993.1           HLYELDGRKPFPINHGETSDETLLEDAIEVCKKFMERDPDELRFNAIALSAA          230
```

## PAXX

| Sequence | Header | AA Seq | |
|---|---|---|---|
| **Present** | i1_HQ_samplec9816d\|c19870/f5p21/1131 | >VIRT110879 | >VIRT112892 |
| **Absent** | | | |
| | | | |
| **NCBI** | NM_183241 | NP_899064.1 | |
| | NR_138076 | | |

>i1_HQ_samplec9816d|c19870/f5p21/1131

isoform=c19870;full_length_coverage=5;non_full_length_coverage=21;isoform_length=1131

cccaagccccgcccgccggttcccgctcgcggcgccatggatccgctgtcgccGGCTCTGACGCTGCCGCCGGGCCCCGAGCCGCCCCGCT
TCGTGTGCTACTGCGAAGGGGAGGAAAGCGGGGAGGGGGACCGCGGCGGCTTCAACCTCTACGTGACCGACGCCGCG
GAGCTTTGGAGCACCTGCTTCACGCCGGACAGCCTGGCGGCCCTCGTGGGTAACTGGGCGGGTCTGGGAGCCGCCACA
CCCCTCCTTGCAGTGCAGATCGTCTATGGGGCGACAGACATCTGGGATTCCCCAGAAGGCTCTGACACCCTCTGCCCGC
CCTGTAGCTGTAGTCCTCCCATTGGCTAGGGCTCTGGGGTCGGGCAGGTTTCGGGTGCCCCCAGTGGGCCTCGGGTTCC
AGGCAGCTCGTGACAAGCCCCTGTGCTCTCTAGAAAGCCCGTTTTGGCCTGAGTGCGGCTGAGGACATCACCCCCCGGT
TCAGGTGAGACCCAAGCAGGGAGGAAGGACGGGTGGGGAGGAGGAGGGTCTGCCACAGCTCTCCGCACCTCTCCTCT
CCCAGGGCAGCCTGTGAGCAGCAAGCTGTGGCTCTGACTCTGCAGGAGGACAGAGCATCCCTGACGCTTTCAGGGGG
GCCCTCGGCACTGGCCTTTGACCTCTCCAAGGTACCAGGCCCAGAGGCAGCCCCCAGGCTGCGGGCGCTGACACTGGG
CCTGGCAAAACGCGTGTGGAGCCTGGAGCGGCGACTGGCAGCTGCAGAAGAGACAGCTGTCAGCCCGAGGAAGAGC
CCCCGGCCTGCAGGGCCTCAGCTCTTCTTACCAGACCCAGATCCCCAGAGAGGTGGCCCTGGACCTGGAGTCAGGAGG
CGGTGTCCAGGAGAGTCGCTCATCAACCCCGGGTTCAAGAGTAAGAAACCAGCTGGTGGCGTGGACTTCGATGAGACC
TGAAGGTGCAGCACAAGCGTGGCCCCGCGGGGAGTCCGCCTATGAGGGGAGAGGCAGTCTTTGAGGCCCCCATCAGA
GACCCCCCGCCACCACCTCCACCTGCCTGTCCTGGGCCAGGACTAACACGGCTCCTCAAATTCCTTCCCTGTCAAAtaaac
agctcccttggttggaggctctggtg

>VIRT110879

MGASKTASPLIGGLPAGPRLCCTFRSHRSPRHQLVSYS

>VIRT112892

MLCPPAESEPQLAAHRLPWERRGAESCGRPSSSPPVLPPCLGLT

```
>NM_183241.2 Homo sapiens PAXX, non-homologous end joining factor (PAXX), transcript variant 2, mRNA
CGCCGGGTTCCCGCTCGCCCGGCGCCATGGATCCGCTGTCGCCGCCGCTCTGCACGCTGCCGCCGGGCCC
CGAGCCGCCCCGCTTCGTGTGCTACTGCGAAGGGGAGGAAAGCGGGGAGGGGGACCGCGGCGGCTTCAAC
CTCTACGTGACCGACGCCGCGGAGCTTTGGAGCACCTGCTTCACGCCGGACAGCCTGGCGGCCCTCAAAG
CCCGTTTTGGCCTGAGTGCGGCTGAGGACATCACCCCCCGGTTCAGGGCAGCCTGTGAGCAGCAAGCTGT
GGCTCTGACTCTGCAGGAGGACAGAGCATCCCTGACGCTTTCAGGGGGGCCCTCGGCACTGGCCTTTGAC
CTCTCCAAGGTACCAGGCCCAGAGGCAGCCCCCAGGCTGCGGGCGCTGACACTGGGCCTGGCAAAACGCG
TGTGGAGCCTGGAGCGGCGACTGGCAGCTGCAGAAGAGACAGCTGTCAGCCCGAGGAAGAGCCCCCGGCC
TGCAGGGCCTCAGCTCTTCTTACCAGACCCAGATCCCCAGAGAGGTGGCCCTGGACCTGGAGTCAGGAGG
CGGTGTCCAGGAGAGTCGCTCATCAACCCCGGGTTCAAGAGTAAGAAACCAGCTGGTGGCGTGGACTTCG
ATGAGACCTGAAGGTGCAGCACAAGCGTGGCCCCGCGGGGAGTCCGCCTATGAGGGGAGAGGCAGTCTTT
```

```
GAGGCCCCCATCAGAGACCCCCCGCCACCACCTCCACCTGCCTGTCCTGGGCCAGGACTAACACGGCTCC
TCAAATTCCTTCCCTGTCAAATAAACAGCTCCCTTGGTTGGAGGCTCTGGTGGGAAAAA

>NP_899064.1 protein PAXX isoform 2 [Homo sapiens]
MDPLSPPLCTLPPGPEPPRFVCYCEGEESGEGDRGGFNLYVTDAAELWSTCFTPDSLAALKARFGLSAAE
DITPRFRAACEQQAVALTLQEDRASLTLSGGPSALAFDLSKVPGPEAAPRLRALTLGLAKRVWSLERRLA
AAEETAVSPRKSPRPAGPQLFLPDPDPQRGGPGPGVRRRCPGESLINPGFKSKKPAGGVDFDET


>NR_138076.1 Homo sapiens PAXX, non-homologous end joining factor (PAXX), transcript variant 6, non-
coding RNA
CGCCGGGTTCCCGCTCGCCCGGCGCCATGGATCCGCTGTCGCCGCCGCTCTGCACGCTGCCGCCGGGCCC
CGAGCCGCCCCGCTTCGTGTGCTACTGCGAAGGGGAGGAAAGCGGGGAGGGGGACCGCGGCGGCTTCAAC
CTCTACGTGACCGACGCCGCGGAGCTTTGGAGCACCTGCTTCACGCCGGACAGCCTGGCGGCCCTCGTGG
GGCAGCCTGTGAGCAGCAAGCTGTGGCTCTGACTCTGCAGGAGGACAGAGCATCCCTGACGCTTTCAGGG
GGGCCCTCGGCACTGGCCTTTGACCTCTCCAAGGTACCAGGCCCAGAGGCAGCCCCCAGGCTGCGGGCGC
TGACACTGGGCCTGGCAAAACGCGTGTGGAGCCTGGAGCGGCGACTGGCAGCTGCAGAAGAGACAGCTGT
CAGCCCGAGGAAGAGCCCCCGGCCTGCAGGGCCTCAGCTCTTCTTACCAGACCCAGATCCCCAGAGAGGT
GGCCCTGGACCTGGAGTCAGGAGGCGGTGTCCAGGAGAGTCGCTCATCAACCCCGGGTTCAAGAGTAAGA
AACCAGCTGGTGGCGTGGACTTCGATGAGACCTGAAGGTGCAGCACAAGCGTGGCCCCGCGGGGAGTCCG
CCTATGAGGGGAGAGGCAGTCTTTGAGGCCCCCATCAGAGACCCCCCGCCACCACCTCCACCTGCCTGTC
CTGGGCCAGGACTAACACGGCTCCTCAAATTCCTTCCCTGTCAAATAAACAGCTCCCTTGGTTGGAGGCT
CTGGTGGG
```

```
i1_HQ_samplec9816d|c19870/f5p21/1131      cccaagccccgcccgccggttcccgctcgcggcgccatggatccgctgtcgccGGCTCTG
NM_183241.2                               ------------------------------------------------------------      0
NR_138076.1                               ------------------------------------------------------------      0


i1_HQ_samplec9816d|c19870/f5p21/1131      ACGCTGCCGCCGGGCCCCGAGCCGCCCCGCTTCGTGTGCTACTGCGAAGGGGAGGAAAGC      12
NM_183241.2                               ------------------------------------------------------------      0
NR_138076.1                               ------------------------------------------------------------      0


i1_HQ_samplec9816d|c19870/f5p21/1131      GGGGAGGGGGACCGCGGCGGCTTCAACCTCTACGTGACCGACGCCGCGGAGCTTTGGAGC      18
NM_183241.2                               ------------------------------------------------------------      0
NR_138076.1                               ------------------------------------------------------------      0


i1_HQ_samplec9816d|c19870/f5p21/1131      ACCTGCTTCACGCCGGACAGCCTGGCGGCCCTCGTGGGTAACTGGGCGGGTCTGGGAGCC      24
NM_183241.2                               ------------------------------------------------------------      0
NR_138076.1                               ------------------------------------------------------------      0


i1_HQ_samplec9816d|c19870/f5p21/1131      GCCACACCCCTCCTTGCAGTGCAGATCGTCTATGGGGCGACAGACATCTGGGATTCCCCA      30
NM_183241.2                               ----------------------------------------CGCCGGGTTCCCGCTCGCCC      20
NR_138076.1                               ----------------------------------------CGCCGGGTTCCCGCTCGCCC      20
                                                                                  *       *     ** **

i1_HQ_samplec9816d|c19870/f5p21/1131      GAAGGCTCTGACACCCTCTGCCCGCCCTGTAGCTGTAGTCCTCCCATTGGCTAGGGCTCT      36
NM_183241.2                               GGCGCCATGGATCCG-----------CTGTCGCCGCCGCTCTGCACGCTGCCGCCGGGCC      69
NR_138076.1                               GGCGCCATGGATCCG-----------CTGTCGCCGCCGCTCTGCACGCTGCCGCCGGGCC      69
                                          *  * *   **  *            **** ** *  *  ** *     **    *  *

i1_HQ_samplec9816d|c19870/f5p21/1131      GGGGTCGGGCAGGTTTCGGGTGCCCCCAGTGGGCCTCGGGTTCCAGGCAGCTCGTGACAA      42
NM_183241.2                               CCGAGCCGCCCCGCTTCGTGTGCTACTGCGAAGGGGAGGAAAGCGGGGAGGGGGACCGCG      12
NR_138076.1                               CCGAGCCGCCCCGCTTCGTGTGCTACTGCGAAGGGGAGGAAAGCGGGGAGGGGGACCGCG      12
                                            *  * * *  * **** ****  *      *    **    * ** **   *

i1_HQ_samplec9816d|c19870/f5p21/1131      GCCCCTGTGCTCTCTAGAAAGCCCGTTTTGGCCTGAGTGCGGCTGAGGACATCACCCCCC      48
NM_183241.2                               GCGGCTTCAACCTCTACGT-------GACCGAC-----GCCGCGGAGCTTTGGAGCACCT      17
NR_138076.1                               GCGGCTTCAACCTCTACGT-------GACCGAC-----GCCGCGGAGCTTTGGAGCACCT      17
                                          **  **     *****              * *     ** ** ***      * * **

i1_HQ_samplec9816d|c19870/f5p21/1131      GGTTCAGGTGAGA--CCCAAGCAGGGAGGAAGGACGGGTGGGGAGGAGGAGGGTCTGCCA      53
NM_183241.2                               GCTTCACGCCGGACAGCCTGGCGGCCCTCAAAGCCCGTTT--------TGGCCTGAGTGC      22
NR_138076.1                               GCTTCACGCCGGACAGCCTGGCGGCCCTCG------------------------------      20
                                          * **** *   **   **  ** *

i1_HQ_samplec9816d|c19870/f5p21/1131      CAGCTCTCCGCACCTCTCCTCTCCCAGGGCAGCCTGTGAGCAGCAAGCTGTGGCTCTGAC      59
NM_183241.2                               GGCTGAGGACATCACCCCCCGGTTCAGGGCAGCCTGTGAGCAGCAAGCTGTGGCTCTGAC      28
NR_138076.1                               ------------------------TGGGGCAGCCTGTGAGCAGCAAGCTGTGGCTCTGAC      24
                                                                    **********************************

i1_HQ_samplec9816d|c19870/f5p21/1131      TCTGCAGGAGGACAGAGCATCCCTGACGCTTTCAGGGGGGCCCTCGGCACTGGCCTTTGA      65
NM_183241.2                               TCTGCAGGAGGACAGAGCATCCCTGACGCTTTCAGGGGGGCCCTCGGCACTGGCCTTTGA      34
NR_138076.1                               TCTGCAGGAGGACAGAGCATCCCTGACGCTTTCAGGGGGGCCCTCGGCACTGGCCTTTGA      30
```

```
                                           ************************************************************

i1_HQ_samplec9816d|c19870/f5p21/1131       CCTCTCCAAGGTACCAGGCCCAGAGGCAGCCCCCAGGCTGCGGGCGCTGACACTGGGCCT       71
NM_183241.2                                CCTCTCCAAGGTACCAGGCCCAGAGGCAGCCCCCAGGCTGCGGGCGCTGACACTGGGCCT       40
NR_138076.1                                CCTCTCCAAGGTACCAGGCCCAGAGGCAGCCCCCAGGCTGCGGGCGCTGACACTGGGCCT       36
                                           ************************************************************

i1_HQ_samplec9816d|c19870/f5p21/1131       GGCAAAACGCGTGTGGAGCCTGGAGCGGCGACTGGCAGCTGCAGAAGAGACAGCTGTCAG       77
NM_183241.2                                GGCAAAACGCGTGTGGAGCCTGGAGCGGCGACTGGCAGCTGCAGAAGAGACAGCTGTCAG       46
NR_138076.1                                GGCAAAACGCGTGTGGAGCCTGGAGCGGCGACTGGCAGCTGCAGAAGAGACAGCTGTCAG       42
                                           ************************************************************

i1_HQ_samplec9816d|c19870/f5p21/1131       CCCGAGGAAGAGCCCCCGGCCTGCAGGGCCTCAGCTCTTCTTACCAGACCCAGATCCCCA       83
NM_183241.2                                CCCGAGGAAGAGCCCCCGGCCTGCAGGGCCTCAGCTCTTCTTACCAGACCCAGATCCCCA       52
NR_138076.1                                CCCGAGGAAGAGCCCCCGGCCTGCAGGGCCTCAGCTCTTCTTACCAGACCCAGATCCCCA       48
                                           ************************************************************

i1_HQ_samplec9816d|c19870/f5p21/1131       GAGAGGTGGCCCTGGACCTGGAGTCAGGAGGCGGTGTCCAGGAGAGTCGCTCATCAACCC       89
NM_183241.2                                GAGAGGTGGCCCTGGACCTGGAGTCAGGAGGCGGTGTCCAGGAGAGTCGCTCATCAACCC       58
NR_138076.1                                GAGAGGTGGCCCTGGACCTGGAGTCAGGAGGCGGTGTCCAGGAGAGTCGCTCATCAACCC       54
                                           ************************************************************

i1_HQ_samplec9816d|c19870/f5p21/1131       CGGGTTCAAGAGTAAGAAACCAGCTGGTGGCGTGGACTTCGATGAGACCTGAAGGTGCAG       95
NM_183241.2                                CGGGTTCAAGAGTAAGAAACCAGCTGGTGGCGTGGACTTCGATGAGACCTGAAGGTGCAG       64
NR_138076.1                                CGGGTTCAAGAGTAAGAAACCAGCTGGTGGCGTGGACTTCGATGAGACCTGAAGGTGCAG       60
                                           ************************************************************

i1_HQ_samplec9816d|c19870/f5p21/1131       CACAAGCGTGGCCCCGCGGGGAGTCCGCCTATGAGGGGAGAGGCAGTCTTTGAGGCCCCC       10
NM_183241.2                                CACAAGCGTGGCCCCGCGGGGAGTCCGCCTATGAGGGGAGAGGCAGTCTTTGAGGCCCCC       70
NR_138076.1                                CACAAGCGTGGCCCCGCGGGGAGTCCGCCTATGAGGGGAGAGGCAGTCTTTGAGGCCCCC       66
                                           ************************************************************

i1_HQ_samplec9816d|c19870/f5p21/1131       ATCAGAGACCCCCCGCCACCACCTCCACCTGCCTGTCCTGGGCCAGGACTAACACGGCTC       10
NM_183241.2                                ATCAGAGACCCCCCGCCACCACCTCCACCTGCCTGTCCTGGGCCAGGACTAACACGGCTC       76
NR_138076.1                                ATCAGAGACCCCCCGCCACCACCTCCACCTGCCTGTCCTGGGCCAGGACTAACACGGCTC       72
                                           ************************************************************

i1_HQ_samplec9816d|c19870/f5p21/1131       CTCAAATTCCTTCCCTGTCAAAtaaacagctcccttggttggaggctctggtg-------       11
NM_183241.2                                CTCAAATTCCTTCCCTGTCAAATAAACAGCTCCCTTGGTTGGAGGCTCTGGTGGGAAAAA       82
NR_138076.1                                CTCAAATTCCTTCCCTGTCAAATAAACAGCTCCCTTGGTTGGAGGCTCTGGTGGG-----       77
                                           *****************************************************
```

```
VIRT110879          MGASKTASPLIGGLPAG---PRLCCTFRSHRSPRHQLVS---YS----------------     38
VIRT112892          ------------------------------------------------------------     0
NP_899064.1         ---MDPLSPPLCTLPPGPEPPRFVCYCEGEESGEGDRGGFNLYVTDAAELWSTCFTPDSL     57

VIRT110879          ------------------------------------------------------------     38
VIRT112892          ----------------------------------------------------MLCPPAES     8
NP_899064.1         AALKARFGLSAAEDITPRFRAACEQQAVALTLQEDRASLTLSGGPSALAFDLSKVPGPEA     117

VIRT110879          ------------------------------------------------------------     38
VIRT112892          EPQLAAH-----RLPWERRGA-----ESCGRPSS---------------------SPPVL     37
NP_899064.1         APRLRALTLGLAKRVWSLERRLAAAEETAVSPRKSPRPAGPQLFLPDPDPQRGGPGPGVR     177

VIRT110879          ---------------------------     38
VIRT112892          PPCLGLT--------------------     44
NP_899064.1         RRCPGESLINPGFKSKKPAGGVDFDET     204
```

## IFITM1

| Sequence | Header | AA Seq | |
|---|---|---|---|
| Present | i1_HQ_samplec9816d\|c2133/f4p0/1237 | >VIRT168080 | |
| Absent | i0_HQ_samplec9816d\|c33361/f2p4/957 | >VIRT175835 | |
| | i0_HQ_samplec9816d\|c49271/f4p37/679 | >VIRT177472 | |
| NCBI | NM_003641 | >NP_003632.3 | |
| | | | |

>i1_HQ_samplec9816d|c2133/f4p0/1237
isoform=c2133;full_length_coverage=4;non_full_length_coverage=0;isoform_length=1237

gagaaactgaaacgacaggggaaaggaggtctcactgagcaccgtcccagcatcCGGACACCACAGCGGCCCTTCGCTCCACGCAGAAA
ACCACACTTCTCAAACCTTCACTCAACACTTCCTTCCCCAAAGCCAGAAGATGCACAAGGAGGAACATGAGGTGGCTGT
GCTGGGGGCACCCCCCAGCACCATCCTTCCAAGGTCCACCGTGATCAACATCCACAGCGAGACCTCCGTGCCCGACCAT
GTCGTCTGGTCCCTGTTCAACACCCTCTTCTTGAACTGGTGCTGTCTGGGCTTCATAGCATTCGCCTACTCCGTGAAGGT
GCGTATGGCCCTGGCGGAAATCCAGGGGGTGCCGGTGAGCCTGGGGCTCCACCTGCCCACATGCTGCCTGGGGTGGG
GACTTGTGTGTCCCTGTGACTGTGAGTTTGTGTGCACCTCTGCCCCGTGTGTGCTCACGTCAGTAGCTTTGTCTGTGTGA
TCTGTGTGTGTGTGTGGCTTTGGGGAATCTGCCCAGTGCAGGTCTAGGAGGAGGCTCCAGGAGGCTGGCTGGCTGGCT
CAGAGTCTGTCCCTGGCTATCCATTAGCCCAGAGCAATTCTCCCTATAGCCCAGTAAGAAATTACACCCTCACCTTCCAG
ACTGGCACCCAGGCTCTCCCAGAAAGTGAGAAGGGAACTCACAGGTGACTTCACCCCATGGTGGGGAGAACAGCCTGT
GCTGGGGCCAAGGCAGAAGGAGGATGAGCCCCGAGGCTCCTGGAGAGTCTGAGCCCGGGTGAGGAAGGGGAGGAG
GTGGTCCCTGATCTCAGGGCGGGGAGGAGACGGAGCCATAGCACGCGGCTCTCAGCTGGGGGATCCTGGTCCCCTCA
CCATCTCCTCTCCCCCAGTCTAGGGACAGGAAGATGGTTGGCGACGTGACCGGGGCCCAGGCCTATGCCTCCACCGCCA
AGTGCCTGAACATCTGGGCCCTGATTCTGGGCATCCTCATGACCATTGGATTCATCCTGTTACTGGTATTCGGCTCTGTG
ACAGTCTACCATATTATGTTACAGATAATACAGGAAAAACGGGGTTACTAGTAGCCGCCCATAGCCTGCAACCTTTGCA
CTCCACTGTGCAATGCTGGCCCTGCACGCTGGGGCTGTTGCCCCTGCCCCCTTGGTCCTGCCCCTAGATACAGCAGTTTA
TACCCACACACCTGTCTACAGTGTCATTcaataaagtgcacgtgctt

>VIRT168080

MVGHGGLAVDVDHGGPWKDGAGGCPQHSHLMFLLVHLLALGKEVLSEGLRSVVFCVERRA
AVVSGCWDGAQ

>i0_HQ_samplec9816d|c33361/f2p4/957
isoform=c33361;full_length_coverage=2;non_full_length_coverage=4;isoform_length=957

ctctgaagctgcccctacaggccctggaatcctccccgtgtctcaacaCACTACCCTCTGGGGAGTCAGTTCTCCCCAGAGTCCCCGTTCC
TGGGCCTGGAGGGCTGCTTTGGGGCAGGAAACTTTGGCCACCAGGCCTCTGACCTGCACCAGGAGACACTGGGAGGT
TTAGTCCCCAAACCCGCACAGAGCAGGACTGCAGCCTGAGGAAAGAGCAAGGATTTCAGGAGAGAGGCCTGCGACAA
GTGAGCAGGAAATAGAAACTTAAGAGAAATACACACTTCTGAGAAACTGAAACGACAGGGGAAAGGAGGTCTCACTG
AGCACCGTCCCAGCATCCGGACACCACAGCGGCCCTTCGCTCCACGCAGAAAACCACACTTCTCAAACCTTCACTCAACA
CTTCCTTCCCCAAAGCCAGAAGATGCACAAGGAGGAACATGAGGTGGCTGTGCTGGGGGCACCCCCCAGCACCATCCT
TCCAAGGTCCACCGTGATCAACATCCACAGCGAGACCTCCGTGCCCGACCATGTCGTCTGGTCCCTGTTCAACACCCTCT
TCTTGAACTGGTGCTGTCTGGGCTTCATAGCATTCGCCTACTCCGTGAAGTCTAGGGACAGGAAGATGGTTGGCGACGT
GACCGGGGCCCAGGCCTATGCCTCCACCGCCAAGTGCCTGAACATCTGGGCCCTGATTCTGGGCATCCTCATGACCATT
GGATTCATCCTGTTACTGGTATTCGGCTCTGTGACAGTCTACCATATTATGTTACAGATAATACAGGAAAAACGGGGTTA
CTAGTAGCCGCCCATAGCCTGCAACCTTTGCACTCCACTGTGCAATGCTGGCCCTGCACGCTGGGGCTGTTGCCCCTGCC
CCCTTGGTCCTGCCCCTAGATACAGCAGTTTATACCCACACACCTGTCTACAGTGTCATTCAATAAAGTGCACGTGCTT

>VIRT175835

MLGRCSVRPPFPCRFSFSEVCISLKFLFPAHLSQASLLKSLLFPQAAVLLCAGLGTKPPS
VSWCRSEAWWPKFPAPKQPSRPRNGDSGEN

>i0_HQ_samplec9816d|c49271/f4p37/679
isoform=c49271;full_length_coverage=4;non_full_length_coverage=37;isoform_length=679

tcttctgagaaactGAAACGACAGGGGAAAGGAGGTCTCACTGAGCACCGTCCCAGCATCCGGACACCACAGCGGCCCTTC
GCTCCACGCAGAAAACCACACTTCTCAAACCTTCACTCAACACTTCCTTCCCCAAAGCCAGAAGATGCACAAGGAGGAA
CATGAGGTGGCTGTGCTGGGGGCACCCCCCAGCACCATCCTTCCAAGGTCCACCGTGATCAACATCCACAGCGAGACCT
CCGTGCCCGACCATGTCGTCTGGTCCCTGTTCAACACCCTCTTCTTGAACTGGTGCTGTCTGGGCTTCATAGCATTCGCCT
ACTCCGTGAAGTCTAGGGACAGGAAGATGGTTGGCGACGTGACCGGGGCCCAGGCCTATGCCTCCACCGCCAAGTGCC
TGAACATCTGGGCCCTGATTCTGGGCATCCTCATGACCATTGGATTCATCCTGTTACTGGTATTCGGCTCTGTGACAGTC
TACCATATTATGTTACAGATAATACAGGAAAAACGGGGTTACTAGTAGCCGCCCATAGCCTGCAACCTTTGCACTCCACT
GTGCAATGCTGGCCCTGCACGCTGGGGCTGTTGCCCCTGCCCCTTGGTCCTGCCCCTAGATACAGCAGTTTATACCCAC
ACACCTGTCTACAGTGTCATTCAATAAAGTGCACGTGCTTG

```
>VIRT177472
```

```
MVGHGGLAVDVDHGGPWKDGAGGCPQHSHLMFLLVHLLALGKEVLSEGLRSVVFCVERRA
AVVSGCWDGAQ
```

```
>NM_003641.3 Homo sapiens interferon induced transmembrane protein 1 (IFITM1), mRNA
AAACAGCAGGAAATAGAAACTTAAGAGAAATACACACTTCTGAGAAACTGAAACGACAGGGGAAAGGAGG
TCTCACTGAGCACCGTCCCAGCATCCGGACACCACAGCGGCCCTTCGCTCCACGCAGAAAACCACACTTC
TCAAACCTTCACTCAACACTTCCTTCCCCAAAGCCAGAAGATGCACAAGGAGGAACATGAGGTGGCTGTG
CTGGGGGCACCCCCCAGCACCATCCTTCCAAGGTCCACCGTGATCAACATCCACAGCGAGACCTCCGTGC
CCGACCATGTCGTCTGGTCCCTGTTCAACACCCTCTTCTTGAACTGGTGCTGTCTGGGCTTCATAGCATT
CGCCTACTCCGTGAAGTCTAGGGACAGGAAGATGGTTGGCGACGTGACCGGGGCCCAGGCCTATGCCTCC
ACCGCCAAGTGCCTGAACATCTGGGCCCTGATTCTGGGCATCCTCATGACCATTGGATTCATCCTGTTAC
TGGTATTCGGCTCTGTGACAGTCTACCATATTATGTTACAGATAATACAGGAAAAACGGGGTTACTAGTA
GCCGCCCATAGCCTGCAACCTTTGCACTCCACTGTGCAATGCTGGCCCTGCACGCTGGGGCTGTTGCCCC
TGCCCCCTTGGTCCTGCCCCTAGATACAGCAGTTTATACCCACACACCTGTCTACAGTGTCATTCAATAA
AGTGCACGTGCTTGTGAAAAAAAAAAAAAAAAA
```

```
>NP_003632.3 interferon-induced transmembrane protein 1 [Homo sapiens]
MHKEEHEVAVLGAPPSTILPRSTVINIHSETSVPDHVVWSLFNTLFLNWCCLGFIAFAYSVKSRDRKMVG
DVTGAQAYASTAKCLNIWALILGILMTIGFILLLVFGSVTVYHIMLQIIQEKRGY
```

```
i1_HQ_samplec9816d|c2133/f4p0/1237      gagaaactgaaacgacaggggaaaggaggtctcactgagcaccgtcccagcatcCGGACA 60
i0_HQ_samplec9816d|c33361/f2p4/957      ------------------------------------------------------------ 0
i0_HQ_samplec9816d|c49271/f4p37/679     ------------------------------------------------------------ 0
NM_003641.3                             ------------------------------------------------------------ 0
```

```
i1_HQ_samplec9816d|c2133/f4p0/1237      CCACAGCGGCCCTTCGCTCCACGCAGAAAACCACACTTCTCAAACCTTCACTCAACACTT 120
i0_HQ_samplec9816d|c33361/f2p4/957      ------------------------------------------------------------ 0
i0_HQ_samplec9816d|c49271/f4p37/679     ------------------------------------------------------------ 0
NM_003641.3                             ------------------------------------------------------------ 0
```

```
i1_HQ_samplec9816d|c2133/f4p0/1237      CCTTCCCCAAAGCCAGAAGATGCACAAGGAGGAACATGAGGTGGCTGTGCTGGGGGCACC 180
i0_HQ_samplec9816d|c33361/f2p4/957      ------------------------------------------------------------ 0
i0_HQ_samplec9816d|c49271/f4p37/679     ------------------------------------------------------------ 0
NM_003641.3                             ------------------------------------------------------------ 0
```

```
i1_HQ_samplec9816d|c2133/f4p0/1237      CCCCAGCACCATCCTTCCAAGGTCCACCGTGATCAACATCCACAGCGAGACCTCCGTGCC 240
i0_HQ_samplec9816d|c33361/f2p4/957      -------------------------------------ctctgaagctgcccctacag--- 20
i0_HQ_samplec9816d|c49271/f4p37/679     ------------------------------------------------------------ 0
NM_003641.3                             ------------------------------------------------------------ 0
```

```
i1_HQ_samplec9816d|c2133/f4p0/1237      CGACCATGTCGTCTGGT-CCCTGTTCAACACCCTCTTCTTGAACTGGTGCTGTCTGGGCT 299
i0_HQ_samplec9816d|c33361/f2p4/957      --gccctggaatcctccccgtgtctcaacaCACTACCCTCTGGGGAGT-----CAGTTCT 73
i0_HQ_samplec9816d|c49271/f4p37/679     ------------------------------------------------------------ 0
NM_003641.3                             ------------------------------------------------------------ 0

i1_HQ_samplec9816d|c2133/f4p0/1237      TCATAGCATTCGCCTACTCCGTGAAGGTGCGTATGGCCCTGGCGGAAATCCAGGGGGTGC 359
i0_HQ_samplec9816d|c33361/f2p4/957      CCCCAGAGTCCCCGTT---------------CCTGGGCCTGGAGGGCTGCTTTGG--GGC 116
i0_HQ_samplec9816d|c49271/f4p37/679     ------------------------------------------------------------ 0
NM_003641.3                             ------------------------------------------------------------ 0

i1_HQ_samplec9816d|c2133/f4p0/1237      CGGTGAGCCTGGGGCTCCACCTGCCCACATGCTGCCTGG---GGTGGGGACTTGTGTGTC 416
i0_HQ_samplec9816d|c33361/f2p4/957      AGGAAACTTTGGCCACCAGGCCTCTGACCTGCACCAGGAGACACTGGGAGGTTTAGTCCC 176
i0_HQ_samplec9816d|c49271/f4p37/679     ------------------------------------------------------------ 0
NM_003641.3                             ------------------------------------------------------------ 0

i1_HQ_samplec9816d|c2133/f4p0/1237      CCTGTGACTGTGAGTTTGTGTGCACCTCTGCCCCGTGTG-------TGCTCACGTCAGTA 469
i0_HQ_samplec9816d|c33361/f2p4/957      CAA---AC----CCGCACAGAGCAGGACTGCAGCCTGAGGAAAGAGCAAGGATTTCAGGA 229
i0_HQ_samplec9816d|c49271/f4p37/679     ------------------------------------------------------------ 0
NM_003641.3                             ------------------------------------------------------------ 0

i1_HQ_samplec9816d|c2133/f4p0/1237      GCTTTGTCTGTGTGATCTGTGTGT--GTGTGTGGCTTTGGGGAATCTGCCCAGTGCAGGT 527
i0_HQ_samplec9816d|c33361/f2p4/957      GAGAGGCCTGCGACAAGTGAGCAGGAAATAGAAACTTAAGA-GAAATACACACTTCTGAG 288
i0_HQ_samplec9816d|c49271/f4p37/679     ---------------------------------------------------tcttctgag 9
NM_003641.3                             ---------------AAACAGCAGGAAATAGAAACTTAAGA-GAAATACACACTTCTGAG 44
                                                                                     * * *

i1_HQ_samplec9816d|c2133/f4p0/1237      CTAGGAGGAGGCTCCAGGAGGCTGGCTGGCTGGCTCAGAGTCTGTCCCTGGCTATCCATT 587
i0_HQ_samplec9816d|c33361/f2p4/957      AAAC--TGAAACGACAGGGGAAAGGAGGTCTCACTGAGCAC-CGTCCCAGCATCCGG--- 342
i0_HQ_samplec9816d|c49271/f4p37/679     aaac--tGAAACGACAGGGGAAAGGAGGTCTCACTGAGCAC-CGTCCCAGCATCCGG--- 63
NM_003641.3                             AAAC--TGAAACGACAGGGGAAAGGAGGTCTCACTGAGCAC-CGTCCCAGCATCCGG--- 98
                                          *    **  *  **** *   **  * **  ** **     ***** *  *

i1_HQ_samplec9816d|c2133/f4p0/1237      AGCCCAGAGCAATTCTCCCTATAGCCCAGTAAGAAATTACACCCTCACCTTCCAGACTGG 647
i0_HQ_samplec9816d|c33361/f2p4/957      ACACCACAGCGGCCCT---TCGCTCC-ACGCAGAAAA-----CCACACTTCTCAAACCTT 393
i0_HQ_samplec9816d|c49271/f4p37/679     ACACCACAGCGGCCCT---TCGCTCC-ACGCAGAAAA-----CCACACTTCTCAAACCTT 114
NM_003641.3                             ACACCACAGCGGCCCT---TCGCTCC-ACGCAGAAAA-----CCACACTTCTCAAACCTT 149
                                        *  *** ***    **   *    ** *   *****      ** *** *  ** **

i1_HQ_samplec9816d|c2133/f4p0/1237      CACCCAGGC---TCTCCCAGAAAGTGAGAAGGGAACTCACAGGTGACTTCACCCCATGGT 704
i0_HQ_samplec9816d|c33361/f2p4/957      CACTCAACACTTCCTTCCCCAAAGCCAGAAGATGCACAAGGAGG---------------- 437
i0_HQ_samplec9816d|c49271/f4p37/679     CACTCAACACTTCCTTCCCCAAAGCCAGAAGATGCACAAGGAGG---------------- 158
NM_003641.3                             CACTCAACACTTCCTTCCCCAAAGCCAGAAGATGCACAAGGAGG---------------- 193
                                        *** **       ** **  ****  *****       *   *

i1_HQ_samplec9816d|c2133/f4p0/1237      GGGGAGAACAGCCTGTGCTGGGGCCAAGGCAGAAGGAGGATGAGCCCCGAGGCTCCTGGA 764
i0_HQ_samplec9816d|c33361/f2p4/957      AACATGAGGTGGCTGTGCTGGGGGCACCCCCCAGCA--------------CCATCCTTCC 483
i0_HQ_samplec9816d|c49271/f4p37/679     AACATGAGGTGGCTGTGCTGGGGGCACCCCCCAGCA--------------CCATCCTTCC 204
NM_003641.3                             AACATGAGGTGGCTGTGCTGGGGGCACCCCCCAGCA--------------CCATCCTTCC 239
                                             **   * *********** **   *  *                    ****

i1_HQ_samplec9816d|c2133/f4p0/1237      GAGTCTGAGCCCGGGTGAGGAAGGGGAGGAGGTGGTCCCTGATCTCAGGGCGGGGAGGAG 824
i0_HQ_samplec9816d|c33361/f2p4/957      AA-GGTCCACCGTGATCAACATCCACAGCGAGACCTCCGTGCCCGACCATGTCGTCTGGT 542
i0_HQ_samplec9816d|c49271/f4p37/679     AA-GGTCCACCGTGATCAACATCCACAGCGAGACCTCCGTGCCCGACCATGTCGTCTGGT 263
NM_003641.3                             AA-GGTCCACCGTGATCAACATCCACAGCGAGACCTCCGTGCCCGACCATGTCGTCTGGT 298
                                         *   *   **  * * *  *     **   *   *** **  *         *   *

i1_HQ_samplec9816d|c2133/f4p0/1237      ACGGAGCCATAGCACGCGGCT--CTCAGCTGGGGGATCCTGGTCCCCTCACCATCTCCTC 882
i0_HQ_samplec9816d|c33361/f2p4/957      CCCTGTTCAACACCCTCTTCTTGAACTGGTGCTGTCTGGGCTTCATAGCATTCGCCTACT 602
i0_HQ_samplec9816d|c49271/f4p37/679     CCCTGTTCAACACCCTCTTCTTGAACTGGTGCTGTCTGGGCTTCATAGCATTCGCCTACT 323
NM_003641.3                             CCCTGTTCAACACCCTCTTCTTGAACTGGTGCTGTCTGGGCTTCATAGCATTCGCCTACT 358
                                         *     **   * * *  **     * * **  *  *     **    **    *

i1_HQ_samplec9816d|c2133/f4p0/1237      TCCCCCAGTCTAGGGACAGGAAGATGGTTGGCGACGTGACCGGGGCCCAGGCCTATGCCT 942
i0_HQ_samplec9816d|c33361/f2p4/957      CCGTGAAGTCTAGGGACAGGAAGATGGTTGGCGACGTGACCGGGGCCCAGGCCTATGCCT 662
i0_HQ_samplec9816d|c49271/f4p37/679     CCGTGAAGTCTAGGGACAGGAAGATGGTTGGCGACGTGACCGGGGCCCAGGCCTATGCCT 383
NM_003641.3                             CCGTGAAGTCTAGGGACAGGAAGATGGTTGGCGACGTGACCGGGGCCCAGGCCTATGCCT 418
                                         *    ******************************************************

i1_HQ_samplec9816d|c2133/f4p0/1237      CCACCGCCAAGTGCCTGAACATCTGGGCCCTGATTCTGGGCATCCTCATGACCATTGGAT 1002
i0_HQ_samplec9816d|c33361/f2p4/957      CCACCGCCAAGTGCCTGAACATCTGGGCCCTGATTCTGGGCATCCTCATGACCATTGGAT 722
i0_HQ_samplec9816d|c49271/f4p37/679     CCACCGCCAAGTGCCTGAACATCTGGGCCCTGATTCTGGGCATCCTCATGACCATTGGAT 443
NM_003641.3                             CCACCGCCAAGTGCCTGAACATCTGGGCCCTGATTCTGGGCATCCTCATGACCATTGGAT 478
                                        ************************************************************

i1_HQ_samplec9816d|c2133/f4p0/1237      TCATCCTGTTACTGGTATTCGGCTCTGTGACAGTCTACCATATTATGTTACAGATAATAC 1062
i0_HQ_samplec9816d|c33361/f2p4/957      TCATCCTGTTACTGGTATTCGGCTCTGTGACAGTCTACCATATTATGTTACAGATAATAC 782
i0_HQ_samplec9816d|c49271/f4p37/679     TCATCCTGTTACTGGTATTCGGCTCTGTGACAGTCTACCATATTATGTTACAGATAATAC 503
NM_003641.3                             TCATCCTGTTACTGGTATTCGGCTCTGTGACAGTCTACCATATTATGTTACAGATAATAC 538
```

```
                                          ************************************************************

i1_HQ_samplec9816d|c2133/f4p0/1237        AGGAAAAACGGGGTTACTAGTAGCCGCCCATAGCCTGCAACCTTTGCACTCCACTGTGCA 1122
i0_HQ_samplec9816d|c33361/f2p4/957        AGGAAAAACGGGGTTACTAGTAGCCGCCCATAGCCTGCAACCTTTGCACTCCACTGTGCA 842
i0_HQ_samplec9816d|c49271/f4p37/679       AGGAAAAACGGGGTTACTAGTAGCCGCCCATAGCCTGCAACCTTTGCACTCCACTGTGCA 563
NM_003641.3                               AGGAAAAACGGGGTTACTAGTAGCCGCCCATAGCCTGCAACCTTTGCACTCCACTGTGCA 598
                                          ************************************************************

i1_HQ_samplec9816d|c2133/f4p0/1237        ATGCTGGCCCTGCACGCTGGGGCTGTTGCCCCTGCCCCCTTGGTCCTGCCCCTAGATACA 1182
i0_HQ_samplec9816d|c33361/f2p4/957        ATGCTGGCCCTGCACGCTGGGGCTGTTGCCCCTGCCCCCTTGGTCCTGCCCCTAGATACA 902
i0_HQ_samplec9816d|c49271/f4p37/679       ATGCTGGCCCTGCACGCTGGGGCTGTTGCCCCTGCCCCCTTGGTCCTGCCCCTAGATACA 623
NM_003641.3                               ATGCTGGCCCTGCACGCTGGGGCTGTTGCCCCTGCCCCCTTGGTCCTGCCCCTAGATACA 658
                                          ************************************************************

i1_HQ_samplec9816d|c2133/f4p0/1237        GCAGTTTATACCCACACACCTGTCTACAGTGTCATTcaataaagtgcacgtgctt----- 1237
i0_HQ_samplec9816d|c33361/f2p4/957        GCAGTTTATACCCACACACCTGTCTACAGTGTCATTCAATAAAGTGCACGTGCTT----- 957
i0_HQ_samplec9816d|c49271/f4p37/679       GCAGTTTATACCCACACACCTGTCTACAGTGTCATTCAATAAAGTGCACGTGCTTG---- 679
NM_003641.3                               GCAGTTTATACCCACACACCTGTCTACAGTGTCATTCAATAAAGTGCACGTGCTTGTGAA 718
                                          *******************************************************

i1_HQ_samplec9816d|c2133/f4p0/1237        ---------------     1237
i0_HQ_samplec9816d|c33361/f2p4/957        ---------------     957
i0_HQ_samplec9816d|c49271/f4p37/679       ---------------     679
NM_003641.3                               AAAAAAAAAAAAAAA     733
```

```
VIRT175835      ---------MLGRCSV--RPPFPCRFSFSEVCISLKFLFPAHLSQASLLKSLLFPQAAVL     49
VIRT168080      ---------MVGHGGLAVDVDHGGPWKDG------AGGCPQHSHLMFLLVHLLALG-KEV     44
VIRT177472      ---------MVGHGGLAVDVDHGGPWKDG------AGGCPQHSHLMFLLVHLLALG-KEV     44
NP_003632.3     MHKEEHEVAVLGAPPSTILPRSTVINIHS------ETSVPD--HVVWSLFNTLFLN---W     49
                         ::*                .          *        *   *

VIRT175835      LCAGLGTKPPSVSWCRSEAWWPKFPAPKQPSRPRNGDS-GEN------------------     90
VIRT168080      LSEGLRS----VVFCV----------E-RRAAVVSGCWDGAQ------------------     71
VIRT177472      LSEGLRS----VVFCV----------E-RRAAVVSGCWDGAQ------------------     71
NP_003632.3     CCLGFIA----FAYSV----------K-SRDRKMVGDVTGAQAYASTAKCLNIWALILGI     94
                 . *: :    . :.                    *   * :

VIRT175835      -------------------------------   90
VIRT168080      -------------------------------   71
VIRT177472      -------------------------------   71
NP_003632.3     LMTIGFILLLVFGSVTVYHIMLQIIQEKRGY   125
```

## IFITM3

| Sequence | Header | AA Seq | |
|---|---|---|---|
| **Present** | i1_HQ_samplec9816d\|c8447/f5p0/1206 | VIRT11976 | |
| **Absent** | i0_HQ_samplec9816d\|c108452/f2p0/628 | | |
| | | | |
| **NCBI** | NM_021034 | NP_066362.2 | |
| | NR_049759 | >VIRT13170 | |

>i1_HQ_samplec9816d|c8447/f5p0/1206

isoform=c8447;full_length_coverage=5;non_full_length_coverage=0;isoform_length=1206

actggggaaagggagggctcactgagaaccatcccagtaacccgaccgccgctggtcTTCGCTGGACACCATGAATCAAACTGTCCAAACC
TTCTTCTCTCCTGTCAACAGCGGCCAGCCCCCCAACTATGAGATGCTCAAGGAGGAGCACGAGGTGGCTGTGCTGGGG
GCGCCCCACAACCCTGCTCCCCCGACGTCCACCGTGATCCACATCCGCAGCGAGACCTCCGTGCCCGACCATGTCGTCTG
GTCCCTGTTCAACACCCTCTTCATGAACCCCTGCTGCCTGGGCTTCATAGCATTCGCCTACTCCGTGAAGGTGCGTATGG
CCCCAGGGAATGCTCAGAGGGTGCCGCTGAGCCTGGAGCTCCACCTGCCCACATGCTGCCTGGGGTGGGGACTTGTGT
GTCCCTGTGACTGTGAGTTTGTGTGCACCTCTGTCCCGTGTGTGCCCACGTCAGTGGCTTTGTCTGTGTGATCTGTGTGT
GTGTGTGGCTTGGGGAATCTGCCCAGTGCAGGTTTAGGAGGAGGCTCCAGGAGGCTGGCTGGCTGGCTCAGAGTCTG
TCCCCGGCTATCCACTAGCCCAGAGCAGTTCTCCCTATAGCCCAGTAAGAAATTACACCTTCACCTTCCAGACTGGCACC
CACGCTCTCCCAGAAAGTGAGAAGGGAACTCACAGGTGACTTCACCCCATGGTGGGGAGAACAGCCTGTGCTGGGGTC
AAGGCAGAAGGAGGATGAGCCCCGAGGCTCCTGGAGAGTCTGAGCCTGGGTGAGGAAGGGGAGGAGGTGGTCCCTG

ATCTCAGGGCGGGGAGAGCCAATGAGGAGACGGAGCCATAGCACGCGGCTCTCAGCTGGGGGATCCTGGTCCCCTCA
CCATCTCCTCTCCCCCAGTCTAGGGACAGGAAGATGGTTGGCGACGTGACCGGGGCCCAGGCCTATGCCTCCACCGCCA
AGTGCCTGAACATCTGGGCCCTGATTCTGGGCATCCTCATGACCATTCTGCTCATCGTCATCCCAGTGCTGATCTTCCAG
GCCTATGGATAGATCAGGAGGCATCACTGAGGCCAGGAGCTCTGCCCATGACCTGTATCCCACGTACTCCAACTTCCAT
TCCTCGCCCTGCCCCCGGAGCCGAGTCCTGTATCAGCCCTTTATCCTCACACGCTTTTctacaatggcattcaataaagtgcacgtgt
ttctg

>VIRT11976

MNQTVQTFFSPVNSGQPPNYEMLKEEHEVAVLGAPHNPAPPTSTVIHIRSETSVPDHVVW
SLFNTLFMNPCCLGFIAFAYSVKVRMAPGNAQRVPLSLELHLPTCCLGWGLVCPCDCEFV
CTSVPCVPTSVALSV

>i0_HQ_samplec9816d|c108452/f2p0/628
isoform=c108452;full_length_coverage=2;non_full_length_coverage=0;isoform_length=628

gaaaagggagggctcactgagaaccatcccagtaacccgaccgccgctggtcttcgctggACACCATGAATCAAACTGTCCAAACCTTCTTCT
CTCCTGTCAACAGCGGCCAGCCCCCCAACTATGAGATGCTCAAGGAGGAGCACGAGGTGGCTGTGCTGGGGGCGCCCC
ACAACCCTGCTCCCCCGACGTCCACCGTGATCCACATCCGCAGCGAGACCTCCGTGCCCGACCATGTCGTCTGGTCCCTG
TTCAACACCCTCTTCATGAACCCCTGCTGCCTGGGCTTCATAGCATTCGCCTACTCCGTGAAGTCTAGGGACAGGAAGAT
GGTTGGCGACGTGACCGGGGCCCAGGCCTATGCCTCCACCGCCAAGTGCCTGAACATCTGGGCCCTGATTCTGGGCAT
CCTCATGACCATTCTGCTCATCGTCATCCCAGTGCTGATCTTCCAGGCCTATGGATAGATCAGGAGGCATCACTGAGGCC
AGGAGCTCTGCCCATGACCTGTATCCCACGTACTCCAACTTCCATTCCTCGCCCTGCCCCCGGAGCCGAGTCCTGTATCA
GCCCTTTATCCTCACACGCTTTTCTACAATGGCATTCAataaagtgcacgtgtttctgg

>VIRT13170
MNQTVQTFFSPVNSGQPPNYEMLKEEHEVAVLGAPHNPAPPTSTVIHIRSETSVPDHVVW
SLFNTLFMNPCCLGFIAFAYSVKSRDRKMVGDVTGAQAYASTAKCLNIWALILGILMTIL
LIVIPVLIFQAYG

>NM_021034.2 Homo sapiens interferon induced transmembrane protein 3 (IFITM3), transcript variant 1, mRNA
AGGAAAAGGAAACTGTTGAGAAACCGAAACTACTGGGGAAAGGGAGGGCTCACTGAGAACCATCCCAGTA
ACCCGACCGCCGCTGGTCTTCGCTGGACACCATGAATCACACTGTCCAAACCTTCTTCTCTCCTGTCAAC
AGTGGCCAGCCCCCCAACTATGAGATGCTCAAGGAGGAGCACGAGGTGGCTGTGCTGGGGGCGCCCCACA
ACCCTGCTCCCCCGACGTCCACCGTGATCCACATCCGCAGCGAGACCTCCGTGCCCGACCATGTCGTCTG
GTCCCTGTTCAACACCCTCTTCATGAACCCCTGCTGCCTGGGCTTCATAGCATTCGCCTACTCCGTGAAG
TCTAGGGACAGGAAGATGGTTGGCGACGTGACCGGGGCCCAGGCCTATGCCTCCACCGCCAAGTGCCTGA
ACATCTGGGCCCTGATTCTGGGCATCCTCATGACCATTCTGCTCATCGTCATCCCAGTGCTGATCTTCCA
GGCCTATGGATAGATCAGGAGGCATCACTGAGGCCAGGAGCTCTGCCCATGACCTGTATCCCACGTACTC
CAACTTCCATTCCTCGCCCTGCCCCCGGAGCCGAGTCCTGTATCAGCCCTTTATCCTCACACGCTTTTCT
ACAATGGCATTCAATAAAGTGCACGTGTTTCTGGTGCTAAAAAAAAAA

>NP_066362.2 interferon-induced transmembrane protein 3 [Homo sapiens]
MNHTVQTFFSPVNSGQPPNYEMLKEEHEVAVLGAPHNPAPPTSTVIHIRSETSVPDHVVWSLFNTLFMNP
CCLGFIAFAYSVKSRDRKMVGDVTGAQAYASTAKCLNIWALILGILMTILLIVIPVLIFQAYG

>NR_049759.1 Homo sapiens interferon induced transmembrane protein 3 (IFITM3), transcript variant 2, non-coding RNA
AGGAAAAGGAAACTGTTGAGAAACCGAAACTACTGGGGAAAGGGAGGGCTCACTGAGAACCATCCCAGTA
ACCCGACCGCCGCTGGTCTTCGCTGGACACCATGAATCACACTGTCCAAACCTTCTTCTCTCCTGTCAAC
AGTGGCCAGCCCCCCAACTATGAGATGCTCAAGGAGGAGCACGAGGTGGCTGTGCTGGGGGCGCCCCACA
ACCCTGCTCCCCCGACGTCCACCGTGATCCACATCCGCAGCGAGACCTCCGTGCCCGACCATGTCGTCTG
GTCCCTGTTCAACACCCTCTTCATGAACCCCTGCTGCCTGGGCTTCATAGCATTCGCCTACTCCGTGAAG
GGACAGGAAGATGGTTGGCGACGTGACCGGGGCCCAGGCCTATGCCTCCACCGCCAAGTGCCTGAACATC
TGGGCCCTGATTCTGGGCATCCTCATGACCATTCTGCTCATCGTCATCCCAGTGCTGATCTTCCAGGCCT
ATGGATAGATCAGGAGGCATCACTGAGGCCAGGAGCTCTGCCCATGACCTGTATCCCACGTACTCCAACT

```
TCCATTCCTCGCCCTGCCCCCGGAGCCGAGTCCTGTATCAGCCCTTTATCCTCACACGCTTTTCTACAAT
GGCATTCAATAAAGTGCACGTGTTTCTGGTGCTAAAAAAAAAA
```

```
i1_HQ_samplec9816d|c8447/f5p0/1206      -------------------------------actggggaaagggagggctcactgagaac 29
NR_049759.1                             AGGAAAAGGAAACTGTTGAGAAACCGAAACTACTGGGGAAAGGGAGGGCTCACTGAGAAC 60
i0_HQ_samplec9816d|c108452/f2p0/628     ------------------------------------gaaaagggagggctcactgagaac 24
NM_021034.2                             AGGAAAAGGAAACTGTTGAGAAACCGAAACTACTGGGGAAAGGGAGGGCTCACTGAGAAC 60
                                                                            * **********************

i1_HQ_samplec9816d|c8447/f5p0/1206      catcccagtaacccgaccgccgctggtcTTCGCTGGACACCATGAATCAAACTGTCCAAA 89
NR_049759.1                             CATCCCAGTAACCCGACCGCCGCTGGTCTTCGCTGGACACCATGAATCACACTGTCCAAA 120
i0_HQ_samplec9816d|c108452/f2p0/628     catcccagtaacccgaccgccgctggtcttcgctggACACCATGAATCAAACTGTCCAAA 84
NM_021034.2                             CATCCCAGTAACCCGACCGCCGCTGGTCTTCGCTGGACACCATGAATCACACTGTCCAAA 120
                                        ************************************************* **********

i1_HQ_samplec9816d|c8447/f5p0/1206      CCTTCTTCTCTCCTGTCAACAGCGGCCAGCCCCCCAACTATGAGATGCTCAAGGAGGAGC 149
NR_049759.1                             CCTTCTTCTCTCCTGTCAACAGTGGCCAGCCCCCCAACTATGAGATGCTCAAGGAGGAGC 180
i0_HQ_samplec9816d|c108452/f2p0/628     CCTTCTTCTCTCCTGTCAACAGCGGCCAGCCCCCCAACTATGAGATGCTCAAGGAGGAGC 144
NM_021034.2                             CCTTCTTCTCTCCTGTCAACAGTGGCCAGCCCCCCAACTATGAGATGCTCAAGGAGGAGC 180
                                        ********************** *************************************

i1_HQ_samplec9816d|c8447/f5p0/1206      ACGAGGTGGCTGTGCTGGGGGCGCCCCACAACCCTGCTCCCCCGACGTCCACCGTGATCC 209
NR_049759.1                             ACGAGGTGGCTGTGCTGGGGGCGCCCCACAACCCTGCTCCCCCGACGTCCACCGTGATCC 240
i0_HQ_samplec9816d|c108452/f2p0/628     ACGAGGTGGCTGTGCTGGGGGCGCCCCACAACCCTGCTCCCCCGACGTCCACCGTGATCC 204
NM_021034.2                             ACGAGGTGGCTGTGCTGGGGGCGCCCCACAACCCTGCTCCCCCGACGTCCACCGTGATCC 240
                                        ************************************************************

i1_HQ_samplec9816d|c8447/f5p0/1206      ACATCCGCAGCGAGACCTCCGTGCCCGACCATGTCGTCTGGTCCCTGTTCAACACCCTCT 269
NR_049759.1                             ACATCCGCAGCGAGACCTCCGTGCCCGACCATGTCGTCTGGTCCCTGTTCAACACCCTCT 300
i0_HQ_samplec9816d|c108452/f2p0/628     ACATCCGCAGCGAGACCTCCGTGCCCGACCATGTCGTCTGGTCCCTGTTCAACACCCTCT 264
NM_021034.2                             ACATCCGCAGCGAGACCTCCGTGCCCGACCATGTCGTCTGGTCCCTGTTCAACACCCTCT 300
                                        ************************************************************

i1_HQ_samplec9816d|c8447/f5p0/1206      TCATGAACCCCTGCTGCCTGGGCTTCATAGCATTCGCCTACTCCGTGAAGGTGCGTATGG 329
NR_049759.1                             TCATGAACCCCTGCTGCCTGGGCTTCATAGCATTCGCCTA-------------------- 340
i0_HQ_samplec9816d|c108452/f2p0/628     TCATGAACCCCTGCTGCCTGGGCTTCATAGCATTCGCCTA-------------------- 304
NM_021034.2                             TCATGAACCCCTGCTGCCTGGGCTTCATAGCATTCGCCTA-------------------- 340
                                        ****************************************

i1_HQ_samplec9816d|c8447/f5p0/1206      CCCCAGGGAATGCTCAGAGGGTGCCGCTGAGCCTGGAGCTCCACCTGCCCACATGCTGCC 389
NR_049759.1                             ------------------------------------------------------------ 340
i0_HQ_samplec9816d|c108452/f2p0/628     ------------------------------------------------------------ 304
NM_021034.2                             ------------------------------------------------------------ 340

i1_HQ_samplec9816d|c8447/f5p0/1206      TGGGGTGGGGACTTGTGTGTCCCTGTGACTGTGAGTTTGTGTGCACCTCTGTCCCGTGTG 449
NR_049759.1                             ------------------------------------------------------------ 340
i0_HQ_samplec9816d|c108452/f2p0/628     ------------------------------------------------------------ 304
NM_021034.2                             ------------------------------------------------------------ 340

i1_HQ_samplec9816d|c8447/f5p0/1206      TGCCCACGTCAGTGGCTTTGTCTGTGTGATCTGTGTGTGTGTGTGGCTTGGGGAATCTGC 509
NR_049759.1                             ------------------------------------------------------------ 340
i0_HQ_samplec9816d|c108452/f2p0/628     ------------------------------------------------------------ 304
NM_021034.2                             ------------------------------------------------------------ 340

i1_HQ_samplec9816d|c8447/f5p0/1206      CCAGTGCAGGTTTAGGAGGAGGCTCCAGGAGGCTGGCTGGCTGGCTCAGAGTCTGTCCCC 569
NR_049759.1                             ------------------------------------------------------------ 340
i0_HQ_samplec9816d|c108452/f2p0/628     ------------------------------------------------------------ 304
NM_021034.2                             ------------------------------------------------------------ 340

i1_HQ_samplec9816d|c8447/f5p0/1206      GGCTATCCACTAGCCCAGAGCAGTTCTCCCTATAGCCCAGTAAGAAATTACACCTTCACC 629
NR_049759.1                             ------------------------------------------------------------ 340
i0_HQ_samplec9816d|c108452/f2p0/628     ------------------------------------------------------------ 304
NM_021034.2                             ------------------------------------------------------------ 340

i1_HQ_samplec9816d|c8447/f5p0/1206      TTCCAGACTGGCACCCACGCTCTCCCAGAAAGTGAGAAGGGAACTCACAGGTGACTTCAC 689
NR_049759.1                             ------------------------------------------------------------ 340
i0_HQ_samplec9816d|c108452/f2p0/628     ------------------------------------------------------------ 304
NM_021034.2                             ------------------------------------------------------------ 340

i1_HQ_samplec9816d|c8447/f5p0/1206      CCCATGGTGGGGAGAACAGCCTGTGCTGGGGTCAAGGCAGAAGGAGGATGAGCCCCGAGG 749
NR_049759.1                             ------------------------------------------------------------ 340
```

```
i0_HQ_samplec9816d|c108452/f2p0/628       ------------------------------------------------------------ 304
NM_021034.2                               ------------------------------------------------------------ 340

i1_HQ_samplec9816d|c8447/f5p0/1206        CTCCTGGAGAGTCTGAGCCTGGGTGAGGAAGGGGAGGAGGTGGTCCCTGATCTCAGGGCG 809
NR_049759.1                               ------------------------------------------------------------ 340
i0_HQ_samplec9816d|c108452/f2p0/628       ------------------------------------------------------------ 304
NM_021034.2                               ------------------------------------------------------------ 340

i1_HQ_samplec9816d|c8447/f5p0/1206        GGGAGAGCCAATGAGGAGACGGAGCCATAGCACGCGGCTCTCAGCTGGGGGATCCTGGTC 869
NR_049759.1                               ------------------------------------------------------------ 340
i0_HQ_samplec9816d|c108452/f2p0/628       ------------------------------------------------------------ 304
NM_021034.2                               ------------------------------------------------------------ 340

i1_HQ_samplec9816d|c8447/f5p0/1206        CCCTCACCATCTCCTCTCCCCCAGTCTAGGGACAGGAAGATGGTTGGCGACGTGACCGGG 929
NR_049759.1                               --------------CTCCGTGAA-----GGGACAGGAAGATGGTTGGCGACGTGACCGGG 381
i0_HQ_samplec9816d|c108452/f2p0/628       --------------CTCCGTGAAGTCTAGGGACAGGAAGATGGTTGGCGACGTGACCGGG 350
NM_021034.2                               --------------CTCCGTGAAGTCTAGGGACAGGAAGATGGTTGGCGACGTGACCGGG 386
                                                           *    *     ********************************

i1_HQ_samplec9816d|c8447/f5p0/1206        GCCCAGGCCTATGCCTCCACCGCCAAGTGCCTGAACATCTGGGCCCTGATTCTGGGCATC 989
NR_049759.1                               GCCCAGGCCTATGCCTCCACCGCCAAGTGCCTGAACATCTGGGCCCTGATTCTGGGCATC 441
i0_HQ_samplec9816d|c108452/f2p0/628       GCCCAGGCCTATGCCTCCACCGCCAAGTGCCTGAACATCTGGGCCCTGATTCTGGGCATC 410
NM_021034.2                               GCCCAGGCCTATGCCTCCACCGCCAAGTGCCTGAACATCTGGGCCCTGATTCTGGGCATC 446
                                          ************************************************************

i1_HQ_samplec9816d|c8447/f5p0/1206        CTCATGACCATTCTGCTCATCGTCATCCCAGTGCTGATCTTCCAGGCCTATGGATAGATC 1049
NR_049759.1                               CTCATGACCATTCTGCTCATCGTCATCCCAGTGCTGATCTTCCAGGCCTATGGATAGATC 501
i0_HQ_samplec9816d|c108452/f2p0/628       CTCATGACCATTCTGCTCATCGTCATCCCAGTGCTGATCTTCCAGGCCTATGGATAGATC 470
NM_021034.2                               CTCATGACCATTCTGCTCATCGTCATCCCAGTGCTGATCTTCCAGGCCTATGGATAGATC 506
                                          ************************************************************

i1_HQ_samplec9816d|c8447/f5p0/1206        AGGAGGCATCACTGAGGCCAGGAGCTCTGCCCATGACCTGTATCCCACGTACTCCAACTT 1109
NR_049759.1                               AGGAGGCATCACTGAGGCCAGGAGCTCTGCCCATGACCTGTATCCCACGTACTCCAACTT 561
i0_HQ_samplec9816d|c108452/f2p0/628       AGGAGGCATCACTGAGGCCAGGAGCTCTGCCCATGACCTGTATCCCACGTACTCCAACTT 530
NM_021034.2                               AGGAGGCATCACTGAGGCCAGGAGCTCTGCCCATGACCTGTATCCCACGTACTCCAACTT 566
                                          ************************************************************

i1_HQ_samplec9816d|c8447/f5p0/1206        CCATTCCTCGCCCTGCCCCCGGAGCCGAGTCCTGTATCAGCCCTTTATCCTCACACGCTT 1169
NR_049759.1                               CCATTCCTCGCCCTGCCCCCGGAGCCGAGTCCTGTATCAGCCCTTTATCCTCACACGCTT 621
i0_HQ_samplec9816d|c108452/f2p0/628       CCATTCCTCGCCCTGCCCCCGGAGCCGAGTCCTGTATCAGCCCTTTATCCTCACACGCTT 590
NM_021034.2                               CCATTCCTCGCCCTGCCCCCGGAGCCGAGTCCTGTATCAGCCCTTTATCCTCACACGCTT 626
                                          ************************************************************

i1_HQ_samplec9816d|c8447/f5p0/1206        TTctacaatggcattcaataaagtgcacgtgtttctg---------------         1206
NR_049759.1                               TTCTACAATGGCATTCAATAAAGTGCACGTGTTTCTGGTGCTAAAAAAAAAA         673
i0_HQ_samplec9816d|c108452/f2p0/628       TTCTACAATGGCATTCAataaagtgcacgtgtttctgg--------------         628
NM_021034.2                               TTCTACAATGGCATTCAATAAAGTGCACGTGTTTCTGGTGCTAAAAAAAAAA         678
                                          *************************************
```

```
VIRT11976          MNQTVQTFFSPVNSGQPPNYEMLKEEHEVAVLGAPHNPAPPTSTVIHIRSETSVPDHVVW     60
VIRT13170          MNQTVQTFFSPVNSGQPPNYEMLKEEHEVAVLGAPHNPAPPTSTVIHIRSETSVPDHVVW     60
NP_066362.2        MNHTVQTFFSPVNSGQPPNYEMLKEEHEVAVLGAPHNPAPPTSTVIHIRSETSVPDHVVW     60
                   **:*********************************************************

VIRT11976          SLFNTLFMNPCCLGFIAFAYSVKVRMAPGNAQRVPLSLELHLPTCCLGWGLVCPCDCEFV     120
VIRT13170          SLFNTLFMNPCCLGFIAFAYSVKSRDKMVGDVTGAQAYASTAKCLNIWALILGILMTIL     120
NP_066362.2        SLFNTLFMNPCCLGFIAFAYSVKSRDKMVGDVTGAQAYASTAKCLNIWALILGILMTIL     120
                   ***********************  *     .:  .  .      .*   *.*:      ::

VIRT11976          CTSVPCVPTSVALSV     135
VIRT13170          LIVIPVLIFQA-YG-     133
NP_066362.2        LIVIPVLIFQA-YG-     133
                      :* :  ..  .
```

## GSTP1

| Sequence | Header | AA Seq |
|---|---|---|
| **Present** | i1_HQ_samplec9816d\|c1892/f2p5/1103 | VIRT85086 |
| **Absent** | i0_HQ_samplec9816d\|c3248/f3p6/738 | VIRT85693 |
| **NCBI** | NM_000852 | NP_000843.1 |
| | | |

>i1_HQ_samplec9816d|c1892/f2p5/1103
isoform=c1892;full_length_coverage=2;non_full_length_coverage=5;isoform_length=1103

gctggagtttcgccgccgcagtcttcgccaccatgccgccctaacccgtggtctatttcccaGTTCGAGGCCGCTGCGCGGCCCTGCGCATGCT
GCTGGCAGATCAGGGCCAGAGCTGGAAGGAGGAGGTGGTGACCGTGGAGACGTGGCAGGAGGGCTCACTCAAAGCC
TCCTGCCTATACGGGCAGCTCCCCAAGTTCCAGGACGGAGACCTCACCCTGTACCAGTCCAATACCATCCTGCGTCACCT
GGGCCGCACCCTTGGTGAGTCTTGAACCTCCAAGTCCAGGGCAGGCATGGGCAAGCCTCTGCCCCCGGAGCCCTTTTGT
TTAAATCAGCTGCCCCGCAGCCCTCTGGAGTGGAGGAAACTGAGACCCACTGAGGTTACGTAGTTTGCCCAAGGTCAA
GCCTGGGTGCCTGCAATCCTTGCCCTGTGCCAGGCTGCCTCCCAGGTGTCAGGTGAGCTCTGAGCACCTGCTGTGTGGC
AGTCTCTCATCCTTCCACGCACATCCTCTTCCCCTCCTCCCAGGCTGGGGCTCACAGACAGCCCCCTGGTTGGCCCATCCC
CAGTGACTGTGTGTTGATCAGGCGCCCAGTCACGCGGCCTGCTCCCCTCCACCCAACCCCAGGGCTCTATGGGAAGGAC
CAGCAGGAGGCAGCCCTGGTGGACATGGTGAATGACGGCGTGGAGGACCTCCGCTGCAAATACATCTCCCTCATCTAC
ACCAACTATGAGGCGGGCAAGGATGACTATGTGAAGGCACTGCCCGGGCAACTGAAGCCTTTTGAGACCCTGCTGTCC
CAGAACCAGGGAGGCAAGACCTTCATTGTGGGAGACCAGATCTCCTTCGCTGACTACAACCTGCTGGACTTGCTGCTGA
TCCATGAGGTCCTAGCCCCTGGCTGCCTGGATGCGTTCCCCCTGCTCTCAGCATATGTGGGGCGCCTCAGTGCCCGGCC
CAAGCTCAAGGCCTTCCTGGCCTCCCCTGAGTACGTGAACCTCCCCATCAATGGCAACGGGAAACAGTGAGGGTTGGG
GGACTCTGAGCGGGAGGCAGAGTTTGCCTTCctttctccaggaccaataaaatttctaagagag

>VIRT85086

MDQQQVQQVVVSEGDLVSHNEGLASLVLGQQGLKRLQLPGQCLHIVILARLIVGVDEGDV
FAAEVLHAVIHHVHQGCLLLVLPIEPWGWVEGSRPRDWAPDQHTVTGDGPTRGLSVSPSL
GGGEEDVRGRMRDCHTAGAQSSPDTWEAAWHRARIAGTQA

>i0_HQ_samplec9816d|c3248/f3p6/738
isoform=c3248;full_length_coverage=3;non_full_length_coverage=6;isoform_length=738

gctGGAGTTTCGCCGCCGCAGTCTTCGCCACCATGCCGCCCTACACCGTGGTCTATTTCCCAGTTCGAGGCCGCTGCGCG
GCCCTGCGCATGCTGCTGGCAGATCAGGGCCAGAGCTGGAAGGAGGAGGTGGTGACCGTGGAGACGTGGCAGGAGG
GCTCACTCAAAGCCTCCTGCCTATACGGGCAGCTCCCCAAGTTCCAGGACGGAGACCTCACCCTGTACCAGTCCAATACC
ATCCTGCGTCACCTGGGCCGCACCCTTGGGCTCTATGGGAAGGACCAGCAGGAGGCAGCCCTGGTGGACATGGTGAAT
GACGGCGTGGAGGACCTCCGCTGCAAATACGTCTCCCTCATCTACACCAACTATGAGGCGGGCAAGGATGACTATGTG
AAGGCACTGCCCGGGCAACTGAAGCCTTTTGAGACCCTGCTGTCCCAGAACCAGGGAGGCAAGACCTTCATTGTGGGA
GACCAGATCTCCTTCGCTGACTACAACCTGCTGGACTTGCTGCTGATCCATGAGGTCCTAGCCCCTGGCTGCCTGGATGC
GTTCCCCCTGCTCTCAGCATATGTGGGGCGCCTCAGTGCCCGGCCCAAGCTCAAGGCCTTCCTGGCCTCCCCTGAGTAC
GTGAACCTCCCCATCAATGGCAACGGGAAACAGTGAGGGTTGGGGGGACTCTGAGCGGGAGGCAGAGTTTGCCTTCC
TTTCTCCAGGACCAATAAAATTTCTAAGAGAG

>VIRT85693

MDQQQVQQVVVSEGDLVSHNEGLASLVLGQQGLKRLQLPGQCLHIVILARLIVGVDEGDV
FAAEVLHAVIHHVHQGCLLLVLPIEPKGAAQVTQDGIGLVQGEVSVLELGELPV

>NM_000852.3 Homo sapiens glutathione S-transferase pi 1 (GSTP1), mRNA
TGGGAAAGAGGGAAAGGCTTCCCCGGCCAGCTGCGCGGCGACTCCGGGGACTCCAGGGCGCCCCTCTGCG
GCCGACGCCCGGGGTGCAGCGGCCGCCGGGGCTGGGGCCGGCGGGAGTCCGCGGACCCTCCAGAAGAGC
GGCCGGCGCCGTGACTCAGCACTGGGGCGGAGCGGGGCGGGACCACCCTTATAAGGCTCGGAGGCCGCGA
GGCCTTCGCTGGAGTTTCGCCGCCGCAGTCTTCGCCACCATGCCGCCCTACACCGTGGTCTATTTCCCAG
TTCGAGGCCGCTGCGCGGCCCTGCGCATGCTGCTGGCAGATCAGGGCCAGAGCTGGAAGGAGGAGGTGGT

```
GACCGTGGAGACGTGGCAGGAGGGCTCACTCAAAGCCTCCTGCCTATACGGGCAGCTCCCCAAGTTCCAG
GACGGAGACCTCACCCTGTACCAGTCCAATACCATCCTGCGTCACCTGGGCCGCACCCTTGGGCTCTATG
GGAAGGACCAGCAGGAGGCAGCCCTGGTGGACATGGTGAATGACGGCGTGGAGGACCTCCGCTGCAAATA
CATCTCCCTCATCTACACCAACTATGAGGCGGGCAAGGATGACTATGTGAAGGCACTGCCCGGGCAACTG
AAGCCTTTTGAGACCCTGCTGTCCCAGAACCAGGGAGGCAAGACCTTCATTGTGGGAGACCAGATCTCCT
TCGCTGACTACAACCTGCTGGACTTGCTGCTGATCCATGAGGTCCTAGCCCCTGGCTGCCTGGATGCGTT
CCCCCTGCTCTCAGCATATGTGGGGCGCCTCAGTGCCCGGCCCAAGCTCAAGGCCTTCCTGGCCTCCCCT
GAGTACGTGAACCTCCCCATCAATGGCAACGGGAAACAGTGAGGGTTGGGGGGACTCTGAGCGGGAGGCA
GAGTTTGCCTTCCTTTCTCCAGGACCAATAAAATTTCTAAGAGAGCTAAAAAAAAAAAAAAAAAAAAAAA
AAAAAA
```

```
>NP_000843.1 glutathione S-transferase P [Homo sapiens]
MPPYTVVYFPVRGRCAALRMLLADQGQSWKEEVVTVETWQEGSLKASCLYGQLPKFQDGDLTLYQSNTIL
RHLGRTLGLYGKDQQEAALVDMVNDGVEDLRCKYISLIYTNYEAGKDDYVKALPGQLKPFETLLSQNQGG
KTFIVGDQISFADYNLLDLLLIHEVLAPGCLDAFPLLSAYVGRLSARPKLKAFLASPEYVNLPINGNGKQ
```

```
i1_HQ_samplec9816d|c1892/f2p5/1103      gctggagtttcgccgccgcagtcttcgccaccatgccgccctaacccgtggtctatttcc  60
i0_HQ_samplec9816d|c3248/f3p6/738       ------------------------------------------------------------  0
NM_000852.3                             ------------------------------------------------------------  0

i1_HQ_samplec9816d|c1892/f2p5/1103      caGTTCGAGGCCGCTGCGCGGCCCTGCGCATGCTGCTGGCAGATCAGGGCCAGAGCTGGA  120
i0_HQ_samplec9816d|c3248/f3p6/738       ------------------------------------------------------------  0
NM_000852.3                             -------------------------------------------------------TGGGA  5

i1_HQ_samplec9816d|c1892/f2p5/1103      AGGAGGAGGTGGTGACCGTGGAGACGTGGCAG----GAGGGCTCACTCAAAGCCTCCTGC  176
i0_HQ_samplec9816d|c3248/f3p6/738       ------------------------------------------------------------  0
NM_000852.3                             AAGAGGGAAAGGCTTCCCCGGCCAGCTGCGCGGCGACTCCGGGGACTCCAGGGCGC--CC  63

i1_HQ_samplec9816d|c1892/f2p5/1103      CTATACGGGCAGCTCCCCAAGTTCCAGGACGGA---GACCTCACCCTGTACCAGTCCAAT  233
i0_HQ_samplec9816d|c3248/f3p6/738       ------------------------------------------------------------  0
NM_000852.3                             CTCTGCGGCCGACGCCCGGGGTGCAGCGGCCGCCGGGGCTGGGGCCGGCGGGAGTCCGCG  123

i1_HQ_samplec9816d|c1892/f2p5/1103      ACCATCCTGCGTCACC-TGGGCCGCACCCTTGGTGAGTCTTGAACCTCCAAGTCCAGGGC  292
i0_HQ_samplec9816d|c3248/f3p6/738       ------------------------------------------------------------  0
NM_000852.3                             G-GACCCTCCAGAAGAGCGGCCGGCGCCGTGACTCAGCACTGGGGCGGAGCGGGGCGGGA  182

i1_HQ_samplec9816d|c1892/f2p5/1103      AGGCATGGGCAAGCCTCTGCCC-CCGGAGCCCTTTTGTTTAAATCAGCTGCCCCGCAGCC  351
i0_HQ_samplec9816d|c3248/f3p6/738       -----------------------------------gctGGAGTTTC-----GCCGCCGCA  20
NM_000852.3                             CCACCCTTATAAGGCTCGGAGGCCGCGAGGCCTTCGCTGGAGTTTC-----GCCGCCGCA  237
                                                                             *  *  *        **** **

i1_HQ_samplec9816d|c1892/f2p5/1103      CTCTGGAGTGGAGGAAACTGAGACCCACTGAGGTTACGTAGTTTGCCCAAGGTCAAGCCT  411
i0_HQ_samplec9816d|c3248/f3p6/738       GTCT-------TCGCCACCATGCCGCCCTACACCGTGGTCTATTTCC-CAGTTCGAGGCC  72
NM_000852.3                             GTCT-------TCGCCACCATGCCGCCCTACACCGTGGTCTATTTCC-CAGTTCGAGGCC  289
                                         ***         *  **   * * * **        **   ** **  ** ** ** *

i1_HQ_samplec9816d|c1892/f2p5/1103      GGGTGCCTGCAATCCTTGCCCTGTGCCAGGCTGC--CTCCCAGGTGTCAGGTGAGCTCTG  469
i0_HQ_samplec9816d|c3248/f3p6/738       GCTGCGCGGCCCTGCGCATGCTGCTGGCAGATCAGGGCCAGAGCTGGAAGGAGGAGGTGG  132
NM_000852.3                             GCTGCGCGGCCCTGCGCATGCTGCTGGCAGATCAGGGCCAGAGCTGGAAGGAGGAGGTGG  349
                                        *     * **  * *     ***      * *      *  ** **  *** *      *

i1_HQ_samplec9816d|c1892/f2p5/1103      AGCACCTGCTGTGTGGCAGTCTCTCATCCTTCCACGCACATCCTCTTCCCCTCCTCCCAG  529
i0_HQ_samplec9816d|c3248/f3p6/738       TGAC-----CGTGGAGACGTGGCAGGAGGGCTCA--------CTCAAAGCCTCCTGCCTA  179
NM_000852.3                             TGAC-----CGTGGAGACGTGGCAGGAGGGCTCA--------CTCAAAGCCTCCTGCCTA  396
                                         *        ***  *  **  *         **        ***    ****** **

i1_HQ_samplec9816d|c1892/f2p5/1103      GCTGGGGCTCACAGACAGCCCCCTGGTTGGCCCATCCCCAGTGACTGTGTGTTGATCAGG  589
i0_HQ_samplec9816d|c3248/f3p6/738       TACGGGCAGCTCCCCAAGTTCCAG---------------GACGGAGACCTCACCCTGTAC  224
NM_000852.3                             TACGGGCAGCTCCCCAAGTTCCAG---------------GACGGAGACCTCACCCTGTAC  441
                                           ***   * *    **  **                    *      *     *

i1_HQ_samplec9816d|c1892/f2p5/1103      CGCCCAGTCACGCGGCCTGCTCCCCTCCACCCAACCCCAGGGCTCTATGGAAGGACCAG  649
i0_HQ_samplec9816d|c3248/f3p6/738       CAGTCCAATACCATCCTGCGTCACCTGGGCCGCACCCTTGGGCTCTATGGAAGGACCAG  284
NM_000852.3                             CAGTCCAATACCATCCTGCGTCACCTGGGCCGCACCCTTGGGCTCTATGGAAGGACCAG  501
                                        *   *    **    *    ** ***   **  ****   ********************

i1_HQ_samplec9816d|c1892/f2p5/1103      CAGGAGGCAGCCCTGGTGGACATGGTGAATGACGGCGTGGAGGACCTCCGCTGCAAATAC  709
i0_HQ_samplec9816d|c3248/f3p6/738       CAGGAGGCAGCCCTGGTGGACATGGTGAATGACGGCGTGGAGGACCTCCGCTGCAAATAC  344
NM_000852.3                             CAGGAGGCAGCCCTGGTGGACATGGTGAATGACGGCGTGGAGGACCTCCGCTGCAAATAC  561
```

```
                                        ************************************************************

i1_HQ_samplec9816d|c1892/f2p5/1103      ATCTCCCTCATCTACACCAACTATGAGGCGGGCAAGGATGACTATGTGAAGGCACTGCCC  769
i0_HQ_samplec9816d|c3248/f3p6/738       GTCTCCCTCATCTACACCAACTATGAGGCGGGCAAGGATGACTATGTGAAGGCACTGCCC  404
NM_000852.3                             ATCTCCCTCATCTACACCAACTATGAGGCGGGCAAGGATGACTATGTGAAGGCACTGCCC  621
                                         ***********************************************************

i1_HQ_samplec9816d|c1892/f2p5/1103      GGGCAACTGAAGCCTTTTGAGACCCTGCTGTCCCAGAACCAGGGAGGCAAGACCTTCATT  829
i0_HQ_samplec9816d|c3248/f3p6/738       GGGCAACTGAAGCCTTTTGAGACCCTGCTGTCCCAGAACCAGGGAGGCAAGACCTTCATT  464
NM_000852.3                             GGGCAACTGAAGCCTTTTGAGACCCTGCTGTCCCAGAACCAGGGAGGCAAGACCTTCATT  681
                                        ************************************************************

i1_HQ_samplec9816d|c1892/f2p5/1103      GTGGGAGACCAGATCTCCTTCGCTGACTACAACCTGCTGGACTTGCTGCTGATCCATGAG  889
i0_HQ_samplec9816d|c3248/f3p6/738       GTGGGAGACCAGATCTCCTTCGCTGACTACAACCTGCTGGACTTGCTGCTGATCCATGAG  524
NM_000852.3                             GTGGGAGACCAGATCTCCTTCGCTGACTACAACCTGCTGGACTTGCTGCTGATCCATGAG  741
                                        ************************************************************

i1_HQ_samplec9816d|c1892/f2p5/1103      GTCCTAGCCCCTGGCTGCCTGGATGCGTTCCCCCTGCTCTCAGCATATGTGGGGCGCCTC  949
i0_HQ_samplec9816d|c3248/f3p6/738       GTCCTAGCCCCTGGCTGCCTGGATGCGTTCCCCCTGCTCTCAGCATATGTGGGGCGCCTC  584
NM_000852.3                             GTCCTAGCCCCTGGCTGCCTGGATGCGTTCCCCCTGCTCTCAGCATATGTGGGGCGCCTC  801
                                        ************************************************************

i1_HQ_samplec9816d|c1892/f2p5/1103      AGTGCCCGGCCCAAGCTCAAGGCCTTCCTGGCCTCCCCTGAGTACGTGAACCTCCCCATC  1009
i0_HQ_samplec9816d|c3248/f3p6/738       AGTGCCCGGCCCAAGCTCAAGGCCTTCCTGGCCTCCCCTGAGTACGTGAACCTCCCCATC  644
NM_000852.3                             AGTGCCCGGCCCAAGCTCAAGGCCTTCCTGGCCTCCCCTGAGTACGTGAACCTCCCCATC  861
                                        ************************************************************

i1_HQ_samplec9816d|c1892/f2p5/1103      AATGGCAACGGGAAACAGTGAGGGTTGGGGGGACTCTGAGCGGGAGGCAGAGTTTGCCTT  1069
i0_HQ_samplec9816d|c3248/f3p6/738       AATGGCAACGGGAAACAGTGAGGGTTGGGGGGACTCTGAGCGGGAGGCAGAGTTTGCCTT  704
NM_000852.3                             AATGGCAACGGGAAACAGTGAGGGTTGGGGGGACTCTGAGCGGGAGGCAGAGTTTGCCTT  921
                                        ************************************************************

i1_HQ_samplec9816d|c1892/f2p5/1103      Cctttctccaggaccaataaaatttctaagagag--------------------------  1103
i0_HQ_samplec9816d|c3248/f3p6/738       CCTTTCTCCAGGACCAATAAAATTTCTAAGAGAG--------------------------  738
NM_000852.3                             CCTTTCTCCAGGACCAATAAAATTTCTAAGAGAGCTAAAAAAAAAAAAAAAAAAAAAAAA  981
                                        **********************************

i1_HQ_samplec9816d|c1892/f2p5/1103      -----     1103
i0_HQ_samplec9816d|c3248/f3p6/738       -----     738
NM_000852.3                             AAAAA     986
```

```
NP_000843.1       MPPYTVVYFPVRGRCAALRMLLADQGQSWKEEVVTVETWQEGSLKASCLYGQLPKFQDGD    60
VIRT85086         ------------------------------------------------------------    0
VIRT85693         ------------------------------------------------------------    0

NP_000843.1       LTLYQSNTILRHLGRTLGLYGKDQQEAALVDMVNDGVEDLRCKYISLIYTNYEAGKDDYV    120
VIRT85086         ---------------------MDQQQVQQV------------------------------    9
VIRT85693         ---------------------MDQQQVQQV------------------------------    9
                                        ***:.  *

NP_000843.1       KALPGQLKPFETLLSQNQGGKTFIVGDQIS---------------------FADYNLLDL    159
VIRT85086         ------VVSEGDLVSHNEGLASLVLGQQGLKRLQLPGQCLHIVILARLIVGVDEGDVFAA    63
VIRT85693         ------VVSEGDLVSHNEGLASLVLGQQGLKRLQLPGQCLHIVILARLIVGVDEGDVFAA    63
                        :     *:*:*:*  ::::*:*                       . : :::

NP_000843.1       LLIH---EVLAPGCLDAFPLLSAYVGRLSARPKLKAFLAS----------PEYVNLPING    206
VIRT85086         EVLHAVIHHVHQGCLLLVLPIEPWGWVEGSRPR--DWAPDQHTVTGDGPTRGLSVSPSLG    121
VIRT85693         EVLHAVIHHVHQGCLLLVLPIEPKGAAQVTQD-------------GIGLVQGEVSVLEL-    109
                   ::*   . :  ***  .  :.       ::

NP_000843.1       NGKQ---------------------------------    210
VIRT85086         GGEEDVRGRMRDCHTAGAQSSPDTWEAAWHRARIAGTQA    160
VIRT85693         -GELPV-------------------------------    114
                   *:
```

**KRT14**

| Sequence | Header | AA Seq |
|---|---|---|
| **Present** | i1_HQ_samplec9816d\|c41519/f7p0/1143 | VIRT5673 |
| | i0_HQ_samplec9816d\|c8457/f2p5/954 | VIRT6606 |
| **Absent** | i1_HQ_samplec9816d\|c11199/f3p18/1241 | VIRT7678 |
| **NCBI** | NM_000526 | NP_000517.2 |
| | | |

>i1_HQ_samplec9816d|c41519/f7p0/1143
isoform=c41519;full_length_coverage=7;non_full_length_coverage=0;isoform_length=1143

AGACCAAAGGTCGCTACTGCATGCAGCTGGCCCAGATCCAGGAGATGATTGGCAGCGTGGAGGAGCAGCTGGCCCAG
CTCCGCTGCGAGATGGAGCAGCAGAACCAGGAGTACAAGATCCTGCTGGACGTGAAGACGCGGCTGGAGCAGGAGAT
CGCCACCTACCGCCGCCTGCTGGAGGGCGAGGACGCCCAGTGAGTCTTGGCCCTCCCCTTAGTCCGCGCCCCCCATGGC
ACTCTCACGGCCCCACCATGTATCTAATGATCCTGTCCTTTTCTATTTTCACAGCCTCTCCTCCTCCCAGTTCTCCTCTGGA
TCGCAGTCATCCAGAGATGGTAAGACCCTCCTCCTCTGCAGGCCTGGGCTCCAGGCCACCCTCTGTACCCCAAGCAGGT
CTAGGCATTGGCTAGGGGCTCCGTGAGGGGCTGAGCTCTAGTGCTGTCACCCAGTTTCCCTTGTGAACCTCCTTGGGTG
GAAGAAGCTATTTTCTAAACCCTCCTTAGGGCTAGGAGAGGCAGCCCCCACCTCTTGCTTCTACGTGGTGTCTGTGGCA
GATCCTATTGCTGTTGTGGTCAGCACCATGAACAGGGCCCTACAGGGCTCTTCCCACTGAGACCACTCCATTGGGTGAA
TATGGATGGAACCAGCCAGGTGTGAGCTCTTAGGAAGCTCTAATCTGAGGGCAAAGACTCTGTCTCTGACCTTTGGGA
GCCCTCGTCTGAAAGAAATGTGTTGATGGTATCAGTGCTTGGGCAACAGCAGGGAGTGAAGCAGTAATCAGGGGAGA
GGGCAATGGGGAGCCAGTTTGAGTTTCCTCACCTTCTTGGCCTCCTTACTCCTGATTAGTCCATTGTCTGTCCACCTCTGG
TAACGTCCTCTTCCCACCTCTTCCCCAGTGACCTCCTCCAGCCGCCAAATCCGCACCAAGGTCATGGATGTGCACGATGG
CAAGGTGGTGTCCACCCACGAGCAGGTCCTTCGCACCAAGAACTGAGGCTGCCCAGCCCCGCTCAGGCCTAGGAGGCC
CCCCGTGTGGACACAGATCCCACTGGAAGATCCCCTCTCCTGCCCAAGCACTTCACAGCTGGACCCTGCTTCACCCTCAC
CCCCTCCTGGCAAtcaatacagcttcattatctgagtt

>VIRT5673

MALSRPHHVSNDPVLFYFHSLSSSQFSSGSQSSRDGKTLLLCRPGLQATLCTPSRSRHWL
GAP

>i0_HQ_samplec9816d|c8457/f2p5/954
isoform=c8457;full_length_coverage=2;non_full_length_coverage=5;isoform_length=954

atgcccaaTGAGTCTTGGCCCTCCCCTTAGTCCGCCCCCCCATGGCACTCTCACGGCCCCACCATGTATCTAATGATCCTGC
CTTTTCTATTTTCACAGCCTCTCCTCCTCCCAGTTCTCCTCTGGATCGCAGTCATCCAGAGATGGTAAGACCCTCCTCCTCT
GCAGGCCTGGGCTCCAGGCCACCCTCTGTACCCCAAGCAGGTCTAGGCATTGGCTAGGGGCTCCGTGAGGGGCTGAGC
TCTAGTGCTGTCACCCAGTTTCCCTTGTGAACCTCCTTGGGTGGAAGAAGCTATTTTCTAAACCCTCCTTAGGGCTAGGA
GAGGCAGCCCCCACCTCTTGCTTCTACGTGGTGTCTGTGGCAGATCCTATTGCTGTTGTGGTCAGCACCATGAACAGGG
CCCTACAGGGCTCTTCCCACTGAGACCACTCCATTGGGTGAATATGGATGGAACCAGCCAGGTGTGAGCTCTTAGGAAG
CTCTAATCTGAGGGCAAAGACTCTGTCTCTGACCTTTGGGAGCCCTCGTCTGAAAGAAATGTGTTGATGGTATCAGTGC
TTGGGCAACAGCAGGGAGTGAAGCAGTAATCAGGGGAGAGGGCAATGGGGAGCCAGTTTGAGTTTCCTCACCTTCTT
GGCCTCCTTACTCCTGATTAGTCCATTGTCTGTCCACCTCTGGTAACGTCCTCTTCCCACCTCTTCCCCAGTGACCTCCTCC
AGCCGCCAAATCCGCACCAAGGTCATGGATGTGCACGATGGCAAGGTGGTGTCCACCCACGAGCAGGTCCTTCGCACC
AAGAACTGAGGCTGCCCAGCCCCGCTCAGGCCTAGGAGGCCCCCGTGTGGACACAGATCCCACTGGAAGATCCCCTCT
CCTGCCCAAGCACTTCACAGCTGGACCCTGCTTCACCCTCACCCCCTCCTggcaatcaatacagcttcattatctgagtt

>VIRT6606

MCTMARWCPPTSRSFAPRTEAAQPRSGLGGPRVDTDPTGRSPLLPKHFTAGPCFTLTPSW
QSIQLHYLS

>i1_HQ_samplec9816d|c11199/f3p18/1241
isoform=c11199;full_length_coverage=3;non_full_length_coverage=18;isoform_length=1241

CTTCTGGTGGGCAGTGAGAAGGTGACCATGCAGAACCTCAACGACCGCCTGGCCTCCTACCTGGACAAGGTGCGTGCT
CTGGAGGAGGCCAACGCCGACCTGGAAGTGAAGATCCGTGACTGGTACCAGAGGCAGCGGCCTGCTGAGATCAAAGA
CTACAGTCCCTACTTCAAGACCATTGAGGACCTGAGGAACAAGATTCTCACAGCCACAGTGGACAATGCCAATGTCCTT
CTGCAGATTGACAATGCCCGTCTGGCCGCGGATGACTTCCGCACCAAGTATGAGACAGAGTTGAACCTGCGCATGAGT
GTGGAAGCCGACATCAATGGCCTGCGCAGGGTGCTGGACGAACTGACCCTGGCCAGAGCTGACCTGGAGATGCAGAT
TGAGAGCCTGAAGGAGGAGCTGGCCTACCTGAAGAAGAACCACGAGGAGGAGATGAATGCCCTGAGAGGCCAGGTG
GGTGGAGATGTCAATGTGGAGATGGACGCTGCACCTGGCGTGGACCTGAGCCGCATTCTGAACGAGATGCGTGACCA
GTATGAGAAGATGGCAGAGAAGAACCGCAAGGATGCCGAGGAATGGTTCTTCACCAAGACAGAGGAGCTGAACCGCG
AGGTGGCCACCAACAGCGAGCTGGTGCAGAGCGGCAAGAGCGAGATCTCGGAGCTCCGGCGCACCATGCAGAACCTG
GAGATTGAGCTGCAGTCCCAGCTCAGCATGAAAGCATCCCTGGAGAACAGCCTGGAGGAGACCAAAGGTCGCTACTGC
ATGCAGCTGGCCCAGATCCAGGAGATGATTGGCAGCGTGGAGGAGCAGCTGGCCCAGCTCCGCTGCGAGATGGAGCA
GCAGAACCAGGAGTACAAGATCCTGCTGGACGTGAAGACGCGGCTGGAGCAGGAGATCGCCACCTACCGCCGCCTGC
TGGAGGGCGAGGACGCCCACCTCTCCTCCTCCCAGTTCTCCTCTGGATCGCAGTCATCCAGAGATGTGACCTCCTCCAGC
CGCCAAATCCGCACCAAGGTCATGGATGTGCACGATGGCAAGGTGGTGTCCACCCACGAGCAGGTCCTTCGCACCAAG
AACTGAGGCTGCCCAGCCCCGCTCAGGCCTAGGAGGCCCCCCGTGTGGACACAGATCCCACTGGAAGATCCCCTCTCCT
GCCCAAGCACTTCACAGCTGGACCCTGCTTCACCCTCACCCCCTCCTGGCAATCAATACagcttcattatctgagt

>VIRT7678
MQNLNDRLASYLDKVRALEEANADLEVKIRDWYQRQRPAEIKDYSPYFKTIEDLRNKILT
ATVDNANVLLQIDNARLAADDFRTKYETELNLRMSVEADINGLRRVLDELTLARADLEMQ
IESLKEELAYLKKNHEEEMNALRGQVGGDVNVEMDAAPGVDLSRILNEMRDQYEKMAEKN
RKDAEEWFFTKTEELNREVATNSELVQSGKSEISELRRTMQNLEIELQSQLSMKASLENS
LEETKGRYCMQLAQIQEMIGSVEEQLAQLRCEMEQQNQEYKILLDVKTRLEQEIATYRRL
LEGEDAHLSSSQFSSGSQSSRDVTSSSRQIRTKVMDVHDGKVVSTHEQVLRTKN

>NM_000526.4 Homo sapiens keratin 14 (KRT14), mRNA
ACCCGAGCACCTTCTCTTCACTCAGCCAACTGCTCGCTCGCTCACCTCCCTCCTCTGCACCATGACCACC
TGCAGCCGCCAGTTCACCTCCTCCAGCTCCATGAAGGGCTCCTGCGGCATCGGGGGCGGCATCGGGGGCG
GCTCCAGCCGCATCTCCTCCGTCCTGGCCGGAGGGTCCTGCCGCGCCCCCAGCACCTACGGGGGCGGCCT
GTCTGTCTCATCCTCCCGCTTCTCCTCTGGGGGAGCCTACGGGCTGGGGGGCGGCTATGGCGGTGGCTTC
AGCAGCAGCAGCAGCAGCTTTGGTAGTGGCTTTGGGGGAGGATATGGTGGTGGCCTTGGTGCTGGCTTGG
GTGGTGGCTTTGGTGGTGGCTTTGCTGGTGGTGATGGGCTTCTGGTGGGCAGTGAGAAGGTGACCATGCA
GAACCTCAATGACCGCCTGGCCTCCTACCTGGACAAGGTGCGTGCTCTGGAGGAGGCCAACGCCGACCTG
GAAGTGAAGATCCGTGACTGGTACCAGAGGCAGCGGCCTGCTGAGATCAAAGACTACAGTCCCTACTTCA
AGACCATTGAGGACCTGAGGAACAAGATTCTCACAGCCACAGTGGACAATGCCAATGTCCTTCTGCAGAT
TGACAATGCCCGTCTGGCCGCGGATGACTTCCGCACCAAGTATGAGACAGAGTTGAACCTGCGCATGAGT
GTGGAAGCCGACATCAATGGCCTGCGCAGGGTGCTGGACGAACTGACCCTGGCCAGAGCTGACCTGGAGA
TGCAGATTGAGAGCCTGAAGGAGGAGCTGGCCTACCTGAAGAAGAACCACGAGGAGGAGATGAATGCCCT
GAGAGGCCAGGTGGGTGGAGATGTCAATGTGGAGATGGACGCTGCACCTGGCGTGGACCTGAGCCGCATT
CTGAACGAGATGCGTGACCAGTATGAGAAGATGGCAGAGAAGAACCGCAAGGATGCCGAGGAATGGTTCT
TCACCAAGACAGAGGAGCTGAACCGCGAGGTGGCCACCAACAGCGAGCTGGTGCAGAGCGGCAAGAGCGA
GATCTCGGAGCTCCGGCGCACCATGCAGAACCTGGAGATTGAGCTGCAGTCCCAGCTCAGCATGAAAGCA
TCCCTGGAGAACAGCCTGGAGGAGACCAAAGGTCGCTACTGCATGCAGCTGGCCCAGATCCAGGAGATGA
TTGGCAGCGTGGAGGAGCAGCTGGCCCAGCTCCGCTGCGAGATGGAGCAGCAGAACCAGGAGTACAAGAT
CCTGCTGGACGTGAAGACGCGGCTGGAGCAGGAGATCGCCACCTACCGCCGCCTGCTGGAGGGCGAGGAC
GCCCACCTCTCCTCCTCCCAGTTCTCCTCTGGATCGCAGTCATCCAGAGATGTGACCTCCTCCAGCCGCC
AAATCCGCACCAAGGTCATGGATGTGCACGATGGCAAGGTGGTGTCCACCCACGAGCAGGTCCTTCGCAC
CAAGAACTGAGGCTGCCCAGCCCCGCTCAGGCCTAGGAGGCCCCCCGTGTGGACACAGATCCCACTGGAA
GATCCCCTCTCCTGCCCAAGCACTTCACAGCTGGACCCTGCTTCACCCTCACCCCCTCCTGGCAATCAAT
ACAGCTTCATTATCTGAGTTGCATAAAAAAAAAAAAAAAAAAA

>NP_000517.2 keratin, type I cytoskeletal 14 [Homo sapiens]
MTTCSRQFTSSSSMKGSCGIGGGIGGGSSRISSVLAGGSCRAPSTYGGGLSVSSSRFSSGGAYGLGGGYG
GGFSSSSSSFGSGFGGGYGGGLGAGLGGGFGGGFAGGDGLLVGSEKVTMQNLNDRLASYLDKVRALEEAN
ADLEVKIRDWYQRQRPAEIKDYSPYFKTIEDLRNKILTATVDNANVLLQIDNARLAADDFRTKYETELNL
RMSVEADINGLRRVLDELTLARADLEMQIESLKEELAYLKKNHEEEMNALRGQVGGDVNVEMDAAPGVDL

```
SRILNEMRDQYEKMAEKNRKDAEEWFFTKTEELNREVATNSELVQSGKSEISELRRTMQNLEIELQSQLS
MKASLENSLEETKGRYCMQLAQIQEMIGSVEEQLAQLRCEMEQQNQEYKILLDVKTRLEQEIATYRRLLE
GEDAHLSSSQFSSGSQSSRDVTSSSRQIRTKVMDVHDGKVVSTHEQVLRTKN


i1_HQ_samplec9816d|c11199/f3p18/1241     ------------------------------------------------------------     0
NM_000526.4                              ACCCGAGCACCTTCTCTTCACTCAGCCAACTGCTCGCTCGCTCACCTCCCTCCTCTGCAC     60
i1_HQ_samplec9816d|c41519/f7p0/1143      ------------------------------------------------------------     0
i0_HQ_samplec9816d|c8457/f2p5/954        ------------------------------------------------------------     0

i1_HQ_samplec9816d|c11199/f3p18/1241     ------------------------------------------------------------     0
NM_000526.4                              CATGACCACCTGCAGCCGCCAGTTCACCTCCTCCAGCTCCATGAAGGGCTCCTGCGGCAT     12
i1_HQ_samplec9816d|c41519/f7p0/1143      ------------------------------------------------------------     0
i0_HQ_samplec9816d|c8457/f2p5/954        ------------------------------------------------------------     0

i1_HQ_samplec9816d|c11199/f3p18/1241     ------------------------------------------------------------     0
NM_000526.4                              CGGGGGCGGCATCGGGGGCGGCTCCAGCCGCATCTCCTCCGTCCTGGCCGGAGGGTCCTG     18
i1_HQ_samplec9816d|c41519/f7p0/1143      ------------------------------------------------------------     0
i0_HQ_samplec9816d|c8457/f2p5/954        ------------------------------------------------------------     0

i1_HQ_samplec9816d|c11199/f3p18/1241     ------------------------------------------------------------     0
NM_000526.4                              CCGCGCCCCCAGCACCTACGGGGGCGGCCTGTCTGTCTCATCCTCCCGCTTCTCCTCTGG     24
i1_HQ_samplec9816d|c41519/f7p0/1143      ------------------------------------------------------------     0
i0_HQ_samplec9816d|c8457/f2p5/954        ------------------------------------------------------------     0

i1_HQ_samplec9816d|c11199/f3p18/1241     ------------------------------------------------------------     0
NM_000526.4                              GGGAGCCTACGGGCTGGGGGGCGGCTATGGCGGTGGCTTCAGCAGCAGCAGCAGCAGCTT     30
i1_HQ_samplec9816d|c41519/f7p0/1143      ------------------------------------------------------------     0
i0_HQ_samplec9816d|c8457/f2p5/954        ------------------------------------------------------------     0

i1_HQ_samplec9816d|c11199/f3p18/1241     ------------------------------------------------------------     0
NM_000526.4                              TGGTAGTGGCTTTGGGGGAGGATATGGTGGTGGCCTTGGTGCTGGCTTGGGTGGTGGCTT     36
i1_HQ_samplec9816d|c41519/f7p0/1143      ------------------------------------------------------------     0
i0_HQ_samplec9816d|c8457/f2p5/954        ------------------------------------------------------------     0

i1_HQ_samplec9816d|c11199/f3p18/1241     ----------------------------CTTCTGGTGGGCAGTGAGAAGGTGACCATGCA     32
NM_000526.4                              TGGTGGTGGCTTTGCTGGTGGTGATGGGCTTCTGGTGGGCAGTGAGAAGGTGACCATGCA     42
i1_HQ_samplec9816d|c41519/f7p0/1143      --------AGACCAAAGGTCGCTACTGCATGCAGCT------------GGCCCAGATCCA     40
i0_HQ_samplec9816d|c8457/f2p5/954        ------------------------------------------------------------     0

i1_HQ_samplec9816d|c11199/f3p18/1241     GAACCTCAACGACCGCCTGGCCTCCTACCTGGACAAGGTGCGTGCTCTGGAGGAGGCCAA     92
NM_000526.4                              GAACCTCAATGACCGCCTGGCCTCCTACCTGGACAAGGTGCGTGCTCTGGAGGAGGCCAA     48
i1_HQ_samplec9816d|c41519/f7p0/1143      GGAGATGATTGGCAGCGTGGAGGAGCAGCTGGCCCAGCTCCGCTGCGAGATGGAGCAGCA     10
i0_HQ_samplec9816d|c8457/f2p5/954        ------------------------------------------------------------     0

i1_HQ_samplec9816d|c11199/f3p18/1241     CGCCGACCTGGAAGTGAAGATCCGTGACTGGTACCAGAGGCAGCGGCCTGCTGAGATCAA     15
NM_000526.4                              CGCCGACCTGGAAGTGAAGATCCGTGACTGGTACCAGAGGCAGCGGCCTGCTGAGATCAA     54
i1_HQ_samplec9816d|c41519/f7p0/1143      ---GAACCAGGAGTACAAGATCCTGCTGGACGTGAAGACGCGGCTGGAGCAGGAGATCGC     15
i0_HQ_samplec9816d|c8457/f2p5/954        ------------------------------------------------------------     0

i1_HQ_samplec9816d|c11199/f3p18/1241     AGACTACAGTCCCTACTTCAAGACCATTGAGGACCTGAGGAACAAGATTCTCACAGCCAC     21
NM_000526.4                              AGACTACAGTCCCTACTTCAAGACCATTGAGGACCTGAGGAACAAGATTCTCACAGCCAC     60
i1_HQ_samplec9816d|c41519/f7p0/1143      CACCTACCGCCGCCTGCTGGAGGGCGAGGACGCCC-------------------------     19
i0_HQ_samplec9816d|c8457/f2p5/954        -----------------------------atgccc-------------------------     6
                                                                      * * **

i1_HQ_samplec9816d|c11199/f3p18/1241     AGTGGACAATGCCAATGTCCTTCTG-CAGATTGACAATGCCCGTCTGGCCGCGGATGACT     27
NM_000526.4                              AGTGGACAATGCCAATGTCCTTCTG-CAGATTGACAATGCCCGTCTGGCCGCGGATGACT     65
i1_HQ_samplec9816d|c41519/f7p0/1143      AGTGAGTCTTGGCCCTCCCCTTAGTCCGCGCCCCCCATGGCAC----------TCTCACG     24
i0_HQ_samplec9816d|c8457/f2p5/954        aaTGAGTCTTGGCCCTCCCCTTAGTCCG-CCCCCCCATGGCAC----------TCTCACG     55
                                         * **     ** *  *  ****    *       * *** *              * **

i1_HQ_samplec9816d|c11199/f3p18/1241     TCCGCACCAAGTATGAGACAGAGTTGAACCTGCGCATGAGTGTGGAAGCCGACATCAATG     33
NM_000526.4                              TCCGCACCAAGTATGAGACAGAGTTGAACCTGCGCATGAGTGTGGAAGCCGACATCAATG     71
i1_HQ_samplec9816d|c41519/f7p0/1143      GCCCCACCATGTATCTAATG------ATCCTGTCCTTTTCTATTTTCACAGCC-----TC     29
i0_HQ_samplec9816d|c8457/f2p5/954        GCCCCACCATGTATCTAATG------ATCCTGC-CTTTTCTATTTTCACAGCC-----TC     10
                                          ** ***** ****   *        * ****  * *   * *     * * *     *

i1_HQ_samplec9816d|c11199/f3p18/1241     GCCTGCGCAGGGTGCTGGACGAACTGACCCTGGCCAGAGCTGACCTGGAGATGCAGATTG     39
NM_000526.4                              GCCTGCGCAGGGTGCTGGACGAACTGACCCTGGCCAGAGCTGACCTGGAGATGCAGATTG     77
i1_HQ_samplec9816d|c41519/f7p0/1143      TCCTCCTCCCAGTTCTCCT----------CTGGATCGCAGTCATCCAGAGATGGTAAGAC     34
```

```
i0_HQ_samplec9816d|c8457/f2p5/954          TCCTCCTCCCAGTTCTCCT----------CTGGATCGCAGTCATCCAGAGATGGTAAGAC       15
                                            *** * *   ** **             ****   *   * * *  ******   *

i1_HQ_samplec9816d|c11199/f3p18/1241       AGAGCCTGAAGGAGGAGCTGGCCTAC----CTGAAGAAGAACCACGAGGAGGAGAT-GAA       44
NM_000526.4                                AGAGCCTGAAGGAGGAGCTGGCCTAC----CTGAAGAAGAACCACGAGGAGGAGAT-GAA       83
i1_HQ_samplec9816d|c41519/f7p0/1143        CCTCCTCCTCTGCAGGCCTGGGCTCCAGGCCACCCTCTGTACCCCAAGCAGGTCTAGGCA       40
i0_HQ_samplec9816d|c8457/f2p5/954          CCTCCTCCTCTGCAGGCCTGGGCTCCAGGCCACCCTCTGTACCCCAAGCAGGTCTAGGCA       21
                                               *      *  *  **** ** *    *       * *** * ** ***     * *

i1_HQ_samplec9816d|c11199/f3p18/1241       TGCCCTGAGAGGCCAGGTGGGTGGAGATGTCAATGTGGAGATGGACGCTGCACCTGGCGT       50
NM_000526.4                                TGCCCTGAGAGGCCAGGTGGGTGGAGATGTCAATGTGGAGATGGACGCTGCACCTGGCGT       89
i1_HQ_samplec9816d|c41519/f7p0/1143        TTGGCTA-GGGGCTCCGTGAGGGGCTGAGCTCTAGTGCTGTCACC--CAGTTTCCCTTGT       45
i0_HQ_samplec9816d|c8457/f2p5/954          TTGGCTA-GGGGCTCCGTGAGGGGCTGAGCTCTAGTGCTGTCACC--CAGTTTCCCTTGT       27
                                           *   **  * ***   *** * **    *     ***  *       * *   *    **

i1_HQ_samplec9816d|c11199/f3p18/1241       GGACCTGAGCCGCATTCTGAACGAGATGCGTGACCAGTATGAGAAGATGGCAGAGAAGAA       56
NM_000526.4                                GGACCTGAGCCGCATTCTGAACGAGATGCGTGACCAGTATGAGAAGATGGCAGAGAAGAA       95
i1_HQ_samplec9816d|c41519/f7p0/1143        GAACCTCCTTGGGTGGAAGAAGCTATTTTCTAAACCCTCCTTAGGGCTAGGAGAGGCAGC       51
i0_HQ_samplec9816d|c8457/f2p5/954          GAACCTCCTTGGGTGGAAGAAGCTATTTTCTAAACCCTCCTTAGGGCTAGGAGAGGCAGC       33
                                           * ****     *      ***     *   * * *  *       * * * ****

i1_HQ_samplec9816d|c11199/f3p18/1241       CCGCAA-GGATGCCGAGGAATGGTTCTTCACCAAGACAGAGGAGCTGAACCGCGAGGTGG       62
NM_000526.4                                CCGCAA-GGATGCCGAGGAATGGTTCTTCACCAAGACAGAGGAGCTGAACCGCGAGGTGG       10
i1_HQ_samplec9816d|c41519/f7p0/1143        CCCCACCTCTTGCTTCTACGTGGTGTCT----GTGGCAGATCCTATTGCTGTTGTGGTCA       57
i0_HQ_samplec9816d|c8457/f2p5/954          CCCCACCTCTTGCTTCTACGTGGTGTCT----GTGGCAGATCCTATTGCTGTTGTGGTCA       38
                                           ** **     ***       ****   *      * ****     *       * ***

i1_HQ_samplec9816d|c11199/f3p18/1241       CCACCAACAGCGAGCTGGTGCAGAGCGGCAAGAGCGAGATCTCGGAGCTCCGGCGCACCA       68
NM_000526.4                                CCACCAACAGCGAGCTGGTGCAGAGCGGCAAGAGCGAGATCTCGGAGCTCCGGCGCACCA       10
i1_HQ_samplec9816d|c41519/f7p0/1143        GCACCATGAACAGGGCCCTACA-------------GGGCTCTTCCCACTGA---GACCAC       61
i0_HQ_samplec9816d|c8457/f2p5/954          GCACCATGAACAGGGCCCTACA-------------GGGCTCTTCCCACTGA---GACCAC       43
                                            *****  * *  *    * **             * * ***      **    *  *

i1_HQ_samplec9816d|c11199/f3p18/1241       TGCAGAACCTGGAGATTGAGCTGCAGTCCCAGCTCAGCATGAAAGCATCCCTGGAGAACA       74
NM_000526.4                                TGCAGAACCTGGAGATTGAGCTGCAGTCCCAGCTCAGCATGAAAGCATCCCTGGAGAACA       11
i1_HQ_samplec9816d|c41519/f7p0/1143        TCCATTGGGTGAATATGGATGG----AACCAGCCAGGTGTGAGC-----TCTTAGGAAGC       66
i0_HQ_samplec9816d|c8457/f2p5/954          TCCATTGGGTGAATATGGATGG----AACCAGCCAGGTGTGAGC-----TCTTAGGAAGC       48
                                           * **     ** * ** **         *****   *  ***        **   ***

i1_HQ_samplec9816d|c11199/f3p18/1241       GCCTGGAGGAGACCAAAG---GTCGCTACTGCATGCAGCTGGC-CCAGATCCAGGAGATG       80
NM_000526.4                                GCCTGGAGGAGACCAAAG---GTCGCTACTGCATGCAGCTGGC-CCAGATCCAGGAGATG       11
i1_HQ_samplec9816d|c41519/f7p0/1143        TCTAATCTGAGGGCAAAGACTCTGTCTCTGACCTTTGGGAGCCCTCGTCTGAAAGAAATG       72
i0_HQ_samplec9816d|c8457/f2p5/954          TCTAATCTGAGGGCAAAGACTCTGTCTCTGACCTTTGGGAGCCCTCGTCTGAAAGAAATG       54
                                            *      ***  *****    * **     * *   *  * *  *   *  * ** ***

i1_HQ_samplec9816d|c11199/f3p18/1241       ATTGGCAGCGTGGAGGAGCAGCTGGCCCAGCTCCGCTGCGAGATGGAGCAGCAGAACCAG       86
NM_000526.4                                ATTGGCAGCGTGGAGGAGCAGCTGGCCCAGCTCCGCTGCGAGATGGAGCAGCAGAACCAG       12
i1_HQ_samplec9816d|c41519/f7p0/1143        TGT----T---------------------GATGGTATCAGTGCTTGGGCAACAGCAGGGA       76
i0_HQ_samplec9816d|c8457/f2p5/954          TGT----T---------------------GATGGTATCAGTGCTTGGGCAACAGCAGGGA       57
                                             *                          * *    *  * * * * *** *** *

i1_HQ_samplec9816d|c11199/f3p18/1241       GAGTACAAGATCCTGCTGGACGTGAAGACGCGGCTGGAGCAGGAGATCGCCACCTACCGC       92
NM_000526.4                                GAGTACAAGATCCTGCTGGACGTGAAGACGCGGCTGGAGCAGGAGATCGCCACCTACCGC       13
i1_HQ_samplec9816d|c41519/f7p0/1143        GTGAAGCAGTAATCAGGGGAGAGGGCAATGGGGAGCCAGTTTGAGTTTCCTCACCTTCTT       82
i0_HQ_samplec9816d|c8457/f2p5/954          GTGAAGCAGTAATCAGGGGAGAGGGCAATGGGGAGCCAGTTTGAGTTTCCTCACCTTCTT       63
                                           * * *  **        ***   *   * * **    **   *** *  *   *   *

i1_HQ_samplec9816d|c11199/f3p18/1241       CGCCTGCTGGAGGGCGAGGACGCCCACCTCTCCTCCTCCCAGTTCTCCTCTGGATCGCAG       98
NM_000526.4                                CGCCTGCTGGAGGGCGAGGACGCCCACCTCTCCTCCTCCCAGTTCTCCTCTGGATCGCAG       13
i1_HQ_samplec9816d|c41519/f7p0/1143        GGCCTCCTTACTCCTGATTAGTCCATT--GTCTGTCCACCTCTGGTAACGTCCTCTTCCC       88
i0_HQ_samplec9816d|c8457/f2p5/954          GGCCTCCTTACTCCTGATTAGTCCATT--GTCTGTCCACCTCTGGTAACGTCCTCTTCCC       69
                                            **** **       **  *  **      **   *  **  *  *    *      *

i1_HQ_samplec9816d|c11199/f3p18/1241       TCATCCAGAGATGTGACCTCCTCCAGCCGCCAAATCCGCACCAAGGTCATGGATGTGCAC       10
NM_000526.4                                TCATCCAGAGATGTGACCTCCTCCAGCCGCCAAATCCGCACCAAGGTCATGGATGTGCAC       14
i1_HQ_samplec9816d|c41519/f7p0/1143        ACCTCTTCCCCAGTGACCTCCTCCAGCCGCCAAATCCGCACCAAGGTCATGGATGTGCAC       94
i0_HQ_samplec9816d|c8457/f2p5/954          ACCTCTTCCCCAGTGACCTCCTCCAGCCGCCAAATCCGCACCAAGGTCATGGATGTGCAC       75
                                            * **       ************************************************

i1_HQ_samplec9816d|c11199/f3p18/1241       GATGGCAAGGTGGTGTCCACCCACGAGCAGGTCCTTCGCACCAAGAACTGAGGCTGCCCA       11
NM_000526.4                                GATGGCAAGGTGGTGTCCACCCACGAGCAGGTCCTTCGCACCAAGAACTGAGGCTGCCCA       14
i1_HQ_samplec9816d|c41519/f7p0/1143        GATGGCAAGGTGGTGTCCACCCACGAGCAGGTCCTTCGCACCAAGAACTGAGGCTGCCCA       10
i0_HQ_samplec9816d|c8457/f2p5/954          GATGGCAAGGTGGTGTCCACCCACGAGCAGGTCCTTCGCACCAAGAACTGAGGCTGCCCA       81
                                           ************************************************************

i1_HQ_samplec9816d|c11199/f3p18/1241       GCCCCGCTCAGGCCTAGGAGGCCCCCCGTGTGGACACAGATCCCACTGGAAGATCCCCTC       11
NM_000526.4                                GCCCCGCTCAGGCCTAGGAGGCCCCCCGTGTGGACACAGATCCCACTGGAAGATCCCCTC       15
i1_HQ_samplec9816d|c41519/f7p0/1143        GCCCCGCTCAGGCCTAGGAGGCCCCCCGTGTGGACACAGATCCCACTGGAAGATCCCCTC       10
i0_HQ_samplec9816d|c8457/f2p5/954          GCCCCGCTCAGGCCTAGGAGGC-CCCCGTGTGGACACAGATCCCACTGGAAGATCCCCTC       87
                                           ********************** *************************************

i1_HQ_samplec9816d|c11199/f3p18/1241       TCCTGCCCAAGCACTTCACAGCTGGACCCTGCTTCACCCTCACCCCCTCCTGGCAATCAA       12
```

```
NM_000526.4                               TCCTGCCCAAGCACTTCACAGCTGGACCCTGCTTCACCCTCACCCCCTCCTGGCAATCAA            16
i1_HQ_samplec9816d|c41519/f7p0/1143       TCCTGCCCAAGCACTTCACAGCTGGACCCTGCTTCACCCTCACCCCCTCCTGGCAAtcaa            11
i0_HQ_samplec9816d|c8457/f2p5/954         TCCTGCCCAAGCACTTCACAGCTGGACCCTGCTTCACCCTCACCCCCTCCTGgcaatcaa            93
                                          ************************************************************

i1_HQ_samplec9816d|c11199/f3p18/1241      TACagcttcattatctgagt------------------------     1241
NM_000526.4                               TACAGCTTCATTATCTGAGTTGCATAAAAAAAAAAAAAAAAAAA     1653
i1_HQ_samplec9816d|c41519/f7p0/1143       tacagcttcattatctgagtt-----------------------     1143
i0_HQ_samplec9816d|c8457/f2p5/954         tacagcttcattatctgagtt-----------------------     954
                                          ********************
```

```
VIRT6606         ------------------------------------------------------------     0
VIRT5673         ------------------------------------------------------------     0
VIRT7678         ------------------------------------------------------------     0
NP_000517.2      MTTCSRQFTSSSSMKGSCGIGGGIGGGSSRISSVLAGGSCRAPSTYGGGLSVSSSRFSSG     60

VIRT6606         ------------------------------------------------------------     0
VIRT5673         ------------------------------------------------------------     0
VIRT7678         ----------------------------------------------------------MQ     2
NP_000517.2      GAYGLGGGYGGGFSSSSSFGSGFGGGYGGGLGAGLGGGFGGGFAGGDGLLVGSEKVTMQ     120

VIRT6606         ------------------------------------------------------------     0
VIRT5673         ------------------------------------------------------------     0
VIRT7678         NLNDRLASYLDKVRALEEANADLEVKIRDWYQRQRPAEIKDYSPYFKTIEDLRNKILTAT     62
NP_000517.2      NLNDRLASYLDKVRALEEANADLEVKIRDWYQRQRPAEIKDYSPYFKTIEDLRNKILTAT     180

VIRT6606         ----------------------------------------------MCTMA---------     5
VIRT5673         -----------MALSRPHHVSNDPVLFYFHSLSSSQFSSGSQS-----------------     32
VIRT7678         VDNANVLLQIDNARLAADDFRTKYETELNLRMSVEADINGLRRVLDELTLARADLEMQIE     122
NP_000517.2      VDNANVLLQIDNARLAADDFRTKYETELNLRMSVEADINGLRRVLDELTLARADLEMQIE     240

VIRT6606         ------------------------------------------------------------     5
VIRT5673         ------------------------------------------------------------     32
VIRT7678         SLKEELAYLKKNHEEEMNALRGQVGGDVNVEMDAAPGVDLSRILNEMRDQYEKMAEKNRK     182
NP_000517.2      SLKEELAYLKKNHEEEMNALRGQVGGDVNVEMDAAPGVDLSRILNEMRDQYEKMAEKNRK     300

VIRT6606         --RWCPPTSRSFAPRTEAAQPRSGLGGPRVDT----------------------------     35
VIRT5673         --SRDGKTLLLCRPGLQATLCTPSRSRH--------------------------------     58
VIRT7678         DAEEWFFTKTEELNREVATNSELVQSGKSEISELRRTMQNLEIELQSQLSMKASLENSLE     242
NP_000517.2      DAEEWFFTKTEELNREVATNSELVQSGKSEISELRRTMQNLEIELQSQLSMKASLENSLE     360
                        *         *

VIRT6606         ------------------------------------------------------------     35
VIRT5673         ------------------------------------------------------------     58
VIRT7678         ETKGRYCMQLAQIQEMIGSVEEQLAQLRCEMEQQNQEYKILLDVKTRLEQEIATYRRLLE     302
NP_000517.2      ETKGRYCMQLAQIQEMIGSVEEQLAQLRCEMEQQNQEYKILLDVKTRLEQEIATYRRLLE     420

VIRT6606         --------DPTGRSPLLPKHFTAGPCFTLTPSWQSIQLHYLS----------   69
VIRT5673         WLGAP-----------------------------------------------   63
VIRT7678         GEDAHLSSSQFSSGSQSSRDVTSSSRQIRTKVMDVHDGKVVSTHEQVLRTKN   354
NP_000517.2      GEDAHLSSSQFSSGSQSSRDVTSSSRQIRTKVMDVHDGKVVSTHEQVLRTKN   472
```

## KRT16

| Sequence | Header | AA Seq |
|---|---|---|
| Present | i1_HQ_samplec9816d\|c55807/f2p0/1013 | |
| | i1_HQ_samplec9816d\|c63528/f5p0/1150 | |
| | i1_HQ_samplec9816d\|c42043/f3p0/1040 | |
| Absent | i1_HQ_samplec9816d\|c1544/f10p0/1305 | |
| NCBI | NM_005557 | |
| | | |

>i1_HQ_samplec9816d|c55807/f2p0/1013
isoform=c55807;full_length_coverage=2;non_full_length_coverage=0;isoform_length=1013

atgcgtgaccagtacgagcagatggcagagaaaaaccgcagagacgctgagacctggttCCTGAGCAAGACCGAGGAGCTGAACAAAGAA
GTGGCCTCCAACAGCGAACTGGTACAGAGCAGCCGCAGTGAGGTGACGGAGCTCCGGAGGGTGCTCCAGGGCCTGGA
GATTGAGCTGCAGTCCCAGCTCAGCATGGTATGAAGGACCCAGCACAGCAGCAGCCCCCAAGTCACCAGTAATGGCCA
CCACCCCCTCAAAAAGCCACAGTCTAGTTCCACCTTTCTTTTCTCAGGATGGGACCAGGGGACTCATGGGACCGTTATAT
AGATAGAGAAACTAAGCCCTAGAATAGTGGGCTAGCTTTTCTCCATATTGTCTGGCCCATCAGTACCCCAACTGGGATC
AAAATCCAGGCATCTCTCAAAAAACATGCCCAGAGACCTGGAGGAACAGGAGTGACCACCTCCATGGACTCTTTTTCTC
TCTCTCACTTGCAGAAAGCATCCCTGGAGAACAGCCTGGAGGAGACCAAAGGCCGCTACTGCATGCAGCTGTCCCAGA
TCCAGGGACTGATTGGCAGTGTGGAGGAGCAGCTGGCCCAGCTACGCTGTGAGATGGAGCAGCAGAGCCAGGAGTAC
CAGATCTTGCTGGATGTGAAGACGCGGCTGGAGCAGGAGATTGCCACCTACCGCCGCCTGCTGGAGGGCGAGGATGC
CCACCTTTCCTCCCAGCAAGCATCTGGCCAATCCTATTCTTCCCGCGAGGTCTTCACCTCCTCCTCGTCCTCTTCGAGCCGT
CAGACCCGGCCCATCCTCAAGGAGCAGAGCTCATCCAGCTTCAGCCAGGGCCAGAGCTCCTAGAACTGAGCTGCCTCTA
CCACAGCCTCCTGCCCACCAGCTGGCCTCACCTCCTGAAGGCCCGGGTCAGGACCCTGCTCTCCTGGCGCAGTTCCCAG
CTATCTCCCCTGCTCCTCTGCTGgtggtgggctaataaagctgactttctggttgatg

>VIRT34198

MDSFSLSHLQKASLENSLEETKGRYCMQLSQIQGLIGSVEEQLAQLRCEMEQQSQEYQIL
LDVKTRLEQEIATYRRLLEGEDAHLSSQQASGQSYSSREVFTSSSSSSSRQTRPILKEQS
SSSFSQGQSS

>i1_HQ_samplec9816d|c63528/f5p0/1150
isoform=c63528;full_length_coverage=5;non_full_length_coverage=0;isoform_length=1150

aacgtGGAGATGGATGCTGCACCTGGCGTGGACCTGAGCCGCATCCTGAATGAGATGCGCACCAGTACGAGCAGATGG
CAGAGAAAAACCGCAGAGACGCTGAGACCTGGTTCCTGAGCAAGGTGGGGCTCGGGCCCGCAGTGGCCTGCAGCACT
TCCCAGCTGGGGGCTTTGGGAGAGCCTCACCTTTCACTCTGCTTTCCTGCCTCAGACCGAGGAGCTGAACAAAGAAGTG
GCCTCCAACAGCGAACTGGTACAGAGCAGCCGCAGTGAGGTGACGGAGCTCCGGAGGGTGCTCCAGGGCCTGGAGAT
TGAGCTGCAGTCCCAGCTCAGCATGGTATGAAGGACCCAGCACAGCAGCAGCCCCCAAGTCACCAGTAATGGCCACCA
CCCCCTCAAAAAGCCACAGTCTAGTTCCACCTTTCTTTTCTCAGGATGGGACCAGGGGACTCATGGGACCGTTATATAGA
TAGAGAAACTAAGCCCTAGAATAGTGGGCTAGCTTTTCTCCATATTGTCTGGCCCATCAGTACCCCAACTGGGATCAAA
ATCCAGGCATCTCTCAAAAAACATGCCCAGAGACCTGGAGGAACAGGAGTGACCACCTCCATGGACTCTTTTTCTCTCTC
TCACTTGCAGAAAGCATCCCTGGAGAACAGCCTGGAGGAGACCAAAGGCCGCTACTGCATGCAGCTGTCCCAGATCCA
GGGACTGATTGGCAGTGTGGAGGAGCAGCTGGCCCAGCTACGCTGTGAGATGGAGCAGCAGAGCCAGGAGTACCAG
ATCTTGCTGGATGTGAAGACGCGGCTGGAGCAGGAGATTGCCACCTACCGCCGCCTGCTGGAGGGCGAGGATGCCCA
CCTTTCCTCCCAGCAAGCATCTGGCCAATCCTATTCTTCCCGCGAGGTCTTCACCTCCTCCTCGTCCTCTTCGAGCCGTCA
GACCCGGCCCATCCTCAAGGAGCAGAGCTCATCCAGCTTCAGCCAGGGCCAGAGCTCCTAGAACTGAGCTGCCTCTACC
ACAGCCTCCTGCCCACCAGCTGGCCTCACCTCCTGAAGGCCGGGTCAGGACCCTGCTCTCCAGCGCAGTTCCCAGCTGT
CTCCCTGCTCCTCTGCTGGTGGTGGGctaataaagctgactttctggttgat

>VIRT38686

MDSFSLSHLQKASLENSLEETKGRYCMQLSQIQGLIGSVEEQLAQLRCEMEQQSQEYQIL
LDVKTRLEQEIATYRRLLEGEDAHLSSQQASGQSYSSREVFTSSSSSSSRQTRPILKEQS
SSSFSQGQSS

>i1_HQ_samplec9816d|c42043/f3p0/1040
isoform=c42043;full_length_coverage=3;non_full_length_coverage=0;isoform_length=1040

gtgacggagctccggagggtgctccagggcctggagattgagctgcagtcccagctcagcatGGTATGAAGGACCCAGCACAGCAGCAGCCC
CCAAGTCACCAGTAATGGCCACCACCCCCTCAAAAAGCCACAGTCTAGTTCCACCTTTCTTTTCTCAGGATGGGACCAGG
GGACTCATGGGACCGTTATATAGATAGAGAAACTAAGCCCTAGAATAGTGGGCTAGCTTTTCTCCATATTGTCTGGCCC
ATCAGTACCCCAACTGGGATCAAAATCCAGGCATCTCTCAAAAAACATGCCCAGAGACCTGGAGGAACAGGAGTGACC
ACCTCCATGGACTCTTTTTCTCTCTCTCACTTGCAGAAAGCATCCCTGGAGAACAGCCTGGAGGAGACCAAAGGCCGCT
ACTGCATGCAGCTGTCCCAGATCCAGGGACTGATTGGCAGTGTGGAGGAGCAGCTGGCCCAGCTACGCTGTGAGATG
GAGCAGCAGAGCCAGGAGTACCAGATCTTGCTGGATGTGAAGACGCGGCTGGAGCAGGAGATTGCCACCTACCGCCG
CCTGCTGGAGGGCGAGGATGCCCACCTTTCCTCCCAGCAAGCATCTGGCCAATCCTATTCTTCCCGCGAGGGTAAGGCT
TCTGAGGCTCCCCGGCACTGCAGCCCCTCTGCCTGTTTCCATGGAGTGGGGGCTGGGCCCTTCTCCTCAGAGCTCCCAG
CCCTCCCTTCTCCCTGCCCTGGAGTCAGCTTAGCTCTCAGACCCCTTCTCACCTCCTCTTCTCTCTCCCACAGTCTTCACCTC
CTCCTCGTCCTCTTCGAGCCGTCAGACCCGGCCCATCCTCAAGGAGCAGAGCTCATCCAGCTTCAGCCAGGGCCAGAGC
TCCTAGAACTGAGCTGCCTCTACCACAGCCTCCTGCCCACCAGCTGGCCTCACCTCCTGAAGGCCCGGGTCAGGACCCT
GCTCTCCTGGCGCAGTTCCCAGCTATCTCCCCTGCTCCTCTGCTggtggtgggctaataaagctgactttctggttgat

>VIRT39978
MDSFSLSHLQKASLENSLEETKGRYCMQLSQIQGLIGSVEEQLAQLRCEMEQQSQEYQIL
LDVKTRLEQEIATYRRLLEGEDAHLSSQQASGQSYSSREGKASEAPRHCSPSACFHGVGA
GPFSSELPALPSPCPGVSLALRPLLTSSSLSHSLHLLLVLFEPSDPAHPQGAELIQLQPG
PELLELSCLYHSLLPTSWPHLLKARVRTLLSWRSSQLSPLLLCWWWANKADFLVD

>i1_HQ_samplec9816d|c1544/f10p0/1305
isoform=c1544;full_length_coverage=10;non_full_length_coverage=0;isoform_length=1305

acacacagcagagttgatcctgggctgaataatccagagtgaggagttggacgggaccgggaGTGATGAAATCCAGAGGGGAACCTGGAGT
CAGCAGTTAGGAGGGCCCCGCCTTCCCCAGCTGCATATAAAGGTGCGTGCTCTGGAGGAGGCCAACGCCGACCTGGAA
GTGAAGATCCGTGACTGGTACCAGAGGCAGCGGCCCAGTGAGATCAAAGACTACAGTCCCTACTTCAAGACCATCGAG
GACCTGAGGAACAAGATCATTGCGGCCACCATTGAGAATGCGCAGCCCATTTTGCAGATTGACAATGCCAGGCTGGCA
GCCGATGACTTCAGGACCAAGTATGAGCACGAACTGGCCCTGCGGCAGACTGTGGAGGCCGACGTCAATGGCCTGCGC
CGGGTGTTGGATGAGCTGACCCTGGCCAGGACTGACCTGGAGATGCAGATCGAAGGCCTGAAGGAGGAGCTGGCCTA
CCTGAGGAAGAACCACGAGGAGGAGATGCTTGCTCTGAGAGGTCAGACCGGCGGAGATGTGAACGTGGAGATGGAT
GCTGCACCTGGCGTGGACCTGAGCCGCATCCTGAATGAGATGCGTGACCAGTACGAGCAGATGGCAGAGAAAAACCG
CAGAGACGCTGAGACCTGGTTCCTGAGCAAGACCGAGGAGCTGAACAAAGAAGTGGCCTCCAACAGCGAACTGGTAC
AGAGCAGCCGCAGTGAGGTGACGGAGCTCCGGAGGGTGCTCCAGGGCCTGGAGATTGAGCTGCAGTCCCAGCTCAGC
ATGAAAGCATCCCTGGAGAACAGCCTGGAGGAGACCAAAGGCCGCTACTGCATGCAGCTGTCCCAGATCCAGGGACTG
ATTGGCAGTGTGGAGGAGCAGCTGGCCCAGCTACGCTGTGAGATGGAGCAGCAGAGCCAGGAGTACCAGATCTTGCT
GGATGTGAAGACGCGGCTGGAGCAGGAGATTGCCACCTACCGCCGCCTGCTGGAGGGCGAGGATGCCCACCTTTCCTC
CCAGCAAGCATCTGGCCAATCCTATTCTTCCCGCGAGGTCTTCACCTCCTCCTCGTCCTCTTCGAGCCGTCAGACCCGGCC
CATCCTCAAGGAGCAGAGCTCATCCAGCTTCAGCCAGGGCCAGAGCTCCTAGAACTGAGCTGCCTCTACCACAGCCTCC
TGCCCACCAGCTGGCCTCACCTCCTGAAGGCCCGGGTCAGGACCCTGCTCTCCTGGCGCAGTTCCCAGCTATCTCCCCTG
CTCCTCTGCTGGTGGTGGGCTAATAAAGCTgactttctggttgat

>VIRT41599
MQIEGLKEELAYLRKNHEEEMLALRGQTGGDVNVEMDAAPGVDLSRILNEMRDQYEQMAE
KNRRDAETWFLSKTEELNKEVASNSELVQSSRSEVTELRRVLQGLEIELQSQLSMKASLE

```
NSLEETKGRYCMQLSQIQGLIGSVEEQLAQLRCEMEQQSQEYQILLDVKTRLEQEIATYR
RLLEGEDAHLSSQQASGQSYSSREVFTSSSSSSSRQTRPILKEQSSSSFSQGQSS
```

```
>NM_005557.3 Homo sapiens keratin 16 (KRT16), mRNA
AGTTAGGAGGGCCCCGCCTTCCCCAGCTGCATATAAAGGTCTCTGGGGTTGGAGGCAGCCACAGCACGCT
CTCAGCCTTCCTGAGCACCTTTCCTTCTTTCAGCCAACTGCTCACTCGCTCACCTCCCTCCTTGGCACCA
TGACCACCTGCAGCCGCCAGTTCACCTCCTCCAGCTCCATGAAGGGCTCCTGCGGCATCGGAGGCGGCAT
CGGGGGCGGCTCCAGCCGCATCTCCTCCGTCCTGGCCGGAGGGTCCTGCCGTGCCCCCAGCACCTACGGG
GGCGGCCTGTCTGTCTCCTCTCGCTTCTCCTCTGGGGGAGCCTGCGGGCTGGGGGGCGGCTATGGCGGTG
GCTTCAGCAGCAGCAGCAGCTTTGGTAGTGGCTTCGGGGGAGGATATGGTGGTGGCCTTGGTGCTGGCTT
CGGTGGTGGCTTGGGTGCTGGCTTTGGTGGTGGTTTTGCTGGTGGTGATGGGCTTCTGGTGGGCAGTGAG
AAGGTGACCATGCAGAACCTCAATGACCGCCTGGCCTCCTACCTGGACAAGGTGCGTGCTCTGGAGGAGG
CCAACGCCGACCTGGAAGTGAAGATCCGTGACTGGTACCAGAGGCAGCGGCCCAGTGAGATCAAAGACTA
CAGTCCCTACTTCAAGACCATCGAGGACCTGAGGAACAAGATCATTGCGGCCACCATTGAGAATGCGCAG
CCCATTTTGCAGATTGACAATGCCAGGCTGGCAGCCGATGACTTCAGGACCAAGTATGAGCATGAACTGG
CCCTGCGGCAGACTGTGGAGGCCGACGTCAATGGCCTGCGCCGGGTGTTGGATGAGCTGACCCTGGCCAG
GACTGACCTGGAGATGCAGATCGAAGGCCTGAAGGAGGAGCTGGCCTACCTGAGGAAGAACCACGAGGAG
GAGATGCTTGCTCTGAGAGGTCAGACCGGCGGAGATGTGAACGTGGAGATGGATGCTGCACCTGGCGTGG
ACCTGAGCCGCATCCTGAATGAGATGCGTGACCAGTACGAGCAGATGGCAGAGAAAAACCGCAGAGACGC
TGAGACCTGGTTCCTGAGCAAGACCGAGGAGCTGAACAAAGAAGTGGCCTCCAACAGCGAACTGGTACAG
AGCAGCCGCAGTGAGGTGACGGAGCTCCGGAGGGTGCTCCAGGGCCTGGAGATTGAGCTGCAGTCCCAGC
TCAGCATGAAAGCATCCCTGGAGAACAGCCTGGAGGAGACCAAAGGCCGCTACTGCATGCAGCTGTCCCA
GATCCAGGGACTGATTGGCAGTGTGGAGGAGCAGCTGGCCCAGCTACGCTGTGAGATGGAGCAGCAGAGC
CAGGAGTACCAGATCTTGCTGGATGTGAAGACGCGGCTGGAGCAGGAGATTGCCACCTACCGCCGCCTGC
TGGAGGGCGAGGATGCCCACCTTTCCTCCCAGCAAGCATCTGGCCAATCCTATTCTTCCCGCGAGGTCTT
CACCTCCTCCTCGTCCTCTTCGAGCCGTCAGACCCGGCCCATCCTCAAGGAGCAGAGCTCATCCAGCTTC
AGCCAGGGCCAGAGCTCCTAGAACTGAGCTGCCTCTACCACAGCCTCCTGCCCACCAGCTGGCCTCACCT
CCTGAAGGCCCGGGTCAGGACCCTGCTCTCCTGGCGCAGTTCCCAGCTATCTCCCCTGCTCCTCTGCTGG
TGGTGGGCTAATAAAGCTGACTTTCTGGTTGATGCAAAAA
>NP_005548.2 keratin, type I cytoskeletal 16 [Homo sapiens]
MTTCSRQFTSSSSMKGSCGIGGGIGGGSSRISSVLAGGSCRAPSTYGGGLSVSSRFSSGGACGLGGGYGG
GFSSSSSFGSGFGGGYGGGLGAGFGGGLGAGFGGGFAGGDGLLVGSEKVTMQNLNDRLASYLDKVRALEE
ANADLEVKIRDWYQRQRPSEIKDYSPYFKTIEDLRNKIIAATIENAQPILQIDNARLAADDFRTKYEHEL
ALRQTVEADVNGLRRVLDELTLARTDLEMQIEGLKEELAYLRKNHEEEMLALRGQTGGDVNVEMDAAPGV
DLSRILNEMRDQYEQMAEKNRRDAETWFLSKTEELNKEVASNSELVQSSRSEVTELRRVLQGLEIELQSQ
LSMKASLENSLEETKGRYCMQLSQIQGLIGSVEEQLAQLRCEMEQQSQEYQILLDVKTRLEQEIATYRRL
LEGEDAHLSSQQASGQSYSSREVFTSSSSSSSRQTRPILKEQSSSSFSQGQSS
```

```
i1_HQ_samplec9816d|c1544/f10p0/1305      ------------------------------------------------------------ 0
NM_005557.3                              AGTTAGGAGGGCCCCGCCTTCCCCAGCTGCATATAAAGGTCTCTGGGGTTGGAGGCAGCC 60
i1_HQ_samplec9816d|c42043/f3p0/1040      ------------------------------------------------------------ 0
i1_HQ_samplec9816d|c55807/f2p0/1013      ------------------------------------------------------------ 0
i1_HQ_samplec9816d|c63528/f5p0/1150      ------------------------------------------------------------ 0

i1_HQ_samplec9816d|c1544/f10p0/1305      ------------------------------------------------------------ 0
NM_005557.3                              ACAGCACGCTCTCAGCCTTCCTGAGCACCTTTCCTTCTTTCAGCCAACTGCTCACTCGCT 120
i1_HQ_samplec9816d|c42043/f3p0/1040      ------------------------------------------------------------ 0
i1_HQ_samplec9816d|c55807/f2p0/1013      ------------------------------------------------------------ 0
i1_HQ_samplec9816d|c63528/f5p0/1150      ------------------------------------------------------------ 0

i1_HQ_samplec9816d|c1544/f10p0/1305      ------------------------------------------------------------ 0
NM_005557.3                              CACCTCCCTCCTTGGCACCATGACCACCTGCAGCCGCCAGTTCACCTCCTCCAGCTCCAT 180
i1_HQ_samplec9816d|c42043/f3p0/1040      ------------------------------------------------------------ 0
i1_HQ_samplec9816d|c55807/f2p0/1013      ------------------------------------------------------------ 0
i1_HQ_samplec9816d|c63528/f5p0/1150      ------------------------------------------------------------ 0

i1_HQ_samplec9816d|c1544/f10p0/1305      ------------------------------------------------------------ 0
NM_005557.3                              GAAGGGCTCCTGCGGCATCGGAGGCGGCATCGGGGGCGGCTCCAGCCGCATCTCCTCCGT 240
i1_HQ_samplec9816d|c42043/f3p0/1040      ------------------------------------------------------------ 0
i1_HQ_samplec9816d|c55807/f2p0/1013      ------------------------------------------------------------ 0
i1_HQ_samplec9816d|c63528/f5p0/1150      ------------------------------------------------------------ 0

i1_HQ_samplec9816d|c1544/f10p0/1305      ------------------------------------------------------------ 0
NM_005557.3                              CCTGGCCGGAGGGTCCTGCCGTGCCCCCAGCACCTACGGGGGCGGCCTGTCTGTCTCCTC 300
i1_HQ_samplec9816d|c42043/f3p0/1040      ------------------------------------------------------------ 0
i1_HQ_samplec9816d|c55807/f2p0/1013      ------------------------------------------------------------ 0
i1_HQ_samplec9816d|c63528/f5p0/1150      ------------------------------------------------------------ 0

i1_HQ_samplec9816d|c1544/f10p0/1305      ------------------------------------------------------------ 0
NM_005557.3                              TCGCTTCTCCTCTGGGGGAGCCTGCGGGCTGGGGGGCGGCTATGGCGGTGGCTTCAGCAG 360
i1_HQ_samplec9816d|c42043/f3p0/1040      ------------------------------------------------------------ 0
```

```
i1_HQ_samplec9816d|c55807/f2p0/1013      ------------------------------------------------------------ 0
i1_HQ_samplec9816d|c63528/f5p0/1150      ------------------------------------------------------------ 0


i1_HQ_samplec9816d|c1544/f10p0/1305      ----------------------------------acacacagcagagttgatcctgggct 26
NM_005557.3                              CAGCAGCAGCTTTGGTAGTGGCTTCGGGGGAGGATATGGTGGTGGCCTTGGTGCTGGCTT 420
i1_HQ_samplec9816d|c42043/f3p0/1040      ------------------------------------------------------------ 0
i1_HQ_samplec9816d|c55807/f2p0/1013      ------------------------------------------------------------ 0
i1_HQ_samplec9816d|c63528/f5p0/1150      ------------------------------------------------------------ 0


i1_HQ_samplec9816d|c1544/f10p0/1305      gaataatcc---------agagtgaggagttggacgggaccgggaGTGATGAAAT----- 72
NM_005557.3                              CGGTGGTGGCTTGGGTGCTGGCTTTGGTGGTGGTTT-TGCTGGTGGTGATGGGCTTCTGG 479
i1_HQ_samplec9816d|c42043/f3p0/1040      ------------------------------------------------------------ 0
i1_HQ_samplec9816d|c55807/f2p0/1013      ------------------------------------------------------------ 0
i1_HQ_samplec9816d|c63528/f5p0/1150      ------------------------------------------------------------ 0


i1_HQ_samplec9816d|c1544/f10p0/1305      -CCAGAGGGGAACCTGGAGTCAGCAGTTAGGAGGGCCCCGCCTTCCCCAGCTGCATATAA 131
NM_005557.3                              TGGGCAGTGAGAAGGTGACCATGCAGAACCTCAATGACCGCCTGGCCTCCTACCTGGACA 539
i1_HQ_samplec9816d|c42043/f3p0/1040      ------------------------------------------------------------ 0
i1_HQ_samplec9816d|c55807/f2p0/1013      ------------------------------------------------------------ 0
i1_HQ_samplec9816d|c63528/f5p0/1150      --------------------------------------------------aacgtGGAGA 10


i1_HQ_samplec9816d|c1544/f10p0/1305      AGGTGCGTG-CTCTGGAGGAGGC--CAACGCCGACCTGGAAGTGAAGATCCGTGACTGGT 188
NM_005557.3                              AGGTGCGTG-CTCTGGAGGAGGC--CAACGCCGACCTGGAAGTGAAGATCCGTGACTGGT 596
i1_HQ_samplec9816d|c42043/f3p0/1040      ------------------------------------------------------------ 0
i1_HQ_samplec9816d|c55807/f2p0/1013      ------------------------------------------------------------ 0
i1_HQ_samplec9816d|c63528/f5p0/1150      TGGATGCTGCACCTGGCGTGGACCTGAGCCGCATCCTGAATGAGATGCGC---A-CCAGT 66


i1_HQ_samplec9816d|c1544/f10p0/1305      ACCAGAGGCAGCGGCCCAGTGAGATCAAAGACTACAGTCCCTACTTCAAGACCATCGAGG 248
NM_005557.3                              ACCAGAGGCAGCGGCCCAGTGAGATCAAAGACTACAGTCCCTACTTCAAGACCATCGAGG 656
i1_HQ_samplec9816d|c42043/f3p0/1040      ------------------------------------------------------------ 0
i1_HQ_samplec9816d|c55807/f2p0/1013      ------------------------------------------------------------ 0
i1_HQ_samplec9816d|c63528/f5p0/1150      ACGAGCAGATGGCAGAGAAAAACCGCAGAGACGCTGAGACCTGGTTCCTGAGCAAGGTGG 126


i1_HQ_samplec9816d|c1544/f10p0/1305      ACCTGAGGAACAAGATCATTGCGGCC------------ACCATTGAGAATGCGCAGCC-- 294
NM_005557.3                              ACCTGAGGAACAAGATCATTGCGGCC------------ACCATTGAGAATGCGCAGCC-- 702
i1_HQ_samplec9816d|c42043/f3p0/1040      ------------------------------------------------------------ 0
i1_HQ_samplec9816d|c55807/f2p0/1013      ----------atgcgtgaccagtac-----gagcagatggcagagaaaaaccgcagagac 45
i1_HQ_samplec9816d|c63528/f5p0/1150      GGCTCGGGCCCGCAGTGGCCTGCAGCACTTCCCAGCTGGGGGCTTTGGGAGAGCCTCACC 186


i1_HQ_samplec9816d|c1544/f10p0/1305      --------CATTTTGCAGATTGACAATGCCAGGCTGGCAGCCGATGACTTCAGGACCAAG 346
NM_005557.3                              --------CATTTTGCAGATTGACAATGCCAGGCTGGCAGCCGATGACTTCAGGACCAAG 754
i1_HQ_samplec9816d|c42043/f3p0/1040      ------------------------------------------------------------ 0
i1_HQ_samplec9816d|c55807/f2p0/1013      gctgagacctggttCCTGAGCAAGACCGAGGAGCTGAAC---AAAGAAGTGGCCTCCAAC 102
i1_HQ_samplec9816d|c63528/f5p0/1150      TTTCACTCTGCTTTCCTGCCTCAGACCGAGGAGCTGAAC---AAAGAAGTGGCCTCCAAC 243


i1_HQ_samplec9816d|c1544/f10p0/1305      TATGAGCACGAACTGGCCCTGCGGCAGACTGTGGAGGCCGACGTCAATGGCCTGCGCCGG 406
NM_005557.3                              TATGAGCATGAACTGGCCCTGCGGCAGACTGTGGAGGCCGACGTCAATGGCCTGCGCCGG 814
i1_HQ_samplec9816d|c42043/f3p0/1040      ------------------------------------------gtgacggagctccggagg 18
i1_HQ_samplec9816d|c55807/f2p0/1013      AGCGA------AC------TGGTACAGAGCAGCCGCAGTGAGGTGACGGAGCTCCGGAGG 150
i1_HQ_samplec9816d|c63528/f5p0/1150      AGCGA------AC------TGGTACAGAGCAGCCGCAGTGAGGTGACGGAGCTCCGGAGG 291
                                                                                   ** *  *  ** **  **

i1_HQ_samplec9816d|c1544/f10p0/1305      GTGTTGGATGAGCTGACCCTGGCCAGGACTGACCTGGAGATGCAGATCGAAGGCCTGAAG 466
NM_005557.3                              GTGTTGGATGAGCTGACCCTGGCCAGGACTGACCTGGAGATGCAGATCGAAGGCCTGAAG 874
i1_HQ_samplec9816d|c42043/f3p0/1040      gtgctccagggcctgga---------gattgagctgcagtcccagctc------agcatG 63
i1_HQ_samplec9816d|c55807/f2p0/1013      GTGCTCCAGGGCCTGGA---------GATTGAGCTGCAGTCCCAGCTC------AGCATG 195
i1_HQ_samplec9816d|c63528/f5p0/1150      GTGCTCCAGGGCCTGGA---------GATTGAGCTGCAGTCCCAGCTC------AGCATG 336
                                         *** *  * *  ***           ** *** *** **   *** **         * *

i1_HQ_samplec9816d|c1544/f10p0/1305      GAGGAGCTGGCCTACCTGAGGAAGAACCACGAGGAGGAGATGCTTGCTCTGAGAGGTCAG 526
NM_005557.3                              GAGGAGCTGGCCTACCTGAGGAAGAACCACGAGGAGGAGATGCTTGCTCTGAGAGGTCAG 934
i1_HQ_samplec9816d|c42043/f3p0/1040      GTATGAAGGACCCAGCACAGCAGCAGCCCCCAAGTCACCAG--------TAATGGCCACC 115
i1_HQ_samplec9816d|c55807/f2p0/1013      GTATGAAGGACCCAGCACAGCAGCAGCCCCCAAGTCACCAG--------TAATGGCCACC 247
i1_HQ_samplec9816d|c63528/f5p0/1150      GTATGAAGGACCCAGCACAGCAGCAGCCCCCAAGTCACCAG--------TAATGGCCACC 388
                                         *       * ** * *  ** *  * ** * * *     *         * *  *

i1_HQ_samplec9816d|c1544/f10p0/1305      ACCGGC-GGAGATGTGAACGTGGAGATGGATGCTGCACCTGGCGTGGACCTGAGCCGCAT 585
NM_005557.3                              ACCGGC-GGAGATGTGAACGTGGAGATGGATGCTGCACCTGGCGTGGACCTGAGCCGCAT 993
i1_HQ_samplec9816d|c42043/f3p0/1040      ACCCCCTCAAAAAGCCACAGTCTAGT-------TCCACCTTTCTTTTCTCAGGATGGGAC 168
i1_HQ_samplec9816d|c55807/f2p0/1013      ACCCCCTCAAAAAGCCACAGTCTAGT-------TCCACCTTTCTTTTCTCAGGATGGGAC 300
i1_HQ_samplec9816d|c63528/f5p0/1150      ACCCCCTCAAAAAGCCACAGTCTAGT-------TCCACCTTTCTTTTCTCAGGATGGGAC 441
                                         ***  *   * * *  *  **  **        * *****  * *    * *    * *

i1_HQ_samplec9816d|c1544/f10p0/1305      CCTGAATGAGATGCGTGACCAGTACGAGCAGATGGCAGAGAAAAACCGCAGAGACGCTGA 645
```

```
NM_005557.3                              CCTGAATGAGATGCGTGACCAGTACGAGCAGATGGCAGAGAAAAACCGCAGAGACGCTGA 1053
i1_HQ_samplec9816d|c42043/f3p0/1040      CAGGGGACTCAT--GGGACCGTTAT--ATAGATAGAGAAACTAAGCCCTAGAAT-AGTGG 223
i1_HQ_samplec9816d|c55807/f2p0/1013      CAGGGGACTCAT--GGGACCGTTAT--ATAGATAGAGAAACTAAGCCCTAGAAT-AGTGG 355
i1_HQ_samplec9816d|c63528/f5p0/1150      CAGGGGACTCAT--GGGACCGTTAT--ATAGATAGAGAAACTAAGCCCTAGAAT-AGTGG 496
                                         *  *      **  * ****  **     **** *   *   ** **  ***     **

i1_HQ_samplec9816d|c1544/f10p0/1305      GACCTGGTTCCTGAGCAAGACCGAGGAGCTGAAC-AAAGAAGTGGCCTC---CAACAGCG 701
NM_005557.3                              GACCTGGTTCCTGAGCAAGACCGAGGAGCTGAAC-AAAGAAGTGGCCTC---CAACAGCG 1109
i1_HQ_samplec9816d|c42043/f3p0/1040      GCTAGCTTTTCTCCATATTGTCTGGCCCATCAGTACCCCAACTGGGATCAAAATCCAGGC 283
i1_HQ_samplec9816d|c55807/f2p0/1013      GCTAGCTTTTCTCCATATTGTCTGGCCCATCAGTACCCCAACTGGGATCAAAATCCAGGC 415
i1_HQ_samplec9816d|c63528/f5p0/1150      GCTAGCTTTTCTCCATATTGTCTGGCCCATCAGTACCCCAACTGGGATCAAAATCCAGGC 556
                                         *      ** **    *    *  *    * *       ** ***  **      ***

i1_HQ_samplec9816d|c1544/f10p0/1305      AACTGGTACAGAGCAGCCGCAGTGAGGTGACGGAGCTCCGGAGGGTGCTCCAGGGCCTGG 761
NM_005557.3                              AACTGGTACAGAGCAGCCGCAGTGAGGTGACGGAGCTCCGGAGGGTGCTCCAGGGCCTGG 1169
i1_HQ_samplec9816d|c42043/f3p0/1040      ATCTCTCAAAAAACATGCCCAGAGACCTGGAGGAACAGG--AG--TGACCA---CCTCCA 336
i1_HQ_samplec9816d|c55807/f2p0/1013      ATCTCTCAAAAAACATGCCCAGAGACCTGGAGGAACAGG--AG--TGACCA---CCTCCA 468
i1_HQ_samplec9816d|c63528/f5p0/1150      ATCTCTCAAAAAACATGCCCAGAGACCTGGAGGAACAGG--AG--TGACCA---CCTCCA 609
                                         * **   * * * **  * *** **  **  *** *     **  **  *     *

i1_HQ_samplec9816d|c1544/f10p0/1305      AGATTGAGCTGCAGTCCCAGCTCAGCATGAAAGCATCCCTGGAGAACAGCCTGGAGGAGA 821
NM_005557.3                              AGATTGAGCTGCAGTCCCAGCTCAGCATGAAAGCATCCCTGGAGAACAGCCTGGAGGAGA 1229
i1_HQ_samplec9816d|c42043/f3p0/1040      TGGACTCTTTTTCTCTCTCACTTGCAGAAAGCATCCCTGGAGAACAGCCTGGAGGAGA 396
i1_HQ_samplec9816d|c55807/f2p0/1013      TGGACTCTTTTTCTCTCTCACTTGCAGAAAGCATCCCTGGAGAACAGCCTGGAGGAGA 528
i1_HQ_samplec9816d|c63528/f5p0/1150      TGGACTCTTTTTCTCTCTCACTTGCAGAAAGCATCCCTGGAGAACAGCCTGGAGGAGA 669
                                          *       *      *   * *     ********************************

i1_HQ_samplec9816d|c1544/f10p0/1305      CCAAAGGCCGCTACTGCATGCAGCTGTCCCAGATCCAGGGACTGATTGGCAGTGTGGAGG 881
NM_005557.3                              CCAAAGGCCGCTACTGCATGCAGCTGTCCCAGATCCAGGGACTGATTGGCAGTGTGGAGG 1289
i1_HQ_samplec9816d|c42043/f3p0/1040      CCAAAGGCCGCTACTGCATGCAGCTGTCCCAGATCCAGGGACTGATTGGCAGTGTGGAGG 456
i1_HQ_samplec9816d|c55807/f2p0/1013      CCAAAGGCCGCTACTGCATGCAGCTGTCCCAGATCCAGGGACTGATTGGCAGTGTGGAGG 588
i1_HQ_samplec9816d|c63528/f5p0/1150      CCAAAGGCCGCTACTGCATGCAGCTGTCCCAGATCCAGGGACTGATTGGCAGTGTGGAGG 729
                                         ************************************************************

i1_HQ_samplec9816d|c1544/f10p0/1305      AGCAGCTGGCCCAGCTACGCTGTGAGATGGAGCAGCAGAGCCAGGAGTACCAGATCTTGC 941
NM_005557.3                              AGCAGCTGGCCCAGCTACGCTGTGAGATGGAGCAGCAGAGCCAGGAGTACCAGATCTTGC 1349
i1_HQ_samplec9816d|c42043/f3p0/1040      AGCAGCTGGCCCAGCTACGCTGTGAGATGGAGCAGCAGAGCCAGGAGTACCAGATCTTGC 516
i1_HQ_samplec9816d|c55807/f2p0/1013      AGCAGCTGGCCCAGCTACGCTGTGAGATGGAGCAGCAGAGCCAGGAGTACCAGATCTTGC 648
i1_HQ_samplec9816d|c63528/f5p0/1150      AGCAGCTGGCCCAGCTACGCTGTGAGATGGAGCAGCAGAGCCAGGAGTACCAGATCTTGC 789
                                         ************************************************************

i1_HQ_samplec9816d|c1544/f10p0/1305      TGGATGTGAAGACGCGGCTGGAGCAGGAGATTGCCACCTACCGCCGCCTGCTGGAGGGCG 1001
NM_005557.3                              TGGATGTGAAGACGCGGCTGGAGCAGGAGATTGCCACCTACCGCCGCCTGCTGGAGGGCG 1409
i1_HQ_samplec9816d|c42043/f3p0/1040      TGGATGTGAAGACGCGGCTGGAGCAGGAGATTGCCACCTACCGCCGCCTGCTGGAGGGCG 576
i1_HQ_samplec9816d|c55807/f2p0/1013      TGGATGTGAAGACGCGGCTGGAGCAGGAGATTGCCACCTACCGCCGCCTGCTGGAGGGCG 708
i1_HQ_samplec9816d|c63528/f5p0/1150      TGGATGTGAAGACGCGGCTGGAGCAGGAGATTGCCACCTACCGCCGCCTGCTGGAGGGCG 849
                                         ************************************************************

i1_HQ_samplec9816d|c1544/f10p0/1305      AGGATGCCCACCTTTCCTCCCAGCAAGCATCTGGCCAATCCTATTCTTCCCGCGAG---- 1057
NM_005557.3                              AGGATGCCCACCTTTCCTCCCAGCAAGCATCTGGCCAATCCTATTCTTCCCGCGAG---- 1465
i1_HQ_samplec9816d|c42043/f3p0/1040      AGGATGCCCACCTTTCCTCCCAGCAAGCATCTGGCCAATCCTATTCTTCCCGCGAGGGTA 636
i1_HQ_samplec9816d|c55807/f2p0/1013      AGGATGCCCACCTTTCCTCCCAGCAAGCATCTGGCCAATCCTATTCTTCCCGCGAG---- 764
i1_HQ_samplec9816d|c63528/f5p0/1150      AGGATGCCCACCTTTCCTCCCAGCAAGCATCTGGCCAATCCTATTCTTCCCGCGAG---- 905
                                         ********************************************************

i1_HQ_samplec9816d|c1544/f10p0/1305      ------------------------------------------------------------ 1057
NM_005557.3                              ------------------------------------------------------------ 1465
i1_HQ_samplec9816d|c42043/f3p0/1040      AGGCTTCTGAGGCTCCCCGGCACTGCAGCCCCTCTGCCTGTTTCCATGGAGTGGGGGCTG 696
i1_HQ_samplec9816d|c55807/f2p0/1013      ------------------------------------------------------------ 764
i1_HQ_samplec9816d|c63528/f5p0/1150      ------------------------------------------------------------ 905

i1_HQ_samplec9816d|c1544/f10p0/1305      ------------------------------------------------------------ 1057
NM_005557.3                              ------------------------------------------------------------ 1465
i1_HQ_samplec9816d|c42043/f3p0/1040      GGCCCTTCTCCTCAGAGCTCCCAGCCCTCCCTTCTCCCTGCCCTGGAGTCAGCTTAGCTC 756
i1_HQ_samplec9816d|c55807/f2p0/1013      ------------------------------------------------------------ 764
i1_HQ_samplec9816d|c63528/f5p0/1150      ------------------------------------------------------------ 905

i1_HQ_samplec9816d|c1544/f10p0/1305      ------------------------------------GTCTTCACCTCCTCCTCGTCCTCT 1081
NM_005557.3                              ------------------------------------GTCTTCACCTCCTCCTCGTCCTCT 1489
i1_HQ_samplec9816d|c42043/f3p0/1040      TCAGACCCCTTCTCACCTCCTCTTCTCTCTCCCACAGTCTTCACCTCCTCCTCGTCCTCT 816
i1_HQ_samplec9816d|c55807/f2p0/1013      ------------------------------------GTCTTCACCTCCTCCTCGTCCTCT 788
i1_HQ_samplec9816d|c63528/f5p0/1150      ------------------------------------GTCTTCACCTCCTCCTCGTCCTCT 929
                                                                             ************************

i1_HQ_samplec9816d|c1544/f10p0/1305      TCGAGCCGTCAGACCCGGCCCATCCTCAAGGAGCAGAGCTCATCCAGCTTCAGCCAGGGC 1141
NM_005557.3                              TCGAGCCGTCAGACCCGGCCCATCCTCAAGGAGCAGAGCTCATCCAGCTTCAGCCAGGGC 1549
i1_HQ_samplec9816d|c42043/f3p0/1040      TCGAGCCGTCAGACCCGGCCCATCCTCAAGGAGCAGAGCTCATCCAGCTTCAGCCAGGGC 876
i1_HQ_samplec9816d|c55807/f2p0/1013      TCGAGCCGTCAGACCCGGCCCATCCTCAAGGAGCAGAGCTCATCCAGCTTCAGCCAGGGC 848
i1_HQ_samplec9816d|c63528/f5p0/1150      TCGAGCCGTCAGACCCGGCCCATCCTCAAGGAGCAGAGCTCATCCAGCTTCAGCCAGGGC 989
                                         ************************************************************
```

```
i1_HQ_samplec9816d|c1544/f10p0/1305      CAGAGCTCCTAGAACTGAGCTGCCTCTACCACAGCCTCCTGCCCACCAGCTGGCCTCACC 1201
NM_005557.3                              CAGAGCTCCTAGAACTGAGCTGCCTCTACCACAGCCTCCTGCCCACCAGCTGGCCTCACC 1609
i1_HQ_samplec9816d|c42043/f3p0/1040      CAGAGCTCCTAGAACTGAGCTGCCTCTACCACAGCCTCCTGCCCACCAGCTGGCCTCACC 936
i1_HQ_samplec9816d|c55807/f2p0/1013      CAGAGCTCCTAGAACTGAGCTGCCTCTACCACAGCCTCCTGCCCACCAGCTGGCCTCACC 908
i1_HQ_samplec9816d|c63528/f5p0/1150      CAGAGCTCCTAGAACTGAGCTGCCTCTACCACAGCCTCCTGCCCACCAGCTGGCCTCACC 1049
                                         ************************************************************

i1_HQ_samplec9816d|c1544/f10p0/1305      TCCTGAAGGCCCGGGTCAGGACCCTGCTCTCCTGGCGCAGTTCCCAGCTATCTCCCCTGC 1261
NM_005557.3                              TCCTGAAGGCCCGGGTCAGGACCCTGCTCTCCTGGCGCAGTTCCCAGCTATCTCCCCTGC 1669
i1_HQ_samplec9816d|c42043/f3p0/1040      TCCTGAAGGCCCGGGTCAGGACCCTGCTCTCCTGGCGCAGTTCCCAGCTATCTCCCCTGC 996
i1_HQ_samplec9816d|c55807/f2p0/1013      TCCTGAAGGCCCGGGTCAGGACCCTGCTCTCCTGGCGCAGTTCCCAGCTATCTCCCCTGC 968
i1_HQ_samplec9816d|c63528/f5p0/1150      TCCTGAAGGCCGGGTCAGGAC-CCTGCTCTCCAG-CGCAGTTCCCA-GCTGTCTCCCTGC 1106
                                         *********** **    *   ********** * ***********        ******

i1_HQ_samplec9816d|c1544/f10p0/1305      TCCTCTGCTGGTGGTGGGCTAATAAAGCTgactttctggttgat-------          1305
NM_005557.3                              TCCTCTGCTGGTGGTGGGCTAATAAAGCTGACTTTCTGGTTGATGCAAAAA          1720
i1_HQ_samplec9816d|c42043/f3p0/1040      TCCTCTGCTggtggtgggctaataaagctgactttctggttgat-------          1040
i1_HQ_samplec9816d|c55807/f2p0/1013      TCCTCTGCTGgtggtgggctaataaagctgactttctggttgatg------          1013
i1_HQ_samplec9816d|c63528/f5p0/1150      TCCTCTGCTGGTGGTGGGctaataaagctgactttctggttgat-------          1150
                                         ********************************************
```

```
VIRT39978          ------------------------MDSFSLSHLQKASLENSLEETKGRY-----------     25
VIRT34198          ------------------------------------------------------------     0
VIRT38686          ------------------------------------------------------------     0
VIRT41599          ------------------------------------------------------------     0
NP_005548.2        MTTCSRQFTSSSSMKGSCGIGGGIGGGSSRISSVLAGGSCRAPSTYGGGLSVSSRFSSGG     60

VIRT39978          -CMQ-----------LS--------------------------QIQGLIGSVEEQ----     42
VIRT34198          ------------------------------------------------------------     0
VIRT38686          ------------------------------------------------------------     0
VIRT41599          ------------------------------------------------------------     0
NP_005548.2        ACGLGGGYGGGFSSSSSFGSGFGGGYGGGLGAGFGGGLGAGFGGGFAGGDGLLVGSEKVT     120

VIRT39978          -------------------LAQLRCEMEQQSQEY------------QILLDVKTRLEQEI     71
VIRT34198          ------------------------------------------------------------     0
VIRT38686          ------------------------------------------------------------     0
VIRT41599          ------------------------------------------------------------     0
NP_005548.2        MQNLNDRLASYLDKVRALEEANADLEVKIRDWYQRQRPSEIKDYSPYFKTIEDLRNKIIA     180

VIRT39978          ATYRRLLEGEDAHLSSQQASGQSYSSREGK------------------------------     101
VIRT34198          ------------------------------------------------------------     0
VIRT38686          ------------------------------------------------------------     0
VIRT41599          ----------------------------------------------------------MQ     2
NP_005548.2        ATIENAQPILQIDNARLAADDFRTKYEHELALRQTVEADVNGLRRVLDELTLARTDLEMQ     240

VIRT39978          --------ASEAPRHCSPSACFHGVGAGPFSSELPALPSPCP-GVSLALRPLLTSSSLSH     152
VIRT34198          ------------------------------------------------------------     0
VIRT38686          ------------------------------------------------------------     0
VIRT41599          IEGLKEELAYLRKNHEEEMLALRGQTGGDVNVEMDAAPGVDLSRILNEMRDQYEQMAEKN     62
NP_005548.2        IEGLKEELAYLRKNHEEEMLALRGQTGGDVNVEMDAAPGVDLSRILNEMRDQYEQMAEKN     300

VIRT39978          SLHLLLVLFEPSDPAHPQGAELIQLQ-------------PGPEL------LELSCLYHS     192
VIRT34198          -------------------------------------------MDSFSLSHLQKASLENS     17
VIRT38686          -------------------------------------------MDSFSLSHLQKASLENS     17
VIRT41599          RRDAETWFLSKTEELNKEVASNSELVQSSRSEVTELRRVLQGLEIELQSQLSMKASLENS     122
NP_005548.2        RRDAETWFLSKTEELNKEVASNSELVQSSRSEVTELRRVLQGLEIELQSQLSMKASLENS     360
                                                                           *  *

VIRT39978          LLPTSWPHLLKARVRTLLSWRSSQLSPLLLCWWWANK-----------------------     229
VIRT34198          LEETKGRYCMQLSQIQGLIGSVEEQLAQLRCEMEQQSQEYQILLDVKTRLEQEIATYRRL     77
VIRT38686          LEETKGRYCMQLSQIQGLIGSVEEQLAQLRCEMEQQSQEYQILLDVKTRLEQEIATYRRL     77
VIRT41599          LEETKGRYCMQLSQIQGLIGSVEEQLAQLRCEMEQQSQEYQILLDVKTRLEQEIATYRRL     182
NP_005548.2        LEETKGRYCMQLSQIQGLIGSVEEQLAQLRCEMEQQSQEYQILLDVKTRLEQEIATYRRL     420
                   *  *             *          * *

VIRT39978          ------------ADFLVD------------------------------------ 235
VIRT34198          LEGEDAHLSSQQASGQSYSSREVFTSSSSSSSRQTRPILKEQSSSSFSQGQSS 130
VIRT38686          LEGEDAHLSSQQASGQSYSSREVFTSSSSSSSRQTRPILKEQSSSSFSQGQSS 130
VIRT41599          LEGEDAHLSSQQASGQSYSSREVFTSSSSSSSRQTRPILKEQSSSSFSQGQSS 235
NP_005548.2        LEGEDAHLSSQQASGQSYSSREVFTSSSSSSSRQTRPILKEQSSSSFSQGQSS 473
                               *
```

**KRT17**

| Sequence | Header | AA Seq |
|---|---|---|
| **Present** | i1_HQ_samplec9816d\|c41456/f18p0/1500 | |
| | i1_HQ_samplec9816d\|c64385/f12p0/1166 | |
| **Absent** | i1_HQ_samplec9816d\|c24577/f2p18/1511 | |
| **NCBI** | NM_000422 | |

>i1_HQ_samplec9816d|c41456/f18p0/1500
isoform=c41456;full_length_coverage=18;non_full_length_coverage=0;isoform_length=1500

aagaaccgcaaggatgccgaggattggttcttcagcaagacagaggaactgaaccgcgaggtgGCCACCAACAGTGAGCTGGTGCAGAGTG
GCAAGAGTGAGATCTCGGAGCTCCGGCGCACCATGCAGGCCTTGGAGATAGAGCTGCAGTCCCAGCTCAGCATGAAA
GCATCCCTGGAGGGCAACCTGGCGGAGACAGAGAACCGCTACTGCGTGCAGCTGTCCCAGATCCAGGGGCTGATTGG
CAGCGTGGAGGAGCAGCTGGCCCAGCTTCGCTGCGAGATGGAGCAGCAGAACCAGGAATACAAAATCCTGCTGGATG
TGAAGACGCGGCTGGAGCAGGAGATTGCCACCTACCGCCGCCTGCTGGAGGGAGAGGATGCCCACCTGACTCAGTAC
AAGAAAGAACGTAAGTATCTGCGTGGCTTCGGCCCTGGGGGTTGGGTGCATGGGACAGGCAGCCCACCTGCACGTTG
CTGGGGCAGGGTCCCCAGGAGCTCCAGGAGTTGATGGCTGTCCCTCAGCAGGGAGAAGTGACTCATTAGCACTGAGG
ATTGATACTCAGGAAAAGAGCAAATGAGAGAGACACTGTCTGGTCTGATGGGGGTGGGGCAGGGAACTGGTCCTTAC
CTTGGAGATCCTAGTCTGATGGAGGAGACAGGTCCCAGCTCTGGAGATTGTCATCTGATGGGAAGATAGGAACATGGT
CTCATGATCTTTGCTCTTGACAGCTTTTGGATGAGCAAAAACAGTCCTGTCTCTGGGGTCTGTAGCCTGATGGGAGATA
GGGACATGGTCCTTGTCCTCCAAATTCCAATCTGATGGAGAATATATGATCCTAGCCTTAGGGTTCCTAGTCTAATGGAG
GAGATAGGGGCCTGGTTTTTGTCTTGGCGATCCCAGTCTGATGGGGGAGATGGTAGAAAATCTATGTCCTAGAGACTG
CAGAGAAATGGAGACGGATTTCATGGAGTCTACATGGCTCCTCCCTGGCAGGACACACTGGATCAGAATCAAATAACC
CATCTGGGGAGGCAAGACTCACACAGGGCCACCGGCAGAGGGATGGGATGGAAGGGAGGTGGTGGCAGGGACAGG
AGGGATGTGTGTGCAGTGTGATGTCGAGGTGCCAGTGGAGGCACTCACAGCACCTGGGGGAGGATGAGGGAGAGAG
CCGGCTCCTGTCCATGAGGGCTAGGGGGCAAGCGAGGGCCTCCTGGCCCCTACCCACTTTAAATTGCCTGCTTCTCCTG
CAGCGGTGACCACCCGTCAGGTGCGTACCATTGTGGAAGAGGTCCAGGATGGCAAGGTCATCTCCTCCCGCGAGCAGG
TCCACCAGACCACCCGCTGAGGACTCAGCTACCCCGGCCGGCCACCCAGGAGGCAGGGAGGCAGCCGCCCCATCTGCC
CCACAGTCTCCGGCCTCTCCAGCCTCAGCCCCCTGCTTCAGTCCCTTCCCCATGCTTCCTTGCCTGATGACAATAaagcttgtt
gactcag

>VIRT63810
MQALEIELQSQLSMKASLEGNLAETENRYCVQLSQIQGLIGSVEEQLAQLRCEMEQQNQE
YKILLDVKTRLEQEIATYRRLLEGEDAHLTQYKKERKYLRGFGPGGWVHGTGSPPARCWG
RVPRSSRS

>i1_HQ_samplec9816d|c64385/f12p0/1166
isoform=c64385;full_length_coverage=12;non_full_length_coverage=0;isoform_length=1166

gggctctcagacccacatctaatttcctctctgttttttttttttctttcagcctgactcagtacAAGAAAGAACGTAAGTATCTGCGTGGCTTCGGC
CCTGGGGGTTGGGTGCATGGGACAGGCAGCCCACCTGCCACGTGCTGGGCAGGGTCCCCAGGAGCTCCAGGAGTTGA
TGGCTGTCCCTCAGCAGGGAGAAGTGACTCATTAGCACTGAGGATTGATACTCAGGAAAAGATCAAATGAGAGAGACA
CTGTCTGGTCTGATGGGGGTGGGGCAGGGAACTGGTCCTTACCTTGGAGATCCTAGTCTGATGGAGGAGACAGGTCCC
AGCTCTGGAGATTGTCATCTGATGGGAAGATAGGAACATGGTCTCATGATCTTTGCTCTTGACAGCTTTTGGATGAGCA
AAAACAGTCCTGTCTCTGGGGTCTGTAGCCTGATGGGAGATAGGGACATGGTCCTTGTCCTCCAAATTCCAATCTGATG
GAGAATATATGATCCTAGCCTTAGGGTTCCTAGTCTAATGGAGGAGATAGGGGCCTGGTTTTTGTCTTGGCGATCCCAG
TCTGATGGGGGAGATGGTAGAAAATCTATGTCCTAGAGACTGCAGAGAAATGGAGACGGATTTCATGGAGTCTACATG
GCTCCTCCCTGGCAGGACACACTGGATCAGAATCAAATAACCCATCTGGGGAGGCAAGACTCACACAGGGCCACCGGC

AGAGGGATGGGATGGAAGGGAGGTGGTGGCAGGGACAGGAGGGATGTGTGTGCAGTGTGATGTCGAGGTGCCAGT
GGAGGCACTCACAGCACCTGGGGGAGGATGAGGGAGAGAGCCGGCTCCTGTCCATGAGGGCTAGGGGGCAAGCGAG
GGCCTCCTGGCCCCTACCCACTTTAAATTGCCTGCTTCTCCTGCAGCGGTGACCACCCGTCAGGTGCGTACCATTGTGGA
AGAGGTCCAGGATGGCAAGGTCATCTCCTCCCGCGAGCAGGTCCACCAGACCACCCGCTGAGGACTCAGCTACCCCGG
CCGGCCACCCAGGAGGCAGGGAGGCAGCCGCCCCATCTGCCCCACAGTCTCCGGCCTCTCCAGCCTCAGCCCCCTGCTT
CAGTCCCTTCCCCATGCTTCCTTGCCTGATGGACAATTAAAGCTTGTTGactcag

>VIRT65337
MAPPWQDTLDQNQITHLGRQDSHRATGRGMGWKGGGGRDRRDVCAV

>i1_HQ_samplec9816d|c24577/f2p18/1511
isoform=c24577;full_length_coverage=2;non_full_length_coverage=18;isoform_length=1511

ctacacaacttggggcccctctcctctccagcccttctcctgtgtgcCTGCCTGCCGCCGCCACCATGACCACCTCCATCCGCCAGTTCACC
TCCTCCAGCTCCATCAAGGGCTCCTCCGGCCTGGGGGGCGGCTCGTCCCGCACCTCCTGCCGGCTGTCTGGCGGCCTGG
GTGCCGGCTCCTGCAGGCTGGGATCTGCTGGCGGCCTGGGCAGCACCCTCGGGGGTAGCAGCTACTCCAGCTGCTACA
GCTTTGGCTCTGGTGGTGGCTATGGCAGCAGCTTTGGGGGTGTTGATGGGCTGCTGGCTGGAGGTGAGAAGGCCACC
ATGCAGAACCTCAATGACCGCCTGGCCTCCTACCTGGACAAGGTGCGTGCCCTGGAGGAGGCCAACACTGAGCTGGAG
GTGAAGATCCGTGACTGGTACCAGAGGCAGGCCCCGGGGCCCGCCCGTGACTACAGCCAGTACTACAGGACAATTGA
GGAGCTGCAGAACAAGATCCTCACAGCCACCGTGGACAATGCCAACATCCTGCTACAGATTGACAATGCCCGTCTGGCT
GCTGATGACTTCCGCACCAAGTTTGAGACAGAGCAGGCCCTGCGCCTGAGTGTGGAGGCCGACATCAATGGCCTGCGC
AGGGTGCTGGATGAGCTGACCCTGGCCAGAGCCGACCTGGAGATGCAGATTGAGAACCTCAAGGAGGAGCTGGCCTA
CCTGAAGAAGAACCACGAGGAGGAGATGAACGCCCTGCGAGGCCAGGTGGGTGGTGAGATCAATGTGGAGATGGAC
GCTGCCCCAGGCGTGGACCTGAGCCGCATCCTCAACGAGATGCGTGACCAGTATGAGAAGATGGCAGAGAAGAACCG
CAAGGATGCCGAGGATTGGTTCTTCAGCAAGACAGAGGAACTGAACCGCGAGGTGGCCACCAACAGTGAGCTGGTGC
AGAGTGGCAAGAGTGAGATCTCGGAGCTCCGGCGCACCATGCAGGCCTTGGAGATAGAGCTGCAGTCCCAGCTCAGC
ATGAAAGCATCCCTGGAGGGCAACCTGGCGGAGACAGAGAACCGCTACTGCGTGCAGCTGTCCCAGATCCAGGGGCT
GATTGGCAGCGTGGAGGAGCAGCTGGCCCAGCTTCGCTGCGAGATGGAGCAGCAGAACCAGGAATACAAAATCCTGC
TGGATGTGAAGACGCGGCTGGAGCAGGAGATTGCCACCTACCGCCGCCTGCTGGAGGGAGAGGATGCCCACCTGACT
CAGTACAAGAAAGAACCGGTGACCACCCGTCAGGTGCGTACCATTGTGGAAGAGGTCCAGGATGGCAAGGTCATCTCC
TCCCGCGAGCAGGTCCACCAGACCACCCGCTGAGGACTCAGCTACCCCGGCCGGCCACCCAGGAGGCAGGGAGGCAG
CCGCCCCATCTGCCCCACAGTCTCCGGCCTCTCCAGCCTCAGCCCCCTGCTTCAGTCCCTTCCCCATGCTTCCTTGCCTGAT
gacaataaagcttgttgactc

>VIRT66411
MTTSIRQFTSSSSIKGSSGLGGGSSRTSCRLSGGLGAGSCRLGSAGGLGSTLGGSSYSSC
YSFGSGGGYGSSFGGVDGLLAGGEKATMQNLNDRLASYLDKVRALEEANTELEVKIRDWY
QRQAPGPARDYSQYYRTIEELQNKILTATVDNANILLQIDNARLAADDFRTKFETEQALR
LSVEADINGLRRVLDELTLARADLEMQIENLKEELAYLKKNHEEEMNALRGQVGGEINVE
MDAAPGVDLSRILNEMRDQYEKMAEKNRKDAEDWFFSKTEELNREVATNSELVQSGKSEI
SELRRTMQALEIELQSQLSMKASLEGNLAETENRYCVQLSQIQGLIGSVEEQLAQLRCEM
EQQNQEYKILLDVKTRLEQEIATYRRLLEGEDAHLTQYKKEPVTTRQVRTIVEEVQDGKV
ISSREQVHQTTR
>NM_000422.2 Homo sapiens keratin 17 (KRT17), mRNA
ATCGCTACGCCCACTTGGTGGCCTATAAAGGAAGCGGGCGAACCCCGGCAGCCCTACACAACTTGGGGCC
CCTCTCCTCTCCAGCCCTTCTCCTGTGTGCCTGCCTCCTGCCGCCGCCACCATGACCACCTCCATCCGCC
AGTTCACCTCCTCCAGCTCCATCAAGGGCTCCTCCGGCCTGGGGGGCGGCTCGTCCCGCACCTCCTGCCG
GCTGTCTGGCGGCCTGGGTGCCGGCTCCTGCAGGCTGGGATCTGCTGGCGGCCTGGGCAGCACCCTCGGG
GGTAGCAGCTACTCCAGCTGCTACAGCTTTGGCTCTGGTGGTGGCTATGGCAGCAGCTTTGGGGGTGTTG

```
ATGGGCTGCTGGCTGGAGGTGAGAAGGCCACCATGCAGAACCTCAATGACCGCCTGGCCTCCTACCTGGA
CAAGGTGCGTGCCCTGGAGGAGGCCAACACTGAGCTGGAGGTGAAGATCCGTGACTGGTACCAGAGGCAG
GCCCCGGGGCCCGCCCGTGACTACAGCCAGTACTACAGGACAATTGAGGAGCTGCAGAACAAGATCCTCA
CAGCCACCGTGGACAATGCCAACATCCTGCTACAGATTGACAATGCCCGTCTGGCTGCTGATGACTTCCG
CACCAAGTTTGAGACAGAGCAGGCCCTGCGCCTGAGTGTGGAGGCCGACATCAATGGCCTGCGCAGGGTG
CTGGATGAGCTGACCCTGGCCAGAGCCGACCTGGAGATGCAGATTGAGAACCTCAAGGAGGAGCTGGCCT
ACCTGAAGAAGAACCACGAGGAGGAGATGAACGCCCTGCGAGGCCAGGTGGGTGGTGAGATCAATGTGGA
GATGGACGCTGCCCCAGGCGTGGACCTGAGCCGCATCCTCAACGAGATGCGTGACCAGTATGAGAAGATG
GCAGAGAAGAACCGCAAGGATGCCGAGGATTGGTTCTTCAGCAAGACAGAGGAACTGAACCGCGAGGTGG
CCACCAACAGTGAGCTGGTGCAGAGTGGCAAGAGTGAGATCTCGGAGCTCCGGCGCACCATGCAGGCCTT
GGAGATAGAGCTGCAGTCCCAGCTCAGCATGAAAGCATCCCTGGAGGGCAACCTGGCGGAGACAGAGAAC
CGCTACTGCGTGCAGCTGTCCCAGATCCAGGGGCTGATTGGCAGCGTGGAGGAGCAGCTGGCCCAGCTTC
GCTGCGAGATGGAGCAGCAGAACCAGGAATACAAAATCCTGCTGGATGTGAAGACGCGGCTGGAGCAGGA
GATTGCCACCTACCGCCGCCTGCTGGAGGGAGAGGATGCCCACCTGACTCAGTACAAGAAAGAACCGGTG
ACCACCCGTCAGGTGCGTACCATTGTGGAAGAGGTCCAGGATGGCAAGGTCATCTCCTCCCGCGAGCAGG
TCCACCAGACCACCCGCTGAGGACTCAGCTACCCCGGCCGGCCACCCAGGAGGCAGGGAGGCAGCCGCCC
CATCTGCCCCACAGTCTCCGGCCTCTCCAGCCTCAGCCCCCTGCTTCAGTCCCTTCCCCATGCTTCCTTG
CCTGATGACAATAAAGCTTGTTGACTCAGCTATG
>NP_000413.1 keratin, type I cytoskeletal 17 [Homo sapiens]
MTTSIRQFTSSSSIKGSSGLGGGSSRTSCRLSGGLGAGSCRLGSAGGLGSTLGGSSYSSCYSFGSGGGYG
SSFGGVDGLLAGGEKATMQNLNDRLASYLDKVRALEEANTELEVKIRDWYQRQAPGPARDYSQYYRTIEE
LQNKILTATVDNANILLQIDNARLAADDFRTKFETEQALRLSVEADINGLRRVLDELTLARADLEMQIEN
LKEELAYLKKNHEEEMNALRGQVGGEINVEMDAAPGVDLSRILNEMRDQYEKMAEKNRKDAEDWFFSKTE
ELNREVATNSELVQSGKSEISELRRTMQALEIELQSQLSMKASLEGNLAETENRYCVQLSQIQGLIGSVE
EQLAQLRCEMEQQNQEYKILLDVKTRLEQEIATYRRLLEGEDAHLTQYKKEPVTTRQVRTIVEEVQDGKV
ISSREQVHQTTR
```

```
i1_HQ_samplec9816d|c24577/f2p18/1511      ------------------------------------------------------------    0
NM_000422.2                               ---------ATCGCTACGCCCACTTGGTGGCCTATAAAGGAAGCGGGCGAACCCCGGCAG    51
i1_HQ_samplec9816d|c41456/f18p0/1500      aagaaccgcaaggatgccgaggattggttcttcagcaagacagaggaactgaaccgcgag    60
i1_HQ_samplec9816d|c64385/f12p0/1166      ------------------------------------------------------------    0

i1_HQ_samplec9816d|c24577/f2p18/1511      --ctaca-caacttggggcccctctcctctccagcccttctcctgtgtgcCTGCCTGC--    55
NM_000422.2                               CCCTACA-CAACTTGGGGCCCCTCTCCTCTCCAGCCCTTCTCCTGTGTGCCTGCCTCCTG    110
i1_HQ_samplec9816d|c41456/f18p0/1500      gtgGCCACCAACAGTGAGCTGGTG-------CAGAGTGGCAAGAGTGAGATCTCGGAGCT    113
i1_HQ_samplec9816d|c64385/f12p0/1166      ------------------------------------------------------------    0

i1_HQ_samplec9816d|c24577/f2p18/1511      -CGCCGCCACCATGACCACCTCCATCCGCCAGTTCACCTCCTCCAGCTCCATCAAGGGCT    114
NM_000422.2                               CCGCCGCCACCATGACCACCTCCATCCGCCAGTTCACCTCCTCCAGCTCCATCAAGGGCT    170
i1_HQ_samplec9816d|c41456/f18p0/1500      -CCGGCGCACCATGCAGGCCTTGGAGATAGAGCTGCAGTCCCAGCTCAGCATGAAAGCAT    172
i1_HQ_samplec9816d|c64385/f12p0/1166      ------------------------------------------------------------    0

i1_HQ_samplec9816d|c24577/f2p18/1511      CCTCC------GGCCTGGGGGGCGGCTCGTCCCGCACCTCCTGCCGGCTGTCTGGCGGCC    168
NM_000422.2                               CCTCC------GGCCTGGGGGGCGGCTCGTCCCGCACCTCCTGCCGGCTGTCTGGCGGCC    224
i1_HQ_samplec9816d|c41456/f18p0/1500      CCCTGGAGGGCAACCTGGCGGAGACAGAGAACCGCTACTGCGTGCAGCTGTCCCAGATCC    232
i1_HQ_samplec9816d|c64385/f12p0/1166      ------------------------------------------------------------    0

i1_HQ_samplec9816d|c24577/f2p18/1511      TGGGTGCCGGCT-------------CCTGCAGGCTGGGATCTGCTGGCGGCCTGGGCAGC    215
NM_000422.2                               TGGGTGCCGGCT-------------CCTGCAGGCTGGGATCTGCTGGCGGCCTGGGCAGC    271
i1_HQ_samplec9816d|c41456/f18p0/1500      AGGGGCTGATTGGCAGCGTGGAGGAGCAGCTGGCCCAGCTTCGCTGCGAGATGGAGCAGC    292
i1_HQ_samplec9816d|c64385/f12p0/1166      ------------------------------------------------------------    0

i1_HQ_samplec9816d|c24577/f2p18/1511      ACCCTCGGGGGTAGCAGCTACTCCAGCTGCTACAGCTTTGGCTCTGGTGGTGGCTATGGC    275
NM_000422.2                               ACCCTCGGGGGTAGCAGCTACTCCAGCTGCTACAGCTTTGGCTCTGGTGGTGGCTATGGC    331
i1_HQ_samplec9816d|c41456/f18p0/1500      AGAACCAGGAATACAAAATCCTGCTGGATGTGAAGACGCGGCTGGAGCAGGAGATTGCCA    352
i1_HQ_samplec9816d|c64385/f12p0/1166      -------------------------------------------gggctctcagacccaca    17
                                                                                              *

i1_HQ_samplec9816d|c24577/f2p18/1511      AGCAGCTTTGGGGGTGTTGATGGGCTGCTGGCTGGAGGTGAGAAGGCCACCATGCAGAAC    335
NM_000422.2                               AGCAGCTTTGGGGGTGTTGATGGGCTGCTGGCTGGAGGTGAGAAGGCCACCATGCAGAAC    391
i1_HQ_samplec9816d|c41456/f18p0/1500      CCTACCGCCGCCTGCTGGAGGGAGAGGATGCC---CACCTGACTCAGTACAAGAAAGAAC    409
i1_HQ_samplec9816d|c64385/f12p0/1166      tctaatttcctctctgtttttttttttctttc---agcctgactcagtacAAGAAAGAAC    74
                                             *                        *  *                ** *   *****

i1_HQ_samplec9816d|c24577/f2p18/1511      CTCAATGACCGCCTGGCCTCCTACCTGGACAAGGTGCGTGCCCTGGAGGAGGCCAACACT    395
NM_000422.2                               CTCAATGACCGCCTGGCCTCCTACCTGGACAAGGTGCGTGCCCTGGAGGAGGCCAACACT    451
i1_HQ_samplec9816d|c41456/f18p0/1500      GTAAGTATCTGCGTGGCTTCGGCCCTGGGGGTTGG-------------------------    444
i1_HQ_samplec9816d|c64385/f12p0/1166      GTAAGTATCTGCGTGGCTTCGGCCCTGGGGGTTGG-------------------------    109
                                           * * *  * ** **** **   *****     *

i1_HQ_samplec9816d|c24577/f2p18/1511      GAGCTGGAGGTGAAGATCCGTGACTGGTACCAGAGGCAGGCCCCGGGGCCCGCCCGTGAC    455
NM_000422.2                               GAGCTGGAGGTGAAGATCCGTGACTGGTACCAGAGGCAGGCCCCGGGGCCCGCCCGTGAC    511
i1_HQ_samplec9816d|c41456/f18p0/1500      --------------------------GTGCATGGGACAGGCAGCC-CACCTGCACGTTGC    477
i1_HQ_samplec9816d|c64385/f12p0/1166      --------------------------GTGCATGGGACAGGCAGCC-CACCTGCCACGTGC    142
                                                                    ** *  * * *****  *    ** **      *

i1_HQ_samplec9816d|c24577/f2p18/1511      TACAGCCAGTACTACAGGACAATTGAGGAGCTGCAGAACAAGATCCTC-ACAGCCACCGT    514
```

```
NM_000422.2                              TACAGCCAGTACTACAGGACAATTGAGGAGCTGCAGAACAAGATCCTC-ACAGCCACCGT    570
i1_HQ_samplec9816d|c41456/f18p0/1500     TGGGGCAGGGTCC----------CCAGGAGCTCCAGGAGTTGATGGCTGTCCCTCAGCAG    527
i1_HQ_samplec9816d|c64385/f12p0/1166     TG-GGCAGGGTCC----------CCAGGAGCTCCAGGAGTTGATGGCTGTCCCTCAGCAG    191
                                         *   **  *  *             ******* *** *   ***      *   ** *

i1_HQ_samplec9816d|c24577/f2p18/1511     G-GACAATGCCAACATCCTGCTACAGATTGACAATGCCCGTCTGGCTGC-TG-----ATG    567
NM_000422.2                              G-GACAATGCCAACATCCTGCTACAGATTGACAATGCCCGTCTGGCTGC-TG-----ATG    623
i1_HQ_samplec9816d|c41456/f18p0/1500     GGAGAAGTGACTCATTAGCACTGAGGATTGATACTCAGGAAAAGAGCAAATGAGAGAGAC    587
i1_HQ_samplec9816d|c64385/f12p0/1166     GGAGAAGTGACTCATTAGCACTGAGGATTGATACTCAGGAAAAGATCAAATGAGAGAGAC    251
                                         *    * ** *    *    **   ****** * *        *      **

i1_HQ_samplec9816d|c24577/f2p18/1511     ACTTCCGCACCAAGTTTGAGACAGAGCAGGCCCTGCGCCTGAGTGTGGAGGCCGACATCA    627
NM_000422.2                              ACTTCCGCACCAAGTTTGAGACAGAGCAGGCCCTGCGCCTGAGTGTGGAGGCCGACATCA    683
i1_HQ_samplec9816d|c41456/f18p0/1500     ACTGTCTGGTCTGATGGGGGTGGGGCAGGGAACTGGTCCTTACCTTGGAGATCCTAGTCT    647
i1_HQ_samplec9816d|c64385/f12p0/1166     ACTGTCTGGTCTGATGGGGGTGGGGCAGGGAACTGGTCCTTACCTTGGAGATCCTAGTCT    311
                                         ***  *    *   *  * *   *    **  ***  *** *   *****  *    **

i1_HQ_samplec9816d|c24577/f2p18/1511     ATGGCCTGCGCAGGGTGCTGGATGAGCTGACCCTGGCCAGAGCCGACCTGGAGATGCAGA    687
NM_000422.2                              ATGGCCTGCGCAGGGTGCTGGATGAGCTGACCCTGGCCAGAGCCGACCTGGAGATGCAGA    743
i1_HQ_samplec9816d|c41456/f18p0/1500     GATGGAGGAGACAGGTCCCAGCTCTGGAGATTGTCATCTGA-----------TGGGAAGA    696
i1_HQ_samplec9816d|c64385/f12p0/1166     GATGGAGGAGACAGGTCCCAGCTCTGGAGATTGTCATCTGA-----------TGGGAAGA    360
                                            *   * *   *** *  *  *  *  **   *   * **              * ***

i1_HQ_samplec9816d|c24577/f2p18/1511     TTGAGAACCTCAAGGAGGAGCTGGCCTACCTGAAGAAGAACCACGAGGAGGAGATGAACG    747
NM_000422.2                              TTGAGAACCTCAAGGAGGAGCTGGCCTACCTGAAGAAGAACCACGAGGAGGAGATGAACG    803
i1_HQ_samplec9816d|c41456/f18p0/1500     TAGGAACATGGTCTCATGATCTTTGCTCTTGACAGCTTTTGGATGAGCAAAAACAGTCCT    756
i1_HQ_samplec9816d|c64385/f12p0/1166     TAGGAACATGGTCTCATGATCTTTGCTCTTGACAGCTTTTGGATGAGCAAAAACAGTCCT    420
                                         * *  *         * ** **   **      **       * *** *  *   *  *

i1_HQ_samplec9816d|c24577/f2p18/1511     CCCTGCGAGGCCAGGTGGGTGGTGAGATCAATGTGGAGATGGACGCTGCCCCAGGCGTGG    807
NM_000422.2                              CCCTGCGAGGCCAGGTGGGTGGTGAGATCAATGTGGAGATGGACGCTGCCCCAGGCGTGG    863
i1_HQ_samplec9816d|c41456/f18p0/1500     GTCTCTGGGGTCTGTAGCCTGATGGGAG--ATAGGGACATGGTCCTTGTCCTCCAAATTC    814
i1_HQ_samplec9816d|c64385/f12p0/1166     GTCTCTGGGGTCTGTAGCCTGATGGGAG--ATAGGGACATGGTCCTTGTCCTCCAAATTC    478
                                           **  * ** *  *  *  ** ** **   **  *** **** *  ** **      *

i1_HQ_samplec9816d|c24577/f2p18/1511     ACCTGAGCCGCATCCTCAACGAGATGCGTGACCAGTATGAGAAGATGGCAGAGAAGAACC    867
NM_000422.2                              ACCTGAGCCGCATCCTCAACGAGATGCGTGACCAGTATGAGAAGATGGCAGAGAAGAACC    923
i1_HQ_samplec9816d|c41456/f18p0/1500     CAATCTGATGG-------AGAATATATGATCCTAGCCTTAGGG---TTCCTAGTCTAAT-    863
i1_HQ_samplec9816d|c64385/f12p0/1166     CAATCTGATGG-------AGAATATATGATCCTAGCCTTAGGG---TTCCTAGTCTAAT-    527
                                            *  *  *        *  * **  *   * **  * **       *  **   **

i1_HQ_samplec9816d|c24577/f2p18/1511     GCAAGGATGCCGAGGATTGGTTCTTCAGCAAGACAGAG-GAACTGAACCGCGAGGTGGCC    926
NM_000422.2                              GCAAGGATGCCGAGGATTGGTTCTTCAGCAAGACAGAG-GAACTGAACCGCGAGGTGGCC    982
i1_HQ_samplec9816d|c41456/f18p0/1500     -GGAGGAGATAGGGGCCTGGTTTTTGTCTTGGCGATCCCAGTCTGATGGGGGAGATGGTA    922
i1_HQ_samplec9816d|c64385/f12p0/1166     -GGAGGAGATAGGGGCCTGGTTTTTGTCTTGGCGATCCCAGTCTGATGGGGGAGATGGTA    586
                                            ****    * **  ***** **      *  *       ****   * *** ***

i1_HQ_samplec9816d|c24577/f2p18/1511     ACCAACAGTGAGCTGGTGCAGAGTGGCAAGAGTGAGATCTCGGAGCTCCGGCGCACCATG    986
NM_000422.2                              ACCAACAGTGAGCTGGTGCAGAGTGGCAAGAGTGAGATCTCGGAGCTCCGGCGCACCATG    1042
i1_HQ_samplec9816d|c41456/f18p0/1500     GA--AAATCTATGTCCTAGAGACTGCAGAGAAATGGAGA-CGGATTTCATGG--AGTCTA    977
i1_HQ_samplec9816d|c64385/f12p0/1166     GA--AAATCTATGTCCTAGAGACTGCAGAGAAATGGAGA-CGGATTTCATGG--AGTCTA    641
                                             * *   *  *  *  *** **   ***    **   ****  **  *   *   *

i1_HQ_samplec9816d|c24577/f2p18/1511     CAGGCCTTGGAGATAGAGCTGCAGTCCCAGCTCAGCATGAAAGCATCCCTGGAGGGCAAC    1046
NM_000422.2                              CAGGCCTTGGAGATAGAGCTGCAGTCCCAGCTCAGCATGAAAGCATCCCTGGAGGGCAAC    1102
i1_HQ_samplec9816d|c41456/f18p0/1500     CATGGCTCCTCCCTGG-CAGGACACACTGGATCAGAATCAAATAACCCATCTGGGGAGGC    1036
i1_HQ_samplec9816d|c64385/f12p0/1166     CATGGCTCCTCCCTGG-CAGGACACACTGGATCAGAATCAAATAACCCATCTGGGGAGGC    700
                                         ** * **      * *    *     *  * **** ** ***  * ** *   ***   *

i1_HQ_samplec9816d|c24577/f2p18/1511     CTGGCGGAGACAGAGAACCGCTACTGCGTGCAGCTGTCCCAGATCCAGGGGCTG-ATTGG    1105
NM_000422.2                              CTGGCGGAGACAGAGAACCGCTACTGCGTGCAGCTGTCCCAGATCCAGGGGCTG-ATTGG    1161
i1_HQ_samplec9816d|c41456/f18p0/1500     AAGACTCACACAGGGC--------CACCGGCAGAGGGATGGGATGGAAGGGAGGTGGTGG    1088
i1_HQ_samplec9816d|c64385/f12p0/1166     AAGACTCACACAGGGC--------CACCGGCAGAGGGATGGGATGGAAGGGAGGTGGTGG    752
                                           * *  * **** *           *  ****  *     ***  * ***  *   ***

i1_HQ_samplec9816d|c24577/f2p18/1511     CAGCGTGGAGGAGCAGCTGGCCCAGCTTCGCTG--CGAGATGGAGCAGC----AGAACCA    1159
NM_000422.2                              CAGCGTGGAGGAGCAGCTGGCCCAGCTTCGCTG--CGAGATGGAGCAGC----AGAACCA    1215
i1_HQ_samplec9816d|c41456/f18p0/1500     CAGGG-------ACAGGAGGGATGTGTGTGCAGTGTGATGTCGAGGTGCCAGTGGAGGCA    1141
i1_HQ_samplec9816d|c64385/f12p0/1166     CAGGG-------ACAGGAGGGATGTGTGTGCAGTGTGATGTCGAGGTGCCAGTGGAGGCA    805
                                         *** *        ***  **      *  ** *   **  * ***  **     **  **

i1_HQ_samplec9816d|c24577/f2p18/1511     GGAATACAAAATCCTGCTGGATGTGA---AGACGCGGCTGGAGC-----AGGAGATTGCC    1211
NM_000422.2                              GGAATACAAAATCCTGCTGGATGTGA---AGACGCGGCTGGAGC-----AGGAGATTGCC    1267
i1_HQ_samplec9816d|c41456/f18p0/1500     CTCACAGCACCTGGGGGAGGATGAGGGAGAGAGCCGGCTCCTGTCCATGAGGGCTAGGGG    1201
i1_HQ_samplec9816d|c64385/f12p0/1166     CTCACAGCACCTGGGGGAGGATGAGGGAGAGAGCCGGCTCCTGTCCATGAGGGCTAGGGG    865
                                            * *  *  *   *  ***** *    ***  *****   *      ***     *

i1_HQ_samplec9816d|c24577/f2p18/1511     ACCTACCGCCGCCTGCTGGAGGGAGAGGATGCCCACCTGACTCAGTACAAGAAAGAACCG    1271
NM_000422.2                              ACCTACCGCCGCCTGCTGGAGGGAGAGGATGCCCACCTGACTCAGTACAAGAAAGAACCG    1327
i1_HQ_samplec9816d|c41456/f18p0/1500     GCAAGCGAGGGCCTCCTGGCCC---CTACCCACTTTAAATTGCCTGCTTCTCCTGCAGCG    1258
i1_HQ_samplec9816d|c64385/f12p0/1166     GCAAGCGAGGGCCTCCTGGCCC---CTACCCACTTTAAATTGCCTGCTTCTCCTGCAGCG    922
                                          *   *    **** ****             *         *           * * **

i1_HQ_samplec9816d|c24577/f2p18/1511     GTGACCACCCGTCAGGTGCGTACCATTGTGGAAGAGGTCCAGGATGGCAAGGTCATCTCC    1331
NM_000422.2                              GTGACCACCCGTCAGGTGCGTACCATTGTGGAAGAGGTCCAGGATGGCAAGGTCATCTCC    1387
i1_HQ_samplec9816d|c41456/f18p0/1500     GTGACCACCCGTCAGGTGCGTACCATTGTGGAAGAGGTCCAGGATGGCAAGGTCATCTCC    1318
i1_HQ_samplec9816d|c64385/f12p0/1166     GTGACCACCCGTCAGGTGCGTACCATTGTGGAAGAGGTCCAGGATGGCAAGGTCATCTCC    982
                                         ************************************************************

i1_HQ_samplec9816d|c24577/f2p18/1511     TCCCGCGAGCAGGTCCACCAGACCACCCGCTGAGGACTCAGCTACCCCGGCCGGCCACCC    1391
NM_000422.2                              TCCCGCGAGCAGGTCCACCAGACCACCCGCTGAGGACTCAGCTACCCCGGCCGGCCACCC    1447
i1_HQ_samplec9816d|c41456/f18p0/1500     TCCCGCGAGCAGGTCCACCAGACCACCCGCTGAGGACTCAGCTACCCCGGCCGGCCACCC    1378
i1_HQ_samplec9816d|c64385/f12p0/1166     TCCCGCGAGCAGGTCCACCAGACCACCCGCTGAGGACTCAGCTACCCCGGCCGGCCACCC    1042
                                         ************************************************************
```

```
i1_HQ_samplec9816d|c24577/f2p18/1511      AGGAGGCAGGGAGGCAGCCGCCCCATCTGCCCCACAGTCTCCGGCCTCTCCAGCCTCAGC    1451
NM_000422.2                               AGGAGGCAGGGAGGCAGCCGCCCCATCTGCCCCACAGTCTCCGGCCTCTCCAGCCTCAGC    1507
i1_HQ_samplec9816d|c41456/f18p0/1500      AGGAGGCAGGGAGGCAGCCGCCCCATCTGCCCCACAGTCTCCGGCCTCTCCAGCCTCAGC    1438
i1_HQ_samplec9816d|c64385/f12p0/1166      AGGAGGCAGGGAGGCAGCCGCCCCATCTGCCCCACAGTCTCCGGCCTCTCCAGCCTCAGC    1102
                                          ************************************************************

i1 HQ samplec9816d|c24577/f2p18/1511      CCCCTGCTTCAGTCCCTTCCCCATGCTTCCTTGCCTGATgacaataaagcttgt--tgac    1509
NM_000422.2                               CCCCTGCTTCAGTCCCTTCCCCATGCTTCCTTGCCTGATGACAATAAAGCTTGT--TGAC    1565
i1_HQ_samplec9816d|c41456/f18p0/1500      CCCCTGCTTCAGTCCCTTCCCCATGCTTCCTTGCCTGATGACAATAaagc--ttgttgac    1496
i1_HQ_samplec9816d|c64385/f12p0/1166      CCCCTGCTTCAGTCCCTTCCCCATGCTTCCTTGCCTGATGGACAATTAAAGCTTGTTGac    1162
                                          ****************************************   *   *     *  ****

i1_HQ_samplec9816d|c24577/f2p18/1511      tc-------         1511
NM_000422.2                               TCAGCTATG         1574
i1_HQ_samplec9816d|c41456/f18p0/1500      tcag-----         1500
i1_HQ_samplec9816d|c64385/f12p0/1166      tcag-----         1166
                                          **
```

```
VIRT65337        ----------------------------------MAPPWQ-DTLDQNQITHLGRQDSHR     24
NP_000413.1      MTTSIRQFTSSSSIKGSSGLGGGSSRTSCRLSGGLGAGSCRLGSAGGLGSTLGGSSYSSC     60
VIRT63810        ------------------------------------------------------------     0
VIRT66411        MTTSIRQFTSSSSIKGSSGLGGGSSRTSCRLSGGLGAGSCRLGSAGGLGSTLGGSSYSSC     60

VIRT65337        ---ATGRGMGWKGGGG--RDRRDV------------------------------------     43
NP_000413.1      YSFGSGGGYGSSFGGVDGLLAGGEKATMQNLNDRLASYLDKVRALEEANTELEVKIRDWY     120
VIRT63810        ------------------------------------------------------------     0
VIRT66411        YSFGSGGGYGSSFGGVDGLLAGGEKATMQNLNDRLASYLDKVRALEEANTELEVKIRDWY     120

VIRT65337        ------------------------------------------------------------     43
NP_000413.1      QRQAPGPARDYSQYYRTIEELQNKILTATVDNANILLQIDNARLAADDFRTKFETEQALR     180
VIRT63810        ------------------------------------------------------------     0
VIRT66411        QRQAPGPARDYSQYYRTIEELQNKILTATVDNANILLQIDNARLAADDFRTKFETEQALR     180

VIRT65337        ------------------------------------------------------------     43
NP_000413.1      LSVEADINGLRRVLDELTLARADLEMQIENLKEELAYLKKNHEEEMNALRGQVGGEINVE     240
VIRT63810        ------------------------------------------------------------     0
VIRT66411        LSVEADINGLRRVLDELTLARADLEMQIENLKEELAYLKKNHEEEMNALRGQVGGEINVE     240

VIRT65337        ------------------------------------------------------------     43
NP_000413.1      MDAAPGVDLSRILNEMRDQYEKMAEKNRKDAEDWFFSKTEELNREVATNSELVQSGKSEI     300
VIRT63810        ------------------------------------------------------------     0
VIRT66411        MDAAPGVDLSRILNEMRDQYEKMAEKNRKDAEDWFFSKTEELNREVATNSELVQSGKSEI     300

VIRT65337        ------------------------------------------------------------     43
NP_000413.1      SELRRTMQALEIELQSQLSMKASLEGNLAETENRYCVQLSQIQGLIGSVEEQLAQLRCEM     360
VIRT63810        ------MQALEIELQSQLSMKASLEGNLAETENRYCVQLSQIQGLIGSVEEQLAQLRCEM     54
VIRT66411        SELRRTMQALEIELQSQLSMKASLEGNLAETENRYCVQLSQIQGLIGSVEEQLAQLRCEM     360

VIRT65337        ------------------------------------------------------------     43
NP_000413.1      EQQNQEYKILLDVKTRLEQEIATYRRLLEGEDAHLTQYKKEPVTTRQVRTIVEEVQDGKV     420
VIRT63810        EQQNQEYKILLDVKTRLEQEIATYRRLLEGEDAHLTQYKKERKYLRGFGPGGWVHGTGSP     114
VIRT66411        EQQNQEYKILLDVKTRLEQEIATYRRLLEGEDAHLTQYKKEPVTTRQVRTIVEEVQDGKV     420

VIRT65337        ---CAV--------    46
NP_000413.1      ISSREQVHQTTR--    432
VIRT63810        PARCWGRVPRSSRS    128
VIRT66411        ISSREQVHQTTR--    432
```

## SPAG7

| Sequence | Header | AA Seq |
|---|---|---|
| **Present** | i1_HQ_samplec9816d\|c12681/f2p0/1109 | |
| | | |
| **Absent** | i0_HQ_samplec9816d\|c625/f16p11/997 | |
| **NCBI** | NM_004890 | |

>i1_HQ_samplec9816d|c12681/f2p0/1109
isoform=c12681;full_length_coverage=2;non_full_length_coverage=0;isoform_length=1109

actcccaagatggcggacctactgggctccatcctgagctccatggagaagccacccagcCTCGGTGACCAGGAGACTCGGCGCAAGGCCC
GAGAACAGGCCGCCCGCCTGAAGAAACTACAAGAGCAAGAGAAACAACAGAAAGTGGAGTTTCGTAAAAGGATGGA
GAAGGAGGTGTCAGATTTCATTCAAGACAGTGGGCAGATCAAGAAAAAGTTTCAGCCAATGAACAAGATCGAGAGGA

GCATACTACATGATGTGGTGGAAGTGGCTGGCCTGACATCCTTCTCCTTTGGGGAAGATGATGACTGTCGCTATGTCAT
GATCTTCAAAAAGGAGTTTGCACCCTCAGATGAAGAGCTAGACTCTTACCGTCGTGGAGAGGAATGGGACCCCCAGAA
GGCTGAGGAGAAGCGGAAGCTGAAGGAGCTGGCCCAGAGGCAAGAGGAGGAGGCAGCCCAGCAGGGGCCTGTGGT
GGTGAGCCCTGCCAGCGACTACAAGGACAAGTACAGCCACCTCATCGGCAAGGGAGCAGCCAAAGACGCAGCCCACA
TGCTACAGGCCAATAAGACCTACGGCTGTGGTGAGGCCACAGTGCGGCTGGGGGTGGCTGGGAGAGGAGCATAGGAT
GTGGCAGGAGGGCAGTGGAGGCTGAGGTACGGATTTCTAGGCCCGCCTACCCTCCTCTCTGCCCCTAGTGCCCGTGGC
CAATAAGAGGGACACACGCTCCATTGAAGAGGCTATGAATGAGATCAGAGCCAAGAAGCGTCTGCGGCAGAGTGGGG
AAGAGTTGCCGCCAACCTCCTAGGCGCCCCGCCCAGCTCCCTTTGACCCCTGGGGCAGGGCAGGGGGCAGGGAGAGA
CAAGGCTGCTGCTATTAGAGCCCATCCTGGAGCCCCACCTCTGAACCACCTCCTACCAGCTGTCCCTCAGGCTGGGGGA
AAACAGGTGTTTGATTTGTCACCGTTGGAGCTTGGATATGTGCGTGGCATGTGTGTGTGTGTGTGAGAGTGTGAATGC
ACAGGTGGGTATTTAATCTGTATTATTCCCCGTTCTTGGAATTTTCTTCCCCATGGGGctggggtactttacattcaataaatactgt
tt

>VIRT83091
MADLLGSILSSMEKPPSLGDQETRRKAREQAARLKKLQEQEKQQKVEFRKRMEKEVSDFI
QDSGQIKKKFQPMNKIERSILHDVVEVAGLTSFSFGEDDDCRYVMIFKKEFAPSDEELDS
YRRGEEWDPQKAEEKRKLKELAQRQEEEAAQQGPVVVSPASDYKDKYSHLIGKGAAKDAA
HMLQANKTYGCGEATVRLGVAGRGA
>i0_HQ_samplec9816d|c625/f16p11/997
isoform=c625;full_length_coverage=16;non_full_length_coverage=11;isoform_length=997

ACTCCCAAGATGGCGGACCTACTGGGCTCCATCCTGAGCTCCATGGAGAAGCCACCCAGCCTCGGTGACCAGGAGACT
CGGCGCAAGGCCCGAGAACAGGCCGCCCGCCTGAAGAAACTACAAGAGCAAGAGAAACAACAGAAAGTGGAGTTTCG
TAAAAGGATGGAGAAGGAGGTGTCAGATTTCATTCAAGACAGTGGGCAGATCAAGAAAAAGTTTCAGCCAATGAACA
AGATCGAGAGGAGCATACTACATGATGTGGTGGAAGTGGCTGGCCTGACATCCTTCTCCTTTGGGGAAGATGATGACT
GTCGCTATGTCATGATCTTCAAAAAGGAGTTTGCACCCTCAGATGAAGAGCTAGACTCTTACCGTCGTGGAGAGGAATG
GGACCCCCAGAAGGCTGAGGAGAAGCGGAAGCTGAAGGAGCTGGCCCAGAGGCAAGAGGAGGAGGCAGCCCAGCA
GGGGCCTGTGGTGGTGAGCCCTGCCAGCGACTACAAGGACAAGTACAGCCACCTCATCGGCAAGGGAGCAGCCAAAG
ACGCAGCCCACATGCTACAGGCCAATAAGACCTACGGCTGTGTGCCCGTGGCCAATAAGAGGGACACACGCTCCATTG
AAGAGGCTATGAATGAGATCAGAGCCAAGAAGCGTCTGCGGCAGAGTGGGGAAGAGTTGCCGCCAACCTCCTAGGCG
CCCCGCCCAGCTCCCTTTGACCCCTGGGGCAGGGCAGGGGGCAGGGAGAGACAAGGCTGCTGCTATTAGAGCCCATCC
TGGAGCCCCACCTCTGAACCACCTCCTACCAGCTGTCCCTCAGGCTGGGGGAAAACAGGTGTTTGATTTGTCACCGTTG
GAGCTTGGATATGTGCGTGGCATGTGTGTGTGTGTGTGAGAGTGTGAATGCACAGGTGGGTATTTAATCTGTATTATTC
CCCGTTCTTGGAATTTTCTTCCCCATGGGGCTGGGGTACTTTACATTCAATAAATACTGTTTAAC

>VIRT85203
MADLLGSILSSMEKPPSLGDQETRRKAREQAARLKKLQEQEKQQKVEFRKRMEKEVSDFI
QDSGQIKKKFQPMNKIERSILHDVVEVAGLTSFSFGEDDDCRYVMIFKKEFAPSDEELDS
YRRGEEWDPQKAEEKRKLKELAQRQEEEAAQQGPVVVSPASDYKDKYSHLIGKGAAKDAA
HMLQANKTYGCVPVANKRDTRSIEEAMNEIRAKKRLRQSGEELPPTS

>NM_004890.2 Homo sapiens sperm associated antigen 7 (SPAG7), mRNA
AAGACGCACCGCCCCCTCTCAGTCACTCCCAAGATGGCGGACCTACTGGGCTCCATCCTGAGCTCCATGG
AGAAGCCACCCAGCCTCGGTGACCAGGAGACTCGGCGCAAGGCCCGAGAACAGGCCGCCCGCCTGAAGAA
ACTACAAGAGCAAGAGAAACAACAGAAAGTGGAGTTTCGTAAAAGGATGGAGAAGGAGGTGTCAGATTTC
ATTCAAGACAGTGGGCAGATCAAGAAAAAGTTTCAGCCAATGAACAAGATCGAGAGGAGCATACTACATG
ATGTGGTGGAAGTGGCTGGCCTGACATCCTTCTCCTTTGGGGAAGATGATGACTGTCGCTATGTCATGAT
CTTCAAAAAGGAGTTTGCACCCTCAGATGAAGAGCTAGACTCTTACCGTCGTGGAGAGGAATGGGACCCC
CAGAAGGCTGAGGAGAAGCGGAAGCTGAAGGAGCTGGCCCAGAGGCAAGAGGAGGAGGCAGCCCAGCAGG
GGCCTGTGGTGGTGAGCCCTGCCAGCGACTACAAGGACAAGTACAGCCACCTCATCGGCAAGGGAGCAGC
CAAAGACGCAGCCCACATGCTACAGGCCAATAAGACCTACGGCTGTGTGCCCGTGGCCAATAAGAGGGAC
ACACGCTCCATTGAAGAGGCTATGAATGAGATCAGAGCCAAGAAGCGTCTGCGGCAGAGTGGGGAAGAGT

```
TGCCGCCAACCTCCTAGGCGCCCCGCCCAGCTCCCTTTGACCCCTGGGGCAGGGCAGGGGGCAGGGAGAG
ACAAGGCTGCTGCTATTAGAGCCCATCCTGGAGCCCCACCTCTGAACCACCTCCTACCAGCTGTCCCTCA
GGCTGGGGGAAAACAGGTGTTTGATTTGTCACCGTTGGAGCTTGGATATGTGCGTGGCATGTGTGTGTGT
GTGTGAGAGTGTGAATGCACAGGTGGGTATTTAATCTGTATTATTCCCCGTTCTTGGAATTTTCTTCCCC
ATGGGGCTGGGGTACTTTACATTCAATAAATACTGTTTAACCCAAAAAAAAAAAAAAA
>NP_004881.2 sperm-associated antigen 7 [Homo sapiens]
MADLLGSILSSMEKPPSLGDQETRRKAREQAARLKKLQEQEKQQKVEFRKRMEKEVSDFIQDSGQIKKKF
QPMNKIERSILHDVVEVAGLTSFSFGEDDDCRYVMIFKKEFAPSDEELDSYRRGEEWDPQKAEEKRKLKE
LAQRQEEEAAQQGPVVVSPASDYKDKYSHLIGKGAAKDAAHMLQANKTYGCVPVANKRDTRSIEEAMNEI
RAKKRLRQSGEELPPTS
i1_HQ_samplec9816d|c12681/f2p0/1109      ------------------------actcccaagatggcggacctactgggctccatcctg     36
i0_HQ_samplec9816d|c625/f16p11/997       ------------------------ACTCCCAAGATGGCGGACCTACTGGGCTCCATCCTG     36
NM_004890.2                              AAGACGCACCGCCCCCTCTCAGTCACTCCCAAGATGGCGGACCTACTGGGCTCCATCCTG     60
                                                                 ************************************

i1_HQ_samplec9816d|c12681/f2p0/1109      agctccatggagaagccacccagcCTCGGTGACCAGGAGACTCGGCGCAAGGCCCGAGAA     96
i0_HQ_samplec9816d|c625/f16p11/997       AGCTCCATGGAGAAGCCACCCAGCCTCGGTGACCAGGAGACTCGGCGCAAGGCCCGAGAA     96
NM_004890.2                              AGCTCCATGGAGAAGCCACCCAGCCTCGGTGACCAGGAGACTCGGCGCAAGGCCCGAGAA     120
                                         ************************************************************

i1_HQ_samplec9816d|c12681/f2p0/1109      CAGGCCGCCCGCCTGAAGAAACTACAAGAGCAAGAGAAACAACAGAAAGTGGAGTTTCGT     156
i0_HQ_samplec9816d|c625/f16p11/997       CAGGCCGCCCGCCTGAAGAAACTACAAGAGCAAGAGAAACAACAGAAAGTGGAGTTTCGT     156
NM_004890.2                              CAGGCCGCCCGCCTGAAGAAACTACAAGAGCAAGAGAAACAACAGAAAGTGGAGTTTCGT     180
                                         ************************************************************

i1_HQ_samplec9816d|c12681/f2p0/1109      AAAAGGATGGAGAAGGAGGTGTCAGATTTCATTCAAGACAGTGGGCAGATCAAGAAAAAG     216
i0_HQ_samplec9816d|c625/f16p11/997       AAAAGGATGGAGAAGGAGGTGTCAGATTTCATTCAAGACAGTGGGCAGATCAAGAAAAAG     216
NM_004890.2                              AAAAGGATGGAGAAGGAGGTGTCAGATTTCATTCAAGACAGTGGGCAGATCAAGAAAAAG     240
                                         ************************************************************

i1_HQ_samplec9816d|c12681/f2p0/1109      TTTCAGCCAATGAACAAGATCGAGAGGAGCATACTACATGATGTGGTGGAAGTGGCTGGC     276
i0_HQ_samplec9816d|c625/f16p11/997       TTTCAGCCAATGAACAAGATCGAGAGGAGCATACTACATGATGTGGTGGAAGTGGCTGGC     276
NM_004890.2                              TTTCAGCCAATGAACAAGATCGAGAGGAGCATACTACATGATGTGGTGGAAGTGGCTGGC     300
                                         ************************************************************

i1_HQ_samplec9816d|c12681/f2p0/1109      CTGACATCCTTCTCCTTTGGGGAAGATGATGACTGTCGCTATGTCATGATCTTCAAAAAG     336
i0_HQ_samplec9816d|c625/f16p11/997       CTGACATCCTTCTCCTTTGGGGAAGATGATGACTGTCGCTATGTCATGATCTTCAAAAAG     336
NM_004890.2                              CTGACATCCTTCTCCTTTGGGGAAGATGATGACTGTCGCTATGTCATGATCTTCAAAAAG     360
                                         ************************************************************

i1_HQ_samplec9816d|c12681/f2p0/1109      GAGTTTGCACCCTCAGATGAAGAGCTAGACTCTTACCGTCGTGGAGAGGAATGGGACCCC     396
i0_HQ_samplec9816d|c625/f16p11/997       GAGTTTGCACCCTCAGATGAAGAGCTAGACTCTTACCGTCGTGGAGAGGAATGGGACCCC     396
NM_004890.2                              GAGTTTGCACCCTCAGATGAAGAGCTAGACTCTTACCGTCGTGGAGAGGAATGGGACCCC     420
                                         ************************************************************

i1_HQ_samplec9816d|c12681/f2p0/1109      CAGAAGGCTGAGGAGAAGCGGAAGCTGAAGGAGCTGGCCCAGAGGCAAGAGGAGGAGGCA     456
i0_HQ_samplec9816d|c625/f16p11/997       CAGAAGGCTGAGGAGAAGCGGAAGCTGAAGGAGCTGGCCCAGAGGCAAGAGGAGGAGGCA     456
NM_004890.2                              CAGAAGGCTGAGGAGAAGCGGAAGCTGAAGGAGCTGGCCCAGAGGCAAGAGGAGGAGGCA     480
                                         ************************************************************

i1_HQ_samplec9816d|c12681/f2p0/1109      GCCCAGCAGGGGCCTGTGGTGGTGAGCCCTGCCAGCGACTACAAGGACAAGTACAGCCAC     516
i0_HQ_samplec9816d|c625/f16p11/997       GCCCAGCAGGGGCCTGTGGTGGTGAGCCCTGCCAGCGACTACAAGGACAAGTACAGCCAC     516
NM_004890.2                              GCCCAGCAGGGGCCTGTGGTGGTGAGCCCTGCCAGCGACTACAAGGACAAGTACAGCCAC     540
                                         ************************************************************

i1_HQ_samplec9816d|c12681/f2p0/1109      CTCATCGGCAAGGGAGCAGCCAAAGACGCAGCCCACATGCTACAGGCCAATAAGACCTAC     576
i0_HQ_samplec9816d|c625/f16p11/997       CTCATCGGCAAGGGAGCAGCCAAAGACGCAGCCCACATGCTACAGGCCAATAAGACCTAC     576
NM_004890.2                              CTCATCGGCAAGGGAGCAGCCAAAGACGCAGCCCACATGCTACAGGCCAATAAGACCTAC     600
                                         ************************************************************

i1_HQ_samplec9816d|c12681/f2p0/1109      GGCTGTGGTGAGGCCACAGTGCGGCTGGGGGTGGCTGGGAGAGGAGCATAGGATGTGGCA     636
i0_HQ_samplec9816d|c625/f16p11/997       GGCTGT------------------------------------------------------     582
NM_004890.2                              GGCTGT------------------------------------------------------     606
                                         ******

i1_HQ_samplec9816d|c12681/f2p0/1109      GGAGGGCAGTGGAGGCTGAGGTACGGATTTCTAGGCCCGCCTACCCTCCTCTCTGCCCCT     696
i0_HQ_samplec9816d|c625/f16p11/997       ------------------------------------------------------------     582
NM_004890.2                              ------------------------------------------------------------     606


i1_HQ_samplec9816d|c12681/f2p0/1109      AGTGCCCGTGGCCAATAAGAGGGACACACGCTCCATTGAAGAGGCTATGAATGAGATCAG     756
i0_HQ_samplec9816d|c625/f16p11/997       -GTGCCCGTGGCCAATAAGAGGGACACACGCTCCATTGAAGAGGCTATGAATGAGATCAG     641
NM_004890.2                              -GTGCCCGTGGCCAATAAGAGGGACACACGCTCCATTGAAGAGGCTATGAATGAGATCAG     665
                                          ***********************************************************

i1_HQ_samplec9816d|c12681/f2p0/1109      AGCCAAGAAGCGTCTGCGGCAGAGTGGGGAAGAGTTGCCGCCAACCTCCTAGGCGCCCCG     816
i0_HQ_samplec9816d|c625/f16p11/997       AGCCAAGAAGCGTCTGCGGCAGAGTGGGGAAGAGTTGCCGCCAACCTCCTAGGCGCCCCG     701
NM_004890.2                              AGCCAAGAAGCGTCTGCGGCAGAGTGGGGAAGAGTTGCCGCCAACCTCCTAGGCGCCCCG     725
                                         ************************************************************

i1_HQ_samplec9816d|c12681/f2p0/1109      CCCAGCTCCCTTTGACCCCTGGGGCAGGGCAGGGGGCAGGGAGAGACAAGGCTGCTGCTA     876
i0_HQ_samplec9816d|c625/f16p11/997       CCCAGCTCCCTTTGACCCCTGGGGCAGGGCAGGGGGCAGGGAGAGACAAGGCTGCTGCTA     761
NM_004890.2                              CCCAGCTCCCTTTGACCCCTGGGGCAGGGCAGGGGGCAGGGAGAGACAAGGCTGCTGCTA     785
                                         ************************************************************

i1_HQ_samplec9816d|c12681/f2p0/1109      TTAGAGCCCATCCTGGAGCCCCACCTCTGAACCACCTCCTACCAGCTGTCCCTCAGGCTG     936
i0_HQ_samplec9816d|c625/f16p11/997       TTAGAGCCCATCCTGGAGCCCCACCTCTGAACCACCTCCTACCAGCTGTCCCTCAGGCTG     821
NM_004890.2                              TTAGAGCCCATCCTGGAGCCCCACCTCTGAACCACCTCCTACCAGCTGTCCCTCAGGCTG     845
                                         ************************************************************

i1_HQ_samplec9816d|c12681/f2p0/1109      GGGGAAAACAGGTGTTTGATTTGTCACCGTTGGAGCTTGGATATGTGCGTGGCATGTGTG     996
i0_HQ_samplec9816d|c625/f16p11/997       GGGGAAAACAGGTGTTTGATTTGTCACCGTTGGAGCTTGGATATGTGCGTGGCATGTGTG     881
NM_004890.2                              GGGGAAAACAGGTGTTTGATTTGTCACCGTTGGAGCTTGGATATGTGCGTGGCATGTGTG     905
```

```
                                        ************************************************************

i1_HQ_samplec9816d|c12681/f2p0/1109     TGTGTGTGTGAGAGTGTGAATGCACAGGTGGGTATTTAATCTGTATTATTCCCCGTTCTT    1056
i0_HQ_samplec9816d|c625/f16p11/997      TGTGTGTGTGAGAGTGTGAATGCACAGGTGGGTATTTAATCTGTATTATTCCCCGTTCTT    941
NM_004890.2                             TGTGTGTGTGAGAGTGTGAATGCACAGGTGGGTATTTAATCTGTATTATTCCCCGTTCTT    965
                                        ************************************************************

i1_HQ_samplec9816d|c12681/f2p0/1109     GGAATTTTCTTCCCCATGGGGctggggtactttacattcaataaatactgttt-------    1109
i0_HQ_samplec9816d|c625/f16p11/997      GGAATTTTCTTCCCCATGGGGCTGGGGTACTTTACATTCAATAAATACTGTTTAAC----    997
NM_004890.2                             GGAATTTTCTTCCCCATGGGGCTGGGGTACTTTACATTCAATAAATACTGTTTAACCCAA    1025
                                        *****************************************************

i1_HQ_samplec9816d|c12681/f2p0/1109     -------------    1109
i0_HQ_samplec9816d|c625/f16p11/997      -------------    997
NM_004890.2                             AAAAAAAAAAAAA    1038
```

```
VIRT83091       MADLLGSILSSMEKPPSLGDQETRRKAREQAARLKKLQEQEKQQKVEFRKRMEKEVSDFI    60
VIRT85203       MADLLGSILSSMEKPPSLGDQETRRKAREQAARLKKLQEQEKQQKVEFRKRMEKEVSDFI    60
NP_004881.2     MADLLGSILSSMEKPPSLGDQETRRKAREQAARLKKLQEQEKQQKVEFRKRMEKEVSDFI    60
                ************************************************************

VIRT83091       QDSGQIKKKFQPMNKIERSILHDVVEVAGLTSFSFGEDDDCRYVMIFKKEFAPSDEELDS    120
VIRT85203       QDSGQIKKKFQPMNKIERSILHDVVEVAGLTSFSFGEDDDCRYVMIFKKEFAPSDEELDS    120
NP_004881.2     QDSGQIKKKFQPMNKIERSILHDVVEVAGLTSFSFGEDDDCRYVMIFKKEFAPSDEELDS    120
                ************************************************************

VIRT83091       YRRGEEWDPQKAEEKRKLKELAQRQEEEAAQQGPVVVSPASDYKDKYSHLIGKGAAKDAA    180
VIRT85203       YRRGEEWDPQKAEEKRKLKELAQRQEEEAAQQGPVVVSPASDYKDKYSHLIGKGAAKDAA    180
NP_004881.2     YRRGEEWDPQKAEEKRKLKELAQRQEEEAAQQGPVVVSPASDYKDKYSHLIGKGAAKDAA    180
                ************************************************************

VIRT83091       HMLQANKTYGCGEATVRLGVAGRGA----------------------    205
VIRT85203       HMLQANKTYGCVPVANKRDTRSIEEAMNEIRAKKRLRQSGEELPPTS    227
NP_004881.2     HMLQANKTYGCVPVANKRDTRSIEEAMNEIRAKKRLRQSGEELPPTS    227
                ***********  .: :  .. .
```

## RPS11

| Sequence | Header | AA Seq |
|---|---|---|
| **Present** | i1_HQ_samplec9816d\|c27462/f2p0/1054 | |
| | | |
| **Absent** | i0_HQ_samplec9816d\|c92306/f20p24/550 | |
| **NCBI** | NM_001015 | |

>i1_HQ_samplec9816d|c27462/f2p0/1054

isoform=c27462;full_length_coverage=2;non_full_length_coverage=0;isoform_length=1054

gcggccgggaagatggcggacattcagactgagcgtgcctaccaaaagcagCCGACCATCTTTTCAAAACAAGAAGAGGGTCCTGCTGG
GAGAAACTGGCAAGGAGAAGCTCCCGCGGTACTACAAGAACATCGGTCTGGGCTTCAAGACACCCAAGGAGGTGCGG
GGAACCTCAGAAGAAAGAAGGGGAACCTGGCGTTCCTGCACGTGTGCCCACGACGAGTTGCCCTGCCTGCATCTAAGT
GGCTTCTGGGGCTGCTGGGAATTGTAGTTGCTTCCCTGAGGCCACGCCCCTGGCTCTTTTAAGGAACCGCCCGCCCAAG
GCTCACTCCTTTATCTTTCCTATCCTTTCAGGCTATTGAGGGCACCTACATTGACAAGAAATGCCCCTTCACTGGTAATGT
GTCCATTCGAGGGCGGATCCTCTCTGGTAAGTGCGGGAGTTACTGGTGTCTGGGGCCTGAAATACTGAAAGAAGGGTC
TTGGGGCCCAGACTCCTGGGTCCTGGGTGAGAGGGGTGGTTAGGAATGCAAACTTGTAGGTCCAGGTACATTGGCAG
ATGATGTTTGTTTTCACGATGGTCTTCAGATGCCCACGTGGGCACTGCTGAGAAAGCCACTTGGTAAAACTGATGCCGG
AAATGGGGCTTTTTGGGATCCCTGCTCAGCTGCTTCTGAGTCCCAGCATGCCCTGGGTTACCTATGGCCCTCTTTCCCAT
GGGACCTGACCTATGATCGGCCCCCGCTCCTAGGCGTGGTGACCAAGATGAAGATGCAGAGGACCATTGTCATCCGCC
GAGACTATCTGCACTACATCCGCAAGTACAACCGCTTCGAGAAGCGCCACAAGAACATGTCTGTACACCTGTCCCCCTG
CTTCAGGGACGTCCAGATCGGTGACATCGTCACAGTGGGCGAGTGCCGGCCTCTGAGCAAGACAGTGCGCTTCAACGT
GCTCAAGGTCACCAAGGCTGCCGGCACCAAGAAGCAGTTCCAGAAGTTCTGAGGCTGGACATCGGccgctccccacaatgaa
ataagttattttctcattc

```
>VIRT95885
MGKRAIGNPGHAGTQKQLSRDPKKPHFRHQFYQVAFSAVPTWASEDHRENKHHLPMYLDL
QVCIPNHPSHPGPRSLGPKTLLSVFQAPDTSNSRTYQRGSALEWTHYQ
```

>i0_HQ_samplec9816d|c92306/f20p24/550
isoform=c92306;full_length_coverage=20;non_full_length_coverage=24;isoform_length=550

CTTTTTTTCAGGCGCCGGGAAGATGGCGGACATTCAGACTGAGCGTGCCTACCAAAAGCAGCCGACCATCTTTCAAAAC
AAGAAGAGGGTCCTGCTGGGAGAAACTGGCAAGGAGAAGCTCCCGCGGTACTACAAGAACATCGGTCTGGGCTTCAA
GACACCCAAGGAGGCTATTGAGGGCACCTACATTGACAAGAAATGCCCCTTCACTGGTAATGTGTCCATTCGAGGGCG
GATCCTCTCTGGCGTGGTGACCAAGATGAAGATGCAGAGGACCATTGTCATCCGCCGAGACTATCTGCACTACATCCGC
AAGTACAACCGCTTCGAGAAGCGCCACAAGAACATGTCTGTACACCTGTCCCCCTGCTTCAGGGACGTCCAGATCGGTG
ACATCGTCACAGTGGGCGAGTGCCGGCCTCTGAGCAAGACAGTGCGCTTCAACGTGCTCAAGGTCACCAAGGCTGCCG
GCACCAAGAAGCAGTTCCAGAAGTTCTGAGGCTGGACATCGGCCCGCTCCCCACAATGAAATAAAGTTATTTTCTCATT
C

```
>VIRT97525
MTMVLCIFILVTTPERIRPRMDTLPVKGHFLSM
```

```
>NM_001015.4 Homo sapiens ribosomal protein S11 (RPS11), mRNA
TCTCCTTACGTCACTTCCTCTCCAGCCCCTGCGTAATCGATAAGGAAACCCGGACGCTGCTGCCCCTTTC
TTTTTTTCAGGCGGCCGGGAAGATGGCGGACATTCAGACTGAGCGTGCCTACCAAAAGCAGCCGACCATC
TTTCAAAACAAGAAGAGGGTCCTGCTGGGAGAAACTGGCAAGGAGAAGCTCCCGCGGTACTACAAGAACA
TCGGTCTGGGCTTCAAGACACCCAAGGAGGCTATTGAGGGCACCTACATTGACAAGAAATGCCCCTTCAC
TGGTAATGTGTCCATTCGAGGGCGGATCCTCTCTGGCGTGGTGACCAAGATGAAGATGCAGAGGACCATT
GTCATCCGCCGAGACTATCTGCACTACATCCGCAAGTACAACCGCTTCGAGAAGCGCCACAAGAACATGT
CTGTACACCTGTCCCCCTGCTTCAGGGACGTCCAGATCGGTGACATCGTCACAGTGGGCGAGTGCCGGCC
TCTGAGCAAGACAGTGCGCTTCAACGTGCTCAAGGTCACCAAGGCTGCCGGCACCAAGAAGCAGTTCCAG
AAGTTCTGAGGCTGGACATCGGCCCGCTCCCCACAATGAAATAAAGTTATTTTCTCATTCCCAGGCCAGA
CTTGGGATCTTCCGCG
```

```
>NP_001006.1 40S ribosomal protein S11 [Homo sapiens]
MADIQTERAYQKQPTIFQNKKRVLLGETGKEKLPRYYKNIGLGFKTPKEAIEGTYIDKKCPFTGNVSIRG
RILSGVVTKMKMQRTIVIRRDYLHYIRKYNRFEKRHKNMSVHLSPCFRDVQIGDIVTVGECRPLSKTVRF
NVLKVTKAAGTKKQFQKF
```

```
i1_HQ_samplec9816d|c27462/f2p0/1054       gcggccgggaagatggcggacattcagactgagcgtgcctaccaaaagcagCCGACCATC    60
i0_HQ_samplec9816d|c92306/f20p24/550      ------------------------------------------------------------    0
NM_001015.4                               ------------------------------------------------------------    0

i1_HQ_samplec9816d|c27462/f2p0/1054       TTTTCAAAACAAGAAGAGGGTCCTGCTGGGAGAAACTGGCAAGGAGAAGCTCCCGCGGTA    120
i0_HQ_samplec9816d|c92306/f20p24/550      ------------------------------------------------------------    0
NM_001015.4                               ------------------------------------------------------------    0

i1_HQ_samplec9816d|c27462/f2p0/1054       CTACAAGAACATCGGTCTGGGCTTCAAGACACCCAAGGAGGTGCGGGGAACCTCAGAAGA    180
i0_HQ_samplec9816d|c92306/f20p24/550      ------------------------------------------------------------    0
NM_001015.4                               ------------------------------------------------------------    0

i1_HQ_samplec9816d|c27462/f2p0/1054       AAGAAGGGGAACCTGGCGTTCCTGCACGTGTGCCCACGACGAGTTGCCCTGCCTGCATCT    240
i0_HQ_samplec9816d|c92306/f20p24/550      ------------------------------------------------------------    0
NM_001015.4                               ------------------------------------------------------------    0

i1_HQ_samplec9816d|c27462/f2p0/1054       AAGTGGCTTCTGGGGCTGCTGGGAATTGTAGTTGCTTCCCTGAGGCCACGCCCCTGGCTC    300
i0_HQ_samplec9816d|c92306/f20p24/550      ------------------------------------------------------------    0
NM_001015.4                               ------------------------------------------------------------    0

i1_HQ_samplec9816d|c27462/f2p0/1054       TTTTAAGGAACCGCCCGCCCAAGGCTCACTCCTTTATCTTTCCTATCCTTTCAGGCTATT    360
i0_HQ_samplec9816d|c92306/f20p24/550      ------------------------------------------------------------    0
NM_001015.4                               ------------------------------------------------------------    0

i1_HQ_samplec9816d|c27462/f2p0/1054       GAGGGCACCTACATTGACAAGAAATGCCCCTTCACTGGTAATGTGTCCATTCGAGGGCGG    420
i0_HQ_samplec9816d|c92306/f20p24/550      ------------------------------------------------------------    0
NM_001015.4                               -----------------------------------TCTCCTTACGT-----------CAC    14

i1_HQ_samplec9816d|c27462/f2p0/1054       ATCCTCTCTGGTAAGTGCGGGAGTTACTGGTGTCTGGGGCCTGAAATACTGAAAGAAGGG    480
i0_HQ_samplec9816d|c92306/f20p24/550      ------------------------------------------------------------    0
NM_001015.4                               TTCCTCTCCAGCCCCTG-------------------CGTAAT-CGATAAGGAAACCCGGA    54

i1_HQ_samplec9816d|c27462/f2p0/1054       TCTTGGGGCCCAGACTCCTGGGTCCTGGGTGAGAGGGGTGGTTAGGAATGCAACTTG-T    539
```

```
i0_HQ_samplec9816d|c92306/f20p24/550      ----------------CTTTTTTTCAGGCGCCGGGAAGATGGCGGACATTCAGACTGAGC    44
NM_001015.4                               CGCTGCTGCCCCTTTCTTTTTTTCAGGCGGCCGGGAAGATGGCGGACATTCAGACTGAGC    114
                                                          *   *   *     * *  *  *   *  ** ** ***

i1_HQ_samplec9816d|c27462/f2p0/1054       AGGTCCAGGTACATTGGCAGATGATGTTTGTTTTCACGATGG---TCTTCAGATGCCCAC    596
i0_HQ_samplec9816d|c92306/f20p24/550      GTGCCTACCAAAAGCAGCCGACCATCTTTCAAAACAAGAAGAGGGTCCTGCTGGGAGAAA    104
NM_001015.4                               GTGCCTACCAAAAGCAGCCGACCATCTTTCAAAACAAGAAGAGGGTCCTGCTGGGAGAAA    174
                                            * * *    * *    ** **   ** ***      ** ** *     ** *      *   *

i1_HQ_samplec9816d|c27462/f2p0/1054       GTGGGCACTGCTGAGAAAGCCACTTGGTAAAACTGATGCCGGAAATGGGGCTTTTTGGGA    656
i0_HQ_samplec9816d|c92306/f20p24/550      CTGG-CAA---GGAGAAGCTCCCGCGGTACTACAAGAACATCGGTCTGGGC-TTCAAGAC    159
NM_001015.4                               CTGG-CAA---GGAGAAGCTCCCGCGGTACTACAAGAACATCGGTCTGGGC-TTCAAGAC    229
                                           *** **     *****   * *  ****  **      *         **** **   *

i1_HQ_samplec9816d|c27462/f2p0/1054       TCCCTGCTCAGCTGCTTCTGAGTCCCAGCATGCCCTGGGTTACCTATGGCCCTCTTTCCC    716
i0_HQ_samplec9816d|c92306/f20p24/550      ACCCAAGGAGGCTA-TTGAG--------GGCACCTACATTGACAAGAAATGCCCCTTCAC    210
NM_001015.4                               ACCCAAGGAGGCTA-TTGAG--------GGCACCTACATTGACAAGAAATGCCCCTTCAC    280
                                           ***       ***  **  *             **      * **         * * *** *

i1_HQ_samplec9816d|c27462/f2p0/1054       ATGGGACCTGACCTATGATCGGCCCCCGCTCCTAGGCGTGGTGACCAAGATGAAGATGCA    776
i0_HQ_samplec9816d|c92306/f20p24/550      TGGTAATGTGTCCATTCGAGGGCGGATCCTCTCTGGCGTGGTGACCAAGATGAAGATGCA    270
NM_001015.4                               TGGTAATGTGTCCATTCGAGGGCGGATCCTCTCTGGCGTGGTGACCAAGATGAAGATGCA    340
                                            *  *  ** **  *    ***     ***   *************************

i1_HQ_samplec9816d|c27462/f2p0/1054       GAGGACCATTGTCATCCGCCGAGACTATCTGCACTACATCCGCAAGTACAACCGCTTCGA    836
i0_HQ_samplec9816d|c92306/f20p24/550      GAGGACCATTGTCATCCGCCGAGACTATCTGCACTACATCCGCAAGTACAACCGCTTCGA    330
NM_001015.4                               GAGGACCATTGTCATCCGCCGAGACTATCTGCACTACATCCGCAAGTACAACCGCTTCGA    400
                                          ************************************************************

i1_HQ_samplec9816d|c27462/f2p0/1054       GAAGCGCCACAAGAACATGTCTGTACACCTGTCCCCCTGCTTCAGGGACGTCCAGATCGG    896
i0_HQ_samplec9816d|c92306/f20p24/550      GAAGCGCCACAAGAACATGTCTGTACACCTGTCCCCCTGCTTCAGGGACGTCCAGATCGG    390
NM_001015.4                               GAAGCGCCACAAGAACATGTCTGTACACCTGTCCCCCTGCTTCAGGGACGTCCAGATCGG    460
                                          ************************************************************

i1_HQ_samplec9816d|c27462/f2p0/1054       TGACATCGTCACAGTGGGCGAGTGCCGGCCTCTGAGCAAGACAGTGCGCTTCAACGTGCT    956
i0_HQ_samplec9816d|c92306/f20p24/550      TGACATCGTCACAGTGGGCGAGTGCCGGCCTCTGAGCAAGACAGTGCGCTTCAACGTGCT    450
NM_001015.4                               TGACATCGTCACAGTGGGCGAGTGCCGGCCTCTGAGCAAGACAGTGCGCTTCAACGTGCT    520
                                          ************************************************************

i1_HQ_samplec9816d|c27462/f2p0/1054       CAAGGTCACCAAGGCTGCCGGCACCAAGAAGCAGTTCCAGAAGTTCTGAGGCTGGACATC    1016
i0_HQ_samplec9816d|c92306/f20p24/550      CAAGGTCACCAAGGCTGCCGGCACCAAGAAGCAGTTCCAGAAGTTCTGAGGCTGGACATC    510
NM_001015.4                               CAAGGTCACCAAGGCTGCCGGCACCAAGAAGCAGTTCCAGAAGTTCTGAGGCTGGACATC    580
                                          ************************************************************

i1_HQ_samplec9816d|c27462/f2p0/1054       GGccgctccccacaatgaaa-ta-agttattttctcattc--------------------    1054
i0_HQ_samplec9816d|c92306/f20p24/550      GGCCCGCTCCCCACAATGAAATAAAGTTATTTTCTCATTC--------------------    550
NM_001015.4                               GGCCCGCTCCCCACAATGAAATAAAGTTATTTTCTCATTCCCAGGCCAGACTTGGGATCT    640
                                          ****     ***    *    ** ** ****************

i1_HQ_samplec9816d|c27462/f2p0/1054       ------            1054
i0_HQ_samplec9816d|c92306/f20p24/550      ------            550
NM_001015.4                               TCCGCG            646
```

```
VIRT95885        ------------------MGKRAIGNPGHAGTQK----------------QLSRDPKK     24
VIRT97525        MTMV---------LCIF-----------------------ILVTTP---------ERIR     18
NP_001006.1      MADIQTERAYQKQPTIFQNKKRVLLGETGKEKLPRYYKNIGLGFKTPKEAIEGTYIDKKC     60
                                                                          :

VIRT95885        PH-----FRHQFYQ------------VAFSAVPTWASEDHRENKHHLPMYLDLQVCIPNH     67
VIRT97525        PRMDTLPVKGHFLSM---------------------------------------------     33
NP_001006.1      PFTGNVSIRGRILSGVVTKMKMQRTIVIRRDYLHYIRKYNRFEKRHKNMSVHLSPCFRDV     120
                 *       .: :: .

VIRT95885        PSH-----PGPRSLGPKTLLSVFQAPDTSNSRTYQRGSALEWTHYQ     108
VIRT97525        ----------------------------------------------     33
NP_001006.1      QIGDIVTVGECRPLSKTVRFNVLKVTKA-------AGTKKQFQKF-     158
```

## NAA10

| Sequence | Header | AA Seq |
|---|---|---|
| **Present** | i1_HQ_samplec9816d\|c63491/f3p24/1053 | |
| | | |
| **Absent** | i0_HQ_samplec9816d\|c92336/f66p39/908 | |
| **NCBI** | NM_001256119 | |
| | NM_003491 | |

>i1_HQ_samplec9816d|c63491/f3p24/1053
isoform=c63491;full_length_coverage=3;non_full_length_coverage=24;isoform_length=1053

gagtgtgcctgcggccgctggacctatggggctggcccagcccGCCGAAGAGGCCGGGGCGCGGGGCCGGGGCTGCGATTCCCTCCT
GTTTGCGTGATGTCGGGCCTCCGTTGGGTGGGCTCCGGGGACCTCCGGGGCGCCCACTCCTGTTCCTGTGCTCCGGGC
GTTGTGCAAAGTCAGATCGTCACAGTGCCTGCCCAGCCCCGAGGGAGGGGGGCCGAGCAGACCGACGGGGAGCAGG

CTTCTCACCCGTGGTCACCGCCGCCTTAGACTGAGCGCGTTCCACTGCCCTCCTTCCCTGCAGCCAGAGGACCTAATGAA
CATGCAGCACTGCAACCTCCTCTGCCTGCCCGAGAACTACCAGATGAAATACTACTTCTACCATGGCCTTTCCTGGCCCC
AGCTCTCTTACATTGCTGAGGACGAGAATGGGAAGATTGTGGGTATGTCCTGGCCAAAATGGAAGAGGACCCAGATG
ATGTGCCCCATGGACATATCACCTCATTGGCTGTGAAGCGTTCCCACCGGCGCCTCGGTCTGGCTCAGAAACTGATGGA
CCAGGCCTCTCGAGCCATGATAGAGAACTTCAATGCCAAATATGTCTCCCTGCATGTCAGGAAGAGTAACCGGGCCGCC
CTGCACCTCTATTCCAACACCCTCAACTTTCAGATCAGTGAAGTGGAGCCCAAATACTATGCAGATGGGGAGGACGCCT
ATGCCATGAAGCGGGACCTCACTCAGATGGCCGACGAGCTGAGGCGGCACCTGGAGCTGAAAGAGAAGGGCAGGCA
CGTGGTGCTGGGTGCCATCGAGAACAAGGTGGAGAGCAAAGGCAATTCACCTCCGAGCTCAGGAGAGGCCTGTCGCG
AGGAGAAGGGCCTGGCTGCCGAGGATAGTGGTGGGGACAGCAAGGACCTCAGCGAGGTCAGCGAGACCACAGAGAG
CACAGATGTCAAGGACAGCTCAGAGGCCTCCGACTCAGCCTCCTAGAGCCTGCCCCATCCCCTCCTCACCCCAcgagctttc
acaataaatttgctccgtggcactgg

>VIRT111589
MNMQHCNLLCLPENYQMKYYFYHGLSWPQLSYIAEDENGKIVGYVLAKMEEDPDDVPHGH
ITSLAVKRSHRRLGLAQKLMDQASRAMIENFNAKYVSLHVRKSNRAALHLYSNTLNFQIS
EVEPKYYADGEDAYAMKRDLTQMADELRRHLELKEKGRHVVLGAIENKVESKGNSPPSSG
EACREEKGLAAEDSGGDSKDLSEVSETTESTDVKDSSEASDSAS

>i0_HQ_samplec9816d|c92336/f66p39/908
isoform=c92336;full_length_coverage=66;non_full_length_coverage=39;isoform_length=908

gcccggcgcgcaccgccccttccGCCGTCGCCCAGCGAGCCCAGCTCCGGTCCCAGCCCCGGCCGTCCCGGCGTCGCTTCGGAG
CGCGGCGGCAGCTGACTGCGCCTTCACGATCCGCTGGGACCCGCGAGCCCCGCCGCCGTTATGAACATCCGCAATGCG
AGGCCAGAGGACCTAATGAACATGCAGCACTGCAACCTCCTCTGCCTGCCCGAGAACTACCAGATGAAATACTACTTCT
ACCATGGCCTTTCCTGGCCCCAGCTCTCTTACATTGCTGAGGACGAGAATGGGAAGATTGTGGGTATGTCCTGGCCAA
AATGGAAGAGGACCCAGATGATGTGCCCCATGGACATATCACCTCATTGGCTGTGAAGCGTTCCCACCGGCGCCTCGGT
CTGGCTCAGAAACTGATGGACCAGGCCTCTCGAGCCATGATAGAGAACTTCAATGCCAAATATGTCTCCCTGCATGTCA
GGAAGAGTAACCGGGCCGCCCTGCACCTCTATTCCAACACCCTCAACTTTCAGATCAGTGAAGTGGAGCCCAAATACTA
TGCAGATGGGGAGGACGCCTATGCCATGAAGCGGGACCTCACTCAGATGGCCGACGAGCTGAGGCGGCACCTGGAGC
TGAAAGAGAAGGGCAGGCACGTGGTGCTGGGTGCCATCGAGAACAAGGTGGAGAGCAAAGGCAATTCACCTCCGAG
CTCAGGAGAGGCCTGTCGCGAGGAGAAGGGCCTGGCTGCCGAGGATAGTGGTGGGGACAGCAAGGACCTCAGCGAG
GTCAGCGAGACCACAGAGAGCACAGATGTCAAGGACAGCTCAGAGGCCTCCGACTCAGCCTCCTAGAGCCTGCCCCAT
CCCCTCCTCACCCCACGAGCTTTCACAATAAATTTGCTCCGTGG

>VIRT112564

MNIRNARPEDLMNMQHCNLLCLPENYQMKYYFYHGLSWPQLSYIAEDENGKIVGYVLAKM
EEDPDDVPHGHITSLAVKRSHRRLGLAQKLMDQASRAMIENFNAKYVSLHVRKSNRAALH
LYSNTLNFQISEVEPKYYADGEDAYAMKRDLTQMADELRRHLELKEKGRHVVLGAIENKV
ESKGNSPPSSGEACREEKGLAAEDSGGDSKDLSEVSETTESTDVKDSSEASDSAS

>NM_001256119.1 Homo sapiens N(alpha)-acetyltransferase 10, NatA catalytic subunit (NAA10), transcript variant 2, mRNA
GACCGGCTCCGCGCATTGGCCCGCCCCGTGCGCTGGCCTGTCCCGCGCATTGGCCAAGCGGCCGGGAGGA
CGGGCCCGGCGGCCGTGCACTTCCGGCCGGTGGCCGGCCCGGCGCGCACCGCCCCTTCCGCCGTCGCCCA
GCGAGCCCAGCTCCGGTCCCAGCCCCGGCCGTCCCGGCGTCGCTTCGGAGCGCGGCGGCAGCTGACTGCG
CCTTCACGATCCGCTGGGACCCGCGAGCCCCGCCGCCGTTATGAACATCCGCAATGCGAGGCCAGAGGAC
CTAATGAACATGCAGCACTGCAACCTCCTCTGCCTGCCCGAGAACTACCAGATGAAATACTACTTCTACC
ATGGCCTTTCCTGGCCCCAGCTCTCTTACATTGCTGAGGACGAGAATGGGAAGATTGTGGGTATGTCCT
GGCCAAAATGGAAGAGGACCCAGATGATGTGCCCCATGGACATATCACCTCATTGGCTGTGAAGCGTTCC
CACCGGCGCCTCGGTCTGGCTCAGAAACTGATGGACCAGGCCTCTCGAGCCATGATAGAGAACTTCAATG
CCAAATATGTCTCCCTGCATGTCAGGAAGAGGATCAGTGAAGTGGAGCCCAAATACTATGCAGATGGGGA

```
GGACGCCTATGCCATGAAGCGGGACCTCACTCAGATGGCCGACGAGCTGAGGCGGCACCTGGAGCTGAAA
GAGAAGGGCAGGCACGTGGTGCTGGGTGCCATCGAGAACAAGGTGGAGAGCAAAGGCAATTCACCTCCGA
GCTCAGGAGAGGCCTGTCGCGAGGAGAAGGGCCTGGCTGCCGAGGATAGTGGTGGGGACAGCAAGGACCT
CAGCGAGGTCAGCGAGACCACAGAGAGCACAGATGTCAAGGACAGCTCAGAGGCCTCCGACTCAGCCTCC
TAGAGCCTGCCCCATCCCCTCCTCACCCCACGAGCTTTCACAATAAATTCGCTCCGTGGCACTGGGGAAC
TTTGTGTGTGAGCGCGCGCACATTTAGAGGGTGTGTTTCTCCAGGTCCTCTGGTGGGGATGTGAGCCTTG
GCCTTTTGACCCAGAGCATCCTGAAAAAAAAAAAAAAAAAA
>NP_001243048.1 N-alpha-acetyltransferase 10 isoform 2 [Homo sapiens]
MNIRNARPEDLMNMQHCNLLCLPENYQMKYYFYHGLSWPQLSYIAEDENGKIVGYVLAKMEEDPDDVPHG
HITSLAVKRSHRRLGLAQKLMDQASRAMIENFNAKYVSLHVRKRISEVEPKYYADGEDAYAMKRDLTQMA
DELRRHLELKEKGRHVVLGAIENKVESKGNSPPSSGEACREEKGLAAEDSGGDSKDLSEVSETTESTDVK
DSSEASDSAS
>NM_003491.3 Homo sapiens N(alpha)-acetyltransferase 10, NatA catalytic subunit (NAA10), transcript variant
1, mRNA
GACCGGCTCCGCGCATTGGCCCGCCCCGTGCGCTGGCCTGTCCCGCGCATTGGCCAAGCGGCCGGGAGGA
CGGGCCCGGCGGCCGTGCACTTCCGGCCGGTGGCCGGCCCGGCGCGCACCGCCCCTTCCGCCGTCGCCCA
GCGAGCCCAGCTCCGGTCCCAGCCCCGGCCGTCCCGGCGTCGCTTCGGAGCGCGGCGGCAGCTGACTGCG
CCTTCACGATCCGCTGGGACCCGCGAGCCCCGCCGCCGTTATGAACATCCGCAATGCGAGGCCAGAGGAC
CTAATGAACATGCAGCACTGCAACCTCCTCTGCCTGCCCGAGAACTACCAGATGAAATACTACTTCTACC
ATGGCCTTTCCTGGCCCCAGCTCTCTTACATTGCTGAGGACGAGAATGGGAAGATTGTGGGGTATGTCCT
GGCCAAAATGGAAGAGGACCCAGATGATGTGCCCCATGGACATATCACCTCATTGGCTGTGAAGCGTTCC
CACCGGCGCCTCGGTCTGGCTCAGAAACTGATGGACCAGGCCTCTCGAGCCATGATAGAGAACTTCAATG
CCAAATATGTCTCCCTGCATGTCAGGAAGAGTAACCGGGCCGCCCTGCACCTCTATTCCAACACCCTCAA
CTTTCAGATCAGTGAAGTGGAGCCCAAATACTATGCAGATGGGGAGGACGCCTATGCCATGAAGCGGGAC
CTCACTCAGATGGCCGACGAGCTGAGGCGGCACCTGGAGCTGAAAGAGAAGGGCAGGCACGTGGTGCTGG
GTGCCATCGAGAACAAGGTGGAGAGCAAAGGCAATTCACCTCCGAGCTCAGGAGAGGCCTGTCGCGAGGA
GAAGGGCCTGGCTGCCGAGGATAGTGGTGGGGACAGCAAGGACCTCAGCGAGGTCAGCGAGACCACAGAG
AGCACAGATGTCAAGGACAGCTCAGAGGCCTCCGACTCAGCCTCCTAGAGCCTGCCCCATCCCCTCCTCA
CCCCACGAGCTTTCACAATAAATTCGCTCCGTGGCACTGGGGAACTTTGTGTGTGAGCGCGCGCACATTT
AGAGGGTGTGTTTCTCCAGGTCCTCTGGTGGGGATGTGAGCCTTGGCCTTTTGACCCAGAGCATCCTGAA
AAAAAAAAAAAAAAAA
>NP_003482.1 N-alpha-acetyltransferase 10 isoform 1 [Homo sapiens]
MNIRNARPEDLMNMQHCNLLCLPENYQMKYYFYHGLSWPQLSYIAEDENGKIVGYVLAKMEEDPDDVPHG
HITSLAVKRSHRRLGLAQKLMDQASRAMIENFNAKYVSLHVRKSNRAALHLYSNTLNFQISEVEPKYYAD
GEDAYAMKRDLTQMADELRRHLELKEKGRHVVLGAIENKVESKGNSPPSSGEACREEKGLAAEDSGGDSK
DLSEVSETTESTDVKDSSEASDSAS
```

```
i1_HQ_samplec9816d|c63491/f3p24/1053      gagtgtgcctgcggccgctggacctatggggctggcccagcccGCCGAAGAGGCCGGGGC    60
NM_001256119.1                            -----------------------------GACCGGCTCCGCGCATTGGCCCGCCCCGTGC    31
i0_HQ_samplec9816d|c92336/f66p39/908      ------------------------------------------------------------    0
NM_003491.3                               -----------------------------GACCGGCTCCGCGCATTGGCCCGCCCCGTGC    31

i1_HQ_samplec9816d|c63491/f3p24/1053      GCGGGGCCGGGGCTGCGATTCCCTCCTGTTTGCGTGATGTCGGGCCTCCGTTGGGTGGGC    120
NM_001256119.1                            GCTGGCCTGT-CCCGCGCATTGGCCAAGCGGCCGGGAGGACGGGCCCGGCGGCCGTGCAC    90
i0_HQ_samplec9816d|c92336/f66p39/908      ------------------------------------------------------------    0
NM_003491.3                               GCTGGCCTGT-CCCGCGCATTGGCCAAGCGGCCGGGAGGACGGGCCCGGCGGCCGTGCAC    90

i1_HQ_samplec9816d|c63491/f3p24/1053      -----TCCGGGGACCTCCGGGGCGCCCACTCCTGTTCCTGTGCTCCGGGCGTTGTGCAAA    175
NM_001256119.1                            TTCCGGCCGGTGGCCGGCCCGGCGCGCACCGCCCCTTCCGCCGT-----CGCCCAGCGAG    145
i0_HQ_samplec9816d|c92336/f66p39/908      ----------------gcccggcgcgcaccgccccttccGCCGT-----CGCCCAGCGAG    39
NM_003491.3                               TTCCGGCCGGTGGCCGGCCCGGCGCGCACCGCCCCTTCCGCCGT-----CGCCCAGCGAG    145
                                                           *  ***** ***  *   * * *   *     **    ** *

i1_HQ_samplec9816d|c63491/f3p24/1053      GTCAGATCGTCACAGTGCCTGCCCAGCCCCGAGGGAGGGGGGCCGAGCAGACCGACGGGG    235
NM_001256119.1                            CCCAGCTCCGGTCCCAGCCCCGGCCGTCCCGGCGTCGCTTC--GGAGCGCGGCGGCAGC-    202
i0_HQ_samplec9816d|c92336/f66p39/908      CCCAGCTCCGGTCCCAGCCCCGGCCGTCCCGGCGTCGCTTC--GGAGCGCGGCGGCAGC-    96
NM_003491.3                               CCCAGCTCCGGTCCCAGCCCCGGCCGTCCCGGCGTCGCTTC--GGAGCGCGGCGGCAGC-    202
                                            *** **    *   ***    * * ****  *  *       ****    ** * *

i1_HQ_samplec9816d|c63491/f3p24/1053      AGCAGGCTTCTCACCCGTGGTCACCGCCGCCTTAGACTGAGCGCGTTCCACTGCCCTCCT    295
NM_001256119.1                            TGACTGCGCCTTCACGATCCGCTGGGACCCGCGAGCCCCGCCGCCGTTATGAACATCCGC    262
i0_HQ_samplec9816d|c92336/f66p39/908      TGACTGCGCCTTCACGATCCGCTGGGACCCGCGAGCCCCGCCGCCGTTATGAACATCCGC    156
NM_003491.3                               TGACTGCGCCTTCACGATCCGCTGGGACCCGCGAGCCCCGCCGCCGTTATGAACATCCGC    262
                                           *   **  **   *  *   *   * * *   ** *    ***  *      *   *

i1_HQ_samplec9816d|c63491/f3p24/1053      TCCCTGCAGCCAGAGGACCTAATGAACATGCAGCACTGCAACCTCCTCTGCCTGCCCGAG    355
NM_001256119.1                            AATGCGAGGCCAGAGGACCTAATGAACATGCAGCACTGCAACCTCCTCTGCCTGCCCGAG    322
i0_HQ_samplec9816d|c92336/f66p39/908      AATGCGAGGCCAGAGGACCTAATGAACATGCAGCACTGCAACCTCCTCTGCCTGCCCGAG    216
NM_003491.3                               AATGCGAGGCCAGAGGACCTAATGAACATGCAGCACTGCAACCTCCTCTGCCTGCCCGAG    322
                                               *  ****************************************************

i1_HQ_samplec9816d|c63491/f3p24/1053      AACTACCAGATGAAATACTACTTCTACCATGGCCTTTCCTGGCCCCAGCTCTCTTACATT    415
NM_001256119.1                            AACTACCAGATGAAATACTACTTCTACCATGGCCTTTCCTGGCCCCAGCTCTCTTACATT    382
i0_HQ_samplec9816d|c92336/f66p39/908      AACTACCAGATGAAATACTACTTCTACCATGGCCTTTCCTGGCCCCAGCTCTCTTACATT    276
NM_003491.3                               AACTACCAGATGAAATACTACTTCTACCATGGCCTTTCCTGGCCCCAGCTCTCTTACATT    382
                                          ************************************************************

i1_HQ_samplec9816d|c63491/f3p24/1053      GCTGAGGACGAGAATGGGAAGATTGTGGGGTATGTCCTGGCCAAAATGGAAGAGGACCCA    475
NM_001256119.1                            GCTGAGGACGAGAATGGGAAGATTGTGGGGTATGTCCTGGCCAAAATGGAAGAGGACCCA    442
i0_HQ_samplec9816d|c92336/f66p39/908      GCTGAGGACGAGAATGGGAAGATTGTGGGGTATGTCCTGGCCAAAATGGAAGAGGACCCA    336
NM_003491.3                               GCTGAGGACGAGAATGGGAAGATTGTGGGGTATGTCCTGGCCAAAATGGAAGAGGACCCA    442
                                          ************************************************************

i1_HQ_samplec9816d|c63491/f3p24/1053      GATGATGTGCCCCATGGACATATCACCTCATTGGCTGTGAAGCGTTCCCACCGGCGCCTC    535
```

```
NM_001256119.1                          GATGATGTGCCCCATGGACATATCACCTCATTGGCTGTGAAGCGTTCCCACCGGCGCCTC    502
i0_HQ_samplec9816d|c92336/f66p39/908    GATGATGTGCCCCATGGACATATCACCTCATTGGCTGTGAAGCGTTCCCACCGGCGCCTC    396
NM_003491.3                             GATGATGTGCCCCATGGACATATCACCTCATTGGCTGTGAAGCGTTCCCACCGGCGCCTC    502
                                        ************************************************************

i1_HQ_samplec9816d|c63491/f3p24/1053    GGTCTGGCTCAGAAACTGATGGACCAGGCCTCTCGAGCCATGATAGAGAACTTCAATGCC    595
NM_001256119.1                          GGTCTGGCTCAGAAACTGATGGACCAGGCCTCTCGAGCCATGATAGAGAACTTCAATGCC    562
i0_HQ_samplec9816d|c92336/f66p39/908    GGTCTGGCTCAGAAACTGATGGACCAGGCCTCTCGAGCCATGATAGAGAACTTCAATGCC    456
NM_003491.3                             GGTCTGGCTCAGAAACTGATGGACCAGGCCTCTCGAGCCATGATAGAGAACTTCAATGCC    562
                                        ************************************************************

i1_HQ_samplec9816d|c63491/f3p24/1053    AAATATGTCTCCCTGCATGTCAGGAAGAGTAACCGGGCCGCCCTGCACCTCTATTCCAAC    655
NM_001256119.1                          AAATATGTCTCCCTGCATGTCAGGAAGAGG------------------------------    592
i0_HQ_samplec9816d|c92336/f66p39/908    AAATATGTCTCCCTGCATGTCAGGAAGAGTAACCGGGCCGCCCTGCACCTCTATTCCAAC    516
NM_003491.3                             AAATATGTCTCCCTGCATGTCAGGAAGAGTAACCGGGCCGCCCTGCACCTCTATTCCAAC    622
                                        *****************************

i1_HQ_samplec9816d|c63491/f3p24/1053    ACCCTCAACTTTCAGATCAGTGAAGTGGAGCCCAAATACTATGCAGATGGGGAGGACGCC    715
NM_001256119.1                          ---------------ATCAGTGAAGTGGAGCCCAAATACTATGCAGATGGGGAGGACGCC    637
i0_HQ_samplec9816d|c92336/f66p39/908    ACCCTCAACTTTCAGATCAGTGAAGTGGAGCCCAAATACTATGCAGATGGGGAGGACGCC    576
NM_003491.3                             ACCCTCAACTTTCAGATCAGTGAAGTGGAGCCCAAATACTATGCAGATGGGGAGGACGCC    682
                                                       *********************************************

i1_HQ_samplec9816d|c63491/f3p24/1053    TATGCCATGAAGCGGGACCTCACTCAGATGGCCGACGAGCTGAGGCGGCACCTGGAGCTG    775
NM_001256119.1                          TATGCCATGAAGCGGGACCTCACTCAGATGGCCGACGAGCTGAGGCGGCACCTGGAGCTG    697
i0_HQ_samplec9816d|c92336/f66p39/908    TATGCCATGAAGCGGGACCTCACTCAGATGGCCGACGAGCTGAGGCGGCACCTGGAGCTG    636
NM_003491.3                             TATGCCATGAAGCGGGACCTCACTCAGATGGCCGACGAGCTGAGGCGGCACCTGGAGCTG    742
                                        ************************************************************

i1_HQ_samplec9816d|c63491/f3p24/1053    AAAGAGAAGGGCAGGCACGTGGTGCTGGGTGCCATCGAGAACAAGGTGGAGAGCAAAGGC    835
NM_001256119.1                          AAAGAGAAGGGCAGGCACGTGGTGCTGGGTGCCATCGAGAACAAGGTGGAGAGCAAAGGC    757
i0_HQ_samplec9816d|c92336/f66p39/908    AAAGAGAAGGGCAGGCACGTGGTGCTGGGTGCCATCGAGAACAAGGTGGAGAGCAAAGGC    696
NM_003491.3                             AAAGAGAAGGGCAGGCACGTGGTGCTGGGTGCCATCGAGAACAAGGTGGAGAGCAAAGGC    802
                                        ************************************************************

i1_HQ_samplec9816d|c63491/f3p24/1053    AATTCACCTCCGAGCTCAGGAGAGGCCTGTCGCGAGGAGAAGGGCCTGGCTGCCGAGGAT    895
NM_001256119.1                          AATTCACCTCCGAGCTCAGGAGAGGCCTGTCGCGAGGAGAAGGGCCTGGCTGCCGAGGAT    817
i0_HQ_samplec9816d|c92336/f66p39/908    AATTCACCTCCGAGCTCAGGAGAGGCCTGTCGCGAGGAGAAGGGCCTGGCTGCCGAGGAT    756
NM_003491.3                             AATTCACCTCCGAGCTCAGGAGAGGCCTGTCGCGAGGAGAAGGGCCTGGCTGCCGAGGAT    862
                                        ************************************************************

i1_HQ_samplec9816d|c63491/f3p24/1053    AGTGGTGGGGACAGCAAGGACCTCAGCGAGGTCAGCGAGACCACAGAGAGCACAGATGTC    955
NM_001256119.1                          AGTGGTGGGGACAGCAAGGACCTCAGCGAGGTCAGCGAGACCACAGAGAGCACAGATGTC    877
i0_HQ_samplec9816d|c92336/f66p39/908    AGTGGTGGGGACAGCAAGGACCTCAGCGAGGTCAGCGAGACCACAGAGAGCACAGATGTC    816
NM_003491.3                             AGTGGTGGGGACAGCAAGGACCTCAGCGAGGTCAGCGAGACCACAGAGAGCACAGATGTC    922
                                        ************************************************************

i1_HQ_samplec9816d|c63491/f3p24/1053    AAGGACAGCTCAGAGGCCTCCGACTCAGCCTCCTAGAGCCTGCCCCATCCCCTCCTCACC    1015
NM_001256119.1                          AAGGACAGCTCAGAGGCCTCCGACTCAGCCTCCTAGAGCCTGCCCCATCCCCTCCTCACC    937
i0_HQ_samplec9816d|c92336/f66p39/908    AAGGACAGCTCAGAGGCCTCCGACTCAGCCTCCTAGAGCCTGCCCCATCCCCTCCTCACC    876
NM_003491.3                             AAGGACAGCTCAGAGGCCTCCGACTCAGCCTCCTAGAGCCTGCCCCATCCCCTCCTCACC    982
                                        ************************************************************

i1_HQ_samplec9816d|c63491/f3p24/1053    CCAcgagctttcacaataaatttgctccgtggcactgg----------------------    1053
NM_001256119.1                          CCACGAGCTTTCACAATAAATTCGCTCCGTGGCACTGGGGAACTTTGTGTGTGAGCGCGC    997
i0_HQ_samplec9816d|c92336/f66p39/908    CCACGAGCTTTCACAATAAATTTGCTCCGTGG----------------------------    908
NM_003491.3                             CCACGAGCTTTCACAATAAATTCGCTCCGTGGCACTGGGGAACTTTGTGTGTGAGCGCGC    1042
                                        ********************* *********

i1_HQ_samplec9816d|c63491/f3p24/1053    ------------------------------------------------------------    1053
NM_001256119.1                          GCACATTTAGAGGGTGTGTTTCTCCAGGTCCTCTGGTGGGGATGTGAGCCTTGGCCTTTT    1057
i0_HQ_samplec9816d|c92336/f66p39/908    ------------------------------------------------------------    908
NM_003491.3                             GCACATTTAGAGGGTGTGTTTCTCCAGGTCCTCTGGTGGGGATGTGAGCCTTGGCCTTTT    1102

i1_HQ_samplec9816d|c63491/f3p24/1053    ----------------------------------    1053
NM_001256119.1                          GACCCAGAGCATCCTGAAAAAAAAAAAAAAAAAA    1091
i0_HQ_samplec9816d|c92336/f66p39/908    ----------------------------------    908
NM_003491.3                             GACCCAGAGCATCCTGAAAAAAAAAAAAAAAAAA    1136
```

```
NP_001243048.1      MNIRNARPEDLMNMQHCNLLCLPENYQMKYYFYHGLSWPQLSYIAEDENGKIVGYVLAKM   60
VIRT111589          -----------MNMQHCNLLCLPENYQMKYYFYHGLSWPQLSYIAEDENGKIVGYVLAKM   49
VIRT112564          MNIRNARPEDLMNMQHCNLLCLPENYQMKYYFYHGLSWPQLSYIAEDENGKIVGYVLAKM   60
NP_003482.1         MNIRNARPEDLMNMQHCNLLCLPENYQMKYYFYHGLSWPQLSYIAEDENGKIVGYVLAKM   60
                               *************************************************

NP_001243048.1      EEDPDDVPHGHITSLAVKRSHRRLGLAQKLMDQASRAMIENFNAKYVSLHVRK-------   113
VIRT111589          EEDPDDVPHGHITSLAVKRSHRRLGLAQKLMDQASRAMIENFNAKYVSLHVRKSNRAALH   109
VIRT112564          EEDPDDVPHGHITSLAVKRSHRRLGLAQKLMDQASRAMIENFNAKYVSLHVRKSNRAALH   120
NP_003482.1         EEDPDDVPHGHITSLAVKRSHRRLGLAQKLMDQASRAMIENFNAKYVSLHVRKSNRAALH   120
                    *****************************************************

NP_001243048.1      --------RISEVEPKYYADGEDAYAMKRDLTQMADELRRHLELKEKGRHVVLGAIENKV   165
VIRT111589          LYSNTLNFQISEVEPKYYADGEDAYAMKRDLTQMADELRRHLELKEKGRHVVLGAIENKV   169
VIRT112564          LYSNTLNFQISEVEPKYYADGEDAYAMKRDLTQMADELRRHLELKEKGRHVVLGAIENKV   180
NP_003482.1         LYSNTLNFQISEVEPKYYADGEDAYAMKRDLTQMADELRRHLELKEKGRHVVLGAIENKV   180
                            :***************************************************

NP_001243048.1      ESKGNSPPSSGEACREEKGLAAEDSGGDSKDLSEVSETTESTDVKDSSEASDSAS        220
VIRT111589          ESKGNSPPSSGEACREEKGLAAEDSGGDSKDLSEVSETTESTDVKDSSEASDSAS        224
VIRT112564          ESKGNSPPSSGEACREEKGLAAEDSGGDSKDLSEVSETTESTDVKDSSEASDSAS        235
```

NP_003482.1 ESKGNSPPSSGEACREEKGLAAEDSGGDSKDLSEVSETTESTDVKDSSEASDSAS 235
*******************************************************

## EMC9

| Sequence | Header | AA Seq |
|---|---|---|
| **Present** | i0_HQ_samplec9816d\|c2150/f3p12/894 | |
| | i1_HQ_samplec9816d\|c6322/f2p3/1046 | |
| **Absent** | i0_HQ_samplec9816d\|c1265/f10p11/810 | |
| **NCBI** | NM_016049 | |
| | NM_001346877 | |
| | NM_001346874 | |

>i0_HQ_samplec9816d|c2150/f3p12/894
isoform=c2150;full_length_coverage=3;non_full_length_coverage=12;isoform_length=894

ggggcggactccgggacccagggcgccgggagccggcgggctacctgcgagtcgagttagcgtTGTCGCCGAACCGAAGCCTCGCTCGCCAT
GGGGGAGGTGGAGATCTCGGCCCTGGCCTACGTGAAGATGTGCCTGCATGCTGCCCGGTACCCACACGCCGCAGTCAA
CGGGCTGTTTTTGGCGCCAGCGCCGCGGTCTGGAGAATGCCTGTGCCTCACCGACTGTGTGCCCCTCTTCCACAGCCAC
CTGGCCCTGTCCGTCATGTTGGAGGTCGCCCTCAACCAGGTGGATGTGTGGGGAGCACAGGCCGGTCTGGTGGTGGCT
GGTTACTACCATGCCAATGCAGCTGTGAACGATCAGAGCACTCAAGCATCAAGCAGTCGTGCATCAAGCATCCAGCAG
GCCCGATCCCCGCAGGCCACCTACAGGGAAAGGCCACAGCTGCGCCCACACCTATGAGACCTGGGAGGCCCAGCTCAA
ATGGATGGGTTTTGCCCGCCCTGGGCCCCTGGCCTTGAAAATTGCTGGGCGAATTGCAGAATTCTTCCCTGATGCAGTA
CTTATTATGTTGGATAATCAGAAACTGGTGCCTCAGCCTCGTGTGCCCCCGGTCATCGTCCTGGAGAACCAAGGTCTCC
GCTGGGTCCCTAAGGATAAGAACTTAGTGATGTGGAGGGACTGGGAAGAGTCACGGCAGATGGTGGGAGCTCTACTG
GAAGATCGGGCCCACCAGCACCTTGTGGACTTTGACTGCCACCTTGATGACATCCGGCAGGACTGGACCAACCAGCGG
CTCAACACTCAAATCACCCAGTGGGTTGGTCCCACTAATGGAAATGGAAATGCCTGAGCCAGGGCCAGCGGGGCCCGG
TTCCAATAAAGAGACTTGGG

>VIRT130505
MGEVEISALAYVKMCLHAARYPHAAVNGLFLAPAPRSGECLCLTDCVPLFHSHLALSVML
EVALNQVDVWGAQAGLVVAGYYHANAAVNDQSTQASSSRASSIQQARSPQATYRERPQLR
PHL

>i1_HQ_samplec9816d|c6322/f2p3/1046
isoform=c6322;full_length_coverage=2;non_full_length_coverage=3;isoform_length=1046

gtccgggacccagggcgccgggagccggcgggctacctgcgagtcgagttagcgttgtcgCCGAACCGAAGTGAGAGGGCACGGGGAAGG
GTCAAAGAGCGTGCCGAAGCGCTGGAGGGAGCTTCACGGACGCGAGCTAGGCACCGGCTCGCCTAATCCGGTACTAA
TCCGGCTTGCTGCTTCCCGTCCAGGCCTCGCTCGCCATGGGGGAGGTGGAGATCTCGGCCCTGGCCTACGTGAAGATGT
GCCTGCATGCTGCCCGGTACCCACACGCCGCAGTCAACGGGCTGTTTTTGGCGCCAGCGCCGCGGTCTGGAGAATGCC
TGTGCCTCACCGACTGTGTGCCCCTCTTCCACAGCCACCTGGCCCTGTCCGTCATGTTGGAGGTCGCCCTCAACCAGGTG
GATGTGTGGGGAGCACAGGCCGGTCTGGTGGTGGCTGGTTACTACCATGCCAATGCAGCTGTGAACGATCAGAGCACT
CAAGCATCAAGCAGTCGTGCATCAAGCATCCAGCAGGCCCGATCCCCGCAGGCCACCTACAGGGAAAGGCCACAGCTG
CGCCCACACCTATGAGACCTGGGAGGCCCAGCTCAAATGGATGGGTTTTGCCCACAGTTGGCTCCTCACTCCTCTGATCT
TCCCTCTACACAGCCCTGGGCCCCTGGCCTTGAAAATTGCTGGGCGAATTGCAGAATTCTTCCCTGATGCAGTACTTATT
ATGTTGGATAATCAGAAACTGGTGCCTCAGCCTCGTGTGCCCCCGGTCATCGTCCTGGAGAACCAAGGTCTCCGCTGGG
TCCCTAAGGATAAGAACTTAGTGATGTGGAGGGACTGGGAAGAGTCACGGCAGATGGTGGGAGCTCTACTGGAAGAT
CGGGCCCACCAGCACCTTGTGGACTTTGACTGCCACCTTGATGACATCCGGCAGGACTGGACCAACCAGCGGCTCAACA
CTCAAATCACCCAGTGGGTTGGTCCCACTAATGGAAATGGAAATGCCTGAGCCAGggccagcggggcccggttccaataaagaga
cttggg

>VIRT147453
MGFAHSWLLTPLIFPLHSPGPLALKIAGRIAEFFPDAVLIMLDNQKLVPQPRVPPVIVLE
NQGLRWVPKDKNLVMWRDWEESRQMVGALLEDRAHQHLVDFDCHLDDIRQDWTNQRLNTQ
ITQWVGPTNGNGNA

>i0_HQ_samplec9816d|c1265/f10p11/810
isoform=c1265;full_length_coverage=10;non_full_length_coverage=11;isoform_length=810

CCGAACCGAAGTGAGAGGGCACGGGGAAGGGTCAAAGAGCGTGCCGAAGCGCTGGAGGGAGCTTCACGGACGCGA
GCTAGGCACCGGCTCGCCTAATCCGGTACTAATCCGGCTTGCTGCTTCCCGTCCAGGCCTCGCTCGCCATGGGGGAGGT
GGAGATCTCGGCCCTGGCCTACGTGAAGATGTGCCTGCATGCTGCCCGGTACCCACACGCCGCAGTCAACGGGCTGTTT
TTGGCGCCAGCGCCGCGGTCTGGAGAATGCCTGTGCCTCACCGACTGTGTGCCCCTCTTCCACAGCCACCTGGCCCTGT
CCGTCATGTTGGAGGTCGCCCTCAACCAGGTGGATGTGTGGGGAGCACAGGCCGGTCTGGTGGTGGCTGGTTACTACC
ATGCCAATGCAGCTGTGAACGATCAGAGCCCTGGGCCCCTGGCCTTGAAAATTGCTGGGCGAATTGCAGAATTCTTCCC
TGATGCAGTACTTATTATGTTGGATAATCAGAAACTGGTGCCTCAGCCTCGTGTGCCCCCGGTCATCGTCCTGGAGAAC
CAAGGTCTCCGCTGGGTCCCTAAGGATAAGAACTTAGTGATGTGGAGGGACTGGGAAGAGTCACGGCAGATGGTGGG
AGCTCTACTGGAAGATCGGGCCCACCAGCACCTTGTGGACTTTGACTGCCACCTTGATGACATCCGGCAGGACTGGACC
AACCAGCGGCTCAACACTCAAATCACCCAGTGGGTTGGTCCCACTAATGGAAATGGAAATGCCTGAGCCAGGGCCAGC
GGGGCCCGGTTCCAATAAAGAGACTTGG

>VIRT151133
MGEVEISALAYVKMCLHAARYPHAAVNGLFLAPAPRSGECLCLTDCVPLFHSHLALSVML
EVALNQVDVWGAQAGLVVAGYYHANAAVNDQSPGPLALKIAGRIAEFFPDAVLIMLDNQK
LVPQPRVPPVIVLENQGLRWVPKDKNLVMWRDWEESRQMVGALLEDRAHQHLVDFDCHLD
DIRQDWTNQRLNTQITQWVGPTNGNGNA

>NM_016049.3 Homo sapiens ER membrane protein complex subunit 9 (EMC9), transcript variant 1, mRNA
GTCGCGGCCGCCAGCTAGGGGCGCGGGAAGGCGGGGCTCGGATGCAATCGGGACCTCCTCCTGGACTGGG
CCGGGGGCGGACTCCGGGACCCAGGGCGCCGGGAGCCGGCGGGCTACCTGCGAGTCGAGTTAGCGTTGTC
GCCGAACCGAAGCCTCGCTCGCCATGGGGGAGGTGGAGATCTCGGCCCTGGCCTACGTGAAGATGTGCCT
GCATGCTGCCCGGTACCCACACGCCGCAGTCAACGGGCTGTTTTTGGCGCCAGCGCCGCGGTCTGGAGAA
TGCCTGTGCCTCACCGACTGTGTGCCCCTCTTCCACAGCCACCTGGCCCTGTCCGTCATGTTGGAGGTCG
CCCTCAACCAGGTGGATGTGTGGGGAGCACAGGCCGGTCTGGTGGTGGCTGGTTACTACCATGCCAATGC
AGCTGTGAACGATCAGAGCCCTGGGCCCCTGGCCTTGAAAATTGCTGGGCGAATTGCAGAATTCTTCCCT
GATGCAGTACTTATTATGTTGGATAATCAGAAACTGGTGCCTCAGCCTCGTGTGCCCCCGGTCATCGTCC
TGGAGAACCAAGGTCTCCGCTGGGTCCCTAAGGATAAGAACTTAGTGATGTGGAGGGACTGGGAAGAGTC
ACGGCAGATGGTGGGAGCTCTACTGGAAGATCGGGCCCACCAGCACCTTGTGGACTTTGACTGCCACCTT
GATGACATCCGGCAGGACTGGACCAACCAGCGGCTCAACACTCAAATCACCCAGTGGGTTGGTCCCACTA
ATGGAAATGGAAATGCCTGAGCCAGGGCCAGCGGGGCCCGGTTCCAATAAAGAGACTTGGGCTGAAAAAA
AAAAAAAAAAAAAAAAAAAAAAAAAAAAAAAAAAAAAAAAAAAAAAAAAAAAAAAAAAA
>NP_057133.2 ER membrane protein complex subunit 9 isoform a [Homo sapiens]
MGEVEISALAYVKMCLHAARYPHAAVNGLFLAPAPRSGECLCLTDCVPLFHSHLALSVMLEVALNQVDVW
GAQAGLVVAGYYHANAAVNDQSPGPLALKIAGRIAEFFPDAVLIMLDNQKLVPQPRVPPVIVLENQGLRW
VPKDKNLVMWRDWEESRQMVGALLEDRAHQHLVDFDCHLDDIRQDWTNQRLNTQITQWVGPTNGNGNA

>NM_001346877.1 Homo sapiens ER membrane protein complex subunit 9 (EMC9), transcript variant 5, mRNA
GTCGCGGCCGCCAGCTAGGGGCGCGGGAAGGCGGGGCTCGGATGCAATCGGGACCTCCTCCTGGACTGGG
CCGGGGGCGGACTCCGGGACCCAGGGCGCCGGGAGCCGGCGGGCTACCTGCGAGTCGAGTTAGCGTTGTC
GCCGAACCGAAGTGAGAGGGCACGGGGAAGGGTCAAAGAGCGTGCCGAAGCGCTGGAGGGAGCTTCACGG
ACGCGAGCTAGGCACCGGCTCGCCTAATCCGGTGGATGTGTGGGGAGCACAGGCCGGTCTGGTGGTGGCT
GGTTACTACCATGCCAATGCAGCTGTGAACGATCAGAGCAATATCTGAGCACCTGTCTCCCACAGCACTC
AAGCATCAAGCAGTCGTGCATCAAGCATCCAGCAGGCCCGATCCCCGCAGGCCACCTACAGGGAAAGGCC
ACAGCTGCGCCCACACCTATGAGACCTGGGAGGCCCAGCTCAAATGGATGGGTTTTGCCCACAGTTGGCT
CCTCACTCCTCTGATCTTCCCTCTACACAGCCCTGGGCCCCTGGCCTTGAAAATTGCTGGGCGAATTGCA
GAATTCTTCCCTGATGCAGTACTTATTATGTTGGATAATCAGAAACTGGTGCCTCAGCCTCGTGTGCCCC
CGGTCATCGTCCTGGAGAACCAAGGTCTCCGCTGGGTCCCTAAGGATAAGAACTTAGTGATGTGGAGGGA
CTGGGAAGAGTCACGGCAGATGGTGGGAGCTCTACTGGAAGATCGGGCCCACCAGCACCTTGTGGACTTT
GACTGCCACCTTGATGACATCCGGCAGGACTGGACCAACCAGCGGCTCAACACTCAAATCACCCAGTGGG
TTGGTCCCACTAATGGAAATGGAAATGCCTGAGCCAGGGCCAGCGGGGCCCGGTTCCAATAAAGAGACTT
GGGCTGA
>NP_001333806.1 ER membrane protein complex subunit 9 isoform b precursor [Homo sapiens]
MGFAHSWLLTPLIFPLHSPGPLALKIAGRIAEFFPDAVLIMLDNQKLVPQPRVPPVIVLENQGLRWVPKD

```
KNLVMWRDWEESRQMVGALLEDRAHQHLVDFDCHLDDIRQDWTNQRLNTQITQWVGPTNGNGNA
>NM_001346874.1 Homo sapiens ER membrane protein complex subunit 9 (EMC9), transcript variant 2, mRNA
GTCGCGGCCGCCAGCTAGGGGCGCGGGAAGGCGGGGCTCGGATGCAATCGGGACCTCCTCCTGGACTGGG
CCGGGGGCGGACTCCGGGACCCAGGGCGCCGGGAGCCGGCGGGCTACCTGCGAGTCGAGTTAGCGTTGTC
GCCGAACCGAAGCCTCGCTCGCCATGGGGGAGGTGGAGATCTCGGCCCTGGCCTACGTGAAGATGTGCCT
GCATGCTGCCCGGTACCCACACGCCGCAGTCAACGGGCTGTTTTTGGCGCCAGCGCCGCGGTCTGGAGAA
TGCCTGTGCCTCACCGACTGTGTGCCCCTCTTCCACAGCCACCTGGCCCTGTCCGTCATGTTGGAGGTCG
CCCTCAACCAGGTGGATGTGTGGGGAGCACAGGCCGGTCTGGTGGTGGCTGGTTACTACCATGCCAATGC
AGCTGTGAACGATCAGAGCAATATCTGAGCACCTGTCTCCCACAGCACTCAAGCATCAAGCAGTCGTGCA
TCAAGCATCCAGCAGGCCCGATCCCCGCAGGCCACCTACAGGGAAAGGCCACAGCTGCGCCCACACCTAT
GAGACCTGGGAGGCCCAGCTCAAATGGATGGGTTTTGCCCACAGTTGGCTCCTCACTCCTCTGATCTTCC
CTCTACACAGCCCTGGGCCCCTGGCCTTGAAAATTGCTGGGCGAATTGCAGAATTCTTCCCTGATGCAGT
ACTTATTATGTTGGATAATCAGAAACTGGTGCCTCAGCCTCGTGTGCCCCCGGTCATCGTCCTGGAGAAC
CAAGGTCTCCGCTGGGTCCCTAAGGATAAGAACTTAGTGATGTGGAGGGACTGGGAAGAGTCACGGCAGA
TGGTGGGAGCTCTACTGGAAGATCGGGCCCACCAGCACCTTGTGGACTTTGACTGCCACCTTGATGACAT
CCGGCAGGACTGGACCAACCAGCGGCTCAACACTCAAATCACCCAGTGGGTTGGTCCCACTAATGGAAAT
GGAAATGCCTGAGCCAGGGCCAGCGGGGCCCGGTTCCAATAAAGAGACTTGGGCTGA
>NP_001333803.1 ER membrane protein complex subunit 9 isoform b precursor [Homo sapiens]
MGFAHSWLLTPLIFPLHSPGPLALKIAGRIAEFFPDAVLIMLDNQKLVPQPRVPPVIVLENQGLRWVPKD
KNLVMWRDWEESRQMVGALLEDRAHQHLVDFDCHLDDIRQDWTNQRLNTQITQWVGPTNGNGNA
```

```
i1_HQ_samplec9816d|c6322/f2p3/1046      gtccgggacccagggcgccgggagccggcgggctacctgcgagtcgagttagcgttgtcg     60
i0_HQ_samplec9816d|c1265/f10p11/810     ------------------------------------------------------------     0
NM_001346877.1                          ------------------GTCGCGGCCGCCAGCTAGGGGCGCGG----------------     26
NM_016049.3                             ------------------GTCGCGGCCGCCAGCTAGGGGCGCGG----------------     26
i0_HQ_samplec9816d|c2150/f3p12/894      ------------------------------------------------------------     0
NM_001346874.1                          ------------------GTCGCGGCCGCCAGCTAGGGGCGCGG----------------     26

i1_HQ_samplec9816d|c6322/f2p3/1046      CCGAACCGAAGTGAGAGGGCACGGGGAAGGGTCAAAGAGCGTGCCGAAGCGCTGGAGGGA     120
i0_HQ_samplec9816d|c1265/f10p11/810     CCGAACCGAAGTGAGAGGGCACGGGGAAGGGTCAAAGAGCGTGCCGAAGCGCTGGAGGGA     60
NM_001346877.1                          GAAGGCGGGGCTCGGATGCAATCGGGACCT------CCTCCTGGACTGGGCCGGGGGCGG     80
NM_016049.3                             GAAGGCGGGGCTCGGATGCAATCGGGACCT------CCTCCTGGACTGGGCCGGGGGCGG     80
i0_HQ_samplec9816d|c2150/f3p12/894      -----------------------------------------------------ggggcgg     7
NM_001346874.1                          GAAGGCGGGGCTCGGATGCAATCGGGACCT------CCTCCTGGACTGGGCCGGGGGCGG     80
                                                                                     ** * *

i1_HQ_samplec9816d|c6322/f2p3/1046      GCTTCACGGA--CGCGAGCTAGGCACCGGCTCGCCTAATCCGGTACTAATCCGGCTTGCT     178
i0_HQ_samplec9816d|c1265/f10p11/810     GCTTCACGGA--CGCGAGCTAGGCACCGGCTCGCCTAATCCGGTACTAATCCGGCTTGCT     118
NM_001346877.1                          ACTCCGGGACCCAGGGCGCCGGGAGCCGGCGGGCTACCTGCGAG--TCGAGTTAGCGTTG     138
NM_016049.3                             ACTCCGGGACCCAGGGCGCCGGGAGCCGGCGGGCTACCTGCGAG--TCGAGTTAGCGTTG     138
i0_HQ_samplec9816d|c2150/f3p12/894      actccgggacccagggcgccgggagccggcgggctacctgcgag--tcgagttagcgtTG     65
NM_001346874.1                          ACTCCGGGACCCAGGGCGCCGGGAGCCGGCGGGCTACCTGCGAG--TCGAGTTAGCGTTG     138
                                         ** *  *     * * **  **  *****  **    * **    *

i1_HQ_samplec9816d|c6322/f2p3/1046      GCTTCCCGTCCAGGCCTCGCTCGCCATGGGGGAGGTGGAGATCTCGGCCCTGGCCTACGT     238
i0_HQ_samplec9816d|c1265/f10p11/810     GCTTCCCGTCCAGGCCTCGCTCGCCATGGGGGAGGTGGAGATCTCGGCCCTGGCCTACGT     178
NM_001346877.1                          TCGCCGAACCGAAGTGAGAGGGCACGGGGAAGGGTCAAAGAGCGTGCCGA----------     188
NM_016049.3                             TCGCCGAACCGAAGCCTCGCTCGCCATGGGGGAGGTGGAGATCTCGGCCCTGGCCTACGT     198
i0_HQ_samplec9816d|c2150/f3p12/894      TCGCCGAACCGAAGCCTCGCTCGCCATGGGGGAGGTGGAGATCTCGGCCCTGGCCTACGT     125
NM_001346874.1                          TCGCCGAACCGAAGCCTCGCTCGCCATGGGGGAGGTGGAGATCTCGGCCCTGGCCTACGT     198
                                         *  *    * * *          *  **  * *    *** *  * *

i1_HQ_samplec9816d|c6322/f2p3/1046      GAAGATGTGCCTGCATGCTGCCCGGTACCCACACGCCGCAGTCAACGGGCTGTTTTTGGC     298
i0_HQ_samplec9816d|c1265/f10p11/810     GAAGATGTGCCTGCATGCTGCCCGGTACCCACACGCCGCAGTCAACGGGCTGTTTTTGGC     238
NM_001346877.1                          ------------------------------------------------------------     188
NM_016049.3                             GAAGATGTGCCTGCATGCTGCCCGGTACCCACACGCCGCAGTCAACGGGCTGTTTTTGGC     258
i0_HQ_samplec9816d|c2150/f3p12/894      GAAGATGTGCCTGCATGCTGCCCGGTACCCACACGCCGCAGTCAACGGGCTGTTTTTGGC     185
NM_001346874.1                          GAAGATGTGCCTGCATGCTGCCCGGTACCCACACGCCGCAGTCAACGGGCTGTTTTTGGC     258

i1_HQ_samplec9816d|c6322/f2p3/1046      GCCAGCGCCGCGGTCTGGAGAATGCCTGTGCCTCACCGACTGTGTGCCCCTCTTCCACAG     358
i0_HQ_samplec9816d|c1265/f10p11/810     GCCAGCGCCGCGGTCTGGAGAATGCCTGTGCCTCACCGACTGTGTGCCCCTCTTCCACAG     298
NM_001346877.1                          --------------------------------------------------AGCGCTGGAG     198
NM_016049.3                             GCCAGCGCCGCGGTCTGGAGAATGCCTGTGCCTCACCGACTGTGTGCCCCTCTTCCACAG     318
i0_HQ_samplec9816d|c2150/f3p12/894      GCCAGCGCCGCGGTCTGGAGAATGCCTGTGCCTCACCGACTGTGTGCCCCTCTTCCACAG     245
NM_001346874.1                          GCCAGCGCCGCGGTCTGGAGAATGCCTGTGCCTCACCGACTGTGTGCCCCTCTTCCACAG     318
                                                                                      *   **

i1_HQ_samplec9816d|c6322/f2p3/1046      CCACCTGGCCCTGTCCGTCATGTTGGAGGTCGCCCTCAACCAGGTGGATGTGTGGGGAGC     418
i0_HQ_samplec9816d|c1265/f10p11/810     CCACCTGGCCCTGTCCGTCATGTTGGAGGTCGCCCTCAACCAGGTGGATGTGTGGGGAGC     358
NM_001346877.1                          GGAGCTTCACGGACGCGAGCTAGGCACCGGCTCGCCTAATCCGGTGGATGTGTGGGGAGC     258
NM_016049.3                             CCACCTGGCCCTGTCCGTCATGTTGGAGGTCGCCCTCAACCAGGTGGATGTGTGGGGAGC     378
i0_HQ_samplec9816d|c2150/f3p12/894      CCACCTGGCCCTGTCCGTCATGTTGGAGGTCGCCCTCAACCAGGTGGATGTGTGGGGAGC     305
NM_001346874.1                          CCACCTGGCCCTGTCCGTCATGTTGGAGGTCGCCCTCAACCAGGTGGATGTGTGGGGAGC     378
                                          * **    *     **    *       * * * *  ** * *****************

i1_HQ_samplec9816d|c6322/f2p3/1046      ACAGGCCGGTCTGGTGGTGGCTGGTTACTACCATGCCAATGCAGCTGTGAACGATCAGAG     478
i0_HQ_samplec9816d|c1265/f10p11/810     ACAGGCCGGTCTGGTGGTGGCTGGTTACTACCATGCCAATGCAGCTGTGAACGATCAGAG     418
NM_001346877.1                          ACAGGCCGGTCTGGTGGTGGCTGGTTACTACCATGCCAATGCAGCTGTGAACGATCAGAG     318
NM_016049.3                             ACAGGCCGGTCTGGTGGTGGCTGGTTACTACCATGCCAATGCAGCTGTGAACGATCAGA-     437
i0_HQ_samplec9816d|c2150/f3p12/894      ACAGGCCGGTCTGGTGGTGGCTGGTTACTACCATGCCAATGCAGCTGTGAACGATCAGAG     365
NM_001346874.1                          ACAGGCCGGTCTGGTGGTGGCTGGTTACTACCATGCCAATGCAGCTGTGAACGATCAGAG     438
                                        ***********************************************************

i1_HQ_samplec9816d|c6322/f2p3/1046      C---------------------------ACTCAAGCATCAAGCAGTCGTGCATCAAGCAT     511
i0_HQ_samplec9816d|c1265/f10p11/810     ------------------------------------------------------------     418
NM_001346877.1                          CAATATCTGAGCACCTGTCTCCCACAGCACTCAAGCATCAAGCAGTCGTGCATCAAGCAT     378
```

```
NM_016049.3                             ------------------------------------------------------------     437
i0_HQ_samplec9816d|c2150/f3p12/894      ---------------------------CACTCAAGCATCAAGCAGTCGTGCATCAAGCAT     398
NM_001346874.1                          CAATATCTGAGCACCTGTCTCCCACAGCACTCAAGCATCAAGCAGTCGTGCATCAAGCAT     498

i1_HQ_samplec9816d|c6322/f2p3/1046      CCAGCAGGCCCGATCCCCGCAGGCCACCTACAGGGAAAGGCCACAGCTGCGCCCACACCT     571
i0_HQ_samplec9816d|c1265/f10p11/810     ------------------------------------------------------------     418
NM_001346877.1                          CCAGCAGGCCCGATCCCCGCAGGCCACCTACAGGGAAAGGCCACAGCTGCGCCCACACCT     438
NM_016049.3                             ------------------------------------------------------------     437
i0_HQ_samplec9816d|c2150/f3p12/894      CCAGCAGGCCCGATCCCCGCAGGCCACCTACAGGGAAAGGCCACAGCTGCGCCCACACCT     458
NM_001346874.1                          CCAGCAGGCCCGATCCCCGCAGGCCACCTACAGGGAAAGGCCACAGCTGCGCCCACACCT     558

i1_HQ_samplec9816d|c6322/f2p3/1046      ATGAGACCTGGGAGGCCCAGCTCAAATGGATGGGTTTTGCCCACAGTTGGCTCCTCACTC     631
i0_HQ_samplec9816d|c1265/f10p11/810     ------------------------------------------------------------     418
NM_001346877.1                          ATGAGACCTGGGAGGCCCAGCTCAAATGGATGGGTTTTGCCCACAGTTGGCTCCTCACTC     498
NM_016049.3                             ------------------------------------------------------------     437
i0_HQ_samplec9816d|c2150/f3p12/894      ATGAGACCTGGGAGGCCCAGCTCAAATGGATGGGTTTTGCCCGC----------------     502
NM_001346874.1                          ATGAGACCTGGGAGGCCCAGCTCAAATGGATGGGTTTTGCCCACAGTTGGCTCCTCACTC     618

i1_HQ_samplec9816d|c6322/f2p3/1046      CTCTGATCTTCCCTCTACACAGCCCTGGGCCCCTGGCCTTGAAAATTGCTGGGCGAATTG     691
i0_HQ_samplec9816d|c1265/f10p11/810     ----------------------CCCTGGGCCCCTGGCCTTGAAAATTGCTGGGCGAATTG     456
NM_001346877.1                          CTCTGATCTTCCCTCTACACAGCCCTGGGCCCCTGGCCTTGAAAATTGCTGGGCGAATTG     558
NM_016049.3                             ---------------------GCCCTGGGCCCCTGGCCTTGAAAATTGCTGGGCGAATTG     476
i0_HQ_samplec9816d|c2150/f3p12/894      -----------------------CCTGGGCCCCTGGCCTTGAAAATTGCTGGGCGAATTG     539
NM_001346874.1                          CTCTGATCTTCCCTCTACACAGCCCTGGGCCCCTGGCCTTGAAAATTGCTGGGCGAATTG     678
                                                               *************************************

i1_HQ_samplec9816d|c6322/f2p3/1046      CAGAATTCTTCCCTGATGCAGTACTTATTATGTTGGATAATCAGAAACTGGTGCCTCAGC     751
i0_HQ_samplec9816d|c1265/f10p11/810     CAGAATTCTTCCCTGATGCAGTACTTATTATGTTGGATAATCAGAAACTGGTGCCTCAGC     516
NM_001346877.1                          CAGAATTCTTCCCTGATGCAGTACTTATTATGTTGGATAATCAGAAACTGGTGCCTCAGC     618
NM_016049.3                             CAGAATTCTTCCCTGATGCAGTACTTATTATGTTGGATAATCAGAAACTGGTGCCTCAGC     536
i0_HQ_samplec9816d|c2150/f3p12/894      CAGAATTCTTCCCTGATGCAGTACTTATTATGTTGGATAATCAGAAACTGGTGCCTCAGC     599
NM_001346874.1                          CAGAATTCTTCCCTGATGCAGTACTTATTATGTTGGATAATCAGAAACTGGTGCCTCAGC     738
                                        ************************************************************

i1_HQ_samplec9816d|c6322/f2p3/1046      CTCGTGTGCCCCCGGTCATCGTCCTGGAGAACCAAGGTCTCCGCTGGGTCCCTAAGGATA     811
i0_HQ_samplec9816d|c1265/f10p11/810     CTCGTGTGCCCCCGGTCATCGTCCTGGAGAACCAAGGTCTCCGCTGGGTCCCTAAGGATA     576
NM_001346877.1                          CTCGTGTGCCCCCGGTCATCGTCCTGGAGAACCAAGGTCTCCGCTGGGTCCCTAAGGATA     678
NM_016049.3                             CTCGTGTGCCCCCGGTCATCGTCCTGGAGAACCAAGGTCTCCGCTGGGTCCCTAAGGATA     596
i0_HQ_samplec9816d|c2150/f3p12/894      CTCGTGTGCCCCCGGTCATCGTCCTGGAGAACCAAGGTCTCCGCTGGGTCCCTAAGGATA     659
NM_001346874.1                          CTCGTGTGCCCCCGGTCATCGTCCTGGAGAACCAAGGTCTCCGCTGGGTCCCTAAGGATA     798
                                        ************************************************************

i1_HQ_samplec9816d|c6322/f2p3/1046      AGAACTTAGTGATGTGGAGGGACTGGGAAGAGTCACGGCAGATGGTGGGAGCTCTACTGG     871
i0_HQ_samplec9816d|c1265/f10p11/810     AGAACTTAGTGATGTGGAGGGACTGGGAAGAGTCACGGCAGATGGTGGGAGCTCTACTGG     636
NM_001346877.1                          AGAACTTAGTGATGTGGAGGGACTGGGAAGAGTCACGGCAGATGGTGGGAGCTCTACTGG     738
NM_016049.3                             AGAACTTAGTGATGTGGAGGGACTGGGAAGAGTCACGGCAGATGGTGGGAGCTCTACTGG     656
i0_HQ_samplec9816d|c2150/f3p12/894      AGAACTTAGTGATGTGGAGGGACTGGGAAGAGTCACGGCAGATGGTGGGAGCTCTACTGG     719
NM_001346874.1                          AGAACTTAGTGATGTGGAGGGACTGGGAAGAGTCACGGCAGATGGTGGGAGCTCTACTGG     858
                                        ************************************************************

i1_HQ_samplec9816d|c6322/f2p3/1046      AAGATCGGGCCCACCAGCACCTTGTGGACTTTGACTGCCACCTTGATGACATCCGGCAGG     931
i0_HQ_samplec9816d|c1265/f10p11/810     AAGATCGGGCCCACCAGCACCTTGTGGACTTTGACTGCCACCTTGATGACATCCGGCAGG     696
NM_001346877.1                          AAGATCGGGCCCACCAGCACCTTGTGGACTTTGACTGCCACCTTGATGACATCCGGCAGG     798
NM_016049.3                             AAGATCGGGCCCACCAGCACCTTGTGGACTTTGACTGCCACCTTGATGACATCCGGCAGG     716
i0_HQ_samplec9816d|c2150/f3p12/894      AAGATCGGGCCCACCAGCACCTTGTGGACTTTGACTGCCACCTTGATGACATCCGGCAGG     779
NM_001346874.1                          AAGATCGGGCCCACCAGCACCTTGTGGACTTTGACTGCCACCTTGATGACATCCGGCAGG     918
                                        ************************************************************

i1_HQ_samplec9816d|c6322/f2p3/1046      ACTGGACCAACCAGCGGCTCAACACTCAAATCACCCAGTGGGTTGGTCCCACTAATGGAA     991
i0_HQ_samplec9816d|c1265/f10p11/810     ACTGGACCAACCAGCGGCTCAACACTCAAATCACCCAGTGGGTTGGTCCCACTAATGGAA     756
NM_001346877.1                          ACTGGACCAACCAGCGGCTCAACACTCAAATCACCCAGTGGGTTGGTCCCACTAATGGAA     858
NM_016049.3                             ACTGGACCAACCAGCGGCTCAACACTCAAATCACCCAGTGGGTTGGTCCCACTAATGGAA     776
i0_HQ_samplec9816d|c2150/f3p12/894      ACTGGACCAACCAGCGGCTCAACACTCAAATCACCCAGTGGGTTGGTCCCACTAATGGAA     839
NM_001346874.1                          ACTGGACCAACCAGCGGCTCAACACTCAAATCACCCAGTGGGTTGGTCCCACTAATGGAA     978
                                        ************************************************************

i1_HQ_samplec9816d|c6322/f2p3/1046      ATGGAAATGCCTGAGCCAGggccagcggggcccggttccaataaagagacttggg-----     1046
i0_HQ_samplec9816d|c1265/f10p11/810     ATGGAAATGCCTGAGCCAGGGCCAGCGGGGCCCGGTTCCAATAAAGAGACTTGG------     810
NM_001346877.1                          ATGGAAATGCCTGAGCCAGGGCCAGCGGGGCCCGGTTCCAATAAAGAGACTTGGGCTGA-     917
NM_016049.3                             ATGGAAATGCCTGAGCCAGGGCCAGCGGGGCCCGGTTCCAATAAAGAGACTTGGGCTGAA     836
i0_HQ_samplec9816d|c2150/f3p12/894      ATGGAAATGCCTGAGCCAGGGCCAGCGGGGCCCGGTTCCAATAAAGAGACTTGGG-----     894
NM_001346874.1                          ATGGAAATGCCTGAGCCAGGGCCAGCGGGGCCCGGTTCCAATAAAGAGACTTGGGCTGA-     1037
                                        ******************************************************

i1_HQ_samplec9816d|c6322/f2p3/1046      ------------------------------------------------------------     1046
i0_HQ_samplec9816d|c1265/f10p11/810     ------------------------------------------------------------     810
NM_001346877.1                          ------------------------------------------------------------     917
NM_016049.3                             AAAAAAAAAAAAAAAAAAAAAAAAAAAAAAAAAAAAAAAAAAAAAAAAAAAAAAAAAAAA     896
i0_HQ_samplec9816d|c2150/f3p12/894      ------------------------------------------------------------     894
NM_001346874.1                          ------------------------------------------------------------     1037
```

```
VIRT130505          MGEVEISALAYVKMCLHAARYPHAAVNGLFLAPAPRSGECLCLTDCVPLFHSHLALSVML  60
VIRT147453          ------------------------------------------------------------  0
NP_001333806.1      ------------------------------------------------------------  0
NP_001333803.1      ------------------------------------------------------------  0
VIRT151133          MGEVEISALAYVKMCLHAARYPHAAVNGLFLAPAPRSGECLCLTDCVPLFHSHLALSVML  60
NP_057133.2         MGEVEISALAYVKMCLHAARYPHAAVNGLFLAPAPRSGECLCLTDCVPLFHSHLALSVML  60
```

```
VIRT130505          EVALNQVDVWGAQAGLVVAGYYHANAAVNDQSTQASSSRASSIQQARSPQAT-------- 112
VIRT147453          -------------MGF-AHSWLLTPLIFPLHSPGPLALKIAGRIAEFFPDAVLIMLDNQK 46
NP_001333806.1      -------------MGF-AHSWLLTPLIFPLHSPGPLALKIAGRIAEFFPDAVLIMLDNQK 46
NP_001333803.1      -------------MGF-AHSWLLTPLIFPLHSPGPLALKIAGRIAEFFPDAVLIMLDNQK 46
VIRT151133          EVALNQVDVWGAQAGLVVAGYYHANAAVNDQSPGPLALKIAGRIAEFFPDAVLIMLDNQK 120
NP_057133.2         EVALNQVDVWGAQAGLVVAGYYHANAAVNDQSPGPLALKIAGRIAEFFPDAVLIMLDNQK 120
                                  *: . .:  :   .  :*    : : :.      *:*.

VIRT130505          YRERPQLRPHL------------------------------------------------- 123
VIRT147453          LVPQPRVPPVIVLENQGLRWVPKDKNLVMWRDWEESRQMVGALLEDRAHQHLVDFDCHLD 106
NP_001333806.1      LVPQPRVPPVIVLENQGLRWVPKDKNLVMWRDWEESRQMVGALLEDRAHQHLVDFDCHLD 106
NP_001333803.1      LVPQPRVPPVIVLENQGLRWVPKDKNLVMWRDWEESRQMVGALLEDRAHQHLVDFDCHLD 106
VIRT151133          LVPQPRVPPVIVLENQGLRWVPKDKNLVMWRDWEESRQMVGALLEDRAHQHLVDFDCHLD 180
NP_057133.2         LVPQPRVPPVIVLENQGLRWVPKDKNLVMWRDWEESRQMVGALLEDRAHQHLVDFDCHLD 180
                       :*:: * :

VIRT130505          ----------------------------   123
VIRT147453          DIRQDWTNQRLNTQITQWVGPTNGNGNA   134
NP_001333806.1      DIRQDWTNQRLNTQITQWVGPTNGNGNA   134
NP_001333803.1      DIRQDWTNQRLNTQITQWVGPTNGNGNA   134
VIRT151133          DIRQDWTNQRLNTQITQWVGPTNGNGNA   208
NP_057133.2         DIRQDWTNQRLNTQITQWVGPTNGNGNA   208
```
